# Transcriptomics of diapause in an isogenic self-fertilizing vertebrate

**DOI:** 10.1186/s12864-015-2210-0

**Published:** 2015-11-23

**Authors:** Felix Mesak, Andrey Tatarenkov, John C. Avise

**Affiliations:** Department of Ecology and Evolutionary Biology, University of California, Irvine, CA 92697-2525 USA; Department of Ecology and Evolutionary Biology, Ayala School of Biological Sciences, University of California, Irvine, CA 92697-2525 USA

**Keywords:** RNA-Seq, Genome sequencing, Transcriptome, Diapause, *Kryptolebias marmoratus*, Self-fertilization, Isogenic

## Abstract

**Background:**

Many vertebrate species have the ability to undergo weeks or even months of diapause (a temporary arrest of development during early ontogeny). Identification of diapause genes has been challenging due in part to the genetic heterogeneity of most vertebrate animals.

**Results:**

Here we take the advantage of the mangrove rivulus fish (*Kryptolebias marmoratus* or Kmar)—the only vertebrate that is extremely inbred due to consistent self-fertilization—to generate isogenic lineages for transcriptomic dissection. Because the Kmar genome is not publicly available, we built *de novo* genomic (642 Mb) and transcriptomic assemblies to serve as references for global genetic profiling of diapause in Kmar, via RNA-Seq. Transcripts unique to diapause in Kmar proved to constitute only a miniscule fraction (0.1 %) of the total pool of transcribed products. Most genes displayed lower expression in diapause than in post-diapause. However, some genes (notably *dusp27*, *klhl38* and *sqstm1*) were significantly up-regulated during diapause, whereas others (*col9a1*, *dspp* and *fmnl1*) were substantially down-regulated, compared to both pre-diapause and post-diapause.

**Conclusion:**

Kmar offers a strong model for understanding patterns of gene expression during diapause. Our study highlights the importance of using a combination of genome and transcriptome assemblies as references for NGS-based RNA-Seq analyses. As for all identified diapause genes, in future studies it will be critical to link various levels of RNA expression with the functional roles of the coded products.

**Electronic supplementary material:**

The online version of this article (doi:10.1186/s12864-015-2210-0) contains supplementary material, which is available to authorized users.

## Background

Many organisms have the ability to enter a temporary state of suspended animation before resuming the normal progression of life. Various terms describe the phenomenon in specific situations in nature: (i) embryonic diapause, in which an embryo’s development is temporarily arrested during early ontogeny [1]; (ii) torpor, when body temperature and metabolic rate in endothermic animals are significantly reduced during certain times of the day [2]; (iii) hibernation or multiday torpor, as utilized by various animals to escape harsh winter conditions [3, 4]; (iv) aestivation, when multiday torpor occurs in hot or dry seasons in warm climates [5]; and (v) cryptobiosis, which entails a temporary absence of measurable metabolic activity [6, 7]. In this study, we will use the term “diapause” when it occurs during embryonic development. Other terms sometimes used to describe this phenomenon in Kmar are “delayed hatching” or “embryonic quiescence”.

Diapause was first described in roe deer in 1854, where blastocyst development and subsequent implantation in the uterus were delayed for 4–5 months [8]. Similar phenomena have since been discovered in more than 130 species of mammals [1, 9, 10]. Remarkably, mammalian embryonic diapause can last up to 90 % of the total gestational period [11]. Another form of diapause is displayed by some egg-laying fishes in which embryonic hatching can be delayed for a year or more. Such fish may display any of three different types of diapause: Diapause I, occurring during the dispersed-cell phase of early ontogeny; Diapause II, occurring mid-somite embryogenesis; and Diapause III, occurring just prior to hatching [12, 13].

Whereas the role of environmental cues in triggering diapause in killifish or more generally in vertebrates is well established [1, 13], biological mechanisms underlying the phenomenon have yet to be elucidated. For example, there is no consensus on how patterns of gene expression change during diapause. This problem promised to resolve following two major breakthroughs in the measurement of gene expression: (i) cDNA microarray technology in the mid-1990s and (ii) next generation sequencing (NGS) based RNA-Sequencing (RNA-Seq) technology in the mid-2000s [14, 15]. Indeed, about 40 studies used cDNA microarrays or RNA-Seq to assay biological samples undergoing some form of suspended animation, and many more papers have addressed the diapause syndrome more generally. RNA-Seq is superior to cDNA microarray in that it allows: (i) the capture of whole-genome expression (transcriptomics); (ii) execution without prior knowledge of annotated genes; and (iii) the detection and quantification of low-abundance genes or those with higher-fold changes of expression [16]. The use of RNA-Seq to determine genes with distinct levels of expression during diapause previously has been confined to invertebrates (mainly insects) [17–19].

**Fig. 1:**
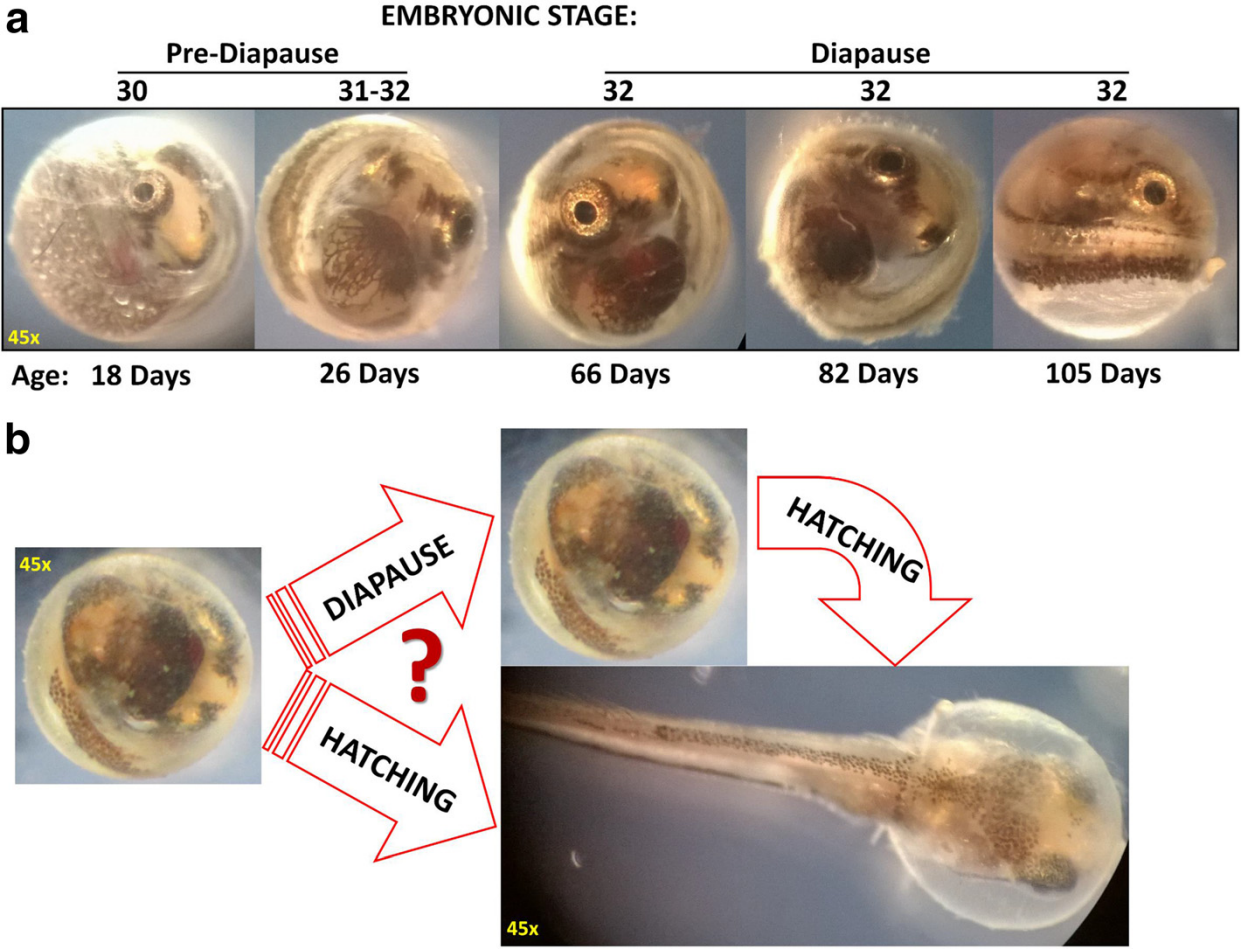
Images of diapause or hatched Kmar embryos. **a** Each Kmar embryo enters diapause at stage 32, which is marked by the diminished presence of nutritional oil droplets (bubble features clearly visible at stage 30). Any prolongation of embryonic stage 32 beyond 24 days is considered diapause. **b** Kmar embryos can either enter diapause or hatch (the biological mechanism that allows an embryo to enter and stay in diapause instead of hatching is unknown)

To address this issue, we take advantage of the unique mating/reproductive system of the mangrove rivulus fish (*Kryptolebias marmoratus* or Kmar) - the world’s only vertebrate species that is highly inbred due to consistent self-fertilization (selfing). We generate isogenic lineages for transcriptomic dissection of one form of diapause. In this hermaphroditic species, selfing repeated across successive generations leads to a rapid decay of heterozygosity and to the rise of isogenic lines that are effectively “clonal” [20, 21]. In captivity, Kmar survives well in 25 ppt saltwater, where >90 % of Kmar embryos enter diapause at stage 32 (the last stage prior to hatching when nutritional oil droplets are depleted) (Fig. 1a). In Kmar, the average duration of embryonic stages 1–32 is 24 days. However, in the laboratory we regularly observe Kmar embryos remaining in stage 32 (diapause) for up to 2.5 months. We suggest that Kmar offers an excellent model to understand the biology of diapause because of its genetic homogeneity, simple rearing environment, translucent externally developing embryos, and relatively straightforward form of diapause. Here, we report the use of next generation sequencing and RNA-Seq to distinguish the Kmar diapause transcriptome from those of pre-diapause and post-diapause (hatched) embryos (Fig. 1a, b). Since a Kmar genome sequence was not publicly available, we sequenced the whole genome of this species. We then built *de novo* genome assemblies to serve as a genomic reference for the RNA-Seq data. We also built *de novo* transcriptome assemblies to further capture and validate the RNA-Seq data. Differentially expressed genes unique to diapause in Kmar are herein identified and discussed.

## Results

### Mapping of Kmar RNA-Seq reads to known reference genomes

**Fig. 2:**
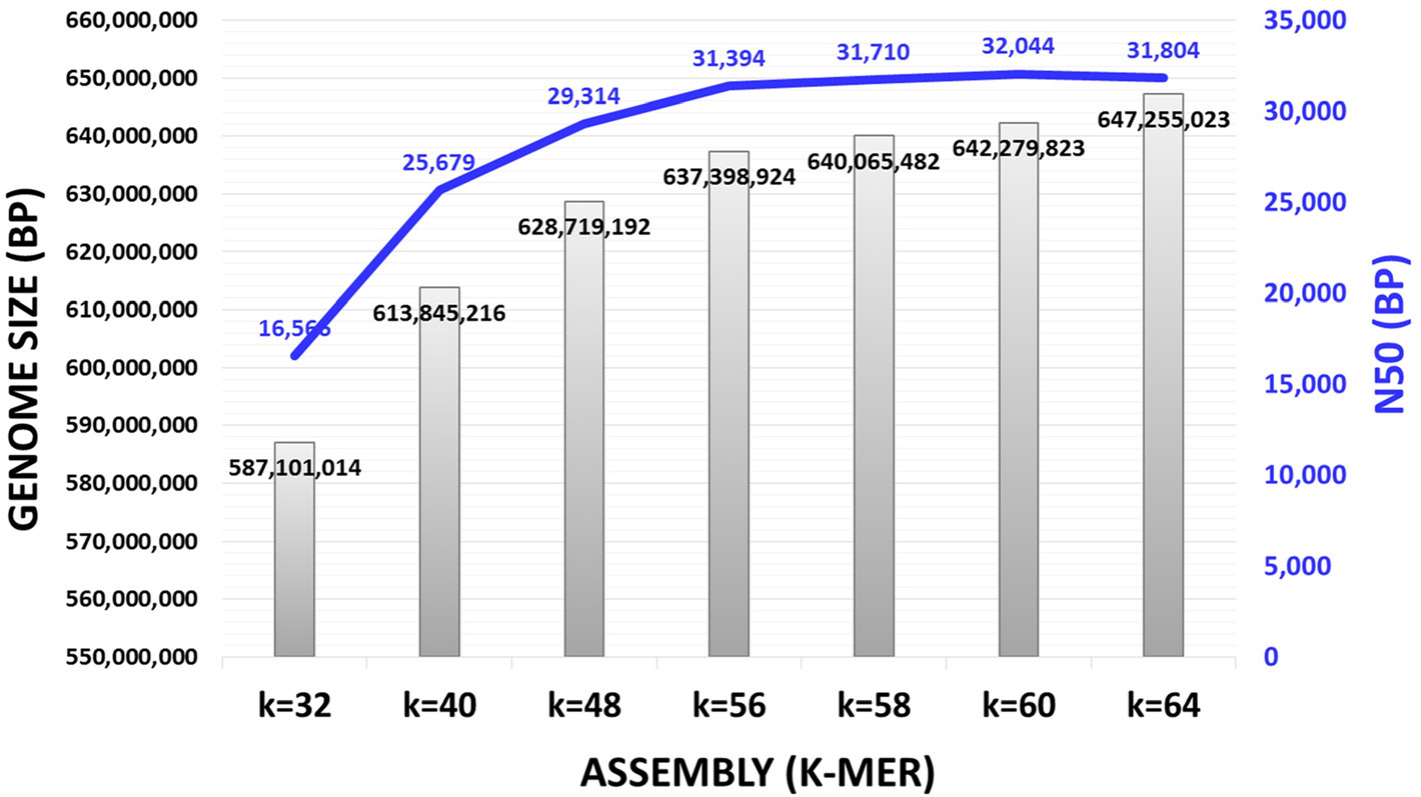
Optimization of Kmar *de novo* genome assemblies. Raw reads for pair-ended Kmar WGS were assembled at various k-mer values to build a *de novo* genome. The graph shows a positive correlation between higher k-mer values and Kmar genome size. A similar correlation was found for N50, but peaked at k = 60. Thus, a Kmar genome with the highest N50 value (32,044 bp) that yielded a genome size of 642,279,823 bp was selected to serve as a reference genome for the RNA-Seq data analyses. [Note: k-mer at higher than k = 64 failed to assemble a *de novo* genome]

By using TopHat software [22], we found that less than 10 % of Kmar RNA-Seq reads could be mapped against the annotated genomes of humans, mice, and several fishes (Amazon molly, platyfish, medaka, zebrafish, and fugu) (Table 1). To increase the number of mapped reads, we sequenced the whole Kmar genome and built a *de novo* reference genome using ABySS software [23]. The genome size was determined to be 642 Mb (Fig. 2). Two additional references were built from RNA-Seq reads using Trans-ABySS and Trinity software [24, 25]. More than 89 % of the RNA-Seq reads were mapped against these three Kmar references (Table 1). The references were used as templates to identify transcripts. A computational program called “tuxedo suite” was utilized to identify, quantitate, and calculate differential transcript abundance between diapause and pre- or post-diapause RNA samples (Additional file 1: Table S1) [22].

**Table 1:** Alignment of Kmar RNA-Seq reads against various reference genomes

Reference Genome/Transcriptome	No. of Mapped RNA-Seq Reads:					Percentage:				
	RNA01:pre-Dia	RNA02:Dia-R1	RNA03:Dia-R2	RNA04:Dia-R3	RNA05:post-Dia	RNA01:pre-Dia	RNA02:Dia-R1	RNA03:Dia-R2	RNA04:Dia-R3	RNA05:post-Dia
Kmar *de novo* Genome (ABySS)^a^	31,590,161	39,192,211	31,524,390	32,811,042	30,944,064	97.08	97.42	97.35	97.32	96.98
Kmar *de novo* Transcriptome (Trans-ABySS)^a^	30,584,171	37,850,616	30,651,957	31,942,287	29,994,937	93.99	94.08	94.66	94.74	94.01
Kmar *de novo* Transcriptome (Trinity)^a^	29,039,228	35,715,712	29,057,499	30,264,954	28,112,433	89.24	88.78	89.74	89.77	88.11
Kmar *de novo* Partial-Genome (ABySS, RADSeq)^b^	4,078,090	4,631,901	3,720,990	3,781,213	3,789,332	12.53	11.51	11.49	11.22	11.88
Amazon Molly Genome^c^	3,735,199	4,105,696	3,022,398	3,096,027	2,577,109	11.48	10.21	9.33	9.18	8.08
Platyfish Genome^d^	3,250,075	3,733,889	2,736,897	2,824,479	2,278,491	9.99	9.28	8.45	8.38	7.14
Medaka Genome^e^	3,668,978	3,476,396	2,512,691	2,499,786	2,198,448	11.28	8.64	7.76	7.41	6.89
Zebrafish Genome^f^	1,841,434	1,552,206	1,124,437	1,073,152	982,792	5.66	3.86	3.47	3.18	3.08
Fugu Genome^g^	1,302,617	1,557,208	1,097,370	1,143,775	1,036,617	4.00	3.87	3.39	3.39	3.25
Mouse Genome^h^	1,324,000	998,352	763,984	712,118	639,505	4.07	2.48	2.36	2.11	2.00
Human Genome^i^	1,681,163	1,273,803	961,456	899,559	853,593	5.17	3.17	2.97	2.67	2.68

^a^This study;

^b^(Mesak, et al. [32]);

^c^PoeFor_5.1.2;

^d^Xipmac4.4.2 (Schartl, et al. [40]);

^e^oryLat2, MEDAKA1 (Kasahara, et al. [41]);

^f^danRer7, Zv9 (Howe, et al. [42]);

^g^fr3, FUGU4 (Aparicio, et al. [43]);

^h^mm10, GRCm38 (Mouse Genome Sequencing, et al. [44]);

^i^hg38, GRCh38 (Lander, et al. [45]; Venter, et al. [46]);

Reference genome database can be downloaded from http://www.ensembl.org/info/data/ftp/index.html or http://hgdownload.soe.ucsc.edu/downloads.html

### Mitochondrial housekeeping genes had high and stable levels of gene expression across developmental stages

**Fig. 3:**
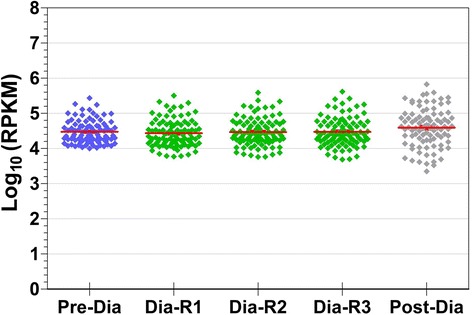
Mitochondrial housekeeping gene expression. The graph shows 93 highly abundant Kmar mitochondrial transcripts that were identified from the *de novo* genome (ABySS) assembly

We found that the level of expression of mitochondrial housekeeping genes was high and consistent across the three developmental stages surveyed in Kmar (Fig. 3, average Log_10_(RPKM) = ~4.5, RPKM: reads per kilobase per million mapped reads). These mitochondrial genes include cytochrome oxidase, ribosomal RNAs, ATPase, and NADH dehydrogenase. The fact that the expression of such housekeeping genes was not affected by developmental stage makes comparison of the expression of other genes straightforward.

### Genes with distinct levels of expression in diapause constitute a minority of surveyed genes

**Fig. 4:**
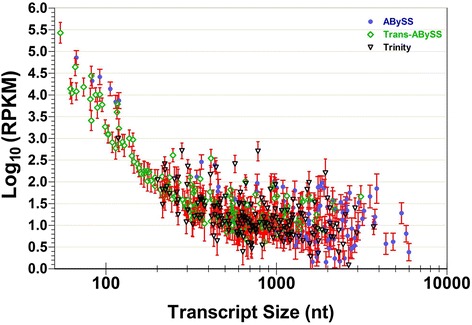
This composite graph shows differentially expressed genes in diapause versus pre- or post-diapause, as identified from the *de novo* genome and transcriptomes assemblies

We observed that >97 % of the surveyed gene transcripts were detected in all Kmar samples (Table 2), including pre-diapause, three diapause replicates, and post-diapause. Of these detected transcripts, only 0.1 % were up- and down-regulated in diapause compared to both pre- and post-diapause. Most of these latter transcripts had lengths between 200 and 4000 nt and with expression abundances in the range 0.5 < Log_10_(RPKM) < 2.5 (Fig. 4). When diapause transcripts were compared to pre-diapause transcripts, the numbers of up- and down-regulated genes were approximately equal. However, for 67 % of surveyed genes, the level of gene expression in diapause was down-regulated compared to post-diapause (Table 3).

**Table 2:** The percentages of transcripts that exist in various developmental stages of Kmar fish

Comparison	Transcripts detected in	WGS^b^ (*n* = 67,374)	RNA-Seq^c^ (*n* = 206,747)	RNA-Seq^d^ (*n* = 97,979)
Diapause vs. pre-Diapause	Diapause only	1.92 + 0.38 %	1.11 + 0.04 %	0.23 + 0.00 %
	pre-Diapause only	0.86 + 0.40 %	0.62 + 0.15 %	0.16 + 0.04 %
	both Diapause & pre-Diapause	96.94 + 0.16 %	98.01 + 0.14 %	99.59 + 0.04 %
	other^a^	0.28 + 0.14 %	0.50 + 0.15 %	0.02 + 0.00 %
Diapause vs. post-Diapause	Diapause only	1.05 + 0.48 %	1.21 + 0.09 %	0.36 + 0.00 %
	post-Diapause only	1.03 + 0.49 %	1.01 + 0.22 %	0.17 + 0.04 %
	both Diapause & post-Diapause	97.81 + 1.02 %	97.68 + 0.18 %	99.45 + 0.04 %
	other^a^	0.11 + 0.06 %	0.09 + 0.02 %	0.01 + 0.00 %

^a^transcripts exist in other developmental stage, i.e. in post-diapause for diapause vs. pre-diapause, and vice versa: in pre-diapause for diapause vs. post-diapause;

^b^assembly was built by ABySS;

^c^transcriptome was assembled by Trans-ABySS;

^d^transcriptome was assembled by Trinity

**Table 3:** Percentages of up- or down-regulated transcripts in diapause versus pre- or post-diapause

Reference	Total transcripts	No. of differentially regulated transcripts	Diapause/pre-Diapause		Diapause/post-Diapause	
			Up-regulated	Down-regulated	Up-regulated	Down-regulated
Kmar *de novo* Genome (ABySS)	67,374	120	21.67 %	9.17 %	20.00 %	66.67 %
Kmar *de novo* Transcriptome (Trans-ABySs)	206,747	198	10.61 %	11.62 %	11.62 %	78.28 %
Kmar *de novo* Transcriptome (Trinity)	97,979	176	19.89 %	15.34 %	13.64 %	69.89 %

### Most functional clusters of genes were down-regulated in diapause compared to post-diapause

**Table 4:** Clusters of genes that were up- or down-regulated during diapause

Gene Cluster	List of Genes	No. of Data^a^	Diapause versus	
			pre-Diapause	post-Diapause
Cardiac and Skeletal Muscle	*bag3, myh2, mylk2, myo18b*	21	UP^b^ (1.5)	UP (1.9)
Immune System	*a2m, c4a, hla-a, ifitml, mlfl, rhcg*	27	UP (2.4)	-^c^
Extracellular Matrices (ECM)	*tecta, clip2, comp, ctsl, col2a1, col9a1, colllal, col4a3bp, collOal, dspp, mucl, muc2, postn, tnc*	237	-	DOWN^d^ (−3.2)
Respiration	*arg2, mt-atp8, mthfd2, mtco3, egln3, gimap4, hbal, hbb, irgm, ucp2, mpo, mt-nd2, noxl, ptges3, atpla2, tbxasl*	126	-	DOWN (−1.6)
Development	*teml, adm, faml23a, litaf, celal, fmnll, igfbp4, ill2b, mkl67, matnl, rbp2, bsphl, sqstml, spaca4, eed*	114	-	DOWN (−1.7)
Other Cellular and Enzymatic Processes	*agr2, agxt2l, asmt, c100r11, calu, hhat, nans, natterin-3, odcl, zg16*	63	-	DOWN (−3.4)
Hormones	*entpd5, fshr, sult2b1, zpldl*	36	-	DOWN (−3.5)
Toxin-like Proteins	*lntx-77, lntx-id, neovtx, rtx-s II, sntx*	33	-	DOWN (−4.0)
Cytoskeleton	*actal, actxl, ckap4, filipl, nlrp2, plsl, tubb*	30	-	DOWN (−2.3)
Ion Binding	*calr, hpx, umod*	30	-	DOWN (−4.3)
Protein Modification	*camk2nl, dusp27, mkrnl, fkbp9, gls2, klhl30, klhl38, mylk4, itgblbp3, papln, fkbp9, pdia4, tgm2, tgm3, ppp5c, prss2, tat, ubb, ubl5, ubell2, uhrfl*	114	-	-
Nucleic Acid Modification	*banfl, cmpkl, eeflal, mafb, muc5ac, myc, nfe2l1, papss2, pprcl, rcll, rn7sk, rps4yl, setdbl, slc28a3, srrm2*	93	-	-
Carbohydrate Modification	*b3gnt3, chia, ctl, floll, gale, gmds, nptx2, ugt2b17*	54	-	-
Environmental Responses	*faml34b, hspala, hspbl, mss5l, ota, per2, prrtl*	51	-	-
Cell Signaling	*cirhla, fbxo32, hhat, plaur, ywha, putative GPI-anchored proteins*	30	-	-
Transporter Functions	*fabp6, kpna2, slc15a1, slclal, tapl, tm4sf1*	24	-	-
Unknown	n/a	381	-	DOWN (−2.0)

^a^No. of Data are from 3 diapause replicates and 3 genome/transcriptome references (RPKM and fold change data are listed in the Supplementary document);

^b^AVERAGE(Log2(FoldChange)) ≥ 1.5;

^c^-1.5 < AVERAGE(Log2(FoldChange)) < 1.5;

^d^AVERAGE(Log2(FoldChange))≤1.5

**Fig. 5:**
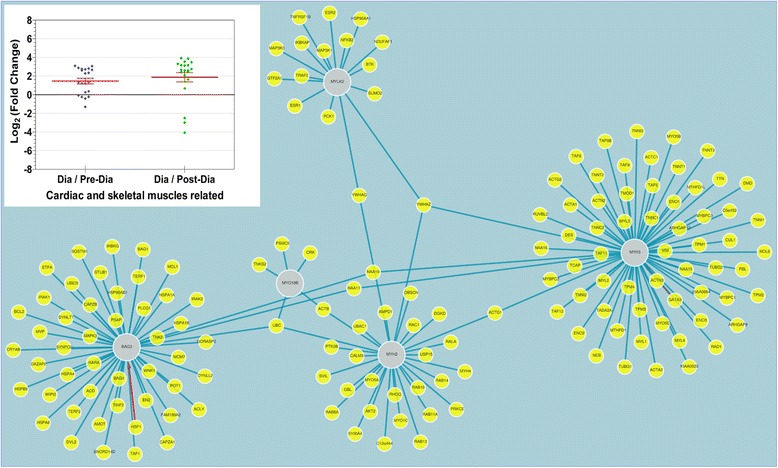
Image from interactome analysis that visualize the interaction between four genes (*myo18b, mylk2, myh2,* and *bag3*) that are related to cardiac and skeletal muscle function. Inset: the up-regulated cluster of genes related to cardiac and skeletal muscle in diapause versus both pre- and post-diapause. The graph shows fold-changes of transcript abundance

Transcripts that showed distinct abundances in Kmar diapause compared to other developmental stages were annotated and their putative biological functions were identified from a gene ontology database [26]. We identified 16 operational clusters of genes related to the following functions: extracellular matrix (ECM) (15.9 %), respiration (8.5 %), protein modification (7.7 %), development (7.7 %), nucleic acid modification (6.3 %), other cellular or enzymatic processes (4.3 %), carbohydrate modification (3.6 %), environmental responses (3.4 %), hormones (2.4 %), toxin-like proteins (2.2 %), cytoskeleton (2.0 %), cell signaling (2.0 %), ion binding (2.0 %), immune system (1.8 %), transporter functions (1.6 %), and cardiac and skeletal muscles (1.4 %). The remaining 25.7 % of transcripts with differential expression could not be assigned to any known gene. Most of these functional clusters were down-regulated in diapause compared to post-diapause, with the exception of four genes related to cardiac or skeletal muscle (*myo18b, mylk2, myh2,* and *bag3*) (Table 4, Fig. 5, Additional file 1: Table S2 ). Interestingly, an interactome analysis showed that those four loci are within the same interacting network of genes (Fig. 5) [27].

### Few genes were up- or down-regulated in diapause 

**Table 5:** Diapause genes identified from all three references

Gene Name	No. of Data^a^	Diapause versus	
		pre-Diapause	post-Diapause
Dual specificity phosphatase 27 (*dusp27*)	12	UP^b^ (2.7)	UP (2.1)
Kelch-like family member 38 (*klhl38*)	12	UP (4.8)	UP (2.4)
Sequestosome 1 (*sqstml*)	9	UP (2.1)	UP (2.5)
Collagen a-1(IX) chain-like (*col9a1*)	15	DOWN^c^ (−2.2)	DOWN (−2.6)
Dentin sialophosphoprotein-like (*dspp*)	12	DOWN (−2.3)	DOWN (−2.3)
Formin-like protein (*fmnll*)	9	DOWN (−2.8)	DOWN (−3.0)
Transmembrane 4 L6 family member 5-like (*tm4sf1*)	9	UP (1.6)	DOWN (−3.1)
Arginase-2-like (*arg2*)	15	UP (2.9)	_−_^d^
Rhesus glycoprotein (*rhcg*)	12	UP (3.7)	-
Elastase-1-like (*celal*)	9	UP (2.5)	-
Interleukin-12 subunit b-like (*il12b*)	9	-	UP (3.5)
Galactose-specific lectin nattectin-like (*ctl*)	15	DOWN (−3.9)	UP (3.2)
Egl-9 family hypoxia-inducible factor 3 (*egln3*)	12	DOWN (−2.5)	-
Hemoglobin subunit b-like (*hbb*)	9	DOWN (−3.6)	-
a-Tectorin-like (*tecta*)	78	-	DOWN (−4.6)
Mucin-2-like (*muc2*)	39	-	DOWN (−2.8)
Cartilage intermediate layer protein 2-like (*clip2*)	27	-	DOWN (−2.1)
GATA zinc finger domain-containing protein 14-like (*muc5ac*)	24	-	DOWN (−2.7)
Natterin-3-like	24	-	DOWN (−4.6)
Interferon-inducible GTPase 5-like (*irgm*)	21	-	DOWN (−3.4)
CUB and zona pellucida-like domain-containing protein 1-like (*zpldl*)	18	-	DOWN (−4.2)
Sperm acrosome membrane-associated protein 4-like (*spaca4*)	15	-	DOWN (−3.3)
Neoverrucotoxin subunit a-like (*neovtx*)	12	-	DOWN (−4.5)
Cytolysin RTX-S-2-like (*rtx-s II*)	9	-	DOWN (−3.7)
Cytoskeleton-associated protein 4 (*ckap4*)	9	-	DOWN (−3.3)
Seminal plasma glycoprotein 120 (*spp120/bsph1*)	9	-	DOWN (−4.1)
Retinol binding protein 2 (rbp2)	9	-	DOWN (−2.2)

^a^No. of Data are from three diapause replicates and three genome/transcriptome references (RPKM and fold change data are listed in the Supplementary document);

^b^AVERAGE(Log2(FoldChange)) ≥ 1.5;

^c^AVERAGE(Log2(FoldChange))≤**−**1.5;

^d^-1.5 < AVERAGE(Log2(FoldChange)) < 1.5

Our transcriptome profiling identified three genes (*dusp27, klhl38,* and *sqstm1*) that had higher expression in diapause than in both pre- and post-diapause in all three references (Table 5). Conversely, three other genes (*col9a1, dspp,* and *fmnl1)* were found to have lower expression in diapause than in both pre- and post-diapause (Table 5, Additional file 1: Table S2).

## Discussion

Environmental cues are known to contribute to the induction and termination of diapause in fishes, but any gene expression changes associated with the phenomenon are less well understood. Here we used NGS technologies to characterize patterns of gene expression during diapause in the world’s only vertebrate species that normally reproduces as a self-fertilizing hermaphrodite. Isogenic sets of Kmar embryos were screened for gene-expression profiles during diapause and compared against pre-diapause embryos and post-diapause larvae. To our knowledge, this is the first such study of naturally occurring diapause in any vertebrate species. Thus, massively parallel genetic expression analyses of vertebrate diapause via NGS-based RNA-Seq are unavailable for comparison with the current study.

Nevertheless, several diapause studies using an earlier technology (cDNA microarrays) can provide some useful perspectives. By using microarrays, 15,686 expression sequence tags (ESTs) were obtained from a time-course analysis of the Mummichog fish (*Fundulus heteroclitus*), including embryos with delayed hatching due to desiccation [28]. The individual *F. heteroclitus* genes identified were different than those reported in the current study. However, the ontologies of various genes involved in energy metabolism, cellular stress response, cytoskeleton, ion regulation, extracellular matrices, transcriptional control, and growth may share some degree of similarity with those reported here [28]. For invertebrates, several studies using RNA-Seq were published recently [17–19]. Again, particular genes found to be differentially regulated during diapause are not in obvious agreement with those identified in the current study. It may seem counterintuitive to observe that diapause occurs broadly across such animal taxa, yet few if any of the genes associated with the phenomenon were reportedly identical.

However, when we compare functional clusters of diapause genes (based on gene ontologies), we find some degree of similarity as exemplified by the following two summaries: (i) up-regulated transcripts during diapause in a leafcutting bee (*Megachile rotundata*) were involved in oxidative stress, neural activity, development, immune response, and ion homeostasis [18]; and (ii) differentially expressed diapause genes in a mosquito (*Aedes albopictus*) were involved in the cell cycle, oxidative phosphorylation, and DNA replication [17]. Similar statements apply to our current study of Kmar. Overall, we have identified 16 functional clusters of genes associated with diapause in Kmar. Within a particular cluster (e.g. genes related to extracellular matrices), we found that multiple genes were differentially regulated during diapause. Another example involves cardiac muscle related genes that on average were up-regulated in diapause over both pre- and post-diapause (and which therefore might highlight the importance of maintaining a functional heart during diapause). Similarly, genes related to the immune system were up-regulated in diapause over pre-diapause (perhaps suggesting the importance of maintaining embryo resistance against pathogens). Conversely, numerous gene clusters were down-regulated in diapause versus post-diapause (as might generally be expected). Examples include clusters involved in extracellular matrices, respiration or energy metabolism, cellular or enzymatic processes, hormones, cytoskeleton, toxin-like, ion binding, and development-related functions. Our results clearly provide evidence that after diapause is terminated, such gene clusters are activated beyond levels that were expressed during diapause.

## Conclusions

*Kryptolebias marmoratus* offers a strong model for understanding patterns of gene expression during diapause. Our study highlights the importance of using a combination of genome and transcriptome assemblies as references for NGS-based RNA-Seq analyses. For example, one gene cluster that proved to be up-regulated in Kmar diapause over both pre-diapause and post-diapause was related to cardiac function. Several other gene clusters important for various functions were on average similar in diapause and pre-diapause. However, numerous genes and gene clusters were down-regulated in diapause compared to post-diapause, suggesting that lower gene expression during delayed development is emblematic of the diapause phenomenon. Three individual diapause genes (*dusp27, klhl38,* and *sqstm1*) were highly up-regulated in diapause versus both pre-diapause and post-diapause. As for all identified diapause genes, in the future studies it will be critical to link various levels of RNA expression with the functional roles of the coded products.

## Methods

Live Kmar were provided by Dr. Ryan Earley, University of Alabama, Tuscaloosa. Progenitors of Kmar lineages used in this study were: Rad2 and Rad7 (collected at N27°20'48.4", W080°14'16.9"), OSR2.5 and OSR7.8 (N24°36'49.4", W081°33'06.7"), Nuke 13 (N27°21'00.2", W080°14'22.5"), PLT03 (N24°59'24.8", W080°33'04.7"), SOB8 (N24°36'05.2", W081°34'34.1") and FDS08 (N27°37'42.1", W082°42'13.6"). The fish were kept in 25 ppt saltwater (Instant Ocean® Sea Salt, Cat. No. SS15-10, United Pet Group Inc., Cincinnati, OH) on a 12 h light and 12 h dark photoperiod at 26 °C. Embryonic stages were determined under inverted light microscopy following the descriptions by [29]. Maintenance of Kmar (aquatic species) follows General Guidance for the Care and Maintenance of Ectothermic Species, Institutional Animal Care and Use Committee (IACUC) Protocol #2015-3161-0, University of California, Irvine.

Total RNA was extracted from pooled embryos at appropriate stages of development (Additional file 1: Table S3) following the protocol by Peterson and Freeman [30] using TRIzol® RNA isolation reagent (Thermo Fisher Scientific, http://www.thermofisher.com/us/en/home.html) and DNAseI treatment. Combining purified RNA minimizes the variance in gene expression across different lineages. We had three replicates of RNA-Seq samples representing diapause embryos (the prolonged stage 32), and one sample each for pre-diapause embryos (stage 31) and post-diapause (up to 48 h of hatched larvae) (Fig. 1). Quality control for the RNA was performed using NanoDrop 8000 (www.nanodrop.com) and Agilent 2100 Bioanalyzer (www.genomics.agilent.com) (Additional file 2: Figure S1). mRNA was enriched from a 500 ng subset of purified total RNA by using oligo(dT) magnetic beads and was sheared into ~200 nt fragments. The first strand of cDNA was produced by random hexamer priming followed by mRNA template removal using RNase H and the antisense strand was synthesized by DNA polymerase I. Sequencing primers and barcodes were ligated to the cDNA fragments, purified using agarose gel electrophoresis, and amplified by PCR. These libraries were then subjected to next generation sequencing (NGS) using Illumina HiSeq^TM^2000 at single-end, producing 50 bp-long reads. The library preparation and NGS were done at BGI America (bgiamericas.com). Image data generated by Illumina HiSeq^TM^2000 were transferred into sequence data in the form of fastq (.fq) files encoded by Illumina GA Pipeline v1.5. The relationship between sequencing error probabilities (*P*) and the quality value or Phred quality score (*Q*) is as follows: *Q* = -10Log_10_*P* [31]. Original reads were filtered to remove the following: adaptors; N (unresolved nucleotide positions); and sequences with low quality according to BGI’s in-house software (Additional file 1: Table S4). Filtered reads were then subjected to a further quality control check using a FastQC program (www.bioinformatics.babraham.ac.uk/projects/fastqc/) (Additional file 2: Figure S2–S3). Reads with Phred quality scores higher than 30 (>99.9 % base call accuracy) were used to create *de novo* transcriptome assemblies. Two RNA-Seq assemblers were utilized: Trinity (trinityrnaseq.github.io) and Trans-ABySS (www.bcgsc.ca/platform/bioinfo/software/trans-abyss) [24, 25]. K-mer size for Trans-ABySS assembly was k = 32 (such k-mer size offers a good tradeoff for assembling both rare and common transcripts) [24]. Three different modules were run by Trinity: with k-mer sizes up to 32 for “Inchworm”; and fixed k-mer sizes of 25 for “Chrysalis” and “Butterfly”; these parameters were reported to perform well for both highly and lowly expressed transcripts [25].

Purified genomic DNA of Kmar FDS08 from our previous study was randomly selected for whole genome sequencing (WGS) (Additional file 1: Table S5) [32] and sheared using Adaptive Focused Acoustics™ S2 (Covaris Inc., Woburn, MA). Libraries were constructed using NEXTflex™ DNA Sequencing Kit (Bioo Scientific Corp., Austin, TX). Library preparation and WGS were performed at UCI Genomics High-Throughput Facility (ghtf.biochem.uci.edu) on an Illumina HiSeq2500 sequencing system using PE100 cycles. Reads with *Q* < 30 were trimmed by using Trimmomatic software [33], whereas reads with *Q* >30 were used to create *de novo* genome assembly via the ABySS assembler [23]. N50 values as visualized by ABySS-Explorer [34, 35] serve as a quality control for the assemblies that were built successfully using various k-mers: k = 32, 40, 48, 56, 58, 60, and 64 [32].

Once a reference genome or transcriptome was built, RNA-Seq reads were processed into mRNA transcripts by using the approaches described in the following six steps: (i) index the reference genome or transcriptome for alignment (we used bowtie2, a Burrows-Wheeler indexer that keeps memory footprint small) [22]; (ii) download annotated genomes for the alignment from http://www.ensembl.org/info/data/ftp/index.html or http://hgdownload.soe.ucsc.edu/downloads.html (known reference genomes used in this study were from *Poecilia formosa* (amazon molly), *Xiphophorus maculatus* (platyfish), *Oryzias latipes* (medaka), *Danio rerio* (zebrafish), *Takifugu rubripes* (fugu), *Mus musculus* (mouse), and *Homo sapiens* (human)); (iii) align all RNA-Seq reads against an indexed reference genome, indexed reference transcriptome, or annotated genome by using the TopHat program (Table 1) [22]. (iv) index and sort the alignments by using the SamTools suite of programs (the sorted and indexed TopHat mappings can be visualized by an integrative genomics viewer (IGV) program [36]; (v) assemble the transcripts, quantitate gene expression, and compare fold changes of gene expression between biological samples from the TopHat mappings file using the software Cufflinks [22]; and (vi) visualize the results using the cummeRbund program run in an R environment using Bioconductor (Additional file 2: Figure S4–S5) [22]. These six steps were done for each reference genome or transcriptome.

The level of gene expression was measured with RPKM (reads per kilobase of transcript per million mapped reads) [37]. A fold change in gene expression between samples was measured by Log2 of the RPKM ratio. Each measurement of differential gene expression between samples had to satisfy the following three requirements performed by the cuffdiff program [22]: (i) enough alignments to perform a statistical test (‘OK’ status); (ii) the uncorrected p-value must be ≤0.0002 (in the test statistic used to compute significance of the observed change in RPKM), and (iii) the q value (the false discovery rate or FDR) after Benjamini Hochberg correction for multiple testing) must be ≤0.05 (Additional file 1: Table S1) [22]. Genes with differential expression were selected when an average of the three diapause replicates differed from pre-diapause or post-diapause samples by at least 1.5-fold (Tables 1 and 4, Additional file 1: Table S2). Sequences from these genes were aligned against nucleotide databases using BLAST (www.ncbi.nlm.nih.gov/blast/) [38]. Gene ontology was established by using AmiGO (amigo.geneontology.org) [26]. Molecular interaction analysis and visualization of human orthologs of Kmar genes were done by using networks available from The Biological General Repository for Interaction Datasets (BioGRID) (thebiogrid.org) or the Unified Human Interactome (UniHI) (www.unihi.org) [27, 39].

The sequences reported in this paper have been deposited in the National Center for Biotechnology Information (NCBI) Sequence Read Archive (SRA) database (www.ncbi.nlm.nih.gov) (accession nos. PRJNA282391, PRJNA288308, SRR1999414, SRR2001218, SRR2001221, SRR2001227, SRR2001231, SRR2079677, and SRR2080613).

Computational analyses were done using the University of California, Irvine (UCI) High Performance Computing (HPC) (hpc.oit.uci.edu).

## Additional files

Additional file 1:
**Table S1.** Abundances of transcripts identified from all three references. Listed transcripts are only those with p_values <0.0002 and FDR <0.05 (see text). **Table S2.** Differentially expressed genes in diapause versus pre- or post-diapause. **Table S3.** Total number of Kmar embryos used for RNA extraction. **Table S4.** Numbers of filtered RNA-Seq reads for downstream analyses. **Table S5.** NGS data used in this study and deposited at the NCBI SRA database. (PDF 915 kb) Additional file 2:
**Figure S1.** Quantity and quality of Kmar pooled RNA samples. A high quality of the samples was obtained prior to RNA-Seq. (A) table showing RNA quantity and quality as measured by NanoDrop and Agilent bioanalyzer. [Note: RIN is an RNA integrity number from Agilent2100’s measurement.] (B) Chromatogram for each pooled RNA sample that shows high integrity for both 28S and 18S rRNA. A 500 ng subset of pooled RNA was used for RNA-Seq processing. **Figure S2.** Quality of RNA-Seq data. Graphs indicate the high quality of RNA-Seq data from each sample. **Figure S3.** Quality of Kmar pair-ended (read1 and read2) WGS data. (A) Graphs indicate quality of WGS reads before and after the trimming. (B) Both raw and trimmed WGS reads were assembled to build a *de novo* genome at k = 32 and k = 64. The graph shows a relatively similar N50 value achieved by either raw or trimmed WGS reads. **Figure S4.** Pairwise comparison between samples for all genome/transcriptome references. The scatter diagram shows a negative binomial data distribution for transcript abundance in all samples. The number of transcripts for ABySS is 67,374; Trans-ABySS is 206,747; and Trinity is 97,979. These scatter diagram indicate that most of the transcripts were present in all developmental stages of Kmar. **Figure S5.** Phylogenetics of the RNA-Seq data. The tree shows Jensen-Shannon distance analyses for the five RNA-Seq samples. Transcriptomes from all three diapause replicates clustered together and displayed a slightly closer distance to pre- than post-diapause. (PDF 729 kb)
