# Supplementary material for: Transcriptomics of diapause in an isogenic self-fertilizing vertebrate
Source: BMC Genomics. 2015 Nov 23;16:989. doi: 10.1186/s12864-015-2210-0 (PMC4657215; doi:10.1186/s12864-015-2210-0)
Supplement: Additional file 1: — Table S1. Abundances of transcripts identified from all three references. Listed transcripts are only those with p_values <0.0002 and FDR <0.05 (see text). Table S2. Differentially expressed genes in diapause versus pre- or post-diapause. Table S3. Total number of Kmar embryos used for RNA extraction. Table S4. Numbers of filtered RNA-Seq reads for downstream analyses. Table S5. NGS data used in this study and deposited at the NCBI SRA database. (PDF 915 kb) [file 12864_2015_2210_MOESM1_ESM.pdf]

Supplementary Table S1. Abundances of transcripts identified from all three references. Listed transcripts are only those with p_values<0.0002 and FDR<0.05 (see text).

NGS	assembler	gene_id	locus	sample_1	sample_2	value_1	value_2	log2(fold_change)	p_value	FDR
WGS	ABySS	XLOC_000030	1006226:0-119	cdRNA02-Dia-R1	cdRNA05-postDia	4034.78	19643.6	2.2835	0.0001	0.0499641
WGS	ABySS	XLOC_000030	1006226:0-119	cdRNA01-preDia	cdRNA05-postDia	3272.98	19643.6	2.58539	5.00E-05	0.0289221
WGS	ABySS	XLOC_000633	1145608:18-115	cdRNA01-preDia	cdRNA05-postDia	678.869	15845.7	4.54481	5.00E-05	0.0289221
WGS	ABySS	XLOC_000633	1145608:18-115	cdRNA04-Dia-R3	cdRNA05-postDia	605.302	15845.7	4.71029	5.00E-05	0.0289221
WGS	ABySS	XLOC_000714	1162821:4896-6557	cdRNA02-Dia-R1	cdRNA05-postDia	4.60538	38.4108	3.06012	5.00E-05	0.0289221
WGS	ABySS	XLOC_001085	1245327:0-92	cdRNA03-Dia-R2	cdRNA05-postDia	23781	116635	2.29412	0.0001	0.0499641
WGS	ABySS	XLOC_001085	1245327:0-92	cdRNA02-Dia-R1	cdRNA05-postDia	13505.4	116635	3.1104	5.00E-05	0.0289221
WGS	ABySS	XLOC_001085	1245327:0-92	cdRNA01-preDia	cdRNA05-postDia	11889.5	116635	3.29424	5.00E-05	0.0289221
WGS	ABySS	XLOC_001263	1289175:1-118	cdRNA01-preDia	cdRNA04-Dia-R3	278.389	3495.65	3.65039	0.0001	0.0499641
WGS	ABySS	XLOC_001263	1289175:1-118	cdRNA01-preDia	cdRNA03-Dia-R2	278.389	4069.01	3.86951	0.0001	0.0499641
WGS	ABySS	XLOC_001268	129004:0-67	cdRNA01-preDia	cdRNA05-postDia	118140	16581.8	-2.83283	5.00E-05	0.0289221
WGS	ABySS	XLOC_001268	129004:0-67	cdRNA02-Dia-R1	cdRNA05-postDia	112133	16581.8	-2.75754	5.00E-05	0.0289221
WGS	ABySS	XLOC_001268	129004:0-67	cdRNA03-Dia-R2	cdRNA05-postDia	100335	16581.8	-2.59716	5.00E-05	0.0289221
WGS	ABySS	XLOC_001404	1321710:9777-12103	cdRNA03-Dia-R2	cdRNA05-postDia	2.18478	23.4252	3.4225	0.0001	0.0499641
WGS	ABySS	XLOC_001687	1386513:0-89	cdRNA02-Dia-R1	cdRNA05-postDia	11610.8	61794.5	2.41201	5.00E-05	0.0289221
WGS	ABySS	XLOC_001734	1398059:0-152	cdRNA01-preDia	cdRNA04-Dia-R3	229.801	2224.69	3.27514	5.00E-05	0.0289221
WGS	ABySS	XLOC_001734	1398059:0-152	cdRNA01-preDia	cdRNA03-Dia-R2	229.801	2326.89	3.33994	5.00E-05	0.0289221
WGS	ABySS	XLOC_001944	1441199:98-936	cdRNA03-Dia-R2	cdRNA05-postDia	39.7164	6.4759	-2.61658	0.0001	0.0499641
WGS	ABySS	XLOC_001944	1441199:98-936	cdRNA04-Dia-R3	cdRNA05-postDia	38.1668	6.4759	-2.55917	0.0001	0.0499641
WGS	ABySS	XLOC_001960	1445990:0-105	cdRNA01-preDia	cdRNA05-postDia	3433.83	21177.7	2.62466	5.00E-05	0.0289221
WGS	ABySS	XLOC_001960	1445990:0-105	cdRNA02-Dia-R1	cdRNA05-postDia	2809.06	21177.7	2.91439	5.00E-05	0.0289221
WGS	ABySS	XLOC_002168	1486847:0-78	cdRNA03-Dia-R2	cdRNA05-postDia	5799.07	40242.6	2.79483	5.00E-05	0.0289221
WGS	ABySS	XLOC_002335	1521010:0-114	cdRNA01-preDia	cdRNA05-postDia	4931.08	26502.6	2.42616	5.00E-05	0.0289221
WGS	ABySS	XLOC_002335	1521010:0-114	cdRNA04-Dia-R3	cdRNA05-postDia	4505.63	26502.6	2.55634	5.00E-05	0.0289221
WGS	ABySS	XLOC_002335	1521010:0-114	cdRNA03-Dia-R2	cdRNA05-postDia	3807.07	26502.6	2.79938	5.00E-05	0.0289221
WGS	ABySS	XLOC_002719	1601028:0-133	cdRNA02-Dia-R1	cdRNA05-postDia	691.824	4834.55	2.80491	5.00E-05	0.0289221
WGS	ABySS	XLOC_002780	161046:0-119	cdRNA01-preDia	cdRNA05-postDia	3053.45	18005.9	2.55995	5.00E-05	0.0289221
WGS	ABySS	XLOC_002936	164835:9452-26881	cdRNA01-preDia	cdRNA05-postDia	1.96798	13.5228	2.78061	5.00E-05	0.0289221
WGS	ABySS	XLOC_002986	1661360:0-119	cdRNA01-preDia	cdRNA05-postDia	18879.5	2239.63	-3.07549	0.0001	0.0499641
WGS	ABySS	XLOC_003476	1773601:9029-13253	cdRNA03-Dia-R2	cdRNA05-postDia	102.72	10.4072	-3.30306	5.00E-05	0.0289221
WGS	ABySS	XLOC_003476	1773601:9029-13253	cdRNA04-Dia-R3	cdRNA05-postDia	92.5226	10.4072	-3.15222	5.00E-05	0.0289221
WGS	ABySS	XLOC_003476	1773601:9029-13253	cdRNA01-preDia	cdRNA04-Dia-R3	14.0068	92.5226	2.72368	5.00E-05	0.0289221
WGS	ABySS	XLOC_003476	1773601:9029-13253	cdRNA01-preDia	cdRNA03-Dia-R2	14.0068	102.72	2.87451	5.00E-05	0.0289221
WGS	ABySS	XLOC_003547	1789444:0-119	cdRNA01-preDia	cdRNA05-postDia	4859.58	828.763	-2.5518	5.00E-05	0.0289221
WGS	ABySS	XLOC_003881	1861224:0-83	cdRNA04-Dia-R3	cdRNA05-postDia	16152.1	95492.2	2.56366	0.0001	0.0499641
WGS	ABySS	XLOC_003881	1861224:0-83	cdRNA02-Dia-R1	cdRNA05-postDia	15289.1	95492.2	2.64288	0.0001	0.0499641
WGS	ABySS	XLOC_003881	1861224:0-83	cdRNA03-Dia-R2	cdRNA05-postDia	14998.4	95492.2	2.67057	5.00E-05	0.0289221
WGS	ABySS	XLOC_003881	1861224:0-83	cdRNA01-preDia	cdRNA05-postDia	11593.7	95492.2	3.04204	5.00E-05	0.0289221
WGS	ABySS	XLOC_003971	1880164:0-112	cdRNA01-preDia	cdRNA02-Dia-R1	8866.39	536.324	-4.04717	5.00E-05	0.0289221
WGS	ABySS	XLOC_003971	1880164:0-112	cdRNA02-Dia-R1	cdRNA05-postDia	536.324	7277.88	3.76234	0.0001	0.0499641
WGS	ABySS	XLOC_004111	1912252:0-447	cdRNA03-Dia-R2	cdRNA05-postDia	92.0356	763.441	3.05225	5.00E-05	0.0289221
WGS	ABySS	XLOC_004111	1912252:0-447	cdRNA04-Dia-R3	cdRNA05-postDia	89.6358	763.441	3.09037	5.00E-05	0.0289221
WGS	ABySS	XLOC_004111	1912252:0-447	cdRNA01-preDia	cdRNA05-postDia	34.4377	763.441	4.47046	5.00E-05	0.0289221
WGS	ABySS	XLOC_004430	1980940:0-11583	cdRNA01-preDia	cdRNA04-Dia-R3	7.20976	39.101	2.43918	5.00E-05	0.0289221

WGS	ABySS	XLOC_004430	1980940:0-11583	cdRNA01-preDia	cdRNA03-Dia-R2	7.20976	41.4144	2.52211	5.00E-05	0.0289221
WGS	ABySS	XLOC_004696	2037673:0-74	cdRNA02-Dia-R1	cdRNA05-postDia	33792.3	206792	2.61341	5.00E-05	0.0289221
WGS	ABySS	XLOC_004963	2103326:0-119	cdRNA04-Dia-R3	cdRNA05-postDia	5729.83	39395.8	2.78148	5.00E-05	0.0289221
WGS	ABySS	XLOC_004963	2103326:0-119	cdRNA02-Dia-R1	cdRNA05-postDia	5216.76	39395.8	2.91682	5.00E-05	0.0289221
WGS	ABySS	XLOC_004963	2103326:0-119	cdRNA03-Dia-R2	cdRNA05-postDia	5002.78	39395.8	2.97724	5.00E-05	0.0289221
WGS	ABySS	XLOC_004963	2103326:0-119	cdRNA01-preDia	cdRNA05-postDia	3821.8	39395.8	3.36572	5.00E-05	0.0289221
WGS	ABySS	XLOC_005091	2137065:0-111	cdRNA01-preDia	cdRNA05-postDia	7486.84	52491.8	2.80966	5.00E-05	0.0289221
WGS	ABySS	XLOC_005989	2188352:1-3542	cdRNA04-Dia-R3	cdRNA05-postDia	264.872	23.4769	-3.49598	5.00E-05	0.0289221
WGS	ABySS	XLOC_005989	2188352:1-3542	cdRNA03-Dia-R2	cdRNA05-postDia	259.018	23.4769	-3.46374	5.00E-05	0.0289221
WGS	ABySS	XLOC_005989	2188352:1-3542	cdRNA01-preDia	cdRNA03-Dia-R2	43.6234	259.018	2.56988	5.00E-05	0.0289221
WGS	ABySS	XLOC_005989	2188352:1-3542	cdRNA01-preDia	cdRNA04-Dia-R3	43.6234	264.872	2.60212	5.00E-05	0.0289221
WGS	ABySS	XLOC_006061	2188496:375-9934	cdRNA01-preDia	cdRNA03-Dia-R2	12.3464	64.4925	2.38504	0.0001	0.0499641
WGS	ABySS	XLOC_006067	2188506:38309-50481	cdRNA01-preDia	cdRNA04-Dia-R3	4.02964	21.44	2.41158	0.0001	0.0499641
WGS	ABySS	XLOC_006067	2188506:38309-50481	cdRNA01-preDia	cdRNA03-Dia-R2	4.02964	22.9358	2.50888	5.00E-05	0.0289221
WGS	ABySS	XLOC_006227	2188952:223944-228047	cdRNA02-Dia-R1	cdRNA05-postDia	8.212	49.2746	2.58504	5.00E-05	0.0289221
WGS	ABySS	XLOC_006227	2188952:223944-228047	cdRNA04-Dia-R3	cdRNA05-postDia	7.84564	49.2746	2.65088	5.00E-05	0.0289221
WGS	ABySS	XLOC_006227	2188952:223944-228047	cdRNA03-Dia-R2	cdRNA05-postDia	7.45265	49.2746	2.72502	5.00E-05	0.0289221
WGS	ABySS	XLOC_006262	2189015:615-3027	cdRNA04-Dia-R3	cdRNA05-postDia	25.3542	141.511	2.48062	0.0001	0.0499641
WGS	ABySS	XLOC_006262	2189015:615-3027	cdRNA03-Dia-R2	cdRNA05-postDia	23.8108	141.511	2.57123	0.0001	0.0499641
WGS	ABySS	XLOC_006413	2189470:17695-23090	cdRNA01-preDia	cdRNA05-postDia	8.96956	43.8448	2.2893	0.0001	0.0499641
WGS	ABySS	XLOC_006413	2189470:17695-23090	cdRNA02-Dia-R1	cdRNA05-postDia	6.44815	43.8448	2.76545	5.00E-05	0.0289221
WGS	ABySS	XLOC_006501	2189680:32642-38719	cdRNA01-preDia	cdRNA04-Dia-R3	13.7902	65.9719	2.25821	0.0001	0.0499641
WGS	ABySS	XLOC_006967	2191278:2202-5007	cdRNA03-Dia-R2	cdRNA05-postDia	85.1695	9.6015	-3.14901	5.00E-05	0.0289221
WGS	ABySS	XLOC_006967	2191278:2202-5007	cdRNA04-Dia-R3	cdRNA05-postDia	79.0432	9.6015	-3.04131	5.00E-05	0.0289221
WGS	ABySS	XLOC_006967	2191278:2202-5007	cdRNA02-Dia-R1	cdRNA05-postDia	40.0558	9.6015	-2.06068	5.00E-05	0.0289221
WGS	ABySS	XLOC_007270	2192126:21143-29573	cdRNA04-Dia-R3	cdRNA05-postDia	0.489036	5.9379	3.60194	5.00E-05	0.0289221
WGS	ABySS	XLOC_007270	2192126:21143-29573	cdRNA03-Dia-R2	cdRNA05-postDia	0.462253	5.9379	3.6832	5.00E-05	0.0289221
WGS	ABySS	XLOC_007531	2192977:7579-9568	cdRNA01-preDia	cdRNA05-postDia	1.54199	19.4423	3.65634	0.0001	0.0499641
WGS	ABySS	XLOC_007531	2192977:7579-9568	cdRNA03-Dia-R2	cdRNA05-postDia	1.35251	19.4423	3.84549	5.00E-05	0.0289221
WGS	ABySS	XLOC_007569	2193085:48763-53452	cdRNA01-preDia	cdRNA05-postDia	6.58341	40.5686	2.62346	5.00E-05	0.0289221
WGS	ABySS	XLOC_007573	2193085:107118-109433	cdRNA01-preDia	cdRNA04-Dia-R3	24.1615	163.616	2.75953	5.00E-05	0.0289221
WGS	ABySS	XLOC_007573	2193085:107118-109433	cdRNA01-preDia	cdRNA03-Dia-R2	24.1615	170.345	2.81768	5.00E-05	0.0289221
WGS	ABySS	XLOC_007573	2193085:107118-109433	cdRNA01-preDia	cdRNA05-postDia	24.1615	184.901	2.93598	5.00E-05	0.0289221
WGS	ABySS	XLOC_007722	2193479:1-8176	cdRNA04-Dia-R3	cdRNA05-postDia	542.984	77.1333	-2.81548	5.00E-05	0.0289221
WGS	ABySS	XLOC_007722	2193479:1-8176	cdRNA03-Dia-R2	cdRNA05-postDia	518.928	77.1333	-2.75011	5.00E-05	0.0289221
WGS	ABySS	XLOC_008241	2194876:7280-9318	cdRNA04-Dia-R3	cdRNA05-postDia	1.67243	9.29519	2.47454	0.0001	0.0499641
WGS	ABySS	XLOC_008241	2194876:7280-9318	cdRNA02-Dia-R1	cdRNA05-postDia	1.58038	9.29519	2.55621	0.0001	0.0499641
WGS	ABySS	XLOC_008288	2195029:18836-30004	cdRNA02-Dia-R1	cdRNA05-postDia	4.87838	20.9619	2.1033	0.0001	0.0499641
WGS	ABySS	XLOC_008525	2195825:3717-7624	cdRNA01-preDia	cdRNA05-postDia	12.0191	57.3034	2.25329	0.0001	0.0499641
WGS	ABySS	XLOC_008647	2196137:1512-4555	cdRNA04-Dia-R3	cdRNA05-postDia	1.12646	8.2003	2.86388	5.00E-05	0.0289221
WGS	ABySS	XLOC_008833	2196718:9751-13684	cdRNA03-Dia-R2	cdRNA05-postDia	1.98331	16.3864	3.04651	5.00E-05	0.0289221
WGS	ABySS	XLOC_008833	2196718:9751-13684	cdRNA04-Dia-R3	cdRNA05-postDia	1.78273	16.3864	3.20033	5.00E-05	0.0289221
WGS	ABySS	XLOC_008861	2196767:27669-29035	cdRNA03-Dia-R2	cdRNA05-postDia	5.17899	34.8999	2.75248	5.00E-05	0.0289221
WGS	ABySS	XLOC_008910	2196832:62195-71282	cdRNA01-preDia	cdRNA03-Dia-R2	5.25019	0.870346	-2.59271	5.00E-05	0.0289221
WGS	ABySS	XLOC_008910	2196832:62195-71282	cdRNA01-preDia	cdRNA04-Dia-R3	5.25019	0.957299	-2.45533	5.00E-05	0.0289221
WGS	ABySS	XLOC_008910	2196832:62195-71282	cdRNA04-Dia-R3	cdRNA05-postDia	0.957299	8.73614	3.18995	5.00E-05	0.0289221
WGS	ABySS	XLOC_008910	2196832:62195-71282	cdRNA03-Dia-R2	cdRNA05-postDia	0.870346	8.73614	3.32734	5.00E-05	0.0289221

WGS	ABySS	XLOC_008942	2196946:10802-20446	cdRNA01-preDia	cdRNA05-postDia	3.11331	40.1745	3.68976	5.00E-05	0.0289221
WGS	ABySS	XLOC_008959	2196995:9289-11098	cdRNA02-Dia-R1	cdRNA05-postDia	12.3221	61.3109	2.3149	0.0001	0.0499641
WGS	ABySS	XLOC_008959	2196995:9289-11098	cdRNA04-Dia-R3	cdRNA05-postDia	11.6191	61.3109	2.39965	5.00E-05	0.0289221
WGS	ABySS	XLOC_008959	2196995:9289-11098	cdRNA03-Dia-R2	cdRNA05-postDia	10.0378	61.3109	2.6107	5.00E-05	0.0289221
WGS	ABySS	XLOC_009089	2197451:37901-39426	cdRNA02-Dia-R1	cdRNA05-postDia	10.4256	72.9595	2.80696	5.00E-05	0.0289221
WGS	ABySS	XLOC_009089	2197451:37901-39426	cdRNA01-preDia	cdRNA05-postDia	3.02276	72.9595	4.59316	5.00E-05	0.0289221
WGS	ABySS	XLOC_009089	2197451:37901-39426	cdRNA03-Dia-R2	cdRNA05-postDia	2.07583	72.9595	5.13533	5.00E-05	0.0289221
WGS	ABySS	XLOC_009089	2197451:37901-39426	cdRNA04-Dia-R3	cdRNA05-postDia	1.57443	72.9595	5.53419	5.00E-05	0.0289221
WGS	ABySS	XLOC_009138	2197620:1-1464	cdRNA02-Dia-R1	cdRNA05-postDia	10.7307	70.976	2.72559	5.00E-05	0.0289221
WGS	ABySS	XLOC_009138	2197620:1-1464	cdRNA03-Dia-R2	cdRNA05-postDia	3.91584	70.976	4.17994	5.00E-05	0.0289221
WGS	ABySS	XLOC_009138	2197620:1-1464	cdRNA01-preDia	cdRNA05-postDia	3.24649	70.976	4.45038	5.00E-05	0.0289221
WGS	ABySS	XLOC_009138	2197620:1-1464	cdRNA04-Dia-R3	cdRNA05-postDia	3.08602	70.976	4.52351	5.00E-05	0.0289221
WGS	ABySS	XLOC_009251	2198056:19123-33594	cdRNA01-preDia	cdRNA04-Dia-R3	19.1086	100.339	2.39259	5.00E-05	0.0289221
WGS	ABySS	XLOC_009251	2198056:19123-33594	cdRNA01-preDia	cdRNA03-Dia-R2	19.1086	101.91	2.41501	5.00E-05	0.0289221
WGS	ABySS	XLOC_009251	2198056:19123-33594	cdRNA01-preDia	cdRNA02-Dia-R1	19.1086	105.262	2.46169	5.00E-05	0.0289221
WGS	ABySS	XLOC_009849	2199795:63-5148	cdRNA01-preDia	cdRNA04-Dia-R3	1.31029	8.02739	2.61504	5.00E-05	0.0289221
WGS	ABySS	XLOC_009953	2200079:653-5516	cdRNA03-Dia-R2	cdRNA05-postDia	2.79588	27.6377	3.30526	5.00E-05	0.0289221
WGS	ABySS	XLOC_009953	2200079:653-5516	cdRNA02-Dia-R1	cdRNA05-postDia	2.6799	27.6377	3.36639	5.00E-05	0.0289221
WGS	ABySS	XLOC_009953	2200079:653-5516	cdRNA04-Dia-R3	cdRNA05-postDia	2.40989	27.6377	3.5196	5.00E-05	0.0289221
WGS	ABySS	XLOC_010009	2200201:2932-7019	cdRNA01-preDia	cdRNA02-Dia-R1	11396.2	9852.54	-0.209991	5.00E-05	0.0289221
WGS	ABySS	XLOC_010141	2200636:4344-5742	cdRNA03-Dia-R2	cdRNA05-postDia	22.6001	103.591	2.1965	5.00E-05	0.0289221
WGS	ABySS	XLOC_010141	2200636:4344-5742	cdRNA02-Dia-R1	cdRNA05-postDia	15.8677	103.591	2.70673	5.00E-05	0.0289221
WGS	ABySS	XLOC_010196	2200771:749-4091	cdRNA01-preDia	cdRNA05-postDia	6.77884	38.7146	2.51377	5.00E-05	0.0289221
WGS	ABySS	XLOC_010196	2200771:749-4091	cdRNA02-Dia-R1	cdRNA05-postDia	5.01759	38.7146	2.94781	5.00E-05	0.0289221
WGS	ABySS	XLOC_010254	2200992:2-3088	cdRNA02-Dia-R1	cdRNA05-postDia	26.5844	549.656	4.36987	5.00E-05	0.0289221
WGS	ABySS	XLOC_010254	2200992:2-3088	cdRNA01-preDia	cdRNA05-postDia	12.8282	549.656	5.42114	5.00E-05	0.0289221
WGS	ABySS	XLOC_010254	2200992:2-3088	cdRNA04-Dia-R3	cdRNA05-postDia	10.7162	549.656	5.68066	5.00E-05	0.0289221
WGS	ABySS	XLOC_010254	2200992:2-3088	cdRNA03-Dia-R2	cdRNA05-postDia	9.33328	549.656	5.88	5.00E-05	0.0289221
WGS	ABySS	XLOC_010532	2201692:2988-5254	cdRNA02-Dia-R1	cdRNA03-Dia-R2	2.99347	24.9748	3.06058	5.00E-05	0.0289221
WGS	ABySS	XLOC_010595	2201753:46-5769	cdRNA01-preDia	cdRNA05-postDia	14.0385	1.55303	-3.17623	5.00E-05	0.0289221
WGS	ABySS	XLOC_010809	2202357:666-3950	cdRNA02-Dia-R1	cdRNA05-postDia	17.4923	106.186	2.60179	0.0001	0.0499641
WGS	ABySS	XLOC_010928	2202626:56617-67323	cdRNA01-preDia	cdRNA04-Dia-R3	14.1191	96.4474	2.77209	5.00E-05	0.0289221
WGS	ABySS	XLOC_010928	2202626:56617-67323	cdRNA01-preDia	cdRNA03-Dia-R2	14.1191	103.45	2.87322	5.00E-05	0.0289221
WGS	ABySS	XLOC_011116	2203169:0-905	cdRNA02-Dia-R1	cdRNA05-postDia	6.66359	53.0336	2.99253	5.00E-05	0.0289221
WGS	ABySS	XLOC_011116	2203169:0-905	cdRNA04-Dia-R3	cdRNA05-postDia	5.7461	53.0336	3.20625	5.00E-05	0.0289221
WGS	ABySS	XLOC_011116	2203169:0-905	cdRNA03-Dia-R2	cdRNA05-postDia	5.55992	53.0336	3.25377	5.00E-05	0.0289221
WGS	ABySS	XLOC_011116	2203169:0-905	cdRNA01-preDia	cdRNA05-postDia	1.59211	53.0336	5.05789	5.00E-05	0.0289221
WGS	ABySS	XLOC_011174	2203361:2462-6761	cdRNA01-preDia	cdRNA05-postDia	5.89527	29.8522	2.34021	5.00E-05	0.0289221
WGS	ABySS	XLOC_011174	2203361:2462-6761	cdRNA03-Dia-R2	cdRNA05-postDia	5.19804	29.8522	2.5218	0.0001	0.0499641
WGS	ABySS	XLOC_011174	2203361:2462-6761	cdRNA04-Dia-R3	cdRNA05-postDia	4.58051	29.8522	2.70426	5.00E-05	0.0289221
WGS	ABySS	XLOC_011461	2204223:0-1779	cdRNA01-preDia	cdRNA05-postDia	1.39281	13.4572	3.2723	5.00E-05	0.0289221
WGS	ABySS	XLOC_011522	2204372:5722-20723	cdRNA01-preDia	cdRNA05-postDia	20.0677	122.58	2.61078	5.00E-05	0.0289221
WGS	ABySS	XLOC_012336	2206993:2-2529	cdRNA01-preDia	cdRNA05-postDia	6.48248	55.3423	3.09376	5.00E-05	0.0289221
WGS	ABySS	XLOC_012336	2206993:2-2529	cdRNA02-Dia-R1	cdRNA05-postDia	3.36102	55.3423	4.04141	5.00E-05	0.0289221
WGS	ABySS	XLOC_012336	2206993:2-2529	cdRNA03-Dia-R2	cdRNA05-postDia	1.99358	55.3423	4.79495	5.00E-05	0.0289221
WGS	ABySS	XLOC_012336	2206993:2-2529	cdRNA04-Dia-R3	cdRNA05-postDia	1.66667	55.3423	5.05334	5.00E-05	0.0289221
WGS	ABySS	XLOC_012829	2208195:0-10345	cdRNA03-Dia-R2	cdRNA05-postDia	33.0256	6.97602	-2.24311	0.0001	0.0499641

WGS	ABySS	XLOC_012829	2208195:0-10345	cdRNA04-Dia-R3	cdRNA05-postDia	32.3343	6.97602	-2.21259	5.00E-05	0.0289221
WGS	ABySS	XLOC_012829	2208195:0-10345	cdRNA01-preDia	cdRNA04-Dia-R3	7.73843	32.3343	2.06295	0.0001	0.0499641
WGS	ABySS	XLOC_012833	2208210:353-5927	cdRNA02-Dia-R1	cdRNA05-postDia	3.32738	21.4156	2.6862	5.00E-05	0.0289221
WGS	ABySS	XLOC_013070	2208979:17354-21440	cdRNA04-Dia-R3	cdRNA05-postDia	2.08597	13.4177	2.68535	5.00E-05	0.0289221
WGS	ABySS	XLOC_013070	2208979:17354-21440	cdRNA03-Dia-R2	cdRNA05-postDia	1.92582	13.4177	2.80059	5.00E-05	0.0289221
WGS	ABySS	XLOC_013393	2209850:1446-7261	cdRNA02-Dia-R1	cdRNA04-Dia-R3	11.3189	1.4325	-2.98212	0.0001	0.0499641
WGS	ABySS	XLOC_013393	2209850:1446-7261	cdRNA02-Dia-R1	cdRNA05-postDia	11.3189	58.7158	2.37502	0.0001	0.0499641
WGS	ABySS	XLOC_013393	2209850:1446-7261	cdRNA03-Dia-R2	cdRNA05-postDia	2.53326	58.7158	4.53468	5.00E-05	0.0289221
WGS	ABySS	XLOC_013393	2209850:1446-7261	cdRNA01-preDia	cdRNA05-postDia	2.11777	58.7158	4.79313	5.00E-05	0.0289221
WGS	ABySS	XLOC_013393	2209850:1446-7261	cdRNA04-Dia-R3	cdRNA05-postDia	1.4325	58.7158	5.35714	5.00E-05	0.0289221
WGS	ABySS	XLOC_013884	2211151:1186-2108	cdRNA01-preDia	cdRNA04-Dia-R3	15.517	91.8069	2.56475	0.0001	0.0499641
WGS	ABySS	XLOC_014000	2211574:694-6558	cdRNA01-preDia	cdRNA05-postDia	10.0063	0.749936	-3.738	5.00E-05	0.0289221
WGS	ABySS	XLOC_014000	2211574:694-6558	cdRNA03-Dia-R2	cdRNA05-postDia	8.63514	0.749936	-3.52538	5.00E-05	0.0289221
WGS	ABySS	XLOC_014000	2211574:694-6558	cdRNA04-Dia-R3	cdRNA05-postDia	7.85598	0.749936	-3.38895	5.00E-05	0.0289221
WGS	ABySS	XLOC_014264	2212646:9751-10744	cdRNA02-Dia-R1	cdRNA05-postDia	7.34191	62.8035	3.09662	5.00E-05	0.0289221
WGS	ABySS	XLOC_014264	2212646:9751-10744	cdRNA03-Dia-R2	cdRNA05-postDia	5.37773	62.8035	3.54578	5.00E-05	0.0289221
WGS	ABySS	XLOC_014264	2212646:9751-10744	cdRNA04-Dia-R3	cdRNA05-postDia	4.59921	62.8035	3.77139	5.00E-05	0.0289221
WGS	ABySS	XLOC_014344	2212867:7812-9477	cdRNA03-Dia-R2	cdRNA05-postDia	16.4084	2.4131	-2.76547	0.0001	0.0499641
WGS	ABySS	XLOC_014523	2213466:96-12277	cdRNA01-preDia	cdRNA04-Dia-R3	3.01857	36.3461	3.58986	5.00E-05	0.0289221
WGS	ABySS	XLOC_014523	2213466:96-12277	cdRNA01-preDia	cdRNA03-Dia-R2	3.01857	37.5283	3.63604	0.0001	0.0499641
WGS	ABySS	XLOC_014601	2213762:2545-5038	cdRNA01-preDia	cdRNA05-postDia	5.29637	104.096	4.29676	5.00E-05	0.0289221
WGS	ABySS	XLOC_014601	2213762:2545-5038	cdRNA02-Dia-R1	cdRNA05-postDia	2.7905	104.096	5.22124	5.00E-05	0.0289221
WGS	ABySS	XLOC_014849	2214666:2446-4039	cdRNA03-Dia-R2	cdRNA05-postDia	12.1209	81.6094	2.75124	5.00E-05	0.0289221
WGS	ABySS	XLOC_014849	2214666:2446-4039	cdRNA04-Dia-R3	cdRNA05-postDia	10.6152	81.6094	2.94261	5.00E-05	0.0289221
WGS	ABySS	XLOC_014991	2215192:3131-4323	cdRNA04-Dia-R3	cdRNA05-postDia	1.27171	17.1773	3.75566	5.00E-05	0.0289221
WGS	ABySS	XLOC_014991	2215192:3131-4323	cdRNA03-Dia-R2	cdRNA05-postDia	0.781161	17.1773	4.45874	0.0001	0.0499641
WGS	ABySS	XLOC_014993	2215192:5280-7230	cdRNA04-Dia-R3	cdRNA05-postDia	1.07943	12.5751	3.54222	5.00E-05	0.0289221
WGS	ABySS	XLOC_014993	2215192:5280-7230	cdRNA02-Dia-R1	cdRNA05-postDia	1.01536	12.5751	3.63051	5.00E-05	0.0289221
WGS	ABySS	XLOC_014993	2215192:5280-7230	cdRNA03-Dia-R2	cdRNA05-postDia	0.795661	12.5751	3.98227	5.00E-05	0.0289221
WGS	ABySS	XLOC_014993	2215192:5280-7230	cdRNA01-preDia	cdRNA05-postDia	0.529012	12.5751	4.57113	5.00E-05	0.0289221
WGS	ABySS	XLOC_015016	2215276:0-3524	cdRNA04-Dia-R3	cdRNA05-postDia	77.0342	11.4359	-2.75193	5.00E-05	0.0289221
WGS	ABySS	XLOC_015016	2215276:0-3524	cdRNA03-Dia-R2	cdRNA05-postDia	74.439	11.4359	-2.70248	5.00E-05	0.0289221
WGS	ABySS	XLOC_015261	2216119:11965-15212	cdRNA01-preDia	cdRNA04-Dia-R3	11.6265	78.405	2.75353	5.00E-05	0.0289221
WGS	ABySS	XLOC_015261	2216119:11965-15212	cdRNA01-preDia	cdRNA03-Dia-R2	11.6265	82.678	2.83009	5.00E-05	0.0289221
WGS	ABySS	XLOC_015364	2216359:3622-4921	cdRNA01-preDia	cdRNA04-Dia-R3	6.19957	35.0242	2.49811	5.00E-05	0.0289221
WGS	ABySS	XLOC_015414	2216548:5162-6082	cdRNA01-preDia	cdRNA05-postDia	88.2077	478.547	2.43969	5.00E-05	0.0289221
WGS	ABySS	XLOC_015635	2217310:0-2168	cdRNA03-Dia-R2	cdRNA05-postDia	4.52261	25.7636	2.51011	5.00E-05	0.0289221
WGS	ABySS	XLOC_015635	2217310:0-2168	cdRNA04-Dia-R3	cdRNA05-postDia	4.50663	25.7636	2.51521	5.00E-05	0.0289221
WGS	ABySS	XLOC_015690	2217601:1-8717	cdRNA01-preDia	cdRNA04-Dia-R3	13.9454	78.7057	2.49668	5.00E-05	0.0289221
WGS	ABySS	XLOC_015690	2217601:1-8717	cdRNA01-preDia	cdRNA03-Dia-R2	13.9454	79.4138	2.5096	5.00E-05	0.0289221
WGS	ABySS	XLOC_015705	2217654:3791-21577	cdRNA01-preDia	cdRNA05-postDia	59.6553	436.198	2.87026	5.00E-05	0.0289221
WGS	ABySS	XLOC_015705	2217654:3791-21577	cdRNA02-Dia-R1	cdRNA05-postDia	44.7914	436.198	3.28369	5.00E-05	0.0289221
WGS	ABySS	XLOC_015705	2217654:3791-21577	cdRNA04-Dia-R3	cdRNA05-postDia	43.0597	436.198	3.34057	5.00E-05	0.0289221
WGS	ABySS	XLOC_015705	2217654:3791-21577	cdRNA03-Dia-R2	cdRNA05-postDia	40.3762	436.198	3.43341	5.00E-05	0.0289221
WGS	ABySS	XLOC_015718	2217726:22383-40190	cdRNA04-Dia-R3	cdRNA05-postDia	2.75538	14.6526	2.41084	0.0001	0.0499641
WGS	ABySS	XLOC_015976	2218597:1196-6565	cdRNA03-Dia-R2	cdRNA05-postDia	2.29948	12.1066	2.39642	0.0001	0.0499641
WGS	ABySS	XLOC_016189	2219134:307-548	cdRNA04-Dia-R3	cdRNA05-postDia	138.956	797.489	2.52083	0.0001	0.0499641

WGS	ABySS	XLOC_016189	2219134:307-548	cdRNA03-Dia-R2	cdRNA05-postDia	135.529	797.489	2.55687	5.00E-05	0.0289221
WGS	ABySS	XLOC_016190	2219135:307-695	cdRNA04-Dia-R3	cdRNA05-postDia	79.4631	440.315	2.47018	5.00E-05	0.0289221
WGS	ABySS	XLOC_016322	2219409:10524-18130	cdRNA03-Dia-R2	cdRNA05-postDia	2.04906	12.7958	2.64264	5.00E-05	0.0289221
WGS	ABySS	XLOC_016322	2219409:10524-18130	cdRNA04-Dia-R3	cdRNA05-postDia	2.01401	12.7958	2.66753	5.00E-05	0.0289221
WGS	ABySS	XLOC_016419	2219636:7422-40368	cdRNA03-Dia-R2	cdRNA05-postDia	1.397	15.2786	3.45111	5.00E-05	0.0289221
WGS	ABySS	XLOC_016872	2221247:184-3312	cdRNA03-Dia-R2	cdRNA05-postDia	63.1924	11.9492	-2.40283	5.00E-05	0.0289221
WGS	ABySS	XLOC_016872	2221247:184-3312	cdRNA01-preDia	cdRNA04-Dia-R3	11.4217	56.5183	2.30695	0.0001	0.0499641
WGS	ABySS	XLOC_016872	2221247:184-3312	cdRNA01-preDia	cdRNA03-Dia-R2	11.4217	63.1924	2.46798	5.00E-05	0.0289221
WGS	ABySS	XLOC_017020	2221707:4175-11977	cdRNA01-preDia	cdRNA05-postDia	2.65588	19.6632	2.88823	5.00E-05	0.0289221
WGS	ABySS	XLOC_017143	2222163:1648-4181	cdRNA04-Dia-R3	cdRNA05-postDia	154.732	16.7312	-3.20915	5.00E-05	0.0289221
WGS	ABySS	XLOC_017143	2222163:1648-4181	cdRNA03-Dia-R2	cdRNA05-postDia	151.691	16.7312	-3.18052	5.00E-05	0.0289221
WGS	ABySS	XLOC_017197	2222307:431-1941	cdRNA03-Dia-R2	cdRNA05-postDia	1.06372	9.99377	3.23191	5.00E-05	0.0289221
WGS	ABySS	XLOC_017197	2222307:431-1941	cdRNA02-Dia-R1	cdRNA05-postDia	0.991144	9.99377	3.33386	0.0001	0.0499641
WGS	ABySS	XLOC_017401	2222782:10961-16605	cdRNA01-preDia	cdRNA03-Dia-R2	7.44673	40.5591	2.44535	5.00E-05	0.0289221
WGS	ABySS	XLOC_017417	2222837:1212-9438	cdRNA01-preDia	cdRNA05-postDia	6.86922	48.5947	2.82258	5.00E-05	0.0289221
WGS	ABySS	XLOC_017417	2222837:1212-9438	cdRNA01-preDia	cdRNA04-Dia-R3	6.86922	63.8586	3.21666	5.00E-05	0.0289221
WGS	ABySS	XLOC_017417	2222837:1212-9438	cdRNA01-preDia	cdRNA03-Dia-R2	6.86922	65.1922	3.24648	5.00E-05	0.0289221
WGS	ABySS	XLOC_017610	2223271:24-6540	cdRNA01-preDia	cdRNA04-Dia-R3	9.09325	67.7652	2.89768	5.00E-05	0.0289221
WGS	ABySS	XLOC_017610	2223271:24-6540	cdRNA01-preDia	cdRNA03-Dia-R2	9.09325	70.1223	2.94701	5.00E-05	0.0289221
WGS	ABySS	XLOC_017928	2224215:5829-15457	cdRNA04-Dia-R3	cdRNA05-postDia	0.674162	3.72135	2.46466	5.00E-05	0.0289221
WGS	ABySS	XLOC_017928	2224215:5829-15457	cdRNA03-Dia-R2	cdRNA05-postDia	0.588677	3.72135	2.66028	0.0001	0.0499641
WGS	ABySS	XLOC_018062	2224578:758-3609	cdRNA02-Dia-R1	cdRNA05-postDia	5.57746	41.7589	2.9044	5.00E-05	0.0289221
WGS	ABySS	XLOC_018062	2224578:758-3609	cdRNA01-preDia	cdRNA05-postDia	2.52254	41.7589	4.04913	5.00E-05	0.0289221
WGS	ABySS	XLOC_018062	2224578:758-3609	cdRNA04-Dia-R3	cdRNA05-postDia	1.06844	41.7589	5.28851	5.00E-05	0.0289221
WGS	ABySS	XLOC_018062	2224578:758-3609	cdRNA03-Dia-R2	cdRNA05-postDia	0.733832	41.7589	5.83049	5.00E-05	0.0289221
WGS	ABySS	XLOC_018470	2225734:11158-15028	cdRNA01-preDia	cdRNA03-Dia-R2	775.246	46.4262	-4.06164	5.00E-05	0.0289221
WGS	ABySS	XLOC_018470	2225734:11158-15028	cdRNA01-preDia	cdRNA04-Dia-R3	775.246	50.7304	-3.93373	5.00E-05	0.0289221
WGS	ABySS	XLOC_018471	2225734:9845-10985	cdRNA01-preDia	cdRNA03-Dia-R2	1805.19	106.125	-4.08832	5.00E-05	0.0289221
WGS	ABySS	XLOC_018471	2225734:9845-10985	cdRNA01-preDia	cdRNA04-Dia-R3	1805.19	107.111	-4.07497	5.00E-05	0.0289221
WGS	ABySS	XLOC_018768	2226515:551-3146	cdRNA02-Dia-R1	cdRNA05-postDia	50.4086	285.75	2.50301	0.0001	0.0499641
WGS	ABySS	XLOC_018768	2226515:551-3146	cdRNA04-Dia-R3	cdRNA05-postDia	47.0226	285.75	2.60333	0.0001	0.0499641
WGS	ABySS	XLOC_018768	2226515:551-3146	cdRNA03-Dia-R2	cdRNA05-postDia	46.0453	285.75	2.63363	0.0001	0.0499641
WGS	ABySS	XLOC_018818	2226711:337-3190	cdRNA01-preDia	cdRNA05-postDia	16.5384	294.437	4.15407	5.00E-05	0.0289221
WGS	ABySS	XLOC_018818	2226711:337-3190	cdRNA02-Dia-R1	cdRNA05-postDia	6.1854	294.437	5.57295	5.00E-05	0.0289221
WGS	ABySS	XLOC_018945	2227113:1686-5911	cdRNA01-preDia	cdRNA05-postDia	23.8546	116.076	2.28273	5.00E-05	0.0289221
WGS	ABySS	XLOC_018945	2227113:1686-5911	cdRNA02-Dia-R1	cdRNA05-postDia	21.3034	116.076	2.44591	5.00E-05	0.0289221
WGS	ABySS	XLOC_019094	2227533:3612-7770	cdRNA04-Dia-R3	cdRNA05-postDia	3.16021	23.5794	2.89944	5.00E-05	0.0289221
WGS	ABySS	XLOC_019094	2227533:3612-7770	cdRNA03-Dia-R2	cdRNA05-postDia	2.5512	23.5794	3.20828	5.00E-05	0.0289221
WGS	ABySS	XLOC_019137	2227620:0-2747	cdRNA02-Dia-R1	cdRNA05-postDia	6.44014	32.4941	2.33501	0.0001	0.0499641
WGS	ABySS	XLOC_019137	2227620:0-2747	cdRNA03-Dia-R2	cdRNA05-postDia	5.89185	32.4941	2.46338	0.0001	0.0499641
WGS	ABySS	XLOC_019137	2227620:0-2747	cdRNA01-preDia	cdRNA05-postDia	1.75894	32.4941	4.2074	5.00E-05	0.0289221
WGS	ABySS	XLOC_019138	2227620:2825-4893	cdRNA03-Dia-R2	cdRNA05-postDia	5.01462	29.6524	2.56393	5.00E-05	0.0289221
WGS	ABySS	XLOC_019138	2227620:2825-4893	cdRNA01-preDia	cdRNA05-postDia	1.75601	29.6524	4.07777	5.00E-05	0.0289221
WGS	ABySS	XLOC_019150	2227667:62407-72870	cdRNA01-preDia	cdRNA05-postDia	1.96368	13.7461	2.80739	5.00E-05	0.0289221
WGS	ABySS	XLOC_019303	2228007:74-1211	cdRNA03-Dia-R2	cdRNA05-postDia	7.58597	65.0461	3.10006	5.00E-05	0.0289221
WGS	ABySS	XLOC_019303	2228007:74-1211	cdRNA04-Dia-R3	cdRNA05-postDia	6.71653	65.0461	3.27567	5.00E-05	0.0289221
WGS	ABySS	XLOC_019303	2228007:74-1211	cdRNA02-Dia-R1	cdRNA05-postDia	6.04432	65.0461	3.42781	5.00E-05	0.0289221

WGS	ABySS	XLOC_019345	2228164:78-1648	cdRNA01-preDia	cdRNA05-postDia	34.6834	3.07361	-3.49624	5.00E-05	0.0289221
WGS	ABySS	XLOC_019345	2228164:78-1648	cdRNA01-preDia	cdRNA02-Dia-R1	34.6834	5.59704	-2.63151	5.00E-05	0.0289221
WGS	ABySS	XLOC_019439	2228444:17208-19191	cdRNA03-Dia-R2	cdRNA05-postDia	78.5677	8.89376	-3.14307	5.00E-05	0.0289221
WGS	ABySS	XLOC_019439	2228444:17208-19191	cdRNA04-Dia-R3	cdRNA05-postDia	76.6531	8.89376	-3.10748	5.00E-05	0.0289221
WGS	ABySS	XLOC_019607	2228890:0-2126	cdRNA01-preDia	cdRNA05-postDia	2.63837	24.2997	3.20322	5.00E-05	0.0289221
WGS	ABySS	XLOC_019695	2229153:7977-14222	cdRNA04-Dia-R3	cdRNA05-postDia	78.3115	15.2043	-2.36475	5.00E-05	0.0289221
WGS	ABySS	XLOC_019695	2229153:7977-14222	cdRNA03-Dia-R2	cdRNA05-postDia	75.7021	15.2043	-2.31585	0.0001	0.0499641
WGS	ABySS	XLOC_019695	2229153:7977-14222	cdRNA01-preDia	cdRNA03-Dia-R2	7.17055	75.7021	3.40018	5.00E-05	0.0289221
WGS	ABySS	XLOC_019695	2229153:7977-14222	cdRNA01-preDia	cdRNA04-Dia-R3	7.17055	78.3115	3.44907	5.00E-05	0.0289221
WGS	ABySS	XLOC_019696	2229153:19709-24924	cdRNA04-Dia-R3	cdRNA05-postDia	47.1531	5.02631	-3.22978	5.00E-05	0.0289221
WGS	ABySS	XLOC_019696	2229153:19709-24924	cdRNA03-Dia-R2	cdRNA05-postDia	46.6824	5.02631	-3.21531	5.00E-05	0.0289221
WGS	ABySS	XLOC_019696	2229153:19709-24924	cdRNA02-Dia-R1	cdRNA03-Dia-R2	6.91955	46.6824	2.75413	5.00E-05	0.0289221
WGS	ABySS	XLOC_019696	2229153:19709-24924	cdRNA02-Dia-R1	cdRNA04-Dia-R3	6.91955	47.1531	2.7686	5.00E-05	0.0289221
WGS	ABySS	XLOC_019696	2229153:19709-24924	cdRNA01-preDia	cdRNA03-Dia-R2	1.01853	46.6824	5.51831	5.00E-05	0.0289221
WGS	ABySS	XLOC_019696	2229153:19709-24924	cdRNA01-preDia	cdRNA04-Dia-R3	1.01853	47.1531	5.53279	5.00E-05	0.0289221
WGS	ABySS	XLOC_019870	2229548:1756-14554	cdRNA04-Dia-R3	cdRNA05-postDia	1.00144	5.60063	2.48351	5.00E-05	0.0289221
WGS	ABySS	XLOC_019870	2229548:1756-14554	cdRNA03-Dia-R2	cdRNA05-postDia	0.849448	5.60063	2.72099	5.00E-05	0.0289221
WGS	ABySS	XLOC_019996	2229912:8642-14234	cdRNA03-Dia-R2	cdRNA05-postDia	4.06158	23.7729	2.5492	0.0001	0.0499641
WGS	ABySS	XLOC_019996	2229912:8642-14234	cdRNA01-preDia	cdRNA05-postDia	3.64767	23.7729	2.70427	5.00E-05	0.0289221
WGS	ABySS	XLOC_020585	2231772:25467-26352	cdRNA01-preDia	cdRNA04-Dia-R3	178.09	12.5101	-3.83145	5.00E-05	0.0289221
WGS	ABySS	XLOC_020585	2231772:25467-26352	cdRNA01-preDia	cdRNA03-Dia-R2	178.09	13.807	-3.68914	5.00E-05	0.0289221
WGS	ABySS	XLOC_020585	2231772:25467-26352	cdRNA01-preDia	cdRNA02-Dia-R1	178.09	20.8314	-3.09578	5.00E-05	0.0289221
WGS	ABySS	XLOC_020585	2231772:25467-26352	cdRNA01-preDia	cdRNA05-postDia	178.09	26.866	-2.72876	5.00E-05	0.0289221
WGS	ABySS	XLOC_020684	2232133:25614-31175	cdRNA03-Dia-R2	cdRNA05-postDia	27.6611	128.164	2.21206	0.0001	0.0499641
WGS	ABySS	XLOC_020810	2232570:34753-37801	cdRNA02-Dia-R1	cdRNA05-postDia	22.6462	125.75	2.47322	0.0001	0.0499641
WGS	ABySS	XLOC_020953	2232959:630-3839	cdRNA03-Dia-R2	cdRNA05-postDia	3.81759	23.6292	2.62983	5.00E-05	0.0289221
WGS	ABySS	XLOC_020953	2232959:630-3839	cdRNA04-Dia-R3	cdRNA05-postDia	3.51787	23.6292	2.74779	5.00E-05	0.0289221
WGS	ABySS	XLOC_021015	2233122:12030-14540	cdRNA01-preDia	cdRNA05-postDia	4.95975	28.2702	2.51094	5.00E-05	0.0289221
WGS	ABySS	XLOC_021015	2233122:12030-14540	cdRNA02-Dia-R1	cdRNA05-postDia	4.05227	28.2702	2.80248	5.00E-05	0.0289221
WGS	ABySS	XLOC_021138	2233429:4-574	cdRNA02-Dia-R1	cdRNA05-postDia	7.76518	63.4185	3.02981	5.00E-05	0.0289221
WGS	ABySS	XLOC_021138	2233429:4-574	cdRNA04-Dia-R3	cdRNA05-postDia	6.05797	63.4185	3.388	5.00E-05	0.0289221
WGS	ABySS	XLOC_021138	2233429:4-574	cdRNA03-Dia-R2	cdRNA05-postDia	5.37709	63.4185	3.56001	5.00E-05	0.0289221
WGS	ABySS	XLOC_021139	2233439:7013-44919	cdRNA01-preDia	cdRNA03-Dia-R2	13.712	2.28925	-2.58249	0.0001	0.0499641
WGS	ABySS	XLOC_021139	2233439:7013-44919	cdRNA01-preDia	cdRNA04-Dia-R3	13.712	2.47757	-2.46844	5.00E-05	0.0289221
WGS	ABySS	XLOC_021386	2234068:0-647	cdRNA03-Dia-R2	cdRNA05-postDia	10.2787	81.1705	2.98129	5.00E-05	0.0289221
WGS	ABySS	XLOC_021386	2234068:0-647	cdRNA02-Dia-R1	cdRNA05-postDia	9.01983	81.1705	3.16978	5.00E-05	0.0289221
WGS	ABySS	XLOC_021386	2234068:0-647	cdRNA04-Dia-R3	cdRNA05-postDia	8.6754	81.1705	3.22595	5.00E-05	0.0289221
WGS	ABySS	XLOC_021386	2234068:0-647	cdRNA01-preDia	cdRNA05-postDia	6.4286	81.1705	3.65838	5.00E-05	0.0289221
WGS	ABySS	XLOC_021426	2234249:578-7279	cdRNA04-Dia-R3	cdRNA05-postDia	26.3035	5.53075	-2.24971	5.00E-05	0.0289221
WGS	ABySS	XLOC_021426	2234249:578-7279	cdRNA03-Dia-R2	cdRNA05-postDia	26.0478	5.53075	-2.23561	5.00E-05	0.0289221
WGS	ABySS	XLOC_021426	2234249:578-7279	cdRNA01-preDia	cdRNA03-Dia-R2	5.97128	26.0478	2.12505	5.00E-05	0.0289221
WGS	ABySS	XLOC_021426	2234249:578-7279	cdRNA01-preDia	cdRNA04-Dia-R3	5.97128	26.3035	2.13915	0.0001	0.0499641
WGS	ABySS	XLOC_021960	2236156:71442-76436	cdRNA03-Dia-R2	cdRNA05-postDia	16.3265	2.1235	-2.9427	5.00E-05	0.0289221
WGS	ABySS	XLOC_021960	2236156:71442-76436	cdRNA04-Dia-R3	cdRNA05-postDia	16.2648	2.1235	-2.93724	5.00E-05	0.0289221
WGS	ABySS	XLOC_021961	2236156:76521-83822	cdRNA03-Dia-R2	cdRNA05-postDia	12.1559	1.74336	-2.80172	5.00E-05	0.0289221
WGS	ABySS	XLOC_021961	2236156:76521-83822	cdRNA04-Dia-R3	cdRNA05-postDia	12.0073	1.74336	-2.78397	5.00E-05	0.0289221
WGS	ABySS	XLOC_022053	2236455:6-1527	cdRNA03-Dia-R2	cdRNA05-postDia	24.818	3.20712	-2.95204	5.00E-05	0.0289221

WGS	ABySS	XLOC_022053	2236455:6-1527	cdRNA04-Dia-R3	cdRNA05-postDia	24.2716	3.20712	-2.91992	5.00E-05	0.0289221
WGS	ABySS	XLOC_022322	2237274:1860-4526	cdRNA03-Dia-R2	cdRNA05-postDia	1.37638	12.0753	3.1331	0.0001	0.0499641
WGS	ABySS	XLOC_022322	2237274:1860-4526	cdRNA04-Dia-R3	cdRNA05-postDia	1.10149	12.0753	3.45452	0.0001	0.0499641
WGS	ABySS	XLOC_022322	2237274:1860-4526	cdRNA02-Dia-R1	cdRNA05-postDia	1.09534	12.0753	3.4626	5.00E-05	0.0289221
WGS	ABySS	XLOC_022614	2238156:7676-12787	cdRNA04-Dia-R3	cdRNA05-postDia	46.2799	7.36255	-2.65211	5.00E-05	0.0289221
WGS	ABySS	XLOC_022614	2238156:7676-12787	cdRNA03-Dia-R2	cdRNA05-postDia	44.6719	7.36255	-2.60109	5.00E-05	0.0289221
WGS	ABySS	XLOC_022756	2238529:987-20061	cdRNA03-Dia-R2	cdRNA05-postDia	69.9431	4.60838	-3.92385	5.00E-05	0.0289221
WGS	ABySS	XLOC_022756	2238529:987-20061	cdRNA04-Dia-R3	cdRNA05-postDia	67.7842	4.60838	-3.87862	5.00E-05	0.0289221
WGS	ABySS	XLOC_022756	2238529:987-20061	cdRNA02-Dia-R1	cdRNA05-postDia	25.3167	4.60838	-2.45775	5.00E-05	0.0289221
WGS	ABySS	XLOC_022756	2238529:987-20061	cdRNA01-preDia	cdRNA04-Dia-R3	10.0026	67.7842	2.76057	5.00E-05	0.0289221
WGS	ABySS	XLOC_022756	2238529:987-20061	cdRNA01-preDia	cdRNA03-Dia-R2	10.0026	69.9431	2.8058	5.00E-05	0.0289221
WGS	ABySS	XLOC_022757	2238529:21510-36089	cdRNA04-Dia-R3	cdRNA05-postDia	115.719	13.0337	-3.15031	5.00E-05	0.0289221
WGS	ABySS	XLOC_022757	2238529:21510-36089	cdRNA03-Dia-R2	cdRNA05-postDia	113.321	13.0337	-3.12009	5.00E-05	0.0289221
WGS	ABySS	XLOC_022757	2238529:21510-36089	cdRNA01-preDia	cdRNA03-Dia-R2	13.5261	113.321	3.0666	5.00E-05	0.0289221
WGS	ABySS	XLOC_022757	2238529:21510-36089	cdRNA01-preDia	cdRNA04-Dia-R3	13.5261	115.719	3.09682	5.00E-05	0.0289221
WGS	ABySS	XLOC_023002	2239314:972-8371	cdRNA03-Dia-R2	cdRNA05-postDia	45.208	9.01375	-2.32638	5.00E-05	0.0289221
WGS	ABySS	XLOC_023002	2239314:972-8371	cdRNA04-Dia-R3	cdRNA05-postDia	40.6485	9.01375	-2.173	5.00E-05	0.0289221
WGS	ABySS	XLOC_023109	2239659:919-3636	cdRNA03-Dia-R2	cdRNA05-postDia	88.8266	6.59009	-3.75262	5.00E-05	0.0289221
WGS	ABySS	XLOC_023109	2239659:919-3636	cdRNA04-Dia-R3	cdRNA05-postDia	74.335	6.59009	-3.49567	5.00E-05	0.0289221
WGS	ABySS	XLOC_023390	2240588:9258-56876	cdRNA03-Dia-R2	cdRNA05-postDia	4.74872	24.1219	2.34474	5.00E-05	0.0289221
WGS	ABySS	XLOC_023390	2240588:9258-56876	cdRNA04-Dia-R3	cdRNA05-postDia	4.21122	24.1219	2.51804	5.00E-05	0.0289221
WGS	ABySS	XLOC_023572	2241176:257-4659	cdRNA01-preDia	cdRNA05-postDia	0.984335	6.50455	2.72423	5.00E-05	0.0289221
WGS	ABySS	XLOC_023739	2241667:38-13000	cdRNA01-preDia	cdRNA05-postDia	2.16533	9.57522	2.14472	5.00E-05	0.0289221
WGS	ABySS	XLOC_023775	2241823:641-10570	cdRNA03-Dia-R2	cdRNA05-postDia	65.6117	10.5092	-2.6423	5.00E-05	0.0289221
WGS	ABySS	XLOC_023775	2241823:641-10570	cdRNA04-Dia-R3	cdRNA05-postDia	59.2796	10.5092	-2.49588	0.0001	0.0499641
WGS	ABySS	XLOC_023995	2242419:44331-47867	cdRNA03-Dia-R2	cdRNA05-postDia	1.7794	15.7424	3.14519	5.00E-05	0.0289221
WGS	ABySS	XLOC_024149	2242834:5023-7052	cdRNA04-Dia-R3	cdRNA05-postDia	20.6623	2.23631	-3.20781	5.00E-05	0.0289221
WGS	ABySS	XLOC_024149	2242834:5023-7052	cdRNA03-Dia-R2	cdRNA05-postDia	20.3099	2.23631	-3.18299	5.00E-05	0.0289221
WGS	ABySS	XLOC_024149	2242834:5023-7052	cdRNA01-preDia	cdRNA03-Dia-R2	3.83541	20.3099	2.40473	5.00E-05	0.0289221
WGS	ABySS	XLOC_024149	2242834:5023-7052	cdRNA01-preDia	cdRNA04-Dia-R3	3.83541	20.6623	2.42955	5.00E-05	0.0289221
WGS	ABySS	XLOC_024333	2243366:612-5476	cdRNA03-Dia-R2	cdRNA05-postDia	158.112	28.2112	-2.48661	0.0001	0.0499641
WGS	ABySS	XLOC_024505	2243867:1074-10112	cdRNA03-Dia-R2	cdRNA05-postDia	15.0057	2.67725	-2.48669	5.00E-05	0.0289221
WGS	ABySS	XLOC_024589	2244198:18333-23702	cdRNA03-Dia-R2	cdRNA05-postDia	18.4384	2.51407	-2.87462	5.00E-05	0.0289221
WGS	ABySS	XLOC_024589	2244198:18333-23702	cdRNA04-Dia-R3	cdRNA05-postDia	17.5278	2.51407	-2.80155	5.00E-05	0.0289221
WGS	ABySS	XLOC_024591	2244215:3974-9781	cdRNA01-preDia	cdRNA03-Dia-R2	1.14402	12.374	3.43512	5.00E-05	0.0289221
WGS	ABySS	XLOC_024591	2244215:3974-9781	cdRNA01-preDia	cdRNA04-Dia-R3	1.14402	13.0009	3.50642	5.00E-05	0.0289221
WGS	ABySS	XLOC_025037	2245857:48188-57602	cdRNA01-preDia	cdRNA05-postDia	2.96636	16.5746	2.48221	0.0001	0.0499641
WGS	ABySS	XLOC_025053	2245930:831-4681	cdRNA03-Dia-R2	cdRNA05-postDia	10.8958	1.95688	-2.47715	5.00E-05	0.0289221
WGS	ABySS	XLOC_025053	2245930:831-4681	cdRNA04-Dia-R3	cdRNA05-postDia	10.1907	1.95688	-2.38063	5.00E-05	0.0289221
WGS	ABySS	XLOC_025316	2246858:2440-4390	cdRNA01-preDia	cdRNA05-postDia	25.4459	175.068	2.78241	5.00E-05	0.0289221
WGS	ABySS	XLOC_025316	2246858:2440-4390	cdRNA04-Dia-R3	cdRNA05-postDia	13.7836	175.068	3.66689	5.00E-05	0.0289221
WGS	ABySS	XLOC_025316	2246858:2440-4390	cdRNA03-Dia-R2	cdRNA05-postDia	12.4702	175.068	3.81136	5.00E-05	0.0289221
WGS	ABySS	XLOC_025316	2246858:2440-4390	cdRNA02-Dia-R1	cdRNA05-postDia	11.373	175.068	3.94423	5.00E-05	0.0289221
WGS	ABySS	XLOC_025317	2246858:13-2364	cdRNA03-Dia-R2	cdRNA05-postDia	1.0245	10.8999	3.41132	5.00E-05	0.0289221
WGS	ABySS	XLOC_025317	2246858:13-2364	cdRNA04-Dia-R3	cdRNA05-postDia	0.929462	10.8999	3.55178	5.00E-05	0.0289221
WGS	ABySS	XLOC_025317	2246858:13-2364	cdRNA02-Dia-R1	cdRNA05-postDia	0.865039	10.8999	3.65541	0.0001	0.0499641
WGS	ABySS	XLOC_025317	2246858:13-2364	cdRNA01-preDia	cdRNA05-postDia	0.806639	10.8999	3.75625	5.00E-05	0.0289221

WGS	ABySS	XLOC_025367	2246993:0-2379	cdRNA01-preDia	cdRNA05-postDia	179.619	42.2191	-2.08897	0.0001	0.0499641
WGS	ABySS	XLOC_025374	2246999:2-4259	cdRNA04-Dia-R3	cdRNA05-postDia	197.982	26.8145	-2.88429	5.00E-05	0.0289221
WGS	ABySS	XLOC_025374	2246999:2-4259	cdRNA03-Dia-R2	cdRNA05-postDia	190.859	26.8145	-2.83142	5.00E-05	0.0289221
WGS	ABySS	XLOC_025492	2247450:51-5020	cdRNA02-Dia-R1	cdRNA05-postDia	16.5566	87.0253	2.39403	0.0001	0.0499641
WGS	ABySS	XLOC_025598	2247711:24546-34615	cdRNA02-Dia-R1	cdRNA05-postDia	6.73459	29.6843	2.14004	0.0001	0.0499641
WGS	ABySS	XLOC_025598	2247711:24546-34615	cdRNA03-Dia-R2	cdRNA05-postDia	6.56785	29.6843	2.1762	5.00E-05	0.0289221
WGS	ABySS	XLOC_025598	2247711:24546-34615	cdRNA04-Dia-R3	cdRNA05-postDia	6.41013	29.6843	2.21127	5.00E-05	0.0289221
WGS	ABySS	XLOC_025786	2248098:157-1898	cdRNA01-preDia	cdRNA05-postDia	3.23008	20.8952	2.69353	5.00E-05	0.0289221
WGS	ABySS	XLOC_025786	2248098:157-1898	cdRNA02-Dia-R1	cdRNA05-postDia	1.44636	20.8952	3.85268	5.00E-05	0.0289221
WGS	ABySS	XLOC_025786	2248098:157-1898	cdRNA04-Dia-R3	cdRNA05-postDia	1.00982	20.8952	4.371	5.00E-05	0.0289221
WGS	ABySS	XLOC_025786	2248098:157-1898	cdRNA03-Dia-R2	cdRNA05-postDia	1.00428	20.8952	4.37894	0.0001	0.0499641
WGS	ABySS	XLOC_026487	2250591:6674-9087	cdRNA04-Dia-R3	cdRNA05-postDia	37.8246	7.59181	-2.31681	0.0001	0.0499641
WGS	ABySS	XLOC_026525	2250719:0-1111	cdRNA02-Dia-R1	cdRNA05-postDia	4.59171	26.3049	2.51823	5.00E-05	0.0289221
WGS	ABySS	XLOC_026525	2250719:0-1111	cdRNA03-Dia-R2	cdRNA05-postDia	4.38225	26.3049	2.58559	0.0001	0.0499641
WGS	ABySS	XLOC_026525	2250719:0-1111	cdRNA01-preDia	cdRNA05-postDia	1.10322	26.3049	4.57554	5.00E-05	0.0289221
WGS	ABySS	XLOC_026735	2251242:26181-30866	cdRNA03-Dia-R2	cdRNA05-postDia	1.2493	8.14104	2.7041	5.00E-05	0.0289221
WGS	ABySS	XLOC_026735	2251242:26181-30866	cdRNA02-Dia-R1	cdRNA05-postDia	1.09741	8.14104	2.8911	5.00E-05	0.0289221
WGS	ABySS	XLOC_026735	2251242:26181-30866	cdRNA01-preDia	cdRNA05-postDia	0.980819	8.14104	3.05315	5.00E-05	0.0289221
WGS	ABySS	XLOC_026778	2251375:2610-7025	cdRNA04-Dia-R3	cdRNA05-postDia	1.43872	11.5748	3.00813	5.00E-05	0.0289221
WGS	ABySS	XLOC_026778	2251375:2610-7025	cdRNA03-Dia-R2	cdRNA05-postDia	1.13019	11.5748	3.35635	5.00E-05	0.0289221
WGS	ABySS	XLOC_026778	2251375:2610-7025	cdRNA02-Dia-R1	cdRNA05-postDia	1.04137	11.5748	3.47443	5.00E-05	0.0289221
WGS	ABySS	XLOC_026964	2251878:162-10865	cdRNA02-Dia-R1	cdRNA05-postDia	2.66671	15.8762	2.57373	5.00E-05	0.0289221
WGS	ABySS	XLOC_026964	2251878:162-10865	cdRNA03-Dia-R2	cdRNA05-postDia	2.03189	15.8762	2.96597	5.00E-05	0.0289221
WGS	ABySS	XLOC_026964	2251878:162-10865	cdRNA04-Dia-R3	cdRNA05-postDia	2.00676	15.8762	2.98393	5.00E-05	0.0289221
WGS	ABySS	XLOC_026975	2251893:10222-15441	cdRNA01-preDia	cdRNA03-Dia-R2	41.9225	7.42526	-2.49721	5.00E-05	0.0289221
WGS	ABySS	XLOC_026975	2251893:10222-15441	cdRNA01-preDia	cdRNA04-Dia-R3	41.9225	7.76968	-2.4318	5.00E-05	0.0289221
WGS	ABySS	XLOC_027039	2252018:1588-2558	cdRNA01-preDia	cdRNA03-Dia-R2	21.7045	164.583	2.92276	5.00E-05	0.0289221
WGS	ABySS	XLOC_027039	2252018:1588-2558	cdRNA01-preDia	cdRNA04-Dia-R3	21.7045	166.97	2.94352	5.00E-05	0.0289221
WGS	ABySS	XLOC_027057	2252048:21678-29767	cdRNA03-Dia-R2	cdRNA05-postDia	14.3969	67.5501	2.2302	5.00E-05	0.0289221
WGS	ABySS	XLOC_027057	2252048:21678-29767	cdRNA02-Dia-R1	cdRNA05-postDia	14.0519	67.5501	2.26519	5.00E-05	0.0289221
WGS	ABySS	XLOC_027343	2253302:1021-5466	cdRNA04-Dia-R3	cdRNA05-postDia	2.68265	14.5646	2.44074	0.0001	0.0499641
WGS	ABySS	XLOC_027343	2253302:1021-5466	cdRNA03-Dia-R2	cdRNA05-postDia	2.29447	14.5646	2.66623	5.00E-05	0.0289221
WGS	ABySS	XLOC_027381	2253360:45-3863	cdRNA01-preDia	cdRNA04-Dia-R3	5.98493	31.4985	2.39588	5.00E-05	0.0289221
WGS	ABySS	XLOC_027381	2253360:45-3863	cdRNA01-preDia	cdRNA03-Dia-R2	5.98493	34.3608	2.52136	0.0001	0.0499641
WGS	ABySS	XLOC_028363	2256514:6801-14103	cdRNA01-preDia	cdRNA05-postDia	39.9726	210.415	2.39615	5.00E-05	0.0289221
WGS	ABySS	XLOC_028363	2256514:6801-14103	cdRNA03-Dia-R2	cdRNA05-postDia	38.8083	210.415	2.4388	5.00E-05	0.0289221
WGS	ABySS	XLOC_028363	2256514:6801-14103	cdRNA04-Dia-R3	cdRNA05-postDia	35.2847	210.415	2.57612	5.00E-05	0.0289221
WGS	ABySS	XLOC_028637	2257279:14800-16988	cdRNA01-preDia	cdRNA02-Dia-R1	1.9381	33.6129	4.1163	0.0001	0.0499641
WGS	ABySS	XLOC_028735	2257734:0-928	cdRNA02-Dia-R1	cdRNA05-postDia	5.74437	76.7947	3.74078	5.00E-05	0.0289221
WGS	ABySS	XLOC_028735	2257734:0-928	cdRNA01-preDia	cdRNA05-postDia	4.77365	76.7947	4.00784	5.00E-05	0.0289221
WGS	ABySS	XLOC_028837	2258027:3-6105	cdRNA02-Dia-R1	cdRNA05-postDia	7.78911	56.7629	2.86542	5.00E-05	0.0289221
WGS	ABySS	XLOC_028837	2258027:3-6105	cdRNA01-preDia	cdRNA05-postDia	1.97166	56.7629	4.84747	5.00E-05	0.0289221
WGS	ABySS	XLOC_028837	2258027:3-6105	cdRNA04-Dia-R3	cdRNA05-postDia	1.35946	56.7629	5.38385	5.00E-05	0.0289221
WGS	ABySS	XLOC_028837	2258027:3-6105	cdRNA03-Dia-R2	cdRNA05-postDia	1.2544	56.7629	5.49988	5.00E-05	0.0289221
WGS	ABySS	XLOC_028859	2258082:1-2594	cdRNA04-Dia-R3	cdRNA05-postDia	28.1521	173.39	2.62271	5.00E-05	0.0289221
WGS	ABySS	XLOC_028859	2258082:1-2594	cdRNA02-Dia-R1	cdRNA05-postDia	25.52	173.39	2.76432	5.00E-05	0.0289221
WGS	ABySS	XLOC_028859	2258082:1-2594	cdRNA03-Dia-R2	cdRNA05-postDia	24.7868	173.39	2.80637	5.00E-05	0.0289221

WGS	ABySS	XLOC_028859	2258082:1-2594	cdRNA01-preDia	cdRNA05-postDia	20.1371	173.39	3.10609	5.00E-05	0.0289221
WGS	ABySS	XLOC_028937	2258306:4235-5628	cdRNA03-Dia-R2	cdRNA05-postDia	12.0923	70.3443	2.54035	0.0001	0.0499641
WGS	ABySS	XLOC_028937	2258306:4235-5628	cdRNA04-Dia-R3	cdRNA05-postDia	11.1071	70.3443	2.66295	5.00E-05	0.0289221
WGS	ABySS	XLOC_028937	2258306:4235-5628	cdRNA01-preDia	cdRNA05-postDia	4.4422	70.3443	3.98509	5.00E-05	0.0289221
WGS	ABySS	XLOC_028939	2258314:207-20087	cdRNA03-Dia-R2	cdRNA05-postDia	5.15342	26.7718	2.37711	0.0001	0.0499641
WGS	ABySS	XLOC_029085	2258929:3-3557	cdRNA03-Dia-R2	cdRNA05-postDia	5.10923	35.6363	2.80217	5.00E-05	0.0289221
WGS	ABySS	XLOC_029085	2258929:3-3557	cdRNA04-Dia-R3	cdRNA05-postDia	5.09717	35.6363	2.80558	5.00E-05	0.0289221
WGS	ABySS	XLOC_029331	2259873:7495-12606	cdRNA04-Dia-R3	cdRNA05-postDia	20.3912	2.64168	-2.94842	5.00E-05	0.0289221
WGS	ABySS	XLOC_029331	2259873:7495-12606	cdRNA03-Dia-R2	cdRNA05-postDia	18.2776	2.64168	-2.79055	5.00E-05	0.0289221
WGS	ABySS	XLOC_029367	2259990:0-1735	cdRNA02-Dia-R1	cdRNA05-postDia	2.06268	24.8746	3.59208	5.00E-05	0.0289221
WGS	ABySS	XLOC_029367	2259990:0-1735	cdRNA03-Dia-R2	cdRNA05-postDia	1.46036	24.8746	4.09027	5.00E-05	0.0289221
WGS	ABySS	XLOC_029459	2260170:44004-47366	cdRNA02-Dia-R1	cdRNA05-postDia	2.56521	23.9007	3.2199	5.00E-05	0.0289221
WGS	ABySS	XLOC_029459	2260170:44004-47366	cdRNA04-Dia-R3	cdRNA05-postDia	2.48551	23.9007	3.26544	5.00E-05	0.0289221
WGS	ABySS	XLOC_029459	2260170:44004-47366	cdRNA03-Dia-R2	cdRNA05-postDia	2.13207	23.9007	3.48673	5.00E-05	0.0289221
WGS	ABySS	XLOC_029482	2260205:1162-8164	cdRNA01-preDia	cdRNA05-postDia	12.1031	0.714582	-4.08214	5.00E-05	0.0289221
WGS	ABySS	XLOC_029482	2260205:1162-8164	cdRNA02-Dia-R1	cdRNA05-postDia	9.70932	0.714582	-3.7642	0.0001	0.0499641
WGS	ABySS	XLOC_029694	2260850:30736-39095	cdRNA04-Dia-R3	cdRNA05-postDia	8.23867	54.8776	2.73573	5.00E-05	0.0289221
WGS	ABySS	XLOC_029694	2260850:30736-39095	cdRNA02-Dia-R1	cdRNA05-postDia	7.29836	54.8776	2.91057	5.00E-05	0.0289221
WGS	ABySS	XLOC_029694	2260850:30736-39095	cdRNA03-Dia-R2	cdRNA05-postDia	6.76987	54.8776	3.01902	5.00E-05	0.0289221
WGS	ABySS	XLOC_029800	2261089:9171-9628	cdRNA03-Dia-R2	cdRNA05-postDia	163.045	20.454	-2.99482	5.00E-05	0.0289221
WGS	ABySS	XLOC_029800	2261089:9171-9628	cdRNA04-Dia-R3	cdRNA05-postDia	157.598	20.454	-2.94579	5.00E-05	0.0289221
WGS	ABySS	XLOC_029800	2261089:9171-9628	cdRNA02-Dia-R1	cdRNA05-postDia	106.549	20.454	-2.38106	0.0001	0.0499641
WGS	ABySS	XLOC_030380	2262173:10685-16659	cdRNA03-Dia-R2	cdRNA05-postDia	63.0861	7.79824	-3.0161	5.00E-05	0.0289221
WGS	ABySS	XLOC_030380	2262173:10685-16659	cdRNA04-Dia-R3	cdRNA05-postDia	61.1415	7.79824	-2.97093	5.00E-05	0.0289221
WGS	ABySS	XLOC_030649	2262683:18-477	cdRNA02-Dia-R1	cdRNA05-postDia	13.0623	314.467	4.58943	5.00E-05	0.0289221
WGS	ABySS	XLOC_030744	2262833:612-4441	cdRNA03-Dia-R2	cdRNA05-postDia	37.7962	8.01692	-2.23712	5.00E-05	0.0289221
WGS	ABySS	XLOC_030744	2262833:612-4441	cdRNA04-Dia-R3	cdRNA05-postDia	35.6397	8.01692	-2.15236	5.00E-05	0.0289221
WGS	ABySS	XLOC_030839	2262969:7923-10579	cdRNA03-Dia-R2	cdRNA05-postDia	28.2273	2.65298	-3.41141	5.00E-05	0.0289221
WGS	ABySS	XLOC_030839	2262969:7923-10579	cdRNA04-Dia-R3	cdRNA05-postDia	27.8673	2.65298	-3.39289	5.00E-05	0.0289221
WGS	ABySS	XLOC_030839	2262969:7923-10579	cdRNA02-Dia-R1	cdRNA05-postDia	17.8764	2.65298	-2.75237	5.00E-05	0.0289221
WGS	ABySS	XLOC_030906	2263008:1854-4013	cdRNA02-Dia-R1	cdRNA05-postDia	5.5432	26.4832	2.25629	0.0001	0.0499641
WGS	ABySS	XLOC_031015	2263059:49855-53435	cdRNA01-preDia	cdRNA05-postDia	5.09516	32.8224	2.68748	0.0001	0.0499641
WGS	ABySS	XLOC_031015	2263059:49855-53435	cdRNA02-Dia-R1	cdRNA05-postDia	3.36276	32.8224	3.28697	5.00E-05	0.0289221
WGS	ABySS	XLOC_031051	2263079:19533-21237	cdRNA03-Dia-R2	cdRNA05-postDia	3.87786	21.2456	2.45383	0.0001	0.0499641
WGS	ABySS	XLOC_031051	2263079:19533-21237	cdRNA02-Dia-R1	cdRNA05-postDia	2.85562	21.2456	2.89529	5.00E-05	0.0289221
WGS	ABySS	XLOC_031051	2263079:19533-21237	cdRNA01-preDia	cdRNA05-postDia	1.84624	21.2456	3.5245	5.00E-05	0.0289221
WGS	ABySS	XLOC_031558	2263355:475-8784	cdRNA03-Dia-R2	cdRNA05-postDia	18.8226	2.93476	-2.68115	0.0001	0.0499641
WGS	ABySS	XLOC_031711	2263443:565-3451	cdRNA03-Dia-R2	cdRNA05-postDia	12.495	1.73758	-2.8462	5.00E-05	0.0289221
WGS	ABySS	XLOC_031711	2263443:565-3451	cdRNA04-Dia-R3	cdRNA05-postDia	11.4516	1.73758	-2.7204	5.00E-05	0.0289221
WGS	ABySS	XLOC_031745	2263447:2466-6621	cdRNA01-preDia	cdRNA05-postDia	20.4821	134.202	2.71197	5.00E-05	0.0289221
WGS	ABySS	XLOC_031745	2263447:2466-6621	cdRNA04-Dia-R3	cdRNA05-postDia	18.5812	134.202	2.85249	5.00E-05	0.0289221
WGS	ABySS	XLOC_031745	2263447:2466-6621	cdRNA03-Dia-R2	cdRNA05-postDia	17.7984	134.202	2.91458	5.00E-05	0.0289221
WGS	ABySS	XLOC_031745	2263447:2466-6621	cdRNA02-Dia-R1	cdRNA05-postDia	14.807	134.202	3.18005	5.00E-05	0.0289221
WGS	ABySS	XLOC_031797	2263468:6703-8575	cdRNA02-Dia-R1	cdRNA05-postDia	0.624207	9.71835	3.96062	0.0001	0.0499641
WGS	ABySS	XLOC_032093	2263628:4665-8143	cdRNA02-Dia-R1	cdRNA05-postDia	11.8163	67.9495	2.52369	0.0001	0.0499641
WGS	ABySS	XLOC_032093	2263628:4665-8143	cdRNA01-preDia	cdRNA05-postDia	4.81928	67.9495	3.81757	5.00E-05	0.0289221
WGS	ABySS	XLOC_032093	2263628:4665-8143	cdRNA03-Dia-R2	cdRNA05-postDia	3.29896	67.9495	4.36438	5.00E-05	0.0289221

WGS	ABySS	XLOC_032093	2263628:4665-8143	cdRNA04-Dia-R3	cdRNA05-postDia	2.85743	67.9495	4.57167	5.00E-05	0.0289221
WGS	ABySS	XLOC_032268	2263751:67554-67800	cdRNA01-preDia	cdRNA04-Dia-R3	26.158	276.953	3.40432	5.00E-05	0.0289221
WGS	ABySS	XLOC_032268	2263751:67554-67800	cdRNA01-preDia	cdRNA03-Dia-R2	26.158	283.684	3.43896	5.00E-05	0.0289221
WGS	ABySS	XLOC_032284	2263769:2076-6207	cdRNA01-preDia	cdRNA05-postDia	2.48326	16.6589	2.74599	5.00E-05	0.0289221
WGS	ABySS	XLOC_032284	2263769:2076-6207	cdRNA02-Dia-R1	cdRNA05-postDia	2.37424	16.6589	2.81075	5.00E-05	0.0289221
WGS	ABySS	XLOC_032284	2263769:2076-6207	cdRNA03-Dia-R2	cdRNA05-postDia	1.6635	16.6589	3.324	5.00E-05	0.0289221
WGS	ABySS	XLOC_032284	2263769:2076-6207	cdRNA04-Dia-R3	cdRNA05-postDia	1.20363	16.6589	3.79083	5.00E-05	0.0289221
WGS	ABySS	XLOC_032621	2263942:17841-23395	cdRNA03-Dia-R2	cdRNA05-postDia	1.2142	9.29932	2.93712	0.0001	0.0499641
WGS	ABySS	XLOC_032635	2263946:91444-93686	cdRNA01-preDia	cdRNA03-Dia-R2	2.81372	17.0794	2.60171	5.00E-05	0.0289221
WGS	ABySS	XLOC_032776	2264005:61942-62283	cdRNA01-preDia	cdRNA03-Dia-R2	494.935	74.0496	-2.74067	5.00E-05	0.0289221
WGS	ABySS	XLOC_032776	2264005:61942-62283	cdRNA01-preDia	cdRNA04-Dia-R3	494.935	74.9263	-2.72369	5.00E-05	0.0289221
WGS	ABySS	XLOC_032776	2264005:61942-62283	cdRNA01-preDia	cdRNA05-postDia	494.935	103.328	-2.26001	0.0001	0.0499641
WGS	ABySS	XLOC_032801	2264026:2643-4742	cdRNA01-preDia	cdRNA03-Dia-R2	19.5843	103.83	2.40646	5.00E-05	0.0289221
WGS	ABySS	XLOC_032801	2264026:2643-4742	cdRNA01-preDia	cdRNA04-Dia-R3	19.5843	111.857	2.51388	5.00E-05	0.0289221
WGS	ABySS	XLOC_032938	2264098:18747-24820	cdRNA01-preDia	cdRNA04-Dia-R3	19.194	2.26132	-3.08542	5.00E-05	0.0289221
WGS	ABySS	XLOC_032938	2264098:18747-24820	cdRNA01-preDia	cdRNA03-Dia-R2	19.194	2.55454	-2.90952	5.00E-05	0.0289221
WGS	ABySS	XLOC_032965	2264118:79267-82688	cdRNA03-Dia-R2	cdRNA05-postDia	5.48189	32.1243	2.55092	5.00E-05	0.0289221
WGS	ABySS	XLOC_032965	2264118:79267-82688	cdRNA04-Dia-R3	cdRNA05-postDia	4.85665	32.1243	2.72563	5.00E-05	0.0289221
WGS	ABySS	XLOC_033279	2264334:4450-9051	cdRNA01-preDia	cdRNA02-Dia-R1	20.5522	179.379	3.12565	5.00E-05	0.0289221
WGS	ABySS	XLOC_033279	2264334:4450-9051	cdRNA01-preDia	cdRNA05-postDia	20.5522	392.366	4.25484	5.00E-05	0.0289221
WGS	ABySS	XLOC_033308	2264357:983-8423	cdRNA01-preDia	cdRNA02-Dia-R1	4.92316	28.3716	2.52679	0.0001	0.0499641
WGS	ABySS	XLOC_033308	2264357:983-8423	cdRNA01-preDia	cdRNA05-postDia	4.92316	48.1814	3.29082	5.00E-05	0.0289221
WGS	ABySS	XLOC_033372	2264416:9920-18630	cdRNA04-Dia-R3	cdRNA05-postDia	3.35546	18.0399	2.42661	5.00E-05	0.0289221
WGS	ABySS	XLOC_033372	2264416:9920-18630	cdRNA03-Dia-R2	cdRNA05-postDia	3.18895	18.0399	2.50004	5.00E-05	0.0289221
WGS	ABySS	XLOC_033474	2264445:103395-104680	cdRNA01-preDia	cdRNA05-postDia	29.2015	4.37236	-2.73956	5.00E-05	0.0289221
WGS	ABySS	XLOC_033545	2264474:6-1563	cdRNA04-Dia-R3	cdRNA05-postDia	2.37624	15.5246	2.7078	5.00E-05	0.0289221
WGS	ABySS	XLOC_033545	2264474:6-1563	cdRNA03-Dia-R2	cdRNA05-postDia	1.93946	15.5246	3.00083	5.00E-05	0.0289221
WGS	ABySS	XLOC_034062	2264765:24308-29686	cdRNA02-Dia-R1	cdRNA05-postDia	13.5274	70.9792	2.39152	5.00E-05	0.0289221
WGS	ABySS	XLOC_034062	2264765:24308-29686	cdRNA04-Dia-R3	cdRNA05-postDia	13.2348	70.9792	2.42306	5.00E-05	0.0289221
WGS	ABySS	XLOC_034062	2264765:24308-29686	cdRNA03-Dia-R2	cdRNA05-postDia	13.1781	70.9792	2.42925	5.00E-05	0.0289221
WGS	ABySS	XLOC_034062	2264765:24308-29686	cdRNA01-preDia	cdRNA05-postDia	8.65433	70.9792	3.0359	5.00E-05	0.0289221
WGS	ABySS	XLOC_034088	2264777:222-4487	cdRNA01-preDia	cdRNA05-postDia	1.73915	14.0273	3.01178	5.00E-05	0.0289221
WGS	ABySS	XLOC_034088	2264777:222-4487	cdRNA04-Dia-R3	cdRNA05-postDia	1.2347	14.0273	3.506	5.00E-05	0.0289221
WGS	ABySS	XLOC_034214	2264850:494-2003	cdRNA02-Dia-R1	cdRNA05-postDia	2.43663	14.3461	2.55769	0.0001	0.0499641
WGS	ABySS	XLOC_034214	2264850:494-2003	cdRNA03-Dia-R2	cdRNA05-postDia	2.15865	14.3461	2.73246	5.00E-05	0.0289221
WGS	ABySS	XLOC_034280	2264899:34142-50664	cdRNA03-Dia-R2	cdRNA05-postDia	4.88614	35.0264	2.84167	5.00E-05	0.0289221
WGS	ABySS	XLOC_034280	2264899:34142-50664	cdRNA04-Dia-R3	cdRNA05-postDia	4.63576	35.0264	2.91756	5.00E-05	0.0289221
WGS	ABySS	XLOC_034364	2264934:97618-107162	cdRNA03-Dia-R2	cdRNA05-postDia	16.3004	92.2487	2.50062	5.00E-05	0.0289221
WGS	ABySS	XLOC_034420	2264968:17473-19441	cdRNA01-preDia	cdRNA05-postDia	65.7143	12.6146	-2.38111	5.00E-05	0.0289221
WGS	ABySS	XLOC_034499	2265014:5773-9215	cdRNA01-preDia	cdRNA03-Dia-R2	52.4695	10.063	-2.38242	0.0001	0.0499641
WGS	ABySS	XLOC_034499	2265014:5773-9215	cdRNA01-preDia	cdRNA04-Dia-R3	52.4695	10.6218	-2.30445	0.0001	0.0499641
WGS	ABySS	XLOC_034501	2265016:12841-15377	cdRNA03-Dia-R2	cdRNA05-postDia	16.4469	2.44472	-2.75007	5.00E-05	0.0289221
WGS	ABySS	XLOC_034501	2265016:12841-15377	cdRNA04-Dia-R3	cdRNA05-postDia	15.3968	2.44472	-2.65489	0.0001	0.0499641
WGS	ABySS	XLOC_034675	2265119:34053-36972	cdRNA01-preDia	cdRNA03-Dia-R2	22.5418	138.918	2.62356	5.00E-05	0.0289221
WGS	ABySS	XLOC_034675	2265119:34053-36972	cdRNA01-preDia	cdRNA04-Dia-R3	22.5418	147.474	2.70978	5.00E-05	0.0289221
WGS	ABySS	XLOC_034675	2265119:34053-36972	cdRNA01-preDia	cdRNA05-postDia	22.5418	153.376	2.7664	5.00E-05	0.0289221
WGS	ABySS	XLOC_034761	2265176:108504-113134	cdRNA01-preDia	cdRNA05-postDia	2.20996	14.9165	2.75482	0.0001	0.0499641

WGS	ABySS	XLOC_035364	2265546:72-3248	cdRNA01-preDia	cdRNA03-Dia-R2	9.29533	58.705	2.65891	5.00E-05	0.0289221
WGS	ABySS	XLOC_035364	2265546:72-3248	cdRNA01-preDia	cdRNA04-Dia-R3	9.29533	59.0667	2.66777	5.00E-05	0.0289221
WGS	ABySS	XLOC_035433	2265576:17981-20986	cdRNA02-Dia-R1	cdRNA05-postDia	3.57583	19.5519	2.45096	0.0001	0.0499641
WGS	ABySS	XLOC_035433	2265576:17981-20986	cdRNA03-Dia-R2	cdRNA05-postDia	3.02119	19.5519	2.69412	5.00E-05	0.0289221
WGS	ABySS	XLOC_035450	2265584:2277-7129	cdRNA03-Dia-R2	cdRNA05-postDia	69.4688	8.4617	-3.03735	5.00E-05	0.0289221
WGS	ABySS	XLOC_035450	2265584:2277-7129	cdRNA04-Dia-R3	cdRNA05-postDia	65.2805	8.4617	-2.94763	5.00E-05	0.0289221
WGS	ABySS	XLOC_035576	2265649:4389-7435	cdRNA01-preDia	cdRNA05-postDia	2.99411	18.2355	2.60655	0.0001	0.0499641
WGS	ABySS	XLOC_035576	2265649:4389-7435	cdRNA04-Dia-R3	cdRNA05-postDia	2.66868	18.2355	2.77255	0.0001	0.0499641
WGS	ABySS	XLOC_035576	2265649:4389-7435	cdRNA03-Dia-R2	cdRNA05-postDia	2.37358	18.2355	2.94161	5.00E-05	0.0289221
WGS	ABySS	XLOC_036116	2265922:164-752	cdRNA02-Dia-R1	cdRNA05-postDia	26.7922	227.925	3.08867	5.00E-05	0.0289221
WGS	ABySS	XLOC_036116	2265922:164-752	cdRNA01-preDia	cdRNA05-postDia	10.0387	227.925	4.50491	5.00E-05	0.0289221
WGS	ABySS	XLOC_036116	2265922:164-752	cdRNA03-Dia-R2	cdRNA05-postDia	9.00253	227.925	4.66209	5.00E-05	0.0289221
WGS	ABySS	XLOC_036116	2265922:164-752	cdRNA04-Dia-R3	cdRNA05-postDia	6.75451	227.925	5.07656	5.00E-05	0.0289221
WGS	ABySS	XLOC_036228	2265978:792-5388	cdRNA01-preDia	cdRNA05-postDia	2.15047	15.4759	2.8473	0.0001	0.0499641
WGS	ABySS	XLOC_036716	2266223:19796-23485	cdRNA01-preDia	cdRNA05-postDia	21.6184	1.39918	-3.94961	5.00E-05	0.0289221
WGS	ABySS	XLOC_036716	2266223:19796-23485	cdRNA02-Dia-R1	cdRNA05-postDia	18.5189	1.39918	-3.72635	5.00E-05	0.0289221
WGS	ABySS	XLOC_036716	2266223:19796-23485	cdRNA03-Dia-R2	cdRNA05-postDia	15.9902	1.39918	-3.51454	0.0001	0.0499641
WGS	ABySS	XLOC_036716	2266223:19796-23485	cdRNA04-Dia-R3	cdRNA05-postDia	15.0004	1.39918	-3.42235	0.0001	0.0499641
WGS	ABySS	XLOC_036859	2266277:43852-46094	cdRNA01-preDia	cdRNA05-postDia	22.7556	117.642	2.37011	5.00E-05	0.0289221
WGS	ABySS	XLOC_036930	2266309:328-2642	cdRNA01-preDia	cdRNA05-postDia	7.89778	142.299	4.17134	5.00E-05	0.0289221
WGS	ABySS	XLOC_036930	2266309:328-2642	cdRNA02-Dia-R1	cdRNA05-postDia	6.05842	142.299	4.55384	5.00E-05	0.0289221
WGS	ABySS	XLOC_036930	2266309:328-2642	cdRNA04-Dia-R3	cdRNA05-postDia	1.12584	142.299	6.98178	5.00E-05	0.0289221
WGS	ABySS	XLOC_036930	2266309:328-2642	cdRNA03-Dia-R2	cdRNA05-postDia	0.952582	142.299	7.22287	5.00E-05	0.0289221
WGS	ABySS	XLOC_037689	2266757:7164-11952	cdRNA01-preDia	cdRNA03-Dia-R2	10.7517	67.5815	2.65207	5.00E-05	0.0289221
WGS	ABySS	XLOC_037689	2266757:7164-11952	cdRNA01-preDia	cdRNA04-Dia-R3	10.7517	70.2286	2.7075	5.00E-05	0.0289221
WGS	ABySS	XLOC_037689	2266757:7164-11952	cdRNA01-preDia	cdRNA02-Dia-R1	10.7517	127.069	3.56298	5.00E-05	0.0289221
WGS	ABySS	XLOC_037689	2266757:7164-11952	cdRNA01-preDia	cdRNA05-postDia	10.7517	361.499	5.07136	5.00E-05	0.0289221
WGS	ABySS	XLOC_037799	2266834:62972-65212	cdRNA04-Dia-R3	cdRNA05-postDia	16.827	95.6279	2.50666	5.00E-05	0.0289221
WGS	ABySS	XLOC_037799	2266834:62972-65212	cdRNA03-Dia-R2	cdRNA05-postDia	16.7624	95.6279	2.5122	5.00E-05	0.0289221
WGS	ABySS	XLOC_037928	2266917:1310-9192	cdRNA01-preDia	cdRNA04-Dia-R3	44.1687	9.73045	-2.18244	5.00E-05	0.0289221
WGS	ABySS	XLOC_038554	2267230:1237-2667	cdRNA02-Dia-R1	cdRNA05-postDia	3.32276	46.9186	3.81971	5.00E-05	0.0289221
WGS	ABySS	XLOC_038569	2267243:20-1258	cdRNA04-Dia-R3	cdRNA05-postDia	21.4948	107.922	2.32793	0.0001	0.0499641
WGS	ABySS	XLOC_038594	2267262:1928-17581	cdRNA01-preDia	cdRNA03-Dia-R2	13.6713	67.7652	2.30939	0.0001	0.0499641
WGS	ABySS	XLOC_038646	2267283:76764-92773	cdRNA01-preDia	cdRNA03-Dia-R2	9.72071	40.8858	2.07247	0.0001	0.0499641
WGS	ABySS	XLOC_038874	2267363:4257-12620	cdRNA01-preDia	cdRNA03-Dia-R2	4.59044	27.515	2.58351	5.00E-05	0.0289221
WGS	ABySS	XLOC_038874	2267363:4257-12620	cdRNA01-preDia	cdRNA04-Dia-R3	4.59044	28.9208	2.6554	5.00E-05	0.0289221
WGS	ABySS	XLOC_038993	2267404:5132-8193	cdRNA01-preDia	cdRNA02-Dia-R1	22.6264	0.883876	-4.67802	0.0001	0.0499641
WGS	ABySS	XLOC_038994	2267404:8455-10144	cdRNA01-preDia	cdRNA02-Dia-R1	20.3201	0.989722	-4.35974	5.00E-05	0.0289221
WGS	ABySS	XLOC_039016	2267417:4538-8535	cdRNA01-preDia	cdRNA05-postDia	1.93318	21.5921	3.48146	5.00E-05	0.0289221
WGS	ABySS	XLOC_039111	2267469:20893-22694	cdRNA01-preDia	cdRNA03-Dia-R2	23.2205	2.73717	-3.08464	5.00E-05	0.0289221
WGS	ABySS	XLOC_039111	2267469:20893-22694	cdRNA01-preDia	cdRNA04-Dia-R3	23.2205	2.77063	-3.06711	5.00E-05	0.0289221
WGS	ABySS	XLOC_039111	2267469:20893-22694	cdRNA04-Dia-R3	cdRNA05-postDia	2.77063	22.8726	3.04533	5.00E-05	0.0289221
WGS	ABySS	XLOC_039111	2267469:20893-22694	cdRNA03-Dia-R2	cdRNA05-postDia	2.73717	22.8726	3.06286	5.00E-05	0.0289221
WGS	ABySS	XLOC_039760	2267740:3211-8111	cdRNA03-Dia-R2	cdRNA05-postDia	59.5896	609.283	3.35398	5.00E-05	0.0289221
WGS	ABySS	XLOC_039760	2267740:3211-8111	cdRNA04-Dia-R3	cdRNA05-postDia	57.7154	609.283	3.40009	5.00E-05	0.0289221
WGS	ABySS	XLOC_039760	2267740:3211-8111	cdRNA01-preDia	cdRNA05-postDia	22.8397	609.283	4.7375	5.00E-05	0.0289221
WGS	ABySS	XLOC_039761	2267740:13870-18009	cdRNA01-preDia	cdRNA05-postDia	54.1692	338.535	2.64376	5.00E-05	0.0289221

WGS	ABySS	XLOC_039964	2267857:16451-19969	cdRNA01-preDia	cdRNA05-postDia	2.64309	15.1345	2.51754	0.0001	0.0499641
WGS	ABySS	XLOC_039964	2267857:16451-19969	cdRNA01-preDia	cdRNA02-Dia-R1	2.64309	16.2855	2.62329	5.00E-05	0.0289221
WGS	ABySS	XLOC_040168	2267953:4239-10848	cdRNA02-Dia-R1	cdRNA05-postDia	13.8392	82.1932	2.57026	5.00E-05	0.0289221
WGS	ABySS	XLOC_040168	2267953:4239-10848	cdRNA03-Dia-R2	cdRNA05-postDia	11.5818	82.1932	2.82716	5.00E-05	0.0289221
WGS	ABySS	XLOC_040168	2267953:4239-10848	cdRNA04-Dia-R3	cdRNA05-postDia	11.4946	82.1932	2.83806	5.00E-05	0.0289221
WGS	ABySS	XLOC_040360	2268078:4-4707	cdRNA04-Dia-R3	cdRNA05-postDia	32.9158	4.63576	-2.8279	5.00E-05	0.0289221
WGS	ABySS	XLOC_040360	2268078:4-4707	cdRNA03-Dia-R2	cdRNA05-postDia	32.1031	4.63576	-2.79183	5.00E-05	0.0289221
WGS	ABySS	XLOC_040644	2268196:212039-217879	cdRNA02-Dia-R1	cdRNA05-postDia	6.18631	28.7763	2.21773	5.00E-05	0.0289221
WGS	ABySS	XLOC_040644	2268196:212039-217879	cdRNA01-preDia	cdRNA05-postDia	5.98192	28.7763	2.2662	5.00E-05	0.0289221
WGS	ABySS	XLOC_040713	2268242:57-2218	cdRNA01-preDia	cdRNA03-Dia-R2	1.27827	24.6287	4.26808	5.00E-05	0.0289221
WGS	ABySS	XLOC_040713	2268242:57-2218	cdRNA01-preDia	cdRNA04-Dia-R3	1.27827	25.4925	4.31781	5.00E-05	0.0289221
WGS	ABySS	XLOC_040779	2268282:83350-91450	cdRNA01-preDia	cdRNA03-Dia-R2	50.8303	9.18123	-2.46893	5.00E-05	0.0289221
WGS	ABySS	XLOC_040779	2268282:83350-91450	cdRNA01-preDia	cdRNA04-Dia-R3	50.8303	9.21979	-2.46288	5.00E-05	0.0289221
WGS	ABySS	XLOC_040827	2268311:11515-14742	cdRNA01-preDia	cdRNA05-postDia	1.75995	11.4849	2.70614	5.00E-05	0.0289221
WGS	ABySS	XLOC_040883	2268345:6-3707	cdRNA01-preDia	cdRNA05-postDia	53.5137	339.58	2.66577	0.0001	0.0499641
WGS	ABySS	XLOC_041178	2268486:6371-18195	cdRNA04-Dia-R3	cdRNA05-postDia	11.3065	1.1387	-3.3117	5.00E-05	0.0289221
WGS	ABySS	XLOC_041178	2268486:6371-18195	cdRNA03-Dia-R2	cdRNA05-postDia	10.6934	1.1387	-3.23126	5.00E-05	0.0289221
WGS	ABySS	XLOC_041178	2268486:6371-18195	cdRNA01-preDia	cdRNA04-Dia-R3	1.99574	11.3065	2.50216	0.0001	0.0499641
WGS	ABySS	XLOC_041182	2268486:18258-22636	cdRNA04-Dia-R3	cdRNA05-postDia	13.0413	2.00563	-2.70096	5.00E-05	0.0289221
WGS	ABySS	XLOC_041182	2268486:18258-22636	cdRNA03-Dia-R2	cdRNA05-postDia	12.8509	2.00563	-2.67974	5.00E-05	0.0289221
WGS	ABySS	XLOC_041182	2268486:18258-22636	cdRNA01-preDia	cdRNA03-Dia-R2	0.700747	12.8509	4.19683	5.00E-05	0.0289221
WGS	ABySS	XLOC_041182	2268486:18258-22636	cdRNA01-preDia	cdRNA04-Dia-R3	0.700747	13.0413	4.21805	5.00E-05	0.0289221
WGS	ABySS	XLOC_041292	2268545:68160-72265	cdRNA04-Dia-R3	cdRNA05-postDia	13.2268	65.929	2.31745	0.0001	0.0499641
WGS	ABySS	XLOC_041292	2268545:68160-72265	cdRNA02-Dia-R1	cdRNA05-postDia	13.0457	65.929	2.33734	5.00E-05	0.0289221
WGS	ABySS	XLOC_041292	2268545:68160-72265	cdRNA03-Dia-R2	cdRNA05-postDia	12.9078	65.929	2.35267	5.00E-05	0.0289221
WGS	ABySS	XLOC_041877	2268814:476-32232	cdRNA03-Dia-R2	cdRNA05-postDia	30.0314	6.57157	-2.19216	5.00E-05	0.0289221
WGS	ABySS	XLOC_041878	2268814:35818-41270	cdRNA03-Dia-R2	cdRNA05-postDia	51.9141	8.6533	-2.5848	0.0001	0.0499641
WGS	ABySS	XLOC_042198	2268979:48336-53347	cdRNA03-Dia-R2	cdRNA05-postDia	1.92612	11.2414	2.54505	0.0001	0.0499641
WGS	ABySS	XLOC_042554	2269131:34150-35058	cdRNA01-preDia	cdRNA03-Dia-R2	5.02857	47.5775	3.24206	5.00E-05	0.0289221
WGS	ABySS	XLOC_042554	2269131:34150-35058	cdRNA01-preDia	cdRNA04-Dia-R3	5.02857	49.9084	3.31106	5.00E-05	0.0289221
WGS	ABySS	XLOC_042841	2269310:3211-8100	cdRNA04-Dia-R3	cdRNA05-postDia	6.6116	52.3593	2.98537	5.00E-05	0.0289221
WGS	ABySS	XLOC_042841	2269310:3211-8100	cdRNA02-Dia-R1	cdRNA05-postDia	5.54254	52.3593	3.23983	5.00E-05	0.0289221
WGS	ABySS	XLOC_042841	2269310:3211-8100	cdRNA03-Dia-R2	cdRNA05-postDia	5.5018	52.3593	3.25047	5.00E-05	0.0289221
WGS	ABySS	XLOC_042841	2269310:3211-8100	cdRNA01-preDia	cdRNA05-postDia	2.20379	52.3593	4.57039	5.00E-05	0.0289221
WGS	ABySS	XLOC_043088	2269468:74977-77522	cdRNA01-preDia	cdRNA05-postDia	235.833	26.0557	-3.17809	5.00E-05	0.0289221
WGS	ABySS	XLOC_043132	2269488:4036-24257	cdRNA04-Dia-R3	cdRNA05-postDia	7.28369	1.34442	-2.43768	5.00E-05	0.0289221
WGS	ABySS	XLOC_043132	2269488:4036-24257	cdRNA03-Dia-R2	cdRNA05-postDia	7.08485	1.34442	-2.39775	0.0001	0.0499641
WGS	ABySS	XLOC_043449	2269645:19442-38489	cdRNA03-Dia-R2	cdRNA05-postDia	1.46748	9.10112	2.6327	5.00E-05	0.0289221
WGS	ABySS	XLOC_043655	2269758:30166-32015	cdRNA01-preDia	cdRNA05-postDia	17.0757	2.85668	-2.57953	5.00E-05	0.0289221
WGS	ABySS	XLOC_044005	2269964:0-1667	cdRNA01-preDia	cdRNA05-postDia	2.63059	27.7523	3.39915	5.00E-05	0.0289221
WGS	ABySS	XLOC_044006	2269964:1739-3143	cdRNA01-preDia	cdRNA05-postDia	1.60356	13.5402	3.0779	5.00E-05	0.0289221
WGS	ABySS	XLOC_044125	2270018:73936-78564	cdRNA02-Dia-R1	cdRNA05-postDia	3.27888	17.7879	2.43962	0.0001	0.0499641
WGS	ABySS	XLOC_044125	2270018:73936-78564	cdRNA04-Dia-R3	cdRNA05-postDia	2.28727	17.7879	2.95919	5.00E-05	0.0289221
WGS	ABySS	XLOC_044125	2270018:73936-78564	cdRNA03-Dia-R2	cdRNA05-postDia	1.90376	17.7879	3.22397	5.00E-05	0.0289221
WGS	ABySS	XLOC_044315	2270113:23018-26875	cdRNA01-preDia	cdRNA05-postDia	3.13032	18.8309	2.58872	0.0001	0.0499641
WGS	ABySS	XLOC_044345	2270126:3497-11602	cdRNA04-Dia-R3	cdRNA05-postDia	9.71075	70.2346	2.85453	5.00E-05	0.0289221
WGS	ABySS	XLOC_044345	2270126:3497-11602	cdRNA02-Dia-R1	cdRNA05-postDia	8.61923	70.2346	3.02655	5.00E-05	0.0289221

WGS	AByS	XLOC_044345	2270126:3497-11602	cdRNA03-Dia-R2	cdRNA05-postDia	8.27042	70.2346	3.08615	5.00E-05	0.0289221
WGS	AByS	XLOC_044345	2270126:3497-11602	cdRNA01-preDia	cdRNA05-postDia	2.8856	70.2346	4.60524	5.00E-05	0.0289221
WGS	AByS	XLOC_044348	2270127:6735-26514	cdRNA03-Dia-R2	cdRNA05-postDia	86.0819	15.1372	-2.50761	5.00E-05	0.0289221
WGS	AByS	XLOC_044348	2270127:6735-26514	cdRNA04-Dia-R3	cdRNA05-postDia	80.3964	15.1372	-2.40903	5.00E-05	0.0289221
WGS	AByS	XLOC_044492	2270215:18035-23284	cdRNA02-Dia-R1	cdRNA05-postDia	15.1416	338.561	4.48282	5.00E-05	0.0289221
WGS	AByS	XLOC_044492	2270215:18035-23284	cdRNA01-preDia	cdRNA05-postDia	5.47929	338.561	5.94928	5.00E-05	0.0289221
WGS	AByS	XLOC_044492	2270215:18035-23284	cdRNA04-Dia-R3	cdRNA05-postDia	3.92389	338.561	6.43099	5.00E-05	0.0289221
WGS	AByS	XLOC_044492	2270215:18035-23284	cdRNA03-Dia-R2	cdRNA05-postDia	3.76551	338.561	6.49042	5.00E-05	0.0289221
WGS	AByS	XLOC_044718	2270301:117514-123356	cdRNA03-Dia-R2	cdRNA05-postDia	4.68052	23.6195	2.33524	0.0001	0.0499641
WGS	AByS	XLOC_044718	2270301:117514-123356	cdRNA04-Dia-R3	cdRNA05-postDia	4.67013	23.6195	2.33844	0.0001	0.0499641
WGS	AByS	XLOC_045190	2270549:2623-9320	cdRNA01-preDia	cdRNA04-Dia-R3	7.18224	54.3958	2.92099	5.00E-05	0.0289221
WGS	AByS	XLOC_045190	2270549:2623-9320	cdRNA01-preDia	cdRNA03-Dia-R2	7.18224	55.0113	2.93722	5.00E-05	0.0289221
WGS	AByS	XLOC_045281	2270598:4234-14191	cdRNA01-preDia	cdRNA04-Dia-R3	9.17277	57.9992	2.6606	5.00E-05	0.0289221
WGS	AByS	XLOC_045281	2270598:4234-14191	cdRNA01-preDia	cdRNA03-Dia-R2	9.17277	61.451	2.74401	5.00E-05	0.0289221
WGS	AByS	XLOC_045404	2270634:2902-11967	cdRNA01-preDia	cdRNA02-Dia-R1	5.57477	30.5133	2.45245	5.00E-05	0.0289221
WGS	AByS	XLOC_045404	2270634:2902-11967	cdRNA01-preDia	cdRNA04-Dia-R3	5.57477	43.7258	2.9715	5.00E-05	0.0289221
WGS	AByS	XLOC_045404	2270634:2902-11967	cdRNA01-preDia	cdRNA03-Dia-R2	5.57477	45.9012	3.04155	5.00E-05	0.0289221
WGS	AByS	XLOC_045558	2270728:89274-96201	cdRNA04-Dia-R3	cdRNA05-postDia	10.8702	1.76454	-2.62302	0.0001	0.0499641
WGS	AByS	XLOC_045569	2270729:666-16258	cdRNA03-Dia-R2	cdRNA05-postDia	40.7533	231.876	2.50837	5.00E-05	0.0289221
WGS	AByS	XLOC_045696	2270793:9130-11497	cdRNA02-Dia-R1	cdRNA05-postDia	21.0717	110.34	2.38858	0.0001	0.0499641
WGS	AByS	XLOC_045735	2270826:15-3037	cdRNA02-Dia-R1	cdRNA05-postDia	14.0216	98.2263	2.80846	5.00E-05	0.0289221
WGS	AByS	XLOC_045735	2270826:15-3037	cdRNA04-Dia-R3	cdRNA05-postDia	8.23063	98.2263	3.57703	5.00E-05	0.0289221
WGS	AByS	XLOC_045735	2270826:15-3037	cdRNA03-Dia-R2	cdRNA05-postDia	5.91731	98.2263	4.0531	5.00E-05	0.0289221
WGS	AByS	XLOC_045735	2270826:15-3037	cdRNA01-preDia	cdRNA05-postDia	2.94045	98.2263	5.062	5.00E-05	0.0289221
WGS	AByS	XLOC_046016	2270981:11604-26851	cdRNA04-Dia-R3	cdRNA05-postDia	18.0868	4.13138	-2.13024	0.0001	0.0499641
WGS	AByS	XLOC_046157	2271098:2551-5011	cdRNA03-Dia-R2	cdRNA05-postDia	1.8203	13.7921	2.9216	5.00E-05	0.0289221
WGS	AByS	XLOC_046157	2271098:2551-5011	cdRNA04-Dia-R3	cdRNA05-postDia	1.798	13.7921	2.93938	5.00E-05	0.0289221
WGS	AByS	XLOC_046240	2271147:27572-29133	cdRNA01-preDia	cdRNA03-Dia-R2	68.3814	9.71677	-2.81505	5.00E-05	0.0289221
WGS	AByS	XLOC_046240	2271147:27572-29133	cdRNA01-preDia	cdRNA04-Dia-R3	68.3814	9.96742	-2.77831	5.00E-05	0.0289221
WGS	AByS	XLOC_046240	2271147:27572-29133	cdRNA04-Dia-R3	cdRNA05-postDia	9.96742	67.2436	2.75411	5.00E-05	0.0289221
WGS	AByS	XLOC_046240	2271147:27572-29133	cdRNA03-Dia-R2	cdRNA05-postDia	9.71677	67.2436	2.79085	0.0001	0.0499641
WGS	AByS	XLOC_046625	2271328:26988-33103	cdRNA01-preDia	cdRNA05-postDia	2.03217	12.9722	2.67433	0.0001	0.0499641
WGS	AByS	XLOC_046790	2271421:12697-14151	cdRNA01-preDia	cdRNA03-Dia-R2	9.91953	48.5257	2.2904	0.0001	0.0499641
WGS	AByS	XLOC_046809	2271439:11448-19830	cdRNA04-Dia-R3	cdRNA05-postDia	3.32791	18.6452	2.48612	0.0001	0.0499641
WGS	AByS	XLOC_046809	2271439:11448-19830	cdRNA03-Dia-R2	cdRNA05-postDia	3.09178	18.6452	2.59229	5.00E-05	0.0289221
WGS	AByS	XLOC_047161	2271587:41130-42584	cdRNA01-preDia	cdRNA05-postDia	5.01851	28.7065	2.51605	5.00E-05	0.0289221
WGS	AByS	XLOC_047239	2271624:112664-114343	cdRNA03-Dia-R2	cdRNA05-postDia	4.84266	30.3369	2.6472	5.00E-05	0.0289221
WGS	AByS	XLOC_047239	2271624:112664-114343	cdRNA04-Dia-R3	cdRNA05-postDia	4.02434	30.3369	2.91425	5.00E-05	0.0289221
WGS	AByS	XLOC_047381	2271681:4056-5922	cdRNA01-preDia	cdRNA04-Dia-R3	22.0289	104.57	2.247	0.0001	0.0499641
WGS	AByS	XLOC_047381	2271681:4056-5922	cdRNA01-preDia	cdRNA05-postDia	22.0289	127.283	2.53058	5.00E-05	0.0289221
WGS	AByS	XLOC_047638	2271836:46137-47232	cdRNA01-preDia	cdRNA05-postDia	5.60829	42.9415	2.93674	5.00E-05	0.0289221
WGS	AByS	XLOC_047761	2271915:926-4004	cdRNA02-Dia-R1	cdRNA05-postDia	22.7888	210.518	3.20755	5.00E-05	0.0289221
WGS	AByS	XLOC_047761	2271915:926-4004	cdRNA04-Dia-R3	cdRNA05-postDia	10.9837	210.518	4.26051	5.00E-05	0.0289221
WGS	AByS	XLOC_047761	2271915:926-4004	cdRNA01-preDia	cdRNA05-postDia	10.623	210.518	4.30869	5.00E-05	0.0289221
WGS	AByS	XLOC_047761	2271915:926-4004	cdRNA03-Dia-R2	cdRNA05-postDia	10.0277	210.518	4.39188	5.00E-05	0.0289221
WGS	AByS	XLOC_047917	2272019:3377-6030	cdRNA01-preDia	cdRNA03-Dia-R2	7.18035	44.1181	2.61924	5.00E-05	0.0289221
WGS	AByS	XLOC_047917	2272019:3377-6030	cdRNA01-preDia	cdRNA04-Dia-R3	7.18035	46.6797	2.70067	5.00E-05	0.0289221

WGS	ABySS	XLOC_048002	2272063:8631-11897	cdRNA02-Dia-R1	cdRNA05-postDia	29.0977	143.379	2.30086	5.00E-05	0.0289221
WGS	ABySS	XLOC_048002	2272063:8631-11897	cdRNA01-preDia	cdRNA05-postDia	20.0475	143.379	2.83834	5.00E-05	0.0289221
WGS	ABySS	XLOC_048034	2272086:9990-11222	cdRNA01-preDia	cdRNA05-postDia	17.8246	113.295	2.66815	5.00E-05	0.0289221
WGS	ABySS	XLOC_048034	2272086:9990-11222	cdRNA02-Dia-R1	cdRNA05-postDia	7.13111	113.295	3.98982	5.00E-05	0.0289221
WGS	ABySS	XLOC_048034	2272086:9990-11222	cdRNA04-Dia-R3	cdRNA05-postDia	6.23037	113.295	4.18463	5.00E-05	0.0289221
WGS	ABySS	XLOC_048034	2272086:9990-11222	cdRNA03-Dia-R2	cdRNA05-postDia	5.98029	113.295	4.24373	5.00E-05	0.0289221
WGS	ABySS	XLOC_048070	2272099:22558-25365	cdRNA04-Dia-R3	cdRNA05-postDia	173.083	30.3133	-2.51344	5.00E-05	0.0289221
WGS	ABySS	XLOC_048070	2272099:22558-25365	cdRNA03-Dia-R2	cdRNA05-postDia	166.149	30.3133	-2.45446	5.00E-05	0.0289221
WGS	ABySS	XLOC_048089	2272113:0-8819	cdRNA01-preDia	cdRNA05-postDia	2.8669	17.5149	2.61102	5.00E-05	0.0289221
WGS	ABySS	XLOC_048169	2272166:361-4114	cdRNA01-preDia	cdRNA05-postDia	3.687	25.0037	2.76162	5.00E-05	0.0289221
WGS	ABySS	XLOC_048181	2272176:55193-60064	cdRNA01-preDia	cdRNA02-Dia-R1	1.00289	7.02212	2.80774	5.00E-05	0.0289221
WGS	ABySS	XLOC_048181	2272176:55193-60064	cdRNA01-preDia	cdRNA03-Dia-R2	1.00289	15.9987	3.99572	5.00E-05	0.0289221
WGS	ABySS	XLOC_048181	2272176:55193-60064	cdRNA01-preDia	cdRNA04-Dia-R3	1.00289	16.7534	4.06221	5.00E-05	0.0289221
WGS	ABySS	XLOC_048181	2272176:55193-60064	cdRNA01-preDia	cdRNA05-postDia	1.00289	22.4791	4.48634	5.00E-05	0.0289221
WGS	ABySS	XLOC_048336	2272251:11406-16247	cdRNA03-Dia-R2	cdRNA05-postDia	26.3802	2.14628	-3.61955	5.00E-05	0.0289221
WGS	ABySS	XLOC_048336	2272251:11406-16247	cdRNA04-Dia-R3	cdRNA05-postDia	24.6162	2.14628	-3.51969	5.00E-05	0.0289221
WGS	ABySS	XLOC_048363	2272271:45629-51503	cdRNA01-preDia	cdRNA05-postDia	3.73639	27.0925	2.85818	5.00E-05	0.0289221
WGS	ABySS	XLOC_048370	2272275:11797-13281	cdRNA03-Dia-R2	cdRNA05-postDia	5.00317	35.021	2.80731	5.00E-05	0.0289221
WGS	ABySS	XLOC_048370	2272275:11797-13281	cdRNA04-Dia-R3	cdRNA05-postDia	4.9601	35.021	2.81978	5.00E-05	0.0289221
WGS	ABySS	XLOC_048860	2272537:0-972	cdRNA04-Dia-R3	cdRNA05-postDia	6.49588	34.9304	2.42689	5.00E-05	0.0289221
WGS	ABySS	XLOC_048860	2272537:0-972	cdRNA03-Dia-R2	cdRNA05-postDia	6.43154	34.9304	2.44125	0.0001	0.0499641
WGS	ABySS	XLOC_048860	2272537:0-972	cdRNA02-Dia-R1	cdRNA05-postDia	6.30211	34.9304	2.47057	5.00E-05	0.0289221
WGS	ABySS	XLOC_048892	2272557:3132-6430	cdRNA03-Dia-R2	cdRNA05-postDia	213.436	23.9877	-3.15344	0.0001	0.0499641
WGS	ABySS	XLOC_048892	2272557:3132-6430	cdRNA04-Dia-R3	cdRNA05-postDia	199.269	23.9877	-3.05435	5.00E-05	0.0289221
WGS	ABySS	XLOC_048892	2272557:3132-6430	cdRNA01-preDia	cdRNA03-Dia-R2	30.5027	213.436	2.80679	5.00E-05	0.0289221
WGS	ABySS	XLOC_049446	2272835:36083-38029	cdRNA02-Dia-R1	cdRNA05-postDia	15.4525	85.3866	2.46617	0.0001	0.0499641
WGS	ABySS	XLOC_049447	2272835:38164-47711	cdRNA02-Dia-R1	cdRNA05-postDia	11.2007	73.3551	2.71131	5.00E-05	0.0289221
WGS	ABySS	XLOC_049746	2272989:1179-3895	cdRNA02-Dia-R1	cdRNA05-postDia	1.20415	16.2384	3.75333	5.00E-05	0.0289221
WGS	ABySS	XLOC_049746	2272989:1179-3895	cdRNA01-preDia	cdRNA05-postDia	0.541345	16.2384	4.90672	5.00E-05	0.0289221
WGS	ABySS	XLOC_049747	2272989:0-1129	cdRNA01-preDia	cdRNA05-postDia	1.20657	21.5148	4.15635	0.0001	0.0499641
WGS	ABySS	XLOC_050608	2273494:22545-31875	cdRNA01-preDia	cdRNA05-postDia	35.4606	409.949	3.53115	5.00E-05	0.0289221
WGS	ABySS	XLOC_050608	2273494:22545-31875	cdRNA04-Dia-R3	cdRNA05-postDia	15.7422	409.949	4.70273	5.00E-05	0.0289221
WGS	ABySS	XLOC_050608	2273494:22545-31875	cdRNA03-Dia-R2	cdRNA05-postDia	15.4405	409.949	4.73065	5.00E-05	0.0289221
WGS	ABySS	XLOC_050794	2273600:88092-100703	cdRNA01-preDia	cdRNA04-Dia-R3	9.30233	45.0339	2.27535	0.0001	0.0499641
WGS	ABySS	XLOC_050794	2273600:88092-100703	cdRNA01-preDia	cdRNA03-Dia-R2	9.30233	47.2118	2.34348	5.00E-05	0.0289221
WGS	ABySS	XLOC_050849	2273630:11477-16485	cdRNA03-Dia-R2	cdRNA05-postDia	57.8181	9.15053	-2.65959	5.00E-05	0.0289221
WGS	ABySS	XLOC_050849	2273630:11477-16485	cdRNA04-Dia-R3	cdRNA05-postDia	55.0261	9.15053	-2.58819	5.00E-05	0.0289221
WGS	ABySS	XLOC_050849	2273630:11477-16485	cdRNA01-preDia	cdRNA03-Dia-R2	11.3618	57.8181	2.34733	5.00E-05	0.0289221
WGS	ABySS	XLOC_050994	2273709:968-2170	cdRNA02-Dia-R1	cdRNA05-postDia	31.6194	3281.8	6.69754	5.00E-05	0.0289221
WGS	ABySS	XLOC_050994	2273709:968-2170	cdRNA03-Dia-R2	cdRNA05-postDia	21.7395	3281.8	7.23802	5.00E-05	0.0289221
WGS	ABySS	XLOC_050994	2273709:968-2170	cdRNA01-preDia	cdRNA05-postDia	19.7597	3281.8	7.37578	5.00E-05	0.0289221
WGS	ABySS	XLOC_050994	2273709:968-2170	cdRNA04-Dia-R3	cdRNA05-postDia	17.8466	3281.8	7.5227	5.00E-05	0.0289221
WGS	ABySS	XLOC_051066	2273766:30737-36268	cdRNA03-Dia-R2	cdRNA05-postDia	2.65271	14.9012	2.48989	5.00E-05	0.0289221
WGS	ABySS	XLOC_051066	2273766:30737-36268	cdRNA04-Dia-R3	cdRNA05-postDia	2.56001	14.9012	2.5412	5.00E-05	0.0289221
WGS	ABySS	XLOC_051122	2273786:15073-26147	cdRNA04-Dia-R3	cdRNA05-postDia	15.821	2.68085	-2.56108	0.0001	0.0499641
WGS	ABySS	XLOC_051122	2273786:15073-26147	cdRNA03-Dia-R2	cdRNA05-postDia	15.5573	2.68085	-2.53683	0.0001	0.0499641
WGS	ABySS	XLOC_051196	2273829:2451-3322	cdRNA03-Dia-R2	cdRNA05-postDia	419.277	32.6538	-3.68258	0.0001	0.0499641

WGS	ABySS	XLOC_051196	2273829:2451-3322	cdRNA04-Dia-R3	cdRNA05-postDia	409.118	32.6538	-3.64719	0.0001	0.0499641
WGS	ABySS	XLOC_051397	2273914:23645-28792	cdRNA01-preDia	cdRNA04-Dia-R3	14.4364	2.14073	-2.75354	5.00E-05	0.0289221
WGS	ABySS	XLOC_051397	2273914:23645-28792	cdRNA01-preDia	cdRNA03-Dia-R2	14.4364	2.69089	-2.42356	0.0001	0.0499641
WGS	ABySS	XLOC_051397	2273914:23645-28792	cdRNA03-Dia-R2	cdRNA05-postDia	2.69089	12.3414	2.19736	0.0001	0.0499641
WGS	ABySS	XLOC_051397	2273914:23645-28792	cdRNA04-Dia-R3	cdRNA05-postDia	2.14073	12.3414	2.52734	0.0001	0.0499641
WGS	ABySS	XLOC_051484	2273957:25436-28738	cdRNA01-preDia	cdRNA05-postDia	5.38922	32.5187	2.59312	5.00E-05	0.0289221
WGS	ABySS	XLOC_051775	2274152:10552-27125	cdRNA01-preDia	cdRNA05-postDia	9.69181	50.8451	2.39127	0.0001	0.0499641
WGS	ABySS	XLOC_052116	2274336:3219-12828	cdRNA04-Dia-R3	cdRNA05-postDia	13.4077	101.891	2.92589	5.00E-05	0.0289221
WGS	ABySS	XLOC_052116	2274336:3219-12828	cdRNA03-Dia-R2	cdRNA05-postDia	13.1571	101.891	2.95311	5.00E-05	0.0289221
WGS	ABySS	XLOC_052116	2274336:3219-12828	cdRNA02-Dia-R1	cdRNA05-postDia	8.80506	101.891	3.53255	5.00E-05	0.0289221
WGS	ABySS	XLOC_052116	2274336:3219-12828	cdRNA01-preDia	cdRNA05-postDia	1.98636	101.891	5.68076	5.00E-05	0.0289221
WGS	ABySS	XLOC_052146	2274354:8287-23726	cdRNA01-preDia	cdRNA05-postDia	28.3317	167.143	2.56059	5.00E-05	0.0289221
WGS	ABySS	XLOC_052146	2274354:8287-23726	cdRNA02-Dia-R1	cdRNA05-postDia	25.3384	167.143	2.72168	5.00E-05	0.0289221
WGS	ABySS	XLOC_052146	2274354:8287-23726	cdRNA04-Dia-R3	cdRNA05-postDia	19.5184	167.143	3.09817	5.00E-05	0.0289221
WGS	ABySS	XLOC_052146	2274354:8287-23726	cdRNA03-Dia-R2	cdRNA05-postDia	18.9875	167.143	3.13796	5.00E-05	0.0289221
WGS	ABySS	XLOC_052179	2274375:6732-13111	cdRNA03-Dia-R2	cdRNA05-postDia	3.01085	23.1504	2.9428	5.00E-05	0.0289221
WGS	ABySS	XLOC_052179	2274375:6732-13111	cdRNA04-Dia-R3	cdRNA05-postDia	2.82536	23.1504	3.03453	5.00E-05	0.0289221
WGS	ABySS	XLOC_052308	2274440:2829-5818	cdRNA03-Dia-R2	cdRNA05-postDia	123.119	21.6492	-2.50767	5.00E-05	0.0289221
WGS	ABySS	XLOC_052308	2274440:2829-5818	cdRNA04-Dia-R3	cdRNA05-postDia	121.534	21.6492	-2.48897	5.00E-05	0.0289221
WGS	ABySS	XLOC_052417	2274508:6504-10810	cdRNA01-preDia	cdRNA05-postDia	0.568976	6.48176	3.50994	0.0001	0.0499641
WGS	ABySS	XLOC_052418	2274508:10926-15098	cdRNA01-preDia	cdRNA05-postDia	0.888714	9.57	3.42873	5.00E-05	0.0289221
WGS	ABySS	XLOC_052452	2274523:83538-86346	cdRNA01-preDia	cdRNA03-Dia-R2	35.2457	5.07874	-2.79491	5.00E-05	0.0289221
WGS	ABySS	XLOC_052452	2274523:83538-86346	cdRNA01-preDia	cdRNA04-Dia-R3	35.2457	5.48136	-2.68484	5.00E-05	0.0289221
WGS	ABySS	XLOC_052651	2274599:13671-15403	cdRNA01-preDia	cdRNA03-Dia-R2	29.231	164.575	2.49318	5.00E-05	0.0289221
WGS	ABySS	XLOC_052912	2274759:14433-15149	cdRNA01-preDia	cdRNA05-postDia	36.9064	2.08094	-4.14856	0.0001	0.0499641
WGS	ABySS	XLOC_052912	2274759:14433-15149	cdRNA01-preDia	cdRNA03-Dia-R2	36.9064	4.14529	-3.15433	5.00E-05	0.0289221
WGS	ABySS	XLOC_052912	2274759:14433-15149	cdRNA01-preDia	cdRNA04-Dia-R3	36.9064	4.25837	-3.1155	5.00E-05	0.0289221
WGS	ABySS	XLOC_052955	2274773:54518-58850	cdRNA04-Dia-R3	cdRNA05-postDia	19.4766	90.6589	2.21871	5.00E-05	0.0289221
WGS	ABySS	XLOC_052955	2274773:54518-58850	cdRNA03-Dia-R2	cdRNA05-postDia	16.6948	90.6589	2.44105	5.00E-05	0.0289221
WGS	ABySS	XLOC_052956	2274773:58955-63763	cdRNA03-Dia-R2	cdRNA05-postDia	20.5343	100.083	2.28509	0.0001	0.0499641
WGS	ABySS	XLOC_053149	2274860:0-3517	cdRNA03-Dia-R2	cdRNA05-postDia	38.8893	5.05996	-2.94218	5.00E-05	0.0289221
WGS	ABySS	XLOC_053149	2274860:0-3517	cdRNA04-Dia-R3	cdRNA05-postDia	36.7101	5.05996	-2.85898	5.00E-05	0.0289221
WGS	ABySS	XLOC_053262	2274930:4565-11022	cdRNA03-Dia-R2	cdRNA05-postDia	6.54897	44.2075	2.75495	5.00E-05	0.0289221
WGS	ABySS	XLOC_053262	2274930:4565-11022	cdRNA04-Dia-R3	cdRNA05-postDia	6.21294	44.2075	2.83094	5.00E-05	0.0289221
WGS	ABySS	XLOC_053262	2274930:4565-11022	cdRNA01-preDia	cdRNA05-postDia	4.97253	44.2075	3.15224	5.00E-05	0.0289221
WGS	ABySS	XLOC_053536	2275058:86867-89309	cdRNA01-preDia	cdRNA02-Dia-R1	27.1304	236.681	3.12496	5.00E-05	0.0289221
WGS	ABySS	XLOC_053536	2275058:86867-89309	cdRNA01-preDia	cdRNA05-postDia	27.1304	446.796	4.04164	5.00E-05	0.0289221
WGS	ABySS	XLOC_053648	2275116:27395-31549	cdRNA03-Dia-R2	cdRNA05-postDia	186.658	13.4036	-3.7997	5.00E-05	0.0289221
WGS	ABySS	XLOC_053648	2275116:27395-31549	cdRNA04-Dia-R3	cdRNA05-postDia	178.838	13.4036	-3.73796	5.00E-05	0.0289221
WGS	ABySS	XLOC_053648	2275116:27395-31549	cdRNA02-Dia-R1	cdRNA05-postDia	68.2428	13.4036	-2.34806	5.00E-05	0.0289221
WGS	ABySS	XLOC_053648	2275116:27395-31549	cdRNA01-preDia	cdRNA04-Dia-R3	33.2183	178.838	2.4286	0.0001	0.0499641
WGS	ABySS	XLOC_053676	2275136:4341-8167	cdRNA03-Dia-R2	cdRNA05-postDia	227.559	42.0383	-2.43647	0.0001	0.0499641
WGS	ABySS	XLOC_053676	2275136:4341-8167	cdRNA04-Dia-R3	cdRNA05-postDia	214.09	42.0383	-2.34844	0.0001	0.0499641
WGS	ABySS	XLOC_053910	2275255:1437-15794	cdRNA01-preDia	cdRNA05-postDia	44.9892	9.36134	-2.26479	5.00E-05	0.0289221
WGS	ABySS	XLOC_053910	2275255:1437-15794	cdRNA04-Dia-R3	cdRNA05-postDia	37.5611	9.36134	-2.00445	5.00E-05	0.0289221
WGS	ABySS	XLOC_053957	2275291:1329-11504	cdRNA03-Dia-R2	cdRNA05-postDia	87.9024	14.0722	-2.64305	5.00E-05	0.0289221
WGS	ABySS	XLOC_053957	2275291:1329-11504	cdRNA04-Dia-R3	cdRNA05-postDia	80.0185	14.0722	-2.50749	5.00E-05	0.0289221

WGS	ABySS	XLOC_054021	2275322:636-9394	cdRNA03-Dia-R2	cdRNA05-postDia	71.5262	8.96252	-2.99649	5.00E-05	0.0289221
WGS	ABySS	XLOC_054021	2275322:636-9394	cdRNA04-Dia-R3	cdRNA05-postDia	71.4763	8.96252	-2.99549	5.00E-05	0.0289221
WGS	ABySS	XLOC_054021	2275322:636-9394	cdRNA02-Dia-R1	cdRNA04-Dia-R3	11.0229	71.4763	2.69696	5.00E-05	0.0289221
WGS	ABySS	XLOC_054021	2275322:636-9394	cdRNA02-Dia-R1	cdRNA03-Dia-R2	11.0229	71.5262	2.69796	5.00E-05	0.0289221
WGS	ABySS	XLOC_054021	2275322:636-9394	cdRNA01-preDia	cdRNA04-Dia-R3	4.96999	71.4763	3.84615	5.00E-05	0.0289221
WGS	ABySS	XLOC_054021	2275322:636-9394	cdRNA01-preDia	cdRNA03-Dia-R2	4.96999	71.5262	3.84716	5.00E-05	0.0289221
WGS	ABySS	XLOC_054708	2275676:15057-19877	cdRNA03-Dia-R2	cdRNA05-postDia	0.99633	5.91638	2.57002	0.0001	0.0499641
WGS	ABySS	XLOC_054789	2275709:44884-60675	cdRNA03-Dia-R2	cdRNA05-postDia	23.0386	2.65494	-3.1173	5.00E-05	0.0289221
WGS	ABySS	XLOC_054789	2275709:44884-60675	cdRNA04-Dia-R3	cdRNA05-postDia	21.8626	2.65494	-3.04171	5.00E-05	0.0289221
WGS	ABySS	XLOC_054818	2275729:2-10707	cdRNA01-preDia	cdRNA05-postDia	3.41684	33.476	3.29239	5.00E-05	0.0289221
WGS	ABySS	XLOC_054973	2275804:8520-10791	cdRNA01-preDia	cdRNA05-postDia	4.83278	25.1554	2.37994	5.00E-05	0.0289221
WGS	ABySS	XLOC_054973	2275804:8520-10791	cdRNA02-Dia-R1	cdRNA05-postDia	3.29148	25.1554	2.93406	5.00E-05	0.0289221
WGS	ABySS	XLOC_055411	2275979:8678-9589	cdRNA01-preDia	cdRNA04-Dia-R3	168.529	29.5843	-2.51009	0.0001	0.0499641
WGS	ABySS	XLOC_055411	2275979:8678-9589	cdRNA01-preDia	cdRNA03-Dia-R2	168.529	30.7294	-2.45531	5.00E-05	0.0289221
WGS	ABySS	XLOC_055456	2275994:24489-35334	cdRNA03-Dia-R2	cdRNA05-postDia	1.36658	9.91203	2.85861	0.0001	0.0499641
WGS	ABySS	XLOC_055456	2275994:24489-35334	cdRNA04-Dia-R3	cdRNA05-postDia	0.788553	9.91203	3.6519	5.00E-05	0.0289221
WGS	ABySS	XLOC_055699	2276108:38691-44752	cdRNA02-Dia-R1	cdRNA05-postDia	7.00282	34.745	2.3108	5.00E-05	0.0289221
WGS	ABySS	XLOC_055840	2276168:2954-4209	cdRNA01-preDia	cdRNA05-postDia	4.06774	51.4418	3.66064	5.00E-05	0.0289221
WGS	ABySS	XLOC_055841	2276170:1375-2186	cdRNA02-Dia-R1	cdRNA05-postDia	46.0381	334.782	2.86232	5.00E-05	0.0289221
WGS	ABySS	XLOC_056168	2276288:99915-106628	cdRNA01-preDia	cdRNA05-postDia	3.07987	16.0153	2.37851	5.00E-05	0.0289221
WGS	ABySS	XLOC_056168	2276288:99915-106628	cdRNA02-Dia-R1	cdRNA05-postDia	3.06056	16.0153	2.38759	5.00E-05	0.0289221
WGS	ABySS	XLOC_056282	2276349:6961-12381	cdRNA02-Dia-R1	cdRNA05-postDia	3.10425	16.7093	2.42833	5.00E-05	0.0289221
WGS	ABySS	XLOC_056315	2276366:54556-55987	cdRNA01-preDia	cdRNA05-postDia	1.63101	11.6606	2.83781	5.00E-05	0.0289221
WGS	ABySS	XLOC_056409	2276402:10778-15725	cdRNA01-preDia	cdRNA04-Dia-R3	147.67	11.8404	-3.64058	5.00E-05	0.0289221
WGS	ABySS	XLOC_056409	2276402:10778-15725	cdRNA01-preDia	cdRNA03-Dia-R2	147.67	12.1689	-3.6011	5.00E-05	0.0289221
WGS	ABySS	XLOC_056522	2276454:110463-115052	cdRNA03-Dia-R2	cdRNA05-postDia	13.8998	2.46512	-2.49533	0.0001	0.0499641
WGS	ABySS	XLOC_056522	2276454:110463-115052	cdRNA04-Dia-R3	cdRNA05-postDia	13.711	2.46512	-2.47561	0.0001	0.0499641
WGS	ABySS	XLOC_056583	2276486:64900-72474	cdRNA01-preDia	cdRNA02-Dia-R1	4.77073	185.395	5.28025	5.00E-05	0.0289221
WGS	ABySS	XLOC_056583	2276486:64900-72474	cdRNA01-preDia	cdRNA03-Dia-R2	4.77073	242.557	5.66797	5.00E-05	0.0289221
WGS	ABySS	XLOC_056583	2276486:64900-72474	cdRNA01-preDia	cdRNA04-Dia-R3	4.77073	253.376	5.73092	5.00E-05	0.0289221
WGS	ABySS	XLOC_056585	2276486:93148-112014	cdRNA01-preDia	cdRNA02-Dia-R1	4.96081	35.6985	2.84722	5.00E-05	0.0289221
WGS	ABySS	XLOC_056610	2276498:21380-24224	cdRNA02-Dia-R1	cdRNA05-postDia	2.98343	20.2432	2.76239	5.00E-05	0.0289221
WGS	ABySS	XLOC_056679	2276548:888-5963	cdRNA01-preDia	cdRNA04-Dia-R3	51.2101	8.63788	-2.56768	5.00E-05	0.0289221
WGS	ABySS	XLOC_056679	2276548:888-5963	cdRNA01-preDia	cdRNA03-Dia-R2	51.2101	8.99019	-2.51	5.00E-05	0.0289221
WGS	ABySS	XLOC_056679	2276548:888-5963	cdRNA03-Dia-R2	cdRNA05-postDia	8.99019	48.4374	2.4297	5.00E-05	0.0289221
WGS	ABySS	XLOC_056679	2276548:888-5963	cdRNA04-Dia-R3	cdRNA05-postDia	8.63788	48.4374	2.48737	5.00E-05	0.0289221
WGS	ABySS	XLOC_057122	2276742:0-3994	cdRNA01-preDia	cdRNA05-postDia	0.758498	5.67825	2.90423	0.0001	0.0499641
WGS	ABySS	XLOC_057196	2276772:10138-12757	cdRNA01-preDia	cdRNA03-Dia-R2	4.90057	24.3089	2.31046	0.0001	0.0499641
WGS	ABySS	XLOC_057279	2276810:67717-77843	cdRNA01-preDia	cdRNA05-postDia	62.0056	10.9096	-2.5068	5.00E-05	0.0289221
WGS	ABySS	XLOC_057279	2276810:67717-77843	cdRNA04-Dia-R3	cdRNA05-postDia	61.3553	10.9096	-2.49159	5.00E-05	0.0289221
WGS	ABySS	XLOC_057279	2276810:67717-77843	cdRNA03-Dia-R2	cdRNA05-postDia	59.7818	10.9096	-2.45411	5.00E-05	0.0289221
WGS	ABySS	XLOC_057535	2276942:17381-44158	cdRNA04-Dia-R3	cdRNA05-postDia	4.5367	26.8207	2.56363	0.0001	0.0499641
WGS	ABySS	XLOC_057567	2276967:15988-18489	cdRNA01-preDia	cdRNA05-postDia	1.96352	15.0438	2.93766	5.00E-05	0.0289221
WGS	ABySS	XLOC_057659	2277045:421-1116	cdRNA02-Dia-R1	cdRNA05-postDia	4.91915	81.2863	4.04653	5.00E-05	0.0289221
WGS	ABySS	XLOC_057659	2277045:421-1116	cdRNA03-Dia-R2	cdRNA05-postDia	4.43002	81.2863	4.19763	5.00E-05	0.0289221
WGS	ABySS	XLOC_057659	2277045:421-1116	cdRNA04-Dia-R3	cdRNA05-postDia	4.14282	81.2863	4.29433	5.00E-05	0.0289221
WGS	ABySS	XLOC_057659	2277045:421-1116	cdRNA01-preDia	cdRNA05-postDia	4.12707	81.2863	4.29982	5.00E-05	0.0289221

WGS	ABySS	XLOC_057752	2277084:69252-71498	cdRNA02-Dia-R1	cdRNA05-postDia	11.6457	117.366	3.33314	5.00E-05	0.0289221
WGS	ABySS	XLOC_057752	2277084:69252-71498	cdRNA04-Dia-R3	cdRNA05-postDia	7.39651	117.366	3.98802	5.00E-05	0.0289221
WGS	ABySS	XLOC_057752	2277084:69252-71498	cdRNA03-Dia-R2	cdRNA05-postDia	6.69312	117.366	4.13219	5.00E-05	0.0289221
WGS	ABySS	XLOC_057752	2277084:69252-71498	cdRNA01-preDia	cdRNA05-postDia	3.88696	117.366	4.91623	5.00E-05	0.0289221
WGS	ABySS	XLOC_057800	2277113:895-19177	cdRNA04-Dia-R3	cdRNA05-postDia	17.4237	2.87951	-2.59716	5.00E-05	0.0289221
WGS	ABySS	XLOC_057800	2277113:895-19177	cdRNA03-Dia-R2	cdRNA05-postDia	16.1938	2.87951	-2.49155	5.00E-05	0.0289221
WGS	ABySS	XLOC_057800	2277113:895-19177	cdRNA01-preDia	cdRNA03-Dia-R2	2.03854	16.1938	2.98983	5.00E-05	0.0289221
WGS	ABySS	XLOC_057800	2277113:895-19177	cdRNA01-preDia	cdRNA04-Dia-R3	2.03854	17.4237	3.09545	5.00E-05	0.0289221
WGS	ABySS	XLOC_058062	2277244:3-6270	cdRNA03-Dia-R2	cdRNA05-postDia	32.3344	6.79301	-2.25095	0.0001	0.0499641
WGS	ABySS	XLOC_058118	2277263:2165-3768	cdRNA02-Dia-R1	cdRNA05-postDia	4.23997	24.4798	2.52946	5.00E-05	0.0289221
WGS	ABySS	XLOC_058118	2277263:2165-3768	cdRNA01-preDia	cdRNA05-postDia	0.969687	24.4798	4.65793	5.00E-05	0.0289221
WGS	ABySS	XLOC_058124	2277273:0-1625	cdRNA02-Dia-R1	cdRNA05-postDia	1.52482	13.7105	3.16857	5.00E-05	0.0289221
WGS	ABySS	XLOC_058124	2277273:0-1625	cdRNA04-Dia-R3	cdRNA05-postDia	1.01424	13.7105	3.75682	5.00E-05	0.0289221
WGS	ABySS	XLOC_058124	2277273:0-1625	cdRNA03-Dia-R2	cdRNA05-postDia	0.869011	13.7105	3.97977	5.00E-05	0.0289221
WGS	ABySS	XLOC_058665	2277504:502-1702	cdRNA01-preDia	cdRNA02-Dia-R1	87.8898	3.86292	-4.50793	5.00E-05	0.0289221
WGS	ABySS	XLOC_058665	2277504:502-1702	cdRNA01-preDia	cdRNA03-Dia-R2	87.8898	11.5147	-2.93222	5.00E-05	0.0289221
WGS	ABySS	XLOC_058665	2277504:502-1702	cdRNA01-preDia	cdRNA04-Dia-R3	87.8898	11.7137	-2.9075	5.00E-05	0.0289221
WGS	ABySS	XLOC_058795	2277566:46664-49108	cdRNA01-preDia	cdRNA05-postDia	63.0458	329.884	2.38749	5.00E-05	0.0289221
WGS	ABySS	XLOC_058827	2277578:1324-7471	cdRNA04-Dia-R3	cdRNA05-postDia	40.9406	6.15685	-2.73327	5.00E-05	0.0289221
WGS	ABySS	XLOC_058827	2277578:1324-7471	cdRNA03-Dia-R2	cdRNA05-postDia	38.683	6.15685	-2.65143	5.00E-05	0.0289221
WGS	ABySS	XLOC_058827	2277578:1324-7471	cdRNA01-preDia	cdRNA03-Dia-R2	7.10613	38.683	2.44456	5.00E-05	0.0289221
WGS	ABySS	XLOC_058827	2277578:1324-7471	cdRNA01-preDia	cdRNA04-Dia-R3	7.10613	40.9406	2.5264	5.00E-05	0.0289221
WGS	ABySS	XLOC_058898	2277622:51556-59481	cdRNA04-Dia-R3	cdRNA05-postDia	32.7145	2.72395	-3.58616	5.00E-05	0.0289221
WGS	ABySS	XLOC_058898	2277622:51556-59481	cdRNA03-Dia-R2	cdRNA05-postDia	31.7437	2.72395	-3.5427	5.00E-05	0.0289221
WGS	ABySS	XLOC_058898	2277622:51556-59481	cdRNA01-preDia	cdRNA03-Dia-R2	3.13859	31.7437	3.33828	5.00E-05	0.0289221
WGS	ABySS	XLOC_058898	2277622:51556-59481	cdRNA01-preDia	cdRNA04-Dia-R3	3.13859	32.7145	3.38174	5.00E-05	0.0289221
WGS	ABySS	XLOC_058902	2277622:59577-62459	cdRNA04-Dia-R3	cdRNA05-postDia	14.6964	2.60029	-2.49872	5.00E-05	0.0289221
WGS	ABySS	XLOC_058902	2277622:59577-62459	cdRNA03-Dia-R2	cdRNA05-postDia	13.8047	2.60029	-2.40841	0.0001	0.0499641
WGS	ABySS	XLOC_058902	2277622:59577-62459	cdRNA01-preDia	cdRNA03-Dia-R2	1.85387	13.8047	2.89655	5.00E-05	0.0289221
WGS	ABySS	XLOC_058902	2277622:59577-62459	cdRNA01-preDia	cdRNA04-Dia-R3	1.85387	14.6964	2.98685	5.00E-05	0.0289221
WGS	ABySS	XLOC_059224	2277846:10438-57783	cdRNA01-preDia	cdRNA03-Dia-R2	19.5901	4.52434	-2.11434	0.0001	0.0499641
WGS	ABySS	XLOC_059900	2278205:46987-49131	cdRNA01-preDia	cdRNA04-Dia-R3	54.683	3.59797	-3.92583	5.00E-05	0.0289221
WGS	ABySS	XLOC_059900	2278205:46987-49131	cdRNA01-preDia	cdRNA03-Dia-R2	54.683	4.04484	-3.75694	5.00E-05	0.0289221
WGS	ABySS	XLOC_059905	2278206:29923-33258	cdRNA03-Dia-R2	cdRNA05-postDia	1.89556	15.5372	3.03503	5.00E-05	0.0289221
WGS	ABySS	XLOC_059905	2278206:29923-33258	cdRNA04-Dia-R3	cdRNA05-postDia	1.51729	15.5372	3.35616	5.00E-05	0.0289221
WGS	ABySS	XLOC_059905	2278206:29923-33258	cdRNA02-Dia-R1	cdRNA05-postDia	1.29666	15.5372	3.58285	5.00E-05	0.0289221
WGS	ABySS	XLOC_059975	2278234:4785-9525	cdRNA01-preDia	cdRNA05-postDia	16.7331	3.07937	-2.442	5.00E-05	0.0289221
WGS	ABySS	XLOC_060086	2278311:133644-138731	cdRNA01-preDia	cdRNA05-postDia	6.41046	52.6995	3.03929	5.00E-05	0.0289221
WGS	ABySS	XLOC_060086	2278311:133644-138731	cdRNA03-Dia-R2	cdRNA05-postDia	5.63468	52.6995	3.22538	5.00E-05	0.0289221
WGS	ABySS	XLOC_060086	2278311:133644-138731	cdRNA04-Dia-R3	cdRNA05-postDia	5.09234	52.6995	3.37139	5.00E-05	0.0289221
WGS	ABySS	XLOC_060086	2278311:133644-138731	cdRNA02-Dia-R1	cdRNA05-postDia	3.70109	52.6995	3.83177	5.00E-05	0.0289221
WGS	ABySS	XLOC_060262	2278401:988-3433	cdRNA01-preDia	cdRNA05-postDia	2.95302	22.476	2.92812	5.00E-05	0.0289221
WGS	ABySS	XLOC_060371	2278454:916-10707	cdRNA01-preDia	cdRNA05-postDia	8.72011	43.1093	2.30558	5.00E-05	0.0289221
WGS	ABySS	XLOC_060371	2278454:916-10707	cdRNA02-Dia-R1	cdRNA05-postDia	6.52379	43.1093	2.72421	5.00E-05	0.0289221
WGS	ABySS	XLOC_060476	2278497:836-2878	cdRNA04-Dia-R3	cdRNA05-postDia	11.7218	1.59413	-2.87834	5.00E-05	0.0289221
WGS	ABySS	XLOC_060476	2278497:836-2878	cdRNA03-Dia-R2	cdRNA05-postDia	10.4137	1.59413	-2.70764	5.00E-05	0.0289221
WGS	ABySS	XLOC_060534	2278532:0-2353	cdRNA01-preDia	cdRNA05-postDia	2.14841	14.9344	2.7973	5.00E-05	0.0289221

WGS	ABySS	XLOC_060581	2278551:15325-18302	cdRNA04-Dia-R3	cdRNA05-postDia	15.3448	102.889	2.74526	5.00E-05	0.0289221
WGS	ABySS	XLOC_060581	2278551:15325-18302	cdRNA03-Dia-R2	cdRNA05-postDia	12.9896	102.889	2.98566	5.00E-05	0.0289221
WGS	ABySS	XLOC_060599	2278557:32274-37494	cdRNA02-Dia-R1	cdRNA05-postDia	31.7156	219.633	2.79183	5.00E-05	0.0289221
WGS	ABySS	XLOC_060657	2278578:681-2273	cdRNA04-Dia-R3	cdRNA05-postDia	2.34294	13.1044	2.48366	0.0001	0.0499641
WGS	ABySS	XLOC_060736	2278617:9381-16186	cdRNA01-preDia	cdRNA05-postDia	18.5866	3.26989	-2.50695	5.00E-05	0.0289221
WGS	ABySS	XLOC_060866	2278673:6850-12243	cdRNA01-preDia	cdRNA04-Dia-R3	9.76245	0.730608	-3.74007	5.00E-05	0.0289221
WGS	ABySS	XLOC_060866	2278673:6850-12243	cdRNA01-preDia	cdRNA03-Dia-R2	9.76245	0.882727	-3.4672	5.00E-05	0.0289221
WGS	ABySS	XLOC_060866	2278673:6850-12243	cdRNA03-Dia-R2	cdRNA05-postDia	0.882727	6.90682	2.96798	0.0001	0.0499641
WGS	ABySS	XLOC_060866	2278673:6850-12243	cdRNA04-Dia-R3	cdRNA05-postDia	0.730608	6.90682	3.24085	5.00E-05	0.0289221
WGS	ABySS	XLOC_060894	2278686:3286-7397	cdRNA04-Dia-R3	cdRNA05-postDia	117.879	10.1103	-3.54341	5.00E-05	0.0289221
WGS	ABySS	XLOC_060894	2278686:3286-7397	cdRNA03-Dia-R2	cdRNA05-postDia	113.626	10.1103	-3.4904	5.00E-05	0.0289221
WGS	ABySS	XLOC_060894	2278686:3286-7397	cdRNA01-preDia	cdRNA03-Dia-R2	18.6569	113.626	2.60652	5.00E-05	0.0289221
WGS	ABySS	XLOC_060894	2278686:3286-7397	cdRNA01-preDia	cdRNA04-Dia-R3	18.6569	117.879	2.65952	5.00E-05	0.0289221
WGS	ABySS	XLOC_060950	2278704:4526-5646	cdRNA01-preDia	cdRNA05-postDia	1.81456	51.2595	4.82013	5.00E-05	0.0289221
WGS	ABySS	XLOC_060950	2278704:4526-5646	cdRNA03-Dia-R2	cdRNA05-postDia	1.68674	51.2595	4.92551	0.0001	0.0499641
WGS	ABySS	XLOC_060986	2278721:3944-5085	cdRNA04-Dia-R3	cdRNA05-postDia	58.5671	12.4268	-2.23664	5.00E-05	0.0289221
WGS	ABySS	XLOC_061166	2278852:10881-12120	cdRNA03-Dia-R2	cdRNA05-postDia	6.11465	46.6183	2.93055	5.00E-05	0.0289221
WGS	ABySS	XLOC_061166	2278852:10881-12120	cdRNA04-Dia-R3	cdRNA05-postDia	5.78418	46.6183	3.01071	5.00E-05	0.0289221
WGS	ABySS	XLOC_061629	2279115:65-5112	cdRNA01-preDia	cdRNA05-postDia	1.14047	11.0137	3.2716	5.00E-05	0.0289221
WGS	ABySS	XLOC_061864	2279205:3852-6664	cdRNA02-Dia-R1	cdRNA05-postDia	5.51927	49.2933	3.15884	5.00E-05	0.0289221
WGS	ABySS	XLOC_061864	2279205:3852-6664	cdRNA03-Dia-R2	cdRNA05-postDia	2.06067	49.2933	4.58021	5.00E-05	0.0289221
WGS	ABySS	XLOC_061864	2279205:3852-6664	cdRNA04-Dia-R3	cdRNA05-postDia	1.86299	49.2933	4.7257	5.00E-05	0.0289221
WGS	ABySS	XLOC_061864	2279205:3852-6664	cdRNA01-preDia	cdRNA05-postDia	1.11906	49.2933	5.46103	5.00E-05	0.0289221
WGS	ABySS	XLOC_062045	2279313:2-9431	cdRNA03-Dia-R2	cdRNA05-postDia	46.7776	9.2655	-2.33588	0.0001	0.0499641
WGS	ABySS	XLOC_062045	2279313:2-9431	cdRNA04-Dia-R3	cdRNA05-postDia	45.1267	9.2655	-2.28404	5.00E-05	0.0289221
WGS	ABySS	XLOC_062169	2279376:17644-18436	cdRNA03-Dia-R2	cdRNA05-postDia	162.732	1024.45	2.65427	5.00E-05	0.0289221
WGS	ABySS	XLOC_062169	2279376:17644-18436	cdRNA04-Dia-R3	cdRNA05-postDia	150.52	1024.45	2.76681	5.00E-05	0.0289221
WGS	ABySS	XLOC_062169	2279376:17644-18436	cdRNA01-preDia	cdRNA05-postDia	147.472	1024.45	2.79632	5.00E-05	0.0289221
WGS	ABySS	XLOC_063107	2279907:102-3192	cdRNA02-Dia-R1	cdRNA05-postDia	20.7765	162.041	2.96334	5.00E-05	0.0289221
WGS	ABySS	XLOC_063107	2279907:102-3192	cdRNA01-preDia	cdRNA05-postDia	9.63683	162.041	4.07166	5.00E-05	0.0289221
WGS	ABySS	XLOC_063107	2279907:102-3192	cdRNA03-Dia-R2	cdRNA05-postDia	7.90236	162.041	4.35794	5.00E-05	0.0289221
WGS	ABySS	XLOC_063107	2279907:102-3192	cdRNA04-Dia-R3	cdRNA05-postDia	7.38108	162.041	4.45639	5.00E-05	0.0289221
WGS	ABySS	XLOC_063550	2280150:17317-20191	cdRNA04-Dia-R3	cdRNA05-postDia	2.57438	14.1663	2.46016	0.0001	0.0499641
WGS	ABySS	XLOC_063550	2280150:17317-20191	cdRNA03-Dia-R2	cdRNA05-postDia	2.28358	14.1663	2.63309	0.0001	0.0499641
WGS	ABySS	XLOC_063550	2280150:17317-20191	cdRNA02-Dia-R1	cdRNA05-postDia	1.85492	14.1663	2.93303	5.00E-05	0.0289221
WGS	ABySS	XLOC_063570	2280154:71922-76273	cdRNA01-preDia	cdRNA03-Dia-R2	86.9056	17.6109	-2.30298	0.0001	0.0499641
WGS	ABySS	XLOC_063682	2280216:250595-255478	cdRNA02-Dia-R1	cdRNA05-postDia	0.48189	50.3025	6.70578	5.00E-05	0.0289221
WGS	ABySS	XLOC_063830	2280316:13961-22067	cdRNA03-Dia-R2	cdRNA05-postDia	21.9956	3.15898	-2.79969	5.00E-05	0.0289221
WGS	ABySS	XLOC_063830	2280316:13961-22067	cdRNA04-Dia-R3	cdRNA05-postDia	20.2385	3.15898	-2.67957	0.0001	0.0499641
WGS	ABySS	XLOC_065131	497136:0-106	cdRNA04-Dia-R3	cdRNA05-postDia	10414.2	58535.5	2.49077	5.00E-05	0.0289221
WGS	ABySS	XLOC_065131	497136:0-106	cdRNA03-Dia-R2	cdRNA05-postDia	9795.58	58535.5	2.57911	5.00E-05	0.0289221
WGS	ABySS	XLOC_065131	497136:0-106	cdRNA02-Dia-R1	cdRNA05-postDia	9492.26	58535.5	2.62449	5.00E-05	0.0289221
WGS	ABySS	XLOC_065131	497136:0-106	cdRNA01-preDia	cdRNA05-postDia	8790.63	58535.5	2.73527	5.00E-05	0.0289221
WGS	ABySS	XLOC_065291	528972:13-798	cdRNA02-Dia-R1	cdRNA05-postDia	194.722	1199.23	2.62263	5.00E-05	0.0289221
WGS	ABySS	XLOC_065291	528972:13-798	cdRNA03-Dia-R2	cdRNA05-postDia	162.881	1199.23	2.88022	5.00E-05	0.0289221
WGS	ABySS	XLOC_065291	528972:13-798	cdRNA04-Dia-R3	cdRNA05-postDia	158.66	1199.23	2.91811	5.00E-05	0.0289221
WGS	ABySS	XLOC_065413	556114:0-95	cdRNA01-preDia	cdRNA05-postDia	12332.8	64400.5	2.38458	0.0001	0.0499641

WGS	ABySS	XLOC_065432	559927:0-118	cdRNA01-preDia	cdRNA05-postDia	403.04	4097.41	3.34572	5.00E-05	0.0289221
WGS	ABySS	XLOC_065634	607895:0-119	cdRNA01-preDia	cdRNA03-Dia-R2	359.23	4512.51	3.65095	5.00E-05	0.0289221
WGS	ABySS	XLOC_065634	607895:0-119	cdRNA01-preDia	cdRNA04-Dia-R3	359.23	5135.77	3.8376	5.00E-05	0.0289221
WGS	ABySS	XLOC_065903	668102:0-121	cdRNA03-Dia-R2	cdRNA05-postDia	607.085	5056.13	3.05806	5.00E-05	0.0289221
WGS	ABySS	XLOC_065903	668102:0-121	cdRNA04-Dia-R3	cdRNA05-postDia	590.309	5056.13	3.09849	5.00E-05	0.0289221
WGS	ABySS	XLOC_066161	730060:0-82	cdRNA01-preDia	cdRNA05-postDia	41581.3	227921	2.45453	5.00E-05	0.0289221
WGS	ABySS	XLOC_066241	750901:0-80	cdRNA01-preDia	cdRNA05-postDia	34603.1	201299	2.54037	0.0001	0.0499641
WGS	ABySS	XLOC_066907	891481:0-89	cdRNA01-preDia	cdRNA05-postDia	2549.47	30393.1	3.57548	5.00E-05	0.0289221
WGS	ABySS	XLOC_067132	942480:205-354	cdRNA03-Dia-R2	cdRNA05-postDia	3886.73	559.005	-2.79762	5.00E-05	0.0289221
WGS	ABySS	XLOC_067132	942480:205-354	cdRNA04-Dia-R3	cdRNA05-postDia	3269.01	559.005	-2.54792	0.0001	0.0499641
RNA-Seq	Trans-ABySS	XLOC_000201	J1748022:2-211	cdRNA02-Dia-R1	cdRNA05-postDia	23.6544	657.062	4.79585	5.00E-05	0.0195319
RNA-Seq	Trans-ABySS	XLOC_000227	J1748303:1-127	cdRNA01-preDia	cdRNA05-postDia	439.668	3019.25	2.7797	0.0002	0.0481888
RNA-Seq	Trans-ABySS	XLOC_000264	J1748770:0-539	cdRNA01-preDia	cdRNA03-Dia-R2	149.488	26.0982	-2.518	5.00E-05	0.0195319
RNA-Seq	Trans-ABySS	XLOC_000264	J1748770:0-539	cdRNA04-Dia-R3	cdRNA05-postDia	33.0046	169.281	2.35868	0.0001	0.0354705
RNA-Seq	Trans-ABySS	XLOC_000264	J1748770:0-539	cdRNA03-Dia-R2	cdRNA05-postDia	26.0982	169.281	2.6974	5.00E-05	0.0195319
RNA-Seq	Trans-ABySS	XLOC_000326	J1749583:0-70	cdRNA02-Dia-R1	cdRNA05-postDia	5678.86	36842.7	2.69771	0.0002	0.0481888
RNA-Seq	Trans-ABySS	XLOC_000326	J1749583:0-70	cdRNA03-Dia-R2	cdRNA05-postDia	4332.32	36842.7	3.08817	0.00015	0.0374403
RNA-Seq	Trans-ABySS	XLOC_000534	J1752569:0-195	cdRNA03-Dia-R2	cdRNA05-postDia	88.7268	501.494	2.49879	0.0002	0.0481888
RNA-Seq	Trans-ABySS	XLOC_000719	J1754802:0-309	cdRNA02-Dia-R1	cdRNA05-postDia	29.2373	151.5	2.37343	0.00015	0.0374403
RNA-Seq	Trans-ABySS	XLOC_000974	J1757944:0-83	cdRNA01-preDia	cdRNA04-Dia-R3	3312.32	19864.9	2.5843	0.0002	0.0481888
RNA-Seq	Trans-ABySS	XLOC_001007	J1758342:0-92	cdRNA02-Dia-R1	cdRNA05-postDia	1571.5	10121.6	2.68722	0.0002	0.0481888
RNA-Seq	Trans-ABySS	XLOC_001165	J1760352:0-713	cdRNA03-Dia-R2	cdRNA05-postDia	7.27387	33.0149	2.18232	0.0002	0.0481888
RNA-Seq	Trans-ABySS	XLOC_001380	J1762506:0-431	cdRNA02-Dia-R1	cdRNA05-postDia	15.9806	99.4173	2.63718	0.0001	0.0354705
RNA-Seq	Trans-ABySS	XLOC_001380	J1762506:0-431	cdRNA03-Dia-R2	cdRNA05-postDia	11.397	99.4173	3.12485	5.00E-05	0.0195319
RNA-Seq	Trans-ABySS	XLOC_001380	J1762506:0-431	cdRNA04-Dia-R3	cdRNA05-postDia	8.27434	99.4173	3.58678	5.00E-05	0.0195319
RNA-Seq	Trans-ABySS	XLOC_001380	J1762506:0-431	cdRNA01-preDia	cdRNA05-postDia	3.61645	99.4173	4.78085	5.00E-05	0.0195319
RNA-Seq	Trans-ABySS	XLOC_001571	J1765090:0-97	cdRNA03-Dia-R2	cdRNA05-postDia	421.535	5307.36	3.65427	0.0002	0.0481888
RNA-Seq	Trans-ABySS	XLOC_001826	J1767702:0-133	cdRNA02-Dia-R1	cdRNA05-postDia	681.728	4579.9	2.74805	5.00E-05	0.0195319
RNA-Seq	Trans-ABySS	XLOC_002382	J1774638:0-122	cdRNA01-preDia	cdRNA05-postDia	8249.13	1514.66	-2.44525	5.00E-05	0.0195319
RNA-Seq	Trans-ABySS	XLOC_002402	J1774869:0-100	cdRNA02-Dia-R1	cdRNA05-postDia	9157.92	729.689	-3.64967	5.00E-05	0.0195319
RNA-Seq	Trans-ABySS	XLOC_002402	J1774869:0-100	cdRNA01-preDia	cdRNA05-postDia	5779.55	729.689	-2.9856	5.00E-05	0.0195319
RNA-Seq	Trans-ABySS	XLOC_002559	J1776690:0-72	cdRNA03-Dia-R2	cdRNA05-postDia	6169.24	36526.4	2.56578	0.00015	0.0374403
RNA-Seq	Trans-ABySS	XLOC_002614	J1777267:0-96	cdRNA01-preDia	cdRNA04-Dia-R3	3284.98	16473.6	2.3262	0.0001	0.0354705
RNA-Seq	Trans-ABySS	XLOC_002689	J1778311:0-289	cdRNA02-Dia-R1	cdRNA05-postDia	31.296	382.126	3.61	5.00E-05	0.0195319
RNA-Seq	Trans-ABySS	XLOC_002689	J1778311:0-289	cdRNA01-preDia	cdRNA05-postDia	16.6942	382.126	4.51663	5.00E-05	0.0195319
RNA-Seq	Trans-ABySS	XLOC_002689	J1778311:0-289	cdRNA04-Dia-R3	cdRNA05-postDia	14.2051	382.126	4.74957	5.00E-05	0.0195319
RNA-Seq	Trans-ABySS	XLOC_002689	J1778311:0-289	cdRNA03-Dia-R2	cdRNA05-postDia	13.3588	382.126	4.83819	5.00E-05	0.0195319
RNA-Seq	Trans-ABySS	XLOC_002834	J1780013:0-82	cdRNA03-Dia-R2	cdRNA05-postDia	62258.7	3558.05	-4.12912	5.00E-05	0.0195319
RNA-Seq	Trans-ABySS	XLOC_002834	J1780013:0-82	cdRNA04-Dia-R3	cdRNA05-postDia	49550.3	3558.05	-3.79973	5.00E-05	0.0195319
RNA-Seq	Trans-ABySS	XLOC_002834	J1780013:0-82	cdRNA02-Dia-R1	cdRNA05-postDia	39902.7	3558.05	-3.48733	5.00E-05	0.0195319
RNA-Seq	Trans-ABySS	XLOC_002834	J1780013:0-82	cdRNA01-preDia	cdRNA05-postDia	35031.2	3558.05	-3.29948	5.00E-05	0.0195319
RNA-Seq	Trans-ABySS	XLOC_002879	J1780551:0-87	cdRNA03-Dia-R2	cdRNA05-postDia	17754.6	2346.8	-2.91943	5.00E-05	0.0195319
RNA-Seq	Trans-ABySS	XLOC_002879	J1780551:0-87	cdRNA04-Dia-R3	cdRNA05-postDia	17353	2346.8	-2.88642	5.00E-05	0.0195319
RNA-Seq	Trans-ABySS	XLOC_002879	J1780551:0-87	cdRNA02-Dia-R1	cdRNA05-postDia	12942.9	2346.8	-2.46339	0.00015	0.0374403
RNA-Seq	Trans-ABySS	XLOC_003074	J1782712:0-167	cdRNA02-Dia-R1	cdRNA05-postDia	147.392	2079.35	3.8184	5.00E-05	0.0195319
RNA-Seq	Trans-ABySS	XLOC_003074	J1782712:0-167	cdRNA01-preDia	cdRNA05-postDia	106.32	2079.35	4.28964	5.00E-05	0.0195319
RNA-Seq	Trans-ABySS	XLOC_003074	J1782712:0-167	cdRNA03-Dia-R2	cdRNA05-postDia	58.1088	2079.35	5.16123	5.00E-05	0.0195319

RNA-Seq	Trans-ABySS	XLOC_003074	J1782712:0-167	cdRNA04-Dia-R3	cdRNA05-postDia	40.9766	2079.35	5.66519	0.0002	0.0481888
RNA-Seq	Trans-ABySS	XLOC_003240	J1784571:2-65	cdRNA03-Dia-R2	cdRNA05-postDia	8952.17	68791.4	2.94192	0.0002	0.0481888
RNA-Seq	Trans-ABySS	XLOC_003313	J1785403:0-116	cdRNA03-Dia-R2	cdRNA05-postDia	6251.85	971.782	-2.68558	5.00E-05	0.0195319
RNA-Seq	Trans-ABySS	XLOC_003313	J1785403:0-116	cdRNA01-preDia	cdRNA05-postDia	5905.65	971.782	-2.60339	5.00E-05	0.0195319
RNA-Seq	Trans-ABySS	XLOC_003313	J1785403:0-116	cdRNA04-Dia-R3	cdRNA05-postDia	5466.07	971.782	-2.4918	5.00E-05	0.0195319
RNA-Seq	Trans-ABySS	XLOC_003313	J1785403:0-116	cdRNA02-Dia-R1	cdRNA05-postDia	5279.29	971.782	-2.44164	0.0001	0.0354705
RNA-Seq	Trans-ABySS	XLOC_003420	J1786645:0-917	cdRNA01-preDia	cdRNA04-Dia-R3	18.0392	2.9937	-2.59113	0.0002	0.0481888
RNA-Seq	Trans-ABySS	XLOC_003557	J1788260:0-112	cdRNA01-preDia	cdRNA05-postDia	6222.3	1111.95	-2.48435	0.00015	0.0374403
RNA-Seq	Trans-ABySS	XLOC_003557	J1788260:0-112	cdRNA03-Dia-R2	cdRNA05-postDia	5861.85	1111.95	-2.39826	0.0002	0.0481888
RNA-Seq	Trans-ABySS	XLOC_003596	J1788722:0-498	cdRNA03-Dia-R2	cdRNA05-postDia	18.055	93.8044	2.37726	0.0002	0.0481888
RNA-Seq	Trans-ABySS	XLOC_003596	J1788722:0-498	cdRNA01-preDia	cdRNA05-postDia	10.2386	93.8044	3.19563	5.00E-05	0.0195319
RNA-Seq	Trans-ABySS	XLOC_003618	J1788994:1-261	cdRNA02-Dia-R1	cdRNA05-postDia	82.56	439.93	2.41376	0.0002	0.0481888
RNA-Seq	Trans-ABySS	XLOC_003754	J1790788:0-288	cdRNA02-Dia-R1	cdRNA05-postDia	34.985	256.227	2.87261	5.00E-05	0.0195319
RNA-Seq	Trans-ABySS	XLOC_003754	J1790788:0-288	cdRNA01-preDia	cdRNA05-postDia	21.9639	256.227	3.54422	5.00E-05	0.0195319
RNA-Seq	Trans-ABySS	XLOC_003936	J1792933:0-74	cdRNA04-Dia-R3	cdRNA05-postDia	14337	71860.2	2.32545	0.0001	0.0354705
RNA-Seq	Trans-ABySS	XLOC_003936	J1792933:0-74	cdRNA02-Dia-R1	cdRNA05-postDia	12306.5	71860.2	2.54577	0.0001	0.0354705
RNA-Seq	Trans-ABySS	XLOC_003936	J1792933:0-74	cdRNA01-preDia	cdRNA05-postDia	3713.34	71860.2	4.2744	5.00E-05	0.0195319
RNA-Seq	Trans-ABySS	XLOC_004008	J1793707:1-86	cdRNA03-Dia-R2	cdRNA05-postDia	1433.13	19468.5	3.7639	5.00E-05	0.0195319
RNA-Seq	Trans-ABySS	XLOC_004008	J1793707:1-86	cdRNA04-Dia-R3	cdRNA05-postDia	1269.94	19468.5	3.93831	5.00E-05	0.0195319
RNA-Seq	Trans-ABySS	XLOC_004019	J1793821:0-100	cdRNA02-Dia-R1	cdRNA05-postDia	6780.91	65453.1	3.27091	5.00E-05	0.0195319
RNA-Seq	Trans-ABySS	XLOC_004102	J1794886:0-562	cdRNA01-preDia	cdRNA05-postDia	105.232	719.576	2.77358	0.00015	0.0374403
RNA-Seq	Trans-ABySS	XLOC_004254	J1796749:0-76	cdRNA03-Dia-R2	cdRNA05-postDia	5338.79	38111.8	2.83565	5.00E-05	0.0195319
RNA-Seq	Trans-ABySS	XLOC_004254	J1796749:0-76	cdRNA04-Dia-R3	cdRNA05-postDia	4087.45	38111.8	3.22097	5.00E-05	0.0195319
RNA-Seq	Trans-ABySS	XLOC_004280	J1797084:0-74	cdRNA01-preDia	cdRNA05-postDia	5686.05	40047.7	2.81622	5.00E-05	0.0195319
RNA-Seq	Trans-ABySS	XLOC_004434	J1798645:0-122	cdRNA03-Dia-R2	cdRNA05-postDia	918.465	4875.96	2.40839	0.00015	0.0374403
RNA-Seq	Trans-ABySS	XLOC_004434	J1798645:0-122	cdRNA02-Dia-R1	cdRNA05-postDia	895.966	4875.96	2.44417	0.0002	0.0481888
RNA-Seq	Trans-ABySS	XLOC_004451	J1798883:0-65	cdRNA01-preDia	cdRNA05-postDia	77983.9	6161.41	-3.66184	0.0002	0.0481888
RNA-Seq	Trans-ABySS	XLOC_004779	J1802829:0-64	cdRNA01-preDia	cdRNA05-postDia	10865.6	62774.6	2.53041	5.00E-05	0.0195319
RNA-Seq	Trans-ABySS	XLOC_004909	J1804132:0-69	cdRNA03-Dia-R2	cdRNA05-postDia	8116.33	51386.9	2.6625	0.0001	0.0354705
RNA-Seq	Trans-ABySS	XLOC_005241	J1807750:0-1135	cdRNA01-preDia	cdRNA05-postDia	1.76365	20.0029	3.50357	5.00E-05	0.0195319
RNA-Seq	Trans-ABySS	XLOC_005448	J1810141:0-65	cdRNA03-Dia-R2	cdRNA05-postDia	39887	248600	2.63983	5.00E-05	0.0195319
RNA-Seq	Trans-ABySS	XLOC_005448	J1810141:0-65	cdRNA04-Dia-R3	cdRNA05-postDia	34045.8	248600	2.86828	5.00E-05	0.0195319
RNA-Seq	Trans-ABySS	XLOC_005645	J1812338:0-371	cdRNA02-Dia-R1	cdRNA05-postDia	30.3087	623.166	4.36181	5.00E-05	0.0195319
RNA-Seq	Trans-ABySS	XLOC_005645	J1812338:0-371	cdRNA01-preDia	cdRNA05-postDia	14.7172	623.166	5.40404	5.00E-05	0.0195319
RNA-Seq	Trans-ABySS	XLOC_005645	J1812338:0-371	cdRNA04-Dia-R3	cdRNA05-postDia	7.90796	623.166	6.30017	5.00E-05	0.0195319
RNA-Seq	Trans-ABySS	XLOC_005645	J1812338:0-371	cdRNA03-Dia-R2	cdRNA05-postDia	7.46348	623.166	6.38362	5.00E-05	0.0195319
RNA-Seq	Trans-ABySS	XLOC_005648	J1812358:0-95	cdRNA01-preDia	cdRNA05-postDia	4618.86	25096.1	2.44185	5.00E-05	0.0195319
RNA-Seq	Trans-ABySS	XLOC_005648	J1812358:0-95	cdRNA04-Dia-R3	cdRNA05-postDia	4115.92	25096.1	2.60817	5.00E-05	0.0195319
RNA-Seq	Trans-ABySS	XLOC_005648	J1812358:0-95	cdRNA02-Dia-R1	cdRNA05-postDia	4100.65	25096.1	2.61354	0.0001	0.0354705
RNA-Seq	Trans-ABySS	XLOC_005648	J1812358:0-95	cdRNA03-Dia-R2	cdRNA05-postDia	3836.67	25096.1	2.70953	5.00E-05	0.0195319
RNA-Seq	Trans-ABySS	XLOC_005894	J1814908:1-156	cdRNA01-preDia	cdRNA05-postDia	981.91	124.859	-2.97529	0.00015	0.0374403
RNA-Seq	Trans-ABySS	XLOC_005954	J1815624:0-89	cdRNA02-Dia-R1	cdRNA05-postDia	4895.47	36842.1	2.91184	5.00E-05	0.0195319
RNA-Seq	Trans-ABySS	XLOC_005954	J1815624:0-89	cdRNA03-Dia-R2	cdRNA05-postDia	4348.14	36842.1	3.08288	5.00E-05	0.0195319
RNA-Seq	Trans-ABySS	XLOC_005954	J1815624:0-89	cdRNA04-Dia-R3	cdRNA05-postDia	3838.04	36842.1	3.26291	5.00E-05	0.0195319
RNA-Seq	Trans-ABySS	XLOC_005954	J1815624:0-89	cdRNA01-preDia	cdRNA05-postDia	1122.25	36842.1	5.03689	5.00E-05	0.0195319
RNA-Seq	Trans-ABySS	XLOC_005985	J1816018:0-352	cdRNA01-preDia	cdRNA03-Dia-R2	204.526	34.7882	-2.55561	0.0001	0.0354705
RNA-Seq	Trans-ABySS	XLOC_006100	J1817537:0-78	cdRNA04-Dia-R3	cdRNA05-postDia	29510.6	155253	2.39531	0.00015	0.0374403

RNA-Seq	Trans-ABySS	XLOC_006100	J1817537:0-78	cdRNA03-Dia-R2	cdRNA05-postDia	28657.4	155253	2.43764	0.0001	0.0354705
RNA-Seq	Trans-ABySS	XLOC_006350	J1820068:1-147	cdRNA02-Dia-R1	cdRNA05-postDia	368.708	2666.69	2.8545	5.00E-05	0.0195319
RNA-Seq	Trans-ABySS	XLOC_006350	J1820068:1-147	cdRNA01-preDia	cdRNA05-postDia	192.954	2666.69	3.78872	5.00E-05	0.0195319
RNA-Seq	Trans-ABySS	XLOC_006350	J1820068:1-147	cdRNA04-Dia-R3	cdRNA05-postDia	117.148	2666.69	4.50865	5.00E-05	0.0195319
RNA-Seq	Trans-ABySS	XLOC_006350	J1820068:1-147	cdRNA03-Dia-R2	cdRNA05-postDia	91.8072	2666.69	4.8603	5.00E-05	0.0195319
RNA-Seq	Trans-ABySS	XLOC_006511	J1821889:2-68	cdRNA02-Dia-R1	cdRNA05-postDia	35152.6	211439	2.58853	5.00E-05	0.0195319
RNA-Seq	Trans-ABySS	XLOC_006511	J1821889:2-68	cdRNA03-Dia-R2	cdRNA05-postDia	18528.5	211439	3.51242	5.00E-05	0.0195319
RNA-Seq	Trans-ABySS	XLOC_006511	J1821889:2-68	cdRNA04-Dia-R3	cdRNA05-postDia	18192.7	211439	3.53881	5.00E-05	0.0195319
RNA-Seq	Trans-ABySS	XLOC_006546	J1822273:0-107	cdRNA03-Dia-R2	cdRNA05-postDia	13029.5	1064.24	-3.61389	5.00E-05	0.0195319
RNA-Seq	Trans-ABySS	XLOC_006546	J1822273:0-107	cdRNA04-Dia-R3	cdRNA05-postDia	10784.9	1064.24	-3.34112	5.00E-05	0.0195319
RNA-Seq	Trans-ABySS	XLOC_006546	J1822273:0-107	cdRNA02-Dia-R1	cdRNA05-postDia	8144.37	1064.24	-2.93598	0.0001	0.0354705
RNA-Seq	Trans-ABySS	XLOC_006546	J1822273:0-107	cdRNA01-preDia	cdRNA05-postDia	7199.21	1064.24	-2.75802	0.00015	0.0374403
RNA-Seq	Trans-ABySS	XLOC_006635	J1823168:1-83	cdRNA04-Dia-R3	cdRNA05-postDia	1896.37	13596.8	2.84196	0.00015	0.0374403
RNA-Seq	Trans-ABySS	XLOC_006656	J1823424:1-235	cdRNA04-Dia-R3	cdRNA05-postDia	16.1247	179.573	3.47722	0.0002	0.0481888
RNA-Seq	Trans-ABySS	XLOC_006945	J1825996:0-82	cdRNA04-Dia-R3	cdRNA05-postDia	3242.18	18743.3	2.53134	0.00015	0.0374403
RNA-Seq	Trans-ABySS	XLOC_006985	J1826298:0-261	cdRNA02-Dia-R1	cdRNA05-postDia	67.5895	468.563	2.79337	5.00E-05	0.0195319
RNA-Seq	Trans-ABySS	XLOC_007003	J1826443:15-372	cdRNA01-preDia	cdRNA03-Dia-R2	642.213	40.6539	-3.98159	5.00E-05	0.0195319
RNA-Seq	Trans-ABySS	XLOC_007003	J1826443:15-372	cdRNA01-preDia	cdRNA04-Dia-R3	642.213	51.69	-3.6351	5.00E-05	0.0195319
RNA-Seq	Trans-ABySS	XLOC_007003	J1826443:15-372	cdRNA01-preDia	cdRNA02-Dia-R1	642.213	106.053	-2.59827	5.00E-05	0.0195319
RNA-Seq	Trans-ABySS	XLOC_007173	J1827670:0-213	cdRNA01-preDia	cdRNA04-Dia-R3	40.5697	298.883	2.88111	0.0002	0.0481888
RNA-Seq	Trans-ABySS	XLOC_007173	J1827670:0-213	cdRNA01-preDia	cdRNA03-Dia-R2	40.5697	311.027	2.93856	5.00E-05	0.0195319
RNA-Seq	Trans-ABySS	XLOC_007189	J1827802:0-77	cdRNA01-preDia	cdRNA05-postDia	19343.9	3402.85	-2.50706	0.0002	0.0481888
RNA-Seq	Trans-ABySS	XLOC_007440	J1829907:0-214	cdRNA02-Dia-R1	cdRNA05-postDia	159.361	1170.74	2.87705	5.00E-05	0.0195319
RNA-Seq	Trans-ABySS	XLOC_007440	J1829907:0-214	cdRNA04-Dia-R3	cdRNA05-postDia	60.2776	1170.74	4.27966	5.00E-05	0.0195319
RNA-Seq	Trans-ABySS	XLOC_007440	J1829907:0-214	cdRNA01-preDia	cdRNA05-postDia	63.1119	1170.74	4.21337	5.00E-05	0.0195319
RNA-Seq	Trans-ABySS	XLOC_007440	J1829907:0-214	cdRNA03-Dia-R2	cdRNA05-postDia	59.2058	1170.74	4.30554	5.00E-05	0.0195319
RNA-Seq	Trans-ABySS	XLOC_007479	J1830264:0-312	cdRNA02-Dia-R1	cdRNA05-postDia	78.5566	1887.73	4.58678	5.00E-05	0.0195319
RNA-Seq	Trans-ABySS	XLOC_007479	J1830264:0-312	cdRNA01-preDia	cdRNA05-postDia	45.302	1887.73	5.38094	5.00E-05	0.0195319
RNA-Seq	Trans-ABySS	XLOC_007479	J1830264:0-312	cdRNA04-Dia-R3	cdRNA05-postDia	28.9106	1887.73	6.02891	5.00E-05	0.0195319
RNA-Seq	Trans-ABySS	XLOC_007479	J1830264:0-312	cdRNA03-Dia-R2	cdRNA05-postDia	27.7924	1887.73	6.08582	5.00E-05	0.0195319
RNA-Seq	Trans-ABySS	XLOC_007673	J1831685:0-127	cdRNA02-Dia-R1	cdRNA05-postDia	346.952	4139.42	3.57662	5.00E-05	0.0195319
RNA-Seq	Trans-ABySS	XLOC_007673	J1831685:0-127	cdRNA01-preDia	cdRNA05-postDia	117.775	4139.42	5.13533	0.0002	0.0481888
RNA-Seq	Trans-ABySS	XLOC_007726	J1832045:0-92	cdRNA01-preDia	cdRNA05-postDia	7394.77	43276.1	2.54899	5.00E-05	0.0195319
RNA-Seq	Trans-ABySS	XLOC_007726	J1832045:0-92	cdRNA04-Dia-R3	cdRNA05-postDia	7128.04	43276.1	2.60199	5.00E-05	0.0195319
RNA-Seq	Trans-ABySS	XLOC_007726	J1832045:0-92	cdRNA03-Dia-R2	cdRNA05-postDia	6800.52	43276.1	2.66985	5.00E-05	0.0195319
RNA-Seq	Trans-ABySS	XLOC_007772	J1832405:5-563	cdRNA01-preDia	cdRNA04-Dia-R3	27.4426	2.05006	-3.74268	0.0001	0.0354705
RNA-Seq	Trans-ABySS	XLOC_007772	J1832405:5-563	cdRNA01-preDia	cdRNA03-Dia-R2	27.4426	3.40221	-3.01187	0.0001	0.0354705
RNA-Seq	Trans-ABySS	XLOC_008084	J1834745:0-227	cdRNA04-Dia-R3	cdRNA05-postDia	92.0129	637.119	2.79166	5.00E-05	0.0195319
RNA-Seq	Trans-ABySS	XLOC_008084	J1834745:0-227	cdRNA03-Dia-R2	cdRNA05-postDia	80.6382	637.119	2.98203	5.00E-05	0.0195319
RNA-Seq	Trans-ABySS	XLOC_008111	J1834930:0-82	cdRNA01-preDia	cdRNA05-postDia	2091.41	19505.7	3.22135	5.00E-05	0.0195319
RNA-Seq	Trans-ABySS	XLOC_008111	J1834930:0-82	cdRNA02-Dia-R1	cdRNA05-postDia	1877.21	19505.7	3.37724	5.00E-05	0.0195319
RNA-Seq	Trans-ABySS	XLOC_008111	J1834930:0-82	cdRNA03-Dia-R2	cdRNA05-postDia	1598.02	19505.7	3.60954	0.00015	0.0374403
RNA-Seq	Trans-ABySS	XLOC_008186	J1835384:0-84	cdRNA01-preDia	cdRNA05-postDia	7149.33	37329.5	2.38444	5.00E-05	0.0195319
RNA-Seq	Trans-ABySS	XLOC_008434	J1836519:0-112	cdRNA02-Dia-R1	cdRNA05-postDia	280.795	3439.64	3.61467	0.0002	0.0481888
RNA-Seq	Trans-ABySS	XLOC_008454	J1836589:0-109	cdRNA01-preDia	cdRNA05-postDia	667.983	6560.41	3.2959	5.00E-05	0.0195319
RNA-Seq	Trans-ABySS	XLOC_008454	J1836589:0-109	cdRNA02-Dia-R1	cdRNA05-postDia	618.095	6560.41	3.40789	5.00E-05	0.0195319
RNA-Seq	Trans-ABySS	XLOC_008454	J1836589:0-109	cdRNA04-Dia-R3	cdRNA05-postDia	432.848	6560.41	3.92185	5.00E-05	0.0195319

RNA-Seq	Trans-ABySS	XLOC_008454	J1836589:0-109	cdRNA03-Dia-R2	cdRNA05-postDia	301.526	6560.41	4.44343	5.00E-05	0.0195319
RNA-Seq	Trans-ABySS	XLOC_008506	J1836904:0-87	cdRNA02-Dia-R1	cdRNA05-postDia	11165	60829.1	2.44577	0.0002	0.0481888
RNA-Seq	Trans-ABySS	XLOC_008506	J1836904:0-87	cdRNA01-preDia	cdRNA05-postDia	4490.09	60829.1	3.75995	5.00E-05	0.0195319
RNA-Seq	Trans-ABySS	XLOC_008520	J1837060:0-296	cdRNA02-Dia-R1	cdRNA05-postDia	191.86	1265.36	2.72142	0.0002	0.0481888
RNA-Seq	Trans-ABySS	XLOC_008520	J1837060:0-296	cdRNA01-preDia	cdRNA05-postDia	156.781	1265.36	3.01273	5.00E-05	0.0195319
RNA-Seq	Trans-ABySS	XLOC_008635	R1745638:0-148	cdRNA04-Dia-R3	cdRNA05-postDia	5135.42	46325.9	3.17327	5.00E-05	0.0195319
RNA-Seq	Trans-ABySS	XLOC_008635	R1745638:0-148	cdRNA03-Dia-R2	cdRNA05-postDia	4916.08	46325.9	3.23624	5.00E-05	0.0195319
RNA-Seq	Trans-ABySS	XLOC_008693	R1745736:0-74	cdRNA01-preDia	cdRNA05-postDia	9979.6	58548.6	2.55258	0.00015	0.0374403
RNA-Seq	Trans-ABySS	XLOC_008700	R1745751:0-100	cdRNA03-Dia-R2	cdRNA05-postDia	17177.9	120326	2.80832	0.0001	0.0354705
RNA-Seq	Trans-ABySS	XLOC_008700	R1745751:0-100	cdRNA04-Dia-R3	cdRNA05-postDia	16158.5	120326	2.89658	5.00E-05	0.0195319
RNA-Seq	Trans-ABySS	XLOC_008707	R1745764:0-589	cdRNA01-preDia	cdRNA03-Dia-R2	4533.18	236.822	-4.25865	5.00E-05	0.0195319
RNA-Seq	Trans-ABySS	XLOC_008707	R1745764:0-589	cdRNA01-preDia	cdRNA04-Dia-R3	4533.18	246.379	-4.20157	5.00E-05	0.0195319
RNA-Seq	Trans-ABySS	XLOC_008719	R1745786:0-60	cdRNA03-Dia-R2	cdRNA05-postDia	73786.5	430105	2.54326	5.00E-05	0.0195319
RNA-Seq	Trans-ABySS	XLOC_008719	R1745786:0-60	cdRNA04-Dia-R3	cdRNA05-postDia	63049	430105	2.77014	5.00E-05	0.0195319
RNA-Seq	Trans-ABySS	XLOC_008755	R1745864:0-73	cdRNA03-Dia-R2	cdRNA05-postDia	12196.5	62820.2	2.36476	0.0001	0.0354705
RNA-Seq	Trans-ABySS	XLOC_008787	R1745934:10-519	cdRNA02-Dia-R1	cdRNA05-postDia	39.7568	317.347	2.99679	5.00E-05	0.0195319
RNA-Seq	Trans-ABySS	XLOC_008787	R1745934:10-519	cdRNA03-Dia-R2	cdRNA05-postDia	30.4202	317.347	3.38296	5.00E-05	0.0195319
RNA-Seq	Trans-ABySS	XLOC_008787	R1745934:10-519	cdRNA04-Dia-R3	cdRNA05-postDia	28.2728	317.347	3.48857	5.00E-05	0.0195319
RNA-Seq	Trans-ABySS	XLOC_008787	R1745934:10-519	cdRNA01-preDia	cdRNA05-postDia	28.1128	317.347	3.49676	5.00E-05	0.0195319
RNA-Seq	Trans-ABySS	XLOC_008788	R1745935:3-416	cdRNA03-Dia-R2	cdRNA05-postDia	868.946	66.6036	-3.7056	5.00E-05	0.0195319
RNA-Seq	Trans-ABySS	XLOC_008788	R1745935:3-416	cdRNA04-Dia-R3	cdRNA05-postDia	847.73	66.6036	-3.66993	5.00E-05	0.0195319
RNA-Seq	Trans-ABySS	XLOC_008788	R1745935:3-416	cdRNA02-Dia-R1	cdRNA05-postDia	430.17	66.6036	-2.69124	5.00E-05	0.0195319
RNA-Seq	Trans-ABySS	XLOC_008800	R1745967:0-119	cdRNA01-preDia	cdRNA05-postDia	1596.49	11772.5	2.88244	5.00E-05	0.0195319
RNA-Seq	Trans-ABySS	XLOC_008800	R1745967:0-119	cdRNA02-Dia-R1	cdRNA05-postDia	1402.22	11772.5	3.06963	5.00E-05	0.0195319
RNA-Seq	Trans-ABySS	XLOC_008800	R1745967:0-119	cdRNA03-Dia-R2	cdRNA05-postDia	855.887	11772.5	3.78185	5.00E-05	0.0195319
RNA-Seq	Trans-ABySS	XLOC_008800	R1745967:0-119	cdRNA04-Dia-R3	cdRNA05-postDia	608.789	11772.5	4.27333	5.00E-05	0.0195319
RNA-Seq	Trans-ABySS	XLOC_008802	R1745969:0-82	cdRNA01-preDia	cdRNA05-postDia	8823.15	49876.2	2.49899	0.0001	0.0354705
RNA-Seq	Trans-ABySS	XLOC_008817	R1745994:0-62	cdRNA01-preDia	cdRNA05-postDia	18902.7	165778	3.13259	0.0002	0.0481888
RNA-Seq	Trans-ABySS	XLOC_008831	R1746024:0-68	cdRNA01-preDia	cdRNA05-postDia	25493.4	153154	2.58679	5.00E-05	0.0195319
RNA-Seq	Trans-ABySS	XLOC_008840	R1746042:0-1917	cdRNA01-preDia	cdRNA05-postDia	20.7149	154.064	2.89479	5.00E-05	0.0195319
RNA-Seq	Trans-ABySS	XLOC_008841	R1746043:0-81	cdRNA02-Dia-R1	cdRNA05-postDia	8270.27	64144.7	2.95532	5.00E-05	0.0195319
RNA-Seq	Trans-ABySS	XLOC_008841	R1746043:0-81	cdRNA04-Dia-R3	cdRNA05-postDia	5875.56	64144.7	3.44853	5.00E-05	0.0195319
RNA-Seq	Trans-ABySS	XLOC_008841	R1746043:0-81	cdRNA03-Dia-R2	cdRNA05-postDia	5866.59	64144.7	3.45074	5.00E-05	0.0195319
RNA-Seq	Trans-ABySS	XLOC_008841	R1746043:0-81	cdRNA01-preDia	cdRNA05-postDia	1813.46	64144.7	5.14451	5.00E-05	0.0195319
RNA-Seq	Trans-ABySS	XLOC_008867	R1746093:0-177	cdRNA01-preDia	cdRNA05-postDia	390.892	3008.18	2.94405	5.00E-05	0.0195319
RNA-Seq	Trans-ABySS	XLOC_008871	R1746098:0-84	cdRNA04-Dia-R3	cdRNA05-postDia	12951.7	1457.31	-3.15176	0.0001	0.0354705
RNA-Seq	Trans-ABySS	XLOC_008873	R1746100:0-90	cdRNA01-preDia	cdRNA05-postDia	2779.95	30006.1	3.43212	5.00E-05	0.0195319
RNA-Seq	Trans-ABySS	XLOC_008884	R1746125:0-66	cdRNA03-Dia-R2	cdRNA05-postDia	4331.34	44966.6	3.37597	0.0002	0.0481888
RNA-Seq	Trans-ABySS	XLOC_008887	R1746129:0-94	cdRNA02-Dia-R1	cdRNA05-postDia	19020.1	2294.17	-3.05148	5.00E-05	0.0195319
RNA-Seq	Trans-ABySS	XLOC_008887	R1746129:0-94	cdRNA01-preDia	cdRNA05-postDia	14392	2294.17	-2.64922	0.0001	0.0354705
RNA-Seq	Trans-ABySS	XLOC_008931	R1746216:0-108	cdRNA01-preDia	cdRNA03-Dia-R2	1693.1	9028.99	2.4149	0.0001	0.0354705
RNA-Seq	Trans-ABySS	XLOC_008931	R1746216:0-108	cdRNA01-preDia	cdRNA04-Dia-R3	1693.1	9341.5	2.46399	5.00E-05	0.0195319
RNA-Seq	Trans-ABySS	XLOC_008933	R1746221:7-1510	cdRNA01-preDia	cdRNA04-Dia-R3	17.4154	125.015	2.84367	5.00E-05	0.0195319
RNA-Seq	Trans-ABySS	XLOC_008933	R1746221:7-1510	cdRNA01-preDia	cdRNA03-Dia-R2	17.4154	134.305	2.94707	5.00E-05	0.0195319
RNA-Seq	Trans-ABySS	XLOC_008935	R1746224:0-2132	cdRNA02-Dia-R1	cdRNA05-postDia	37.145	609.952	4.03746	5.00E-05	0.0195319
RNA-Seq	Trans-ABySS	XLOC_008935	R1746224:0-2132	cdRNA01-preDia	cdRNA05-postDia	18.0974	609.952	5.07484	5.00E-05	0.0195319
RNA-Seq	Trans-ABySS	XLOC_008935	R1746224:0-2132	cdRNA04-Dia-R3	cdRNA05-postDia	11.4477	609.952	5.73557	5.00E-05	0.0195319

RNA-Seq	Trans-ABySS	XLOC_008935	R1746224:0-2132	cdRNA03-Dia-R2	cdRNA05-postDia	11.2446	609.952	5.7614	5.00E-05	0.0195319
RNA-Seq	Trans-ABySS	XLOC_008941	R1746243:0-619	cdRNA01-preDia	cdRNA02-Dia-R1	15.1664	86.1198	2.50547	0.0001	0.0354705
RNA-Seq	Trans-ABySS	XLOC_008941	R1746243:0-619	cdRNA01-preDia	cdRNA05-postDia	15.1664	286.868	4.24144	5.00E-05	0.0195319
RNA-Seq	Trans-ABySS	XLOC_008964	R1746292:0-839	cdRNA01-preDia	cdRNA05-postDia	32.4654	166.116	2.35521	0.00015	0.0374403
RNA-Seq	Trans-ABySS	XLOC_008973	R1746310:0-112	cdRNA03-Dia-R2	cdRNA05-postDia	2669.9	15878.7	2.57223	5.00E-05	0.0195319
RNA-Seq	Trans-ABySS	XLOC_008973	R1746310:0-112	cdRNA01-preDia	cdRNA05-postDia	1723.33	15878.7	3.20382	5.00E-05	0.0195319
RNA-Seq	Trans-ABySS	XLOC_008985	R1746328:0-1541	cdRNA03-Dia-R2	cdRNA05-postDia	128.486	20.6278	-2.63894	5.00E-05	0.0195319
RNA-Seq	Trans-ABySS	XLOC_008985	R1746328:0-1541	cdRNA04-Dia-R3	cdRNA05-postDia	123.729	20.6278	-2.58452	0.0001	0.0354705
RNA-Seq	Trans-ABySS	XLOC_008985	R1746328:0-1541	cdRNA01-preDia	cdRNA03-Dia-R2	20.707	128.486	2.63341	0.0002	0.0481888
RNA-Seq	Trans-ABySS	XLOC_008994	R1746341:0-63	cdRNA01-preDia	cdRNA05-postDia	16546.3	129711	2.97072	0.0002	0.0481888
RNA-Seq	Trans-ABySS	XLOC_008996	R1746343:0-79	cdRNA01-preDia	cdRNA05-postDia	3433.54	22356.3	2.70291	0.00015	0.0374403
RNA-Seq	Trans-ABySS	XLOC_008999	R1746351:0-836	cdRNA02-Dia-R1	cdRNA05-postDia	9.99206	116.04	3.5377	5.00E-05	0.0195319
RNA-Seq	Trans-ABySS	XLOC_008999	R1746351:0-836	cdRNA04-Dia-R3	cdRNA05-postDia	3.64757	116.04	4.99155	5.00E-05	0.0195319
RNA-Seq	Trans-ABySS	XLOC_008999	R1746351:0-836	cdRNA03-Dia-R2	cdRNA05-postDia	3.31733	116.04	5.12846	5.00E-05	0.0195319
RNA-Seq	Trans-ABySS	XLOC_008999	R1746351:0-836	cdRNA01-preDia	cdRNA05-postDia	3.31968	116.04	5.12744	5.00E-05	0.0195319
RNA-Seq	Trans-ABySS	XLOC_009004	R1746356:6-2691	cdRNA03-Dia-R2	cdRNA05-postDia	28.4262	261.483	3.20142	0.0002	0.0481888
RNA-Seq	Trans-ABySS	XLOC_009004	R1746356:6-2691	cdRNA04-Dia-R3	cdRNA05-postDia	27.6345	261.483	3.24217	0.00015	0.0374403
RNA-Seq	Trans-ABySS	XLOC_009023	R1746393:0-88	cdRNA03-Dia-R2	cdRNA05-postDia	2052.57	11753.5	2.51759	0.0002	0.0481888
RNA-Seq	Trans-ABySS	XLOC_009043	R1746432:0-83	cdRNA02-Dia-R1	cdRNA05-postDia	6731.05	36553.8	2.44112	0.00015	0.0374403
RNA-Seq	Trans-ABySS	XLOC_009043	R1746432:0-83	cdRNA01-preDia	cdRNA05-postDia	2208.21	36553.8	4.04907	5.00E-05	0.0195319
RNA-Seq	Trans-ABySS	XLOC_009045	R1746434:1-2067	cdRNA03-Dia-R2	cdRNA05-postDia	256.556	28.0519	-3.19311	5.00E-05	0.0195319
RNA-Seq	Trans-ABySS	XLOC_009045	R1746434:1-2067	cdRNA04-Dia-R3	cdRNA05-postDia	256.006	28.0519	-3.19001	5.00E-05	0.0195319
RNA-Seq	Trans-ABySS	XLOC_009051	R1746442:0-311	cdRNA02-Dia-R1	cdRNA05-postDia	91.142	611.229	2.74553	5.00E-05	0.0195319
RNA-Seq	Trans-ABySS	XLOC_009051	R1746442:0-311	cdRNA04-Dia-R3	cdRNA05-postDia	67.6339	611.229	3.1759	5.00E-05	0.0195319
RNA-Seq	Trans-ABySS	XLOC_009051	R1746442:0-311	cdRNA03-Dia-R2	cdRNA05-postDia	51.176	611.229	3.57818	5.00E-05	0.0195319
RNA-Seq	Trans-ABySS	XLOC_009055	R1746446:0-144	cdRNA04-Dia-R3	cdRNA05-postDia	623.153	3690.81	2.56628	5.00E-05	0.0195319
RNA-Seq	Trans-ABySS	XLOC_009055	R1746446:0-144	cdRNA01-preDia	cdRNA05-postDia	605.245	3690.81	2.60835	5.00E-05	0.0195319
RNA-Seq	Trans-ABySS	XLOC_009055	R1746446:0-144	cdRNA03-Dia-R2	cdRNA05-postDia	597.328	3690.81	2.62734	5.00E-05	0.0195319
RNA-Seq	Trans-ABySS	XLOC_009062	R1746463:0-87	cdRNA01-preDia	cdRNA05-postDia	1641.54	15113.4	3.20271	5.00E-05	0.0195319
RNA-Seq	Trans-ABySS	XLOC_009062	R1746463:0-87	cdRNA02-Dia-R1	cdRNA05-postDia	1493.41	15113.4	3.33914	0.0001	0.0354705
RNA-Seq	Trans-ABySS	XLOC_009070	R1746478:0-128	cdRNA03-Dia-R2	cdRNA05-postDia	669.546	5426.57	3.01879	5.00E-05	0.0195319
RNA-Seq	Trans-ABySS	XLOC_009070	R1746478:0-128	cdRNA02-Dia-R1	cdRNA05-postDia	626.995	5426.57	3.11352	5.00E-05	0.0195319
RNA-Seq	Trans-ABySS	XLOC_009070	R1746478:0-128	cdRNA04-Dia-R3	cdRNA05-postDia	460.038	5426.57	3.56022	5.00E-05	0.0195319
RNA-Seq	Trans-ABySS	XLOC_009070	R1746478:0-128	cdRNA01-preDia	cdRNA05-postDia	149.205	5426.57	5.18467	5.00E-05	0.0195319
RNA-Seq	Trans-ABySS	XLOC_009096	R1746529:0-101	cdRNA01-preDia	cdRNA05-postDia	5072.47	721.073	-2.81447	0.00015	0.0374403
RNA-Seq	Trans-ABySS	XLOC_009115	R1746577:21-575	cdRNA01-preDia	cdRNA04-Dia-R3	129.008	9.87954	-3.70687	5.00E-05	0.0195319
RNA-Seq	Trans-ABySS	XLOC_009115	R1746577:21-575	cdRNA01-preDia	cdRNA03-Dia-R2	129.008	10.8331	-3.57395	5.00E-05	0.0195319
RNA-Seq	Trans-ABySS	XLOC_009115	R1746577:21-575	cdRNA01-preDia	cdRNA02-Dia-R1	129.008	16.8909	-2.93314	5.00E-05	0.0195319
RNA-Seq	Trans-ABySS	XLOC_009115	R1746577:21-575	cdRNA02-Dia-R1	cdRNA05-postDia	16.8909	144.798	3.09972	5.00E-05	0.0195319
RNA-Seq	Trans-ABySS	XLOC_009115	R1746577:21-575	cdRNA03-Dia-R2	cdRNA05-postDia	10.8331	144.798	3.74052	5.00E-05	0.0195319
RNA-Seq	Trans-ABySS	XLOC_009115	R1746577:21-575	cdRNA04-Dia-R3	cdRNA05-postDia	9.87954	144.798	3.87345	5.00E-05	0.0195319
RNA-Seq	Trans-ABySS	XLOC_009120	R1746588:0-342	cdRNA01-preDia	cdRNA03-Dia-R2	17.7111	100.403	2.50308	0.0002	0.0481888
RNA-Seq	Trans-ABySS	XLOC_009126	R1746598:0-137	cdRNA03-Dia-R2	cdRNA05-postDia	857.184	5398.38	2.65485	5.00E-05	0.0195319
RNA-Seq	Trans-ABySS	XLOC_009126	R1746598:0-137	cdRNA04-Dia-R3	cdRNA05-postDia	807.815	5398.38	2.74043	5.00E-05	0.0195319
RNA-Seq	Trans-ABySS	XLOC_009126	R1746598:0-137	cdRNA02-Dia-R1	cdRNA05-postDia	734.168	5398.38	2.87834	5.00E-05	0.0195319
RNA-Seq	Trans-ABySS	XLOC_009126	R1746598:0-137	cdRNA01-preDia	cdRNA05-postDia	361.281	5398.38	3.90133	5.00E-05	0.0195319
RNA-Seq	Trans-ABySS	XLOC_009137	R1746620:1-237	cdRNA02-Dia-R1	cdRNA05-postDia	129.603	2342.57	4.17592	5.00E-05	0.0195319

RNA-Seq	Trans-ABySS	XLOC_009137	R1746620:1-237	cdRNA01-preDia	cdRNA05-postDia	62.3833	2342.57	5.23079	5.00E-05	0.0195319
RNA-Seq	Trans-ABySS	XLOC_009137	R1746620:1-237	cdRNA04-Dia-R3	cdRNA05-postDia	42.7032	2342.57	5.7776	5.00E-05	0.0195319
RNA-Seq	Trans-ABySS	XLOC_009137	R1746620:1-237	cdRNA03-Dia-R2	cdRNA05-postDia	40.9787	2342.57	5.83707	5.00E-05	0.0195319
RNA-Seq	Trans-ABySS	XLOC_009139	R1746627:0-86	cdRNA03-Dia-R2	cdRNA05-postDia	12708	1342.73	-3.24249	0.0002	0.0481888
RNA-Seq	Trans-ABySS	XLOC_009139	R1746627:0-86	cdRNA04-Dia-R3	cdRNA05-postDia	10294.4	1342.73	-2.93862	0.0002	0.0481888
RNA-Seq	Trans-ABySS	XLOC_009162	R1746668:0-96	cdRNA01-preDia	cdRNA05-postDia	3405.53	19161	2.49222	5.00E-05	0.0195319
RNA-Seq	Trans-ABySS	XLOC_009162	R1746668:0-96	cdRNA02-Dia-R1	cdRNA05-postDia	2707.84	19161	2.82295	5.00E-05	0.0195319
RNA-Seq	Trans-ABySS	XLOC_009171	R1746684:0-108	cdRNA01-preDia	cdRNA05-postDia	1603.98	12890.4	3.00656	5.00E-05	0.0195319
RNA-Seq	Trans-ABySS	XLOC_009181	R1746705:0-1007	cdRNA02-Dia-R1	cdRNA05-postDia	32.6702	203.642	2.63999	0.0002	0.0481888
RNA-Seq	Trans-ABySS	XLOC_009181	R1746705:0-1007	cdRNA03-Dia-R2	cdRNA05-postDia	25.1647	203.642	3.01656	5.00E-05	0.0195319
RNA-Seq	Trans-ABySS	XLOC_009181	R1746705:0-1007	cdRNA04-Dia-R3	cdRNA05-postDia	25.1463	203.642	3.01762	5.00E-05	0.0195319
RNA-Seq	Trans-ABySS	XLOC_009185	R1746712:0-972	cdRNA04-Dia-R3	cdRNA05-postDia	70.5389	8.42914	-3.06496	5.00E-05	0.0195319
RNA-Seq	Trans-ABySS	XLOC_009185	R1746712:0-972	cdRNA03-Dia-R2	cdRNA05-postDia	68.4797	8.42914	-3.02222	5.00E-05	0.0195319
RNA-Seq	Trans-ABySS	XLOC_009185	R1746712:0-972	cdRNA01-preDia	cdRNA05-postDia	67.0487	8.42914	-2.99175	5.00E-05	0.0195319
RNA-Seq	Trans-ABySS	XLOC_009185	R1746712:0-972	cdRNA02-Dia-R1	cdRNA05-postDia	46.6242	8.42914	-2.46762	5.00E-05	0.0195319
RNA-Seq	Trans-ABySS	XLOC_009200	R1746738:0-99	cdRNA03-Dia-R2	cdRNA05-postDia	1331.52	7915.65	2.57164	0.00015	0.0374403
RNA-Seq	Trans-ABySS	XLOC_009200	R1746738:0-99	cdRNA04-Dia-R3	cdRNA05-postDia	1200.77	7915.65	2.72075	5.00E-05	0.0195319
RNA-Seq	Trans-ABySS	XLOC_009200	R1746738:0-99	cdRNA01-preDia	cdRNA05-postDia	1178.16	7915.65	2.74817	0.0001	0.0354705
RNA-Seq	Trans-ABySS	XLOC_009204	R1746745:0-934	cdRNA01-preDia	cdRNA05-postDia	488.399	4.79839	-6.66936	5.00E-05	0.0195319
RNA-Seq	Trans-ABySS	XLOC_009204	R1746745:0-934	cdRNA02-Dia-R1	cdRNA05-postDia	101.323	4.79839	-4.40027	5.00E-05	0.0195319
RNA-Seq	Trans-ABySS	XLOC_009204	R1746745:0-934	cdRNA03-Dia-R2	cdRNA05-postDia	94.7147	4.79839	-4.30297	5.00E-05	0.0195319
RNA-Seq	Trans-ABySS	XLOC_009204	R1746745:0-934	cdRNA04-Dia-R3	cdRNA05-postDia	90.5266	4.79839	-4.23772	5.00E-05	0.0195319
RNA-Seq	Trans-ABySS	XLOC_009225	R1746782:0-156	cdRNA03-Dia-R2	cdRNA05-postDia	75.1254	986.483	3.71492	0.00015	0.0374403
RNA-Seq	Trans-ABySS	XLOC_009244	R1746818:0-321	cdRNA02-Dia-R1	cdRNA05-postDia	15.5119	403.56	4.70133	5.00E-05	0.0195319
RNA-Seq	Trans-ABySS	XLOC_009244	R1746818:0-321	cdRNA04-Dia-R3	cdRNA05-postDia	5.73503	403.56	6.13684	0.0002	0.0481888
RNA-Seq	Trans-ABySS	XLOC_009244	R1746818:0-321	cdRNA03-Dia-R2	cdRNA05-postDia	4.49446	403.56	6.48849	0.0002	0.0481888
RNA-Seq	Trans-ABySS	XLOC_009269	R1746866:0-67	cdRNA01-preDia	cdRNA05-postDia	8612.62	73637.5	3.09592	0.00015	0.0374403
RNA-Seq	Trans-ABySS	XLOC_009281	R1746883:0-1257	cdRNA03-Dia-R2	cdRNA05-postDia	338.52	27.9598	-3.59782	5.00E-05	0.0195319
RNA-Seq	Trans-ABySS	XLOC_009281	R1746883:0-1257	cdRNA04-Dia-R3	cdRNA05-postDia	328.428	27.9598	-3.55416	5.00E-05	0.0195319
RNA-Seq	Trans-ABySS	XLOC_009321	R1746959:0-332	cdRNA01-preDia	cdRNA05-postDia	21.8389	144.164	2.72273	0.0002	0.0481888
RNA-Seq	Trans-ABySS	XLOC_009321	R1746959:0-332	cdRNA03-Dia-R2	cdRNA05-postDia	17.9141	144.164	3.00854	5.00E-05	0.0195319
RNA-Seq	Trans-ABySS	XLOC_009321	R1746959:0-332	cdRNA02-Dia-R1	cdRNA05-postDia	14.5273	144.164	3.31087	5.00E-05	0.0195319
RNA-Seq	Trans-ABySS	XLOC_009329	R1746979:0-2242	cdRNA01-preDia	cdRNA03-Dia-R2	5.57259	55.6178	3.31913	5.00E-05	0.0195319
RNA-Seq	Trans-ABySS	XLOC_009329	R1746979:0-2242	cdRNA01-preDia	cdRNA04-Dia-R3	5.57259	57.2491	3.36083	5.00E-05	0.0195319
RNA-Seq	Trans-ABySS	XLOC_009338	R1746992:0-64	cdRNA01-preDia	cdRNA05-postDia	7761.16	72734.1	3.22829	5.00E-05	0.0195319
RNA-Seq	Trans-ABySS	XLOC_009352	R1747013:0-378	cdRNA02-Dia-R1	cdRNA05-postDia	22.5533	296.942	3.71877	5.00E-05	0.0195319
RNA-Seq	Trans-ABySS	XLOC_009352	R1747013:0-378	cdRNA01-preDia	cdRNA05-postDia	3.66433	296.942	6.34049	5.00E-05	0.0195319
RNA-Seq	Trans-ABySS	XLOC_009352	R1747013:0-378	cdRNA03-Dia-R2	cdRNA05-postDia	3.32783	296.942	6.47945	0.0001	0.0354705
RNA-Seq	Trans-ABySS	XLOC_009365	R1747031:0-379	cdRNA03-Dia-R2	cdRNA05-postDia	28.3135	207.726	2.87512	5.00E-05	0.0195319
RNA-Seq	Trans-ABySS	XLOC_009388	R1747071:0-941	cdRNA01-preDia	cdRNA03-Dia-R2	13.9861	104.891	2.90683	5.00E-05	0.0195319
RNA-Seq	Trans-ABySS	XLOC_009397	R1747094:0-1764	cdRNA03-Dia-R2	cdRNA05-postDia	61.2502	10.545	-2.53816	0.0002	0.0481888
RNA-Seq	Trans-ABySS	XLOC_009424	R1747152:0-143	cdRNA03-Dia-R2	cdRNA05-postDia	132.709	1220.19	3.20076	0.00015	0.0374403
RNA-Seq	Trans-ABySS	XLOC_009426	R1747155:0-792	cdRNA02-Dia-R1	cdRNA05-postDia	10.7397	57.988	2.4328	0.0001	0.0354705
RNA-Seq	Trans-ABySS	XLOC_009426	R1747155:0-792	cdRNA01-preDia	cdRNA05-postDia	4.7316	57.988	3.61536	5.00E-05	0.0195319
RNA-Seq	Trans-ABySS	XLOC_009430	R1747159:0-1103	cdRNA01-preDia	cdRNA04-Dia-R3	40.6379	225.703	2.47353	0.00015	0.0374403
RNA-Seq	Trans-ABySS	XLOC_009438	R1747171:0-125	cdRNA02-Dia-R1	cdRNA05-postDia	683.164	4086.19	2.58045	0.0001	0.0354705
RNA-Seq	Trans-ABySS	XLOC_009438	R1747171:0-125	cdRNA04-Dia-R3	cdRNA05-postDia	506.471	4086.19	3.01221	5.00E-05	0.0195319

RNA-Seq	Trans-ABySS	XLOC_009438	R1747171:0-125	cdRNA01-preDia	cdRNA05-postDia	483.131	4086.19	3.08027	5.00E-05	0.0195319
RNA-Seq	Trans-ABySS	XLOC_009484	R1747255:0-596	cdRNA02-Dia-R1	cdRNA05-postDia	25.9636	499.152	4.26492	5.00E-05	0.0195319
RNA-Seq	Trans-ABySS	XLOC_009484	R1747255:0-596	cdRNA01-preDia	cdRNA05-postDia	7.21367	499.152	6.1126	5.00E-05	0.0195319
RNA-Seq	Trans-ABySS	XLOC_009484	R1747255:0-596	cdRNA03-Dia-R2	cdRNA05-postDia	6.6	499.152	6.24087	5.00E-05	0.0195319
RNA-Seq	Trans-ABySS	XLOC_009484	R1747255:0-596	cdRNA04-Dia-R3	cdRNA05-postDia	5.33619	499.152	6.54752	5.00E-05	0.0195319
RNA-Seq	Trans-ABySS	XLOC_009501	R1747280:0-67	cdRNA01-preDia	cdRNA05-postDia	9716.81	70631.9	2.86177	5.00E-05	0.0195319
RNA-Seq	Trans-ABySS	XLOC_009501	R1747280:0-67	cdRNA02-Dia-R1	cdRNA05-postDia	8782.59	70631.9	3.0076	0.0002	0.0481888
RNA-Seq	Trans-ABySS	XLOC_009501	R1747280:0-67	cdRNA03-Dia-R2	cdRNA05-postDia	7775.44	70631.9	3.18332	0.00015	0.0374403
RNA-Seq	Trans-ABySS	XLOC_009501	R1747280:0-67	cdRNA04-Dia-R3	cdRNA05-postDia	5374.22	70631.9	3.71619	5.00E-05	0.0195319
RNA-Seq	Trans-ABySS	XLOC_009509	R1747289:0-63	cdRNA02-Dia-R1	cdRNA05-postDia	11408.1	90695.1	2.99096	5.00E-05	0.0195319
RNA-Seq	Trans-ABySS	XLOC_009509	R1747289:0-63	cdRNA03-Dia-R2	cdRNA05-postDia	7919.23	90695.1	3.51759	0.00015	0.0374403
RNA-Seq	Trans-ABySS	XLOC_009509	R1747289:0-63	cdRNA04-Dia-R3	cdRNA05-postDia	5601.75	90695.1	4.01708	0.0002	0.0481888
RNA-Seq	Trans-ABySS	XLOC_009514	R1747297:0-355	cdRNA01-preDia	cdRNA05-postDia	18.0437	123.081	2.77004	5.00E-05	0.0195319
RNA-Seq	Trans-ABySS	XLOC_009522	R1747313:0-963	cdRNA02-Dia-R1	cdRNA04-Dia-R3	46.5575	4.27465	-3.44514	5.00E-05	0.0195319
RNA-Seq	Trans-ABySS	XLOC_009522	R1747313:0-963	cdRNA02-Dia-R1	cdRNA03-Dia-R2	46.5575	5.11312	-3.18674	5.00E-05	0.0195319
RNA-Seq	Trans-ABySS	XLOC_009523	R1747314:0-824	cdRNA04-Dia-R3	cdRNA05-postDia	137.503	18.3794	-2.9033	5.00E-05	0.0195319
RNA-Seq	Trans-ABySS	XLOC_009523	R1747314:0-824	cdRNA03-Dia-R2	cdRNA05-postDia	135.909	18.3794	-2.88648	5.00E-05	0.0195319
RNA-Seq	Trans-ABySS	XLOC_009524	R1747315:0-103	cdRNA04-Dia-R3	cdRNA05-postDia	1169.83	6586.72	2.49326	5.00E-05	0.0195319
RNA-Seq	Trans-ABySS	XLOC_009524	R1747315:0-103	cdRNA03-Dia-R2	cdRNA05-postDia	879.249	6586.72	2.90522	5.00E-05	0.0195319
RNA-Seq	Trans-ABySS	XLOC_009524	R1747315:0-103	cdRNA02-Dia-R1	cdRNA05-postDia	629.795	6586.72	3.38661	5.00E-05	0.0195319
RNA-Seq	Trans-ABySS	XLOC_009524	R1747315:0-103	cdRNA01-preDia	cdRNA05-postDia	613.952	6586.72	3.42336	5.00E-05	0.0195319
RNA-Seq	Trans-ABySS	XLOC_009532	R1747337:0-604	cdRNA04-Dia-R3	cdRNA05-postDia	119.851	18.179	-2.7209	5.00E-05	0.0195319
RNA-Seq	Trans-ABySS	XLOC_009532	R1747337:0-604	cdRNA03-Dia-R2	cdRNA05-postDia	110.96	18.179	-2.6097	5.00E-05	0.0195319
RNA-Seq	Trans-ABySS	XLOC_009540	R1747353:1-110	cdRNA01-preDia	cdRNA05-postDia	479.577	3679.83	2.9398	0.0001	0.0354705
RNA-Seq	Trans-ABySS	XLOC_009541	R1747355:0-500	cdRNA01-preDia	cdRNA04-Dia-R3	79.0445	7.35521	-3.42583	5.00E-05	0.0195319
RNA-Seq	Trans-ABySS	XLOC_009541	R1747355:0-500	cdRNA01-preDia	cdRNA03-Dia-R2	79.0445	7.38417	-3.42016	5.00E-05	0.0195319
RNA-Seq	Trans-ABySS	XLOC_009541	R1747355:0-500	cdRNA01-preDia	cdRNA02-Dia-R1	79.0445	9.53217	-3.05179	5.00E-05	0.0195319
RNA-Seq	Trans-ABySS	XLOC_009541	R1747355:0-500	cdRNA01-preDia	cdRNA05-postDia	79.0445	15.5784	-2.34312	0.00015	0.0374403
RNA-Seq	Trans-ABySS	XLOC_009542	R1747356:0-142	cdRNA03-Dia-R2	cdRNA05-postDia	312.579	2101.47	2.7491	5.00E-05	0.0195319
RNA-Seq	Trans-ABySS	XLOC_009542	R1747356:0-142	cdRNA02-Dia-R1	cdRNA05-postDia	299.572	2101.47	2.81042	5.00E-05	0.0195319
RNA-Seq	Trans-ABySS	XLOC_009542	R1747356:0-142	cdRNA01-preDia	cdRNA05-postDia	92.9748	2101.47	4.49841	5.00E-05	0.0195319
RNA-Seq	Trans-ABySS	XLOC_009546	R1747366:0-119	cdRNA01-preDia	cdRNA05-postDia	591.293	3621.41	2.61461	0.00015	0.0374403
RNA-Seq	Trans-ABySS	XLOC_009546	R1747366:0-119	cdRNA04-Dia-R3	cdRNA05-postDia	442.756	3621.41	3.03197	5.00E-05	0.0195319
RNA-Seq	Trans-ABySS	XLOC_009546	R1747366:0-119	cdRNA03-Dia-R2	cdRNA05-postDia	358.547	3621.41	3.33632	5.00E-05	0.0195319
RNA-Seq	Trans-ABySS	XLOC_009563	R1747394:0-822	cdRNA04-Dia-R3	cdRNA05-postDia	179.948	30.9945	-2.53749	0.00015	0.0374403
RNA-Seq	Trans-ABySS	XLOC_009586	R1747430:0-966	cdRNA01-preDia	cdRNA04-Dia-R3	20.3508	135.41	2.73417	5.00E-05	0.0195319
RNA-Seq	Trans-ABySS	XLOC_009586	R1747430:0-966	cdRNA01-preDia	cdRNA03-Dia-R2	20.3508	136.574	2.74653	5.00E-05	0.0195319
RNA-Seq	Trans-ABySS	XLOC_009586	R1747430:0-966	cdRNA01-preDia	cdRNA05-postDia	20.3508	178.406	3.132	5.00E-05	0.0195319
RNA-Seq	Trans-ABySS	XLOC_009611	R1747467:3-425	cdRNA04-Dia-R3	cdRNA05-postDia	32.1126	162.699	2.34099	0.0001	0.0354705
RNA-Seq	Trans-ABySS	XLOC_009611	R1747467:3-425	cdRNA03-Dia-R2	cdRNA05-postDia	29.8721	162.699	2.44534	0.00015	0.0374403
RNA-Seq	Trans-ABySS	XLOC_009611	R1747467:3-425	cdRNA01-preDia	cdRNA05-postDia	16.9452	162.699	3.26326	5.00E-05	0.0195319
RNA-Seq	Trans-ABySS	XLOC_009633	R1747509:0-258	cdRNA01-preDia	cdRNA03-Dia-R2	69.7001	440.686	2.66052	5.00E-05	0.0195319
RNA-Seq	Trans-ABySS	XLOC_009633	R1747509:0-258	cdRNA01-preDia	cdRNA04-Dia-R3	69.7001	456.011	2.70984	5.00E-05	0.0195319
RNA-Seq	Trans-ABySS	XLOC_009649	R1747533:10-960	cdRNA01-preDia	cdRNA05-postDia	422.776	45.1717	-3.2264	5.00E-05	0.0195319
RNA-Seq	Trans-ABySS	XLOC_009650	R1747534:0-63	cdRNA01-preDia	cdRNA05-postDia	54567.7	286460	2.39221	0.0002	0.0481888
RNA-Seq	Trans-ABySS	XLOC_009651	R1747537:0-590	cdRNA02-Dia-R1	cdRNA05-postDia	16.882	96.8333	2.52002	0.00015	0.0374403
RNA-Seq	Trans-ABySS	XLOC_009651	R1747537:0-590	cdRNA04-Dia-R3	cdRNA05-postDia	3.42843	96.8333	4.81988	5.00E-05	0.0195319

RNA-Seq	Trans-ABySS	XLOC_009651	R1747537:0-590	cdRNA03-Dia-R2	cdRNA05-postDia	2.77348	96.8333	5.12573	5.00E-05	0.0195319
RNA-Seq	Trans-ABySS	XLOC_009669	R1747576:0-657	cdRNA04-Dia-R3	cdRNA05-postDia	42.407	6.75363	-2.65057	5.00E-05	0.0195319
RNA-Seq	Trans-ABySS	XLOC_009669	R1747576:0-657	cdRNA03-Dia-R2	cdRNA05-postDia	39.1912	6.75363	-2.53679	5.00E-05	0.0195319
RNA-Seq	Trans-ABySS	XLOC_009708	R1747653:0-185	cdRNA01-preDia	cdRNA05-postDia	264.115	1450.8	2.45761	0.0001	0.0354705
RNA-Seq	Trans-ABySS	XLOC_009708	R1747653:0-185	cdRNA02-Dia-R1	cdRNA05-postDia	215.072	1450.8	2.75395	5.00E-05	0.0195319
RNA-Seq	Trans-ABySS	XLOC_009750	R1747725:0-1008	cdRNA01-preDia	cdRNA03-Dia-R2	24.7904	3.107	-2.99619	5.00E-05	0.0195319
RNA-Seq	Trans-ABySS	XLOC_009750	R1747725:0-1008	cdRNA01-preDia	cdRNA04-Dia-R3	24.7904	4.14159	-2.58152	5.00E-05	0.0195319
RNA-Seq	Trans-ABySS	XLOC_009750	R1747725:0-1008	cdRNA01-preDia	cdRNA05-postDia	24.7904	251.258	3.34132	5.00E-05	0.0195319
RNA-Seq	Trans-ABySS	XLOC_009750	R1747725:0-1008	cdRNA02-Dia-R1	cdRNA05-postDia	11.6982	251.258	4.42481	5.00E-05	0.0195319
RNA-Seq	Trans-ABySS	XLOC_009750	R1747725:0-1008	cdRNA04-Dia-R3	cdRNA05-postDia	4.14159	251.258	5.92284	5.00E-05	0.0195319
RNA-Seq	Trans-ABySS	XLOC_009750	R1747725:0-1008	cdRNA03-Dia-R2	cdRNA05-postDia	3.107	251.258	6.3375	5.00E-05	0.0195319
RNA-Seq	Trans-ABySS	XLOC_009760	R1747736:0-872	cdRNA01-preDia	cdRNA02-Dia-R1	28.3353	169.259	2.57856	0.0002	0.0481888
RNA-Seq	Trans-ABySS	XLOC_009760	R1747736:0-872	cdRNA01-preDia	cdRNA05-postDia	28.3353	253.757	3.16278	5.00E-05	0.0195319
RNA-Seq	Trans-ABySS	XLOC_009763	R1747741:0-303	cdRNA01-preDia	cdRNA02-Dia-R1	12.8982	94.9921	2.88064	0.0001	0.0354705
RNA-Seq	Trans-ABySS	XLOC_009763	R1747741:0-303	cdRNA03-Dia-R2	cdRNA05-postDia	20.0097	182.032	3.18542	5.00E-05	0.0195319
RNA-Seq	Trans-ABySS	XLOC_009763	R1747741:0-303	cdRNA04-Dia-R3	cdRNA05-postDia	18.7333	182.032	3.28051	5.00E-05	0.0195319
RNA-Seq	Trans-ABySS	XLOC_009763	R1747741:0-303	cdRNA01-preDia	cdRNA05-postDia	12.8982	182.032	3.81895	5.00E-05	0.0195319
RNA-Seq	Trans-ABySS	XLOC_009766	R1747745:0-3122	cdRNA01-preDia	cdRNA05-postDia	38.0138	358.672	3.23807	0.0002	0.0481888
RNA-Seq	Trans-ABySS	XLOC_009766	R1747745:0-3122	cdRNA03-Dia-R2	cdRNA05-postDia	15.5624	358.672	4.52653	5.00E-05	0.0195319
RNA-Seq	Trans-ABySS	XLOC_009766	R1747745:0-3122	cdRNA04-Dia-R3	cdRNA05-postDia	15.3178	358.672	4.54939	5.00E-05	0.0195319
RNA-Seq	Trans-ABySS	XLOC_009773	R1747756:0-148	cdRNA04-Dia-R3	cdRNA05-postDia	430.653	2428.84	2.49567	0.0001	0.0354705
RNA-Seq	Trans-ABySS	XLOC_009773	R1747756:0-148	cdRNA03-Dia-R2	cdRNA05-postDia	416.125	2428.84	2.54518	5.00E-05	0.0195319
RNA-Seq	Trans-ABySS	XLOC_009923	R1748043:0-658	cdRNA01-preDia	cdRNA05-postDia	67.7106	371.022	2.45405	5.00E-05	0.0195319
RNA-Seq	Trans-ABySS	XLOC_009923	R1748043:0-658	cdRNA04-Dia-R3	cdRNA05-postDia	66.9495	371.022	2.47036	5.00E-05	0.0195319
RNA-Seq	Trans-ABySS	XLOC_009923	R1748043:0-658	cdRNA03-Dia-R2	cdRNA05-postDia	61.6049	371.022	2.59039	5.00E-05	0.0195319
RNA-Seq	Trans-ABySS	XLOC_009967	R1748114:0-364	cdRNA01-preDia	cdRNA05-postDia	22.7384	134.289	2.56214	5.00E-05	0.0195319
RNA-Seq	Trans-ABySS	XLOC_009969	R1748117:0-1424	cdRNA01-preDia	cdRNA05-postDia	50.4327	4.83377	-3.38314	5.00E-05	0.0195319
RNA-Seq	Trans-ABySS	XLOC_009969	R1748117:0-1424	cdRNA01-preDia	cdRNA04-Dia-R3	50.4327	6.15863	-3.03368	5.00E-05	0.0195319
RNA-Seq	Trans-ABySS	XLOC_009969	R1748117:0-1424	cdRNA01-preDia	cdRNA03-Dia-R2	50.4327	6.58149	-2.93787	5.00E-05	0.0195319
RNA-Seq	Trans-ABySS	XLOC_010029	R1748223:0-61	cdRNA03-Dia-R2	cdRNA05-postDia	8156.57	81976.5	3.32918	0.0002	0.0481888
RNA-Seq	Trans-ABySS	XLOC_010058	R1748275:0-153	cdRNA03-Dia-R2	cdRNA05-postDia	200.135	1439.09	2.84611	0.0002	0.0481888
RNA-Seq	Trans-ABySS	XLOC_010058	R1748275:0-153	cdRNA04-Dia-R3	cdRNA05-postDia	187.458	1439.09	2.94052	0.0002	0.0481888
RNA-Seq	Trans-ABySS	XLOC_010058	R1748275:0-153	cdRNA02-Dia-R1	cdRNA05-postDia	169.945	1439.09	3.08202	5.00E-05	0.0195319
RNA-Seq	Trans-ABySS	XLOC_010058	R1748275:0-153	cdRNA01-preDia	cdRNA05-postDia	130.616	1439.09	3.46175	5.00E-05	0.0195319
RNA-Seq	Trans-ABySS	XLOC_010105	R1748351:1-96	cdRNA02-Dia-R1	cdRNA05-postDia	722.273	9933.87	3.78174	5.00E-05	0.0195319
RNA-Seq	Trans-ABySS	XLOC_010105	R1748351:1-96	cdRNA01-preDia	cdRNA05-postDia	506.176	9933.87	4.29464	5.00E-05	0.0195319
RNA-Seq	Trans-ABySS	XLOC_010145	R1748439:1-154	cdRNA03-Dia-R2	cdRNA05-postDia	1013.45	131.211	-2.94932	0.00015	0.0374403
RNA-Seq	Trans-ABySS	XLOC_010145	R1748439:1-154	cdRNA02-Dia-R1	cdRNA05-postDia	830.486	131.211	-2.66207	0.00015	0.0374403
RNA-Seq	Trans-ABySS	XLOC_010147	R1748442:0-373	cdRNA01-preDia	cdRNA05-postDia	17.7783	94.7958	2.41471	0.00015	0.0374403
RNA-Seq	Trans-ABySS	XLOC_010163	R1748473:0-108	cdRNA01-preDia	cdRNA05-postDia	320.797	10204.9	4.99145	5.00E-05	0.0195319
RNA-Seq	Trans-ABySS	XLOC_010202	R1748528:2-638	cdRNA01-preDia	cdRNA03-Dia-R2	3.53203	28.3191	3.0032	5.00E-05	0.0195319
RNA-Seq	Trans-ABySS	XLOC_010202	R1748528:2-638	cdRNA01-preDia	cdRNA04-Dia-R3	3.53203	31.5194	3.15767	5.00E-05	0.0195319
RNA-Seq	Trans-ABySS	XLOC_010256	R1748646:0-1040	cdRNA04-Dia-R3	cdRNA05-postDia	142.931	17.9268	-2.99513	5.00E-05	0.0195319
RNA-Seq	Trans-ABySS	XLOC_010256	R1748646:0-1040	cdRNA03-Dia-R2	cdRNA05-postDia	132.702	17.9268	-2.88801	5.00E-05	0.0195319
RNA-Seq	Trans-ABySS	XLOC_010274	R1748683:0-1527	cdRNA01-preDia	cdRNA03-Dia-R2	11.2732	86.5859	2.94123	0.00015	0.0374403
RNA-Seq	Trans-ABySS	XLOC_010274	R1748683:0-1527	cdRNA01-preDia	cdRNA04-Dia-R3	11.2732	84.7034	2.90952	5.00E-05	0.0195319
RNA-Seq	Trans-ABySS	XLOC_010300	R1748733:0-1292	cdRNA04-Dia-R3	cdRNA05-postDia	60.8967	9.25105	-2.71867	5.00E-05	0.0195319

RNA-Seq	Trans-ABySS	XLOC_010300	R1748733:0-1292	cdRNA03-Dia-R2	cdRNA05-postDia	58.6708	9.25105	-2.66495	5.00E-05	0.0195319
RNA-Seq	Trans-ABySS	XLOC_010323	R1748780:0-511	cdRNA01-preDia	cdRNA03-Dia-R2	20.5016	100.837	2.29822	0.0002	0.0481888
RNA-Seq	Trans-ABySS	XLOC_010349	R1748833:0-387	cdRNA02-Dia-R1	cdRNA05-postDia	10.0862	174.775	4.11504	5.00E-05	0.0195319
RNA-Seq	Trans-ABySS	XLOC_010349	R1748833:0-387	cdRNA04-Dia-R3	cdRNA05-postDia	3.96212	174.775	5.46308	5.00E-05	0.0195319
RNA-Seq	Trans-ABySS	XLOC_010397	R1748920:0-480	cdRNA02-Dia-R1	cdRNA05-postDia	20.0806	103.295	2.3629	0.00015	0.0374403
RNA-Seq	Trans-ABySS	XLOC_010397	R1748920:0-480	cdRNA04-Dia-R3	cdRNA05-postDia	19.0244	103.295	2.44085	5.00E-05	0.0195319
RNA-Seq	Trans-ABySS	XLOC_010433	R1748984:1-1808	cdRNA01-preDia	cdRNA04-Dia-R3	7.90993	55.7135	2.81629	5.00E-05	0.0195319
RNA-Seq	Trans-ABySS	XLOC_010433	R1748984:1-1808	cdRNA01-preDia	cdRNA03-Dia-R2	7.90993	56.9079	2.84689	5.00E-05	0.0195319
RNA-Seq	Trans-ABySS	XLOC_010453	R1749018:0-237	cdRNA02-Dia-R1	cdRNA05-postDia	27.0579	437.529	4.01525	5.00E-05	0.0195319
RNA-Seq	Trans-ABySS	XLOC_010453	R1749018:0-237	cdRNA04-Dia-R3	cdRNA05-postDia	13.7552	437.529	4.99133	0.0002	0.0481888
RNA-Seq	Trans-ABySS	XLOC_010479	R1749063:3-262	cdRNA03-Dia-R2	cdRNA05-postDia	11.569	221.195	4.25699	0.0001	0.0354705
RNA-Seq	Trans-ABySS	XLOC_010490	R1749077:0-2050	cdRNA01-preDia	cdRNA04-Dia-R3	9.36417	63.3884	2.759	0.00015	0.0374403
RNA-Seq	Trans-ABySS	XLOC_010490	R1749077:0-2050	cdRNA01-preDia	cdRNA03-Dia-R2	9.36417	64.6164	2.78668	0.0002	0.0481888
RNA-Seq	Trans-ABySS	XLOC_010501	R1749094:0-2359	cdRNA03-Dia-R2	cdRNA05-postDia	17.5741	121.292	2.78696	0.0002	0.0481888
RNA-Seq	Trans-ABySS	XLOC_010501	R1749094:0-2359	cdRNA04-Dia-R3	cdRNA05-postDia	17.2149	121.292	2.81675	0.00015	0.0374403
RNA-Seq	Trans-ABySS	XLOC_010554	R1749191:0-1661	cdRNA01-preDia	cdRNA05-postDia	8.3895	43.64	2.37899	5.00E-05	0.0195319
RNA-Seq	Trans-ABySS	XLOC_010554	R1749191:0-1661	cdRNA01-preDia	cdRNA04-Dia-R3	8.3895	73.075	3.12272	5.00E-05	0.0195319
RNA-Seq	Trans-ABySS	XLOC_010554	R1749191:0-1661	cdRNA01-preDia	cdRNA03-Dia-R2	8.3895	73.8465	3.13787	5.00E-05	0.0195319
RNA-Seq	Trans-ABySS	XLOC_010607	R1749305:0-1394	cdRNA04-Dia-R3	cdRNA05-postDia	29.7398	156.978	2.40009	0.0002	0.0481888
RNA-Seq	Trans-ABySS	XLOC_010607	R1749305:0-1394	cdRNA03-Dia-R2	cdRNA05-postDia	28.2267	156.978	2.47543	0.0001	0.0354705
RNA-Seq	Trans-ABySS	XLOC_010607	R1749305:0-1394	cdRNA02-Dia-R1	cdRNA05-postDia	25.4329	156.978	2.62579	5.00E-05	0.0195319
RNA-Seq	Trans-ABySS	XLOC_010631	R1749349:0-596	cdRNA02-Dia-R1	cdRNA05-postDia	20.9937	137.88	2.71539	5.00E-05	0.0195319
RNA-Seq	Trans-ABySS	XLOC_010631	R1749349:0-596	cdRNA03-Dia-R2	cdRNA05-postDia	4.43793	137.88	4.95739	5.00E-05	0.0195319
RNA-Seq	Trans-ABySS	XLOC_010631	R1749349:0-596	cdRNA01-preDia	cdRNA05-postDia	3.83953	137.88	5.16634	5.00E-05	0.0195319
RNA-Seq	Trans-ABySS	XLOC_010631	R1749349:0-596	cdRNA04-Dia-R3	cdRNA05-postDia	3.37596	137.88	5.35198	5.00E-05	0.0195319
RNA-Seq	Trans-ABySS	XLOC_010642	R1749366:0-1718	cdRNA02-Dia-R1	cdRNA05-postDia	18.5703	195.84	3.3986	5.00E-05	0.0195319
RNA-Seq	Trans-ABySS	XLOC_010642	R1749366:0-1718	cdRNA01-preDia	cdRNA05-postDia	8.43516	195.84	4.53711	5.00E-05	0.0195319
RNA-Seq	Trans-ABySS	XLOC_010642	R1749366:0-1718	cdRNA04-Dia-R3	cdRNA05-postDia	7.50046	195.84	4.70655	5.00E-05	0.0195319
RNA-Seq	Trans-ABySS	XLOC_010642	R1749366:0-1718	cdRNA03-Dia-R2	cdRNA05-postDia	7.39538	195.84	4.72691	5.00E-05	0.0195319
RNA-Seq	Trans-ABySS	XLOC_010685	R1749432:6-1721	cdRNA01-preDia	cdRNA05-postDia	52.6735	9.80116	-2.42605	0.00015	0.0374403
RNA-Seq	Trans-ABySS	XLOC_010685	R1749432:6-1721	cdRNA03-Dia-R2	cdRNA05-postDia	52.6085	9.80116	-2.42427	0.0001	0.0354705
RNA-Seq	Trans-ABySS	XLOC_010695	R1749455:0-1247	cdRNA01-preDia	cdRNA03-Dia-R2	12.7253	80.9185	2.66877	0.0001	0.0354705
RNA-Seq	Trans-ABySS	XLOC_010695	R1749455:0-1247	cdRNA01-preDia	cdRNA04-Dia-R3	12.7253	85.0534	2.74067	5.00E-05	0.0195319
RNA-Seq	Trans-ABySS	XLOC_010802	R1749653:0-972	cdRNA04-Dia-R3	cdRNA05-postDia	5.72538	28.5783	2.31947	0.00015	0.0374403
RNA-Seq	Trans-ABySS	XLOC_010802	R1749653:0-972	cdRNA03-Dia-R2	cdRNA05-postDia	5.4017	28.5783	2.40343	0.00015	0.0374403
RNA-Seq	Trans-ABySS	XLOC_010828	R1749717:1-344	cdRNA04-Dia-R3	cdRNA05-postDia	22.5608	120.012	2.41129	0.0002	0.0481888
RNA-Seq	Trans-ABySS	XLOC_010835	R1749726:1-1091	cdRNA02-Dia-R1	cdRNA05-postDia	24.0745	195.325	3.0203	5.00E-05	0.0195319
RNA-Seq	Trans-ABySS	XLOC_010835	R1749726:1-1091	cdRNA03-Dia-R2	cdRNA05-postDia	10.5729	195.325	4.20743	5.00E-05	0.0195319
RNA-Seq	Trans-ABySS	XLOC_010835	R1749726:1-1091	cdRNA01-preDia	cdRNA05-postDia	10.0898	195.325	4.27491	5.00E-05	0.0195319
RNA-Seq	Trans-ABySS	XLOC_010835	R1749726:1-1091	cdRNA04-Dia-R3	cdRNA05-postDia	9.82937	195.325	4.31263	5.00E-05	0.0195319
RNA-Seq	Trans-ABySS	XLOC_010843	R1749742:0-755	cdRNA01-preDia	cdRNA05-postDia	58.4397	326.228	2.48086	0.00015	0.0374403
RNA-Seq	Trans-ABySS	XLOC_010843	R1749742:0-755	cdRNA04-Dia-R3	cdRNA05-postDia	28.2792	326.228	3.52807	5.00E-05	0.0195319
RNA-Seq	Trans-ABySS	XLOC_010843	R1749742:0-755	cdRNA03-Dia-R2	cdRNA05-postDia	28.0111	326.228	3.54181	5.00E-05	0.0195319
RNA-Seq	Trans-ABySS	XLOC_010843	R1749742:0-755	cdRNA02-Dia-R1	cdRNA05-postDia	24.0801	326.228	3.75997	5.00E-05	0.0195319
RNA-Seq	Trans-ABySS	XLOC_010861	R1749773:2-618	cdRNA01-preDia	cdRNA03-Dia-R2	165.056	30.0989	-2.45518	0.0002	0.0481888
RNA-Seq	Trans-ABySS	XLOC_010867	R1749785:3-190	cdRNA02-Dia-R1	cdRNA05-postDia	108.013	1572.38	3.86367	5.00E-05	0.0195319
RNA-Seq	Trans-ABySS	XLOC_010889	R1749827:0-114	cdRNA02-Dia-R1	cdRNA05-postDia	594.002	4690.76	2.98128	5.00E-05	0.0195319

RNA-Seq	Trans-ABySS	XLOC_010889	R1749827:0-114	cdRNA04-Dia-R3	cdRNA05-postDia	503.28	4690.76	3.22039	5.00E-05	0.0195319
RNA-Seq	Trans-ABySS	XLOC_010889	R1749827:0-114	cdRNA03-Dia-R2	cdRNA05-postDia	429.011	4690.76	3.45074	5.00E-05	0.0195319
RNA-Seq	Trans-ABySS	XLOC_010924	R1749888:0-551	cdRNA02-Dia-R1	cdRNA05-postDia	13.7457	195.627	3.83106	5.00E-05	0.0195319
RNA-Seq	Trans-ABySS	XLOC_010924	R1749888:0-551	cdRNA01-preDia	cdRNA05-postDia	4.206	195.627	5.53951	5.00E-05	0.0195319
RNA-Seq	Trans-ABySS	XLOC_010924	R1749888:0-551	cdRNA04-Dia-R3	cdRNA05-postDia	3.19863	195.627	5.93451	5.00E-05	0.0195319
RNA-Seq	Trans-ABySS	XLOC_010924	R1749888:0-551	cdRNA03-Dia-R2	cdRNA05-postDia	2.82809	195.627	6.11213	5.00E-05	0.0195319
RNA-Seq	Trans-ABySS	XLOC_010975	R1749982:2-541	cdRNA02-Dia-R1	cdRNA04-Dia-R3	5.9158	34.1514	2.5293	0.0002	0.0481888
RNA-Seq	Trans-ABySS	XLOC_010975	R1749982:2-541	cdRNA02-Dia-R1	cdRNA03-Dia-R2	5.9158	38.3484	2.69652	0.00015	0.0374403
RNA-Seq	Trans-ABySS	XLOC_010975	R1749982:2-541	cdRNA01-preDia	cdRNA04-Dia-R3	3.81207	34.1514	3.1633	5.00E-05	0.0195319
RNA-Seq	Trans-ABySS	XLOC_010975	R1749982:2-541	cdRNA01-preDia	cdRNA03-Dia-R2	3.81207	38.3484	3.33052	5.00E-05	0.0195319
RNA-Seq	Trans-ABySS	XLOC_010990	R1750012:0-449	cdRNA03-Dia-R2	cdRNA05-postDia	46.4267	9.22954	-2.33062	0.0002	0.0481888
RNA-Seq	Trans-ABySS	XLOC_011003	R1750036:2-508	cdRNA01-preDia	cdRNA05-postDia	6.947	67.3886	3.27804	5.00E-05	0.0195319
RNA-Seq	Trans-ABySS	XLOC_011030	R1750096:1-221	cdRNA02-Dia-R1	cdRNA05-postDia	81.2154	534.884	2.7194	5.00E-05	0.0195319
RNA-Seq	Trans-ABySS	XLOC_011030	R1750096:1-221	cdRNA01-preDia	cdRNA05-postDia	34.0212	534.884	3.97472	5.00E-05	0.0195319
RNA-Seq	Trans-ABySS	XLOC_011030	R1750096:1-221	cdRNA03-Dia-R2	cdRNA05-postDia	29.8316	534.884	4.16431	5.00E-05	0.0195319
RNA-Seq	Trans-ABySS	XLOC_011030	R1750096:1-221	cdRNA04-Dia-R3	cdRNA05-postDia	23.0591	534.884	4.53582	5.00E-05	0.0195319
RNA-Seq	Trans-ABySS	XLOC_011060	R1750156:0-425	cdRNA03-Dia-R2	cdRNA05-postDia	146.326	13.2407	-3.46614	5.00E-05	0.0195319
RNA-Seq	Trans-ABySS	XLOC_011060	R1750156:0-425	cdRNA04-Dia-R3	cdRNA05-postDia	134.628	13.2407	-3.34593	5.00E-05	0.0195319
RNA-Seq	Trans-ABySS	XLOC_011060	R1750156:0-425	cdRNA02-Dia-R1	cdRNA04-Dia-R3	23.2532	134.628	2.53348	5.00E-05	0.0195319
RNA-Seq	Trans-ABySS	XLOC_011060	R1750156:0-425	cdRNA02-Dia-R1	cdRNA03-Dia-R2	23.2532	146.326	2.65369	5.00E-05	0.0195319
RNA-Seq	Trans-ABySS	XLOC_011060	R1750156:0-425	cdRNA01-preDia	cdRNA04-Dia-R3	11.7627	134.628	3.51669	5.00E-05	0.0195319
RNA-Seq	Trans-ABySS	XLOC_011060	R1750156:0-425	cdRNA01-preDia	cdRNA03-Dia-R2	11.7627	146.326	3.6369	5.00E-05	0.0195319
RNA-Seq	Trans-ABySS	XLOC_011063	R1750159:0-103	cdRNA02-Dia-R1	cdRNA05-postDia	920.47	6288.29	2.77223	5.00E-05	0.0195319
RNA-Seq	Trans-ABySS	XLOC_011063	R1750159:0-103	cdRNA03-Dia-R2	cdRNA05-postDia	557.572	6288.29	3.49544	5.00E-05	0.0195319
RNA-Seq	Trans-ABySS	XLOC_011063	R1750159:0-103	cdRNA04-Dia-R3	cdRNA05-postDia	513.083	6288.29	3.6154	0.0002	0.0481888
RNA-Seq	Trans-ABySS	XLOC_011127	R1750281:0-1820	cdRNA01-preDia	cdRNA05-postDia	7.47938	47.8246	2.67676	5.00E-05	0.0195319
RNA-Seq	Trans-ABySS	XLOC_011164	R1750338:0-1534	cdRNA02-Dia-R1	cdRNA05-postDia	28.8678	182.96	2.664	0.00015	0.0374403
RNA-Seq	Trans-ABySS	XLOC_011164	R1750338:0-1534	cdRNA03-Dia-R2	cdRNA05-postDia	22.0692	182.96	3.05143	5.00E-05	0.0195319
RNA-Seq	Trans-ABySS	XLOC_011164	R1750338:0-1534	cdRNA04-Dia-R3	cdRNA05-postDia	20.7675	182.96	3.13913	5.00E-05	0.0195319
RNA-Seq	Trans-ABySS	XLOC_011168	R1750344:0-388	cdRNA01-preDia	cdRNA02-Dia-R1	134.482	23.1646	-2.53741	0.00015	0.0374403
RNA-Seq	Trans-ABySS	XLOC_011183	R1750373:0-360	cdRNA04-Dia-R3	cdRNA05-postDia	29.4513	152.946	2.37662	5.00E-05	0.0195319
RNA-Seq	Trans-ABySS	XLOC_011183	R1750373:0-360	cdRNA03-Dia-R2	cdRNA05-postDia	29.3494	152.946	2.38162	0.0002	0.0481888
RNA-Seq	Trans-ABySS	XLOC_011183	R1750373:0-360	cdRNA02-Dia-R1	cdRNA05-postDia	22.1007	152.946	2.79086	5.00E-05	0.0195319
RNA-Seq	Trans-ABySS	XLOC_011294	R1750578:0-2213	cdRNA04-Dia-R3	cdRNA05-postDia	10.7524	54.1394	2.33203	0.00015	0.0374403
RNA-Seq	Trans-ABySS	XLOC_011294	R1750578:0-2213	cdRNA01-preDia	cdRNA05-postDia	10.3298	54.1394	2.38986	0.00015	0.0374403
RNA-Seq	Trans-ABySS	XLOC_011323	R1750629:9-572	cdRNA02-Dia-R1	cdRNA03-Dia-R2	35.9289	5.71563	-2.65216	5.00E-05	0.0195319
RNA-Seq	Trans-ABySS	XLOC_011323	R1750629:9-572	cdRNA03-Dia-R2	cdRNA05-postDia	5.71563	33.2232	2.53921	0.0002	0.0481888
RNA-Seq	Trans-ABySS	XLOC_011323	R1750629:9-572	cdRNA04-Dia-R3	cdRNA05-postDia	2.9728	33.2232	3.4823	0.0002	0.0481888
RNA-Seq	Trans-ABySS	XLOC_011329	R1750636:0-1333	cdRNA01-preDia	cdRNA05-postDia	3.35308	16.8484	2.32905	5.00E-05	0.0195319
RNA-Seq	Trans-ABySS	XLOC_011362	R1750691:0-654	cdRNA02-Dia-R1	cdRNA05-postDia	18.064	131.638	2.86539	5.00E-05	0.0195319
RNA-Seq	Trans-ABySS	XLOC_011362	R1750691:0-654	cdRNA01-preDia	cdRNA05-postDia	6.48664	131.638	4.34296	5.00E-05	0.0195319
RNA-Seq	Trans-ABySS	XLOC_011362	R1750691:0-654	cdRNA03-Dia-R2	cdRNA05-postDia	5.5511	131.638	4.56766	5.00E-05	0.0195319
RNA-Seq	Trans-ABySS	XLOC_011362	R1750691:0-654	cdRNA04-Dia-R3	cdRNA05-postDia	3.51004	131.638	5.22894	5.00E-05	0.0195319
RNA-Seq	Trans-ABySS	XLOC_011376	R1750726:0-516	cdRNA04-Dia-R3	cdRNA05-postDia	7.51575	40.4997	2.42992	0.0002	0.0481888
RNA-Seq	Trans-ABySS	XLOC_011376	R1750726:0-516	cdRNA01-preDia	cdRNA05-postDia	7.35713	40.4997	2.4607	0.0002	0.0481888
RNA-Seq	Trans-ABySS	XLOC_011388	R1750745:0-494	cdRNA01-preDia	cdRNA04-Dia-R3	25.7924	136.752	2.40655	0.0002	0.0481888
RNA-Seq	Trans-ABySS	XLOC_011388	R1750745:0-494	cdRNA01-preDia	cdRNA03-Dia-R2	25.7924	140.587	2.44645	0.0001	0.0354705

RNA-Seq	Trans-ABySS	XLOC_011402	R1750775:1-501	cdRNA02-Dia-R1	cdRNA05-postDia	8.17043	48.982	2.58377	5.00E-05	0.0195319
RNA-Seq	Trans-ABySS	XLOC_011402	R1750775:1-501	cdRNA03-Dia-R2	cdRNA05-postDia	7.68556	48.982	2.67203	0.0001	0.0354705
RNA-Seq	Trans-ABySS	XLOC_011402	R1750775:1-501	cdRNA01-preDia	cdRNA05-postDia	2.31124	48.982	4.40551	0.0001	0.0354705
RNA-Seq	Trans-ABySS	XLOC_011422	R1750820:0-1422	cdRNA03-Dia-R2	cdRNA05-postDia	129.246	14.962	-3.11074	5.00E-05	0.0195319
RNA-Seq	Trans-ABySS	XLOC_011422	R1750820:0-1422	cdRNA04-Dia-R3	cdRNA05-postDia	124.812	14.962	-3.06038	5.00E-05	0.0195319
RNA-Seq	Trans-ABySS	XLOC_011422	R1750820:0-1422	cdRNA02-Dia-R1	cdRNA03-Dia-R2	20.7391	129.246	2.63969	0.00015	0.0374403
RNA-Seq	Trans-ABySS	XLOC_011422	R1750820:0-1422	cdRNA01-preDia	cdRNA04-Dia-R3	10.2229	124.812	3.60987	5.00E-05	0.0195319
RNA-Seq	Trans-ABySS	XLOC_011422	R1750820:0-1422	cdRNA01-preDia	cdRNA03-Dia-R2	10.2229	129.246	3.66023	5.00E-05	0.0195319
RNA-Seq	Trans-ABySS	XLOC_011454	R1750880:0-1607	cdRNA01-preDia	cdRNA05-postDia	8.54986	84.5398	3.30566	5.00E-05	0.0195319
RNA-Seq	Trans-ABySS	XLOC_011548	R1751061:0-717	cdRNA01-preDia	cdRNA05-postDia	7.11257	41.1413	2.53214	0.0001	0.0354705
RNA-Seq	Trans-ABySS	XLOC_011703	R1751348:0-824	cdRNA01-preDia	cdRNA05-postDia	14.8599	88.8219	2.57949	5.00E-05	0.0195319
RNA-Seq	Trans-ABySS	XLOC_011827	R1751594:0-408	cdRNA03-Dia-R2	cdRNA05-postDia	132.477	21.9469	-2.59366	5.00E-05	0.0195319
RNA-Seq	Trans-ABySS	XLOC_011827	R1751594:0-408	cdRNA04-Dia-R3	cdRNA05-postDia	128.038	21.9469	-2.54449	5.00E-05	0.0195319
RNA-Seq	Trans-ABySS	XLOC_011827	R1751594:0-408	cdRNA01-preDia	cdRNA03-Dia-R2	26.3754	132.477	2.32848	0.0002	0.0481888
RNA-Seq	Trans-ABySS	XLOC_011837	R1751609:0-525	cdRNA04-Dia-R3	cdRNA05-postDia	23.9379	162.022	2.75882	5.00E-05	0.0195319
RNA-Seq	Trans-ABySS	XLOC_011837	R1751609:0-525	cdRNA03-Dia-R2	cdRNA05-postDia	21.539	162.022	2.91117	5.00E-05	0.0195319
RNA-Seq	Trans-ABySS	XLOC_011912	R1751756:0-1038	cdRNA02-Dia-R1	cdRNA05-postDia	14.7566	116.309	2.97853	5.00E-05	0.0195319
RNA-Seq	Trans-ABySS	XLOC_011912	R1751756:0-1038	cdRNA04-Dia-R3	cdRNA05-postDia	13.9235	116.309	3.06237	5.00E-05	0.0195319
RNA-Seq	Trans-ABySS	XLOC_011912	R1751756:0-1038	cdRNA03-Dia-R2	cdRNA05-postDia	12.5416	116.309	3.21317	5.00E-05	0.0195319
RNA-Seq	Trans-ABySS	XLOC_011912	R1751756:0-1038	cdRNA01-preDia	cdRNA05-postDia	4.50355	116.309	4.69076	5.00E-05	0.0195319
RNA-Seq	Trans-ABySS	XLOC_011942	R1751811:2-320	cdRNA03-Dia-R2	cdRNA05-postDia	33.7529	199.394	2.56254	0.0001	0.0354705
RNA-Seq	Trans-ABySS	XLOC_011955	R1751833:0-737	cdRNA01-preDia	cdRNA03-Dia-R2	15.8307	87.1223	2.46032	5.00E-05	0.0195319
RNA-Seq	Trans-ABySS	XLOC_011955	R1751833:0-737	cdRNA01-preDia	cdRNA04-Dia-R3	15.8307	88.6637	2.48562	5.00E-05	0.0195319
RNA-Seq	Trans-ABySS	XLOC_011978	R1751874:0-550	cdRNA01-preDia	cdRNA03-Dia-R2	18.9817	96.9487	2.35261	0.0001	0.0354705
RNA-Seq	Trans-ABySS	XLOC_011987	R1751888:0-2523	cdRNA01-preDia	cdRNA04-Dia-R3	6.94361	40.4126	2.54105	0.0002	0.0481888
RNA-Seq	Trans-ABySS	XLOC_011987	R1751888:0-2523	cdRNA01-preDia	cdRNA03-Dia-R2	6.94361	42.2688	2.60583	5.00E-05	0.0195319
RNA-Seq	Trans-ABySS	XLOC_012067	R1752054:0-1677	cdRNA01-preDia	cdRNA03-Dia-R2	87.0091	13.9608	-2.63978	0.0002	0.0481888
RNA-Seq	Trans-ABySS	XLOC_012373	R1752607:0-452	cdRNA02-Dia-R1	cdRNA05-postDia	17.2009	145.171	3.0772	5.00E-05	0.0195319
RNA-Seq	Trans-ABySS	XLOC_012373	R1752607:0-452	cdRNA03-Dia-R2	cdRNA05-postDia	10.4321	145.171	3.79866	5.00E-05	0.0195319
RNA-Seq	Trans-ABySS	XLOC_012373	R1752607:0-452	cdRNA04-Dia-R3	cdRNA05-postDia	9.46726	145.171	3.93867	5.00E-05	0.0195319
RNA-Seq	Trans-ABySS	XLOC_012373	R1752607:0-452	cdRNA01-preDia	cdRNA05-postDia	6.43663	145.171	4.49531	5.00E-05	0.0195319
RNA-Seq	Trans-ABySS	XLOC_012461	R1752793:3-317	cdRNA01-preDia	cdRNA05-postDia	21.875	122.475	2.48514	0.0002	0.0481888
RNA-Seq	Trans-ABySS	XLOC_012461	R1752793:3-317	cdRNA04-Dia-R3	cdRNA05-postDia	20.0956	122.475	2.60754	5.00E-05	0.0195319
RNA-Seq	Trans-ABySS	XLOC_012461	R1752793:3-317	cdRNA02-Dia-R1	cdRNA05-postDia	18.1988	122.475	2.75058	0.00015	0.0374403
RNA-Seq	Trans-ABySS	XLOC_012461	R1752793:3-317	cdRNA03-Dia-R2	cdRNA05-postDia	17.8286	122.475	2.78022	5.00E-05	0.0195319
RNA-Seq	Trans-ABySS	XLOC_012498	R1752861:0-834	cdRNA01-preDia	cdRNA03-Dia-R2	9.70108	50.1303	2.36946	0.00015	0.0374403
RNA-Seq	Trans-ABySS	XLOC_012498	R1752861:0-834	cdRNA01-preDia	cdRNA04-Dia-R3	9.70108	54.0063	2.47691	0.0001	0.0354705
RNA-Seq	Trans-ABySS	XLOC_012533	R1752917:1-687	cdRNA03-Dia-R2	cdRNA05-postDia	12.2161	82.239	2.75104	5.00E-05	0.0195319
RNA-Seq	Trans-ABySS	XLOC_012533	R1752917:1-687	cdRNA02-Dia-R1	cdRNA05-postDia	11.6381	82.239	2.82097	5.00E-05	0.0195319
RNA-Seq	Trans-ABySS	XLOC_012533	R1752917:1-687	cdRNA04-Dia-R3	cdRNA05-postDia	11.5138	82.239	2.83645	5.00E-05	0.0195319
RNA-Seq	Trans-ABySS	XLOC_012533	R1752917:1-687	cdRNA01-preDia	cdRNA05-postDia	9.27326	82.239	3.14868	5.00E-05	0.0195319
RNA-Seq	Trans-ABySS	XLOC_012573	R1752985:0-2502	cdRNA01-preDia	cdRNA02-Dia-R1	2.34204	13.638	2.5418	5.00E-05	0.0195319
RNA-Seq	Trans-ABySS	XLOC_012573	R1752985:0-2502	cdRNA01-preDia	cdRNA05-postDia	2.34204	23.6944	3.33871	5.00E-05	0.0195319
RNA-Seq	Trans-ABySS	XLOC_012576	R1752992:0-484	cdRNA01-preDia	cdRNA03-Dia-R2	4.72373	34.092	2.85143	5.00E-05	0.0195319
RNA-Seq	Trans-ABySS	XLOC_012576	R1752992:0-484	cdRNA01-preDia	cdRNA04-Dia-R3	4.72373	36.743	2.95947	5.00E-05	0.0195319
RNA-Seq	Trans-ABySS	XLOC_012606	R1753059:0-1567	cdRNA01-preDia	cdRNA04-Dia-R3	11.8124	63.2371	2.42046	0.0002	0.0481888
RNA-Seq	Trans-ABySS	XLOC_012606	R1753059:0-1567	cdRNA01-preDia	cdRNA03-Dia-R2	11.8124	64.8664	2.45717	0.0001	0.0354705

RNA-Seq	Trans-ABySS	XLOC_012810	R1753399:0-1253	cdRNA01-preDia	cdRNA05-postDia	8.34805	53.343	2.67579	5.00E-05	0.0195319
RNA-Seq	Trans-ABySS	XLOC_012812	R1753402:0-1157	cdRNA01-preDia	cdRNA03-Dia-R2	74.9476	10.8126	-2.79317	5.00E-05	0.0195319
RNA-Seq	Trans-ABySS	XLOC_012812	R1753402:0-1157	cdRNA01-preDia	cdRNA04-Dia-R3	74.9476	10.975	-2.77167	5.00E-05	0.0195319
RNA-Seq	Trans-ABySS	XLOC_012812	R1753402:0-1157	cdRNA04-Dia-R3	cdRNA05-postDia	10.975	70.1154	2.67552	5.00E-05	0.0195319
RNA-Seq	Trans-ABySS	XLOC_012812	R1753402:0-1157	cdRNA03-Dia-R2	cdRNA05-postDia	10.8126	70.1154	2.69702	5.00E-05	0.0195319
RNA-Seq	Trans-ABySS	XLOC_012876	R1753538:0-890	cdRNA04-Dia-R3	cdRNA05-postDia	43.4369	5.68787	-2.93296	5.00E-05	0.0195319
RNA-Seq	Trans-ABySS	XLOC_012876	R1753538:0-890	cdRNA03-Dia-R2	cdRNA05-postDia	41.2912	5.68787	-2.85988	5.00E-05	0.0195319
RNA-Seq	Trans-ABySS	XLOC_013012	R1753778:0-2015	cdRNA04-Dia-R3	cdRNA05-postDia	35.5716	7.34512	-2.27587	0.00015	0.0374403
RNA-Seq	Trans-ABySS	XLOC_013081	R1753913:0-369	cdRNA01-preDia	cdRNA04-Dia-R3	6.34308	70.2122	3.46846	0.0002	0.0481888
RNA-Seq	Trans-ABySS	XLOC_013081	R1753913:0-369	cdRNA01-preDia	cdRNA02-Dia-R1	6.34308	70.8853	3.48223	0.0002	0.0481888
RNA-Seq	Trans-ABySS	XLOC_013081	R1753913:0-369	cdRNA01-preDia	cdRNA03-Dia-R2	6.34308	75.2537	3.56851	0.0002	0.0481888
RNA-Seq	Trans-ABySS	XLOC_013132	R1754014:0-659	cdRNA03-Dia-R2	cdRNA05-postDia	26.078	2.53366	-3.36354	5.00E-05	0.0195319
RNA-Seq	Trans-ABySS	XLOC_013132	R1754014:0-659	cdRNA04-Dia-R3	cdRNA05-postDia	24.6756	2.53366	-3.28379	5.00E-05	0.0195319
RNA-Seq	Trans-ABySS	XLOC_013132	R1754014:0-659	cdRNA01-preDia	cdRNA04-Dia-R3	3.70888	24.6756	2.73403	0.0001	0.0354705
RNA-Seq	Trans-ABySS	XLOC_013132	R1754014:0-659	cdRNA01-preDia	cdRNA03-Dia-R2	3.70888	26.078	2.81378	5.00E-05	0.0195319
RNA-Seq	Trans-ABySS	XLOC_013161	R1754060:0-2205	cdRNA03-Dia-R2	cdRNA05-postDia	65.6749	7.60052	-3.11117	5.00E-05	0.0195319
RNA-Seq	Trans-ABySS	XLOC_013161	R1754060:0-2205	cdRNA04-Dia-R3	cdRNA05-postDia	64.4947	7.60052	-3.08501	5.00E-05	0.0195319
RNA-Seq	Trans-ABySS	XLOC_013161	R1754060:0-2205	cdRNA01-preDia	cdRNA04-Dia-R3	10.6447	64.4947	2.59905	0.0002	0.0481888
RNA-Seq	Trans-ABySS	XLOC_013202	R1754137:0-1614	cdRNA03-Dia-R2	cdRNA05-postDia	33.98	6.15511	-2.46483	0.00015	0.0374403
RNA-Seq	Trans-ABySS	XLOC_013202	R1754137:0-1614	cdRNA04-Dia-R3	cdRNA05-postDia	31.8068	6.15511	-2.36948	0.0001	0.0354705
RNA-Seq	Trans-ABySS	XLOC_013226	R1754182:0-830	cdRNA01-preDia	cdRNA05-postDia	15.1546	81.8084	2.43249	0.0001	0.0354705
RNA-Seq	Trans-ABySS	XLOC_013239	R1754215:0-1055	cdRNA04-Dia-R3	cdRNA05-postDia	14.3973	69.1407	2.26374	0.0002	0.0481888
RNA-Seq	Trans-ABySS	XLOC_013239	R1754215:0-1055	cdRNA02-Dia-R1	cdRNA05-postDia	9.82848	69.1407	2.8145	0.0001	0.0354705
RNA-Seq	Trans-ABySS	XLOC_013270	R1754256:1-741	cdRNA01-preDia	cdRNA05-postDia	10.7216	85.5351	2.99599	5.00E-05	0.0195319
RNA-Seq	Trans-ABySS	XLOC_013338	R1754387:4-496	cdRNA01-preDia	cdRNA04-Dia-R3	38.3238	4.595	-3.0601	5.00E-05	0.0195319
RNA-Seq	Trans-ABySS	XLOC_013361	R1754438:0-812	cdRNA01-preDia	cdRNA04-Dia-R3	0.975204	25.4174	4.70397	0.0002	0.0481888
RNA-Seq	Trans-ABySS	XLOC_013361	R1754438:0-812	cdRNA01-preDia	cdRNA03-Dia-R2	0.975204	26.1188	4.74324	0.0002	0.0481888
RNA-Seq	Trans-ABySS	XLOC_013361	R1754438:0-812	cdRNA01-preDia	cdRNA05-postDia	0.975204	34.4214	5.14146	0.0002	0.0481888
RNA-Seq	Trans-ABySS	XLOC_013383	R1754480:0-519	cdRNA01-preDia	cdRNA05-postDia	5.21241	39.412	2.91861	5.00E-05	0.0195319
RNA-Seq	Trans-ABySS	XLOC_013457	R1754613:0-305	cdRNA01-preDia	cdRNA05-postDia	17.0454	120.244	2.81851	0.0002	0.0481888
RNA-Seq	Trans-ABySS	XLOC_013457	R1754613:0-305	cdRNA04-Dia-R3	cdRNA05-postDia	8.18171	120.244	3.87742	0.0001	0.0354705
RNA-Seq	Trans-ABySS	XLOC_013457	R1754613:0-305	cdRNA03-Dia-R2	cdRNA05-postDia	8.12172	120.244	3.88804	0.00015	0.0374403
RNA-Seq	Trans-ABySS	XLOC_013591	R1754853:0-851	cdRNA04-Dia-R3	cdRNA05-postDia	10.4251	54.482	2.38572	0.00015	0.0374403
RNA-Seq	Trans-ABySS	XLOC_013591	R1754853:0-851	cdRNA03-Dia-R2	cdRNA05-postDia	10.3418	54.482	2.3973	0.0001	0.0354705
RNA-Seq	Trans-ABySS	XLOC_013649	R1754964:0-285	cdRNA01-preDia	cdRNA05-postDia	18.9356	153.402	3.01815	5.00E-05	0.0195319
RNA-Seq	Trans-ABySS	XLOC_013662	R1754987:0-256	cdRNA01-preDia	cdRNA03-Dia-R2	165.82	11.2819	-3.87754	0.0002	0.0481888
RNA-Seq	Trans-ABySS	XLOC_013662	R1754987:0-256	cdRNA01-preDia	cdRNA04-Dia-R3	165.82	20.919	-2.98673	0.0001	0.0354705
RNA-Seq	Trans-ABySS	XLOC_013678	R1755021:0-544	cdRNA04-Dia-R3	cdRNA05-postDia	14.5646	78.8967	2.4375	0.0001	0.0354705
RNA-Seq	Trans-ABySS	XLOC_013678	R1755021:0-544	cdRNA03-Dia-R2	cdRNA05-postDia	14.3004	78.8967	2.46391	5.00E-05	0.0195319
RNA-Seq	Trans-ABySS	XLOC_013678	R1755021:0-544	cdRNA01-preDia	cdRNA05-postDia	13.5485	78.8967	2.54183	5.00E-05	0.0195319
RNA-Seq	Trans-ABySS	XLOC_013694	R1755050:0-426	cdRNA03-Dia-R2	cdRNA05-postDia	85.7934	16.776	-2.35447	0.0001	0.0354705
RNA-Seq	Trans-ABySS	XLOC_013832	R1755292:1-244	cdRNA01-preDia	cdRNA05-postDia	53.4248	495.463	3.2132	5.00E-05	0.0195319
RNA-Seq	Trans-ABySS	XLOC_013832	R1755292:1-244	cdRNA02-Dia-R1	cdRNA05-postDia	18.1117	495.463	4.77379	5.00E-05	0.0195319
RNA-Seq	Trans-ABySS	XLOC_013832	R1755292:1-244	cdRNA04-Dia-R3	cdRNA05-postDia	16.6683	495.463	4.89359	5.00E-05	0.0195319
RNA-Seq	Trans-ABySS	XLOC_013832	R1755292:1-244	cdRNA03-Dia-R2	cdRNA05-postDia	12.4407	495.463	5.31564	5.00E-05	0.0195319
RNA-Seq	Trans-ABySS	XLOC_013833	R1755293:3-481	cdRNA01-preDia	cdRNA05-postDia	22.4713	109.551	2.28545	0.00015	0.0374403
RNA-Seq	Trans-ABySS	XLOC_013867	R1755345:0-470	cdRNA01-preDia	cdRNA05-postDia	11.3163	98.6775	3.12431	5.00E-05	0.0195319

RNA-Seq	Trans-ABySS	XLOC_013909	R1755422:0-601	cdRNA03-Dia-R2	cdRNA05-postDia	9.10116	58.5229	2.68488	5.00E-05	0.0195319
RNA-Seq	Trans-ABySS	XLOC_013932	R1755461:0-515	cdRNA02-Dia-R1	cdRNA05-postDia	2.26811	201.876	6.47583	5.00E-05	0.0195319
RNA-Seq	Trans-ABySS	XLOC_013950	R1755485:0-663	cdRNA02-Dia-R1	cdRNA05-postDia	15.5875	89.0646	2.51446	5.00E-05	0.0195319
RNA-Seq	Trans-ABySS	XLOC_013950	R1755485:0-663	cdRNA01-preDia	cdRNA05-postDia	4.07403	89.0646	4.45032	5.00E-05	0.0195319
RNA-Seq	Trans-ABySS	XLOC_014026	R1755634:0-892	cdRNA04-Dia-R3	cdRNA05-postDia	42.8763	5.80023	-2.886	5.00E-05	0.0195319
RNA-Seq	Trans-ABySS	XLOC_014026	R1755634:0-892	cdRNA03-Dia-R2	cdRNA05-postDia	41.7547	5.80023	-2.84776	5.00E-05	0.0195319
RNA-Seq	Trans-ABySS	XLOC_014041	R1755655:0-645	cdRNA01-preDia	cdRNA05-postDia	3.61977	39.2114	3.4373	5.00E-05	0.0195319
RNA-Seq	Trans-ABySS	XLOC_014185	R1755933:0-703	cdRNA01-preDia	cdRNA03-Dia-R2	12.7962	60.6086	2.24381	0.0002	0.0481888
RNA-Seq	Trans-ABySS	XLOC_014261	R1756069:0-2210	cdRNA01-preDia	cdRNA03-Dia-R2	5.7297	27.2407	2.24923	0.0001	0.0354705
RNA-Seq	Trans-ABySS	XLOC_014261	R1756069:0-2210	cdRNA01-preDia	cdRNA04-Dia-R3	5.7297	27.6659	2.27158	5.00E-05	0.0195319
RNA-Seq	Trans-ABySS	XLOC_014287	R1756110:0-872	cdRNA01-preDia	cdRNA02-Dia-R1	77.7685	3.97251	-4.29106	5.00E-05	0.0195319
RNA-Seq	Trans-ABySS	XLOC_014287	R1756110:0-872	cdRNA01-preDia	cdRNA05-postDia	77.7685	6.04026	-3.6865	5.00E-05	0.0195319
RNA-Seq	Trans-ABySS	XLOC_014287	R1756110:0-872	cdRNA01-preDia	cdRNA04-Dia-R3	77.7685	7.86061	-3.30647	5.00E-05	0.0195319
RNA-Seq	Trans-ABySS	XLOC_014287	R1756110:0-872	cdRNA01-preDia	cdRNA03-Dia-R2	77.7685	8.14688	-3.25487	5.00E-05	0.0195319
RNA-Seq	Trans-ABySS	XLOC_014429	R1756383:0-1679	cdRNA02-Dia-R1	cdRNA05-postDia	23.2971	1.50305	-3.95419	5.00E-05	0.0195319
RNA-Seq	Trans-ABySS	XLOC_014429	R1756383:0-1679	cdRNA04-Dia-R3	cdRNA05-postDia	21.8807	1.50305	-3.8637	5.00E-05	0.0195319
RNA-Seq	Trans-ABySS	XLOC_014429	R1756383:0-1679	cdRNA03-Dia-R2	cdRNA05-postDia	21.7747	1.50305	-3.85669	5.00E-05	0.0195319
RNA-Seq	Trans-ABySS	XLOC_014429	R1756383:0-1679	cdRNA01-preDia	cdRNA05-postDia	20.0375	1.50305	-3.73674	5.00E-05	0.0195319
RNA-Seq	Trans-ABySS	XLOC_014433	R1756391:0-1922	cdRNA03-Dia-R2	cdRNA05-postDia	38.714	4.07784	-3.24698	5.00E-05	0.0195319
RNA-Seq	Trans-ABySS	XLOC_014433	R1756391:0-1922	cdRNA04-Dia-R3	cdRNA05-postDia	36.9257	4.07784	-3.17875	5.00E-05	0.0195319
RNA-Seq	Trans-ABySS	XLOC_014433	R1756391:0-1922	cdRNA02-Dia-R1	cdRNA04-Dia-R3	6.60866	36.9257	2.48219	5.00E-05	0.0195319
RNA-Seq	Trans-ABySS	XLOC_014433	R1756391:0-1922	cdRNA02-Dia-R1	cdRNA03-Dia-R2	6.60866	38.714	2.55042	5.00E-05	0.0195319
RNA-Seq	Trans-ABySS	XLOC_014433	R1756391:0-1922	cdRNA01-preDia	cdRNA04-Dia-R3	0.610613	36.9257	5.91822	5.00E-05	0.0195319
RNA-Seq	Trans-ABySS	XLOC_014433	R1756391:0-1922	cdRNA01-preDia	cdRNA03-Dia-R2	0.610613	38.714	5.98645	5.00E-05	0.0195319
RNA-Seq	Trans-ABySS	XLOC_014435	R1756393:0-818	cdRNA03-Dia-R2	cdRNA05-postDia	54.4879	12.132	-2.16712	0.0002	0.0481888
RNA-Seq	Trans-ABySS	XLOC_014630	R1756747:0-1800	cdRNA01-preDia	cdRNA03-Dia-R2	49.7539	8.80421	-2.49854	5.00E-05	0.0195319
RNA-Seq	Trans-ABySS	XLOC_014630	R1756747:0-1800	cdRNA01-preDia	cdRNA04-Dia-R3	49.7539	8.90725	-2.48176	0.0001	0.0354705
RNA-Seq	Trans-ABySS	XLOC_014689	R1756847:0-1315	cdRNA01-preDia	cdRNA05-postDia	56.4069	6.18602	-3.18879	5.00E-05	0.0195319
RNA-Seq	Trans-ABySS	XLOC_014724	R1756903:0-726	cdRNA02-Dia-R1	cdRNA05-postDia	12.4194	101.759	3.0345	5.00E-05	0.0195319
RNA-Seq	Trans-ABySS	XLOC_014724	R1756903:0-726	cdRNA04-Dia-R3	cdRNA05-postDia	12.349	101.759	3.0427	5.00E-05	0.0195319
RNA-Seq	Trans-ABySS	XLOC_014724	R1756903:0-726	cdRNA03-Dia-R2	cdRNA05-postDia	11.7073	101.759	3.11969	5.00E-05	0.0195319
RNA-Seq	Trans-ABySS	XLOC_014875	R1757207:0-428	cdRNA03-Dia-R2	cdRNA05-postDia	16.3286	85.7049	2.39198	0.0002	0.0481888
RNA-Seq	Trans-ABySS	XLOC_014974	R1757394:0-575	cdRNA01-preDia	cdRNA03-Dia-R2	6.02382	51.7006	3.10143	5.00E-05	0.0195319
RNA-Seq	Trans-ABySS	XLOC_014974	R1757394:0-575	cdRNA01-preDia	cdRNA04-Dia-R3	6.02382	53.0454	3.13848	5.00E-05	0.0195319
RNA-Seq	Trans-ABySS	XLOC_014979	R1757400:0-1144	cdRNA04-Dia-R3	cdRNA05-postDia	14.76	86.9342	2.55823	5.00E-05	0.0195319
RNA-Seq	Trans-ABySS	XLOC_014979	R1757400:0-1144	cdRNA03-Dia-R2	cdRNA05-postDia	13.9992	86.9342	2.63457	5.00E-05	0.0195319
RNA-Seq	Trans-ABySS	XLOC_014979	R1757400:0-1144	cdRNA02-Dia-R1	cdRNA05-postDia	10.6132	86.9342	3.03407	5.00E-05	0.0195319
RNA-Seq	Trans-ABySS	XLOC_015035	R1757505:0-1033	cdRNA01-preDia	cdRNA05-postDia	8.08762	70.4407	3.12262	5.00E-05	0.0195319
RNA-Seq	Trans-ABySS	XLOC_015198	R1757839:0-259	cdRNA01-preDia	cdRNA05-postDia	180.912	22.9989	-2.97565	5.00E-05	0.0195319
RNA-Seq	Trans-ABySS	XLOC_015198	R1757839:0-259	cdRNA01-preDia	cdRNA03-Dia-R2	180.912	25.86	-2.80649	5.00E-05	0.0195319
RNA-Seq	Trans-ABySS	XLOC_015198	R1757839:0-259	cdRNA01-preDia	cdRNA04-Dia-R3	180.912	26.7023	-2.76025	0.0001	0.0354705
RNA-Seq	Trans-ABySS	XLOC_015205	R1757849:0-329	cdRNA02-Dia-R1	cdRNA05-postDia	15.6499	92.7861	2.56775	0.00015	0.0374403
RNA-Seq	Trans-ABySS	XLOC_015205	R1757849:0-329	cdRNA03-Dia-R2	cdRNA05-postDia	11.9766	92.7861	2.95369	5.00E-05	0.0195319
RNA-Seq	Trans-ABySS	XLOC_015205	R1757849:0-329	cdRNA01-preDia	cdRNA05-postDia	5.04232	92.7861	4.20175	5.00E-05	0.0195319
RNA-Seq	Trans-ABySS	XLOC_015208	R1757857:1-537	cdRNA01-preDia	cdRNA05-postDia	6.59403	47.6774	2.85407	5.00E-05	0.0195319
RNA-Seq	Trans-ABySS	XLOC_015217	R1757875:0-62	cdRNA01-preDia	cdRNA05-postDia	11261.2	109476	3.28118	5.00E-05	0.0195319
RNA-Seq	Trans-ABySS	XLOC_015217	R1757875:0-62	cdRNA02-Dia-R1	cdRNA05-postDia	10959.4	109476	3.32037	5.00E-05	0.0195319

RNA-Seq	Trans-ABySS	XLOC_015217	R1757875:0-62	cdRNA03-Dia-R2	cdRNA05-postDia	8260.32	109476	3.72827	0.0001	0.0354705
RNA-Seq	Trans-ABySS	XLOC_015225	R1757892:0-384	cdRNA04-Dia-R3	cdRNA05-postDia	8.52883	64.223	2.91267	5.00E-05	0.0195319
RNA-Seq	Trans-ABySS	XLOC_015225	R1757892:0-384	cdRNA01-preDia	cdRNA05-postDia	6.32785	64.223	3.3433	5.00E-05	0.0195319
RNA-Seq	Trans-ABySS	XLOC_015235	R1757905:8-262	cdRNA02-Dia-R1	cdRNA05-postDia	28.2826	183.077	2.69446	0.00015	0.0374403
RNA-Seq	Trans-ABySS	XLOC_015244	R1757917:0-293	cdRNA03-Dia-R2	cdRNA05-postDia	21.9113	178.025	3.02233	5.00E-05	0.0195319
RNA-Seq	Trans-ABySS	XLOC_015244	R1757917:0-293	cdRNA02-Dia-R1	cdRNA05-postDia	21.5212	178.025	3.04824	0.0001	0.0354705
RNA-Seq	Trans-ABySS	XLOC_015244	R1757917:0-293	cdRNA04-Dia-R3	cdRNA05-postDia	14.1316	178.025	3.65508	5.00E-05	0.0195319
RNA-Seq	Trans-ABySS	XLOC_015244	R1757917:0-293	cdRNA01-preDia	cdRNA05-postDia	12.1758	178.025	3.86999	5.00E-05	0.0195319
RNA-Seq	Trans-ABySS	XLOC_015344	R1758096:0-350	cdRNA01-preDia	cdRNA04-Dia-R3	9.01016	80.5536	3.16033	5.00E-05	0.0195319
RNA-Seq	Trans-ABySS	XLOC_015344	R1758096:0-350	cdRNA01-preDia	cdRNA03-Dia-R2	9.01016	80.8291	3.16525	5.00E-05	0.0195319
RNA-Seq	Trans-ABySS	XLOC_015376	R1758139:0-327	cdRNA01-preDia	cdRNA05-postDia	23.0347	137.914	2.58189	5.00E-05	0.0195319
RNA-Seq	Trans-ABySS	XLOC_015376	R1758139:0-327	cdRNA04-Dia-R3	cdRNA05-postDia	22.9291	137.914	2.58851	0.0002	0.0481888
RNA-Seq	Trans-ABySS	XLOC_015603	R1758539:0-1380	cdRNA04-Dia-R3	cdRNA05-postDia	39.024	4.18514	-3.22102	5.00E-05	0.0195319
RNA-Seq	Trans-ABySS	XLOC_015603	R1758539:0-1380	cdRNA03-Dia-R2	cdRNA05-postDia	34.9732	4.18514	-3.0629	5.00E-05	0.0195319
RNA-Seq	Trans-ABySS	XLOC_015708	R1758728:0-582	cdRNA02-Dia-R1	cdRNA05-postDia	11.5521	76.244	2.72247	5.00E-05	0.0195319
RNA-Seq	Trans-ABySS	XLOC_015742	R1758783:0-1208	cdRNA03-Dia-R2	cdRNA05-postDia	7.90855	45.1789	2.51416	0.0001	0.0354705
RNA-Seq	Trans-ABySS	XLOC_015919	R1759109:0-1127	cdRNA01-preDia	cdRNA05-postDia	32.9597	2.20978	-3.89873	5.00E-05	0.0195319
RNA-Seq	Trans-ABySS	XLOC_015919	R1759109:0-1127	cdRNA02-Dia-R1	cdRNA05-postDia	27.8769	2.20978	-3.65709	5.00E-05	0.0195319
RNA-Seq	Trans-ABySS	XLOC_015919	R1759109:0-1127	cdRNA03-Dia-R2	cdRNA05-postDia	22.763	2.20978	-3.36472	5.00E-05	0.0195319
RNA-Seq	Trans-ABySS	XLOC_015919	R1759109:0-1127	cdRNA04-Dia-R3	cdRNA05-postDia	20.767	2.20978	-3.23232	5.00E-05	0.0195319
RNA-Seq	Trans-ABySS	XLOC_015928	R1759120:2-1249	cdRNA04-Dia-R3	cdRNA05-postDia	6.54887	38.1756	2.54333	0.0001	0.0354705
RNA-Seq	Trans-ABySS	XLOC_015965	R1759186:5-236	cdRNA02-Dia-R1	cdRNA05-postDia	27.1987	231.911	3.09196	5.00E-05	0.0195319
RNA-Seq	Trans-ABySS	XLOC_015983	R1759218:0-1654	cdRNA01-preDia	cdRNA03-Dia-R2	1.41549	12.0903	3.09448	5.00E-05	0.0195319
RNA-Seq	Trans-ABySS	XLOC_015983	R1759218:0-1654	cdRNA01-preDia	cdRNA04-Dia-R3	1.41549	13.4549	3.24876	5.00E-05	0.0195319
RNA-Seq	Trans-ABySS	XLOC_015998	R1759248:0-1059	cdRNA01-preDia	cdRNA05-postDia	10.6156	89.924	3.08252	5.00E-05	0.0195319
RNA-Seq	Trans-ABySS	XLOC_015998	R1759248:0-1059	cdRNA03-Dia-R2	cdRNA05-postDia	8.34764	89.924	3.42927	5.00E-05	0.0195319
RNA-Seq	Trans-ABySS	XLOC_015998	R1759248:0-1059	cdRNA04-Dia-R3	cdRNA05-postDia	8.18855	89.924	3.45703	5.00E-05	0.0195319
RNA-Seq	Trans-ABySS	XLOC_015998	R1759248:0-1059	cdRNA02-Dia-R1	cdRNA05-postDia	6.52166	89.924	3.78539	5.00E-05	0.0195319
RNA-Seq	Trans-ABySS	XLOC_016218	R1759624:0-1024	cdRNA01-preDia	cdRNA04-Dia-R3	5.56217	29.0502	2.38483	0.00015	0.0374403
RNA-Seq	Trans-ABySS	XLOC_016244	R1759676:3-1369	cdRNA01-preDia	cdRNA03-Dia-R2	6.59422	32.976	2.32215	0.00015	0.0374403
RNA-Seq	Trans-ABySS	XLOC_016304	R1759796:0-500	cdRNA02-Dia-R1	cdRNA05-postDia	1.92913	128.821	6.06128	0.0002	0.0481888
RNA-Seq	Trans-ABySS	XLOC_016550	R1760226:0-608	cdRNA02-Dia-R1	cdRNA05-postDia	5.15489	45.8754	3.15371	0.0001	0.0354705
RNA-Seq	Trans-ABySS	XLOC_016550	R1760226:0-608	cdRNA04-Dia-R3	cdRNA05-postDia	4.12102	45.8754	3.47665	5.00E-05	0.0195319
RNA-Seq	Trans-ABySS	XLOC_016550	R1760226:0-608	cdRNA01-preDia	cdRNA05-postDia	2.82234	45.8754	4.02276	5.00E-05	0.0195319
RNA-Seq	Trans-ABySS	XLOC_016550	R1760226:0-608	cdRNA03-Dia-R2	cdRNA05-postDia	2.76033	45.8754	4.05481	5.00E-05	0.0195319
RNA-Seq	Trans-ABySS	XLOC_016583	R1760280:0-688	cdRNA04-Dia-R3	cdRNA05-postDia	11.9955	69.4402	2.53328	5.00E-05	0.0195319
RNA-Seq	Trans-ABySS	XLOC_016583	R1760280:0-688	cdRNA01-preDia	cdRNA05-postDia	5.46558	69.4402	3.66733	0.0001	0.0354705
RNA-Seq	Trans-ABySS	XLOC_016693	R1760498:0-1734	cdRNA01-preDia	cdRNA04-Dia-R3	3.45802	19.6432	2.50601	0.0001	0.0354705
RNA-Seq	Trans-ABySS	XLOC_016694	R1760499:5-964	cdRNA01-preDia	cdRNA05-postDia	31.5945	4.81569	-2.71386	0.0001	0.0354705
RNA-Seq	Trans-ABySS	XLOC_016925	R1760931:0-347	cdRNA04-Dia-R3	cdRNA05-postDia	11.2741	101.997	3.17745	5.00E-05	0.0195319
RNA-Seq	Trans-ABySS	XLOC_016925	R1760931:0-347	cdRNA03-Dia-R2	cdRNA05-postDia	9.61035	101.997	3.4078	5.00E-05	0.0195319
RNA-Seq	Trans-ABySS	XLOC_016925	R1760931:0-347	cdRNA01-preDia	cdRNA05-postDia	8.24137	101.997	3.6295	5.00E-05	0.0195319
RNA-Seq	Trans-ABySS	XLOC_017154	R1761386:1-1563	cdRNA03-Dia-R2	cdRNA05-postDia	9.32891	47.3111	2.3424	0.00015	0.0374403
RNA-Seq	Trans-ABySS	XLOC_017192	R1761442:0-1715	cdRNA04-Dia-R3	cdRNA05-postDia	6.61125	35.4778	2.42392	0.00015	0.0374403
RNA-Seq	Trans-ABySS	XLOC_017192	R1761442:0-1715	cdRNA03-Dia-R2	cdRNA05-postDia	6.22918	35.4778	2.50981	0.00015	0.0374403
RNA-Seq	Trans-ABySS	XLOC_017455	R1761923:0-618	cdRNA01-preDia	cdRNA04-Dia-R3	21.152	1.54673	-3.77351	0.00015	0.0374403
RNA-Seq	Trans-ABySS	XLOC_017772	R1762519:0-769	cdRNA03-Dia-R2	cdRNA05-postDia	25.9749	3.76688	-2.78568	5.00E-05	0.0195319

RNA-Seq	Trans-ABySS	XLOC_017772	R1762519:0-769	cdRNA04-Dia-R3	cdRNA05-postDia	24.5562	3.76688	-2.70464	5.00E-05	0.0195319
RNA-Seq	Trans-ABySS	XLOC_017876	R1762701:4-637	cdRNA03-Dia-R2	cdRNA05-postDia	36.2887	4.13417	-3.13385	5.00E-05	0.0195319
RNA-Seq	Trans-ABySS	XLOC_017876	R1762701:4-637	cdRNA04-Dia-R3	cdRNA05-postDia	35.724	4.13417	-3.11122	5.00E-05	0.0195319
RNA-Seq	Trans-ABySS	XLOC_018063	R1763068:0-1101	cdRNA02-Dia-R1	cdRNA05-postDia	1.42301	81.413	5.83824	5.00E-05	0.0195319
RNA-Seq	Trans-ABySS	XLOC_018320	R1763553:0-1324	cdRNA01-preDia	cdRNA03-Dia-R2	1.79182	20.7506	3.53366	5.00E-05	0.0195319
RNA-Seq	Trans-ABySS	XLOC_018320	R1763553:0-1324	cdRNA01-preDia	cdRNA04-Dia-R3	1.79182	21.8026	3.605	5.00E-05	0.0195319
RNA-Seq	Trans-ABySS	XLOC_018320	R1763553:0-1324	cdRNA01-preDia	cdRNA05-postDia	1.79182	27.554	3.94277	5.00E-05	0.0195319
RNA-Seq	Trans-ABySS	XLOC_018478	R1763868:0-1620	cdRNA03-Dia-R2	cdRNA05-postDia	3.498	24.6634	2.81777	5.00E-05	0.0195319
RNA-Seq	Trans-ABySS	XLOC_018478	R1763868:0-1620	cdRNA04-Dia-R3	cdRNA05-postDia	3.40976	24.6634	2.85463	5.00E-05	0.0195319
RNA-Seq	Trans-ABySS	XLOC_018531	R1763963:0-570	cdRNA01-preDia	cdRNA05-postDia	4.85996	34.6472	2.83372	0.0002	0.0481888
RNA-Seq	Trans-ABySS	XLOC_018547	R1763987:0-583	cdRNA03-Dia-R2	cdRNA05-postDia	26.2462	3.50967	-2.9027	5.00E-05	0.0195319
RNA-Seq	Trans-ABySS	XLOC_018736	R1764330:0-1776	cdRNA01-preDia	cdRNA05-postDia	25.2713	3.55397	-2.83	5.00E-05	0.0195319
RNA-Seq	Trans-ABySS	XLOC_018736	R1764330:0-1776	cdRNA02-Dia-R1	cdRNA05-postDia	18.6758	3.55397	-2.39366	0.00015	0.0374403
RNA-Seq	Trans-ABySS	XLOC_019193	R1765203:0-278	cdRNA04-Dia-R3	cdRNA05-postDia	8.98248	152.016	4.08097	0.0002	0.0481888
RNA-Seq	Trans-ABySS	XLOC_019399	R1765602:0-1761	cdRNA03-Dia-R2	cdRNA05-postDia	16.1299	1.70867	-3.23879	0.0002	0.0481888
RNA-Seq	Trans-ABySS	XLOC_019399	R1765602:0-1761	cdRNA04-Dia-R3	cdRNA05-postDia	14.477	1.70867	-3.08281	0.0002	0.0481888
RNA-Seq	Trans-ABySS	XLOC_019399	R1765602:0-1761	cdRNA02-Dia-R1	cdRNA05-postDia	13.0954	1.70867	-2.93812	0.0002	0.0481888
RNA-Seq	Trans-ABySS	XLOC_019418	R1765626:0-524	cdRNA03-Dia-R2	cdRNA05-postDia	7.38796	48.911	2.72691	0.0001	0.0354705
RNA-Seq	Trans-ABySS	XLOC_019418	R1765626:0-524	cdRNA04-Dia-R3	cdRNA05-postDia	7.20381	48.911	2.76333	5.00E-05	0.0195319
RNA-Seq	Trans-ABySS	XLOC_019418	R1765626:0-524	cdRNA02-Dia-R1	cdRNA05-postDia	6.61296	48.911	2.88679	5.00E-05	0.0195319
RNA-Seq	Trans-ABySS	XLOC_019443	R1765662:0-788	cdRNA04-Dia-R3	cdRNA05-postDia	5.84795	33.786	2.53042	5.00E-05	0.0195319
RNA-Seq	Trans-ABySS	XLOC_019457	R1765682:0-1358	cdRNA02-Dia-R1	cdRNA05-postDia	3.69672	19.4419	2.39485	5.00E-05	0.0195319
RNA-Seq	Trans-ABySS	XLOC_019517	R1765786:0-482	cdRNA01-preDia	cdRNA05-postDia	24.6358	176.595	2.84162	5.00E-05	0.0195319
RNA-Seq	Trans-ABySS	XLOC_019572	R1765873:0-1253	cdRNA03-Dia-R2	cdRNA05-postDia	2.45224	12.2582	2.32158	0.0002	0.0481888
RNA-Seq	Trans-ABySS	XLOC_019897	R1766493:2-1879	cdRNA04-Dia-R3	cdRNA05-postDia	6.63452	33.9507	2.35538	0.0001	0.0354705
RNA-Seq	Trans-ABySS	XLOC_019897	R1766493:2-1879	cdRNA01-preDia	cdRNA05-postDia	5.61607	33.9507	2.59581	5.00E-05	0.0195319
RNA-Seq	Trans-ABySS	XLOC_020003	R1766664:4-852	cdRNA02-Dia-R1	cdRNA05-postDia	6.78066	57.1456	3.07514	5.00E-05	0.0195319
RNA-Seq	Trans-ABySS	XLOC_020003	R1766664:4-852	cdRNA04-Dia-R3	cdRNA05-postDia	6.0323	57.1456	3.24386	5.00E-05	0.0195319
RNA-Seq	Trans-ABySS	XLOC_020003	R1766664:4-852	cdRNA03-Dia-R2	cdRNA05-postDia	6.02616	57.1456	3.24533	5.00E-05	0.0195319
RNA-Seq	Trans-ABySS	XLOC_020003	R1766664:4-852	cdRNA01-preDia	cdRNA05-postDia	2.33714	57.1456	4.61183	5.00E-05	0.0195319
RNA-Seq	Trans-ABySS	XLOC_020175	R1767015:0-982	cdRNA04-Dia-R3	cdRNA05-postDia	16.2969	2.33666	-2.80208	0.0001	0.0354705
RNA-Seq	Trans-ABySS	XLOC_020175	R1767015:0-982	cdRNA03-Dia-R2	cdRNA05-postDia	15.9395	2.33666	-2.77008	0.0001	0.0354705
RNA-Seq	Trans-ABySS	XLOC_020213	R1767080:0-836	cdRNA02-Dia-R1	cdRNA05-postDia	6.9094	35.0787	2.34396	0.0002	0.0481888
RNA-Seq	Trans-ABySS	XLOC_020213	R1767080:0-836	cdRNA04-Dia-R3	cdRNA05-postDia	6.55212	35.0787	2.42056	5.00E-05	0.0195319
RNA-Seq	Trans-ABySS	XLOC_020213	R1767080:0-836	cdRNA03-Dia-R2	cdRNA05-postDia	5.92884	35.0787	2.56477	5.00E-05	0.0195319
RNA-Seq	Trans-ABySS	XLOC_020839	R1768349:0-721	cdRNA03-Dia-R2	cdRNA05-postDia	5.69454	31.7321	2.47829	0.0001	0.0354705
RNA-Seq	Trans-ABySS	XLOC_020839	R1768349:0-721	cdRNA04-Dia-R3	cdRNA05-postDia	3.8809	31.7321	3.03148	5.00E-05	0.0195319
RNA-Seq	Trans-ABySS	XLOC_021013	R1768628:0-1036	cdRNA01-preDia	cdRNA04-Dia-R3	2.85061	21.0373	2.8836	5.00E-05	0.0195319
RNA-Seq	Trans-ABySS	XLOC_021013	R1768628:0-1036	cdRNA01-preDia	cdRNA03-Dia-R2	2.85061	23.2689	3.02906	5.00E-05	0.0195319
RNA-Seq	Trans-ABySS	XLOC_021799	R1770204:0-976	cdRNA01-preDia	cdRNA05-postDia	1.59516	13.9566	3.12918	0.00015	0.0374403
RNA-Seq	Trans-ABySS	XLOC_021855	R1770302:1-673	cdRNA02-Dia-R1	cdRNA05-postDia	7.17737	39.0336	2.44319	0.0002	0.0481888
RNA-Seq	Trans-ABySS	XLOC_021855	R1770302:1-673	cdRNA04-Dia-R3	cdRNA05-postDia	7.11496	39.0336	2.45579	0.0002	0.0481888
RNA-Seq	Trans-ABySS	XLOC_021855	R1770302:1-673	cdRNA03-Dia-R2	cdRNA05-postDia	6.10012	39.0336	2.67781	0.00015	0.0374403
RNA-Seq	Trans-ABySS	XLOC_022699	R1771914:0-1134	cdRNA01-preDia	cdRNA03-Dia-R2	20.3037	3.69333	-2.45875	0.0002	0.0481888
RNA-Seq	Trans-ABySS	XLOC_022838	R1772153:0-593	cdRNA01-preDia	cdRNA04-Dia-R3	26.2633	3.62148	-2.8584	5.00E-05	0.0195319
RNA-Seq	Trans-ABySS	XLOC_022838	R1772153:0-593	cdRNA01-preDia	cdRNA03-Dia-R2	26.2633	4.35748	-2.59148	0.0002	0.0481888
RNA-Seq	Trans-ABySS	XLOC_023757	R1774122:0-555	cdRNA02-Dia-R1	cdRNA05-postDia	34.6424	2.65274	-3.70698	0.0002	0.0481888

RNA-Seq	Trans-ABySS	XLOC_023765	R1774137:0-1145	cdRNA04-Dia-R3	cdRNA05-postDia	3.7201	17.9998	2.27457	0.00015	0.0374403
RNA-Seq	Trans-ABySS	XLOC_023765	R1774137:0-1145	cdRNA03-Dia-R2	cdRNA05-postDia	3.36573	17.9998	2.41899	0.0002	0.0481888
RNA-Seq	Trans-ABySS	XLOC_025087	R1777048:0-519	cdRNA01-preDia	cdRNA04-Dia-R3	33.8807	2.03282	-4.05891	0.00015	0.0374403
RNA-Seq	Trans-ABySS	XLOC_029303	S1012078:0-441	cdRNA01-preDia	cdRNA05-postDia	4.61853	43.5896	3.23848	0.0001	0.0354705
RNA-Seq	Trans-ABySS	XLOC_029497	S1013860:0-387	cdRNA01-preDia	cdRNA05-postDia	19.4224	118.857	2.61343	0.00015	0.0374403
RNA-Seq	Trans-ABySS	XLOC_029911	S1017779:0-331	cdRNA02-Dia-R1	cdRNA05-postDia	17.5095	102.8	2.55362	5.00E-05	0.0195319
RNA-Seq	Trans-ABySS	XLOC_030525	S1023104:0-920	cdRNA02-Dia-R1	cdRNA05-postDia	4.78522	29.4152	2.6199	0.0001	0.0354705
RNA-Seq	Trans-ABySS	XLOC_030837	S1025990:0-423	cdRNA03-Dia-R2	cdRNA05-postDia	9.36884	64.1756	2.77608	0.0001	0.0354705
RNA-Seq	Trans-ABySS	XLOC_030837	S1025990:0-423	cdRNA02-Dia-R1	cdRNA05-postDia	7.82176	64.1756	3.03646	5.00E-05	0.0195319
RNA-Seq	Trans-ABySS	XLOC_031223	S1029596:0-1150	cdRNA04-Dia-R3	cdRNA05-postDia	6.31781	35.4812	2.48956	0.0001	0.0354705
RNA-Seq	Trans-ABySS	XLOC_031730	S1034390:0-1526	cdRNA04-Dia-R3	cdRNA05-postDia	6.13555	35.4183	2.52923	0.00015	0.0374403
RNA-Seq	Trans-ABySS	XLOC_031730	S1034390:0-1526	cdRNA03-Dia-R2	cdRNA05-postDia	5.90498	35.4183	2.58449	0.00015	0.0374403
RNA-Seq	Trans-ABySS	XLOC_034322	S1059164:0-868	cdRNA01-preDia	cdRNA05-postDia	59.5535	7.54572	-2.98046	5.00E-05	0.0195319
RNA-Seq	Trans-ABySS	XLOC_034709	S1062778:0-342	cdRNA01-preDia	cdRNA05-postDia	4.59176	79.3935	4.1119	5.00E-05	0.0195319
RNA-Seq	Trans-ABySS	XLOC_035980	S1073770:0-521	cdRNA04-Dia-R3	cdRNA05-postDia	5.11732	53.2902	3.38041	5.00E-05	0.0195319
RNA-Seq	Trans-ABySS	XLOC_035980	S1073770:0-521	cdRNA03-Dia-R2	cdRNA05-postDia	4.08072	53.2902	3.70697	5.00E-05	0.0195319
RNA-Seq	Trans-ABySS	XLOC_036531	S1078558:0-575	cdRNA04-Dia-R3	cdRNA05-postDia	13.1175	76.6069	2.54598	5.00E-05	0.0195319
RNA-Seq	Trans-ABySS	XLOC_037194	S108458:0-559	cdRNA03-Dia-R2	cdRNA05-postDia	9.54961	66.5705	2.80137	5.00E-05	0.0195319
RNA-Seq	Trans-ABySS	XLOC_037194	S108458:0-559	cdRNA04-Dia-R3	cdRNA05-postDia	9.37964	66.5705	2.82728	5.00E-05	0.0195319
RNA-Seq	Trans-ABySS	XLOC_037299	S1085387:0-302	cdRNA03-Dia-R2	cdRNA05-postDia	11.8489	94.2215	2.99131	0.0002	0.0481888
RNA-Seq	Trans-ABySS	XLOC_037596	S1087868:2-1160	cdRNA01-preDia	cdRNA05-postDia	62.6299	2.0915	-4.90424	5.00E-05	0.0195319
RNA-Seq	Trans-ABySS	XLOC_037596	S1087868:2-1160	cdRNA01-preDia	cdRNA02-Dia-R1	62.6299	3.30976	-4.24205	5.00E-05	0.0195319
RNA-Seq	Trans-ABySS	XLOC_037596	S1087868:2-1160	cdRNA01-preDia	cdRNA04-Dia-R3	62.6299	5.05662	-3.63061	5.00E-05	0.0195319
RNA-Seq	Trans-ABySS	XLOC_037596	S1087868:2-1160	cdRNA01-preDia	cdRNA03-Dia-R2	62.6299	5.37725	-3.54191	5.00E-05	0.0195319
RNA-Seq	Trans-ABySS	XLOC_037631	S1088120:0-1222	cdRNA01-preDia	cdRNA03-Dia-R2	38.7102	3.94371	-3.29509	5.00E-05	0.0195319
RNA-Seq	Trans-ABySS	XLOC_037631	S1088120:0-1222	cdRNA01-preDia	cdRNA04-Dia-R3	38.7102	4.40323	-3.13608	5.00E-05	0.0195319
RNA-Seq	Trans-ABySS	XLOC_037631	S1088120:0-1222	cdRNA02-Dia-R1	cdRNA05-postDia	8.67812	42.5104	2.29236	0.0001	0.0354705
RNA-Seq	Trans-ABySS	XLOC_037631	S1088120:0-1222	cdRNA04-Dia-R3	cdRNA05-postDia	4.40323	42.5104	3.27118	5.00E-05	0.0195319
RNA-Seq	Trans-ABySS	XLOC_037631	S1088120:0-1222	cdRNA03-Dia-R2	cdRNA05-postDia	3.94371	42.5104	3.43019	5.00E-05	0.0195319
RNA-Seq	Trans-ABySS	XLOC_038790	S1098319:0-549	cdRNA03-Dia-R2	cdRNA05-postDia	2.32731	24.2899	3.38362	0.0002	0.0481888
RNA-Seq	Trans-ABySS	XLOC_039314	S1103126:0-394	cdRNA01-preDia	cdRNA05-postDia	14.6341	147.63	3.33458	0.0001	0.0354705
RNA-Seq	Trans-ABySS	XLOC_040193	S1111262:0-1138	cdRNA01-preDia	cdRNA04-Dia-R3	26.6139	2.37676	-3.48511	5.00E-05	0.0195319
RNA-Seq	Trans-ABySS	XLOC_040193	S1111262:0-1138	cdRNA01-preDia	cdRNA03-Dia-R2	26.6139	3.00887	-3.14488	5.00E-05	0.0195319
RNA-Seq	Trans-ABySS	XLOC_040483	S1113675:0-920	cdRNA03-Dia-R2	cdRNA05-postDia	8.03684	58.0873	2.85352	5.00E-05	0.0195319
RNA-Seq	Trans-ABySS	XLOC_040483	S1113675:0-920	cdRNA04-Dia-R3	cdRNA05-postDia	7.69139	58.0873	2.91691	5.00E-05	0.0195319
RNA-Seq	Trans-ABySS	XLOC_040483	S1113675:0-920	cdRNA01-preDia	cdRNA05-postDia	7.07077	58.0873	3.03828	5.00E-05	0.0195319
RNA-Seq	Trans-ABySS	XLOC_040483	S1113675:0-920	cdRNA02-Dia-R1	cdRNA05-postDia	5.67659	58.0873	3.35513	5.00E-05	0.0195319
RNA-Seq	Trans-ABySS	XLOC_042131	S1128148:0-559	cdRNA01-preDia	cdRNA05-postDia	24.7958	1.87347	-3.72631	0.0002	0.0481888
RNA-Seq	Trans-ABySS	XLOC_042646	S1132859:0-528	cdRNA04-Dia-R3	cdRNA05-postDia	8.30069	51.0441	2.62044	0.0002	0.0481888
RNA-Seq	Trans-ABySS	XLOC_042646	S1132859:0-528	cdRNA03-Dia-R2	cdRNA05-postDia	7.43443	51.0441	2.77945	5.00E-05	0.0195319
RNA-Seq	Trans-ABySS	XLOC_042646	S1132859:0-528	cdRNA02-Dia-R1	cdRNA05-postDia	6.22031	51.0441	3.03669	0.0001	0.0354705
RNA-Seq	Trans-ABySS	XLOC_043063	S1136297:0-462	cdRNA02-Dia-R1	cdRNA05-postDia	21.8721	143.326	2.71213	5.00E-05	0.0195319
RNA-Seq	Trans-ABySS	XLOC_043063	S1136297:0-462	cdRNA01-preDia	cdRNA05-postDia	5.30328	143.326	4.75627	5.00E-05	0.0195319
RNA-Seq	Trans-ABySS	XLOC_043063	S1136297:0-462	cdRNA03-Dia-R2	cdRNA05-postDia	4.49519	143.326	4.99477	5.00E-05	0.0195319
RNA-Seq	Trans-ABySS	XLOC_043063	S1136297:0-462	cdRNA04-Dia-R3	cdRNA05-postDia	3.47467	143.326	5.36628	5.00E-05	0.0195319
RNA-Seq	Trans-ABySS	XLOC_044707	S1150153:0-675	cdRNA01-preDia	cdRNA05-postDia	26.4359	130.099	2.29904	0.00015	0.0374403
RNA-Seq	Trans-ABySS	XLOC_044707	S1150153:0-675	cdRNA04-Dia-R3	cdRNA05-postDia	19.7587	130.099	2.71905	5.00E-05	0.0195319

RNA-Seq	Trans-ABySS	XLOC_044707	S1150153:0-675	cdRNA02-Dia-R1	cdRNA05-postDia	18.0431	130.099	2.85009	5.00E-05	0.0195319
RNA-Seq	Trans-ABySS	XLOC_044707	S1150153:0-675	cdRNA03-Dia-R2	cdRNA05-postDia	17.7103	130.099	2.87695	5.00E-05	0.0195319
RNA-Seq	Trans-ABySS	XLOC_045010	S1152996:0-410	cdRNA01-preDia	cdRNA05-postDia	10.6252	63.0516	2.56905	0.0001	0.0354705
RNA-Seq	Trans-ABySS	XLOC_045010	S1152996:0-410	cdRNA02-Dia-R1	cdRNA05-postDia	9.12937	63.0516	2.78795	0.0002	0.0481888
RNA-Seq	Trans-ABySS	XLOC_046550	S116667:1-775	cdRNA01-preDia	cdRNA03-Dia-R2	25.9254	4.38248	-2.56455	0.0002	0.0481888
RNA-Seq	Trans-ABySS	XLOC_046550	S116667:1-775	cdRNA02-Dia-R1	cdRNA05-postDia	9.66456	52.3515	2.43746	0.0001	0.0354705
RNA-Seq	Trans-ABySS	XLOC_046550	S116667:1-775	cdRNA04-Dia-R3	cdRNA05-postDia	5.84179	52.3515	3.16375	5.00E-05	0.0195319
RNA-Seq	Trans-ABySS	XLOC_046550	S116667:1-775	cdRNA03-Dia-R2	cdRNA05-postDia	4.38248	52.3515	3.57841	5.00E-05	0.0195319
RNA-Seq	Trans-ABySS	XLOC_049709	S1193909:0-843	cdRNA03-Dia-R2	cdRNA05-postDia	3.21118	20.6085	2.68206	0.0001	0.0354705
RNA-Seq	Trans-ABySS	XLOC_049865	S119526:0-1408	cdRNA04-Dia-R3	cdRNA05-postDia	9.53799	58.8498	2.62528	5.00E-05	0.0195319
RNA-Seq	Trans-ABySS	XLOC_050036	S1196816:0-627	cdRNA01-preDia	cdRNA03-Dia-R2	3.12687	25.7308	3.0407	5.00E-05	0.0195319
RNA-Seq	Trans-ABySS	XLOC_050036	S1196816:0-627	cdRNA01-preDia	cdRNA04-Dia-R3	3.12687	27.4505	3.13405	5.00E-05	0.0195319
RNA-Seq	Trans-ABySS	XLOC_050193	S1198265:0-969	cdRNA01-preDia	cdRNA05-postDia	7.21409	36.1672	2.32579	0.0001	0.0354705
RNA-Seq	Trans-ABySS	XLOC_050257	S1198872:0-599	cdRNA01-preDia	cdRNA04-Dia-R3	3.46398	31.9901	3.20713	5.00E-05	0.0195319
RNA-Seq	Trans-ABySS	XLOC_050257	S1198872:0-599	cdRNA01-preDia	cdRNA03-Dia-R2	3.46398	35.6855	3.36484	5.00E-05	0.0195319
RNA-Seq	Trans-ABySS	XLOC_050383	S1199734:0-656	cdRNA04-Dia-R3	cdRNA05-postDia	5.76122	33.6467	2.54602	5.00E-05	0.0195319
RNA-Seq	Trans-ABySS	XLOC_053034	S1223550:9-344	cdRNA02-Dia-R1	cdRNA05-postDia	17.0052	185.607	3.4482	5.00E-05	0.0195319
RNA-Seq	Trans-ABySS	XLOC_053034	S1223550:9-344	cdRNA03-Dia-R2	cdRNA05-postDia	12.471	185.607	3.89559	5.00E-05	0.0195319
RNA-Seq	Trans-ABySS	XLOC_053034	S1223550:9-344	cdRNA04-Dia-R3	cdRNA05-postDia	9.35446	185.607	4.31045	5.00E-05	0.0195319
RNA-Seq	Trans-ABySS	XLOC_053034	S1223550:9-344	cdRNA01-preDia	cdRNA05-postDia	5.51404	185.607	5.073	5.00E-05	0.0195319
RNA-Seq	Trans-ABySS	XLOC_053988	S1231754:0-447	cdRNA01-preDia	cdRNA04-Dia-R3	9.75975	52.7021	2.43294	0.0002	0.0481888
RNA-Seq	Trans-ABySS	XLOC_054457	S1235595:0-484	cdRNA01-preDia	cdRNA03-Dia-R2	6.18971	43.8098	2.82331	0.00015	0.0374403
RNA-Seq	Trans-ABySS	XLOC_054457	S1235595:0-484	cdRNA01-preDia	cdRNA04-Dia-R3	6.18971	45.1283	2.86609	0.0001	0.0354705
RNA-Seq	Trans-ABySS	XLOC_055405	S1243407:1-912	cdRNA04-Dia-R3	cdRNA05-postDia	36.1085	3.3239	-3.44139	5.00E-05	0.0195319
RNA-Seq	Trans-ABySS	XLOC_055405	S1243407:1-912	cdRNA03-Dia-R2	cdRNA05-postDia	34.5756	3.3239	-3.37881	5.00E-05	0.0195319
RNA-Seq	Trans-ABySS	XLOC_055779	S1246452:0-377	cdRNA01-preDia	cdRNA02-Dia-R1	64.7547	3.29567	-4.29634	0.00015	0.0374403
RNA-Seq	Trans-ABySS	XLOC_056484	S1252807:0-814	cdRNA04-Dia-R3	cdRNA05-postDia	2.86933	18.6807	2.70276	5.00E-05	0.0195319
RNA-Seq	Trans-ABySS	XLOC_056689	S1254679:0-667	cdRNA03-Dia-R2	cdRNA05-postDia	10.5015	57.1716	2.44471	5.00E-05	0.0195319
RNA-Seq	Trans-ABySS	XLOC_056750	S1255476:0-886	cdRNA02-Dia-R1	cdRNA04-Dia-R3	16.4997	2.12825	-2.9547	0.0002	0.0481888
RNA-Seq	Trans-ABySS	XLOC_056750	S1255476:0-886	cdRNA02-Dia-R1	cdRNA05-postDia	16.4997	102.072	2.62908	0.0001	0.0354705
RNA-Seq	Trans-ABySS	XLOC_056750	S1255476:0-886	cdRNA01-preDia	cdRNA05-postDia	5.3501	102.072	4.25387	5.00E-05	0.0195319
RNA-Seq	Trans-ABySS	XLOC_056750	S1255476:0-886	cdRNA03-Dia-R2	cdRNA05-postDia	2.61628	102.072	5.28593	5.00E-05	0.0195319
RNA-Seq	Trans-ABySS	XLOC_056750	S1255476:0-886	cdRNA04-Dia-R3	cdRNA05-postDia	2.12825	102.072	5.58378	5.00E-05	0.0195319
RNA-Seq	Trans-ABySS	XLOC_056802	S125602:0-653	cdRNA02-Dia-R1	cdRNA05-postDia	15.3362	78.6056	2.35769	0.0001	0.0354705
RNA-Seq	Trans-ABySS	XLOC_056802	S125602:0-653	cdRNA03-Dia-R2	cdRNA05-postDia	13.0145	78.6056	2.59451	5.00E-05	0.0195319
RNA-Seq	Trans-ABySS	XLOC_057346	S1260801:0-218	cdRNA03-Dia-R2	cdRNA05-postDia	273.307	34.1051	-3.00246	5.00E-05	0.0195319
RNA-Seq	Trans-ABySS	XLOC_057346	S1260801:0-218	cdRNA04-Dia-R3	cdRNA05-postDia	262.692	34.1051	-2.94531	0.0001	0.0354705
RNA-Seq	Trans-ABySS	XLOC_057545	S1262664:0-701	cdRNA02-Dia-R1	cdRNA05-postDia	8.11029	60.6653	2.90305	5.00E-05	0.0195319
RNA-Seq	Trans-ABySS	XLOC_057545	S1262664:0-701	cdRNA03-Dia-R2	cdRNA05-postDia	7.98801	60.6653	2.92496	5.00E-05	0.0195319
RNA-Seq	Trans-ABySS	XLOC_057545	S1262664:0-701	cdRNA04-Dia-R3	cdRNA05-postDia	5.75497	60.6653	3.39799	5.00E-05	0.0195319
RNA-Seq	Trans-ABySS	XLOC_057545	S1262664:0-701	cdRNA01-preDia	cdRNA05-postDia	2.66131	60.6653	4.51066	5.00E-05	0.0195319
RNA-Seq	Trans-ABySS	XLOC_059518	S127993:0-252	cdRNA04-Dia-R3	cdRNA05-postDia	22.6657	145.663	2.68405	0.00015	0.0374403
RNA-Seq	Trans-ABySS	XLOC_059617	S1280851:0-133	cdRNA02-Dia-R1	cdRNA05-postDia	182.903	2911.82	3.99278	5.00E-05	0.0195319
RNA-Seq	Trans-ABySS	XLOC_060161	S1285732:0-962	cdRNA04-Dia-R3	cdRNA05-postDia	26.7518	4.56265	-2.55169	0.0002	0.0481888
RNA-Seq	Trans-ABySS	XLOC_060634	S1290082:2-249	cdRNA01-preDia	cdRNA05-postDia	34.6355	207.507	2.58284	0.0002	0.0481888
RNA-Seq	Trans-ABySS	XLOC_061697	S1299818:0-223	cdRNA03-Dia-R2	cdRNA05-postDia	33.9941	264.869	2.96192	5.00E-05	0.0195319
RNA-Seq	Trans-ABySS	XLOC_061697	S1299818:0-223	cdRNA04-Dia-R3	cdRNA05-postDia	27.2857	264.869	3.27906	5.00E-05	0.0195319

RNA-Seq	Trans-ABySS	XLOC_063733	S1317278:0-1086	cdRNA01-preDia	cdRNA05-postDia	7.65335	40.6196	2.40801	0.0001	0.0354705
RNA-Seq	Trans-ABySS	XLOC_063733	S1317278:0-1086	cdRNA02-Dia-R1	cdRNA05-postDia	6.58861	40.6196	2.62413	5.00E-05	0.0195319
RNA-Seq	Trans-ABySS	XLOC_064522	S132416:0-478	cdRNA01-preDia	cdRNA04-Dia-R3	69.2448	8.25735	-3.06796	5.00E-05	0.0195319
RNA-Seq	Trans-ABySS	XLOC_064522	S132416:0-478	cdRNA01-preDia	cdRNA03-Dia-R2	69.2448	9.4422	-2.87451	5.00E-05	0.0195319
RNA-Seq	Trans-ABySS	XLOC_065495	S1332848:0-462	cdRNA02-Dia-R1	cdRNA05-postDia	19.5287	169.447	3.11717	5.00E-05	0.0195319
RNA-Seq	Trans-ABySS	XLOC_065495	S1332848:0-462	cdRNA03-Dia-R2	cdRNA05-postDia	10.3735	169.447	4.02986	5.00E-05	0.0195319
RNA-Seq	Trans-ABySS	XLOC_065495	S1332848:0-462	cdRNA04-Dia-R3	cdRNA05-postDia	9.92763	169.447	4.09324	5.00E-05	0.0195319
RNA-Seq	Trans-ABySS	XLOC_065495	S1332848:0-462	cdRNA01-preDia	cdRNA05-postDia	6.01039	169.447	4.81723	5.00E-05	0.0195319
RNA-Seq	Trans-ABySS	XLOC_065634	S1334054:0-143	cdRNA02-Dia-R1	cdRNA05-postDia	170.718	1154.23	2.75724	5.00E-05	0.0195319
RNA-Seq	Trans-ABySS	XLOC_066457	S1341310:2-98	cdRNA04-Dia-R3	cdRNA05-postDia	620.582	6826.46	3.45944	0.0002	0.0481888
RNA-Seq	Trans-ABySS	XLOC_066457	S1341310:2-98	cdRNA01-preDia	cdRNA05-postDia	542.473	6826.46	3.65351	0.0002	0.0481888
RNA-Seq	Trans-ABySS	XLOC_066752	S1343884:0-578	cdRNA02-Dia-R1	cdRNA05-postDia	10.5081	61.6475	2.55255	5.00E-05	0.0195319
RNA-Seq	Trans-ABySS	XLOC_067114	S1346977:0-637	cdRNA02-Dia-R1	cdRNA05-postDia	25.2119	152.473	2.59638	0.0001	0.0354705
RNA-Seq	Trans-ABySS	XLOC_067114	S1346977:0-637	cdRNA03-Dia-R2	cdRNA05-postDia	18.2343	152.473	3.06383	5.00E-05	0.0195319
RNA-Seq	Trans-ABySS	XLOC_067114	S1346977:0-637	cdRNA04-Dia-R3	cdRNA05-postDia	15.9717	152.473	3.25497	5.00E-05	0.0195319
RNA-Seq	Trans-ABySS	XLOC_067970	S1354548:0-441	cdRNA01-preDia	cdRNA04-Dia-R3	20.0136	105.911	2.4038	0.0001	0.0354705
RNA-Seq	Trans-ABySS	XLOC_067970	S1354548:0-441	cdRNA01-preDia	cdRNA03-Dia-R2	20.0136	110.668	2.46718	0.0001	0.0354705
RNA-Seq	Trans-ABySS	XLOC_068379	S1358228:0-1797	cdRNA01-preDia	cdRNA05-postDia	24.9095	5.01017	-2.31376	5.00E-05	0.0195319
RNA-Seq	Trans-ABySS	XLOC_069254	S1366535:0-347	cdRNA01-preDia	cdRNA05-postDia	27.5769	146.679	2.41113	0.0001	0.0354705
RNA-Seq	Trans-ABySS	XLOC_069254	S1366535:0-347	cdRNA02-Dia-R1	cdRNA05-postDia	24.2783	146.679	2.59492	0.0001	0.0354705
RNA-Seq	Trans-ABySS	XLOC_070181	S1375561:0-318	cdRNA02-Dia-R1	cdRNA05-postDia	11.8418	79.3002	2.74343	0.0001	0.0354705
RNA-Seq	Trans-ABySS	XLOC_070666	S1379828:0-194	cdRNA01-preDia	cdRNA05-postDia	54.346	378.634	2.80056	5.00E-05	0.0195319
RNA-Seq	Trans-ABySS	XLOC_070666	S1379828:0-194	cdRNA03-Dia-R2	cdRNA05-postDia	53.152	378.634	2.83261	0.00015	0.0374403
RNA-Seq	Trans-ABySS	XLOC_070666	S1379828:0-194	cdRNA02-Dia-R1	cdRNA05-postDia	52.0319	378.634	2.86334	0.0002	0.0481888
RNA-Seq	Trans-ABySS	XLOC_070684	S1380031:0-1583	cdRNA04-Dia-R3	cdRNA05-postDia	6.34631	42.7001	2.75025	5.00E-05	0.0195319
RNA-Seq	Trans-ABySS	XLOC_070684	S1380031:0-1583	cdRNA03-Dia-R2	cdRNA05-postDia	6.08143	42.7001	2.81176	5.00E-05	0.0195319
RNA-Seq	Trans-ABySS	XLOC_070998	S1382809:0-656	cdRNA04-Dia-R3	cdRNA05-postDia	8.9724	46.5953	2.37662	0.0002	0.0481888
RNA-Seq	Trans-ABySS	XLOC_070998	S1382809:0-656	cdRNA02-Dia-R1	cdRNA05-postDia	5.49925	46.5953	3.08288	5.00E-05	0.0195319
RNA-Seq	Trans-ABySS	XLOC_072495	S1396318:0-168	cdRNA02-Dia-R1	cdRNA05-postDia	130.634	1100.03	3.07395	5.00E-05	0.0195319
RNA-Seq	Trans-ABySS	XLOC_072495	S1396318:0-168	cdRNA03-Dia-R2	cdRNA05-postDia	80.7579	1100.03	3.7678	5.00E-05	0.0195319
RNA-Seq	Trans-ABySS	XLOC_072495	S1396318:0-168	cdRNA04-Dia-R3	cdRNA05-postDia	71.5617	1100.03	3.94222	5.00E-05	0.0195319
RNA-Seq	Trans-ABySS	XLOC_073725	S1406973:0-535	cdRNA03-Dia-R2	cdRNA05-postDia	6.06434	41.3916	2.77092	5.00E-05	0.0195319
RNA-Seq	Trans-ABySS	XLOC_073725	S1406973:0-535	cdRNA04-Dia-R3	cdRNA05-postDia	5.80368	41.3916	2.8343	0.0001	0.0354705
RNA-Seq	Trans-ABySS	XLOC_073725	S1406973:0-535	cdRNA01-preDia	cdRNA05-postDia	2.48023	41.3916	4.06079	0.00015	0.0374403
RNA-Seq	Trans-ABySS	XLOC_074735	S1415054:0-540	cdRNA02-Dia-R1	cdRNA05-postDia	16.5949	106.492	2.68193	5.00E-05	0.0195319
RNA-Seq	Trans-ABySS	XLOC_074735	S1415054:0-540	cdRNA03-Dia-R2	cdRNA05-postDia	13.9396	106.492	2.93349	5.00E-05	0.0195319
RNA-Seq	Trans-ABySS	XLOC_074735	S1415054:0-540	cdRNA04-Dia-R3	cdRNA05-postDia	13.3404	106.492	2.99687	5.00E-05	0.0195319
RNA-Seq	Trans-ABySS	XLOC_074874	S1416294:0-175	cdRNA03-Dia-R2	cdRNA05-postDia	84.8612	843.514	3.31323	5.00E-05	0.0195319
RNA-Seq	Trans-ABySS	XLOC_074874	S1416294:0-175	cdRNA04-Dia-R3	cdRNA05-postDia	59.0644	843.514	3.83605	0.0001	0.0354705
RNA-Seq	Trans-ABySS	XLOC_074874	S1416294:0-175	cdRNA02-Dia-R1	cdRNA05-postDia	58.0929	843.514	3.85998	0.00015	0.0374403
RNA-Seq	Trans-ABySS	XLOC_074874	S1416294:0-175	cdRNA01-preDia	cdRNA05-postDia	49.9571	843.514	4.07765	0.0001	0.0354705
RNA-Seq	Trans-ABySS	XLOC_075417	S1421424:0-572	cdRNA03-Dia-R2	cdRNA05-postDia	6.54556	36.9891	2.49851	0.0002	0.0481888
RNA-Seq	Trans-ABySS	XLOC_075417	S1421424:0-572	cdRNA04-Dia-R3	cdRNA05-postDia	5.45218	36.9891	2.7622	0.00015	0.0374403
RNA-Seq	Trans-ABySS	XLOC_075417	S1421424:0-572	cdRNA02-Dia-R1	cdRNA05-postDia	4.29	36.9891	3.10805	5.00E-05	0.0195319
RNA-Seq	Trans-ABySS	XLOC_075417	S1421424:0-572	cdRNA01-preDia	cdRNA05-postDia	3.09843	36.9891	3.57749	5.00E-05	0.0195319
RNA-Seq	Trans-ABySS	XLOC_075746	S1424237:0-479	cdRNA04-Dia-R3	cdRNA05-postDia	11.0215	62.8797	2.51227	5.00E-05	0.0195319
RNA-Seq	Trans-ABySS	XLOC_075746	S1424237:0-479	cdRNA02-Dia-R1	cdRNA05-postDia	8.55004	62.8797	2.87859	5.00E-05	0.0195319

RNA-Seq	Trans-ABySS	XLOC_076714	S1432936:0-598	cdRNA01-preDia	cdRNA05-postDia	91.681	2.81338	-5.02625	5.00E-05	0.0195319
RNA-Seq	Trans-ABySS	XLOC_076714	S1432936:0-598	cdRNA01-preDia	cdRNA02-Dia-R1	91.681	3.15438	-4.86119	5.00E-05	0.0195319
RNA-Seq	Trans-ABySS	XLOC_076714	S1432936:0-598	cdRNA01-preDia	cdRNA04-Dia-R3	91.681	5.63415	-4.02435	5.00E-05	0.0195319
RNA-Seq	Trans-ABySS	XLOC_076714	S1432936:0-598	cdRNA01-preDia	cdRNA03-Dia-R2	91.681	5.8872	-3.96097	5.00E-05	0.0195319
RNA-Seq	Trans-ABySS	XLOC_076747	S1433264:0-229	cdRNA03-Dia-R2	cdRNA05-postDia	26.1145	191.687	2.87583	0.0002	0.0481888
RNA-Seq	Trans-ABySS	XLOC_077119	S1436200:0-944	cdRNA01-preDia	cdRNA03-Dia-R2	19.4766	2.89346	-2.75087	0.0001	0.0354705
RNA-Seq	Trans-ABySS	XLOC_077223	S1437041:0-251	cdRNA01-preDia	cdRNA03-Dia-R2	170.845	17.9829	-3.24799	5.00E-05	0.0195319
RNA-Seq	Trans-ABySS	XLOC_077231	S1437113:0-238	cdRNA01-preDia	cdRNA05-postDia	17.2168	233.442	3.76117	0.0001	0.0354705
RNA-Seq	Trans-ABySS	XLOC_077346	S143827:0-655	cdRNA01-preDia	cdRNA05-postDia	13.0456	92.2174	2.82147	5.00E-05	0.0195319
RNA-Seq	Trans-ABySS	XLOC_077346	S143827:0-655	cdRNA02-Dia-R1	cdRNA05-postDia	5.58593	92.2174	4.04517	5.00E-05	0.0195319
RNA-Seq	Trans-ABySS	XLOC_077346	S143827:0-655	cdRNA03-Dia-R2	cdRNA05-postDia	3.95628	92.2174	4.54282	5.00E-05	0.0195319
RNA-Seq	Trans-ABySS	XLOC_077346	S143827:0-655	cdRNA04-Dia-R3	cdRNA05-postDia	2.93433	92.2174	4.97394	5.00E-05	0.0195319
RNA-Seq	Trans-ABySS	XLOC_077894	S1443347:0-792	cdRNA01-preDia	cdRNA03-Dia-R2	16.9872	1.36553	-3.63691	0.00015	0.0374403
RNA-Seq	Trans-ABySS	XLOC_077980	S1444045:0-670	cdRNA04-Dia-R3	cdRNA05-postDia	5.58814	47.5742	3.08974	5.00E-05	0.0195319
RNA-Seq	Trans-ABySS	XLOC_077980	S1444045:0-670	cdRNA03-Dia-R2	cdRNA05-postDia	3.82894	47.5742	3.63516	5.00E-05	0.0195319
RNA-Seq	Trans-ABySS	XLOC_078235	S1446301:0-802	cdRNA01-preDia	cdRNA05-postDia	14.7964	89.1236	2.59056	0.0001	0.0354705
RNA-Seq	Trans-ABySS	XLOC_078235	S1446301:0-802	cdRNA02-Dia-R1	cdRNA05-postDia	7.41458	89.1236	3.58737	5.00E-05	0.0195319
RNA-Seq	Trans-ABySS	XLOC_078235	S1446301:0-802	cdRNA03-Dia-R2	cdRNA05-postDia	3.58053	89.1236	4.63756	5.00E-05	0.0195319
RNA-Seq	Trans-ABySS	XLOC_078235	S1446301:0-802	cdRNA04-Dia-R3	cdRNA05-postDia	2.71274	89.1236	5.03798	5.00E-05	0.0195319
RNA-Seq	Trans-ABySS	XLOC_078703	S1450467:0-716	cdRNA02-Dia-R1	cdRNA05-postDia	7.47906	39.9226	2.41628	0.00015	0.0374403
RNA-Seq	Trans-ABySS	XLOC_079926	S1461029:0-558	cdRNA01-preDia	cdRNA05-postDia	6.44193	42.2095	2.712	5.00E-05	0.0195319
RNA-Seq	Trans-ABySS	XLOC_082173	S1481014:0-515	cdRNA03-Dia-R2	cdRNA05-postDia	9.75319	57.4547	2.55848	5.00E-05	0.0195319
RNA-Seq	Trans-ABySS	XLOC_082173	S1481014:0-515	cdRNA04-Dia-R3	cdRNA05-postDia	9.05944	57.4547	2.66493	0.0001	0.0354705
RNA-Seq	Trans-ABySS	XLOC_082397	S1482963:0-54	cdRNA01-preDia	cdRNA04-Dia-R3	1.73E+06	82543	-4.39159	5.00E-05	0.0195319
RNA-Seq	Trans-ABySS	XLOC_082397	S1482963:0-54	cdRNA01-preDia	cdRNA03-Dia-R2	1.73E+06	93437.9	-4.21273	5.00E-05	0.0195319
RNA-Seq	Trans-ABySS	XLOC_082397	S1482963:0-54	cdRNA01-preDia	cdRNA02-Dia-R1	1.73E+06	316623	-2.45204	5.00E-05	0.0195319
RNA-Seq	Trans-ABySS	XLOC_082397	S1482963:0-54	cdRNA01-preDia	cdRNA05-postDia	1.73E+06	332212	-2.3827	0.0001	0.0354705
RNA-Seq	Trans-ABySS	XLOC_082543	S1484308:0-259	cdRNA02-Dia-R1	cdRNA05-postDia	29.2097	213.754	2.87144	5.00E-05	0.0195319
RNA-Seq	Trans-ABySS	XLOC_082543	S1484308:0-259	cdRNA01-preDia	cdRNA05-postDia	10.4372	213.754	4.35614	0.00015	0.0374403
RNA-Seq	Trans-ABySS	XLOC_082796	S1486602:0-838	cdRNA03-Dia-R2	cdRNA05-postDia	3.37722	27.4149	3.02105	0.0001	0.0354705
RNA-Seq	Trans-ABySS	XLOC_082796	S1486602:0-838	cdRNA04-Dia-R3	cdRNA05-postDia	1.88537	27.4149	3.86204	5.00E-05	0.0195319
RNA-Seq	Trans-ABySS	XLOC_083167	S1489694:0-340	cdRNA01-preDia	cdRNA05-postDia	20.9529	141.939	2.76005	5.00E-05	0.0195319
RNA-Seq	Trans-ABySS	XLOC_083167	S1489694:0-340	cdRNA04-Dia-R3	cdRNA05-postDia	19.6117	141.939	2.85549	0.00015	0.0374403
RNA-Seq	Trans-ABySS	XLOC_083167	S1489694:0-340	cdRNA03-Dia-R2	cdRNA05-postDia	18.5409	141.939	2.93649	5.00E-05	0.0195319
RNA-Seq	Trans-ABySS	XLOC_083267	S1490525:0-651	cdRNA04-Dia-R3	cdRNA05-postDia	5.06164	38.7843	2.93779	5.00E-05	0.0195319
RNA-Seq	Trans-ABySS	XLOC_083267	S1490525:0-651	cdRNA03-Dia-R2	cdRNA05-postDia	4.09148	38.7843	3.24478	5.00E-05	0.0195319
RNA-Seq	Trans-ABySS	XLOC_084538	S1501937:0-275	cdRNA01-preDia	cdRNA04-Dia-R3	51.8394	281.859	2.44285	5.00E-05	0.0195319
RNA-Seq	Trans-ABySS	XLOC_084538	S1501937:0-275	cdRNA01-preDia	cdRNA03-Dia-R2	51.8394	312.178	2.59025	5.00E-05	0.0195319
RNA-Seq	Trans-ABySS	XLOC_085723	S1513084:10-326	cdRNA04-Dia-R3	cdRNA05-postDia	11.1909	99.5724	3.15342	0.00015	0.0374403
RNA-Seq	Trans-ABySS	XLOC_090466	S155526:0-1206	cdRNA02-Dia-R1	cdRNA05-postDia	4.5592	41.5962	3.1896	5.00E-05	0.0195319
RNA-Seq	Trans-ABySS	XLOC_090466	S155526:0-1206	cdRNA03-Dia-R2	cdRNA05-postDia	4.31832	41.5962	3.26791	5.00E-05	0.0195319
RNA-Seq	Trans-ABySS	XLOC_090466	S155526:0-1206	cdRNA04-Dia-R3	cdRNA05-postDia	3.62144	41.5962	3.52182	5.00E-05	0.0195319
RNA-Seq	Trans-ABySS	XLOC_090548	S1555939:0-369	cdRNA02-Dia-R1	cdRNA05-postDia	13.4052	83.9172	2.64617	0.0001	0.0354705
RNA-Seq	Trans-ABySS	XLOC_090548	S1555939:0-369	cdRNA04-Dia-R3	cdRNA05-postDia	12.9066	83.9172	2.70085	0.0001	0.0354705
RNA-Seq	Trans-ABySS	XLOC_090548	S1555939:0-369	cdRNA03-Dia-R2	cdRNA05-postDia	13.2166	83.9172	2.66661	5.00E-05	0.0195319
RNA-Seq	Trans-ABySS	XLOC_091466	S1564474:0-621	cdRNA02-Dia-R1	cdRNA05-postDia	7.16908	46.1465	2.68636	5.00E-05	0.0195319
RNA-Seq	Trans-ABySS	XLOC_091466	S1564474:0-621	cdRNA04-Dia-R3	cdRNA05-postDia	5.83527	46.1465	2.98335	5.00E-05	0.0195319

RNA-Seq	Trans-ABySS	XLOC_091466	S1564474:0-621	cdRNA03-Dia-R2	cdRNA05-postDia	5.66947	46.1465	3.02494	5.00E-05	0.0195319
RNA-Seq	Trans-ABySS	XLOC_091466	S1564474:0-621	cdRNA01-preDia	cdRNA05-postDia	2.51561	46.1465	4.19724	5.00E-05	0.0195319
RNA-Seq	Trans-ABySS	XLOC_092038	S1570002:0-754	cdRNA04-Dia-R3	cdRNA05-postDia	24.8378	3.06344	-3.01931	5.00E-05	0.0195319
RNA-Seq	Trans-ABySS	XLOC_092038	S1570002:0-754	cdRNA03-Dia-R2	cdRNA05-postDia	23.1958	3.06344	-2.92064	0.0001	0.0354705
RNA-Seq	Trans-ABySS	XLOC_092113	S1570691:0-395	cdRNA03-Dia-R2	cdRNA05-postDia	12.6043	65.1947	2.37084	0.00015	0.0374403
RNA-Seq	Trans-ABySS	XLOC_093272	S1581330:0-619	cdRNA01-preDia	cdRNA04-Dia-R3	28.4645	5.45193	-2.38432	0.0002	0.0481888
RNA-Seq	Trans-ABySS	XLOC_093298	S1581480:0-63	cdRNA04-Dia-R3	cdRNA05-postDia	48768.2	312128	2.67813	5.00E-05	0.0195319
RNA-Seq	Trans-ABySS	XLOC_093298	S1581480:0-63	cdRNA03-Dia-R2	cdRNA05-postDia	44760.9	312128	2.80183	5.00E-05	0.0195319
RNA-Seq	Trans-ABySS	XLOC_095359	S1600634:0-517	cdRNA02-Dia-R1	cdRNA03-Dia-R2	9.55104	53.0148	2.47266	0.00015	0.0374403
RNA-Seq	Trans-ABySS	XLOC_095359	S1600634:0-517	cdRNA02-Dia-R1	cdRNA04-Dia-R3	9.55104	56.4643	2.56361	0.00015	0.0374403
RNA-Seq	Trans-ABySS	XLOC_095359	S1600634:0-517	cdRNA01-preDia	cdRNA03-Dia-R2	1.89429	53.0148	4.80667	0.0002	0.0481888
RNA-Seq	Trans-ABySS	XLOC_095359	S1600634:0-517	cdRNA01-preDia	cdRNA04-Dia-R3	1.89429	56.4643	4.89761	0.0002	0.0481888
RNA-Seq	Trans-ABySS	XLOC_097520	S162011:1-209	cdRNA03-Dia-R2	cdRNA05-postDia	38.8782	335.38	3.10876	0.0001	0.0354705
RNA-Seq	Trans-ABySS	XLOC_097520	S162011:1-209	cdRNA04-Dia-R3	cdRNA05-postDia	38.5359	335.38	3.12152	0.0001	0.0354705
RNA-Seq	Trans-ABySS	XLOC_097520	S162011:1-209	cdRNA02-Dia-R1	cdRNA05-postDia	34.504	335.38	3.28096	0.0002	0.0481888
RNA-Seq	Trans-ABySS	XLOC_097674	S1621546:0-509	cdRNA03-Dia-R2	cdRNA05-postDia	13.6013	79.3731	2.5449	0.0001	0.0354705
RNA-Seq	Trans-ABySS	XLOC_097674	S1621546:0-509	cdRNA04-Dia-R3	cdRNA05-postDia	12.7368	79.3731	2.63965	5.00E-05	0.0195319
RNA-Seq	Trans-ABySS	XLOC_097674	S1621546:0-509	cdRNA01-preDia	cdRNA05-postDia	7.92542	79.3731	3.32409	5.00E-05	0.0195319
RNA-Seq	Trans-ABySS	XLOC_097674	S1621546:0-509	cdRNA02-Dia-R1	cdRNA05-postDia	7.04831	79.3731	3.4933	0.0001	0.0354705
RNA-Seq	Trans-ABySS	XLOC_098193	S1626087:0-822	cdRNA04-Dia-R3	cdRNA05-postDia	18.0984	2.08065	-3.12075	5.00E-05	0.0195319
RNA-Seq	Trans-ABySS	XLOC_098193	S1626087:0-822	cdRNA03-Dia-R2	cdRNA05-postDia	17.2511	2.08065	-3.05158	5.00E-05	0.0195319
RNA-Seq	Trans-ABySS	XLOC_098886	S1632319:0-660	cdRNA02-Dia-R1	cdRNA05-postDia	17.6053	83.7192	2.24955	0.00015	0.0374403
RNA-Seq	Trans-ABySS	XLOC_098886	S1632319:0-660	cdRNA01-preDia	cdRNA05-postDia	14.4029	83.7192	2.5392	5.00E-05	0.0195319
RNA-Seq	Trans-ABySS	XLOC_098886	S1632319:0-660	cdRNA04-Dia-R3	cdRNA05-postDia	9.82987	83.7192	3.09031	5.00E-05	0.0195319
RNA-Seq	Trans-ABySS	XLOC_098886	S1632319:0-660	cdRNA03-Dia-R2	cdRNA05-postDia	9.0975	83.7192	3.20202	5.00E-05	0.0195319
RNA-Seq	Trans-ABySS	XLOC_099528	S1637997:0-161	cdRNA01-preDia	cdRNA05-postDia	147.077	1244.98	3.08148	5.00E-05	0.0195319
RNA-Seq	Trans-ABySS	XLOC_099528	S1637997:0-161	cdRNA04-Dia-R3	cdRNA05-postDia	94.0131	1244.98	3.72712	0.0001	0.0354705
RNA-Seq	Trans-ABySS	XLOC_099528	S1637997:0-161	cdRNA03-Dia-R2	cdRNA05-postDia	80.6935	1244.98	3.94752	5.00E-05	0.0195319
RNA-Seq	Trans-ABySS	XLOC_099528	S1637997:0-161	cdRNA02-Dia-R1	cdRNA05-postDia	60.7639	1244.98	4.35676	0.00015	0.0374403
RNA-Seq	Trans-ABySS	XLOC_100232	S1644217:1-453	cdRNA04-Dia-R3	cdRNA05-postDia	4.47543	37.9019	3.08217	0.0002	0.0481888
RNA-Seq	Trans-ABySS	XLOC_100232	S1644217:1-453	cdRNA02-Dia-R1	cdRNA05-postDia	3.65689	37.9019	3.37358	0.0002	0.0481888
RNA-Seq	Trans-ABySS	XLOC_101290	S165397:1-232	cdRNA02-Dia-R1	cdRNA05-postDia	33.0795	222.207	2.7479	0.0002	0.0481888
RNA-Seq	Trans-ABySS	XLOC_102617	S1665380:1-850	cdRNA04-Dia-R3	cdRNA05-postDia	3.11075	24.4039	2.97178	5.00E-05	0.0195319
RNA-Seq	Trans-ABySS	XLOC_102617	S1665380:1-850	cdRNA02-Dia-R1	cdRNA05-postDia	2.86429	24.4039	3.09086	5.00E-05	0.0195319
RNA-Seq	Trans-ABySS	XLOC_102617	S1665380:1-850	cdRNA03-Dia-R2	cdRNA05-postDia	2.76635	24.4039	3.14105	5.00E-05	0.0195319
RNA-Seq	Trans-ABySS	XLOC_102617	S1665380:1-850	cdRNA01-preDia	cdRNA05-postDia	1.97995	24.4039	3.62357	5.00E-05	0.0195319
RNA-Seq	Trans-ABySS	XLOC_102977	S1668651:4-825	cdRNA01-preDia	cdRNA05-postDia	3.99176	24.3615	2.60951	0.00015	0.0374403
RNA-Seq	Trans-ABySS	XLOC_103326	S1671996:0-238	cdRNA02-Dia-R1	cdRNA05-postDia	44.7129	272.203	2.60592	5.00E-05	0.0195319
RNA-Seq	Trans-ABySS	XLOC_103444	S1673034:0-452	cdRNA04-Dia-R3	cdRNA05-postDia	23.7542	112.275	2.24079	0.0002	0.0481888
RNA-Seq	Trans-ABySS	XLOC_103444	S1673034:0-452	cdRNA02-Dia-R1	cdRNA05-postDia	23.2958	112.275	2.2689	5.00E-05	0.0195319
RNA-Seq	Trans-ABySS	XLOC_103444	S1673034:0-452	cdRNA01-preDia	cdRNA05-postDia	22.6202	112.275	2.31136	0.00015	0.0374403
RNA-Seq	Trans-ABySS	XLOC_103506	S1673520:1-168	cdRNA01-preDia	cdRNA04-Dia-R3	100.066	652.698	2.70546	0.0001	0.0354705
RNA-Seq	Trans-ABySS	XLOC_103506	S1673520:1-168	cdRNA01-preDia	cdRNA03-Dia-R2	100.066	688.13	2.78173	0.0001	0.0354705
RNA-Seq	Trans-ABySS	XLOC_105227	S1688711:0-255	cdRNA02-Dia-R1	cdRNA05-postDia	23.107	225.568	3.28716	0.00015	0.0374403
RNA-Seq	Trans-ABySS	XLOC_105227	S1688711:0-255	cdRNA03-Dia-R2	cdRNA05-postDia	15.6997	225.568	3.84476	0.0002	0.0481888
RNA-Seq	Trans-ABySS	XLOC_105620	S1692035:0-393	cdRNA02-Dia-R1	cdRNA05-postDia	42.0938	270.787	2.68548	5.00E-05	0.0195319
RNA-Seq	Trans-ABySS	XLOC_105904	S1694236:0-197	cdRNA02-Dia-R1	cdRNA05-postDia	121.312	768.97	2.66421	5.00E-05	0.0195319

RNA-Seq	Trans-ABySS	XLOC_105904	S1694236:0-197	cdRNA01-preDia	cdRNA05-postDia	24.0216	768.97	5.00052	5.00E-05	0.0195319
RNA-Seq	Trans-ABySS	XLOC_105947	S1694482:0-1080	cdRNA01-preDia	cdRNA05-postDia	29.4173	187.33	2.67085	5.00E-05	0.0195319
RNA-Seq	Trans-ABySS	XLOC_106017	S1694812:0-281	cdRNA01-preDia	cdRNA04-Dia-R3	292.888	48.1666	-2.60424	5.00E-05	0.0195319
RNA-Seq	Trans-ABySS	XLOC_106017	S1694812:0-281	cdRNA01-preDia	cdRNA03-Dia-R2	292.888	58.3613	-2.32726	0.0001	0.0354705
RNA-Seq	Trans-ABySS	XLOC_106078	S1695212:0-602	cdRNA01-preDia	cdRNA05-postDia	50.5366	10.6948	-2.24042	0.0001	0.0354705
RNA-Seq	Trans-ABySS	XLOC_106094	S1695297:0-445	cdRNA04-Dia-R3	cdRNA05-postDia	22.8499	109.833	2.26505	0.0002	0.0481888
RNA-Seq	Trans-ABySS	XLOC_106094	S1695297:0-445	cdRNA03-Dia-R2	cdRNA05-postDia	19.4341	109.833	2.49865	5.00E-05	0.0195319
RNA-Seq	Trans-ABySS	XLOC_106102	S1695328:0-1150	cdRNA01-preDia	cdRNA05-postDia	8.87127	84.0861	3.24466	5.00E-05	0.0195319
RNA-Seq	Trans-ABySS	XLOC_106102	S1695328:0-1150	cdRNA01-preDia	cdRNA04-Dia-R3	8.87127	87.0504	3.29464	5.00E-05	0.0195319
RNA-Seq	Trans-ABySS	XLOC_106102	S1695328:0-1150	cdRNA01-preDia	cdRNA03-Dia-R2	8.87127	90.8659	3.35653	5.00E-05	0.0195319
RNA-Seq	Trans-ABySS	XLOC_106252	S1696219:0-682	cdRNA01-preDia	cdRNA03-Dia-R2	15.8392	105.639	2.73757	5.00E-05	0.0195319
RNA-Seq	Trans-ABySS	XLOC_106252	S1696219:0-682	cdRNA01-preDia	cdRNA04-Dia-R3	15.8392	111.726	2.81839	5.00E-05	0.0195319
RNA-Seq	Trans-ABySS	XLOC_106545	S1697919:0-1463	cdRNA01-preDia	cdRNA03-Dia-R2	161.102	32.4904	-2.30989	0.0002	0.0481888
RNA-Seq	Trans-ABySS	XLOC_106582	S1698217:0-980	cdRNA03-Dia-R2	cdRNA05-postDia	27.19	5.25676	-2.37083	0.0002	0.0481888
RNA-Seq	Trans-ABySS	XLOC_106582	S1698217:0-980	cdRNA04-Dia-R3	cdRNA05-postDia	26.0763	5.25676	-2.31049	0.0002	0.0481888
RNA-Seq	Trans-ABySS	XLOC_106717	S1698907:0-457	cdRNA04-Dia-R3	cdRNA05-postDia	89.5958	13.8447	-2.6941	5.00E-05	0.0195319
RNA-Seq	Trans-ABySS	XLOC_106717	S1698907:0-457	cdRNA03-Dia-R2	cdRNA05-postDia	82.8651	13.8447	-2.58143	0.00015	0.0374403
RNA-Seq	Trans-ABySS	XLOC_106794	S1699295:0-592	cdRNA01-preDia	cdRNA05-postDia	3.17382	36.3396	3.51725	5.00E-05	0.0195319
RNA-Seq	Trans-ABySS	XLOC_106840	S1699561:0-359	cdRNA04-Dia-R3	cdRNA05-postDia	62.0171	10.2605	-2.59556	0.00015	0.0374403
RNA-Seq	Trans-ABySS	XLOC_106910	S1700042:0-4296	cdRNA03-Dia-R2	cdRNA05-postDia	4.66577	26.992	2.53235	0.0001	0.0354705
RNA-Seq	Trans-ABySS	XLOC_106910	S1700042:0-4296	cdRNA04-Dia-R3	cdRNA05-postDia	4.1105	26.992	2.71515	5.00E-05	0.0195319
RNA-Seq	Trans-ABySS	XLOC_106942	S1700233:0-1961	cdRNA04-Dia-R3	cdRNA05-postDia	84.5066	14.0313	-2.59041	0.0001	0.0354705
RNA-Seq	Trans-ABySS	XLOC_106956	S1700298:0-454	cdRNA01-preDia	cdRNA04-Dia-R3	23.1689	127.212	2.45698	5.00E-05	0.0195319
RNA-Seq	Trans-ABySS	XLOC_106956	S1700298:0-454	cdRNA01-preDia	cdRNA03-Dia-R2	23.1689	133.461	2.52616	5.00E-05	0.0195319
RNA-Seq	Trans-ABySS	XLOC_107058	S1700935:0-579	cdRNA03-Dia-R2	cdRNA05-postDia	104.439	17.972	-2.53884	5.00E-05	0.0195319
RNA-Seq	Trans-ABySS	XLOC_107128	S1701343:0-460	cdRNA03-Dia-R2	cdRNA05-postDia	20.5606	129.377	2.65363	5.00E-05	0.0195319
RNA-Seq	Trans-ABySS	XLOC_107151	S1701500:0-1075	cdRNA01-preDia	cdRNA03-Dia-R2	87.6588	14.4937	-2.59648	0.00015	0.0374403
RNA-Seq	Trans-ABySS	XLOC_107179	S1701667:0-1260	cdRNA04-Dia-R3	cdRNA05-postDia	16.777	92.4586	2.46232	0.00015	0.0374403
RNA-Seq	Trans-ABySS	XLOC_107179	S1701667:0-1260	cdRNA01-preDia	cdRNA05-postDia	11.6616	92.4586	2.98704	5.00E-05	0.0195319
RNA-Seq	Trans-ABySS	XLOC_107210	S1701826:0-325	cdRNA02-Dia-R1	cdRNA05-postDia	36.0906	398.442	3.46468	5.00E-05	0.0195319
RNA-Seq	Trans-ABySS	XLOC_107210	S1701826:0-325	cdRNA04-Dia-R3	cdRNA05-postDia	35.4432	398.442	3.49079	5.00E-05	0.0195319
RNA-Seq	Trans-ABySS	XLOC_107210	S1701826:0-325	cdRNA03-Dia-R2	cdRNA05-postDia	31.9518	398.442	3.6404	5.00E-05	0.0195319
RNA-Seq	Trans-ABySS	XLOC_107210	S1701826:0-325	cdRNA01-preDia	cdRNA05-postDia	31.1846	398.442	3.67546	5.00E-05	0.0195319
RNA-Seq	Trans-ABySS	XLOC_107321	S1702411:1-218	cdRNA04-Dia-R3	cdRNA05-postDia	60.9496	407.298	2.7404	5.00E-05	0.0195319
RNA-Seq	Trans-ABySS	XLOC_107321	S1702411:1-218	cdRNA03-Dia-R2	cdRNA05-postDia	51.6706	407.298	2.97867	0.0001	0.0354705
RNA-Seq	Trans-ABySS	XLOC_107321	S1702411:1-218	cdRNA02-Dia-R1	cdRNA05-postDia	21.7167	407.298	4.22921	0.00015	0.0374403
RNA-Seq	Trans-ABySS	XLOC_107468	S1703181:0-585	cdRNA04-Dia-R3	cdRNA05-postDia	12.9973	67.2647	2.37163	0.0002	0.0481888
RNA-Seq	Trans-ABySS	XLOC_107468	S1703181:0-585	cdRNA02-Dia-R1	cdRNA05-postDia	12.9599	67.2647	2.3758	0.0002	0.0481888
RNA-Seq	Trans-ABySS	XLOC_107468	S1703181:0-585	cdRNA03-Dia-R2	cdRNA05-postDia	12.5274	67.2647	2.42476	0.00015	0.0374403
RNA-Seq	Trans-ABySS	XLOC_107551	S1703652:0-132	cdRNA01-preDia	cdRNA05-postDia	527.407	3694.6	2.80843	5.00E-05	0.0195319
RNA-Seq	Trans-ABySS	XLOC_107551	S1703652:0-132	cdRNA02-Dia-R1	cdRNA05-postDia	456.968	3694.6	3.01525	0.00015	0.0374403
RNA-Seq	Trans-ABySS	XLOC_107555	S1703664:0-1083	cdRNA02-Dia-R1	cdRNA05-postDia	6.15253	34.2006	2.47477	0.0001	0.0354705
RNA-Seq	Trans-ABySS	XLOC_107555	S1703664:0-1083	cdRNA04-Dia-R3	cdRNA05-postDia	3.49682	34.2006	3.28991	5.00E-05	0.0195319
RNA-Seq	Trans-ABySS	XLOC_107555	S1703664:0-1083	cdRNA03-Dia-R2	cdRNA05-postDia	2.68966	34.2006	3.66853	5.00E-05	0.0195319
RNA-Seq	Trans-ABySS	XLOC_107990	S1706240:0-1111	cdRNA01-preDia	cdRNA03-Dia-R2	9.85615	82.5759	3.06662	5.00E-05	0.0195319
RNA-Seq	Trans-ABySS	XLOC_107990	S1706240:0-1111	cdRNA01-preDia	cdRNA04-Dia-R3	9.85615	82.6037	3.06711	5.00E-05	0.0195319
RNA-Seq	Trans-ABySS	XLOC_108093	S1706776:0-544	cdRNA02-Dia-R1	cdRNA05-postDia	17.5852	94.2848	2.42266	0.0001	0.0354705

RNA-Seq	Trans-ABySS	XLOC_108124	S1706995:0-651	cdRNA01-preDia	cdRNA05-postDia	9.79526	82.7266	3.07819	5.00E-05	0.0195319
RNA-Seq	Trans-ABySS	XLOC_108519	S1709352:0-384	cdRNA01-preDia	cdRNA05-postDia	27.5894	291.833	3.40296	5.00E-05	0.0195319
RNA-Seq	Trans-ABySS	XLOC_108519	S1709352:0-384	cdRNA04-Dia-R3	cdRNA05-postDia	22.5066	291.833	3.69672	5.00E-05	0.0195319
RNA-Seq	Trans-ABySS	XLOC_108519	S1709352:0-384	cdRNA03-Dia-R2	cdRNA05-postDia	22.2797	291.833	3.71134	5.00E-05	0.0195319
RNA-Seq	Trans-ABySS	XLOC_108519	S1709352:0-384	cdRNA02-Dia-R1	cdRNA05-postDia	16.5907	291.833	4.1367	5.00E-05	0.0195319
RNA-Seq	Trans-ABySS	XLOC_108561	S1709570:0-175	cdRNA04-Dia-R3	cdRNA05-postDia	123.051	935.533	2.92653	5.00E-05	0.0195319
RNA-Seq	Trans-ABySS	XLOC_108561	S1709570:0-175	cdRNA02-Dia-R1	cdRNA05-postDia	110.377	935.533	3.08335	5.00E-05	0.0195319
RNA-Seq	Trans-ABySS	XLOC_108561	S1709570:0-175	cdRNA03-Dia-R2	cdRNA05-postDia	74.575	935.533	3.64902	5.00E-05	0.0195319
RNA-Seq	Trans-ABySS	XLOC_108591	S1709750:0-182	cdRNA01-preDia	cdRNA04-Dia-R3	879.391	142.87	-2.6218	5.00E-05	0.0195319
RNA-Seq	Trans-ABySS	XLOC_108679	S1710175:0-500	cdRNA02-Dia-R1	cdRNA05-postDia	7.03565	54.3745	2.95017	0.00015	0.0374403
RNA-Seq	Trans-ABySS	XLOC_108900	S1711416:0-240	cdRNA01-preDia	cdRNA05-postDia	217.902	31.7322	-2.77966	5.00E-05	0.0195319
RNA-Seq	Trans-ABySS	XLOC_109017	S1712062:0-409	cdRNA01-preDia	cdRNA05-postDia	18.6844	92.5505	2.3084	0.0002	0.0481888
RNA-Seq	Trans-ABySS	XLOC_109079	S1712353:0-181	cdRNA01-preDia	cdRNA05-postDia	320.752	2155.62	2.74857	5.00E-05	0.0195319
RNA-Seq	Trans-ABySS	XLOC_109114	S1712500:0-849	cdRNA01-preDia	cdRNA03-Dia-R2	30.4771	225.873	2.88971	5.00E-05	0.0195319
RNA-Seq	Trans-ABySS	XLOC_109114	S1712500:0-849	cdRNA01-preDia	cdRNA04-Dia-R3	30.4771	229.004	2.90958	5.00E-05	0.0195319
RNA-Seq	Trans-ABySS	XLOC_109244	S1713280:5-550	cdRNA01-preDia	cdRNA04-Dia-R3	289.435	19.2791	-3.90813	5.00E-05	0.0195319
RNA-Seq	Trans-ABySS	XLOC_109244	S1713280:5-550	cdRNA01-preDia	cdRNA03-Dia-R2	289.435	20.6682	-3.80776	5.00E-05	0.0195319
RNA-Seq	Trans-ABySS	XLOC_109244	S1713280:5-550	cdRNA01-preDia	cdRNA02-Dia-R1	289.435	34.3778	-3.07369	5.00E-05	0.0195319
RNA-Seq	Trans-ABySS	XLOC_109244	S1713280:5-550	cdRNA01-preDia	cdRNA05-postDia	289.435	38.3575	-2.91566	5.00E-05	0.0195319
RNA-Seq	Trans-ABySS	XLOC_109276	S1713448:0-864	cdRNA01-preDia	cdRNA02-Dia-R1	37.7316	336.82	3.15813	5.00E-05	0.0195319
RNA-Seq	Trans-ABySS	XLOC_109276	S1713448:0-864	cdRNA01-preDia	cdRNA05-postDia	37.7316	636.204	4.07565	5.00E-05	0.0195319
RNA-Seq	Trans-ABySS	XLOC_109339	S1713801:0-662	cdRNA01-preDia	cdRNA02-Dia-R1	126.869	5.20719	-4.60669	5.00E-05	0.0195319
RNA-Seq	Trans-ABySS	XLOC_109339	S1713801:0-662	cdRNA01-preDia	cdRNA03-Dia-R2	126.869	14.5119	-3.12803	5.00E-05	0.0195319
RNA-Seq	Trans-ABySS	XLOC_109339	S1713801:0-662	cdRNA01-preDia	cdRNA04-Dia-R3	126.869	16.2184	-2.96764	5.00E-05	0.0195319
RNA-Seq	Trans-ABySS	XLOC_109830	S1716413:0-770	cdRNA02-Dia-R1	cdRNA05-postDia	3.62015	22.8747	2.65963	0.0002	0.0481888
RNA-Seq	Trans-ABySS	XLOC_109932	S1716961:6-469	cdRNA01-preDia	cdRNA04-Dia-R3	14.968	97.5744	2.70463	5.00E-05	0.0195319
RNA-Seq	Trans-ABySS	XLOC_109932	S1716961:6-469	cdRNA01-preDia	cdRNA03-Dia-R2	14.968	98.6846	2.72095	5.00E-05	0.0195319
RNA-Seq	Trans-ABySS	XLOC_109963	S1717128:0-1245	cdRNA01-preDia	cdRNA05-postDia	9.85805	61.5901	2.64332	0.0001	0.0354705
RNA-Seq	Trans-ABySS	XLOC_110054	S1717706:0-431	cdRNA01-preDia	cdRNA05-postDia	5.62559	161.138	4.84015	5.00E-05	0.0195319
RNA-Seq	Trans-ABySS	XLOC_110238	S1718818:0-156	cdRNA02-Dia-R1	cdRNA05-postDia	145.893	1151.55	2.98059	5.00E-05	0.0195319
RNA-Seq	Trans-ABySS	XLOC_110238	S1718818:0-156	cdRNA04-Dia-R3	cdRNA05-postDia	98.3843	1151.55	3.54901	5.00E-05	0.0195319
RNA-Seq	Trans-ABySS	XLOC_110238	S1718818:0-156	cdRNA03-Dia-R2	cdRNA05-postDia	94.8952	1151.55	3.6011	5.00E-05	0.0195319
RNA-Seq	Trans-ABySS	XLOC_110270	S1719031:0-325	cdRNA02-Dia-R1	cdRNA05-postDia	12.3036	151.22	3.6195	5.00E-05	0.0195319
RNA-Seq	Trans-ABySS	XLOC_110270	S1719031:0-325	cdRNA04-Dia-R3	cdRNA05-postDia	11.8144	151.22	3.67803	5.00E-05	0.0195319
RNA-Seq	Trans-ABySS	XLOC_110270	S1719031:0-325	cdRNA03-Dia-R2	cdRNA05-postDia	7.98796	151.22	4.24268	0.0001	0.0354705
RNA-Seq	Trans-ABySS	XLOC_110454	S1720116:0-2048	cdRNA01-preDia	cdRNA03-Dia-R2	212.716	16.1353	-3.72063	5.00E-05	0.0195319
RNA-Seq	Trans-ABySS	XLOC_110454	S1720116:0-2048	cdRNA01-preDia	cdRNA04-Dia-R3	212.716	16.1826	-3.71641	5.00E-05	0.0195319
RNA-Seq	Trans-ABySS	XLOC_110541	S1720621:0-1044	cdRNA02-Dia-R1	cdRNA03-Dia-R2	17.1152	3.02695	-2.49934	5.00E-05	0.0195319
RNA-Seq	Trans-ABySS	XLOC_110541	S1720621:0-1044	cdRNA02-Dia-R1	cdRNA05-postDia	17.1152	112.116	2.71165	5.00E-05	0.0195319
RNA-Seq	Trans-ABySS	XLOC_110541	S1720621:0-1044	cdRNA01-preDia	cdRNA05-postDia	4.1266	112.116	4.76389	5.00E-05	0.0195319
RNA-Seq	Trans-ABySS	XLOC_110541	S1720621:0-1044	cdRNA04-Dia-R3	cdRNA05-postDia	3.25259	112.116	5.10726	5.00E-05	0.0195319
RNA-Seq	Trans-ABySS	XLOC_110541	S1720621:0-1044	cdRNA03-Dia-R2	cdRNA05-postDia	3.02695	112.116	5.21098	5.00E-05	0.0195319
RNA-Seq	Trans-ABySS	XLOC_110584	S1720908:0-595	cdRNA02-Dia-R1	cdRNA05-postDia	12.7143	68.266	2.42471	0.0001	0.0354705
RNA-Seq	Trans-ABySS	XLOC_110588	S1720914:0-448	cdRNA02-Dia-R1	cdRNA05-postDia	22.9893	185.347	3.01119	5.00E-05	0.0195319
RNA-Seq	Trans-ABySS	XLOC_110588	S1720914:0-448	cdRNA03-Dia-R2	cdRNA05-postDia	12.7968	185.347	3.85638	5.00E-05	0.0195319
RNA-Seq	Trans-ABySS	XLOC_110588	S1720914:0-448	cdRNA04-Dia-R3	cdRNA05-postDia	9.7974	185.347	4.24169	5.00E-05	0.0195319
RNA-Seq	Trans-ABySS	XLOC_110682	S1721434:0-709	cdRNA01-preDia	cdRNA05-postDia	3.34167	38.3688	3.52129	5.00E-05	0.0195319

RNA-Seq	Trans-ABySS	XLOC_110768	S1721917:5-809	cdRNA02-Dia-R1	cdRNA05-postDia	31.239	355.824	3.50974	5.00E-05	0.0195319
RNA-Seq	Trans-ABySS	XLOC_110768	S1721917:5-809	cdRNA03-Dia-R2	cdRNA05-postDia	15.0922	355.824	4.55929	5.00E-05	0.0195319
RNA-Seq	Trans-ABySS	XLOC_110768	S1721917:5-809	cdRNA04-Dia-R3	cdRNA05-postDia	15.0838	355.824	4.56009	5.00E-05	0.0195319
RNA-Seq	Trans-ABySS	XLOC_110768	S1721917:5-809	cdRNA01-preDia	cdRNA05-postDia	12.5426	355.824	4.82625	5.00E-05	0.0195319
RNA-Seq	Trans-ABySS	XLOC_110843	S1722329:0-419	cdRNA04-Dia-R3	cdRNA05-postDia	10.3239	64.5428	2.64427	0.00015	0.0374403
RNA-Seq	Trans-ABySS	XLOC_110843	S1722329:0-419	cdRNA03-Dia-R2	cdRNA05-postDia	8.09068	64.5428	2.99592	5.00E-05	0.0195319
RNA-Seq	Trans-ABySS	XLOC_110879	S1722504:0-1973	cdRNA01-preDia	cdRNA04-Dia-R3	10.0296	57.7253	2.52494	0.0001	0.0354705
RNA-Seq	Trans-ABySS	XLOC_110879	S1722504:0-1973	cdRNA01-preDia	cdRNA03-Dia-R2	10.0296	58.8554	2.55291	5.00E-05	0.0195319
RNA-Seq	Trans-ABySS	XLOC_110946	S1722870:0-1238	cdRNA01-preDia	cdRNA04-Dia-R3	13.0125	59.907	2.20282	0.00015	0.0374403
RNA-Seq	Trans-ABySS	XLOC_111077	S1723669:0-2780	cdRNA04-Dia-R3	cdRNA05-postDia	141.363	23.3405	-2.5985	0.0001	0.0354705
RNA-Seq	Trans-ABySS	XLOC_111077	S1723669:0-2780	cdRNA03-Dia-R2	cdRNA05-postDia	139.227	23.3405	-2.57653	0.0002	0.0481888
RNA-Seq	Trans-ABySS	XLOC_111135	S1724022:0-127	cdRNA02-Dia-R1	cdRNA05-postDia	213.509	1770.26	3.0516	0.0002	0.0481888
RNA-Seq	Trans-ABySS	XLOC_111219	S1724511:1-310	cdRNA02-Dia-R1	cdRNA05-postDia	38.2573	222.938	2.54284	5.00E-05	0.0195319
RNA-Seq	Trans-ABySS	XLOC_111373	S1725431:1-928	cdRNA01-preDia	cdRNA05-postDia	3.78504	19.9313	2.39666	0.00015	0.0374403
RNA-Seq	Trans-ABySS	XLOC_111476	S1726059:0-980	cdRNA02-Dia-R1	cdRNA05-postDia	5.23768	24.2839	2.213	0.00015	0.0374403
RNA-Seq	Trans-ABySS	XLOC_111476	S1726059:0-980	cdRNA03-Dia-R2	cdRNA05-postDia	5.17356	24.2839	2.23077	0.00015	0.0374403
RNA-Seq	Trans-ABySS	XLOC_111536	S1726428:0-409	cdRNA04-Dia-R3	cdRNA05-postDia	14.7819	79.5762	2.4285	0.0001	0.0354705
RNA-Seq	Trans-ABySS	XLOC_111557	S1726557:0-717	cdRNA01-preDia	cdRNA03-Dia-R2	16.0033	104.344	2.70491	5.00E-05	0.0195319
RNA-Seq	Trans-ABySS	XLOC_111557	S1726557:0-717	cdRNA01-preDia	cdRNA04-Dia-R3	16.0033	105.851	2.72559	5.00E-05	0.0195319
RNA-Seq	Trans-ABySS	XLOC_111573	S1726671:0-581	cdRNA01-preDia	cdRNA03-Dia-R2	19.2356	90.3969	2.23249	0.0002	0.0481888
RNA-Seq	Trans-ABySS	XLOC_111679	S1727320:0-543	cdRNA01-preDia	cdRNA05-postDia	54.892	7.58596	-2.85519	5.00E-05	0.0195319
RNA-Seq	Trans-ABySS	XLOC_111734	S1727627:0-230	cdRNA02-Dia-R1	cdRNA05-postDia	104.385	1732.2	4.05262	5.00E-05	0.0195319
RNA-Seq	Trans-ABySS	XLOC_111734	S1727627:0-230	cdRNA01-preDia	cdRNA05-postDia	33.4092	1732.2	5.69622	5.00E-05	0.0195319
RNA-Seq	Trans-ABySS	XLOC_111734	S1727627:0-230	cdRNA03-Dia-R2	cdRNA05-postDia	30.6948	1732.2	5.81847	5.00E-05	0.0195319
RNA-Seq	Trans-ABySS	XLOC_111734	S1727627:0-230	cdRNA04-Dia-R3	cdRNA05-postDia	25.5851	1732.2	6.08116	5.00E-05	0.0195319
RNA-Seq	Trans-ABySS	XLOC_111855	S1728259:0-509	cdRNA02-Dia-R1	cdRNA05-postDia	15.308	83.4435	2.44651	5.00E-05	0.0195319
RNA-Seq	Trans-ABySS	XLOC_111923	S1728693:0-1227	cdRNA03-Dia-R2	cdRNA05-postDia	8.85179	52.7483	2.57508	5.00E-05	0.0195319
RNA-Seq	Trans-ABySS	XLOC_111923	S1728693:0-1227	cdRNA04-Dia-R3	cdRNA05-postDia	8.38785	52.7483	2.65275	0.0001	0.0354705
RNA-Seq	Trans-ABySS	XLOC_112109	S1729769:0-859	cdRNA04-Dia-R3	cdRNA05-postDia	5.27923	37.0962	2.81287	5.00E-05	0.0195319
RNA-Seq	Trans-ABySS	XLOC_112438	S1731519:10-375	cdRNA03-Dia-R2	cdRNA05-postDia	124.057	23.344	-2.40988	0.0002	0.0481888
RNA-Seq	Trans-ABySS	XLOC_112438	S1731519:10-375	cdRNA04-Dia-R3	cdRNA05-postDia	121.897	23.344	-2.38454	0.0002	0.0481888
RNA-Seq	Trans-ABySS	XLOC_112567	S1732232:0-186	cdRNA01-preDia	cdRNA04-Dia-R3	105.296	652.443	2.6314	0.00015	0.0374403
RNA-Seq	Trans-ABySS	XLOC_112567	S1732232:0-186	cdRNA01-preDia	cdRNA03-Dia-R2	105.296	694.105	2.7207	5.00E-05	0.0195319
RNA-Seq	Trans-ABySS	XLOC_112597	S1732410:0-265	cdRNA02-Dia-R1	cdRNA05-postDia	75.5766	357.374	2.24142	0.0001	0.0354705
RNA-Seq	Trans-ABySS	XLOC_112597	S1732410:0-265	cdRNA04-Dia-R3	cdRNA05-postDia	58.9678	357.374	2.59944	5.00E-05	0.0195319
RNA-Seq	Trans-ABySS	XLOC_112597	S1732410:0-265	cdRNA03-Dia-R2	cdRNA05-postDia	45.1006	357.374	2.98622	5.00E-05	0.0195319
RNA-Seq	Trans-ABySS	XLOC_112699	S1732930:0-300	cdRNA03-Dia-R2	cdRNA05-postDia	30.8246	169.183	2.45643	0.0002	0.0481888
RNA-Seq	Trans-ABySS	XLOC_112699	S1732930:0-300	cdRNA01-preDia	cdRNA05-postDia	19.6411	169.183	3.10664	5.00E-05	0.0195319
RNA-Seq	Trans-ABySS	XLOC_112749	S1733205:0-1092	cdRNA04-Dia-R3	cdRNA05-postDia	77.9771	12.6828	-2.62018	5.00E-05	0.0195319
RNA-Seq	Trans-ABySS	XLOC_112749	S1733205:0-1092	cdRNA03-Dia-R2	cdRNA05-postDia	76.3053	12.6828	-2.58892	0.0001	0.0354705
RNA-Seq	Trans-ABySS	XLOC_112913	S1734056:0-650	cdRNA02-Dia-R1	cdRNA05-postDia	7.30545	43.7426	2.58199	0.0001	0.0354705
RNA-Seq	Trans-ABySS	XLOC_112934	S1734191:0-463	cdRNA04-Dia-R3	cdRNA05-postDia	92.1353	15.0647	-2.61258	0.0001	0.0354705
RNA-Seq	Trans-ABySS	XLOC_113006	S1734642:0-1168	cdRNA03-Dia-R2	cdRNA05-postDia	110.433	14.523	-2.92676	5.00E-05	0.0195319
RNA-Seq	Trans-ABySS	XLOC_113006	S1734642:0-1168	cdRNA04-Dia-R3	cdRNA05-postDia	105.883	14.523	-2.86606	5.00E-05	0.0195319
RNA-Seq	Trans-ABySS	XLOC_113053	S1734929:0-222	cdRNA02-Dia-R1	cdRNA05-postDia	55.3224	1000.23	4.17633	5.00E-05	0.0195319
RNA-Seq	Trans-ABySS	XLOC_113053	S1734929:0-222	cdRNA01-preDia	cdRNA05-postDia	26.1774	1000.23	5.25587	5.00E-05	0.0195319
RNA-Seq	Trans-ABySS	XLOC_113053	S1734929:0-222	cdRNA03-Dia-R2	cdRNA05-postDia	14.4709	1000.23	6.11104	0.0001	0.0354705

RNA-Seq	Trans-ABySS	XLOC_113053	S1734929:0-222	cdRNA04-Dia-R3	cdRNA05-postDia	13.8488	1000.23	6.17443	0.0002	0.0481888
RNA-Seq	Trans-ABySS	XLOC_113093	S1735220:0-189	cdRNA01-preDia	cdRNA03-Dia-R2	868.876	161.401	-2.4285	0.0001	0.0354705
RNA-Seq	Trans-ABySS	XLOC_113115	S1735338:0-290	cdRNA03-Dia-R2	cdRNA05-postDia	651.765	141.747	-2.20103	0.0002	0.0481888
RNA-Seq	Trans-ABySS	XLOC_113329	S1736503:0-238	cdRNA02-Dia-R1	cdRNA05-postDia	146.151	802.513	2.45707	0.00015	0.0374403
RNA-Seq	Trans-ABySS	XLOC_113565	S1737967:0-1380	cdRNA04-Dia-R3	cdRNA05-postDia	63.346	11.4243	-2.47115	0.0002	0.0481888
RNA-Seq	Trans-ABySS	XLOC_113565	S1737967:0-1380	cdRNA03-Dia-R2	cdRNA05-postDia	61.1841	11.4243	-2.42105	0.00015	0.0374403
RNA-Seq	Trans-ABySS	XLOC_113699	S1738752:0-946	cdRNA03-Dia-R2	cdRNA05-postDia	36.0107	401.207	3.47785	5.00E-05	0.0195319
RNA-Seq	Trans-ABySS	XLOC_113699	S1738752:0-946	cdRNA04-Dia-R3	cdRNA05-postDia	33.1971	401.207	3.59522	5.00E-05	0.0195319
RNA-Seq	Trans-ABySS	XLOC_113704	S1738799:0-542	cdRNA03-Dia-R2	cdRNA05-postDia	62.6869	4.32892	-3.85608	5.00E-05	0.0195319
RNA-Seq	Trans-ABySS	XLOC_113704	S1738799:0-542	cdRNA04-Dia-R3	cdRNA05-postDia	45.5942	4.32892	-3.39677	5.00E-05	0.0195319
RNA-Seq	Trans-ABySS	XLOC_113704	S1738799:0-542	cdRNA01-preDia	cdRNA03-Dia-R2	8.63598	62.6869	2.85973	0.0002	0.0481888
RNA-Seq	Trans-ABySS	XLOC_113733	S1738954:0-297	cdRNA04-Dia-R3	cdRNA05-postDia	23.7219	129.58	2.44955	0.0001	0.0354705
RNA-Seq	Trans-ABySS	XLOC_113804	S1739405:0-1637	cdRNA04-Dia-R3	cdRNA05-postDia	9.80125	57.119	2.54293	0.0002	0.0481888
RNA-Seq	Trans-ABySS	XLOC_113804	S1739405:0-1637	cdRNA03-Dia-R2	cdRNA05-postDia	9.55662	57.119	2.5794	5.00E-05	0.0195319
RNA-Seq	Trans-ABySS	XLOC_113845	S1739687:4-207	cdRNA02-Dia-R1	cdRNA05-postDia	44.364	310.822	2.80863	0.00015	0.0374403
RNA-Seq	Trans-ABySS	XLOC_113845	S1739687:4-207	cdRNA01-preDia	cdRNA05-postDia	38.6143	310.822	3.00888	0.0001	0.0354705
RNA-Seq	Trans-ABySS	XLOC_114018	S1740943:0-247	cdRNA04-Dia-R3	cdRNA05-postDia	707.929	104.145	-2.76501	5.00E-05	0.0195319
RNA-Seq	Trans-ABySS	XLOC_114018	S1740943:0-247	cdRNA03-Dia-R2	cdRNA05-postDia	631.011	104.145	-2.59907	5.00E-05	0.0195319
RNA-Seq	Trans-ABySS	XLOC_114018	S1740943:0-247	cdRNA01-preDia	cdRNA05-postDia	546.114	104.145	-2.39061	0.0001	0.0354705
RNA-Seq	Trans-ABySS	XLOC_114105	S1741579:0-576	cdRNA01-preDia	cdRNA05-postDia	6.25284	54.2316	3.11655	5.00E-05	0.0195319
RNA-Seq	Trans-ABySS	XLOC_114107	S1741600:0-316	cdRNA01-preDia	cdRNA05-postDia	6.37664	83.2999	3.70745	0.0002	0.0481888
RNA-Seq	Trans-ABySS	XLOC_114314	S1743035:0-372	cdRNA02-Dia-R1	cdRNA05-postDia	37.714	694.851	4.20353	5.00E-05	0.0195319
RNA-Seq	Trans-ABySS	XLOC_114314	S1743035:0-372	cdRNA01-preDia	cdRNA05-postDia	20.05	694.851	5.11503	5.00E-05	0.0195319
RNA-Seq	Trans-ABySS	XLOC_114314	S1743035:0-372	cdRNA03-Dia-R2	cdRNA05-postDia	14.3096	694.851	5.60165	5.00E-05	0.0195319
RNA-Seq	Trans-ABySS	XLOC_114314	S1743035:0-372	cdRNA04-Dia-R3	cdRNA05-postDia	14.2017	694.851	5.61256	5.00E-05	0.0195319
RNA-Seq	Trans-ABySS	XLOC_114870	S177058:0-818	cdRNA01-preDia	cdRNA05-postDia	9.13676	54.5216	2.57707	5.00E-05	0.0195319
RNA-Seq	Trans-ABySS	XLOC_114949	S177832:0-578	cdRNA03-Dia-R2	cdRNA05-postDia	10.615	72.1988	2.76587	5.00E-05	0.0195319
RNA-Seq	Trans-ABySS	XLOC_114949	S177832:0-578	cdRNA04-Dia-R3	cdRNA05-postDia	10.2729	72.1988	2.81314	5.00E-05	0.0195319
RNA-Seq	Trans-ABySS	XLOC_115118	S179416:0-512	cdRNA01-preDia	cdRNA05-postDia	4.88663	67.2275	3.78214	5.00E-05	0.0195319
RNA-Seq	Trans-ABySS	XLOC_115118	S179416:0-512	cdRNA04-Dia-R3	cdRNA05-postDia	4.71244	67.2275	3.83451	5.00E-05	0.0195319
RNA-Seq	Trans-ABySS	XLOC_115118	S179416:0-512	cdRNA02-Dia-R1	cdRNA05-postDia	3.70794	67.2275	4.18036	5.00E-05	0.0195319
RNA-Seq	Trans-ABySS	XLOC_115118	S179416:0-512	cdRNA03-Dia-R2	cdRNA05-postDia	2.89653	67.2275	4.53666	0.00015	0.0374403
RNA-Seq	Trans-ABySS	XLOC_115276	S181074:11-491	cdRNA04-Dia-R3	cdRNA05-postDia	14.539	73.7365	2.34245	5.00E-05	0.0195319
RNA-Seq	Trans-ABySS	XLOC_115276	S181074:11-491	cdRNA03-Dia-R2	cdRNA05-postDia	12.9294	73.7365	2.51173	5.00E-05	0.0195319
RNA-Seq	Trans-ABySS	XLOC_116660	S193976:0-524	cdRNA04-Dia-R3	cdRNA05-postDia	23.4791	121.515	2.37169	0.0002	0.0481888
RNA-Seq	Trans-ABySS	XLOC_121307	S235884:0-307	cdRNA04-Dia-R3	cdRNA05-postDia	28.9503	207.559	2.84187	5.00E-05	0.0195319
RNA-Seq	Trans-ABySS	XLOC_121307	S235884:0-307	cdRNA03-Dia-R2	cdRNA05-postDia	28.1499	207.559	2.88232	5.00E-05	0.0195319
RNA-Seq	Trans-ABySS	XLOC_121783	S240427:0-330	cdRNA01-preDia	cdRNA03-Dia-R2	290.996	20.2788	-3.84296	5.00E-05	0.0195319
RNA-Seq	Trans-ABySS	XLOC_121783	S240427:0-330	cdRNA01-preDia	cdRNA04-Dia-R3	290.996	22.7532	-3.67686	5.00E-05	0.0195319
RNA-Seq	Trans-ABySS	XLOC_121783	S240427:0-330	cdRNA01-preDia	cdRNA02-Dia-R1	290.996	51.3399	-2.50285	0.00015	0.0374403
RNA-Seq	Trans-ABySS	XLOC_121783	S240427:0-330	cdRNA04-Dia-R3	cdRNA05-postDia	22.7532	119.899	2.39768	0.0002	0.0481888
RNA-Seq	Trans-ABySS	XLOC_122075	S242614:0-462	cdRNA02-Dia-R1	cdRNA05-postDia	4.29631	44.51	3.37296	0.0001	0.0354705
RNA-Seq	Trans-ABySS	XLOC_122572	S247007:0-604	cdRNA03-Dia-R2	cdRNA05-postDia	10.7057	57.5297	2.42593	0.00015	0.0374403
RNA-Seq	Trans-ABySS	XLOC_122764	S248512:0-717	cdRNA01-preDia	cdRNA04-Dia-R3	18.7594	3.16221	-2.56861	0.0002	0.0481888
RNA-Seq	Trans-ABySS	XLOC_122764	S248512:0-717	cdRNA03-Dia-R2	cdRNA05-postDia	3.5651	22.7314	2.67267	0.00015	0.0374403
RNA-Seq	Trans-ABySS	XLOC_122764	S248512:0-717	cdRNA04-Dia-R3	cdRNA05-postDia	3.16221	22.7314	2.84568	0.00015	0.0374403
RNA-Seq	Trans-ABySS	XLOC_123302	S253202:0-683	cdRNA04-Dia-R3	cdRNA05-postDia	9.09052	50.9113	2.48555	0.0001	0.0354705

RNA-Seq	Trans-ABySS	XLOC_123302	S253202:0-683	cdRNA03-Dia-R2	cdRNA05-postDia	7.6363	50.9113	2.73704	5.00E-05	0.0195319
RNA-Seq	Trans-ABySS	XLOC_123532	S255233:0-286	cdRNA04-Dia-R3	cdRNA05-postDia	19.5	121.014	2.63363	0.0002	0.0481888
RNA-Seq	Trans-ABySS	XLOC_125982	S277097:0-888	cdRNA03-Dia-R2	cdRNA05-postDia	6.2607	32.8657	2.39219	0.00015	0.0374403
RNA-Seq	Trans-ABySS	XLOC_125982	S277097:0-888	cdRNA04-Dia-R3	cdRNA05-postDia	5.67954	32.8657	2.53274	5.00E-05	0.0195319
RNA-Seq	Trans-ABySS	XLOC_126333	S279979:0-608	cdRNA01-preDia	cdRNA04-Dia-R3	32.0618	157.867	2.29978	0.0002	0.0481888
RNA-Seq	Trans-ABySS	XLOC_126873	S28492:0-642	cdRNA03-Dia-R2	cdRNA05-postDia	5.60134	36.9492	2.7217	5.00E-05	0.0195319
RNA-Seq	Trans-ABySS	XLOC_127574	S291555:0-142	cdRNA01-preDia	cdRNA05-postDia	145.273	1434.87	3.30408	5.00E-05	0.0195319
RNA-Seq	Trans-ABySS	XLOC_128190	S297163:0-341	cdRNA02-Dia-R1	cdRNA05-postDia	17.5129	89.2581	2.34956	0.00015	0.0374403
RNA-Seq	Trans-ABySS	XLOC_128190	S297163:0-341	cdRNA03-Dia-R2	cdRNA05-postDia	12.5975	89.2581	2.82484	5.00E-05	0.0195319
RNA-Seq	Trans-ABySS	XLOC_128190	S297163:0-341	cdRNA01-preDia	cdRNA05-postDia	12.5502	89.2581	2.83027	0.0001	0.0354705
RNA-Seq	Trans-ABySS	XLOC_129417	S307464:0-234	cdRNA02-Dia-R1	cdRNA05-postDia	17.6217	218.651	3.63321	0.0002	0.0481888
RNA-Seq	Trans-ABySS	XLOC_129750	S310569:0-100	cdRNA02-Dia-R1	cdRNA05-postDia	976.599	6372.62	2.70605	5.00E-05	0.0195319
RNA-Seq	Trans-ABySS	XLOC_129750	S310569:0-100	cdRNA01-preDia	cdRNA05-postDia	750.591	6372.62	3.08579	5.00E-05	0.0195319
RNA-Seq	Trans-ABySS	XLOC_130700	S319248:2-186	cdRNA02-Dia-R1	cdRNA05-postDia	80.6347	1100.57	3.77071	5.00E-05	0.0195319
RNA-Seq	Trans-ABySS	XLOC_130700	S319248:2-186	cdRNA04-Dia-R3	cdRNA05-postDia	79.9336	1100.57	3.78331	5.00E-05	0.0195319
RNA-Seq	Trans-ABySS	XLOC_130700	S319248:2-186	cdRNA03-Dia-R2	cdRNA05-postDia	49.2576	1100.57	4.48176	5.00E-05	0.0195319
RNA-Seq	Trans-ABySS	XLOC_130700	S319248:2-186	cdRNA01-preDia	cdRNA05-postDia	39.4155	1100.57	4.80335	5.00E-05	0.0195319
RNA-Seq	Trans-ABySS	XLOC_130800	S320040:0-289	cdRNA02-Dia-R1	cdRNA05-postDia	26.4525	159.834	2.59509	0.00015	0.0374403
RNA-Seq	Trans-ABySS	XLOC_130911	S321116:0-488	cdRNA04-Dia-R3	cdRNA05-postDia	5.86222	35.5956	2.60218	0.00015	0.0374403
RNA-Seq	Trans-ABySS	XLOC_130928	S321298:0-612	cdRNA03-Dia-R2	cdRNA05-postDia	36.0791	3.80359	-3.24573	5.00E-05	0.0195319
RNA-Seq	Trans-ABySS	XLOC_130928	S321298:0-612	cdRNA04-Dia-R3	cdRNA05-postDia	33.5867	3.80359	-3.14246	5.00E-05	0.0195319
RNA-Seq	Trans-ABySS	XLOC_132147	S331819:1-254	cdRNA02-Dia-R1	cdRNA05-postDia	25.3251	286.33	3.49903	5.00E-05	0.0195319
RNA-Seq	Trans-ABySS	XLOC_132147	S331819:1-254	cdRNA03-Dia-R2	cdRNA05-postDia	24.858	286.33	3.52589	5.00E-05	0.0195319
RNA-Seq	Trans-ABySS	XLOC_132147	S331819:1-254	cdRNA04-Dia-R3	cdRNA05-postDia	23.7895	286.33	3.58928	5.00E-05	0.0195319
RNA-Seq	Trans-ABySS	XLOC_132309	S333256:0-286	cdRNA03-Dia-R2	cdRNA05-postDia	11.7161	110.381	3.23593	0.0002	0.0481888
RNA-Seq	Trans-ABySS	XLOC_132694	S336712:0-298	cdRNA04-Dia-R3	cdRNA05-postDia	16.5419	107.155	2.69551	0.0002	0.0481888
RNA-Seq	Trans-ABySS	XLOC_134040	S348206:0-376	cdRNA01-preDia	cdRNA05-postDia	7.94219	65.6286	3.04672	5.00E-05	0.0195319
RNA-Seq	Trans-ABySS	XLOC_136138	S366125:0-371	cdRNA01-preDia	cdRNA05-postDia	9.53894	77.6308	3.02473	5.00E-05	0.0195319
RNA-Seq	Trans-ABySS	XLOC_136366	S368462:0-806	cdRNA02-Dia-R1	cdRNA05-postDia	6.08196	31.8183	2.38725	0.00015	0.0374403
RNA-Seq	Trans-ABySS	XLOC_137584	S379317:0-728	cdRNA01-preDia	cdRNA05-postDia	2.95939	45.7777	3.95128	5.00E-05	0.0195319
RNA-Seq	Trans-ABySS	XLOC_137927	S382340:5-585	cdRNA03-Dia-R2	cdRNA05-postDia	12.4567	62.9706	2.33776	0.00015	0.0374403
RNA-Seq	Trans-ABySS	XLOC_138212	S384983:0-385	cdRNA01-preDia	cdRNA05-postDia	14.3486	109.145	2.92726	5.00E-05	0.0195319
RNA-Seq	Trans-ABySS	XLOC_139318	S394841:0-222	cdRNA02-Dia-R1	cdRNA05-postDia	80.469	603.018	2.90569	5.00E-05	0.0195319
RNA-Seq	Trans-ABySS	XLOC_139318	S394841:0-222	cdRNA01-preDia	cdRNA05-postDia	39.8352	603.018	3.92008	5.00E-05	0.0195319
RNA-Seq	Trans-ABySS	XLOC_139318	S394841:0-222	cdRNA04-Dia-R3	cdRNA05-postDia	25.5671	603.018	4.55984	5.00E-05	0.0195319
RNA-Seq	Trans-ABySS	XLOC_139318	S394841:0-222	cdRNA03-Dia-R2	cdRNA05-postDia	20.0366	603.018	4.91149	5.00E-05	0.0195319
RNA-Seq	Trans-ABySS	XLOC_139817	S399405:0-353	cdRNA03-Dia-R2	cdRNA05-postDia	16.3872	96.2517	2.55424	0.0001	0.0354705
RNA-Seq	Trans-ABySS	XLOC_141001	S409833:0-962	cdRNA04-Dia-R3	cdRNA05-postDia	9.06745	49.6042	2.45169	0.0001	0.0354705
RNA-Seq	Trans-ABySS	XLOC_141001	S409833:0-962	cdRNA01-preDia	cdRNA05-postDia	8.78499	49.6042	2.49735	5.00E-05	0.0195319
RNA-Seq	Trans-ABySS	XLOC_141001	S409833:0-962	cdRNA02-Dia-R1	cdRNA05-postDia	5.40638	49.6042	3.19773	5.00E-05	0.0195319
RNA-Seq	Trans-ABySS	XLOC_141037	S41010:0-392	cdRNA04-Dia-R3	cdRNA05-postDia	11.119	70.9414	2.6736	5.00E-05	0.0195319
RNA-Seq	Trans-ABySS	XLOC_141663	S415582:0-696	cdRNA01-preDia	cdRNA04-Dia-R3	51.0787	3.47072	-3.87942	5.00E-05	0.0195319
RNA-Seq	Trans-ABySS	XLOC_141663	S415582:0-696	cdRNA01-preDia	cdRNA03-Dia-R2	51.0787	3.8986	-3.7117	5.00E-05	0.0195319
RNA-Seq	Trans-ABySS	XLOC_141663	S415582:0-696	cdRNA02-Dia-R1	cdRNA04-Dia-R3	26.0801	3.47072	-2.90965	5.00E-05	0.0195319
RNA-Seq	Trans-ABySS	XLOC_141707	S415901:0-571	cdRNA02-Dia-R1	cdRNA05-postDia	14.6441	67.8975	2.21304	0.0002	0.0481888
RNA-Seq	Trans-ABySS	XLOC_141707	S415901:0-571	cdRNA01-preDia	cdRNA05-postDia	13.546	67.8975	2.3255	0.00015	0.0374403
RNA-Seq	Trans-ABySS	XLOC_142674	S424412:0-482	cdRNA01-preDia	cdRNA03-Dia-R2	9.02324	53.7522	2.57461	0.0002	0.0481888

RNA-Seq	Trans-ABySS	XLOC_142674	S424412:0-482	cdRNA01-preDia	cdRNA04-Dia-R3	9.02324	55.4342	2.61906	0.00015	0.0374403
RNA-Seq	Trans-ABySS	XLOC_142765	S425228:0-264	cdRNA02-Dia-R1	cdRNA05-postDia	45.9577	268.84	2.54837	0.0002	0.0481888
RNA-Seq	Trans-ABySS	XLOC_142765	S425228:0-264	cdRNA01-preDia	cdRNA05-postDia	26.2746	268.84	3.355	5.00E-05	0.0195319
RNA-Seq	Trans-ABySS	XLOC_142884	S426097:10-450	cdRNA01-preDia	cdRNA04-Dia-R3	9.85597	58.0637	2.55857	5.00E-05	0.0195319
RNA-Seq	Trans-ABySS	XLOC_143551	S432113:0-296	cdRNA01-preDia	cdRNA05-postDia	16.578	108.67	2.71261	0.0001	0.0354705
RNA-Seq	Trans-ABySS	XLOC_143686	S433280:0-900	cdRNA03-Dia-R2	cdRNA05-postDia	28.7129	3.56755	-3.00869	5.00E-05	0.0195319
RNA-Seq	Trans-ABySS	XLOC_143686	S433280:0-900	cdRNA04-Dia-R3	cdRNA05-postDia	26.988	3.56755	-2.91932	5.00E-05	0.0195319
RNA-Seq	Trans-ABySS	XLOC_144332	S439031:0-708	cdRNA04-Dia-R3	cdRNA05-postDia	3.13398	24.7209	2.97966	5.00E-05	0.0195319
RNA-Seq	Trans-ABySS	XLOC_144332	S439031:0-708	cdRNA02-Dia-R1	cdRNA05-postDia	2.66588	24.7209	3.21304	5.00E-05	0.0195319
RNA-Seq	Trans-ABySS	XLOC_144332	S439031:0-708	cdRNA03-Dia-R2	cdRNA05-postDia	2.56669	24.7209	3.26775	5.00E-05	0.0195319
RNA-Seq	Trans-ABySS	XLOC_144419	S439746:0-239	cdRNA03-Dia-R2	cdRNA05-postDia	18.3625	272.914	3.89361	0.0001	0.0354705
RNA-Seq	Trans-ABySS	XLOC_144435	S439884:1-298	cdRNA03-Dia-R2	cdRNA05-postDia	16.9839	114.979	2.75913	0.0002	0.0481888
RNA-Seq	Trans-ABySS	XLOC_144435	S439884:1-298	cdRNA04-Dia-R3	cdRNA05-postDia	14.936	114.979	2.94451	0.0001	0.0354705
RNA-Seq	Trans-ABySS	XLOC_144435	S439884:1-298	cdRNA01-preDia	cdRNA05-postDia	13.6108	114.979	3.07855	5.00E-05	0.0195319
RNA-Seq	Trans-ABySS	XLOC_145321	S44790:0-182	cdRNA01-preDia	cdRNA05-postDia	111.633	613.492	2.45829	0.0002	0.0481888
RNA-Seq	Trans-ABySS	XLOC_145321	S44790:0-182	cdRNA03-Dia-R2	cdRNA05-postDia	82.4419	613.492	2.89559	0.0001	0.0354705
RNA-Seq	Trans-ABySS	XLOC_145340	S448039:0-184	cdRNA04-Dia-R3	cdRNA05-postDia	36.8924	412.981	3.48468	0.0002	0.0481888
RNA-Seq	Trans-ABySS	XLOC_146164	S455804:6-253	cdRNA04-Dia-R3	cdRNA05-postDia	29.4028	240.395	3.03138	5.00E-05	0.0195319
RNA-Seq	Trans-ABySS	XLOC_146164	S455804:6-253	cdRNA03-Dia-R2	cdRNA05-postDia	28.3601	240.395	3.08348	0.00015	0.0374403
RNA-Seq	Trans-ABySS	XLOC_147298	S465917:0-598	cdRNA03-Dia-R2	cdRNA05-postDia	8.94402	57.0554	2.67337	5.00E-05	0.0195319
RNA-Seq	Trans-ABySS	XLOC_147298	S465917:0-598	cdRNA04-Dia-R3	cdRNA05-postDia	7.04269	57.0554	3.01816	0.0001	0.0354705
RNA-Seq	Trans-ABySS	XLOC_147325	S466135:0-555	cdRNA01-preDia	cdRNA05-postDia	11.3048	49.2652	2.12364	0.0002	0.0481888
RNA-Seq	Trans-ABySS	XLOC_147325	S466135:0-555	cdRNA02-Dia-R1	cdRNA05-postDia	8.42137	49.2652	2.54844	5.00E-05	0.0195319
RNA-Seq	Trans-ABySS	XLOC_147473	S467547:0-256	cdRNA01-preDia	cdRNA05-postDia	38.2107	220.076	2.52595	0.0001	0.0354705
RNA-Seq	Trans-ABySS	XLOC_147473	S467547:0-256	cdRNA02-Dia-R1	cdRNA05-postDia	29.7341	220.076	2.88781	0.00015	0.0374403
RNA-Seq	Trans-ABySS	XLOC_149087	S482092:0-229	cdRNA04-Dia-R3	cdRNA05-postDia	421.98	64.894	-2.70102	0.00015	0.0374403
RNA-Seq	Trans-ABySS	XLOC_150350	S493190:0-827	cdRNA01-preDia	cdRNA05-postDia	2.63554	17.7215	2.74933	0.0001	0.0354705
RNA-Seq	Trans-ABySS	XLOC_150350	S493190:0-827	cdRNA01-preDia	cdRNA03-Dia-R2	2.63554	24.9888	3.24511	5.00E-05	0.0195319
RNA-Seq	Trans-ABySS	XLOC_150350	S493190:0-827	cdRNA01-preDia	cdRNA04-Dia-R3	2.63554	25.8333	3.29306	5.00E-05	0.0195319
RNA-Seq	Trans-ABySS	XLOC_150350	S493190:0-827	cdRNA01-preDia	cdRNA02-Dia-R1	2.63554	43.8345	4.0559	5.00E-05	0.0195319
RNA-Seq	Trans-ABySS	XLOC_150648	S495731:0-374	cdRNA01-preDia	cdRNA05-postDia	9.10548	67.6911	2.89416	5.00E-05	0.0195319
RNA-Seq	Trans-ABySS	XLOC_151132	S499958:0-118	cdRNA04-Dia-R3	cdRNA05-postDia	447.077	2928.98	2.7118	0.0001	0.0354705
RNA-Seq	Trans-ABySS	XLOC_151132	S499958:0-118	cdRNA02-Dia-R1	cdRNA05-postDia	423.939	2928.98	2.78847	5.00E-05	0.0195319
RNA-Seq	Trans-ABySS	XLOC_151142	S500047:0-667	cdRNA01-preDia	cdRNA02-Dia-R1	86.1945	1.59607	-5.755	5.00E-05	0.0195319
RNA-Seq	Trans-ABySS	XLOC_151142	S500047:0-667	cdRNA01-preDia	cdRNA04-Dia-R3	86.1945	5.43995	-3.98593	5.00E-05	0.0195319
RNA-Seq	Trans-ABySS	XLOC_151142	S500047:0-667	cdRNA01-preDia	cdRNA03-Dia-R2	86.1945	5.78062	-3.8983	5.00E-05	0.0195319
RNA-Seq	Trans-ABySS	XLOC_153197	S517810:0-1096	cdRNA04-Dia-R3	cdRNA05-postDia	9.76326	57.5621	2.55968	0.00015	0.0374403
RNA-Seq	Trans-ABySS	XLOC_153197	S517810:0-1096	cdRNA03-Dia-R2	cdRNA05-postDia	9.40163	57.5621	2.61414	0.0001	0.0354705
RNA-Seq	Trans-ABySS	XLOC_153900	S52414:1-159	cdRNA01-preDia	cdRNA05-postDia	119.404	951.101	2.99374	5.00E-05	0.0195319
RNA-Seq	Trans-ABySS	XLOC_153991	S525034:0-580	cdRNA01-preDia	cdRNA05-postDia	3.03251	31.3674	3.37068	5.00E-05	0.0195319
RNA-Seq	Trans-ABySS	XLOC_154782	S531848:2-475	cdRNA02-Dia-R1	cdRNA05-postDia	12.6116	82.5779	2.711	5.00E-05	0.0195319
RNA-Seq	Trans-ABySS	XLOC_155149	S535092:0-487	cdRNA02-Dia-R1	cdRNA05-postDia	22.4325	115.624	2.36578	0.00015	0.0374403
RNA-Seq	Trans-ABySS	XLOC_155158	S535140:0-851	cdRNA01-preDia	cdRNA05-postDia	2.96075	26.4529	3.15939	5.00E-05	0.0195319
RNA-Seq	Trans-ABySS	XLOC_155556	S538828:13-453	cdRNA04-Dia-R3	cdRNA05-postDia	50.2857	5.4483	-3.20627	5.00E-05	0.0195319
RNA-Seq	Trans-ABySS	XLOC_155556	S538828:13-453	cdRNA03-Dia-R2	cdRNA05-postDia	48.3861	5.4483	-3.15071	5.00E-05	0.0195319
RNA-Seq	Trans-ABySS	XLOC_155556	S538828:13-453	cdRNA01-preDia	cdRNA03-Dia-R2	7.15041	48.3861	2.75849	0.0001	0.0354705
RNA-Seq	Trans-ABySS	XLOC_155556	S538828:13-453	cdRNA01-preDia	cdRNA04-Dia-R3	7.15041	50.2857	2.81405	5.00E-05	0.0195319

RNA-Seq	Trans-ABySS	XLOC_156204	S544452:0-366	cdRNA04-Dia-R3	cdRNA05-postDia	10.5128	66.0599	2.65162	0.0002	0.0481888
RNA-Seq	Trans-ABySS	XLOC_156258	S544847:0-434	cdRNA01-preDia	cdRNA03-Dia-R2	4.95737	70.5933	3.83189	5.00E-05	0.0195319
RNA-Seq	Trans-ABySS	XLOC_156258	S544847:0-434	cdRNA01-preDia	cdRNA04-Dia-R3	4.95737	77.3959	3.96461	5.00E-05	0.0195319
RNA-Seq	Trans-ABySS	XLOC_157473	S555625:0-236	cdRNA04-Dia-R3	cdRNA05-postDia	39.2173	249.826	2.67136	0.00015	0.0374403
RNA-Seq	Trans-ABySS	XLOC_158073	S561208:0-234	cdRNA01-preDia	cdRNA05-postDia	46.897	277.268	2.56371	0.0002	0.0481888
RNA-Seq	Trans-ABySS	XLOC_159568	S574540:0-194	cdRNA02-Dia-R1	cdRNA05-postDia	96.0588	514.237	2.42044	0.0001	0.0354705
RNA-Seq	Trans-ABySS	XLOC_159568	S574540:0-194	cdRNA04-Dia-R3	cdRNA05-postDia	66.1275	514.237	2.95911	5.00E-05	0.0195319
RNA-Seq	Trans-ABySS	XLOC_159568	S574540:0-194	cdRNA03-Dia-R2	cdRNA05-postDia	47.8368	514.237	3.42624	5.00E-05	0.0195319
RNA-Seq	Trans-ABySS	XLOC_160652	S584346:0-166	cdRNA04-Dia-R3	cdRNA05-postDia	89.799	746.147	3.05469	0.0002	0.0481888
RNA-Seq	Trans-ABySS	XLOC_160652	S584346:0-166	cdRNA03-Dia-R2	cdRNA05-postDia	71.938	746.147	3.37463	0.0002	0.0481888
RNA-Seq	Trans-ABySS	XLOC_162104	S597500:2-849	cdRNA01-preDia	cdRNA05-postDia	14.4702	92.0578	2.66945	0.00015	0.0374403
RNA-Seq	Trans-ABySS	XLOC_162104	S597500:2-849	cdRNA02-Dia-R1	cdRNA05-postDia	7.5748	92.0578	3.60326	5.00E-05	0.0195319
RNA-Seq	Trans-ABySS	XLOC_162104	S597500:2-849	cdRNA04-Dia-R3	cdRNA05-postDia	4.31548	92.0578	4.41495	5.00E-05	0.0195319
RNA-Seq	Trans-ABySS	XLOC_162104	S597500:2-849	cdRNA03-Dia-R2	cdRNA05-postDia	4.16244	92.0578	4.46704	5.00E-05	0.0195319
RNA-Seq	Trans-ABySS	XLOC_162166	S598012:0-608	cdRNA01-preDia	cdRNA05-postDia	4.51575	74.7396	4.04884	5.00E-05	0.0195319
RNA-Seq	Trans-ABySS	XLOC_162166	S598012:0-608	cdRNA03-Dia-R2	cdRNA05-postDia	4.41653	74.7396	4.08089	5.00E-05	0.0195319
RNA-Seq	Trans-ABySS	XLOC_162166	S598012:0-608	cdRNA04-Dia-R3	cdRNA05-postDia	2.32468	74.7396	5.00677	5.00E-05	0.0195319
RNA-Seq	Trans-ABySS	XLOC_163333	S608015:0-196	cdRNA04-Dia-R3	cdRNA05-postDia	60.4991	371.924	2.62002	0.00015	0.0374403
RNA-Seq	Trans-ABySS	XLOC_163337	S608076:0-329	cdRNA02-Dia-R1	cdRNA05-postDia	49.0719	249.647	2.34692	0.00015	0.0374403
RNA-Seq	Trans-ABySS	XLOC_163337	S608076:0-329	cdRNA03-Dia-R2	cdRNA05-postDia	47.2018	249.647	2.40297	0.00015	0.0374403
RNA-Seq	Trans-ABySS	XLOC_163337	S608076:0-329	cdRNA04-Dia-R3	cdRNA05-postDia	45.1729	249.647	2.46636	5.00E-05	0.0195319
RNA-Seq	Trans-ABySS	XLOC_165678	S629087:1-281	cdRNA03-Dia-R2	cdRNA05-postDia	48.6805	238.714	2.29387	5.00E-05	0.0195319
RNA-Seq	Trans-ABySS	XLOC_168140	S651254:0-765	cdRNA02-Dia-R1	cdRNA05-postDia	7.90358	42.2053	2.41685	0.00015	0.0374403
RNA-Seq	Trans-ABySS	XLOC_168869	S658009:3-325	cdRNA01-preDia	cdRNA05-postDia	19.3782	120.05	2.63112	5.00E-05	0.0195319
RNA-Seq	Trans-ABySS	XLOC_168869	S658009:3-325	cdRNA03-Dia-R2	cdRNA05-postDia	11.8917	120.05	3.3356	0.0001	0.0354705
RNA-Seq	Trans-ABySS	XLOC_168869	S658009:3-325	cdRNA04-Dia-R3	cdRNA05-postDia	10.6693	120.05	3.4921	5.00E-05	0.0195319
RNA-Seq	Trans-ABySS	XLOC_168869	S658009:3-325	cdRNA02-Dia-R1	cdRNA05-postDia	8.67487	120.05	3.79064	5.00E-05	0.0195319
RNA-Seq	Trans-ABySS	XLOC_169115	S659983:0-231	cdRNA01-preDia	cdRNA05-postDia	64.8787	353.203	2.44468	0.0002	0.0481888
RNA-Seq	Trans-ABySS	XLOC_169115	S659983:0-231	cdRNA03-Dia-R2	cdRNA05-postDia	52.715	353.203	2.74421	5.00E-05	0.0195319
RNA-Seq	Trans-ABySS	XLOC_169115	S659983:0-231	cdRNA04-Dia-R3	cdRNA05-postDia	39.2382	353.203	3.17017	5.00E-05	0.0195319
RNA-Seq	Trans-ABySS	XLOC_169115	S659983:0-231	cdRNA02-Dia-R1	cdRNA05-postDia	38.2252	353.203	3.2079	5.00E-05	0.0195319
RNA-Seq	Trans-ABySS	XLOC_170030	S668139:0-62	cdRNA01-preDia	cdRNA05-postDia	31772.6	253750	2.99755	5.00E-05	0.0195319
RNA-Seq	Trans-ABySS	XLOC_171149	S678393:0-63	cdRNA02-Dia-R1	cdRNA05-postDia	128601	5133.68	-4.64676	5.00E-05	0.0195319
RNA-Seq	Trans-ABySS	XLOC_171194	S678809:0-413	cdRNA03-Dia-R2	cdRNA05-postDia	61.6713	5.93917	-3.37627	5.00E-05	0.0195319
RNA-Seq	Trans-ABySS	XLOC_171194	S678809:0-413	cdRNA04-Dia-R3	cdRNA05-postDia	60.2458	5.93917	-3.34253	5.00E-05	0.0195319
RNA-Seq	Trans-ABySS	XLOC_171194	S678809:0-413	cdRNA02-Dia-R1	cdRNA04-Dia-R3	8.51663	60.2458	2.82251	5.00E-05	0.0195319
RNA-Seq	Trans-ABySS	XLOC_171194	S678809:0-413	cdRNA02-Dia-R1	cdRNA03-Dia-R2	8.51663	61.6713	2.85624	5.00E-05	0.0195319
RNA-Seq	Trans-ABySS	XLOC_172354	S68924:0-364	cdRNA04-Dia-R3	cdRNA05-postDia	10.9075	73.2235	2.74699	5.00E-05	0.0195319
RNA-Seq	Trans-ABySS	XLOC_172883	S694139:0-1406	cdRNA04-Dia-R3	cdRNA05-postDia	4.47504	23.461	2.39029	0.00015	0.0374403
RNA-Seq	Trans-ABySS	XLOC_172883	S694139:0-1406	cdRNA03-Dia-R2	cdRNA05-postDia	3.85958	23.461	2.60375	5.00E-05	0.0195319
RNA-Seq	Trans-ABySS	XLOC_173102	S696157:0-244	cdRNA03-Dia-R2	cdRNA05-postDia	42.5709	257.96	2.5992	0.0002	0.0481888
RNA-Seq	Trans-ABySS	XLOC_173464	S699341:0-541	cdRNA04-Dia-R3	cdRNA05-postDia	5.70035	36.313	2.67136	0.00015	0.0374403
RNA-Seq	Trans-ABySS	XLOC_174284	S706664:0-1006	cdRNA02-Dia-R1	cdRNA05-postDia	10.052	70.7112	2.81445	5.00E-05	0.0195319
RNA-Seq	Trans-ABySS	XLOC_174284	S706664:0-1006	cdRNA01-preDia	cdRNA05-postDia	4.89082	70.7112	3.85379	5.00E-05	0.0195319
RNA-Seq	Trans-ABySS	XLOC_174284	S706664:0-1006	cdRNA04-Dia-R3	cdRNA05-postDia	1.9695	70.7112	5.16604	5.00E-05	0.0195319
RNA-Seq	Trans-ABySS	XLOC_174284	S706664:0-1006	cdRNA03-Dia-R2	cdRNA05-postDia	1.27927	70.7112	5.78854	5.00E-05	0.0195319
RNA-Seq	Trans-ABySS	XLOC_174983	S71278:8-267	cdRNA02-Dia-R1	cdRNA05-postDia	33.8217	219.166	2.696	0.0001	0.0354705

RNA-Seq	Trans-ABySS	XLOC_176054	S722140:0-1335	cdRNA03-Dia-R2	cdRNA05-postDia	30.764	5.84141	-2.39686	0.00015	0.0374403
RNA-Seq	Trans-ABySS	XLOC_176508	S726356:0-721	cdRNA04-Dia-R3	cdRNA05-postDia	8.83524	56.5175	2.67736	5.00E-05	0.0195319
RNA-Seq	Trans-ABySS	XLOC_176508	S726356:0-721	cdRNA03-Dia-R2	cdRNA05-postDia	8.28297	56.5175	2.77048	5.00E-05	0.0195319
RNA-Seq	Trans-ABySS	XLOC_177026	S730778:0-1369	cdRNA01-preDia	cdRNA02-Dia-R1	6.96874	53.6585	2.94484	5.00E-05	0.0195319
RNA-Seq	Trans-ABySS	XLOC_177303	S733248:0-759	cdRNA01-preDia	cdRNA04-Dia-R3	7.97138	39.6132	2.31308	0.0001	0.0354705
RNA-Seq	Trans-ABySS	XLOC_177540	S735220:0-647	cdRNA01-preDia	cdRNA05-postDia	11.8399	81.3717	2.78088	5.00E-05	0.0195319
RNA-Seq	Trans-ABySS	XLOC_177540	S735220:0-647	cdRNA03-Dia-R2	cdRNA05-postDia	10.17	81.3717	3.00021	5.00E-05	0.0195319
RNA-Seq	Trans-ABySS	XLOC_177540	S735220:0-647	cdRNA04-Dia-R3	cdRNA05-postDia	8.76922	81.3717	3.21401	5.00E-05	0.0195319
RNA-Seq	Trans-ABySS	XLOC_177540	S735220:0-647	cdRNA02-Dia-R1	cdRNA05-postDia	5.83845	81.3717	3.80087	5.00E-05	0.0195319
RNA-Seq	Trans-ABySS	XLOC_178249	S741185:0-1361	cdRNA01-preDia	cdRNA03-Dia-R2	21.3661	3.54704	-2.59064	0.0001	0.0354705
RNA-Seq	Trans-ABySS	XLOC_178249	S741185:0-1361	cdRNA01-preDia	cdRNA04-Dia-R3	21.3661	3.87424	-2.46334	0.0001	0.0354705
RNA-Seq	Trans-ABySS	XLOC_178249	S741185:0-1361	cdRNA03-Dia-R2	cdRNA05-postDia	3.54704	17.9735	2.34119	0.00015	0.0374403
RNA-Seq	Trans-ABySS	XLOC_178537	S743626:0-389	cdRNA01-preDia	cdRNA05-postDia	3.44872	52.6849	3.93326	0.0002	0.0481888
RNA-Seq	Trans-ABySS	XLOC_178770	S745704:0-646	cdRNA03-Dia-R2	cdRNA05-postDia	33.7076	5.81825	-2.53442	0.00015	0.0374403
RNA-Seq	Trans-ABySS	XLOC_178770	S745704:0-646	cdRNA04-Dia-R3	cdRNA05-postDia	33.3211	5.81825	-2.51778	0.00015	0.0374403
RNA-Seq	Trans-ABySS	XLOC_179703	S754033:0-179	cdRNA04-Dia-R3	cdRNA05-postDia	131.394	795.293	2.59759	0.0001	0.0354705
RNA-Seq	Trans-ABySS	XLOC_179703	S754033:0-179	cdRNA02-Dia-R1	cdRNA05-postDia	98.0385	795.293	3.02006	0.00015	0.0374403
RNA-Seq	Trans-ABySS	XLOC_179703	S754033:0-179	cdRNA03-Dia-R2	cdRNA05-postDia	61.5461	795.293	3.69175	5.00E-05	0.0195319
RNA-Seq	Trans-ABySS	XLOC_179823	S755006:0-265	cdRNA01-preDia	cdRNA05-postDia	42.8663	325.172	2.92329	5.00E-05	0.0195319
RNA-Seq	Trans-ABySS	XLOC_179823	S755006:0-265	cdRNA04-Dia-R3	cdRNA05-postDia	24.9245	325.172	3.70557	5.00E-05	0.0195319
RNA-Seq	Trans-ABySS	XLOC_179823	S755006:0-265	cdRNA03-Dia-R2	cdRNA05-postDia	22.2327	325.172	3.87045	5.00E-05	0.0195319
RNA-Seq	Trans-ABySS	XLOC_179823	S755006:0-265	cdRNA02-Dia-R1	cdRNA05-postDia	19.6117	325.172	4.05142	5.00E-05	0.0195319
RNA-Seq	Trans-ABySS	XLOC_181916	S774052:2-363	cdRNA01-preDia	cdRNA05-postDia	14.4768	88.3826	2.61002	0.00015	0.0374403
RNA-Seq	Trans-ABySS	XLOC_182768	S781332:0-167	cdRNA03-Dia-R2	cdRNA05-postDia	45.8753	571.516	3.639	0.0002	0.0481888
RNA-Seq	Trans-ABySS	XLOC_183246	S785948:0-855	cdRNA03-Dia-R2	cdRNA05-postDia	10.9634	62.7972	2.518	5.00E-05	0.0195319
RNA-Seq	Trans-ABySS	XLOC_183246	S785948:0-855	cdRNA04-Dia-R3	cdRNA05-postDia	10.2299	62.7972	2.61791	5.00E-05	0.0195319
RNA-Seq	Trans-ABySS	XLOC_183250	S785975:0-615	cdRNA01-preDia	cdRNA05-postDia	60.9231	8.19893	-2.89348	5.00E-05	0.0195319
RNA-Seq	Trans-ABySS	XLOC_184619	S798635:0-524	cdRNA02-Dia-R1	cdRNA05-postDia	4.40864	39.2119	3.15289	0.0001	0.0354705
RNA-Seq	Trans-ABySS	XLOC_184619	S798635:0-524	cdRNA04-Dia-R3	cdRNA05-postDia	4.13552	39.2119	3.24515	5.00E-05	0.0195319
RNA-Seq	Trans-ABySS	XLOC_184619	S798635:0-524	cdRNA03-Dia-R2	cdRNA05-postDia	3.34549	39.2119	3.551	5.00E-05	0.0195319
RNA-Seq	Trans-ABySS	XLOC_184933	S801301:0-572	cdRNA01-preDia	cdRNA02-Dia-R1	40.0317	2.00809	-4.31725	5.00E-05	0.0195319
RNA-Seq	Trans-ABySS	XLOC_185208	S803745:0-101	cdRNA02-Dia-R1	cdRNA05-postDia	440.536	5396.42	3.61467	0.0002	0.0481888
RNA-Seq	Trans-ABySS	XLOC_185486	S806171:0-507	cdRNA02-Dia-R1	cdRNA05-postDia	6.87311	52.972	2.94619	0.0002	0.0481888
RNA-Seq	Trans-ABySS	XLOC_186145	S811973:0-336	cdRNA03-Dia-R2	cdRNA05-postDia	39.4873	206.894	2.38943	0.00015	0.0374403
RNA-Seq	Trans-ABySS	XLOC_186145	S811973:0-336	cdRNA02-Dia-R1	cdRNA05-postDia	28.7268	206.894	2.84843	5.00E-05	0.0195319
RNA-Seq	Trans-ABySS	XLOC_186901	S818457:1-169	cdRNA01-preDia	cdRNA05-postDia	1229.41	110.003	-3.48234	5.00E-05	0.0195319
RNA-Seq	Trans-ABySS	XLOC_187376	S822859:5-742	cdRNA02-Dia-R1	cdRNA05-postDia	11.2177	64.6372	2.52658	0.0001	0.0354705
RNA-Seq	Trans-ABySS	XLOC_187747	S826258:0-635	cdRNA02-Dia-R1	cdRNA05-postDia	5.84513	28.1877	2.26976	0.0002	0.0481888
RNA-Seq	Trans-ABySS	XLOC_188418	S832312:1-306	cdRNA02-Dia-R1	cdRNA05-postDia	23.8195	144.038	2.59623	5.00E-05	0.0195319
RNA-Seq	Trans-ABySS	XLOC_188449	S832620:1-567	cdRNA01-preDia	cdRNA03-Dia-R2	26.4582	2.21802	-3.57637	0.00015	0.0374403
RNA-Seq	Trans-ABySS	XLOC_188449	S832620:1-567	cdRNA01-preDia	cdRNA04-Dia-R3	26.4582	2.35853	-3.48775	0.0001	0.0354705
RNA-Seq	Trans-ABySS	XLOC_188912	S836722:0-304	cdRNA01-preDia	cdRNA05-postDia	15.431	90.0075	2.54422	5.00E-05	0.0195319
RNA-Seq	Trans-ABySS	XLOC_189122	S838425:0-446	cdRNA01-preDia	cdRNA03-Dia-R2	34.8657	2.76483	-3.65654	0.00015	0.0374403
RNA-Seq	Trans-ABySS	XLOC_191991	S865189:0-178	cdRNA02-Dia-R1	cdRNA05-postDia	58.2204	489.927	3.07297	0.00015	0.0374403
RNA-Seq	Trans-ABySS	XLOC_191991	S865189:0-178	cdRNA01-preDia	cdRNA05-postDia	46.9376	489.927	3.38375	0.0002	0.0481888
RNA-Seq	Trans-ABySS	XLOC_192880	S873696:0-773	cdRNA03-Dia-R2	cdRNA05-postDia	2.74386	22.1307	3.01177	0.0001	0.0354705
RNA-Seq	Trans-ABySS	XLOC_193676	S880752:0-609	cdRNA02-Dia-R1	cdRNA05-postDia	7.63031	49.4844	2.69716	5.00E-05	0.0195319

RNA-Seq	Trans-ABySS	XLOC_194188	S885128:0-361	cdRNA01-preDia	cdRNA05-postDia	10.4233	64.7388	2.63482	0.0002	0.0481888
RNA-Seq	Trans-ABySS	XLOC_194255	S88570:0-282	cdRNA03-Dia-R2	cdRNA05-postDia	39.7534	278.71	2.80961	0.0001	0.0354705
RNA-Seq	Trans-ABySS	XLOC_194255	S88570:0-282	cdRNA04-Dia-R3	cdRNA05-postDia	32.9721	278.71	3.07945	5.00E-05	0.0195319
RNA-Seq	Trans-ABySS	XLOC_194255	S88570:0-282	cdRNA02-Dia-R1	cdRNA05-postDia	29.1369	278.71	3.25784	5.00E-05	0.0195319
RNA-Seq	Trans-ABySS	XLOC_194255	S88570:0-282	cdRNA01-preDia	cdRNA05-postDia	8.1293	278.71	5.09949	5.00E-05	0.0195319
RNA-Seq	Trans-ABySS	XLOC_195749	S899445:0-1161	cdRNA02-Dia-R1	cdRNA05-postDia	4.87888	35.3977	2.85903	5.00E-05	0.0195319
RNA-Seq	Trans-ABySS	XLOC_195749	S899445:0-1161	cdRNA04-Dia-R3	cdRNA05-postDia	3.47947	35.3977	3.34672	5.00E-05	0.0195319
RNA-Seq	Trans-ABySS	XLOC_195749	S899445:0-1161	cdRNA03-Dia-R2	cdRNA05-postDia	3.30946	35.3977	3.41899	5.00E-05	0.0195319
RNA-Seq	Trans-ABySS	XLOC_196467	S905950:0-174	cdRNA04-Dia-R3	cdRNA05-postDia	62.8372	660.482	3.39383	0.0002	0.0481888
RNA-Seq	Trans-ABySS	XLOC_196467	S905950:0-174	cdRNA03-Dia-R2	cdRNA05-postDia	49.9012	660.482	3.72637	0.00015	0.0374403
RNA-Seq	Trans-ABySS	XLOC_197203	S913024:0-365	cdRNA02-Dia-R1	cdRNA05-postDia	14.3559	82.6652	2.52564	0.0002	0.0481888
RNA-Seq	Trans-ABySS	XLOC_197203	S913024:0-365	cdRNA03-Dia-R2	cdRNA05-postDia	11.3281	82.6652	2.86737	5.00E-05	0.0195319
RNA-Seq	Trans-ABySS	XLOC_197203	S913024:0-365	cdRNA04-Dia-R3	cdRNA05-postDia	10.5768	82.6652	2.96638	0.0001	0.0354705
RNA-Seq	Trans-ABySS	XLOC_197203	S913024:0-365	cdRNA01-preDia	cdRNA05-postDia	3.95503	82.6652	4.38552	5.00E-05	0.0195319
RNA-Seq	Trans-ABySS	XLOC_197645	S917455:0-2027	cdRNA01-preDia	cdRNA05-postDia	5.02888	30.6951	2.6097	5.00E-05	0.0195319
RNA-Seq	Trans-ABySS	XLOC_199210	S930955:1-1224	cdRNA01-preDia	cdRNA05-postDia	6.98248	38.8571	2.47637	5.00E-05	0.0195319
RNA-Seq	Trans-ABySS	XLOC_199252	S931263:0-640	cdRNA02-Dia-R1	cdRNA05-postDia	14.6378	96.5086	2.72095	5.00E-05	0.0195319
RNA-Seq	Trans-ABySS	XLOC_199252	S931263:0-640	cdRNA03-Dia-R2	cdRNA05-postDia	12.6864	96.5086	2.92737	5.00E-05	0.0195319
RNA-Seq	Trans-ABySS	XLOC_199252	S931263:0-640	cdRNA04-Dia-R3	cdRNA05-postDia	9.98704	96.5086	3.27253	5.00E-05	0.0195319
RNA-Seq	Trans-ABySS	XLOC_199485	S933401:0-648	cdRNA04-Dia-R3	cdRNA05-postDia	7.69184	38.0477	2.30641	0.0002	0.0481888
RNA-Seq	Trans-ABySS	XLOC_199485	S933401:0-648	cdRNA03-Dia-R2	cdRNA05-postDia	7.03265	38.0477	2.43567	0.0001	0.0354705
RNA-Seq	Trans-ABySS	XLOC_200157	S939667:0-155	cdRNA02-Dia-R1	cdRNA05-postDia	231.897	1607.05	2.79286	5.00E-05	0.0195319
RNA-Seq	Trans-ABySS	XLOC_200824	S946026:0-1011	cdRNA01-preDia	cdRNA03-Dia-R2	3.5041	24.5425	2.80817	0.00015	0.0374403
RNA-Seq	Trans-ABySS	XLOC_200824	S946026:0-1011	cdRNA01-preDia	cdRNA04-Dia-R3	3.5041	24.9159	2.82995	0.00015	0.0374403
RNA-Seq	Trans-ABySS	XLOC_200995	S947604:0-980	cdRNA01-preDia	cdRNA05-postDia	7.34693	36.7402	2.32214	0.0001	0.0354705
RNA-Seq	Trans-ABySS	XLOC_201408	S951781:0-443	cdRNA01-preDia	cdRNA05-postDia	9.73226	67.8983	2.80253	5.00E-05	0.0195319
RNA-Seq	Trans-ABySS	XLOC_202044	S957540:0-413	cdRNA02-Dia-R1	cdRNA05-postDia	18.6402	100.542	2.43131	0.0002	0.0481888
RNA-Seq	Trans-ABySS	XLOC_203230	S968288:0-1353	cdRNA02-Dia-R1	cdRNA05-postDia	9.58897	51.9806	2.43853	0.0002	0.0481888
RNA-Seq	Trans-ABySS	XLOC_203358	S969408:0-236	cdRNA02-Dia-R1	cdRNA05-postDia	65.8299	397.368	2.59366	5.00E-05	0.0195319
RNA-Seq	Trans-ABySS	XLOC_203358	S969408:0-236	cdRNA01-preDia	cdRNA05-postDia	35.3816	397.368	3.4894	5.00E-05	0.0195319
RNA-Seq	Trans-ABySS	XLOC_203358	S969408:0-236	cdRNA03-Dia-R2	cdRNA05-postDia	30.051	397.368	3.72499	5.00E-05	0.0195319
RNA-Seq	Trans-ABySS	XLOC_203358	S969408:0-236	cdRNA04-Dia-R3	cdRNA05-postDia	28.7593	397.368	3.78837	5.00E-05	0.0195319
RNA-Seq	Trans-ABySS	XLOC_204233	S977408:0-227	cdRNA03-Dia-R2	cdRNA05-postDia	68.2323	416.183	2.60869	0.0001	0.0354705
RNA-Seq	Trans-ABySS	XLOC_204233	S977408:0-227	cdRNA04-Dia-R3	cdRNA05-postDia	58.3738	416.183	2.83382	5.00E-05	0.0195319
RNA-Seq	Trans-ABySS	XLOC_204233	S977408:0-227	cdRNA01-preDia	cdRNA05-postDia	30.6544	416.183	3.76305	5.00E-05	0.0195319
RNA-Seq	Trans-ABySS	XLOC_204980	S984308:0-1360	cdRNA03-Dia-R2	cdRNA05-postDia	9.83997	53.6221	2.4461	0.00015	0.0374403
RNA-Seq	Trans-ABySS	XLOC_204980	S984308:0-1360	cdRNA04-Dia-R3	cdRNA05-postDia	9.2693	53.6221	2.53229	5.00E-05	0.0195319
RNA-Seq	Trans-ABySS	XLOC_204980	S984308:0-1360	cdRNA02-Dia-R1	cdRNA05-postDia	9.06598	53.6221	2.56429	5.00E-05	0.0195319
RNA-Seq	Trans-ABySS	XLOC_205146	S985626:0-733	cdRNA02-Dia-R1	cdRNA05-postDia	9.39763	50.5417	2.42711	5.00E-05	0.0195319
RNA-Seq	Trans-ABySS	XLOC_205146	S985626:0-733	cdRNA04-Dia-R3	cdRNA05-postDia	9.11901	50.5417	2.47052	0.00015	0.0374403
RNA-Seq	Trans-ABySS	XLOC_205146	S985626:0-733	cdRNA03-Dia-R2	cdRNA05-postDia	8.60102	50.5417	2.55489	5.00E-05	0.0195319
RNA-Seq	Trans-ABySS	XLOC_205146	S985626:0-733	cdRNA01-preDia	cdRNA05-postDia	3.19007	50.5417	3.98581	5.00E-05	0.0195319
RNA-Seq	Trans-ABySS	XLOC_205428	S988095:0-1138	cdRNA01-preDia	cdRNA03-Dia-R2	29.9249	5.42247	-2.46432	5.00E-05	0.0195319
RNA-Seq	Trans-ABySS	XLOC_205428	S988095:0-1138	cdRNA01-preDia	cdRNA04-Dia-R3	29.9249	5.99003	-2.32071	0.0001	0.0354705
RNA-Seq	Trans-ABySS	XLOC_205428	S988095:0-1138	cdRNA04-Dia-R3	cdRNA05-postDia	5.99003	61.9178	3.36972	5.00E-05	0.0195319
RNA-Seq	Trans-ABySS	XLOC_205428	S988095:0-1138	cdRNA03-Dia-R2	cdRNA05-postDia	5.42247	61.9178	3.51333	5.00E-05	0.0195319
RNA-Seq	Trans-ABySS	XLOC_206524	S998204:0-684	cdRNA02-Dia-R1	cdRNA05-postDia	8.04763	51.2671	2.6714	5.00E-05	0.0195319

RNA-Seq	Trans-ABySS	XLOC_206524	S998204:0-684	cdRNA04-Dia-R3	cdRNA05-postDia	7.9154	51.2671	2.6953	5.00E-05	0.0195319
RNA-Seq	Trans-ABySS	XLOC_206524	S998204:0-684	cdRNA03-Dia-R2	cdRNA05-postDia	6.87694	51.2671	2.8982	5.00E-05	0.0195319
RNA-Seq	Trans-ABySS	XLOC_206524	S998204:0-684	cdRNA01-preDia	cdRNA05-postDia	2.9456	51.2671	4.1214	5.00E-05	0.0195319
RNA-Seq	Trans-ABySS	XLOC_206598	S998799:0-570	cdRNA02-Dia-R1	cdRNA05-postDia	12.1144	66.9927	2.46729	0.00015	0.0374403
RNA-Seq	Trans-ABySS	XLOC_206598	S998799:0-570	cdRNA04-Dia-R3	cdRNA05-postDia	8.6312	66.9927	2.95637	5.00E-05	0.0195319
RNA-Seq	Trans-ABySS	XLOC_206598	S998799:0-570	cdRNA03-Dia-R2	cdRNA05-postDia	8.04385	66.9927	3.05805	5.00E-05	0.0195319
RNA-Seq	Trinity	XLOC_000251	c100242_g1_i1:1-605	cdRNA03-Dia-R2	cdRNA05-postDia	11.7358	76.122	2.69739	5.00E-05	0.0249845
RNA-Seq	Trinity	XLOC_000251	c100242_g1_i1:1-605	cdRNA02-Dia-R1	cdRNA05-postDia	10.8618	76.122	2.80905	5.00E-05	0.0249845
RNA-Seq	Trinity	XLOC_000251	c100242_g1_i1:1-605	cdRNA04-Dia-R3	cdRNA05-postDia	10.6127	76.122	2.84252	5.00E-05	0.0249845
RNA-Seq	Trinity	XLOC_000251	c100242_g1_i1:1-605	cdRNA01-preDia	cdRNA05-postDia	8.59409	76.122	3.1469	5.00E-05	0.0249845
RNA-Seq	Trinity	XLOC_000365	c100348_g1_i1:0-843	cdRNA03-Dia-R2	cdRNA05-postDia	2.83981	18.5241	2.70554	5.00E-05	0.0249845
RNA-Seq	Trinity	XLOC_000456	c100434_g1_i1:1-651	cdRNA03-Dia-R2	cdRNA05-postDia	11.3215	69.9689	2.62765	5.00E-05	0.0249845
RNA-Seq	Trinity	XLOC_000745	c10070_g1_i1:0-630	cdRNA02-Dia-R1	cdRNA05-postDia	1.18754	38.5448	5.02049	5.00E-05	0.0249845
RNA-Seq	Trinity	XLOC_000986	c100942_g1_i1:0-203	cdRNA03-Dia-R2	cdRNA05-postDia	400.779	48.5888	-3.04411	5.00E-05	0.0249845
RNA-Seq	Trinity	XLOC_000986	c100942_g1_i1:0-203	cdRNA04-Dia-R3	cdRNA05-postDia	387.644	48.5888	-2.99604	5.00E-05	0.0249845
RNA-Seq	Trinity	XLOC_001258	c10119_g1_i1:2-183	cdRNA02-Dia-R1	cdRNA05-postDia	971.644	6180.04	2.66912	5.00E-05	0.0249845
RNA-Seq	Trinity	XLOC_001258	c10119_g1_i1:2-183	cdRNA04-Dia-R3	cdRNA05-postDia	741.492	6180.04	3.05911	5.00E-05	0.0249845
RNA-Seq	Trinity	XLOC_001258	c10119_g1_i1:2-183	cdRNA03-Dia-R2	cdRNA05-postDia	630.566	6180.04	3.2929	5.00E-05	0.0249845
RNA-Seq	Trinity	XLOC_001258	c10119_g1_i1:2-183	cdRNA01-preDia	cdRNA05-postDia	351.759	6180.04	4.13496	5.00E-05	0.0249845
RNA-Seq	Trinity	XLOC_001277	c101217_g1_i1:0-796	cdRNA03-Dia-R2	cdRNA05-postDia	1.73254	13.3298	2.9437	5.00E-05	0.0249845
RNA-Seq	Trinity	XLOC_001753	c10169_g1_i1:0-819	cdRNA01-preDia	cdRNA04-Dia-R3	28.2056	0.986095	-4.83811	5.00E-05	0.0249845
RNA-Seq	Trinity	XLOC_001753	c10169_g1_i1:0-819	cdRNA01-preDia	cdRNA03-Dia-R2	28.2056	1.47531	-4.25689	5.00E-05	0.0249845
RNA-Seq	Trinity	XLOC_001773	c10171_g1_i1:0-6021	cdRNA02-Dia-R1	cdRNA03-Dia-R2	4961.54	4595.74	-0.11049	5.00E-05	0.0249845
RNA-Seq	Trinity	XLOC_001773	c10171_g1_i1:0-6021	cdRNA01-preDia	cdRNA02-Dia-R1	4587.47	4961.54	0.11309	5.00E-05	0.0249845
RNA-Seq	Trinity	XLOC_001836	c101782_g1_i1:0-584	cdRNA03-Dia-R2	cdRNA05-postDia	2.59527	23.7029	3.19111	5.00E-05	0.0249845
RNA-Seq	Trinity	XLOC_002114	c10204_g2_i1:0-848	cdRNA01-preDia	cdRNA04-Dia-R3	4.90851	24.3074	2.30804	0.0001	0.0432976
RNA-Seq	Trinity	XLOC_002114	c10204_g2_i1:0-848	cdRNA01-preDia	cdRNA03-Dia-R2	4.90851	25.4211	2.37267	0.0001	0.0432976
RNA-Seq	Trinity	XLOC_002389	c102329_g1_i1:0-415	cdRNA03-Dia-R2	cdRNA05-postDia	4.48637	36.8272	3.03715	5.00E-05	0.0249845
RNA-Seq	Trinity	XLOC_002390	c10232_g1_i1:4-389	cdRNA01-preDia	cdRNA05-postDia	7.19678	45.0936	2.6475	0.0001	0.0432976
RNA-Seq	Trinity	XLOC_002472	c10240_g1_i2:29-1014	cdRNA04-Dia-R3	cdRNA05-postDia	148.652	18.9324	-2.97301	5.00E-05	0.0249845
RNA-Seq	Trinity	XLOC_002472	c10240_g1_i2:29-1014	cdRNA03-Dia-R2	cdRNA05-postDia	136.53	18.9324	-2.85029	5.00E-05	0.0249845
RNA-Seq	Trinity	XLOC_002472	c10240_g1_i2:29-1014	cdRNA01-preDia	cdRNA05-postDia	125.826	18.9324	-2.7325	5.00E-05	0.0249845
RNA-Seq	Trinity	XLOC_002473	c10240_g1_i3:19-975	cdRNA02-Dia-R1	cdRNA05-postDia	15.473	193.975	3.64805	5.00E-05	0.0249845
RNA-Seq	Trinity	XLOC_002473	c10240_g1_i3:19-975	cdRNA01-preDia	cdRNA05-postDia	7.46135	193.975	4.70029	5.00E-05	0.0249845
RNA-Seq	Trinity	XLOC_002473	c10240_g1_i3:19-975	cdRNA04-Dia-R3	cdRNA05-postDia	7.35886	193.975	4.72025	5.00E-05	0.0249845
RNA-Seq	Trinity	XLOC_002473	c10240_g1_i3:19-975	cdRNA03-Dia-R2	cdRNA05-postDia	7.08189	193.975	4.7756	5.00E-05	0.0249845
RNA-Seq	Trinity	XLOC_002706	c10263_g1_i1:0-1854	cdRNA01-preDia	cdRNA05-postDia	10.0342	64.2035	2.67773	5.00E-05	0.0249845
RNA-Seq	Trinity	XLOC_002899	c10282_g1_i1:0-1786	cdRNA01-preDia	cdRNA05-postDia	5.42661	35.7337	2.71916	5.00E-05	0.0249845
RNA-Seq	Trinity	XLOC_002900	c10282_g1_i2:0-824	cdRNA01-preDia	cdRNA05-postDia	4.33374	29.8912	2.78604	5.00E-05	0.0249845
RNA-Seq	Trinity	XLOC_003211	c10313_g1_i1:0-635	cdRNA01-preDia	cdRNA02-Dia-R1	3.48376	24.58	2.81877	5.00E-05	0.0249845
RNA-Seq	Trinity	XLOC_003211	c10313_g1_i1:0-635	cdRNA03-Dia-R2	cdRNA05-postDia	13.0884	98.7579	2.91561	5.00E-05	0.0249845
RNA-Seq	Trinity	XLOC_003211	c10313_g1_i1:0-635	cdRNA04-Dia-R3	cdRNA05-postDia	10.7288	98.7579	3.2024	5.00E-05	0.0249845
RNA-Seq	Trinity	XLOC_003211	c10313_g1_i1:0-635	cdRNA01-preDia	cdRNA05-postDia	3.48376	98.7579	4.82518	5.00E-05	0.0249845
RNA-Seq	Trinity	XLOC_003213	c10313_g1_i4:0-636	cdRNA02-Dia-R1	cdRNA05-postDia	11.0216	78.7865	2.83761	5.00E-05	0.0249845
RNA-Seq	Trinity	XLOC_003213	c10313_g1_i4:0-636	cdRNA04-Dia-R3	cdRNA05-postDia	7.28223	78.7865	3.4355	5.00E-05	0.0249845
RNA-Seq	Trinity	XLOC_003213	c10313_g1_i4:0-636	cdRNA03-Dia-R2	cdRNA05-postDia	6.93997	78.7865	3.50495	5.00E-05	0.0249845
RNA-Seq	Trinity	XLOC_003213	c10313_g1_i4:0-636	cdRNA01-preDia	cdRNA05-postDia	3.01836	78.7865	4.70611	5.00E-05	0.0249845

RNA-Seq	Trinity	XLOC_003369	c10329_g1_i1:0-677	cdRNA01-preDia	cdRNA05-postDia	7.68545	40.6972	2.40473	0.0001	0.0432976
RNA-Seq	Trinity	XLOC_003369	c10329_g1_i1:0-677	cdRNA02-Dia-R1	cdRNA05-postDia	2.07617	40.6972	4.29293	5.00E-05	0.0249845
RNA-Seq	Trinity	XLOC_003388	c103319_g1_i1:0-288	cdRNA04-Dia-R3	cdRNA05-postDia	215.637	24.1024	-3.16135	5.00E-05	0.0249845
RNA-Seq	Trinity	XLOC_003388	c103319_g1_i1:0-288	cdRNA03-Dia-R2	cdRNA05-postDia	212.502	24.1024	-3.14022	0.0001	0.0432976
RNA-Seq	Trinity	XLOC_003865	c1037_g1_i1:1-1419	cdRNA04-Dia-R3	cdRNA05-postDia	81.458	13.6243	-2.57987	5.00E-05	0.0249845
RNA-Seq	Trinity	XLOC_003865	c1037_g1_i1:1-1419	cdRNA03-Dia-R2	cdRNA05-postDia	77.1758	13.6243	-2.50196	5.00E-05	0.0249845
RNA-Seq	Trinity	XLOC_003909	c10384_g1_i2:0-353	cdRNA03-Dia-R2	cdRNA05-postDia	3.95237	89.7112	4.5045	5.00E-05	0.0249845
RNA-Seq	Trinity	XLOC_003909	c10384_g1_i2:0-353	cdRNA04-Dia-R3	cdRNA05-postDia	3.03802	89.7112	4.88409	5.00E-05	0.0249845
RNA-Seq	Trinity	XLOC_003990	c10392_g1_i1:0-2087	cdRNA03-Dia-R2	cdRNA05-postDia	4.39953	36.3843	3.04789	5.00E-05	0.0249845
RNA-Seq	Trinity	XLOC_003990	c10392_g1_i1:0-2087	cdRNA02-Dia-R1	cdRNA05-postDia	4.31451	36.3843	3.07604	5.00E-05	0.0249845
RNA-Seq	Trinity	XLOC_003990	c10392_g1_i1:0-2087	cdRNA04-Dia-R3	cdRNA05-postDia	3.90509	36.3843	3.21988	5.00E-05	0.0249845
RNA-Seq	Trinity	XLOC_003990	c10392_g1_i1:0-2087	cdRNA01-preDia	cdRNA05-postDia	1.76271	36.3843	4.36745	5.00E-05	0.0249845
RNA-Seq	Trinity	XLOC_004062	c104001_g1_i1:0-219	cdRNA01-preDia	cdRNA05-postDia	14.4482	336.221	4.54045	5.00E-05	0.0249845
RNA-Seq	Trinity	XLOC_004218	c10415_g1_i1:0-1844	cdRNA02-Dia-R1	cdRNA05-postDia	16.629	1.21703	-3.77226	5.00E-05	0.0249845
RNA-Seq	Trinity	XLOC_004218	c10415_g1_i1:0-1844	cdRNA04-Dia-R3	cdRNA05-postDia	15.0619	1.21703	-3.62946	5.00E-05	0.0249845
RNA-Seq	Trinity	XLOC_004218	c10415_g1_i1:0-1844	cdRNA03-Dia-R2	cdRNA05-postDia	14.8211	1.21703	-3.60621	5.00E-05	0.0249845
RNA-Seq	Trinity	XLOC_004218	c10415_g1_i1:0-1844	cdRNA01-preDia	cdRNA05-postDia	14.1029	1.21703	-3.53455	5.00E-05	0.0249845
RNA-Seq	Trinity	XLOC_004264	c10420_g1_i1:0-1664	cdRNA02-Dia-R1	cdRNA05-postDia	5.25878	45.0966	3.10022	5.00E-05	0.0249845
RNA-Seq	Trinity	XLOC_004264	c10420_g1_i1:0-1664	cdRNA03-Dia-R2	cdRNA05-postDia	4.37723	45.0966	3.36493	5.00E-05	0.0249845
RNA-Seq	Trinity	XLOC_004264	c10420_g1_i1:0-1664	cdRNA04-Dia-R3	cdRNA05-postDia	4.024	45.0966	3.48632	5.00E-05	0.0249845
RNA-Seq	Trinity	XLOC_004264	c10420_g1_i1:0-1664	cdRNA01-preDia	cdRNA05-postDia	1.65093	45.0966	4.77167	5.00E-05	0.0249845
RNA-Seq	Trinity	XLOC_004853	c104838_g1_i1:2-298	cdRNA01-preDia	cdRNA05-postDia	134.184	4.96674	-4.75577	5.00E-05	0.0249845
RNA-Seq	Trinity	XLOC_004853	c104838_g1_i1:2-298	cdRNA01-preDia	cdRNA02-Dia-R1	134.184	5.56281	-4.59226	5.00E-05	0.0249845
RNA-Seq	Trinity	XLOC_004853	c104838_g1_i1:2-298	cdRNA01-preDia	cdRNA03-Dia-R2	134.184	7.78384	-4.10759	5.00E-05	0.0249845
RNA-Seq	Trinity	XLOC_004853	c104838_g1_i1:2-298	cdRNA01-preDia	cdRNA04-Dia-R3	134.184	8.26612	-4.02086	5.00E-05	0.0249845
RNA-Seq	Trinity	XLOC_005006	c105018_g1_i1:2-308	cdRNA01-preDia	cdRNA05-postDia	12.3876	90.8703	2.87491	5.00E-05	0.0249845
RNA-Seq	Trinity	XLOC_005006	c105018_g1_i1:2-308	cdRNA02-Dia-R1	cdRNA05-postDia	7.35048	90.8703	3.6279	0.0001	0.0432976
RNA-Seq	Trinity	XLOC_005196	c10570_g1_i3:0-462	cdRNA03-Dia-R2	cdRNA05-postDia	7.03327	41.0895	2.5465	5.00E-05	0.0249845
RNA-Seq	Trinity	XLOC_005196	c10570_g1_i3:0-462	cdRNA04-Dia-R3	cdRNA05-postDia	5.43555	41.0895	2.91827	5.00E-05	0.0249845
RNA-Seq	Trinity	XLOC_005196	c10570_g1_i3:0-462	cdRNA01-preDia	cdRNA05-postDia	4.13501	41.0895	3.31281	0.0001	0.0432976
RNA-Seq	Trinity	XLOC_005264	c10620_g1_i1:0-2648	cdRNA02-Dia-R1	cdRNA05-postDia	1.14458	72.1444	5.978	5.00E-05	0.0249845
RNA-Seq	Trinity	XLOC_005264	c10620_g1_i1:0-2648	cdRNA04-Dia-R3	cdRNA05-postDia	0.418739	72.1444	7.42869	5.00E-05	0.0249845
RNA-Seq	Trinity	XLOC_005264	c10620_g1_i1:0-2648	cdRNA03-Dia-R2	cdRNA05-postDia	0.38739	72.1444	7.54096	5.00E-05	0.0249845
RNA-Seq	Trinity	XLOC_005384	c10701_g2_i3:0-264	cdRNA01-preDia	cdRNA05-postDia	13.6577	150.959	3.46637	5.00E-05	0.0249845
RNA-Seq	Trinity	XLOC_005388	c10704_g1_i2:0-883	cdRNA01-preDia	cdRNA02-Dia-R1	0.814713	15.6025	4.25934	0.0001	0.0432976
RNA-Seq	Trinity	XLOC_005388	c10704_g1_i2:0-883	cdRNA01-preDia	cdRNA05-postDia	0.814713	33.0462	5.34205	5.00E-05	0.0249845
RNA-Seq	Trinity	XLOC_005395	c10708_g1_i1:0-942	cdRNA03-Dia-R2	cdRNA05-postDia	9.6751	45.6875	2.23945	5.00E-05	0.0249845
RNA-Seq	Trinity	XLOC_005395	c10708_g1_i1:0-942	cdRNA01-preDia	cdRNA05-postDia	9.04851	45.6875	2.33605	5.00E-05	0.0249845
RNA-Seq	Trinity	XLOC_005395	c10708_g1_i1:0-942	cdRNA04-Dia-R3	cdRNA05-postDia	8.52565	45.6875	2.42192	0.0001	0.0432976
RNA-Seq	Trinity	XLOC_005400	c10710_g1_i2:9-913	cdRNA03-Dia-R2	cdRNA05-postDia	5.35375	46.915	3.13143	5.00E-05	0.0249845
RNA-Seq	Trinity	XLOC_005400	c10710_g1_i2:9-913	cdRNA04-Dia-R3	cdRNA05-postDia	4.60252	46.915	3.34955	5.00E-05	0.0249845
RNA-Seq	Trinity	XLOC_005402	c10710_g2_i1:3-239	cdRNA02-Dia-R1	cdRNA05-postDia	184.682	18.7132	-3.30292	5.00E-05	0.0249845
RNA-Seq	Trinity	XLOC_005455	c10739_g1_i1:0-2959	cdRNA01-preDia	cdRNA04-Dia-R3	5.72274	41.0818	2.84372	5.00E-05	0.0249845
RNA-Seq	Trinity	XLOC_005455	c10739_g1_i1:0-2959	cdRNA01-preDia	cdRNA03-Dia-R2	5.72274	43.9741	2.94188	5.00E-05	0.0249845
RNA-Seq	Trinity	XLOC_005463	c10744_g2_i1:5-219	cdRNA01-preDia	cdRNA05-postDia	17.8535	298.254	4.06226	0.0001	0.0432976
RNA-Seq	Trinity	XLOC_005464	c10746_g1_i1:0-1065	cdRNA02-Dia-R1	cdRNA04-Dia-R3	13.1685	1.58481	-3.05472	5.00E-05	0.0249845
RNA-Seq	Trinity	XLOC_005464	c10746_g1_i1:0-1065	cdRNA02-Dia-R1	cdRNA05-postDia	13.1685	77.5347	2.55775	5.00E-05	0.0249845

RNA-Seq	Trinity	XLOC_005464	c10746_g1_i1:0-1065	cdRNA01-preDia	cdRNA05-postDia	11.3355	77.5347	2.77399	5.00E-05	0.0249845
RNA-Seq	Trinity	XLOC_005464	c10746_g1_i1:0-1065	cdRNA03-Dia-R2	cdRNA05-postDia	1.87852	77.5347	5.36718	5.00E-05	0.0249845
RNA-Seq	Trinity	XLOC_005464	c10746_g1_i1:0-1065	cdRNA04-Dia-R3	cdRNA05-postDia	1.58481	77.5347	5.61246	5.00E-05	0.0249845
RNA-Seq	Trinity	XLOC_005473	c10755_g1_i1:13-2004	cdRNA01-preDia	cdRNA05-postDia	35.2464	330.887	3.23079	5.00E-05	0.0249845
RNA-Seq	Trinity	XLOC_005473	c10755_g1_i1:13-2004	cdRNA03-Dia-R2	cdRNA05-postDia	13.8193	330.887	4.58158	5.00E-05	0.0249845
RNA-Seq	Trinity	XLOC_005473	c10755_g1_i1:13-2004	cdRNA04-Dia-R3	cdRNA05-postDia	13.7658	330.887	4.58718	5.00E-05	0.0249845
RNA-Seq	Trinity	XLOC_005474	c10755_g2_i1:3-2262	cdRNA02-Dia-R1	cdRNA05-postDia	10.8175	178.739	4.04641	5.00E-05	0.0249845
RNA-Seq	Trinity	XLOC_005474	c10755_g2_i1:3-2262	cdRNA01-preDia	cdRNA05-postDia	5.24931	178.739	5.08958	5.00E-05	0.0249845
RNA-Seq	Trinity	XLOC_005474	c10755_g2_i1:3-2262	cdRNA03-Dia-R2	cdRNA05-postDia	3.70496	178.739	5.59225	5.00E-05	0.0249845
RNA-Seq	Trinity	XLOC_005474	c10755_g2_i1:3-2262	cdRNA04-Dia-R3	cdRNA05-postDia	3.44913	178.739	5.69547	5.00E-05	0.0249845
RNA-Seq	Trinity	XLOC_005475	c10755_g2_i2:0-2532	cdRNA01-preDia	cdRNA05-postDia	13.2902	150.468	3.50103	5.00E-05	0.0249845
RNA-Seq	Trinity	XLOC_005475	c10755_g2_i2:0-2532	cdRNA03-Dia-R2	cdRNA05-postDia	6.15123	150.468	4.61244	5.00E-05	0.0249845
RNA-Seq	Trinity	XLOC_005475	c10755_g2_i2:0-2532	cdRNA04-Dia-R3	cdRNA05-postDia	5.87767	150.468	4.67807	5.00E-05	0.0249845
RNA-Seq	Trinity	XLOC_005480	c10757_g2_i1:0-5591	cdRNA01-preDia	cdRNA03-Dia-R2	25.1741	193.695	2.94378	5.00E-05	0.0249845
RNA-Seq	Trinity	XLOC_005501	c10770_g1_i2:40-1984	cdRNA02-Dia-R1	cdRNA05-postDia	8.12388	68.6476	3.07897	5.00E-05	0.0249845
RNA-Seq	Trinity	XLOC_005501	c10770_g1_i2:40-1984	cdRNA01-preDia	cdRNA05-postDia	3.47451	68.6476	4.30433	5.00E-05	0.0249845
RNA-Seq	Trinity	XLOC_005501	c10770_g1_i2:40-1984	cdRNA03-Dia-R2	cdRNA05-postDia	3.37812	68.6476	4.34492	5.00E-05	0.0249845
RNA-Seq	Trinity	XLOC_005501	c10770_g1_i2:40-1984	cdRNA04-Dia-R3	cdRNA05-postDia	2.94079	68.6476	4.54493	5.00E-05	0.0249845
RNA-Seq	Trinity	XLOC_005593	c10821_g1_i1:0-1680	cdRNA01-preDia	cdRNA05-postDia	7.60296	40.3684	2.40859	5.00E-05	0.0249845
RNA-Seq	Trinity	XLOC_005593	c10821_g1_i1:0-1680	cdRNA01-preDia	cdRNA04-Dia-R3	7.60296	65.5607	3.1082	5.00E-05	0.0249845
RNA-Seq	Trinity	XLOC_005593	c10821_g1_i1:0-1680	cdRNA01-preDia	cdRNA03-Dia-R2	7.60296	65.7218	3.11174	5.00E-05	0.0249845
RNA-Seq	Trinity	XLOC_005595	c10823_g2_i1:0-1937	cdRNA03-Dia-R2	cdRNA05-postDia	539.54	70.4887	-2.93627	5.00E-05	0.0249845
RNA-Seq	Trinity	XLOC_005595	c10823_g2_i1:0-1937	cdRNA04-Dia-R3	cdRNA05-postDia	539.005	70.4887	-2.93483	5.00E-05	0.0249845
RNA-Seq	Trinity	XLOC_005595	c10823_g2_i1:0-1937	cdRNA01-preDia	cdRNA02-Dia-R1	12.6773	404.984	4.99754	5.00E-05	0.0249845
RNA-Seq	Trinity	XLOC_005595	c10823_g2_i1:0-1937	cdRNA01-preDia	cdRNA04-Dia-R3	12.6773	539.005	5.40998	5.00E-05	0.0249845
RNA-Seq	Trinity	XLOC_005595	c10823_g2_i1:0-1937	cdRNA01-preDia	cdRNA03-Dia-R2	12.6773	539.54	5.41141	5.00E-05	0.0249845
RNA-Seq	Trinity	XLOC_005653	c10879_g1_i1:0-1256	cdRNA02-Dia-R1	cdRNA04-Dia-R3	9.92906	1.76546	-2.49161	0.0001	0.0432976
RNA-Seq	Trinity	XLOC_005653	c10879_g1_i1:0-1256	cdRNA02-Dia-R1	cdRNA05-postDia	9.92906	60.5785	2.60908	5.00E-05	0.0249845
RNA-Seq	Trinity	XLOC_005653	c10879_g1_i1:0-1256	cdRNA01-preDia	cdRNA05-postDia	4.31948	60.5785	3.80988	5.00E-05	0.0249845
RNA-Seq	Trinity	XLOC_005653	c10879_g1_i1:0-1256	cdRNA04-Dia-R3	cdRNA05-postDia	1.76546	60.5785	5.10069	5.00E-05	0.0249845
RNA-Seq	Trinity	XLOC_005653	c10879_g1_i1:0-1256	cdRNA03-Dia-R2	cdRNA05-postDia	1.42496	60.5785	5.40981	5.00E-05	0.0249845
RNA-Seq	Trinity	XLOC_005792	c11008_g1_i1:1-967	cdRNA04-Dia-R3	cdRNA05-postDia	18.0065	2.35365	-2.93555	5.00E-05	0.0249845
RNA-Seq	Trinity	XLOC_005792	c11008_g1_i1:1-967	cdRNA03-Dia-R2	cdRNA05-postDia	16.5664	2.35365	-2.81529	5.00E-05	0.0249845
RNA-Seq	Trinity	XLOC_005833	c11046_g1_i1:0-1195	cdRNA02-Dia-R1	cdRNA05-postDia	6.57638	36.7183	2.48113	0.0001	0.0432976
RNA-Seq	Trinity	XLOC_005833	c11046_g1_i1:0-1195	cdRNA03-Dia-R2	cdRNA05-postDia	6.0905	36.7183	2.59187	5.00E-05	0.0249845
RNA-Seq	Trinity	XLOC_005833	c11046_g1_i1:0-1195	cdRNA04-Dia-R3	cdRNA05-postDia	5.85188	36.7183	2.64953	5.00E-05	0.0249845
RNA-Seq	Trinity	XLOC_005957	c11163_g1_i1:40-622	cdRNA04-Dia-R3	cdRNA05-postDia	80.8186	554.693	2.77893	5.00E-05	0.0249845
RNA-Seq	Trinity	XLOC_005957	c11163_g1_i1:40-622	cdRNA03-Dia-R2	cdRNA05-postDia	79.418	554.693	2.80415	5.00E-05	0.0249845
RNA-Seq	Trinity	XLOC_005982	c11188_g1_i1:3-737	cdRNA03-Dia-R2	cdRNA05-postDia	37.2155	194.085	2.38271	0.0001	0.0432976
RNA-Seq	Trinity	XLOC_006090	c11287_g1_i1:0-2242	cdRNA01-preDia	cdRNA03-Dia-R2	7.46463	75.5496	3.33928	5.00E-05	0.0249845
RNA-Seq	Trinity	XLOC_006090	c11287_g1_i1:0-2242	cdRNA01-preDia	cdRNA04-Dia-R3	7.46463	77.6484	3.37881	5.00E-05	0.0249845
RNA-Seq	Trinity	XLOC_006344	c11518_g1_i1:0-1010	cdRNA04-Dia-R3	cdRNA05-postDia	5.07884	25.2185	2.31191	0.0001	0.0432976
RNA-Seq	Trinity	XLOC_006344	c11518_g1_i1:0-1010	cdRNA03-Dia-R2	cdRNA05-postDia	4.69862	25.2185	2.42417	5.00E-05	0.0249845
RNA-Seq	Trinity	XLOC_006353	c11526_g1_i1:0-1564	cdRNA03-Dia-R2	cdRNA05-postDia	6.23643	0.70333	-3.14845	5.00E-05	0.0249845
RNA-Seq	Trinity	XLOC_006353	c11526_g1_i1:0-1564	cdRNA04-Dia-R3	cdRNA05-postDia	5.96422	0.70333	-3.08406	5.00E-05	0.0249845
RNA-Seq	Trinity	XLOC_006365	c11539_g1_i1:0-1869	cdRNA02-Dia-R1	cdRNA04-Dia-R3	11.6713	1.61631	-2.85219	5.00E-05	0.0249845
RNA-Seq	Trinity	XLOC_006365	c11539_g1_i1:0-1869	cdRNA02-Dia-R1	cdRNA03-Dia-R2	11.6713	1.75329	-2.73482	5.00E-05	0.0249845

RNA-Seq	Trinity	XLOC_006768	c1190_g1_i1:0-839	cdRNA01-preDia	cdRNA04-Dia-R3	18.6678	3.10387	-2.58841	5.00E-05	0.0249845
RNA-Seq	Trinity	XLOC_006768	c1190_g1_i1:0-839	cdRNA04-Dia-R3	cdRNA05-postDia	3.10387	23.5364	2.92275	5.00E-05	0.0249845
RNA-Seq	Trinity	XLOC_006819	c11956_g2_i1:12-389	cdRNA02-Dia-R1	cdRNA05-postDia	29.3691	181.024	2.62382	5.00E-05	0.0249845
RNA-Seq	Trinity	XLOC_006819	c11956_g2_i1:12-389	cdRNA01-preDia	cdRNA05-postDia	11.6304	181.024	3.96021	5.00E-05	0.0249845
RNA-Seq	Trinity	XLOC_006902	c12030_g1_i1:0-1353	cdRNA03-Dia-R2	cdRNA05-postDia	111.206	19.8409	-2.48668	0.0001	0.0432976
RNA-Seq	Trinity	XLOC_006983	c12107_g1_i1:0-475	cdRNA03-Dia-R2	cdRNA05-postDia	3.49351	25.2947	2.85609	0.0001	0.0432976
RNA-Seq	Trinity	XLOC_007097	c12215_g1_i1:0-1141	cdRNA02-Dia-R1	cdRNA05-postDia	4.00173	22.2883	2.47759	5.00E-05	0.0249845
RNA-Seq	Trinity	XLOC_007097	c12215_g1_i1:0-1141	cdRNA03-Dia-R2	cdRNA05-postDia	3.57861	22.2883	2.63882	5.00E-05	0.0249845
RNA-Seq	Trinity	XLOC_007097	c12215_g1_i1:0-1141	cdRNA04-Dia-R3	cdRNA05-postDia	3.03388	22.2883	2.87705	5.00E-05	0.0249845
RNA-Seq	Trinity	XLOC_007218	c12324_g1_i1:0-716	cdRNA04-Dia-R3	cdRNA05-postDia	2.44296	20.083	3.03928	0.0001	0.0432976
RNA-Seq	Trinity	XLOC_007218	c12324_g1_i1:0-716	cdRNA03-Dia-R2	cdRNA05-postDia	2.38848	20.083	3.07181	0.0001	0.0432976
RNA-Seq	Trinity	XLOC_007218	c12324_g1_i1:0-716	cdRNA02-Dia-R1	cdRNA05-postDia	1.51117	20.083	3.73224	0.0001	0.0432976
RNA-Seq	Trinity	XLOC_007218	c12324_g1_i1:0-716	cdRNA01-preDia	cdRNA05-postDia	1.15762	20.083	4.11675	5.00E-05	0.0249845
RNA-Seq	Trinity	XLOC_007437	c12527_g1_i1:0-1834	cdRNA03-Dia-R2	cdRNA05-postDia	11.2781	2.1517	-2.38997	5.00E-05	0.0249845
RNA-Seq	Trinity	XLOC_007493	c12580_g1_i1:1-682	cdRNA02-Dia-R1	cdRNA05-postDia	16.5325	82.4666	2.31851	5.00E-05	0.0249845
RNA-Seq	Trinity	XLOC_007493	c12580_g1_i1:1-682	cdRNA03-Dia-R2	cdRNA05-postDia	16.4575	82.4666	2.32506	5.00E-05	0.0249845
RNA-Seq	Trinity	XLOC_007641	c12717_g1_i1:0-585	cdRNA04-Dia-R3	cdRNA05-postDia	87.9419	15.4815	-2.50601	5.00E-05	0.0249845
RNA-Seq	Trinity	XLOC_007641	c12717_g1_i1:0-585	cdRNA03-Dia-R2	cdRNA05-postDia	83.4519	15.4815	-2.4304	0.0001	0.0432976
RNA-Seq	Trinity	XLOC_007658	c12732_g1_i1:0-1618	cdRNA01-preDia	cdRNA03-Dia-R2	16.8787	2.95729	-2.51286	5.00E-05	0.0249845
RNA-Seq	Trinity	XLOC_007658	c12732_g1_i1:0-1618	cdRNA01-preDia	cdRNA04-Dia-R3	16.8787	3.24351	-2.37957	5.00E-05	0.0249845
RNA-Seq	Trinity	XLOC_007658	c12732_g1_i1:0-1618	cdRNA03-Dia-R2	cdRNA05-postDia	2.95729	14.5441	2.29809	5.00E-05	0.0249845
RNA-Seq	Trinity	XLOC_007800	c12864_g1_i1:0-1669	cdRNA03-Dia-R2	cdRNA05-postDia	14.9449	2.66609	-2.48685	5.00E-05	0.0249845
RNA-Seq	Trinity	XLOC_007800	c12864_g1_i1:0-1669	cdRNA04-Dia-R3	cdRNA05-postDia	14.437	2.66609	-2.43697	5.00E-05	0.0249845
RNA-Seq	Trinity	XLOC_007998	c13050_g1_i1:0-218	cdRNA04-Dia-R3	cdRNA05-postDia	47.2502	315.014	2.73702	5.00E-05	0.0249845
RNA-Seq	Trinity	XLOC_007998	c13050_g1_i1:0-218	cdRNA02-Dia-R1	cdRNA05-postDia	33.9401	315.014	3.21435	5.00E-05	0.0249845
RNA-Seq	Trinity	XLOC_008085	c13134_g1_i1:0-1226	cdRNA04-Dia-R3	cdRNA05-postDia	4.45049	27.8051	2.64331	5.00E-05	0.0249845
RNA-Seq	Trinity	XLOC_008085	c13134_g1_i1:0-1226	cdRNA03-Dia-R2	cdRNA05-postDia	4.36177	27.8051	2.67236	5.00E-05	0.0249845
RNA-Seq	Trinity	XLOC_008085	c13134_g1_i1:0-1226	cdRNA02-Dia-R1	cdRNA05-postDia	3.26125	27.8051	3.09185	5.00E-05	0.0249845
RNA-Seq	Trinity	XLOC_008461	c13487_g1_i1:0-1057	cdRNA04-Dia-R3	cdRNA05-postDia	21.1512	3.73788	-2.50045	0.0001	0.0432976
RNA-Seq	Trinity	XLOC_008548	c13572_g1_i1:0-1060	cdRNA01-preDia	cdRNA03-Dia-R2	17.0334	1.52081	-3.48545	5.00E-05	0.0249845
RNA-Seq	Trinity	XLOC_008548	c13572_g1_i1:0-1060	cdRNA03-Dia-R2	cdRNA05-postDia	1.52081	11.8727	2.96474	5.00E-05	0.0249845
RNA-Seq	Trinity	XLOC_008548	c13572_g1_i1:0-1060	cdRNA04-Dia-R3	cdRNA05-postDia	1.37266	11.8727	3.1126	0.0001	0.0432976
RNA-Seq	Trinity	XLOC_008571	c13595_g1_i1:0-435	cdRNA01-preDia	cdRNA03-Dia-R2	69.6164	4.95224	-3.81327	5.00E-05	0.0249845
RNA-Seq	Trinity	XLOC_008571	c13595_g1_i1:0-435	cdRNA01-preDia	cdRNA04-Dia-R3	69.6164	8.03974	-3.11421	5.00E-05	0.0249845
RNA-Seq	Trinity	XLOC_008571	c13595_g1_i1:0-435	cdRNA02-Dia-R1	cdRNA03-Dia-R2	33.751	4.95224	-2.76878	5.00E-05	0.0249845
RNA-Seq	Trinity	XLOC_008599	c13619_g1_i1:10-894	cdRNA01-preDia	cdRNA05-postDia	45.673	4.80604	-3.24842	5.00E-05	0.0249845
RNA-Seq	Trinity	XLOC_008599	c13619_g1_i1:10-894	cdRNA01-preDia	cdRNA03-Dia-R2	45.673	5.4532	-3.06617	5.00E-05	0.0249845
RNA-Seq	Trinity	XLOC_008599	c13619_g1_i1:10-894	cdRNA01-preDia	cdRNA04-Dia-R3	45.673	5.90843	-2.9505	5.00E-05	0.0249845
RNA-Seq	Trinity	XLOC_008745	c13759_g1_i1:0-857	cdRNA02-Dia-R1	cdRNA05-postDia	4.64837	22.5824	2.2804	5.00E-05	0.0249845
RNA-Seq	Trinity	XLOC_008774	c13786_g1_i1:0-345	cdRNA02-Dia-R1	cdRNA05-postDia	39.4112	957.662	4.60284	5.00E-05	0.0249845
RNA-Seq	Trinity	XLOC_008774	c13786_g1_i1:0-345	cdRNA03-Dia-R2	cdRNA05-postDia	11.3937	957.662	6.39321	5.00E-05	0.0249845
RNA-Seq	Trinity	XLOC_008774	c13786_g1_i1:0-345	cdRNA01-preDia	cdRNA05-postDia	10.8557	957.662	6.46299	5.00E-05	0.0249845
RNA-Seq	Trinity	XLOC_008774	c13786_g1_i1:0-345	cdRNA04-Dia-R3	cdRNA05-postDia	7.20919	957.662	7.05354	5.00E-05	0.0249845
RNA-Seq	Trinity	XLOC_008895	c138_g1_i1:0-2145	cdRNA01-preDia	cdRNA02-Dia-R1	1.18074	8.07987	2.77464	5.00E-05	0.0249845
RNA-Seq	Trinity	XLOC_008895	c138_g1_i1:0-2145	cdRNA01-preDia	cdRNA03-Dia-R2	1.18074	18.0682	3.93569	5.00E-05	0.0249845
RNA-Seq	Trinity	XLOC_008895	c138_g1_i1:0-2145	cdRNA01-preDia	cdRNA04-Dia-R3	1.18074	18.6296	3.97983	5.00E-05	0.0249845
RNA-Seq	Trinity	XLOC_008895	c138_g1_i1:0-2145	cdRNA01-preDia	cdRNA05-postDia	1.18074	24.9482	4.40117	5.00E-05	0.0249845

RNA-Seq	Trinity	XLOC_009345	c1432_g1_i1:0-1313	cdRNA04-Dia-R3	cdRNA05-postDia	3.89629	32.0208	3.03884	5.00E-05	0.0249845
RNA-Seq	Trinity	XLOC_009345	c1432_g1_i1:0-1313	cdRNA03-Dia-R2	cdRNA05-postDia	2.8813	32.0208	3.47421	5.00E-05	0.0249845
RNA-Seq	Trinity	XLOC_009359	c14342_g1_i1:0-968	cdRNA01-preDia	cdRNA02-Dia-R1	59.5808	3.15505	-4.23911	5.00E-05	0.0249845
RNA-Seq	Trinity	XLOC_009359	c14342_g1_i1:0-968	cdRNA01-preDia	cdRNA05-postDia	59.5808	4.74713	-3.64972	5.00E-05	0.0249845
RNA-Seq	Trinity	XLOC_009359	c14342_g1_i1:0-968	cdRNA01-preDia	cdRNA04-Dia-R3	59.5808	6.10222	-3.28744	5.00E-05	0.0249845
RNA-Seq	Trinity	XLOC_009359	c14342_g1_i1:0-968	cdRNA01-preDia	cdRNA03-Dia-R2	59.5808	6.35104	-3.22978	5.00E-05	0.0249845
RNA-Seq	Trinity	XLOC_009726	c14689_g1_i1:0-585	cdRNA03-Dia-R2	cdRNA05-postDia	30.8544	4.18419	-2.88246	0.0001	0.0432976
RNA-Seq	Trinity	XLOC_009726	c14689_g1_i1:0-585	cdRNA02-Dia-R1	cdRNA04-Dia-R3	2.57749	25.6663	3.31584	5.00E-05	0.0249845
RNA-Seq	Trinity	XLOC_009726	c14689_g1_i1:0-585	cdRNA02-Dia-R1	cdRNA03-Dia-R2	2.57749	30.8544	3.58144	5.00E-05	0.0249845
RNA-Seq	Trinity	XLOC_009726	c14689_g1_i1:0-585	cdRNA01-preDia	cdRNA03-Dia-R2	1.97046	30.8544	3.96887	5.00E-05	0.0249845
RNA-Seq	Trinity	XLOC_010003	c14947_g1_i1:1-721	cdRNA01-preDia	cdRNA05-postDia	5.28376	28.7325	2.44305	5.00E-05	0.0249845
RNA-Seq	Trinity	XLOC_010144	c15076_g1_i1:0-301	cdRNA01-preDia	cdRNA05-postDia	17.649	122.632	2.79668	5.00E-05	0.0249845
RNA-Seq	Trinity	XLOC_010144	c15076_g1_i1:0-301	cdRNA03-Dia-R2	cdRNA05-postDia	16.4453	122.632	2.89859	5.00E-05	0.0249845
RNA-Seq	Trinity	XLOC_010144	c15076_g1_i1:0-301	cdRNA04-Dia-R3	cdRNA05-postDia	14.6724	122.632	3.06317	5.00E-05	0.0249845
RNA-Seq	Trinity	XLOC_010144	c15076_g1_i1:0-301	cdRNA02-Dia-R1	cdRNA05-postDia	11.2243	122.632	3.44965	5.00E-05	0.0249845
RNA-Seq	Trinity	XLOC_010210	c15142_g1_i1:0-282	cdRNA04-Dia-R3	cdRNA05-postDia	46.8396	231.578	2.30569	0.0001	0.0432976
RNA-Seq	Trinity	XLOC_010210	c15142_g1_i1:0-282	cdRNA03-Dia-R2	cdRNA05-postDia	37.031	231.578	2.64469	5.00E-05	0.0249845
RNA-Seq	Trinity	XLOC_010566	c15480_g1_i1:0-286	cdRNA03-Dia-R2	cdRNA05-postDia	18.9203	107.409	2.50511	5.00E-05	0.0249845
RNA-Seq	Trinity	XLOC_010566	c15480_g1_i1:0-286	cdRNA02-Dia-R1	cdRNA05-postDia	11.894	107.409	3.17481	5.00E-05	0.0249845
RNA-Seq	Trinity	XLOC_010822	c15721_g1_i1:0-868	cdRNA03-Dia-R2	cdRNA05-postDia	2.07937	12.3646	2.572	5.00E-05	0.0249845
RNA-Seq	Trinity	XLOC_011803	c16632_g1_i1:0-1182	cdRNA04-Dia-R3	cdRNA05-postDia	0.930143	10.2694	3.46476	5.00E-05	0.0249845
RNA-Seq	Trinity	XLOC_011803	c16632_g1_i1:0-1182	cdRNA03-Dia-R2	cdRNA05-postDia	0.927735	10.2694	3.4685	5.00E-05	0.0249845
RNA-Seq	Trinity	XLOC_012034	c1684_g1_i1:0-2365	cdRNA01-preDia	cdRNA05-postDia	9.06941	53.4296	2.55856	5.00E-05	0.0249845
RNA-Seq	Trinity	XLOC_012471	c17265_g1_i1:0-984	cdRNA03-Dia-R2	cdRNA05-postDia	56.3915	9.04404	-2.64044	5.00E-05	0.0249845
RNA-Seq	Trinity	XLOC_012471	c17265_g1_i1:0-984	cdRNA04-Dia-R3	cdRNA05-postDia	53.6962	9.04404	-2.56978	5.00E-05	0.0249845
RNA-Seq	Trinity	XLOC_012471	c17265_g1_i1:0-984	cdRNA01-preDia	cdRNA04-Dia-R3	10.1317	53.6962	2.40594	5.00E-05	0.0249845
RNA-Seq	Trinity	XLOC_012471	c17265_g1_i1:0-984	cdRNA01-preDia	cdRNA03-Dia-R2	10.1317	56.3915	2.4766	0.0001	0.0432976
RNA-Seq	Trinity	XLOC_012494	c17287_g1_i1:1-441	cdRNA03-Dia-R2	cdRNA05-postDia	16.7149	104.7	2.64705	5.00E-05	0.0249845
RNA-Seq	Trinity	XLOC_012494	c17287_g1_i1:1-441	cdRNA04-Dia-R3	cdRNA05-postDia	13.9723	104.7	2.90562	5.00E-05	0.0249845
RNA-Seq	Trinity	XLOC_012508	c1729_g1_i1:0-764	cdRNA03-Dia-R2	cdRNA05-postDia	22.4716	3.13145	-2.8432	0.0001	0.0432976
RNA-Seq	Trinity	XLOC_012508	c1729_g1_i1:0-764	cdRNA04-Dia-R3	cdRNA05-postDia	21.9973	3.13145	-2.81242	0.0001	0.0432976
RNA-Seq	Trinity	XLOC_012791	c17565_g1_i1:0-357	cdRNA01-preDia	cdRNA05-postDia	29.3236	149.442	2.34945	0.0001	0.0432976
RNA-Seq	Trinity	XLOC_012791	c17565_g1_i1:0-357	cdRNA03-Dia-R2	cdRNA05-postDia	25.937	149.442	2.5265	5.00E-05	0.0249845
RNA-Seq	Trinity	XLOC_012791	c17565_g1_i1:0-357	cdRNA02-Dia-R1	cdRNA05-postDia	22.8593	149.442	2.70873	5.00E-05	0.0249845
RNA-Seq	Trinity	XLOC_013042	c17793_g1_i1:0-623	cdRNA03-Dia-R2	cdRNA05-postDia	10.4505	51.554	2.30252	0.0001	0.0432976
RNA-Seq	Trinity	XLOC_013152	c17898_g1_i1:0-378	cdRNA03-Dia-R2	cdRNA05-postDia	101.872	19.4407	-2.38961	5.00E-05	0.0249845
RNA-Seq	Trinity	XLOC_013152	c17898_g1_i1:0-378	cdRNA04-Dia-R3	cdRNA05-postDia	100.273	19.4407	-2.36679	5.00E-05	0.0249845
RNA-Seq	Trinity	XLOC_013781	c18493_g1_i1:0-824	cdRNA04-Dia-R3	cdRNA05-postDia	16.0782	1.86418	-3.10849	5.00E-05	0.0249845
RNA-Seq	Trinity	XLOC_013781	c18493_g1_i1:0-824	cdRNA03-Dia-R2	cdRNA05-postDia	15.2068	1.86418	-3.0281	5.00E-05	0.0249845
RNA-Seq	Trinity	XLOC_013781	c18493_g1_i1:0-824	cdRNA02-Dia-R1	cdRNA04-Dia-R3	2.92787	16.0782	2.45719	5.00E-05	0.0249845
RNA-Seq	Trinity	XLOC_013897	c185_g1_i1:0-677	cdRNA02-Dia-R1	cdRNA05-postDia	6.10269	37.1584	2.60617	5.00E-05	0.0249845
RNA-Seq	Trinity	XLOC_014761	c19439_g1_i1:0-688	cdRNA01-preDia	cdRNA05-postDia	3.91867	22.9741	2.55157	0.0001	0.0432976
RNA-Seq	Trinity	XLOC_015727	c20367_g1_i1:0-641	cdRNA01-preDia	cdRNA03-Dia-R2	33.1838	3.88164	-3.09574	5.00E-05	0.0249845
RNA-Seq	Trinity	XLOC_015727	c20367_g1_i1:0-641	cdRNA01-preDia	cdRNA04-Dia-R3	33.1838	4.24996	-2.96496	5.00E-05	0.0249845
RNA-Seq	Trinity	XLOC_016567	c21175_g1_i1:0-470	cdRNA01-preDia	cdRNA05-postDia	11.4378	87.9481	2.94284	5.00E-05	0.0249845
RNA-Seq	Trinity	XLOC_016567	c21175_g1_i1:0-470	cdRNA04-Dia-R3	cdRNA05-postDia	10.6867	87.9481	3.04084	5.00E-05	0.0249845
RNA-Seq	Trinity	XLOC_016567	c21175_g1_i1:0-470	cdRNA03-Dia-R2	cdRNA05-postDia	10.5292	87.9481	3.06225	5.00E-05	0.0249845

RNA-Seq	Trinity	XLOC_016567	c21175_g1_i1:0-470	cdRNA02-Dia-R1	cdRNA05-postDia	10.4041	87.9481	3.07951	5.00E-05	0.0249845
RNA-Seq	Trinity	XLOC_017215	c2179_g1_i1:0-420	cdRNA01-preDia	cdRNA02-Dia-R1	99.6882	17.6329	-2.49915	5.00E-05	0.0249845
RNA-Seq	Trinity	XLOC_017820	c2239_g1_i2:16-501	cdRNA04-Dia-R3	cdRNA05-postDia	3.77672	28.7913	2.93042	0.0001	0.0432976
RNA-Seq	Trinity	XLOC_017820	c2239_g1_i2:16-501	cdRNA02-Dia-R1	cdRNA05-postDia	3.3888	28.7913	3.08678	5.00E-05	0.0249845
RNA-Seq	Trinity	XLOC_018510	c23080_g1_i1:0-587	cdRNA01-preDia	cdRNA05-postDia	80.7798	2.60146	-4.9566	5.00E-05	0.0249845
RNA-Seq	Trinity	XLOC_018510	c23080_g1_i1:0-587	cdRNA01-preDia	cdRNA02-Dia-R1	80.7798	2.87482	-4.81245	5.00E-05	0.0249845
RNA-Seq	Trinity	XLOC_018510	c23080_g1_i1:0-587	cdRNA01-preDia	cdRNA04-Dia-R3	80.7798	5.04708	-4.00048	5.00E-05	0.0249845
RNA-Seq	Trinity	XLOC_018510	c23080_g1_i1:0-587	cdRNA01-preDia	cdRNA03-Dia-R2	80.7798	5.35588	-3.9148	5.00E-05	0.0249845
RNA-Seq	Trinity	XLOC_018521	c23092_g1_i1:11-500	cdRNA04-Dia-R3	cdRNA05-postDia	7.18145	44.8881	2.64399	5.00E-05	0.0249845
RNA-Seq	Trinity	XLOC_018521	c23092_g1_i1:11-500	cdRNA03-Dia-R2	cdRNA05-postDia	6.09016	44.8881	2.88178	5.00E-05	0.0249845
RNA-Seq	Trinity	XLOC_018656	c23223_g1_i1:0-700	cdRNA02-Dia-R1	cdRNA04-Dia-R3	4.07979	21.0911	2.37007	5.00E-05	0.0249845
RNA-Seq	Trinity	XLOC_018656	c23223_g1_i1:0-700	cdRNA01-preDia	cdRNA03-Dia-R2	2.31025	19.8037	3.09965	5.00E-05	0.0249845
RNA-Seq	Trinity	XLOC_018656	c23223_g1_i1:0-700	cdRNA01-preDia	cdRNA04-Dia-R3	2.31025	21.0911	3.19051	5.00E-05	0.0249845
RNA-Seq	Trinity	XLOC_018680	c2324_g1_i1:0-1182	cdRNA02-Dia-R1	cdRNA05-postDia	2.95155	14.5891	2.30535	5.00E-05	0.0249845
RNA-Seq	Trinity	XLOC_018758	c2332_g1_i1:0-660	cdRNA01-preDia	cdRNA03-Dia-R2	26.3421	1.47066	-4.16284	0.0001	0.0432976
RNA-Seq	Trinity	XLOC_018758	c2332_g1_i1:0-660	cdRNA01-preDia	cdRNA04-Dia-R3	26.3421	1.57928	-4.06003	5.00E-05	0.0249845
RNA-Seq	Trinity	XLOC_019122	c236_g1_i2:0-1921	cdRNA04-Dia-R3	cdRNA05-postDia	1.83226	12.8596	2.81115	5.00E-05	0.0249845
RNA-Seq	Trinity	XLOC_019122	c236_g1_i2:0-1921	cdRNA03-Dia-R2	cdRNA05-postDia	1.56234	12.8596	3.04106	5.00E-05	0.0249845
RNA-Seq	Trinity	XLOC_020363	c2493_g1_i2:17-1771	cdRNA04-Dia-R3	cdRNA05-postDia	2.1764	11.4938	2.40084	0.0001	0.0432976
RNA-Seq	Trinity	XLOC_020363	c2493_g1_i2:17-1771	cdRNA02-Dia-R1	cdRNA05-postDia	1.19036	11.4938	3.27138	5.00E-05	0.0249845
RNA-Seq	Trinity	XLOC_020363	c2493_g1_i2:17-1771	cdRNA01-preDia	cdRNA05-postDia	0.892251	11.4938	3.68726	5.00E-05	0.0249845
RNA-Seq	Trinity	XLOC_020980	c25583_g1_i1:0-645	cdRNA04-Dia-R3	cdRNA05-postDia	7.90714	39.4931	2.32037	5.00E-05	0.0249845
RNA-Seq	Trinity	XLOC_022000	c26707_g1_i1:0-703	cdRNA01-preDia	cdRNA05-postDia	2.53392	15.894	2.64904	0.0001	0.0432976
RNA-Seq	Trinity	XLOC_022085	c26785_g1_i1:0-410	cdRNA03-Dia-R2	cdRNA05-postDia	431.352	37.3317	-3.53039	5.00E-05	0.0249845
RNA-Seq	Trinity	XLOC_022085	c26785_g1_i1:0-410	cdRNA04-Dia-R3	cdRNA05-postDia	419.786	37.3317	-3.49118	5.00E-05	0.0249845
RNA-Seq	Trinity	XLOC_022085	c26785_g1_i1:0-410	cdRNA01-preDia	cdRNA04-Dia-R3	67.6954	419.786	2.63253	5.00E-05	0.0249845
RNA-Seq	Trinity	XLOC_022085	c26785_g1_i1:0-410	cdRNA01-preDia	cdRNA03-Dia-R2	67.6954	431.352	2.67174	5.00E-05	0.0249845
RNA-Seq	Trinity	XLOC_022207	c26899_g1_i1:0-843	cdRNA04-Dia-R3	cdRNA05-postDia	2.50669	18.5989	2.89136	5.00E-05	0.0249845
RNA-Seq	Trinity	XLOC_022207	c26899_g1_i1:0-843	cdRNA03-Dia-R2	cdRNA05-postDia	2.2461	18.5989	3.04972	5.00E-05	0.0249845
RNA-Seq	Trinity	XLOC_022246	c26932_g1_i1:0-1239	cdRNA02-Dia-R1	cdRNA05-postDia	9.28708	57.8767	2.63969	5.00E-05	0.0249845
RNA-Seq	Trinity	XLOC_022246	c26932_g1_i1:0-1239	cdRNA03-Dia-R2	cdRNA05-postDia	7.31806	57.8767	2.98345	5.00E-05	0.0249845
RNA-Seq	Trinity	XLOC_022246	c26932_g1_i1:0-1239	cdRNA04-Dia-R3	cdRNA05-postDia	6.4454	57.8767	3.16664	5.00E-05	0.0249845
RNA-Seq	Trinity	XLOC_022249	c26935_g1_i1:0-1129	cdRNA02-Dia-R1	cdRNA05-postDia	12.7224	108.673	3.09455	5.00E-05	0.0249845
RNA-Seq	Trinity	XLOC_022249	c26935_g1_i1:0-1129	cdRNA04-Dia-R3	cdRNA05-postDia	12.5039	108.673	3.11955	5.00E-05	0.0249845
RNA-Seq	Trinity	XLOC_022249	c26935_g1_i1:0-1129	cdRNA03-Dia-R2	cdRNA05-postDia	11.5641	108.673	3.23228	5.00E-05	0.0249845
RNA-Seq	Trinity	XLOC_022249	c26935_g1_i1:0-1129	cdRNA01-preDia	cdRNA05-postDia	3.84243	108.673	4.82184	5.00E-05	0.0249845
RNA-Seq	Trinity	XLOC_022258	c26943_g1_i1:16-620	cdRNA01-preDia	cdRNA04-Dia-R3	116.267	9.57041	-3.60272	5.00E-05	0.0249845
RNA-Seq	Trinity	XLOC_022258	c26943_g1_i1:16-620	cdRNA01-preDia	cdRNA03-Dia-R2	116.267	10.8482	-3.42191	5.00E-05	0.0249845
RNA-Seq	Trinity	XLOC_022258	c26943_g1_i1:16-620	cdRNA01-preDia	cdRNA02-Dia-R1	116.267	16.3671	-2.82858	5.00E-05	0.0249845
RNA-Seq	Trinity	XLOC_022258	c26943_g1_i1:16-620	cdRNA02-Dia-R1	cdRNA05-postDia	16.3671	128.232	2.96988	5.00E-05	0.0249845
RNA-Seq	Trinity	XLOC_022258	c26943_g1_i1:16-620	cdRNA03-Dia-R2	cdRNA05-postDia	10.8482	128.232	3.56322	5.00E-05	0.0249845
RNA-Seq	Trinity	XLOC_022258	c26943_g1_i1:16-620	cdRNA04-Dia-R3	cdRNA05-postDia	9.57041	128.232	3.74403	5.00E-05	0.0249845
RNA-Seq	Trinity	XLOC_022347	c27023_g1_i1:0-1567	cdRNA01-preDia	cdRNA03-Dia-R2	8.7841	1.24454	-2.81928	5.00E-05	0.0249845
RNA-Seq	Trinity	XLOC_022347	c27023_g1_i1:0-1567	cdRNA01-preDia	cdRNA04-Dia-R3	8.7841	1.50168	-2.54832	0.0001	0.0432976
RNA-Seq	Trinity	XLOC_022347	c27023_g1_i1:0-1567	cdRNA04-Dia-R3	cdRNA05-postDia	1.50168	13.4801	3.16618	5.00E-05	0.0249845
RNA-Seq	Trinity	XLOC_022347	c27023_g1_i1:0-1567	cdRNA03-Dia-R2	cdRNA05-postDia	1.24454	13.4801	3.43714	5.00E-05	0.0249845
RNA-Seq	Trinity	XLOC_022454	c27120_g1_i1:0-710	cdRNA04-Dia-R3	cdRNA05-postDia	8.01503	51.7481	2.69073	5.00E-05	0.0249845

RNA-Seq	Trinity	XLOC_022454	c27120_g1_i1:0-710	cdRNA03-Dia-R2	cdRNA05-postDia	7.48429	51.7481	2.78957	5.00E-05	0.0249845
RNA-Seq	Trinity	XLOC_022639	c27290_g1_i1:0-680	cdRNA01-preDia	cdRNA02-Dia-R1	72.6346	1.37539	-5.72274	5.00E-05	0.0249845
RNA-Seq	Trinity	XLOC_022639	c27290_g1_i1:0-680	cdRNA01-preDia	cdRNA04-Dia-R3	72.6346	4.77766	-3.92628	5.00E-05	0.0249845
RNA-Seq	Trinity	XLOC_022639	c27290_g1_i1:0-680	cdRNA01-preDia	cdRNA03-Dia-R2	72.6346	4.97248	-3.86862	0.0001	0.0432976
RNA-Seq	Trinity	XLOC_022662	c27311_g1_i1:0-2026	cdRNA03-Dia-R2	cdRNA05-postDia	24.358	2.99682	-3.02289	5.00E-05	0.0249845
RNA-Seq	Trinity	XLOC_022662	c27311_g1_i1:0-2026	cdRNA04-Dia-R3	cdRNA05-postDia	23.1332	2.99682	-2.94847	5.00E-05	0.0249845
RNA-Seq	Trinity	XLOC_022765	c27406_g1_i1:0-1481	cdRNA01-preDia	cdRNA03-Dia-R2	3.74408	20.6667	2.46463	5.00E-05	0.0249845
RNA-Seq	Trinity	XLOC_022765	c27406_g1_i1:0-1481	cdRNA01-preDia	cdRNA04-Dia-R3	3.74408	21.9347	2.55054	5.00E-05	0.0249845
RNA-Seq	Trinity	XLOC_022799	c27438_g1_i1:6-370	cdRNA04-Dia-R3	cdRNA05-postDia	31.179	154.485	2.30882	5.00E-05	0.0249845
RNA-Seq	Trinity	XLOC_022799	c27438_g1_i1:6-370	cdRNA03-Dia-R2	cdRNA05-postDia	29.0087	154.485	2.41291	5.00E-05	0.0249845
RNA-Seq	Trinity	XLOC_022799	c27438_g1_i1:6-370	cdRNA02-Dia-R1	cdRNA05-postDia	21.3268	154.485	2.85673	5.00E-05	0.0249845
RNA-Seq	Trinity	XLOC_022910	c27543_g1_i1:0-1988	cdRNA03-Dia-R2	cdRNA05-postDia	10.1055	1.74256	-2.53587	5.00E-05	0.0249845
RNA-Seq	Trinity	XLOC_022910	c27543_g1_i1:0-1988	cdRNA04-Dia-R3	cdRNA05-postDia	9.53963	1.74256	-2.45273	5.00E-05	0.0249845
RNA-Seq	Trinity	XLOC_022951	c2757_g1_i1:0-480	cdRNA03-Dia-R2	cdRNA05-postDia	7.43215	46.3518	2.64077	5.00E-05	0.0249845
RNA-Seq	Trinity	XLOC_022951	c2757_g1_i1:0-480	cdRNA04-Dia-R3	cdRNA05-postDia	7.00364	46.3518	2.72645	5.00E-05	0.0249845
RNA-Seq	Trinity	XLOC_022951	c2757_g1_i1:0-480	cdRNA01-preDia	cdRNA05-postDia	5.58328	46.3518	3.05344	5.00E-05	0.0249845
RNA-Seq	Trinity	XLOC_022970	c27597_g1_i1:0-756	cdRNA01-preDia	cdRNA05-postDia	1.64639	14.5123	3.1399	0.0001	0.0432976
RNA-Seq	Trinity	XLOC_023143	c27758_g1_i1:1-421	cdRNA01-preDia	cdRNA03-Dia-R2	33.2904	222.424	2.74013	5.00E-05	0.0249845
RNA-Seq	Trinity	XLOC_023143	c27758_g1_i1:1-421	cdRNA01-preDia	cdRNA04-Dia-R3	33.2904	224.939	2.75635	5.00E-05	0.0249845
RNA-Seq	Trinity	XLOC_023173	c27786_g1_i1:0-1352	cdRNA03-Dia-R2	cdRNA05-postDia	83.8806	15.6574	-2.42149	5.00E-05	0.0249845
RNA-Seq	Trinity	XLOC_023173	c27786_g1_i1:0-1352	cdRNA02-Dia-R1	cdRNA04-Dia-R3	15.8386	81.0895	2.35607	5.00E-05	0.0249845
RNA-Seq	Trinity	XLOC_023173	c27786_g1_i1:0-1352	cdRNA02-Dia-R1	cdRNA03-Dia-R2	15.8386	83.8806	2.40489	0.0001	0.0432976
RNA-Seq	Trinity	XLOC_023173	c27786_g1_i1:0-1352	cdRNA01-preDia	cdRNA04-Dia-R3	9.08681	81.0895	3.15767	5.00E-05	0.0249845
RNA-Seq	Trinity	XLOC_023173	c27786_g1_i1:0-1352	cdRNA01-preDia	cdRNA03-Dia-R2	9.08681	83.8806	3.20649	5.00E-05	0.0249845
RNA-Seq	Trinity	XLOC_023195	c27806_g1_i1:0-279	cdRNA01-preDia	cdRNA03-Dia-R2	108.428	745.186	2.78086	5.00E-05	0.0249845
RNA-Seq	Trinity	XLOC_023195	c27806_g1_i1:0-279	cdRNA01-preDia	cdRNA04-Dia-R3	108.428	782.853	2.852	5.00E-05	0.0249845
RNA-Seq	Trinity	XLOC_023195	c27806_g1_i1:0-279	cdRNA01-preDia	cdRNA05-postDia	108.428	1227.92	3.5014	5.00E-05	0.0249845
RNA-Seq	Trinity	XLOC_023280	c27886_g1_i1:0-3254	cdRNA03-Dia-R2	cdRNA05-postDia	40.9238	5.78886	-2.82159	5.00E-05	0.0249845
RNA-Seq	Trinity	XLOC_023280	c27886_g1_i1:0-3254	cdRNA04-Dia-R3	cdRNA05-postDia	40.936	5.78886	-2.82202	5.00E-05	0.0249845
RNA-Seq	Trinity	XLOC_023297	c27900_g1_i1:0-1418	cdRNA02-Dia-R1	cdRNA05-postDia	8.28544	45.7318	2.46455	5.00E-05	0.0249845
RNA-Seq	Trinity	XLOC_023548	c28129_g1_i1:0-873	cdRNA04-Dia-R3	cdRNA05-postDia	30.8211	5.83825	-2.40031	5.00E-05	0.0249845
RNA-Seq	Trinity	XLOC_023635	c2820_g1_i1:0-1169	cdRNA04-Dia-R3	cdRNA05-postDia	137.512	21.2699	-2.69268	0.0001	0.0432976
RNA-Seq	Trinity	XLOC_023635	c2820_g1_i1:0-1169	cdRNA03-Dia-R2	cdRNA05-postDia	127.422	21.2699	-2.58273	0.0001	0.0432976
RNA-Seq	Trinity	XLOC_023659	c28231_g1_i1:0-1014	cdRNA03-Dia-R2	cdRNA05-postDia	2.62992	20.4196	2.95686	5.00E-05	0.0249845
RNA-Seq	Trinity	XLOC_023827	c28387_g1_i1:0-2540	cdRNA01-preDia	cdRNA05-postDia	2.28356	21.1903	3.21405	5.00E-05	0.0249845
RNA-Seq	Trinity	XLOC_023851	c28409_g1_i1:0-1761	cdRNA01-preDia	cdRNA03-Dia-R2	10.6589	1.84957	-2.52679	5.00E-05	0.0249845
RNA-Seq	Trinity	XLOC_023851	c28409_g1_i1:0-1761	cdRNA03-Dia-R2	cdRNA05-postDia	1.84957	13.3619	2.85286	5.00E-05	0.0249845
RNA-Seq	Trinity	XLOC_023851	c28409_g1_i1:0-1761	cdRNA04-Dia-R3	cdRNA05-postDia	1.63104	13.3619	3.03426	0.0001	0.0432976
RNA-Seq	Trinity	XLOC_023940	c28490_g1_i1:0-1075	cdRNA01-preDia	cdRNA03-Dia-R2	75.9426	12.8175	-2.5668	5.00E-05	0.0249845
RNA-Seq	Trinity	XLOC_023940	c28490_g1_i1:0-1075	cdRNA01-preDia	cdRNA04-Dia-R3	75.9426	13.7949	-2.46078	5.00E-05	0.0249845
RNA-Seq	Trinity	XLOC_024008	c28554_g1_i1:0-1379	cdRNA01-preDia	cdRNA05-postDia	5.4815	31.9521	2.54327	0.0001	0.0432976
RNA-Seq	Trinity	XLOC_024089	c28630_g1_i1:0-1464	cdRNA04-Dia-R3	cdRNA05-postDia	29.9317	3.9539	-2.92032	5.00E-05	0.0249845
RNA-Seq	Trinity	XLOC_024089	c28630_g1_i1:0-1464	cdRNA03-Dia-R2	cdRNA05-postDia	28.7101	3.9539	-2.86021	5.00E-05	0.0249845
RNA-Seq	Trinity	XLOC_024162	c2869_g1_i1:1-224	cdRNA04-Dia-R3	cdRNA05-postDia	49.3838	247.877	2.32752	5.00E-05	0.0249845
RNA-Seq	Trinity	XLOC_024162	c2869_g1_i1:1-224	cdRNA03-Dia-R2	cdRNA05-postDia	44.6092	247.877	2.47421	5.00E-05	0.0249845
RNA-Seq	Trinity	XLOC_024162	c2869_g1_i1:1-224	cdRNA01-preDia	cdRNA05-postDia	28.1694	247.877	3.13742	5.00E-05	0.0249845
RNA-Seq	Trinity	XLOC_024450	c28969_g1_i1:0-464	cdRNA01-preDia	cdRNA05-postDia	8.35838	47.5179	2.50718	5.00E-05	0.0249845

RNA-Seq	Trinity	XLOC_024500	c29015_g1_i1:0-1099	cdRNA03-Dia-R2	cdRNA05-postDia	64.4405	415.206	2.68779	5.00E-05	0.0249845
RNA-Seq	Trinity	XLOC_024500	c29015_g1_i1:0-1099	cdRNA04-Dia-R3	cdRNA05-postDia	64.1603	415.206	2.69407	5.00E-05	0.0249845
RNA-Seq	Trinity	XLOC_024556	c2906_g1_i1:0-1536	cdRNA03-Dia-R2	cdRNA05-postDia	1.33274	8.16855	2.61569	5.00E-05	0.0249845
RNA-Seq	Trinity	XLOC_024556	c2906_g1_i1:0-1536	cdRNA04-Dia-R3	cdRNA05-postDia	1.08133	8.16855	2.91727	5.00E-05	0.0249845
RNA-Seq	Trinity	XLOC_024575	c29089_g1_i1:0-1230	cdRNA01-preDia	cdRNA05-postDia	3.00397	20.5103	2.7714	5.00E-05	0.0249845
RNA-Seq	Trinity	XLOC_024629	c29139_g1_i1:0-414	cdRNA01-preDia	cdRNA05-postDia	17.6841	105.124	2.57157	5.00E-05	0.0249845
RNA-Seq	Trinity	XLOC_024629	c29139_g1_i1:0-414	cdRNA02-Dia-R1	cdRNA05-postDia	16.2938	105.124	2.6897	5.00E-05	0.0249845
RNA-Seq	Trinity	XLOC_024713	c29218_g1_i1:0-2349	cdRNA01-preDia	cdRNA03-Dia-R2	173.438	13.3336	-3.70128	5.00E-05	0.0249845
RNA-Seq	Trinity	XLOC_024713	c29218_g1_i1:0-2349	cdRNA01-preDia	cdRNA04-Dia-R3	173.438	13.5534	-3.67769	5.00E-05	0.0249845
RNA-Seq	Trinity	XLOC_024883	c29378_g1_i1:0-1995	cdRNA01-preDia	cdRNA03-Dia-R2	11.2519	74.427	2.72566	5.00E-05	0.0249845
RNA-Seq	Trinity	XLOC_024883	c29378_g1_i1:0-1995	cdRNA01-preDia	cdRNA04-Dia-R3	11.2519	78.5795	2.80398	5.00E-05	0.0249845
RNA-Seq	Trinity	XLOC_024919	c29410_g1_i1:0-2140	cdRNA04-Dia-R3	cdRNA05-postDia	239.834	26.4742	-3.17938	5.00E-05	0.0249845
RNA-Seq	Trinity	XLOC_024919	c29410_g1_i1:0-2140	cdRNA03-Dia-R2	cdRNA05-postDia	239.12	26.4742	-3.17507	5.00E-05	0.0249845
RNA-Seq	Trinity	XLOC_024986	c29474_g1_i1:0-1683	cdRNA03-Dia-R2	cdRNA05-postDia	18.0575	152.873	3.08167	5.00E-05	0.0249845
RNA-Seq	Trinity	XLOC_024986	c29474_g1_i1:0-1683	cdRNA04-Dia-R3	cdRNA05-postDia	17.0681	152.873	3.16296	5.00E-05	0.0249845
RNA-Seq	Trinity	XLOC_024988	c29476_g1_i1:0-1140	cdRNA01-preDia	cdRNA04-Dia-R3	34.1946	186.763	2.44937	0.0001	0.0432976
RNA-Seq	Trinity	XLOC_024997	c29484_g1_i1:0-717	cdRNA02-Dia-R1	cdRNA05-postDia	6.26495	32.0081	2.35306	0.0001	0.0432976
RNA-Seq	Trinity	XLOC_024997	c29484_g1_i1:0-717	cdRNA03-Dia-R2	cdRNA05-postDia	5.84421	32.0081	2.45336	0.0001	0.0432976
RNA-Seq	Trinity	XLOC_024997	c29484_g1_i1:0-717	cdRNA04-Dia-R3	cdRNA05-postDia	5.3197	32.0081	2.58902	0.0001	0.0432976
RNA-Seq	Trinity	XLOC_025325	c29788_g2_i1:0-1158	cdRNA04-Dia-R3	cdRNA05-postDia	13.9853	89.6951	2.68112	5.00E-05	0.0249845
RNA-Seq	Trinity	XLOC_025325	c29788_g2_i1:0-1158	cdRNA02-Dia-R1	cdRNA05-postDia	13.5693	89.6951	2.72468	5.00E-05	0.0249845
RNA-Seq	Trinity	XLOC_025325	c29788_g2_i1:0-1158	cdRNA03-Dia-R2	cdRNA05-postDia	12.2399	89.6951	2.87344	5.00E-05	0.0249845
RNA-Seq	Trinity	XLOC_025503	c29955_g1_i1:0-823	cdRNA01-preDia	cdRNA05-postDia	6.1281	48.0963	2.97241	5.00E-05	0.0249845
RNA-Seq	Trinity	XLOC_025555	c30001_g1_i1:0-800	cdRNA04-Dia-R3	cdRNA05-postDia	9.92092	56.84	2.51836	0.0001	0.0432976
RNA-Seq	Trinity	XLOC_025555	c30001_g1_i1:0-800	cdRNA01-preDia	cdRNA05-postDia	4.17676	56.84	3.76645	5.00E-05	0.0249845
RNA-Seq	Trinity	XLOC_025725	c30166_g1_i1:0-910	cdRNA02-Dia-R1	cdRNA05-postDia	4.63663	43.2421	3.22129	5.00E-05	0.0249845
RNA-Seq	Trinity	XLOC_025725	c30166_g1_i1:0-910	cdRNA03-Dia-R2	cdRNA05-postDia	4.35836	43.2421	3.31058	5.00E-05	0.0249845
RNA-Seq	Trinity	XLOC_025725	c30166_g1_i1:0-910	cdRNA04-Dia-R3	cdRNA05-postDia	3.16754	43.2421	3.771	5.00E-05	0.0249845
RNA-Seq	Trinity	XLOC_025920	c30351_g1_i1:0-1373	cdRNA01-preDia	cdRNA04-Dia-R3	6.01666	29.7002	2.30344	0.0001	0.0432976
RNA-Seq	Trinity	XLOC_025920	c30351_g1_i1:0-1373	cdRNA01-preDia	cdRNA02-Dia-R1	6.01666	47.4005	2.97787	5.00E-05	0.0249845
RNA-Seq	Trinity	XLOC_025929	c3035_g1_i1:0-1353	cdRNA03-Dia-R2	cdRNA05-postDia	8.78933	1.21404	-2.85593	0.0001	0.0432976
RNA-Seq	Trinity	XLOC_025944	c30375_g1_i1:3-339	cdRNA01-preDia	cdRNA05-postDia	13.0427	103.449	2.98761	5.00E-05	0.0249845
RNA-Seq	Trinity	XLOC_026005	c30433_g1_i1:0-917	cdRNA04-Dia-R3	cdRNA05-postDia	14.7785	79.1511	2.42111	5.00E-05	0.0249845
RNA-Seq	Trinity	XLOC_026005	c30433_g1_i1:0-917	cdRNA03-Dia-R2	cdRNA05-postDia	13.8319	79.1511	2.5166	5.00E-05	0.0249845
RNA-Seq	Trinity	XLOC_026016	c30446_g1_i1:0-1935	cdRNA01-preDia	cdRNA03-Dia-R2	12.6235	74.1352	2.55405	0.0001	0.0432976
RNA-Seq	Trinity	XLOC_026016	c30446_g1_i1:0-1935	cdRNA01-preDia	cdRNA04-Dia-R3	12.6235	76.7487	2.60403	5.00E-05	0.0249845
RNA-Seq	Trinity	XLOC_026153	c30572_g1_i1:0-1265	cdRNA03-Dia-R2	cdRNA05-postDia	2.97447	23.1393	2.95964	5.00E-05	0.0249845
RNA-Seq	Trinity	XLOC_026153	c30572_g1_i1:0-1265	cdRNA04-Dia-R3	cdRNA05-postDia	2.00055	23.1393	3.53188	5.00E-05	0.0249845
RNA-Seq	Trinity	XLOC_026320	c30734_g1_i1:3-1177	cdRNA02-Dia-R1	cdRNA05-postDia	5.30751	31.0213	2.54715	5.00E-05	0.0249845
RNA-Seq	Trinity	XLOC_026320	c30734_g1_i1:3-1177	cdRNA04-Dia-R3	cdRNA05-postDia	3.00882	31.0213	3.36599	5.00E-05	0.0249845
RNA-Seq	Trinity	XLOC_026320	c30734_g1_i1:3-1177	cdRNA03-Dia-R2	cdRNA05-postDia	2.39947	31.0213	3.69247	5.00E-05	0.0249845
RNA-Seq	Trinity	XLOC_026418	c30832_g1_i1:0-886	cdRNA01-preDia	cdRNA05-postDia	18.3663	3.38943	-2.43795	0.0001	0.0432976
RNA-Seq	Trinity	XLOC_026672	c31068_g1_i1:0-1200	cdRNA03-Dia-R2	cdRNA05-postDia	5.58464	30.5317	2.45077	5.00E-05	0.0249845
RNA-Seq	Trinity	XLOC_026672	c31068_g1_i1:0-1200	cdRNA01-preDia	cdRNA05-postDia	1.90428	30.5317	4.00299	5.00E-05	0.0249845
RNA-Seq	Trinity	XLOC_026811	c311_g1_i1:0-2010	cdRNA03-Dia-R2	cdRNA05-postDia	5.98509	36.8321	2.62152	5.00E-05	0.0249845
RNA-Seq	Trinity	XLOC_026811	c311_g1_i1:0-2010	cdRNA04-Dia-R3	cdRNA05-postDia	5.5827	36.8321	2.72193	5.00E-05	0.0249845
RNA-Seq	Trinity	XLOC_026867	c31251_g1_i1:0-974	cdRNA04-Dia-R3	cdRNA05-postDia	80.3843	10.6108	-2.92139	5.00E-05	0.0249845

RNA-Seq	Trinity	XLOC_026867	c31251_g1_i1:0-974	cdRNA03-Dia-R2	cdRNA05-postDia	68.4462	10.6108	-2.68944	5.00E-05	0.0249845
RNA-Seq	Trinity	XLOC_027293	c31654_g1_i1:0-1267	cdRNA04-Dia-R3	cdRNA05-postDia	5.70512	33.8938	2.57069	5.00E-05	0.0249845
RNA-Seq	Trinity	XLOC_027303	c31663_g1_i1:0-302	cdRNA01-preDia	cdRNA05-postDia	23.7127	159.974	2.7541	5.00E-05	0.0249845
RNA-Seq	Trinity	XLOC_027303	c31663_g1_i1:0-302	cdRNA04-Dia-R3	cdRNA05-postDia	14.9156	159.974	3.42294	5.00E-05	0.0249845
RNA-Seq	Trinity	XLOC_027303	c31663_g1_i1:0-302	cdRNA03-Dia-R2	cdRNA05-postDia	10.8667	159.974	3.87985	5.00E-05	0.0249845
RNA-Seq	Trinity	XLOC_027303	c31663_g1_i1:0-302	cdRNA02-Dia-R1	cdRNA05-postDia	8.49019	159.974	4.23589	5.00E-05	0.0249845
RNA-Seq	Trinity	XLOC_027486	c3182_g1_i1:0-560	cdRNA03-Dia-R2	cdRNA05-postDia	28.9211	3.91826	-2.88384	5.00E-05	0.0249845
RNA-Seq	Trinity	XLOC_027486	c3182_g1_i1:0-560	cdRNA04-Dia-R3	cdRNA05-postDia	26.8298	3.91826	-2.77555	0.0001	0.0432976
RNA-Seq	Trinity	XLOC_027578	c31915_g1_i1:0-219	cdRNA03-Dia-R2	cdRNA05-postDia	238.004	30.1871	-2.97899	5.00E-05	0.0249845
RNA-Seq	Trinity	XLOC_027578	c31915_g1_i1:0-219	cdRNA04-Dia-R3	cdRNA05-postDia	230.659	30.1871	-2.93376	5.00E-05	0.0249845
RNA-Seq	Trinity	XLOC_028006	c32319_g1_i1:1-781	cdRNA01-preDia	cdRNA05-postDia	6.99717	69.1232	3.30433	5.00E-05	0.0249845
RNA-Seq	Trinity	XLOC_028035	c32347_g1_i1:0-851	cdRNA01-preDia	cdRNA03-Dia-R2	26.322	199.133	2.91939	0.0001	0.0432976
RNA-Seq	Trinity	XLOC_028035	c32347_g1_i1:0-851	cdRNA01-preDia	cdRNA04-Dia-R3	26.322	202.697	2.94498	5.00E-05	0.0249845
RNA-Seq	Trinity	XLOC_028171	c32478_g1_i1:2-555	cdRNA03-Dia-R2	cdRNA05-postDia	23.7366	118.649	2.32152	0.0001	0.0432976
RNA-Seq	Trinity	XLOC_028171	c32478_g1_i1:2-555	cdRNA04-Dia-R3	cdRNA05-postDia	22.5894	118.649	2.39298	5.00E-05	0.0249845
RNA-Seq	Trinity	XLOC_028360	c3265_g1_i1:0-1109	cdRNA01-preDia	cdRNA03-Dia-R2	17.7266	1.96155	-3.17585	5.00E-05	0.0249845
RNA-Seq	Trinity	XLOC_028360	c3265_g1_i1:0-1109	cdRNA01-preDia	cdRNA04-Dia-R3	17.7266	2.13599	-3.05294	5.00E-05	0.0249845
RNA-Seq	Trinity	XLOC_028750	c33026_g1_i1:0-1284	cdRNA02-Dia-R1	cdRNA05-postDia	3.77497	20.4074	2.43455	5.00E-05	0.0249845
RNA-Seq	Trinity	XLOC_028773	c33047_g1_i1:9-765	cdRNA01-preDia	cdRNA05-postDia	2.4338	16.8228	2.78914	0.0001	0.0432976
RNA-Seq	Trinity	XLOC_028773	c33047_g1_i1:9-765	cdRNA01-preDia	cdRNA04-Dia-R3	2.4338	16.8915	2.79502	0.0001	0.0432976
RNA-Seq	Trinity	XLOC_028773	c33047_g1_i1:9-765	cdRNA01-preDia	cdRNA03-Dia-R2	2.4338	20.0816	3.04459	5.00E-05	0.0249845
RNA-Seq	Trinity	XLOC_028828	c3309_g1_i1:0-701	cdRNA04-Dia-R3	cdRNA05-postDia	2.97427	20.3684	2.77573	5.00E-05	0.0249845
RNA-Seq	Trinity	XLOC_028828	c3309_g1_i1:0-701	cdRNA03-Dia-R2	cdRNA05-postDia	2.61931	20.3684	2.95907	5.00E-05	0.0249845
RNA-Seq	Trinity	XLOC_028914	c33184_g1_i1:0-1446	cdRNA04-Dia-R3	cdRNA05-postDia	53.3151	9.72316	-2.45505	5.00E-05	0.0249845
RNA-Seq	Trinity	XLOC_029041	c33312_g1_i1:0-700	cdRNA03-Dia-R2	cdRNA05-postDia	9.3849	44.9169	2.25884	0.0001	0.0432976
RNA-Seq	Trinity	XLOC_029041	c33312_g1_i1:0-700	cdRNA04-Dia-R3	cdRNA05-postDia	8.71154	44.9169	2.36626	5.00E-05	0.0249845
RNA-Seq	Trinity	XLOC_029041	c33312_g1_i1:0-700	cdRNA02-Dia-R1	cdRNA05-postDia	7.19964	44.9169	2.64126	5.00E-05	0.0249845
RNA-Seq	Trinity	XLOC_029069	c33339_g1_i1:0-471	cdRNA01-preDia	cdRNA05-postDia	3.40369	34.0325	3.32174	5.00E-05	0.0249845
RNA-Seq	Trinity	XLOC_029130	c33398_g1_i1:2-570	cdRNA01-preDia	cdRNA05-postDia	12.4835	72.4823	2.53761	5.00E-05	0.0249845
RNA-Seq	Trinity	XLOC_029132	c3339_g1_i1:2-289	cdRNA04-Dia-R3	cdRNA05-postDia	160.734	16.6758	-3.26885	5.00E-05	0.0249845
RNA-Seq	Trinity	XLOC_029132	c3339_g1_i1:2-289	cdRNA03-Dia-R2	cdRNA05-postDia	148.998	16.6758	-3.15947	5.00E-05	0.0249845
RNA-Seq	Trinity	XLOC_029329	c33586_g1_i1:6-719	cdRNA01-preDia	cdRNA05-postDia	2.87228	19.575	2.76874	5.00E-05	0.0249845
RNA-Seq	Trinity	XLOC_029329	c33586_g1_i1:6-719	cdRNA04-Dia-R3	cdRNA05-postDia	2.45736	19.575	2.99383	0.0001	0.0432976
RNA-Seq	Trinity	XLOC_029329	c33586_g1_i1:6-719	cdRNA03-Dia-R2	cdRNA05-postDia	1.16253	19.575	4.07368	5.00E-05	0.0249845
RNA-Seq	Trinity	XLOC_029607	c33855_g1_i1:7-800	cdRNA01-preDia	cdRNA05-postDia	41.6602	5.95455	-2.80661	5.00E-05	0.0249845
RNA-Seq	Trinity	XLOC_029607	c33855_g1_i1:7-800	cdRNA01-preDia	cdRNA03-Dia-R2	41.6602	7.30033	-2.51264	5.00E-05	0.0249845
RNA-Seq	Trinity	XLOC_030016	c34238_g1_i1:0-768	cdRNA01-preDia	cdRNA04-Dia-R3	12.0502	60.7523	2.33388	0.0001	0.0432976
RNA-Seq	Trinity	XLOC_030083	c3429_g1_i1:0-871	cdRNA02-Dia-R1	cdRNA05-postDia	22.2186	3.46565	-2.68057	5.00E-05	0.0249845
RNA-Seq	Trinity	XLOC_030100	c34315_g1_i1:0-472	cdRNA03-Dia-R2	cdRNA05-postDia	89.4929	15.1682	-2.56072	5.00E-05	0.0249845
RNA-Seq	Trinity	XLOC_030100	c34315_g1_i1:0-472	cdRNA04-Dia-R3	cdRNA05-postDia	86.5523	15.1682	-2.51252	5.00E-05	0.0249845
RNA-Seq	Trinity	XLOC_030100	c34315_g1_i1:0-472	cdRNA01-preDia	cdRNA04-Dia-R3	17.3973	86.5523	2.31471	0.0001	0.0432976
RNA-Seq	Trinity	XLOC_030363	c34567_g1_i1:0-922	cdRNA01-preDia	cdRNA05-postDia	6.82408	54.6203	3.00073	5.00E-05	0.0249845
RNA-Seq	Trinity	XLOC_030411	c34612_g1_i1:0-532	cdRNA04-Dia-R3	cdRNA05-postDia	109.547	24.5443	-2.15809	0.0001	0.0432976
RNA-Seq	Trinity	XLOC_030411	c34612_g1_i1:0-532	cdRNA01-preDia	cdRNA04-Dia-R3	20.1178	109.547	2.44501	0.0001	0.0432976
RNA-Seq	Trinity	XLOC_030588	c34789_g1_i1:0-266	cdRNA04-Dia-R3	cdRNA05-postDia	51.7714	318.207	2.61974	5.00E-05	0.0249845
RNA-Seq	Trinity	XLOC_030588	c34789_g1_i1:0-266	cdRNA03-Dia-R2	cdRNA05-postDia	39.4398	318.207	3.01224	5.00E-05	0.0249845
RNA-Seq	Trinity	XLOC_030972	c35170_g1_i1:0-681	cdRNA03-Dia-R2	cdRNA05-postDia	3.96965	23.3948	2.5591	5.00E-05	0.0249845

RNA-Seq	Trinity	XLOC_031087	c35279_g1_i1:2-772	cdRNA03-Dia-R2	cdRNA05-postDia	13.7303	60.6976	2.14428	0.0001	0.0432976
RNA-Seq	Trinity	XLOC_031087	c35279_g1_i1:2-772	cdRNA02-Dia-R1	cdRNA05-postDia	11.4618	60.6976	2.40481	5.00E-05	0.0249845
RNA-Seq	Trinity	XLOC_031315	c35496_g1_i1:0-1663	cdRNA01-preDia	cdRNA05-postDia	9.47902	1.17463	-3.01253	5.00E-05	0.0249845
RNA-Seq	Trinity	XLOC_031315	c35496_g1_i1:0-1663	cdRNA02-Dia-R1	cdRNA05-postDia	8.79104	1.17463	-2.90383	5.00E-05	0.0249845
RNA-Seq	Trinity	XLOC_031507	c35681_g1_i1:0-692	cdRNA02-Dia-R1	cdRNA05-postDia	18.5991	2.77676	-2.74375	5.00E-05	0.0249845
RNA-Seq	Trinity	XLOC_031531	c35703_g1_i1:0-934	cdRNA01-preDia	cdRNA04-Dia-R3	41.9494	3.27109	-3.6808	5.00E-05	0.0249845
RNA-Seq	Trinity	XLOC_031531	c35703_g1_i1:0-934	cdRNA01-preDia	cdRNA03-Dia-R2	41.9494	3.62064	-3.53433	5.00E-05	0.0249845
RNA-Seq	Trinity	XLOC_031531	c35703_g1_i1:0-934	cdRNA02-Dia-R1	cdRNA03-Dia-R2	21.9726	3.62064	-2.60139	5.00E-05	0.0249845
RNA-Seq	Trinity	XLOC_031584	c35759_g1_i1:0-361	cdRNA01-preDia	cdRNA05-postDia	11.2877	72.3594	2.68043	5.00E-05	0.0249845
RNA-Seq	Trinity	XLOC_031627	c357_g1_i1:0-501	cdRNA02-Dia-R1	cdRNA05-postDia	36.972	298.033	3.01096	5.00E-05	0.0249845
RNA-Seq	Trinity	XLOC_031627	c357_g1_i1:0-501	cdRNA03-Dia-R2	cdRNA05-postDia	27.8923	298.033	3.41753	5.00E-05	0.0249845
RNA-Seq	Trinity	XLOC_031627	c357_g1_i1:0-501	cdRNA04-Dia-R3	cdRNA05-postDia	26.6719	298.033	3.48208	5.00E-05	0.0249845
RNA-Seq	Trinity	XLOC_031627	c357_g1_i1:0-501	cdRNA01-preDia	cdRNA05-postDia	25.2774	298.033	3.55955	5.00E-05	0.0249845
RNA-Seq	Trinity	XLOC_031868	c36033_g1_i1:1-465	cdRNA04-Dia-R3	cdRNA05-postDia	24.3447	139.948	2.52321	5.00E-05	0.0249845
RNA-Seq	Trinity	XLOC_031868	c36033_g1_i1:1-465	cdRNA03-Dia-R2	cdRNA05-postDia	23.8202	139.948	2.55463	0.0001	0.0432976
RNA-Seq	Trinity	XLOC_031868	c36033_g1_i1:1-465	cdRNA02-Dia-R1	cdRNA05-postDia	22.8906	139.948	2.61206	5.00E-05	0.0249845
RNA-Seq	Trinity	XLOC_031868	c36033_g1_i1:1-465	cdRNA01-preDia	cdRNA05-postDia	18.9963	139.948	2.8811	5.00E-05	0.0249845
RNA-Seq	Trinity	XLOC_032816	c369_g1_i1:0-1207	cdRNA01-preDia	cdRNA05-postDia	13.8279	91.4723	2.72576	5.00E-05	0.0249845
RNA-Seq	Trinity	XLOC_033129	c37307_g1_i1:0-819	cdRNA01-preDia	cdRNA05-postDia	46.7737	268.162	2.51934	5.00E-05	0.0249845
RNA-Seq	Trinity	XLOC_033129	c37307_g1_i1:0-819	cdRNA04-Dia-R3	cdRNA05-postDia	23.3581	268.162	3.52111	5.00E-05	0.0249845
RNA-Seq	Trinity	XLOC_033129	c37307_g1_i1:0-819	cdRNA03-Dia-R2	cdRNA05-postDia	22.8994	268.162	3.54972	5.00E-05	0.0249845
RNA-Seq	Trinity	XLOC_033129	c37307_g1_i1:0-819	cdRNA02-Dia-R1	cdRNA05-postDia	19.5488	268.162	3.77795	5.00E-05	0.0249845
RNA-Seq	Trinity	XLOC_033437	c37615_g1_i1:0-643	cdRNA03-Dia-R2	cdRNA05-postDia	4.04367	22.1515	2.45367	0.0001	0.0432976
RNA-Seq	Trinity	XLOC_033737	c37912_g1_i1:0-469	cdRNA03-Dia-R2	cdRNA05-postDia	4.16804	31.883	2.93535	5.00E-05	0.0249845
RNA-Seq	Trinity	XLOC_033737	c37912_g1_i1:0-469	cdRNA04-Dia-R3	cdRNA05-postDia	4.00474	31.883	2.99301	0.0001	0.0432976
RNA-Seq	Trinity	XLOC_033784	c37956_g1_i1:0-221	cdRNA01-preDia	cdRNA03-Dia-R2	537.548	96.9395	-2.47124	5.00E-05	0.0249845
RNA-Seq	Trinity	XLOC_033784	c37956_g1_i1:0-221	cdRNA04-Dia-R3	cdRNA05-postDia	128.67	708.788	2.46168	5.00E-05	0.0249845
RNA-Seq	Trinity	XLOC_033784	c37956_g1_i1:0-221	cdRNA03-Dia-R2	cdRNA05-postDia	96.9395	708.788	2.8702	5.00E-05	0.0249845
RNA-Seq	Trinity	XLOC_034287	c3843_g1_i1:0-428	cdRNA03-Dia-R2	cdRNA05-postDia	14.4402	77.0367	2.41545	0.0001	0.0432976
RNA-Seq	Trinity	XLOC_034733	c38870_g1_i1:0-1055	cdRNA02-Dia-R1	cdRNA05-postDia	6.71409	37.373	2.47673	5.00E-05	0.0249845
RNA-Seq	Trinity	XLOC_035227	c3935_g1_i1:0-425	cdRNA03-Dia-R2	cdRNA05-postDia	8.74592	58.0651	2.73099	0.0001	0.0432976
RNA-Seq	Trinity	XLOC_035227	c3935_g1_i1:0-425	cdRNA04-Dia-R3	cdRNA05-postDia	7.71728	58.0651	2.91151	5.00E-05	0.0249845
RNA-Seq	Trinity	XLOC_035227	c3935_g1_i1:0-425	cdRNA01-preDia	cdRNA05-postDia	2.68172	58.0651	4.43644	5.00E-05	0.0249845
RNA-Seq	Trinity	XLOC_035568	c39695_g1_i1:0-265	cdRNA04-Dia-R3	cdRNA05-postDia	18.3514	136.21	2.89187	5.00E-05	0.0249845
RNA-Seq	Trinity	XLOC_035568	c39695_g1_i1:0-265	cdRNA02-Dia-R1	cdRNA05-postDia	12.2893	136.21	3.47036	5.00E-05	0.0249845
RNA-Seq	Trinity	XLOC_035568	c39695_g1_i1:0-265	cdRNA03-Dia-R2	cdRNA05-postDia	8.98811	136.21	3.92167	0.0001	0.0432976
RNA-Seq	Trinity	XLOC_035780	c39909_g1_i1:0-705	cdRNA01-preDia	cdRNA05-postDia	6.78272	35.0817	2.37078	0.0001	0.0432976
RNA-Seq	Trinity	XLOC_035780	c39909_g1_i1:0-705	cdRNA02-Dia-R1	cdRNA05-postDia	3.97968	35.0817	3.13999	5.00E-05	0.0249845
RNA-Seq	Trinity	XLOC_035941	c40061_g1_i1:0-443	cdRNA01-preDia	cdRNA05-postDia	31.4114	156.413	2.316	0.0001	0.0432976
RNA-Seq	Trinity	XLOC_035941	c40061_g1_i1:0-443	cdRNA02-Dia-R1	cdRNA05-postDia	23.6567	156.413	2.72504	5.00E-05	0.0249845
RNA-Seq	Trinity	XLOC_036519	c40664_g1_i1:0-720	cdRNA03-Dia-R2	cdRNA05-postDia	6.42184	39.0824	2.60546	5.00E-05	0.0249845
RNA-Seq	Trinity	XLOC_036519	c40664_g1_i1:0-720	cdRNA04-Dia-R3	cdRNA05-postDia	5.94987	39.0824	2.71559	5.00E-05	0.0249845
RNA-Seq	Trinity	XLOC_036519	c40664_g1_i1:0-720	cdRNA02-Dia-R1	cdRNA05-postDia	5.53647	39.0824	2.81948	5.00E-05	0.0249845
RNA-Seq	Trinity	XLOC_036732	c40895_g1_i1:2-588	cdRNA01-preDia	cdRNA03-Dia-R2	5.06842	44.4049	3.13111	5.00E-05	0.0249845
RNA-Seq	Trinity	XLOC_036732	c40895_g1_i1:2-588	cdRNA01-preDia	cdRNA04-Dia-R3	5.06842	45.8403	3.17701	5.00E-05	0.0249845
RNA-Seq	Trinity	XLOC_036944	c41115_g1_i1:0-474	cdRNA03-Dia-R2	cdRNA05-postDia	15.0485	83.2502	2.46783	5.00E-05	0.0249845
RNA-Seq	Trinity	XLOC_036944	c41115_g1_i1:0-474	cdRNA01-preDia	cdRNA05-postDia	5.26832	83.2502	3.98204	5.00E-05	0.0249845

RNA-Seq	Trinity	XLOC_037379	c41567_g1_i1:0-524	cdRNA04-Dia-R3	cdRNA05-postDia	5.09312	35.6196	2.80605	0.0001	0.0432976
RNA-Seq	Trinity	XLOC_037379	c41567_g1_i1:0-524	cdRNA03-Dia-R2	cdRNA05-postDia	4.68443	35.6196	2.92673	0.0001	0.0432976
RNA-Seq	Trinity	XLOC_037379	c41567_g1_i1:0-524	cdRNA02-Dia-R1	cdRNA05-postDia	4.46371	35.6196	2.99636	5.00E-05	0.0249845
RNA-Seq	Trinity	XLOC_037470	c41662_g1_i1:12-234	cdRNA01-preDia	cdRNA04-Dia-R3	19.7205	185.385	3.23275	5.00E-05	0.0249845
RNA-Seq	Trinity	XLOC_038360	c42648_g1_i1:0-522	cdRNA01-preDia	cdRNA04-Dia-R3	4.8459	31.8236	2.71526	5.00E-05	0.0249845
RNA-Seq	Trinity	XLOC_038360	c42648_g1_i1:0-522	cdRNA01-preDia	cdRNA05-postDia	4.8459	33.0864	2.7714	5.00E-05	0.0249845
RNA-Seq	Trinity	XLOC_038360	c42648_g1_i1:0-522	cdRNA01-preDia	cdRNA03-Dia-R2	4.8459	33.9896	2.81026	5.00E-05	0.0249845
RNA-Seq	Trinity	XLOC_038410	c42692_g1_i1:0-1792	cdRNA01-preDia	cdRNA04-Dia-R3	22.0213	180.376	3.03403	5.00E-05	0.0249845
RNA-Seq	Trinity	XLOC_038410	c42692_g1_i1:0-1792	cdRNA01-preDia	cdRNA03-Dia-R2	22.0213	191.532	3.12061	5.00E-05	0.0249845
RNA-Seq	Trinity	XLOC_038496	c42774_g1_i1:0-357	cdRNA03-Dia-R2	cdRNA05-postDia	169.233	16.6046	-3.34935	5.00E-05	0.0249845
RNA-Seq	Trinity	XLOC_038496	c42774_g1_i1:0-357	cdRNA04-Dia-R3	cdRNA05-postDia	156.927	16.6046	-3.24044	5.00E-05	0.0249845
RNA-Seq	Trinity	XLOC_038496	c42774_g1_i1:0-357	cdRNA02-Dia-R1	cdRNA04-Dia-R3	27.8961	156.927	2.49196	5.00E-05	0.0249845
RNA-Seq	Trinity	XLOC_038496	c42774_g1_i1:0-357	cdRNA02-Dia-R1	cdRNA03-Dia-R2	27.8961	169.233	2.60087	5.00E-05	0.0249845
RNA-Seq	Trinity	XLOC_038496	c42774_g1_i1:0-357	cdRNA01-preDia	cdRNA04-Dia-R3	13.3757	156.927	3.55241	5.00E-05	0.0249845
RNA-Seq	Trinity	XLOC_038496	c42774_g1_i1:0-357	cdRNA01-preDia	cdRNA03-Dia-R2	13.3757	169.233	3.66132	5.00E-05	0.0249845
RNA-Seq	Trinity	XLOC_038700	c42959_g1_i1:0-867	cdRNA01-preDia	cdRNA03-Dia-R2	11.86	1.60651	-2.88411	0.0001	0.0432976
RNA-Seq	Trinity	XLOC_038883	c43131_g1_i1:0-412	cdRNA04-Dia-R3	cdRNA05-postDia	7.46987	63.0279	3.07684	5.00E-05	0.0249845
RNA-Seq	Trinity	XLOC_038883	c43131_g1_i1:0-412	cdRNA03-Dia-R2	cdRNA05-postDia	4.55091	63.0279	3.79176	5.00E-05	0.0249845
RNA-Seq	Trinity	XLOC_038914	c43160_g1_i1:1-2457	cdRNA04-Dia-R3	cdRNA05-postDia	5.04933	31.0597	2.62088	5.00E-05	0.0249845
RNA-Seq	Trinity	XLOC_038914	c43160_g1_i1:1-2457	cdRNA03-Dia-R2	cdRNA05-postDia	4.78225	31.0597	2.69928	5.00E-05	0.0249845
RNA-Seq	Trinity	XLOC_039214	c43436_g1_i1:0-1125	cdRNA04-Dia-R3	cdRNA05-postDia	10.7413	65.9926	2.61913	5.00E-05	0.0249845
RNA-Seq	Trinity	XLOC_039214	c43436_g1_i1:0-1125	cdRNA03-Dia-R2	cdRNA05-postDia	10.2798	65.9926	2.68249	5.00E-05	0.0249845
RNA-Seq	Trinity	XLOC_039425	c43639_g1_i1:0-301	cdRNA01-preDia	cdRNA04-Dia-R3	43.1419	221.59	2.36073	0.0001	0.0432976
RNA-Seq	Trinity	XLOC_039576	c4377_g1_i1:0-1274	cdRNA01-preDia	cdRNA04-Dia-R3	12.6297	1.4878	-3.08557	5.00E-05	0.0249845
RNA-Seq	Trinity	XLOC_039576	c4377_g1_i1:0-1274	cdRNA04-Dia-R3	cdRNA05-postDia	1.4878	13.4837	3.17997	5.00E-05	0.0249845
RNA-Seq	Trinity	XLOC_039666	c43862_g1_i1:0-297	cdRNA04-Dia-R3	cdRNA05-postDia	21.0618	116.474	2.4673	5.00E-05	0.0249845
RNA-Seq	Trinity	XLOC_039666	c43862_g1_i1:0-297	cdRNA03-Dia-R2	cdRNA05-postDia	16.2375	116.474	2.8426	5.00E-05	0.0249845
RNA-Seq	Trinity	XLOC_039839	c44026_g1_i1:0-770	cdRNA03-Dia-R2	cdRNA05-postDia	7.1091	39.7844	2.48446	5.00E-05	0.0249845
RNA-Seq	Trinity	XLOC_039853	c44040_g1_i1:0-1071	cdRNA04-Dia-R3	cdRNA05-postDia	14.2083	100.442	2.82157	5.00E-05	0.0249845
RNA-Seq	Trinity	XLOC_039853	c44040_g1_i1:0-1071	cdRNA03-Dia-R2	cdRNA05-postDia	13.5136	100.442	2.89388	5.00E-05	0.0249845
RNA-Seq	Trinity	XLOC_039853	c44040_g1_i1:0-1071	cdRNA02-Dia-R1	cdRNA05-postDia	12.7342	100.442	2.97959	5.00E-05	0.0249845
RNA-Seq	Trinity	XLOC_039874	c44059_g1_i1:0-373	cdRNA01-preDia	cdRNA05-postDia	6.76759	59.3162	3.13171	5.00E-05	0.0249845
RNA-Seq	Trinity	XLOC_040030	c44207_g1_i1:0-2163	cdRNA01-preDia	cdRNA04-Dia-R3	12.0015	2.24442	-2.4188	5.00E-05	0.0249845
RNA-Seq	Trinity	XLOC_040030	c44207_g1_i1:0-2163	cdRNA01-preDia	cdRNA03-Dia-R2	12.0015	2.27553	-2.39894	5.00E-05	0.0249845
RNA-Seq	Trinity	XLOC_040030	c44207_g1_i1:0-2163	cdRNA02-Dia-R1	cdRNA05-postDia	3.53949	19.7345	2.4791	0.0001	0.0432976
RNA-Seq	Trinity	XLOC_040030	c44207_g1_i1:0-2163	cdRNA03-Dia-R2	cdRNA05-postDia	2.27553	19.7345	3.11644	5.00E-05	0.0249845
RNA-Seq	Trinity	XLOC_040030	c44207_g1_i1:0-2163	cdRNA04-Dia-R3	cdRNA05-postDia	2.24442	19.7345	3.1363	5.00E-05	0.0249845
RNA-Seq	Trinity	XLOC_040177	c44342_g1_i1:0-662	cdRNA04-Dia-R3	cdRNA05-postDia	15.8894	110.166	2.79354	5.00E-05	0.0249845
RNA-Seq	Trinity	XLOC_040177	c44342_g1_i1:0-662	cdRNA03-Dia-R2	cdRNA05-postDia	14.5563	110.166	2.91997	5.00E-05	0.0249845
RNA-Seq	Trinity	XLOC_040252	c44411_g1_i1:0-585	cdRNA04-Dia-R3	cdRNA05-postDia	5.4715	40.2728	2.8798	5.00E-05	0.0249845
RNA-Seq	Trinity	XLOC_040275	c44433_g1_i1:0-1046	cdRNA04-Dia-R3	cdRNA05-postDia	51.2279	346.579	2.75818	0.0001	0.0432976
RNA-Seq	Trinity	XLOC_040275	c44433_g1_i1:0-1046	cdRNA03-Dia-R2	cdRNA05-postDia	51.2085	346.579	2.75873	0.0001	0.0432976
RNA-Seq	Trinity	XLOC_040300	c44456_g1_i1:0-572	cdRNA04-Dia-R3	cdRNA05-postDia	1.75093	21.2268	3.59969	0.0001	0.0432976
RNA-Seq	Trinity	XLOC_040491	c44630_g1_i1:0-981	cdRNA01-preDia	cdRNA04-Dia-R3	1.67819	14.1466	3.07548	5.00E-05	0.0249845
RNA-Seq	Trinity	XLOC_040491	c44630_g1_i1:0-981	cdRNA01-preDia	cdRNA03-Dia-R2	1.67819	15.2312	3.18205	5.00E-05	0.0249845
RNA-Seq	Trinity	XLOC_040588	c44719_g1_i1:1-1605	cdRNA03-Dia-R2	cdRNA05-postDia	57.6238	9.87844	-2.54431	5.00E-05	0.0249845
RNA-Seq	Trinity	XLOC_040588	c44719_g1_i1:1-1605	cdRNA04-Dia-R3	cdRNA05-postDia	57.0718	9.87844	-2.53042	5.00E-05	0.0249845

RNA-Seq	Trinity	XLOC_040877	c44991_g1_i1:0-503	cdRNA02-Dia-R1	cdRNA05-postDia	22.2936	119.029	2.41661	0.0001	0.0432976
RNA-Seq	Trinity	XLOC_040887	c45000_g1_i1:0-655	cdRNA01-preDia	cdRNA05-postDia	12.2657	68.4863	2.48118	5.00E-05	0.0249845
RNA-Seq	Trinity	XLOC_040887	c45000_g1_i1:0-655	cdRNA04-Dia-R3	cdRNA05-postDia	8.4882	68.4863	3.01228	5.00E-05	0.0249845
RNA-Seq	Trinity	XLOC_040887	c45000_g1_i1:0-655	cdRNA03-Dia-R2	cdRNA05-postDia	7.7847	68.4863	3.1371	5.00E-05	0.0249845
RNA-Seq	Trinity	XLOC_041220	c45312_g1_i1:0-584	cdRNA02-Dia-R1	cdRNA05-postDia	9.24072	54.5376	2.56117	5.00E-05	0.0249845
RNA-Seq	Trinity	XLOC_041379	c45457_g1_i1:0-878	cdRNA01-preDia	cdRNA02-Dia-R1	32.0095	292.333	3.19104	5.00E-05	0.0249845
RNA-Seq	Trinity	XLOC_041379	c45457_g1_i1:0-878	cdRNA01-preDia	cdRNA05-postDia	32.0095	560.098	4.12911	5.00E-05	0.0249845
RNA-Seq	Trinity	XLOC_041413	c45492_g1_i1:0-432	cdRNA03-Dia-R2	cdRNA05-postDia	13.668	69.3935	2.344	0.0001	0.0432976
RNA-Seq	Trinity	XLOC_041494	c45568_g1_i1:0-602	cdRNA01-preDia	cdRNA04-Dia-R3	215.138	14.6658	-3.87473	5.00E-05	0.0249845
RNA-Seq	Trinity	XLOC_041494	c45568_g1_i1:0-602	cdRNA01-preDia	cdRNA03-Dia-R2	215.138	15.7594	-3.77097	5.00E-05	0.0249845
RNA-Seq	Trinity	XLOC_041494	c45568_g1_i1:0-602	cdRNA01-preDia	cdRNA02-Dia-R1	215.138	26.0946	-3.04344	5.00E-05	0.0249845
RNA-Seq	Trinity	XLOC_041494	c45568_g1_i1:0-602	cdRNA01-preDia	cdRNA05-postDia	215.138	29.5404	-2.8645	5.00E-05	0.0249845
RNA-Seq	Trinity	XLOC_041841	c45897_g1_i1:1-202	cdRNA04-Dia-R3	cdRNA05-postDia	51.8201	609.156	3.55523	5.00E-05	0.0249845
RNA-Seq	Trinity	XLOC_041841	c45897_g1_i1:1-202	cdRNA03-Dia-R2	cdRNA05-postDia	48.4016	609.156	3.65368	5.00E-05	0.0249845
RNA-Seq	Trinity	XLOC_041841	c45897_g1_i1:1-202	cdRNA01-preDia	cdRNA05-postDia	41.5553	609.156	3.87371	5.00E-05	0.0249845
RNA-Seq	Trinity	XLOC_041860	c45914_g1_i1:0-3713	cdRNA01-preDia	cdRNA04-Dia-R3	19.9654	3.79145	-2.39668	0.0001	0.0432976
RNA-Seq	Trinity	XLOC_041860	c45914_g1_i1:0-3713	cdRNA03-Dia-R2	cdRNA05-postDia	4.19338	26.7029	2.67081	0.0001	0.0432976
RNA-Seq	Trinity	XLOC_041860	c45914_g1_i1:0-3713	cdRNA04-Dia-R3	cdRNA05-postDia	3.79145	26.7029	2.81618	5.00E-05	0.0249845
RNA-Seq	Trinity	XLOC_041908	c4595_g1_i1:1-2071	cdRNA03-Dia-R2	cdRNA05-postDia	5.98291	31.0129	2.37395	5.00E-05	0.0249845
RNA-Seq	Trinity	XLOC_041931	c45981_g1_i1:0-459	cdRNA02-Dia-R1	cdRNA05-postDia	16.9211	81.7392	2.27221	0.0001	0.0432976
RNA-Seq	Trinity	XLOC_042045	c46086_g1_i1:0-758	cdRNA01-preDia	cdRNA02-Dia-R1	32.9516	1.61148	-4.35389	5.00E-05	0.0249845
RNA-Seq	Trinity	XLOC_042105	c46141_g1_i1:0-480	cdRNA02-Dia-R1	cdRNA05-postDia	2.372	27.4356	3.53187	0.0001	0.0432976
RNA-Seq	Trinity	XLOC_042215	c46248_g1_i1:0-330	cdRNA03-Dia-R2	cdRNA05-postDia	9.89437	103.711	3.38982	5.00E-05	0.0249845
RNA-Seq	Trinity	XLOC_042215	c46248_g1_i1:0-330	cdRNA04-Dia-R3	cdRNA05-postDia	8.91255	103.711	3.54059	5.00E-05	0.0249845
RNA-Seq	Trinity	XLOC_042215	c46248_g1_i1:0-330	cdRNA02-Dia-R1	cdRNA05-postDia	7.23072	103.711	3.84229	5.00E-05	0.0249845
RNA-Seq	Trinity	XLOC_042279	c46307_g1_i1:0-709	cdRNA01-preDia	cdRNA04-Dia-R3	77.6965	11.4084	-2.76775	5.00E-05	0.0249845
RNA-Seq	Trinity	XLOC_042279	c46307_g1_i1:0-709	cdRNA01-preDia	cdRNA03-Dia-R2	77.6965	12.5766	-2.6271	5.00E-05	0.0249845
RNA-Seq	Trinity	XLOC_042279	c46307_g1_i1:0-709	cdRNA03-Dia-R2	cdRNA05-postDia	12.5766	78.9992	2.65109	5.00E-05	0.0249845
RNA-Seq	Trinity	XLOC_042279	c46307_g1_i1:0-709	cdRNA04-Dia-R3	cdRNA05-postDia	11.4084	78.9992	2.79174	5.00E-05	0.0249845
RNA-Seq	Trinity	XLOC_042726	c46721_g1_i1:0-211	cdRNA01-preDia	cdRNA05-postDia	52.6912	298.801	2.50355	0.0001	0.0432976
RNA-Seq	Trinity	XLOC_042726	c46721_g1_i1:0-211	cdRNA02-Dia-R1	cdRNA05-postDia	43.2105	298.801	2.78973	5.00E-05	0.0249845
RNA-Seq	Trinity	XLOC_042817	c46806_g1_i1:0-880	cdRNA01-preDia	cdRNA04-Dia-R3	1.4613	12.3353	3.07747	0.0001	0.0432976
RNA-Seq	Trinity	XLOC_042817	c46806_g1_i1:0-880	cdRNA01-preDia	cdRNA03-Dia-R2	1.4613	13.7137	3.23029	5.00E-05	0.0249845
RNA-Seq	Trinity	XLOC_042867	c46853_g1_i1:0-1004	cdRNA01-preDia	cdRNA05-postDia	6.17401	32.0335	2.3753	0.0001	0.0432976
RNA-Seq	Trinity	XLOC_043008	c46983_g1_i1:0-206	cdRNA01-preDia	cdRNA05-postDia	66.1273	1042.81	3.97908	5.00E-05	0.0249845
RNA-Seq	Trinity	XLOC_043008	c46983_g1_i1:0-206	cdRNA02-Dia-R1	cdRNA05-postDia	45.9713	1042.81	4.5036	5.00E-05	0.0249845
RNA-Seq	Trinity	XLOC_043016	c46990_g1_i1:0-280	cdRNA02-Dia-R1	cdRNA05-postDia	18.0422	106.802	2.56549	0.0001	0.0432976
RNA-Seq	Trinity	XLOC_043369	c47323_g1_i1:0-557	cdRNA01-preDia	cdRNA05-postDia	43.0948	240.819	2.48237	5.00E-05	0.0249845
RNA-Seq	Trinity	XLOC_043759	c4767_g1_i1:0-324	cdRNA01-preDia	cdRNA05-postDia	15.5572	114.091	2.87453	5.00E-05	0.0249845
RNA-Seq	Trinity	XLOC_043815	c47730_g1_i1:3-617	cdRNA03-Dia-R2	cdRNA05-postDia	41.4687	230.67	2.47574	0.0001	0.0432976
RNA-Seq	Trinity	XLOC_043815	c47730_g1_i1:3-617	cdRNA02-Dia-R1	cdRNA05-postDia	40.0649	230.67	2.52542	0.0001	0.0432976
RNA-Seq	Trinity	XLOC_043815	c47730_g1_i1:3-617	cdRNA01-preDia	cdRNA05-postDia	36.14	230.67	2.67416	5.00E-05	0.0249845
RNA-Seq	Trinity	XLOC_044040	c47940_g1_i1:1-704	cdRNA04-Dia-R3	cdRNA05-postDia	22.1037	3.3545	-2.72011	5.00E-05	0.0249845
RNA-Seq	Trinity	XLOC_044040	c47940_g1_i1:1-704	cdRNA03-Dia-R2	cdRNA05-postDia	20.2381	3.3545	-2.5929	0.0001	0.0432976
RNA-Seq	Trinity	XLOC_044040	c47940_g1_i1:1-704	cdRNA01-preDia	cdRNA03-Dia-R2	1.82125	20.2381	3.47407	5.00E-05	0.0249845
RNA-Seq	Trinity	XLOC_044040	c47940_g1_i1:1-704	cdRNA01-preDia	cdRNA04-Dia-R3	1.82125	22.1037	3.60128	5.00E-05	0.0249845
RNA-Seq	Trinity	XLOC_044094	c47991_g1_i1:2-845	cdRNA02-Dia-R1	cdRNA05-postDia	16.0669	119.003	2.88884	5.00E-05	0.0249845

RNA-Seq	Trinity	XLOC_044094	c47991_g1_i1:2-845	cdRNA01-preDia	cdRNA05-postDia	6.12181	119.003	4.2809	5.00E-05	0.0249845
RNA-Seq	Trinity	XLOC_044094	c47991_g1_i1:2-845	cdRNA03-Dia-R2	cdRNA05-postDia	4.56839	119.003	4.70318	5.00E-05	0.0249845
RNA-Seq	Trinity	XLOC_044094	c47991_g1_i1:2-845	cdRNA04-Dia-R3	cdRNA05-postDia	3.67761	119.003	5.01609	5.00E-05	0.0249845
RNA-Seq	Trinity	XLOC_044353	c48238_g1_i1:0-539	cdRNA01-preDia	cdRNA04-Dia-R3	4.71795	34.0556	2.85166	5.00E-05	0.0249845
RNA-Seq	Trinity	XLOC_044353	c48238_g1_i1:0-539	cdRNA01-preDia	cdRNA03-Dia-R2	4.71795	34.2667	2.86058	5.00E-05	0.0249845
RNA-Seq	Trinity	XLOC_044397	c48280_g1_i1:0-1143	cdRNA03-Dia-R2	cdRNA05-postDia	7.56209	35.6532	2.23718	0.0001	0.0432976
RNA-Seq	Trinity	XLOC_044397	c48280_g1_i1:0-1143	cdRNA04-Dia-R3	cdRNA05-postDia	6.5796	35.6532	2.43796	0.0001	0.0432976
RNA-Seq	Trinity	XLOC_044397	c48280_g1_i1:0-1143	cdRNA01-preDia	cdRNA05-postDia	6.18586	35.6532	2.52699	5.00E-05	0.0249845
RNA-Seq	Trinity	XLOC_044397	c48280_g1_i1:0-1143	cdRNA02-Dia-R1	cdRNA05-postDia	4.31012	35.6532	3.04823	5.00E-05	0.0249845
RNA-Seq	Trinity	XLOC_044500	c48376_g1_i1:0-1113	cdRNA01-preDia	cdRNA03-Dia-R2	9.75963	1.46822	-2.73276	5.00E-05	0.0249845
RNA-Seq	Trinity	XLOC_044500	c48376_g1_i1:0-1113	cdRNA01-preDia	cdRNA04-Dia-R3	9.75963	1.67905	-2.53918	0.0001	0.0432976
RNA-Seq	Trinity	XLOC_044614	c48485_g1_i1:0-355	cdRNA04-Dia-R3	cdRNA05-postDia	10.4965	62.8057	2.58099	0.0001	0.0432976
RNA-Seq	Trinity	XLOC_044621	c48492_g1_i1:0-298	cdRNA01-preDia	cdRNA05-postDia	13.2964	118.669	3.15784	5.00E-05	0.0249845
RNA-Seq	Trinity	XLOC_044721	c48587_g1_i1:2-589	cdRNA01-preDia	cdRNA05-postDia	12.5864	61.6026	2.29113	0.0001	0.0432976
RNA-Seq	Trinity	XLOC_044721	c48587_g1_i1:2-589	cdRNA02-Dia-R1	cdRNA05-postDia	11.4216	61.6026	2.43123	5.00E-05	0.0249845
RNA-Seq	Trinity	XLOC_044838	c48696_g1_i1:2-374	cdRNA03-Dia-R2	cdRNA05-postDia	97.7225	17.5202	-2.47967	5.00E-05	0.0249845
RNA-Seq	Trinity	XLOC_044838	c48696_g1_i1:2-374	cdRNA04-Dia-R3	cdRNA05-postDia	93.2183	17.5202	-2.41159	5.00E-05	0.0249845
RNA-Seq	Trinity	XLOC_044838	c48696_g1_i1:2-374	cdRNA01-preDia	cdRNA04-Dia-R3	12.6755	93.2183	2.87857	5.00E-05	0.0249845
RNA-Seq	Trinity	XLOC_044838	c48696_g1_i1:2-374	cdRNA01-preDia	cdRNA03-Dia-R2	12.6755	97.7225	2.94665	5.00E-05	0.0249845
RNA-Seq	Trinity	XLOC_045011	c48858_g1_i1:0-637	cdRNA02-Dia-R1	cdRNA05-postDia	26.5283	2.11699	-3.64745	5.00E-05	0.0249845
RNA-Seq	Trinity	XLOC_045156	c48998_g1_i1:0-659	cdRNA01-preDia	cdRNA04-Dia-R3	2.77895	17.2437	2.63346	0.0001	0.0432976
RNA-Seq	Trinity	XLOC_045522	c49346_g1_i1:0-430	cdRNA01-preDia	cdRNA05-postDia	4.54558	64.8933	3.83553	5.00E-05	0.0249845
RNA-Seq	Trinity	XLOC_045837	c49644_g1_i1:0-1846	cdRNA02-Dia-R1	cdRNA04-Dia-R3	14.4989	2.49305	-2.53996	5.00E-05	0.0249845
RNA-Seq	Trinity	XLOC_045837	c49644_g1_i1:0-1846	cdRNA02-Dia-R1	cdRNA03-Dia-R2	14.4989	2.78691	-2.37921	5.00E-05	0.0249845
RNA-Seq	Trinity	XLOC_045837	c49644_g1_i1:0-1846	cdRNA01-preDia	cdRNA05-postDia	3.00807	91.9685	4.93423	5.00E-05	0.0249845
RNA-Seq	Trinity	XLOC_045837	c49644_g1_i1:0-1846	cdRNA03-Dia-R2	cdRNA05-postDia	2.78691	91.9685	5.0444	5.00E-05	0.0249845
RNA-Seq	Trinity	XLOC_045837	c49644_g1_i1:0-1846	cdRNA04-Dia-R3	cdRNA05-postDia	2.49305	91.9685	5.20516	5.00E-05	0.0249845
RNA-Seq	Trinity	XLOC_045923	c49724_g1_i1:0-325	cdRNA01-preDia	cdRNA05-postDia	7.71902	57.7441	2.90318	5.00E-05	0.0249845
RNA-Seq	Trinity	XLOC_045978	c49778_g1_i1:0-344	cdRNA02-Dia-R1	cdRNA05-postDia	15.8303	117.03	2.88612	5.00E-05	0.0249845
RNA-Seq	Trinity	XLOC_045978	c49778_g1_i1:0-344	cdRNA03-Dia-R2	cdRNA05-postDia	12.0313	117.03	3.28201	5.00E-05	0.0249845
RNA-Seq	Trinity	XLOC_045985	c49784_g1_i1:0-1200	cdRNA02-Dia-R1	cdRNA05-postDia	4.54152	23.2489	2.35592	5.00E-05	0.0249845
RNA-Seq	Trinity	XLOC_045985	c49784_g1_i1:0-1200	cdRNA01-preDia	cdRNA05-postDia	4.24496	23.2489	2.45334	5.00E-05	0.0249845
RNA-Seq	Trinity	XLOC_045985	c49784_g1_i1:0-1200	cdRNA03-Dia-R2	cdRNA05-postDia	4.23799	23.2489	2.45571	5.00E-05	0.0249845
RNA-Seq	Trinity	XLOC_046211	c49997_g1_i1:0-877	cdRNA01-preDia	cdRNA05-postDia	7.22165	44.7702	2.63214	5.00E-05	0.0249845
RNA-Seq	Trinity	XLOC_046364	c50143_g1_i1:18-526	cdRNA02-Dia-R1	cdRNA05-postDia	6.06907	47.4578	2.9671	5.00E-05	0.0249845
RNA-Seq	Trinity	XLOC_046401	c5017_g1_i1:0-1059	cdRNA02-Dia-R1	cdRNA05-postDia	9.22349	41.7197	2.17734	0.0001	0.0432976
RNA-Seq	Trinity	XLOC_046401	c5017_g1_i1:0-1059	cdRNA04-Dia-R3	cdRNA05-postDia	6.11772	41.7197	2.76966	5.00E-05	0.0249845
RNA-Seq	Trinity	XLOC_046401	c5017_g1_i1:0-1059	cdRNA03-Dia-R2	cdRNA05-postDia	5.25984	41.7197	2.98764	5.00E-05	0.0249845
RNA-Seq	Trinity	XLOC_047096	c50846_g1_i1:0-342	cdRNA04-Dia-R3	cdRNA05-postDia	115.567	20.6353	-2.48555	5.00E-05	0.0249845
RNA-Seq	Trinity	XLOC_047096	c50846_g1_i1:0-342	cdRNA03-Dia-R2	cdRNA05-postDia	109.216	20.6353	-2.40401	0.0001	0.0432976
RNA-Seq	Trinity	XLOC_047108	c50857_g1_i1:1-722	cdRNA02-Dia-R1	cdRNA04-Dia-R3	16.8651	2.05277	-3.0384	5.00E-05	0.0249845
RNA-Seq	Trinity	XLOC_047108	c50857_g1_i1:1-722	cdRNA02-Dia-R1	cdRNA05-postDia	16.8651	116.866	2.79275	5.00E-05	0.0249845
RNA-Seq	Trinity	XLOC_047108	c50857_g1_i1:1-722	cdRNA01-preDia	cdRNA05-postDia	4.3564	116.866	4.74558	5.00E-05	0.0249845
RNA-Seq	Trinity	XLOC_047108	c50857_g1_i1:1-722	cdRNA03-Dia-R2	cdRNA05-postDia	3.12841	116.866	5.22328	5.00E-05	0.0249845
RNA-Seq	Trinity	XLOC_047108	c50857_g1_i1:1-722	cdRNA04-Dia-R3	cdRNA05-postDia	2.05277	116.866	5.83114	5.00E-05	0.0249845
RNA-Seq	Trinity	XLOC_047186	c50932_g1_i1:0-328	cdRNA01-preDia	cdRNA05-postDia	10.3745	64.6873	2.64044	0.0001	0.0432976
RNA-Seq	Trinity	XLOC_047269	c51012_g1_i1:0-211	cdRNA03-Dia-R2	cdRNA05-postDia	490.977	75.586	-2.69946	0.0001	0.0432976

RNA-Seq	Trinity	XLOC_047528	c5125_g1_i1:0-2955	cdRNA03-Dia-R2	cdRNA05-postDia	17.0646	2.63675	-2.69417	5.00E-05	0.0249845
RNA-Seq	Trinity	XLOC_047528	c5125_g1_i1:0-2955	cdRNA04-Dia-R3	cdRNA05-postDia	16.7404	2.63675	-2.6665	5.00E-05	0.0249845
RNA-Seq	Trinity	XLOC_047528	c5125_g1_i1:0-2955	cdRNA01-preDia	cdRNA04-Dia-R3	2.34126	16.7404	2.83798	5.00E-05	0.0249845
RNA-Seq	Trinity	XLOC_047528	c5125_g1_i1:0-2955	cdRNA01-preDia	cdRNA03-Dia-R2	2.34126	17.0646	2.86565	5.00E-05	0.0249845
RNA-Seq	Trinity	XLOC_047644	c51372_g1_i1:0-864	cdRNA01-preDia	cdRNA04-Dia-R3	36.4004	4.47946	-3.02256	5.00E-05	0.0249845
RNA-Seq	Trinity	XLOC_047644	c51372_g1_i1:0-864	cdRNA01-preDia	cdRNA02-Dia-R1	36.4004	7.66512	-2.24757	0.0001	0.0432976
RNA-Seq	Trinity	XLOC_047644	c51372_g1_i1:0-864	cdRNA02-Dia-R1	cdRNA05-postDia	7.66512	47.524	2.63228	5.00E-05	0.0249845
RNA-Seq	Trinity	XLOC_047644	c51372_g1_i1:0-864	cdRNA04-Dia-R3	cdRNA05-postDia	4.47946	47.524	3.40726	5.00E-05	0.0249845
RNA-Seq	Trinity	XLOC_047644	c51372_g1_i1:0-864	cdRNA03-Dia-R2	cdRNA05-postDia	3.64601	47.524	3.70427	5.00E-05	0.0249845
RNA-Seq	Trinity	XLOC_048049	c5175_g1_i2:0-1340	cdRNA01-preDia	cdRNA05-postDia	0.869583	7.26236	3.06204	0.0001	0.0432976
RNA-Seq	Trinity	XLOC_048097	c51802_g1_i1:0-930	cdRNA03-Dia-R2	cdRNA05-postDia	4.50998	26.0759	2.53153	5.00E-05	0.0249845
RNA-Seq	Trinity	XLOC_048097	c51802_g1_i1:0-930	cdRNA04-Dia-R3	cdRNA05-postDia	3.1847	26.0759	3.03349	5.00E-05	0.0249845
RNA-Seq	Trinity	XLOC_048158	c51861_g1_i1:0-440	cdRNA04-Dia-R3	cdRNA05-postDia	47.859	7.09258	-2.75441	5.00E-05	0.0249845
RNA-Seq	Trinity	XLOC_048158	c51861_g1_i1:0-440	cdRNA03-Dia-R2	cdRNA05-postDia	46.6347	7.09258	-2.71702	5.00E-05	0.0249845
RNA-Seq	Trinity	XLOC_049292	c5295_g1_i1:0-394	cdRNA02-Dia-R1	cdRNA05-postDia	11.8962	65.1963	2.45429	0.0001	0.0432976
RNA-Seq	Trinity	XLOC_049303	c52970_g1_i1:1-347	cdRNA01-preDia	cdRNA03-Dia-R2	85.1525	13.5247	-2.65445	5.00E-05	0.0249845
RNA-Seq	Trinity	XLOC_049693	c53345_g1_i1:0-497	cdRNA04-Dia-R3	cdRNA05-postDia	36.0911	3.94459	-3.1937	5.00E-05	0.0249845
RNA-Seq	Trinity	XLOC_049693	c53345_g1_i1:0-497	cdRNA03-Dia-R2	cdRNA05-postDia	35.0048	3.94459	-3.1496	5.00E-05	0.0249845
RNA-Seq	Trinity	XLOC_049693	c53345_g1_i1:0-497	cdRNA01-preDia	cdRNA03-Dia-R2	5.1245	35.0048	2.77207	5.00E-05	0.0249845
RNA-Seq	Trinity	XLOC_049693	c53345_g1_i1:0-497	cdRNA01-preDia	cdRNA04-Dia-R3	5.1245	36.0911	2.81616	5.00E-05	0.0249845
RNA-Seq	Trinity	XLOC_050016	c53657_g1_i1:0-1041	cdRNA01-preDia	cdRNA05-postDia	34.4152	3.76171	-3.19358	5.00E-05	0.0249845
RNA-Seq	Trinity	XLOC_050016	c53657_g1_i1:0-1041	cdRNA01-preDia	cdRNA02-Dia-R1	34.4152	5.65311	-2.60593	5.00E-05	0.0249845
RNA-Seq	Trinity	XLOC_050048	c5368_g1_i1:0-447	cdRNA01-preDia	cdRNA05-postDia	3.90244	28.3732	2.86208	5.00E-05	0.0249845
RNA-Seq	Trinity	XLOC_050107	c5374_g1_i1:8-600	cdRNA02-Dia-R1	cdRNA05-postDia	9.74047	70.9778	2.86531	5.00E-05	0.0249845
RNA-Seq	Trinity	XLOC_050107	c5374_g1_i1:8-600	cdRNA03-Dia-R2	cdRNA05-postDia	7.72695	70.9778	3.1994	5.00E-05	0.0249845
RNA-Seq	Trinity	XLOC_050107	c5374_g1_i1:8-600	cdRNA04-Dia-R3	cdRNA05-postDia	5.95891	70.9778	3.57425	5.00E-05	0.0249845
RNA-Seq	Trinity	XLOC_050107	c5374_g1_i1:8-600	cdRNA01-preDia	cdRNA05-postDia	5.09188	70.9778	3.8011	5.00E-05	0.0249845
RNA-Seq	Trinity	XLOC_050115	c53757_g1_i1:5-281	cdRNA04-Dia-R3	cdRNA05-postDia	19.6386	176.28	3.1661	5.00E-05	0.0249845
RNA-Seq	Trinity	XLOC_050115	c53757_g1_i1:5-281	cdRNA03-Dia-R2	cdRNA05-postDia	19.4424	176.28	3.18059	5.00E-05	0.0249845
RNA-Seq	Trinity	XLOC_050335	c53972_g1_i1:0-431	cdRNA03-Dia-R2	cdRNA05-postDia	4.34436	31.6016	2.86278	5.00E-05	0.0249845
RNA-Seq	Trinity	XLOC_050642	c5427_g1_i1:0-743	cdRNA03-Dia-R2	cdRNA05-postDia	3.7326	25.2882	2.76021	5.00E-05	0.0249845
RNA-Seq	Trinity	XLOC_050924	c54555_g1_i1:0-577	cdRNA04-Dia-R3	cdRNA05-postDia	10.9745	50.646	2.2063	0.0001	0.0432976
RNA-Seq	Trinity	XLOC_050924	c54555_g1_i1:0-577	cdRNA03-Dia-R2	cdRNA05-postDia	8.56647	50.646	2.56368	5.00E-05	0.0249845
RNA-Seq	Trinity	XLOC_050949	c54579_g1_i1:0-381	cdRNA02-Dia-R1	cdRNA05-postDia	6.54873	45.2019	2.7871	5.00E-05	0.0249845
RNA-Seq	Trinity	XLOC_050949	c54579_g1_i1:0-381	cdRNA04-Dia-R3	cdRNA05-postDia	5.13292	45.2019	3.13853	0.0001	0.0432976
RNA-Seq	Trinity	XLOC_052179	c55826_g1_i1:0-542	cdRNA01-preDia	cdRNA05-postDia	3.74086	42.2125	3.49623	5.00E-05	0.0249845
RNA-Seq	Trinity	XLOC_052757	c563_g1_i1:0-1350	cdRNA01-preDia	cdRNA03-Dia-R2	10.0336	71.3593	2.83026	5.00E-05	0.0249845
RNA-Seq	Trinity	XLOC_052757	c563_g1_i1:0-1350	cdRNA01-preDia	cdRNA04-Dia-R3	10.0336	73.0947	2.86492	5.00E-05	0.0249845
RNA-Seq	Trinity	XLOC_054102	c5779_g1_i1:0-2092	cdRNA04-Dia-R3	cdRNA05-postDia	6.96639	40.4465	2.53753	5.00E-05	0.0249845
RNA-Seq	Trinity	XLOC_054356	c58067_g1_i1:1-266	cdRNA01-preDia	cdRNA05-postDia	37.1369	292.284	2.97645	5.00E-05	0.0249845
RNA-Seq	Trinity	XLOC_054356	c58067_g1_i1:1-266	cdRNA04-Dia-R3	cdRNA05-postDia	22.1296	292.284	3.72332	5.00E-05	0.0249845
RNA-Seq	Trinity	XLOC_054356	c58067_g1_i1:1-266	cdRNA03-Dia-R2	cdRNA05-postDia	19.6615	292.284	3.89393	5.00E-05	0.0249845
RNA-Seq	Trinity	XLOC_054356	c58067_g1_i1:1-266	cdRNA02-Dia-R1	cdRNA05-postDia	17.3746	292.284	4.07232	5.00E-05	0.0249845
RNA-Seq	Trinity	XLOC_054557	c5830_g1_i2:0-1468	cdRNA01-preDia	cdRNA05-postDia	6.6586	90.1475	3.759	5.00E-05	0.0249845
RNA-Seq	Trinity	XLOC_054557	c5830_g1_i2:0-1468	cdRNA02-Dia-R1	cdRNA05-postDia	2.87231	90.1475	4.97201	5.00E-05	0.0249845
RNA-Seq	Trinity	XLOC_054758	c58515_g1_i1:0-1096	cdRNA04-Dia-R3	cdRNA05-postDia	118.979	15.1021	-2.97789	5.00E-05	0.0249845
RNA-Seq	Trinity	XLOC_054758	c58515_g1_i1:0-1096	cdRNA03-Dia-R2	cdRNA05-postDia	109.988	15.1021	-2.86453	5.00E-05	0.0249845

RNA-Seq	Trinity	XLOC_054781	c58537_g1_i1:0-1049	cdRNA04-Dia-R3	cdRNA05-postDia	129.858	24.7621	-2.39073	0.0001	0.0432976
RNA-Seq	Trinity	XLOC_054805	c58557_g1_i1:0-709	cdRNA04-Dia-R3	cdRNA05-postDia	173.753	31.4892	-2.46411	5.00E-05	0.0249845
RNA-Seq	Trinity	XLOC_055003	c58743_g1_i1:0-1550	cdRNA01-preDia	cdRNA03-Dia-R2	13.416	80.5687	2.58627	0.0001	0.0432976
RNA-Seq	Trinity	XLOC_055003	c58743_g1_i1:0-1550	cdRNA01-preDia	cdRNA04-Dia-R3	13.416	86.2262	2.68418	5.00E-05	0.0249845
RNA-Seq	Trinity	XLOC_055003	c58743_g1_i1:0-1550	cdRNA01-preDia	cdRNA02-Dia-R1	13.416	165.731	3.62682	5.00E-05	0.0249845
RNA-Seq	Trinity	XLOC_055003	c58743_g1_i1:0-1550	cdRNA01-preDia	cdRNA05-postDia	13.416	518.208	5.27151	5.00E-05	0.0249845
RNA-Seq	Trinity	XLOC_055051	c58787_g1_i1:0-1414	cdRNA04-Dia-R3	cdRNA05-postDia	42.3203	3.75496	-3.49448	5.00E-05	0.0249845
RNA-Seq	Trinity	XLOC_055051	c58787_g1_i1:0-1414	cdRNA03-Dia-R2	cdRNA05-postDia	39.8076	3.75496	-3.40617	5.00E-05	0.0249845
RNA-Seq	Trinity	XLOC_055066	c58801_g1_i1:1-2318	cdRNA02-Dia-R1	cdRNA05-postDia	1.69006	8.77085	2.37564	5.00E-05	0.0249845
RNA-Seq	Trinity	XLOC_055066	c58801_g1_i1:1-2318	cdRNA04-Dia-R3	cdRNA05-postDia	1.20187	8.77085	2.86744	0.0001	0.0432976
RNA-Seq	Trinity	XLOC_055066	c58801_g1_i1:1-2318	cdRNA03-Dia-R2	cdRNA05-postDia	0.989497	8.77085	3.14795	5.00E-05	0.0249845
RNA-Seq	Trinity	XLOC_055085	c58820_g1_i1:0-340	cdRNA01-preDia	cdRNA05-postDia	79.8129	417.334	2.38651	5.00E-05	0.0249845
RNA-Seq	Trinity	XLOC_055085	c58820_g1_i1:0-340	cdRNA02-Dia-R1	cdRNA05-postDia	73.3462	417.334	2.50841	5.00E-05	0.0249845
RNA-Seq	Trinity	XLOC_055107	c58841_g1_i1:0-849	cdRNA03-Dia-R2	cdRNA05-postDia	9.66339	56.8474	2.55649	5.00E-05	0.0249845
RNA-Seq	Trinity	XLOC_055107	c58841_g1_i1:0-849	cdRNA04-Dia-R3	cdRNA05-postDia	9.10849	56.8474	2.64181	5.00E-05	0.0249845
RNA-Seq	Trinity	XLOC_055125	c58857_g1_i1:8-2060	cdRNA01-preDia	cdRNA02-Dia-R1	6.30771	33.7849	2.42119	0.0001	0.0432976
RNA-Seq	Trinity	XLOC_055125	c58857_g1_i1:8-2060	cdRNA01-preDia	cdRNA04-Dia-R3	6.30771	46.8257	2.89211	5.00E-05	0.0249845
RNA-Seq	Trinity	XLOC_055125	c58857_g1_i1:8-2060	cdRNA01-preDia	cdRNA03-Dia-R2	6.30771	47.0273	2.89831	5.00E-05	0.0249845
RNA-Seq	Trinity	XLOC_055254	c58977_g1_i1:0-643	cdRNA02-Dia-R1	cdRNA05-postDia	22.0985	135.179	2.61285	5.00E-05	0.0249845
RNA-Seq	Trinity	XLOC_055254	c58977_g1_i1:0-643	cdRNA03-Dia-R2	cdRNA05-postDia	15.9051	135.179	3.08731	5.00E-05	0.0249845
RNA-Seq	Trinity	XLOC_055254	c58977_g1_i1:0-643	cdRNA04-Dia-R3	cdRNA05-postDia	13.9869	135.179	3.27272	5.00E-05	0.0249845
RNA-Seq	Trinity	XLOC_055364	c59077_g1_i1:0-640	cdRNA03-Dia-R2	cdRNA05-postDia	4.61436	28.8854	2.64614	5.00E-05	0.0249845
RNA-Seq	Trinity	XLOC_055364	c59077_g1_i1:0-640	cdRNA02-Dia-R1	cdRNA05-postDia	4.29995	28.8854	2.74795	5.00E-05	0.0249845
RNA-Seq	Trinity	XLOC_055364	c59077_g1_i1:0-640	cdRNA01-preDia	cdRNA05-postDia	1.26877	28.8854	4.50884	5.00E-05	0.0249845
RNA-Seq	Trinity	XLOC_055436	c59145_g1_i1:0-779	cdRNA01-preDia	cdRNA03-Dia-R2	3307.79	208.769	-3.98589	5.00E-05	0.0249845
RNA-Seq	Trinity	XLOC_055436	c59145_g1_i1:0-779	cdRNA01-preDia	cdRNA04-Dia-R3	3307.79	229.198	-3.8512	5.00E-05	0.0249845
RNA-Seq	Trinity	XLOC_055436	c59145_g1_i1:0-779	cdRNA01-preDia	cdRNA05-postDia	3307.79	333.672	-3.30937	5.00E-05	0.0249845
RNA-Seq	Trinity	XLOC_055581	c59280_g1_i1:0-2175	cdRNA03-Dia-R2	cdRNA05-postDia	437.684	58.0948	-2.91341	5.00E-05	0.0249845
RNA-Seq	Trinity	XLOC_055599	c59297_g1_i1:0-571	cdRNA03-Dia-R2	cdRNA05-postDia	626.224	53.1029	-3.55982	5.00E-05	0.0249845
RNA-Seq	Trinity	XLOC_055599	c59297_g1_i1:0-571	cdRNA04-Dia-R3	cdRNA05-postDia	615.528	53.1029	-3.53496	5.00E-05	0.0249845
RNA-Seq	Trinity	XLOC_055599	c59297_g1_i1:0-571	cdRNA02-Dia-R1	cdRNA05-postDia	308.691	53.1029	-2.5393	5.00E-05	0.0249845
RNA-Seq	Trinity	XLOC_055916	c595_g1_i1:0-642	cdRNA03-Dia-R2	cdRNA05-postDia	44.4018	3.36671	-3.72121	5.00E-05	0.0249845
RNA-Seq	Trinity	XLOC_055916	c595_g1_i1:0-642	cdRNA04-Dia-R3	cdRNA05-postDia	34.6144	3.36671	-3.36196	5.00E-05	0.0249845
RNA-Seq	Trinity	XLOC_055916	c595_g1_i1:0-642	cdRNA02-Dia-R1	cdRNA05-postDia	19.4313	3.36671	-2.52897	0.0001	0.0432976
RNA-Seq	Trinity	XLOC_055916	c595_g1_i1:0-642	cdRNA01-preDia	cdRNA04-Dia-R3	6.31489	34.6144	2.45454	5.00E-05	0.0249845
RNA-Seq	Trinity	XLOC_055916	c595_g1_i1:0-642	cdRNA01-preDia	cdRNA03-Dia-R2	6.31489	44.4018	2.81379	5.00E-05	0.0249845
RNA-Seq	Trinity	XLOC_055938	c59619_g2_i1:0-1205	cdRNA04-Dia-R3	cdRNA05-postDia	7.76242	40.6117	2.38732	0.0001	0.0432976
RNA-Seq	Trinity	XLOC_056103	c59775_g1_i1:0-1348	cdRNA04-Dia-R3	cdRNA05-postDia	10.3042	51.3172	2.31621	0.0001	0.0432976
RNA-Seq	Trinity	XLOC_056231	c59894_g1_i1:22-947	cdRNA01-preDia	cdRNA04-Dia-R3	5.37078	26.3377	2.29393	0.0001	0.0432976
RNA-Seq	Trinity	XLOC_056231	c59894_g1_i1:22-947	cdRNA01-preDia	cdRNA03-Dia-R2	5.37078	26.5362	2.30476	0.0001	0.0432976
RNA-Seq	Trinity	XLOC_056261	c59921_g1_i1:0-256	cdRNA01-preDia	cdRNA05-postDia	291.686	1930.93	2.7268	5.00E-05	0.0249845
RNA-Seq	Trinity	XLOC_056261	c59921_g1_i1:0-256	cdRNA02-Dia-R1	cdRNA05-postDia	225.792	1930.93	3.09623	5.00E-05	0.0249845
RNA-Seq	Trinity	XLOC_056347	c60000_g1_i1:1-1402	cdRNA03-Dia-R2	cdRNA05-postDia	7.44818	42.0195	2.4961	5.00E-05	0.0249845
RNA-Seq	Trinity	XLOC_056347	c60000_g1_i1:1-1402	cdRNA04-Dia-R3	cdRNA05-postDia	7.1247	42.0195	2.56016	5.00E-05	0.0249845
RNA-Seq	Trinity	XLOC_056463	c60109_g1_i1:0-592	cdRNA04-Dia-R3	cdRNA05-postDia	9.08489	54.9539	2.59668	5.00E-05	0.0249845
RNA-Seq	Trinity	XLOC_056463	c60109_g1_i1:0-592	cdRNA03-Dia-R2	cdRNA05-postDia	8.947	54.9539	2.61875	5.00E-05	0.0249845
RNA-Seq	Trinity	XLOC_056464	c6010_g1_i1:0-354	cdRNA02-Dia-R1	cdRNA05-postDia	6.12201	48.6653	2.99081	5.00E-05	0.0249845

RNA-Seq	Trinity	XLOC_056743	c6036_g1_i1:0-562	cdRNA03-Dia-R2	cdRNA05-postDia	8.37444	59.3362	2.82485	5.00E-05	0.0249845
RNA-Seq	Trinity	XLOC_056743	c6036_g1_i1:0-562	cdRNA04-Dia-R3	cdRNA05-postDia	8.25808	59.3362	2.84503	5.00E-05	0.0249845
RNA-Seq	Trinity	XLOC_056836	c60456_g1_i1:0-742	cdRNA01-preDia	cdRNA03-Dia-R2	3168.85	169.306	-4.22626	5.00E-05	0.0249845
RNA-Seq	Trinity	XLOC_056836	c60456_g1_i1:0-742	cdRNA01-preDia	cdRNA04-Dia-R3	3168.85	176.27	-4.1681	5.00E-05	0.0249845
RNA-Seq	Trinity	XLOC_056868	c60487_g1_i1:0-885	cdRNA01-preDia	cdRNA04-Dia-R3	55.4118	9.6845	-2.51645	0.0001	0.0432976
RNA-Seq	Trinity	XLOC_056868	c60487_g1_i1:0-885	cdRNA01-preDia	cdRNA03-Dia-R2	55.4118	10.8325	-2.35483	0.0001	0.0432976
RNA-Seq	Trinity	XLOC_056868	c60487_g1_i1:0-885	cdRNA03-Dia-R2	cdRNA05-postDia	10.8325	64.3766	2.57118	0.0001	0.0432976
RNA-Seq	Trinity	XLOC_056900	c60517_g1_i1:10-1823	cdRNA01-preDia	cdRNA05-postDia	58.743	11.9145	-2.3017	0.0001	0.0432976
RNA-Seq	Trinity	XLOC_056900	c60517_g1_i1:10-1823	cdRNA04-Dia-R3	cdRNA05-postDia	58.7045	11.9145	-2.30075	0.0001	0.0432976
RNA-Seq	Trinity	XLOC_057073	c6067_g1_i1:0-1510	cdRNA01-preDia	cdRNA05-postDia	7.80597	47.1795	2.59551	5.00E-05	0.0249845
RNA-Seq	Trinity	XLOC_057148	c60749_g1_i1:0-826	cdRNA04-Dia-R3	cdRNA05-postDia	7.0078	37.8045	2.43152	5.00E-05	0.0249845
RNA-Seq	Trinity	XLOC_057148	c60749_g1_i1:0-826	cdRNA03-Dia-R2	cdRNA05-postDia	6.21538	37.8045	2.60464	5.00E-05	0.0249845
RNA-Seq	Trinity	XLOC_057160	c60760_g1_i1:0-548	cdRNA01-preDia	cdRNA05-postDia	11.0268	67.312	2.60984	5.00E-05	0.0249845
RNA-Seq	Trinity	XLOC_057259	c60852_g1_i1:0-842	cdRNA01-preDia	cdRNA04-Dia-R3	14.6782	2.67341	-2.45692	0.0001	0.0432976
RNA-Seq	Trinity	XLOC_057941	c614_g1_i1:0-1054	cdRNA04-Dia-R3	cdRNA05-postDia	21.1812	3.28219	-2.69006	5.00E-05	0.0249845
RNA-Seq	Trinity	XLOC_057941	c614_g1_i1:0-1054	cdRNA03-Dia-R2	cdRNA05-postDia	20.0029	3.28219	-2.60748	5.00E-05	0.0249845
RNA-Seq	Trinity	XLOC_058031	c61587_g1_i1:0-592	cdRNA02-Dia-R1	cdRNA05-postDia	10.4307	100.047	3.26176	5.00E-05	0.0249845
RNA-Seq	Trinity	XLOC_058031	c61587_g1_i1:0-592	cdRNA01-preDia	cdRNA05-postDia	3.15697	100.047	4.98599	5.00E-05	0.0249845
RNA-Seq	Trinity	XLOC_058031	c61587_g1_i1:0-592	cdRNA04-Dia-R3	cdRNA05-postDia	1.75837	100.047	5.8303	5.00E-05	0.0249845
RNA-Seq	Trinity	XLOC_058031	c61587_g1_i1:0-592	cdRNA03-Dia-R2	cdRNA05-postDia	1.22004	100.047	6.3576	5.00E-05	0.0249845
RNA-Seq	Trinity	XLOC_058114	c61668_g1_i1:0-750	cdRNA01-preDia	cdRNA05-postDia	4.4145	26.4239	2.58152	0.0001	0.0432976
RNA-Seq	Trinity	XLOC_058223	c6176_g1_i1:0-1229	cdRNA01-preDia	cdRNA05-postDia	2.12025	14.6201	2.78564	5.00E-05	0.0249845
RNA-Seq	Trinity	XLOC_058278	c61822_g1_i1:0-641	cdRNA04-Dia-R3	cdRNA05-postDia	9.36726	43.5026	2.2154	0.0001	0.0432976
RNA-Seq	Trinity	XLOC_058278	c61822_g1_i1:0-641	cdRNA03-Dia-R2	cdRNA05-postDia	7.673	43.5026	2.50324	5.00E-05	0.0249845
RNA-Seq	Trinity	XLOC_058289	c61832_g1_i1:0-434	cdRNA01-preDia	cdRNA04-Dia-R3	10.651	93.106	3.12788	5.00E-05	0.0249845
RNA-Seq	Trinity	XLOC_058315	c61855_g1_i1:0-694	cdRNA04-Dia-R3	cdRNA05-postDia	2.62999	17.9753	2.77288	5.00E-05	0.0249845
RNA-Seq	Trinity	XLOC_058315	c61855_g1_i1:0-694	cdRNA03-Dia-R2	cdRNA05-postDia	2.25419	17.9753	2.99533	5.00E-05	0.0249845
RNA-Seq	Trinity	XLOC_058506	c62037_g1_i1:0-2889	cdRNA04-Dia-R3	cdRNA05-postDia	90.6582	10.3758	-3.12721	5.00E-05	0.0249845
RNA-Seq	Trinity	XLOC_058506	c62037_g1_i1:0-2889	cdRNA03-Dia-R2	cdRNA05-postDia	90.5055	10.3758	-3.12478	5.00E-05	0.0249845
RNA-Seq	Trinity	XLOC_058506	c62037_g1_i1:0-2889	cdRNA01-preDia	cdRNA03-Dia-R2	7.03455	90.5055	3.68548	5.00E-05	0.0249845
RNA-Seq	Trinity	XLOC_058506	c62037_g1_i1:0-2889	cdRNA01-preDia	cdRNA04-Dia-R3	7.03455	90.6582	3.68791	5.00E-05	0.0249845
RNA-Seq	Trinity	XLOC_058640	c62160_g1_i1:7-449	cdRNA03-Dia-R2	cdRNA05-postDia	141.215	813.482	2.52622	5.00E-05	0.0249845
RNA-Seq	Trinity	XLOC_058640	c62160_g1_i1:7-449	cdRNA01-preDia	cdRNA05-postDia	136.466	813.482	2.57556	5.00E-05	0.0249845
RNA-Seq	Trinity	XLOC_058640	c62160_g1_i1:7-449	cdRNA04-Dia-R3	cdRNA05-postDia	132.815	813.482	2.61469	5.00E-05	0.0249845
RNA-Seq	Trinity	XLOC_058663	c62182_g1_i1:0-662	cdRNA04-Dia-R3	cdRNA05-postDia	58.9231	330.671	2.48849	5.00E-05	0.0249845
RNA-Seq	Trinity	XLOC_058663	c62182_g1_i1:0-662	cdRNA01-preDia	cdRNA05-postDia	58.1483	330.671	2.50759	0.0001	0.0432976
RNA-Seq	Trinity	XLOC_058663	c62182_g1_i1:0-662	cdRNA03-Dia-R2	cdRNA05-postDia	54.0046	330.671	2.61425	5.00E-05	0.0249845
RNA-Seq	Trinity	XLOC_058680	c62199_g1_i1:0-480	cdRNA01-preDia	cdRNA04-Dia-R3	59.6982	7.27829	-3.03601	5.00E-05	0.0249845
RNA-Seq	Trinity	XLOC_058680	c62199_g1_i1:0-480	cdRNA01-preDia	cdRNA03-Dia-R2	59.6982	8.28971	-2.84829	0.0001	0.0432976
RNA-Seq	Trinity	XLOC_058716	c62234_g1_i1:0-369	cdRNA01-preDia	cdRNA05-postDia	5.73421	45.7881	2.99731	5.00E-05	0.0249845
RNA-Seq	Trinity	XLOC_058742	c6225_g1_i1:0-1169	cdRNA01-preDia	cdRNA03-Dia-R2	52.6168	334.151	2.6669	5.00E-05	0.0249845
RNA-Seq	Trinity	XLOC_058857	c62366_g1_i1:0-1569	cdRNA03-Dia-R2	cdRNA05-postDia	2.22533	15.7378	2.82214	5.00E-05	0.0249845
RNA-Seq	Trinity	XLOC_058857	c62366_g1_i1:0-1569	cdRNA04-Dia-R3	cdRNA05-postDia	2.22145	15.7378	2.82466	5.00E-05	0.0249845
RNA-Seq	Trinity	XLOC_058898	c62404_g1_i1:2-468	cdRNA01-preDia	cdRNA05-postDia	3.46864	37.7242	3.44305	5.00E-05	0.0249845
RNA-Seq	Trinity	XLOC_058898	c62404_g1_i1:2-468	cdRNA01-preDia	cdRNA04-Dia-R3	3.46864	39.9273	3.52493	5.00E-05	0.0249845
RNA-Seq	Trinity	XLOC_058898	c62404_g1_i1:2-468	cdRNA01-preDia	cdRNA02-Dia-R1	3.46864	39.98	3.52683	5.00E-05	0.0249845
RNA-Seq	Trinity	XLOC_058898	c62404_g1_i1:2-468	cdRNA01-preDia	cdRNA03-Dia-R2	3.46864	43.2116	3.63897	5.00E-05	0.0249845

RNA-Seq	Trinity	XLOC_058902	c62408_g1_i1:0-909	cdRNA03-Dia-R2	cdRNA05-postDia	1.67867	11.476	2.77323	0.0001	0.0432976
RNA-Seq	Trinity	XLOC_059112	c62603_g1_i1:2-1258	cdRNA01-preDia	cdRNA05-postDia	2.59169	14.3585	2.46994	5.00E-05	0.0249845
RNA-Seq	Trinity	XLOC_059176	c62668_g1_i1:0-2050	cdRNA01-preDia	cdRNA04-Dia-R3	8.11258	56.2804	2.7944	5.00E-05	0.0249845
RNA-Seq	Trinity	XLOC_059176	c62668_g1_i1:0-2050	cdRNA01-preDia	cdRNA03-Dia-R2	8.11258	57.1436	2.81636	5.00E-05	0.0249845
RNA-Seq	Trinity	XLOC_059660	c63130_g1_i1:0-488	cdRNA04-Dia-R3	cdRNA05-postDia	2.13533	32.1357	3.91165	5.00E-05	0.0249845
RNA-Seq	Trinity	XLOC_060172	c63605_g1_i1:0-991	cdRNA01-preDia	cdRNA05-postDia	2.20915	17.9272	3.02059	5.00E-05	0.0249845
RNA-Seq	Trinity	XLOC_060221	c63652_g1_i1:0-408	cdRNA04-Dia-R3	cdRNA05-postDia	8.73039	56.2517	2.68778	5.00E-05	0.0249845
RNA-Seq	Trinity	XLOC_060221	c63652_g1_i1:0-408	cdRNA03-Dia-R2	cdRNA05-postDia	8.31308	56.2517	2.75844	5.00E-05	0.0249845
RNA-Seq	Trinity	XLOC_060301	c63730_g1_i1:0-302	cdRNA04-Dia-R3	cdRNA05-postDia	391.907	54.5008	-2.84616	5.00E-05	0.0249845
RNA-Seq	Trinity	XLOC_060301	c63730_g1_i1:0-302	cdRNA03-Dia-R2	cdRNA05-postDia	352.39	54.5008	-2.69282	5.00E-05	0.0249845
RNA-Seq	Trinity	XLOC_060301	c63730_g1_i1:0-302	cdRNA01-preDia	cdRNA05-postDia	305.156	54.5008	-2.4852	0.0001	0.0432976
RNA-Seq	Trinity	XLOC_060376	c637_g1_i1:0-842	cdRNA04-Dia-R3	cdRNA05-postDia	37.309	4.06046	-3.19981	5.00E-05	0.0249845
RNA-Seq	Trinity	XLOC_060376	c637_g1_i1:0-842	cdRNA03-Dia-R2	cdRNA05-postDia	29.5556	4.06046	-2.86372	5.00E-05	0.0249845
RNA-Seq	Trinity	XLOC_060376	c637_g1_i1:0-842	cdRNA02-Dia-R1	cdRNA05-postDia	24.8611	4.06046	-2.61418	5.00E-05	0.0249845
RNA-Seq	Trinity	XLOC_060521	c63938_g1_i1:0-795	cdRNA02-Dia-R1	cdRNA05-postDia	11.7826	109.651	3.2182	5.00E-05	0.0249845
RNA-Seq	Trinity	XLOC_060521	c63938_g1_i1:0-795	cdRNA04-Dia-R3	cdRNA05-postDia	6.92644	109.651	3.98467	5.00E-05	0.0249845
RNA-Seq	Trinity	XLOC_060521	c63938_g1_i1:0-795	cdRNA03-Dia-R2	cdRNA05-postDia	5.94065	109.651	4.20616	5.00E-05	0.0249845
RNA-Seq	Trinity	XLOC_060521	c63938_g1_i1:0-795	cdRNA01-preDia	cdRNA05-postDia	3.94466	109.651	4.79688	5.00E-05	0.0249845
RNA-Seq	Trinity	XLOC_060563	c63980_g1_i1:0-543	cdRNA04-Dia-R3	cdRNA05-postDia	13.5286	69.1277	2.35325	0.0001	0.0432976
RNA-Seq	Trinity	XLOC_060688	c64095_g1_i1:0-299	cdRNA02-Dia-R1	cdRNA05-postDia	51.4226	402.339	2.96794	5.00E-05	0.0249845
RNA-Seq	Trinity	XLOC_060688	c64095_g1_i1:0-299	cdRNA04-Dia-R3	cdRNA05-postDia	47.1112	402.339	3.09427	5.00E-05	0.0249845
RNA-Seq	Trinity	XLOC_060688	c64095_g1_i1:0-299	cdRNA03-Dia-R2	cdRNA05-postDia	37.8704	402.339	3.40927	5.00E-05	0.0249845
RNA-Seq	Trinity	XLOC_060699	c64103_g2_i1:2-424	cdRNA01-preDia	cdRNA04-Dia-R3	44.9472	4.694	-3.25934	5.00E-05	0.0249845
RNA-Seq	Trinity	XLOC_060699	c64103_g2_i1:2-424	cdRNA01-preDia	cdRNA03-Dia-R2	44.9472	5.06635	-3.14921	5.00E-05	0.0249845
RNA-Seq	Trinity	XLOC_060883	c64273_g1_i1:0-261	cdRNA02-Dia-R1	cdRNA05-postDia	43.9117	385.53	3.13417	5.00E-05	0.0249845
RNA-Seq	Trinity	XLOC_060883	c64273_g1_i1:0-261	cdRNA04-Dia-R3	cdRNA05-postDia	15.8184	385.53	4.60716	5.00E-05	0.0249845
RNA-Seq	Trinity	XLOC_060883	c64273_g1_i1:0-261	cdRNA01-preDia	cdRNA05-postDia	12.9569	385.53	4.89505	5.00E-05	0.0249845
RNA-Seq	Trinity	XLOC_060883	c64273_g1_i1:0-261	cdRNA03-Dia-R2	cdRNA05-postDia	9.40769	385.53	5.35686	5.00E-05	0.0249845
RNA-Seq	Trinity	XLOC_061316	c64687_g1_i1:0-507	cdRNA03-Dia-R2	cdRNA05-postDia	13.0191	69.4486	2.41532	5.00E-05	0.0249845
RNA-Seq	Trinity	XLOC_061316	c64687_g1_i1:0-507	cdRNA02-Dia-R1	cdRNA05-postDia	12.4728	69.4486	2.47716	0.0001	0.0432976
RNA-Seq	Trinity	XLOC_061514	c64880_g1_i1:0-609	cdRNA02-Dia-R1	cdRNA05-postDia	6.75992	44.4796	2.71807	0.0001	0.0432976
RNA-Seq	Trinity	XLOC_061590	c64951_g1_i1:3-419	cdRNA04-Dia-R3	cdRNA05-postDia	16.8037	127.631	2.92512	5.00E-05	0.0249845
RNA-Seq	Trinity	XLOC_061590	c64951_g1_i1:3-419	cdRNA03-Dia-R2	cdRNA05-postDia	16.0005	127.631	2.99579	5.00E-05	0.0249845
RNA-Seq	Trinity	XLOC_061590	c64951_g1_i1:3-419	cdRNA02-Dia-R1	cdRNA05-postDia	10.5264	127.631	3.5999	5.00E-05	0.0249845
RNA-Seq	Trinity	XLOC_061590	c64951_g1_i1:3-419	cdRNA01-preDia	cdRNA05-postDia	2.98174	127.631	5.41968	5.00E-05	0.0249845
RNA-Seq	Trinity	XLOC_061719	c65076_g1_i1:1-1480	cdRNA02-Dia-R1	cdRNA05-postDia	8.9627	52.9216	2.56185	5.00E-05	0.0249845
RNA-Seq	Trinity	XLOC_061719	c65076_g1_i1:1-1480	cdRNA04-Dia-R3	cdRNA05-postDia	8.14553	52.9216	2.69978	5.00E-05	0.0249845
RNA-Seq	Trinity	XLOC_061719	c65076_g1_i1:1-1480	cdRNA03-Dia-R2	cdRNA05-postDia	7.5804	52.9216	2.80351	5.00E-05	0.0249845
RNA-Seq	Trinity	XLOC_061728	c65085_g1_i1:0-609	cdRNA01-preDia	cdRNA03-Dia-R2	27.7079	134.708	2.28147	5.00E-05	0.0249845
RNA-Seq	Trinity	XLOC_061766	c65124_g1_i1:0-285	cdRNA01-preDia	cdRNA05-postDia	7.74666	151.676	4.29127	0.0001	0.0432976
RNA-Seq	Trinity	XLOC_061809	c65168_g1_i1:0-1103	cdRNA01-preDia	cdRNA05-postDia	10.9883	51.293	2.2228	0.0001	0.0432976
RNA-Seq	Trinity	XLOC_061809	c65168_g1_i1:0-1103	cdRNA02-Dia-R1	cdRNA05-postDia	7.6797	51.293	2.73964	5.00E-05	0.0249845
RNA-Seq	Trinity	XLOC_061826	c65184_g1_i1:0-1112	cdRNA04-Dia-R3	cdRNA05-postDia	7.01294	39.4162	2.4907	5.00E-05	0.0249845
RNA-Seq	Trinity	XLOC_061826	c65184_g1_i1:0-1112	cdRNA01-preDia	cdRNA05-postDia	6.74519	39.4162	2.54686	5.00E-05	0.0249845
RNA-Seq	Trinity	XLOC_061826	c65184_g1_i1:0-1112	cdRNA02-Dia-R1	cdRNA05-postDia	4.35895	39.4162	3.17674	5.00E-05	0.0249845
RNA-Seq	Trinity	XLOC_062052	c65407_g1_i1:0-509	cdRNA01-preDia	cdRNA05-postDia	4.66379	43.1208	3.20881	5.00E-05	0.0249845
RNA-Seq	Trinity	XLOC_062166	c65516_g1_i1:0-1252	cdRNA03-Dia-R2	cdRNA05-postDia	1.76785	10.0509	2.50725	0.0001	0.0432976

RNA-Seq	Trinity	XLOC_062166	c65516_g1_i1:0-1252	cdRNA04-Dia-R3	cdRNA05-postDia	1.57454	10.0509	2.67432	5.00E-05	0.0249845
RNA-Seq	Trinity	XLOC_062359	c65707_g1_i1:0-491	cdRNA01-preDia	cdRNA03-Dia-R2	7.57209	46.0452	2.60429	5.00E-05	0.0249845
RNA-Seq	Trinity	XLOC_062359	c65707_g1_i1:0-491	cdRNA01-preDia	cdRNA04-Dia-R3	7.57209	47.9389	2.66243	5.00E-05	0.0249845
RNA-Seq	Trinity	XLOC_062679	c66014_g1_i1:5-232	cdRNA03-Dia-R2	cdRNA05-postDia	60.3413	374.09	2.63217	5.00E-05	0.0249845
RNA-Seq	Trinity	XLOC_062679	c66014_g1_i1:5-232	cdRNA04-Dia-R3	cdRNA05-postDia	51.8281	374.09	2.85158	5.00E-05	0.0249845
RNA-Seq	Trinity	XLOC_062679	c66014_g1_i1:5-232	cdRNA01-preDia	cdRNA05-postDia	26.5572	374.09	3.81621	5.00E-05	0.0249845
RNA-Seq	Trinity	XLOC_062798	c6612_g1_i1:0-806	cdRNA02-Dia-R1	cdRNA05-postDia	5.93195	28.9974	2.28934	5.00E-05	0.0249845
RNA-Seq	Trinity	XLOC_063066	c66389_g1_i1:0-628	cdRNA03-Dia-R2	cdRNA05-postDia	1.9538	19.8333	3.34356	5.00E-05	0.0249845
RNA-Seq	Trinity	XLOC_063066	c66389_g1_i1:0-628	cdRNA04-Dia-R3	cdRNA05-postDia	1.51968	19.8333	3.70608	0.0001	0.0432976
RNA-Seq	Trinity	XLOC_063472	c66788_g1_i1:0-521	cdRNA04-Dia-R3	cdRNA05-postDia	49.6197	9.30348	-2.41507	5.00E-05	0.0249845
RNA-Seq	Trinity	XLOC_063472	c66788_g1_i1:0-521	cdRNA03-Dia-R2	cdRNA05-postDia	46.2921	9.30348	-2.31492	5.00E-05	0.0249845
RNA-Seq	Trinity	XLOC_063472	c66788_g1_i1:0-521	cdRNA01-preDia	cdRNA03-Dia-R2	1.6204	46.2921	4.83635	5.00E-05	0.0249845
RNA-Seq	Trinity	XLOC_063472	c66788_g1_i1:0-521	cdRNA01-preDia	cdRNA04-Dia-R3	1.6204	49.6197	4.9365	5.00E-05	0.0249845
RNA-Seq	Trinity	XLOC_064067	c67350_g1_i1:0-272	cdRNA01-preDia	cdRNA05-postDia	17.1925	107.199	2.64044	0.0001	0.0432976
RNA-Seq	Trinity	XLOC_064113	c67396_g1_i1:0-602	cdRNA01-preDia	cdRNA05-postDia	11.4171	61.9847	2.44072	5.00E-05	0.0249845
RNA-Seq	Trinity	XLOC_064113	c67396_g1_i1:0-602	cdRNA03-Dia-R2	cdRNA05-postDia	5.05491	61.9847	3.61615	5.00E-05	0.0249845
RNA-Seq	Trinity	XLOC_064113	c67396_g1_i1:0-602	cdRNA04-Dia-R3	cdRNA05-postDia	4.28547	61.9847	3.85439	5.00E-05	0.0249845
RNA-Seq	Trinity	XLOC_064113	c67396_g1_i1:0-602	cdRNA02-Dia-R1	cdRNA05-postDia	4.03756	61.9847	3.94036	5.00E-05	0.0249845
RNA-Seq	Trinity	XLOC_064147	c6742_g1_i1:4-437	cdRNA01-preDia	cdRNA05-postDia	20.3598	212.841	3.38598	5.00E-05	0.0249845
RNA-Seq	Trinity	XLOC_064147	c6742_g1_i1:4-437	cdRNA03-Dia-R2	cdRNA05-postDia	16.5367	212.841	3.68603	5.00E-05	0.0249845
RNA-Seq	Trinity	XLOC_064147	c6742_g1_i1:4-437	cdRNA04-Dia-R3	cdRNA05-postDia	16.2198	212.841	3.71394	5.00E-05	0.0249845
RNA-Seq	Trinity	XLOC_064147	c6742_g1_i1:4-437	cdRNA02-Dia-R1	cdRNA05-postDia	12.2148	212.841	4.12307	5.00E-05	0.0249845
RNA-Seq	Trinity	XLOC_064297	c67572_g1_i1:0-732	cdRNA03-Dia-R2	cdRNA05-postDia	3.13795	18.2668	2.54133	5.00E-05	0.0249845
RNA-Seq	Trinity	XLOC_064922	c6816_g1_i1:0-964	cdRNA01-preDia	cdRNA04-Dia-R3	18.353	3.44121	-2.41503	5.00E-05	0.0249845
RNA-Seq	Trinity	XLOC_065184	c68414_g1_i1:9-234	cdRNA03-Dia-R2	cdRNA05-postDia	21.6525	201.635	3.21914	5.00E-05	0.0249845
RNA-Seq	Trinity	XLOC_065371	c68606_g1_i1:0-459	cdRNA04-Dia-R3	cdRNA05-postDia	108.355	13.9097	-2.9616	5.00E-05	0.0249845
RNA-Seq	Trinity	XLOC_065371	c68606_g1_i1:0-459	cdRNA03-Dia-R2	cdRNA05-postDia	106.895	13.9097	-2.94203	5.00E-05	0.0249845
RNA-Seq	Trinity	XLOC_066169	c69395_g1_i1:0-483	cdRNA04-Dia-R3	cdRNA05-postDia	9.64533	55.8526	2.53372	5.00E-05	0.0249845
RNA-Seq	Trinity	XLOC_066169	c69395_g1_i1:0-483	cdRNA02-Dia-R1	cdRNA05-postDia	7.5728	55.8526	2.88273	5.00E-05	0.0249845
RNA-Seq	Trinity	XLOC_066655	c69865_g1_i1:1-451	cdRNA01-preDia	cdRNA04-Dia-R3	13.6526	91.6732	2.74732	5.00E-05	0.0249845
RNA-Seq	Trinity	XLOC_066655	c69865_g1_i1:1-451	cdRNA01-preDia	cdRNA03-Dia-R2	13.6526	92.2042	2.75566	5.00E-05	0.0249845
RNA-Seq	Trinity	XLOC_066945	c70142_g1_i1:1-228	cdRNA03-Dia-R2	cdRNA05-postDia	31.9992	206.904	2.69285	5.00E-05	0.0249845
RNA-Seq	Trinity	XLOC_067125	c7031_g1_i1:0-1102	cdRNA01-preDia	cdRNA05-postDia	1.80408	11.9388	2.72632	0.0001	0.0432976
RNA-Seq	Trinity	XLOC_067820	c70998_g1_i1:0-214	cdRNA02-Dia-R1	cdRNA05-postDia	50.4224	337.646	2.74338	5.00E-05	0.0249845
RNA-Seq	Trinity	XLOC_067820	c70998_g1_i1:0-214	cdRNA04-Dia-R3	cdRNA05-postDia	35.3221	337.646	3.25687	5.00E-05	0.0249845
RNA-Seq	Trinity	XLOC_067820	c70998_g1_i1:0-214	cdRNA03-Dia-R2	cdRNA05-postDia	28.9644	337.646	3.54316	5.00E-05	0.0249845
RNA-Seq	Trinity	XLOC_068132	c71307_g1_i1:0-236	cdRNA01-preDia	cdRNA05-postDia	36.2991	224.558	2.62908	5.00E-05	0.0249845
RNA-Seq	Trinity	XLOC_068132	c71307_g1_i1:0-236	cdRNA04-Dia-R3	cdRNA05-postDia	34.8197	224.558	2.68912	0.0001	0.0432976
RNA-Seq	Trinity	XLOC_068132	c71307_g1_i1:0-236	cdRNA02-Dia-R1	cdRNA05-postDia	34.0204	224.558	2.72262	5.00E-05	0.0249845
RNA-Seq	Trinity	XLOC_068520	c71699_g1_i1:0-458	cdRNA01-preDia	cdRNA03-Dia-R2	78.4024	9.93951	-2.97965	5.00E-05	0.0249845
RNA-Seq	Trinity	XLOC_068520	c71699_g1_i1:0-458	cdRNA01-preDia	cdRNA04-Dia-R3	78.4024	11.6392	-2.75191	5.00E-05	0.0249845
RNA-Seq	Trinity	XLOC_068520	c71699_g1_i1:0-458	cdRNA04-Dia-R3	cdRNA05-postDia	11.6392	72.3329	2.63566	5.00E-05	0.0249845
RNA-Seq	Trinity	XLOC_068610	c71790_g1_i1:0-449	cdRNA03-Dia-R2	cdRNA05-postDia	4.83029	64.0912	3.72994	5.00E-05	0.0249845
RNA-Seq	Trinity	XLOC_068610	c71790_g1_i1:0-449	cdRNA04-Dia-R3	cdRNA05-postDia	3.71283	64.0912	4.10953	5.00E-05	0.0249845
RNA-Seq	Trinity	XLOC_068610	c71790_g1_i1:0-449	cdRNA02-Dia-R1	cdRNA05-postDia	2.18628	64.0912	4.87358	5.00E-05	0.0249845
RNA-Seq	Trinity	XLOC_068835	c72018_g1_i1:0-215	cdRNA02-Dia-R1	cdRNA05-postDia	31.4325	726.72	4.53107	5.00E-05	0.0249845
RNA-Seq	Trinity	XLOC_068907	c7208_g1_i1:0-1536	cdRNA02-Dia-R1	cdRNA05-postDia	25.9609	170.941	2.71909	5.00E-05	0.0249845

RNA-Seq	Trinity	XLOC_069209	c7238_g1_i1:0-694	cdRNA01-preDia	cdRNA05-postDia	6.28987	43.3521	2.785	5.00E-05	0.0249845
RNA-Seq	Trinity	XLOC_069354	c7253_g1_i1:0-442	cdRNA04-Dia-R3	cdRNA05-postDia	7.64406	41.1932	2.43	5.00E-05	0.0249845
RNA-Seq	Trinity	XLOC_069610	c72809_g1_i1:0-204	cdRNA02-Dia-R1	cdRNA05-postDia	68.358	1430.3	4.38707	5.00E-05	0.0249845
RNA-Seq	Trinity	XLOC_069610	c72809_g1_i1:0-204	cdRNA04-Dia-R3	cdRNA05-postDia	20.1893	1430.3	6.14659	5.00E-05	0.0249845
RNA-Seq	Trinity	XLOC_069661	c7285_g1_i1:0-2317	cdRNA01-preDia	cdRNA05-postDia	10.0608	68.0731	2.75833	5.00E-05	0.0249845
RNA-Seq	Trinity	XLOC_069661	c7285_g1_i1:0-2317	cdRNA02-Dia-R1	cdRNA05-postDia	5.14059	68.0731	3.72708	5.00E-05	0.0249845
RNA-Seq	Trinity	XLOC_069661	c7285_g1_i1:0-2317	cdRNA03-Dia-R2	cdRNA05-postDia	2.87514	68.0731	4.56538	5.00E-05	0.0249845
RNA-Seq	Trinity	XLOC_069661	c7285_g1_i1:0-2317	cdRNA04-Dia-R3	cdRNA05-postDia	2.45755	68.0731	4.7918	5.00E-05	0.0249845
RNA-Seq	Trinity	XLOC_069671	c7286_g1_i1:0-921	cdRNA01-preDia	cdRNA05-postDia	430.02	5.33624	-6.33244	5.00E-05	0.0249845
RNA-Seq	Trinity	XLOC_069671	c7286_g1_i1:0-921	cdRNA02-Dia-R1	cdRNA05-postDia	91.559	5.33624	-4.10081	5.00E-05	0.0249845
RNA-Seq	Trinity	XLOC_069671	c7286_g1_i1:0-921	cdRNA03-Dia-R2	cdRNA05-postDia	82.3086	5.33624	-3.94715	5.00E-05	0.0249845
RNA-Seq	Trinity	XLOC_069671	c7286_g1_i1:0-921	cdRNA04-Dia-R3	cdRNA05-postDia	77.5508	5.33624	-3.86125	5.00E-05	0.0249845
RNA-Seq	Trinity	XLOC_069872	c73082_g1_i1:0-202	cdRNA02-Dia-R1	cdRNA05-postDia	30.7584	308.954	3.32834	5.00E-05	0.0249845
RNA-Seq	Trinity	XLOC_069978	c7319_g1_i1:5-1581	cdRNA01-preDia	cdRNA04-Dia-R3	15.5521	2.90078	-2.4226	5.00E-05	0.0249845
RNA-Seq	Trinity	XLOC_069978	c7319_g1_i1:5-1581	cdRNA01-preDia	cdRNA03-Dia-R2	15.5521	3.01906	-2.36494	5.00E-05	0.0249845
RNA-Seq	Trinity	XLOC_070223	c7345_g1_i1:0-1423	cdRNA03-Dia-R2	cdRNA05-postDia	13.9479	75.2314	2.43128	5.00E-05	0.0249845
RNA-Seq	Trinity	XLOC_070223	c7345_g1_i1:0-1423	cdRNA04-Dia-R3	cdRNA05-postDia	13.5258	75.2314	2.47562	5.00E-05	0.0249845
RNA-Seq	Trinity	XLOC_070223	c7345_g1_i1:0-1423	cdRNA01-preDia	cdRNA05-postDia	9.04379	75.2314	3.05634	5.00E-05	0.0249845
RNA-Seq	Trinity	XLOC_070501	c7376_g1_i1:0-584	cdRNA01-preDia	cdRNA05-postDia	1.76769	26.22	3.89073	0.0001	0.0432976
RNA-Seq	Trinity	XLOC_070552	c7382_g1_i1:0-392	cdRNA02-Dia-R1	cdRNA05-postDia	116.105	521.359	2.16684	0.0001	0.0432976
RNA-Seq	Trinity	XLOC_070836	c74125_g1_i1:0-1772	cdRNA01-preDia	cdRNA02-Dia-R1	27.9975	242.418	3.11413	5.00E-05	0.0249845
RNA-Seq	Trinity	XLOC_070836	c74125_g1_i1:0-1772	cdRNA01-preDia	cdRNA05-postDia	27.9975	627.494	4.48623	5.00E-05	0.0249845
RNA-Seq	Trinity	XLOC_070839	c74128_g1_i1:0-958	cdRNA01-preDia	cdRNA05-postDia	3.9304	20.139	2.35724	5.00E-05	0.0249845
RNA-Seq	Trinity	XLOC_070839	c74128_g1_i1:0-958	cdRNA04-Dia-R3	cdRNA05-postDia	3.66967	20.139	2.45627	5.00E-05	0.0249845
RNA-Seq	Trinity	XLOC_070839	c74128_g1_i1:0-958	cdRNA02-Dia-R1	cdRNA05-postDia	3.11796	20.139	2.69131	5.00E-05	0.0249845
RNA-Seq	Trinity	XLOC_070923	c74206_g1_i1:0-2094	cdRNA01-preDia	cdRNA03-Dia-R2	3.55417	17.5538	2.3042	5.00E-05	0.0249845
RNA-Seq	Trinity	XLOC_070923	c74206_g1_i1:0-2094	cdRNA01-preDia	cdRNA04-Dia-R3	3.55417	18.7512	2.3994	5.00E-05	0.0249845
RNA-Seq	Trinity	XLOC_071009	c74283_g1_i1:0-1131	cdRNA03-Dia-R2	cdRNA05-postDia	11.7453	57.3106	2.28672	0.0001	0.0432976
RNA-Seq	Trinity	XLOC_071009	c74283_g1_i1:0-1131	cdRNA02-Dia-R1	cdRNA05-postDia	8.02558	57.3106	2.83613	5.00E-05	0.0249845
RNA-Seq	Trinity	XLOC_071211	c74472_g1_i1:0-762	cdRNA04-Dia-R3	cdRNA05-postDia	2.3095	13.3564	2.53188	0.0001	0.0432976
RNA-Seq	Trinity	XLOC_071246	c74503_g1_i1:0-608	cdRNA01-preDia	cdRNA03-Dia-R2	14.8663	97.4487	2.7126	0.0001	0.0432976
RNA-Seq	Trinity	XLOC_071246	c74503_g1_i1:0-608	cdRNA01-preDia	cdRNA04-Dia-R3	14.8663	106.39	2.83925	5.00E-05	0.0249845
RNA-Seq	Trinity	XLOC_071590	c74821_g1_i1:0-480	cdRNA04-Dia-R3	cdRNA05-postDia	78.0013	12.9958	-2.58545	5.00E-05	0.0249845
RNA-Seq	Trinity	XLOC_071590	c74821_g1_i1:0-480	cdRNA03-Dia-R2	cdRNA05-postDia	69.605	12.9958	-2.42114	5.00E-05	0.0249845
RNA-Seq	Trinity	XLOC_071595	c74826_g1_i1:0-591	cdRNA03-Dia-R2	cdRNA05-postDia	15.4938	95.8771	2.62949	5.00E-05	0.0249845
RNA-Seq	Trinity	XLOC_071640	c74867_g1_i1:0-1355	cdRNA01-preDia	cdRNA05-postDia	5.08085	24.3425	2.26034	5.00E-05	0.0249845
RNA-Seq	Trinity	XLOC_071640	c74867_g1_i1:0-1355	cdRNA02-Dia-R1	cdRNA05-postDia	3.7231	24.3425	2.7089	5.00E-05	0.0249845
RNA-Seq	Trinity	XLOC_071640	c74867_g1_i1:0-1355	cdRNA03-Dia-R2	cdRNA05-postDia	3.66728	24.3425	2.7307	5.00E-05	0.0249845
RNA-Seq	Trinity	XLOC_071640	c74867_g1_i1:0-1355	cdRNA04-Dia-R3	cdRNA05-postDia	3.16136	24.3425	2.94486	5.00E-05	0.0249845
RNA-Seq	Trinity	XLOC_071659	c74884_g1_i1:0-4504	cdRNA04-Dia-R3	cdRNA05-postDia	4.98037	29.3078	2.55696	0.0001	0.0432976
RNA-Seq	Trinity	XLOC_071877	c75081_g1_i1:3-953	cdRNA01-preDia	cdRNA05-postDia	369.447	40.977	-3.17248	5.00E-05	0.0249845
RNA-Seq	Trinity	XLOC_071923	c75122_g1_i1:21-1058	cdRNA03-Dia-R2	cdRNA05-postDia	28.7934	5.88497	-2.29063	5.00E-05	0.0249845
RNA-Seq	Trinity	XLOC_071923	c75122_g1_i1:21-1058	cdRNA01-preDia	cdRNA03-Dia-R2	3.22561	28.7934	3.15809	5.00E-05	0.0249845
RNA-Seq	Trinity	XLOC_071923	c75122_g1_i1:21-1058	cdRNA01-preDia	cdRNA04-Dia-R3	3.22561	30.5774	3.24482	5.00E-05	0.0249845
RNA-Seq	Trinity	XLOC_071977	c75171_g1_i1:0-681	cdRNA01-preDia	cdRNA05-postDia	9.52626	68.179	2.83935	5.00E-05	0.0249845
RNA-Seq	Trinity	XLOC_071977	c75171_g1_i1:0-681	cdRNA03-Dia-R2	cdRNA05-postDia	8.4355	68.179	3.01478	5.00E-05	0.0249845
RNA-Seq	Trinity	XLOC_071977	c75171_g1_i1:0-681	cdRNA04-Dia-R3	cdRNA05-postDia	7.23093	68.179	3.23707	5.00E-05	0.0249845

RNA-Seq	Trinity	XLOC_071977	c75171_g1_i1:0-681	cdRNA02-Dia-R1	cdRNA05-postDia	4.80378	68.179	3.82709	5.00E-05	0.0249845
RNA-Seq	Trinity	XLOC_071985	c7517_g1_i1:0-3344	cdRNA04-Dia-R3	cdRNA05-postDia	6.77196	35.7304	2.39951	5.00E-05	0.0249845
RNA-Seq	Trinity	XLOC_071985	c7517_g1_i1:0-3344	cdRNA02-Dia-R1	cdRNA05-postDia	6.43518	35.7304	2.4731	5.00E-05	0.0249845
RNA-Seq	Trinity	XLOC_072144	c75328_g1_i1:0-3740	cdRNA03-Dia-R2	cdRNA05-postDia	29.018	5.71459	-2.34423	5.00E-05	0.0249845
RNA-Seq	Trinity	XLOC_072144	c75328_g1_i1:0-3740	cdRNA04-Dia-R3	cdRNA05-postDia	28.5993	5.71459	-2.32326	0.0001	0.0432976
RNA-Seq	Trinity	XLOC_072219	c75396_g1_i1:0-1006	cdRNA02-Dia-R1	cdRNA04-Dia-R3	15.5473	2.12674	-2.86995	5.00E-05	0.0249845
RNA-Seq	Trinity	XLOC_072219	c75396_g1_i1:0-1006	cdRNA02-Dia-R1	cdRNA05-postDia	15.5473	88.9037	2.51558	5.00E-05	0.0249845
RNA-Seq	Trinity	XLOC_072219	c75396_g1_i1:0-1006	cdRNA03-Dia-R2	cdRNA05-postDia	3.2956	88.9037	4.75363	5.00E-05	0.0249845
RNA-Seq	Trinity	XLOC_072219	c75396_g1_i1:0-1006	cdRNA01-preDia	cdRNA05-postDia	3.20248	88.9037	4.79498	5.00E-05	0.0249845
RNA-Seq	Trinity	XLOC_072219	c75396_g1_i1:0-1006	cdRNA04-Dia-R3	cdRNA05-postDia	2.12674	88.9037	5.38553	5.00E-05	0.0249845
RNA-Seq	Trinity	XLOC_072399	c75556_g1_i1:0-1513	cdRNA01-preDia	cdRNA03-Dia-R2	15.546	89.1747	2.52009	0.0001	0.0432976
RNA-Seq	Trinity	XLOC_072399	c75556_g1_i1:0-1513	cdRNA01-preDia	cdRNA04-Dia-R3	15.546	90.3429	2.53887	5.00E-05	0.0249845
RNA-Seq	Trinity	XLOC_072399	c75556_g1_i1:0-1513	cdRNA01-preDia	cdRNA05-postDia	15.546	121.758	2.96941	5.00E-05	0.0249845
RNA-Seq	Trinity	XLOC_072793	c75920_g1_i1:4-480	cdRNA01-preDia	cdRNA05-postDia	73.2142	3.95608	-4.20998	5.00E-05	0.0249845
RNA-Seq	Trinity	XLOC_072793	c75920_g1_i1:4-480	cdRNA01-preDia	cdRNA04-Dia-R3	73.2142	7.80336	-3.22996	5.00E-05	0.0249845
RNA-Seq	Trinity	XLOC_072793	c75920_g1_i1:4-480	cdRNA01-preDia	cdRNA03-Dia-R2	73.2142	8.41161	-3.12167	5.00E-05	0.0249845
RNA-Seq	Trinity	XLOC_072959	c76081_g1_i1:0-520	cdRNA03-Dia-R2	cdRNA05-postDia	8.61361	50.8266	2.56089	5.00E-05	0.0249845
RNA-Seq	Trinity	XLOC_073043	c7615_g1_i1:0-836	cdRNA01-preDia	cdRNA04-Dia-R3	26.0714	4.2581	-2.61419	5.00E-05	0.0249845
RNA-Seq	Trinity	XLOC_073107	c76220_g1_i1:0-1498	cdRNA02-Dia-R1	cdRNA05-postDia	2.59874	15.6773	2.59279	5.00E-05	0.0249845
RNA-Seq	Trinity	XLOC_073412	c7649_g1_i2:4-557	cdRNA01-preDia	cdRNA03-Dia-R2	80.2711	14.468	-2.47201	5.00E-05	0.0249845
RNA-Seq	Trinity	XLOC_073469	c76550_g1_i1:0-2529	cdRNA01-preDia	cdRNA05-postDia	42.9811	5.03979	-3.09227	5.00E-05	0.0249845
RNA-Seq	Trinity	XLOC_073545	c7661_g1_i1:0-2159	cdRNA04-Dia-R3	cdRNA05-postDia	9.96544	1.85517	-2.42539	0.0001	0.0432976
RNA-Seq	Trinity	XLOC_073675	c76738_g1_i1:0-1102	cdRNA04-Dia-R3	cdRNA05-postDia	2.02601	15.5781	2.94281	5.00E-05	0.0249845
RNA-Seq	Trinity	XLOC_073675	c76738_g1_i1:0-1102	cdRNA03-Dia-R2	cdRNA05-postDia	1.88898	15.5781	3.04385	5.00E-05	0.0249845
RNA-Seq	Trinity	XLOC_073711	c76772_g1_i1:0-1428	cdRNA01-preDia	cdRNA02-Dia-R1	0.7425	8.21775	3.46828	5.00E-05	0.0249845
RNA-Seq	Trinity	XLOC_073711	c76772_g1_i1:0-1428	cdRNA01-preDia	cdRNA04-Dia-R3	0.7425	10.0642	3.7607	5.00E-05	0.0249845
RNA-Seq	Trinity	XLOC_073711	c76772_g1_i1:0-1428	cdRNA01-preDia	cdRNA03-Dia-R2	0.7425	11.1837	3.91286	5.00E-05	0.0249845
RNA-Seq	Trinity	XLOC_073804	c76860_g1_i1:0-609	cdRNA01-preDia	cdRNA05-postDia	9.95144	49.9903	2.32867	5.00E-05	0.0249845
RNA-Seq	Trinity	XLOC_073804	c76860_g1_i1:0-609	cdRNA03-Dia-R2	cdRNA05-postDia	7.69483	49.9903	2.69969	5.00E-05	0.0249845
RNA-Seq	Trinity	XLOC_073804	c76860_g1_i1:0-609	cdRNA04-Dia-R3	cdRNA05-postDia	6.08314	49.9903	3.03876	5.00E-05	0.0249845
RNA-Seq	Trinity	XLOC_073865	c76915_g1_i1:0-331	cdRNA03-Dia-R2	cdRNA05-postDia	10.742	66.0461	2.62021	5.00E-05	0.0249845
RNA-Seq	Trinity	XLOC_073987	c77028_g1_i1:0-905	cdRNA03-Dia-R2	cdRNA05-postDia	2.58865	16.431	2.66615	5.00E-05	0.0249845
RNA-Seq	Trinity	XLOC_074046	c77084_g1_i1:1-917	cdRNA04-Dia-R3	cdRNA05-postDia	7.6658	38.0078	2.30979	0.0001	0.0432976
RNA-Seq	Trinity	XLOC_074046	c77084_g1_i1:1-917	cdRNA01-preDia	cdRNA05-postDia	2.33086	38.0078	4.02736	5.00E-05	0.0249845
RNA-Seq	Trinity	XLOC_074099	c77133_g1_i1:0-993	cdRNA01-preDia	cdRNA05-postDia	47.2261	7.02137	-2.74976	5.00E-05	0.0249845
RNA-Seq	Trinity	XLOC_074178	c77207_g1_i1:0-1402	cdRNA03-Dia-R2	cdRNA05-postDia	270.776	23.2877	-3.53946	5.00E-05	0.0249845
RNA-Seq	Trinity	XLOC_074178	c77207_g1_i1:0-1402	cdRNA04-Dia-R3	cdRNA05-postDia	263.679	23.2877	-3.50114	5.00E-05	0.0249845
RNA-Seq	Trinity	XLOC_074236	c77261_g1_i1:0-636	cdRNA02-Dia-R1	cdRNA05-postDia	6.75074	49.7259	2.88088	5.00E-05	0.0249845
RNA-Seq	Trinity	XLOC_074236	c77261_g1_i1:0-636	cdRNA01-preDia	cdRNA05-postDia	2.56104	49.7259	4.2792	5.00E-05	0.0249845
RNA-Seq	Trinity	XLOC_074312	c77335_g1_i1:0-293	cdRNA01-preDia	cdRNA04-Dia-R3	32.0672	181.324	2.4994	5.00E-05	0.0249845
RNA-Seq	Trinity	XLOC_074312	c77335_g1_i1:0-293	cdRNA01-preDia	cdRNA03-Dia-R2	32.0672	218.626	2.76929	5.00E-05	0.0249845
RNA-Seq	Trinity	XLOC_074379	c77398_g1_i1:9-1189	cdRNA01-preDia	cdRNA04-Dia-R3	16.8824	2.17478	-2.95658	0.0001	0.0432976
RNA-Seq	Trinity	XLOC_074379	c77398_g1_i1:9-1189	cdRNA01-preDia	cdRNA03-Dia-R2	16.8824	3.15268	-2.42087	5.00E-05	0.0249845
RNA-Seq	Trinity	XLOC_074600	c77599_g1_i1:0-671	cdRNA01-preDia	cdRNA05-postDia	107.963	1.0241	-6.72004	5.00E-05	0.0249845
RNA-Seq	Trinity	XLOC_074600	c77599_g1_i1:0-671	cdRNA01-preDia	cdRNA02-Dia-R1	107.963	4.5243	-4.5767	5.00E-05	0.0249845
RNA-Seq	Trinity	XLOC_074600	c77599_g1_i1:0-671	cdRNA01-preDia	cdRNA03-Dia-R2	107.963	12.5863	-3.10062	5.00E-05	0.0249845
RNA-Seq	Trinity	XLOC_074600	c77599_g1_i1:0-671	cdRNA01-preDia	cdRNA04-Dia-R3	107.963	14.1222	-2.9345	5.00E-05	0.0249845

RNA-Seq	Trinity	XLOC_074643	c77638_g1_i1:0-249	cdRNA04-Dia-R3	cdRNA05-postDia	22.1921	146.187	2.71969	5.00E-05	0.0249845
RNA-Seq	Trinity	XLOC_074643	c77638_g1_i1:0-249	cdRNA01-preDia	cdRNA05-postDia	15.6502	146.187	3.22356	5.00E-05	0.0249845
RNA-Seq	Trinity	XLOC_074663	c77657_g1_i1:0-1239	cdRNA04-Dia-R3	cdRNA05-postDia	12.4513	73.8959	2.56919	5.00E-05	0.0249845
RNA-Seq	Trinity	XLOC_074663	c77657_g1_i1:0-1239	cdRNA03-Dia-R2	cdRNA05-postDia	11.7394	73.8959	2.65414	5.00E-05	0.0249845
RNA-Seq	Trinity	XLOC_074663	c77657_g1_i1:0-1239	cdRNA02-Dia-R1	cdRNA05-postDia	8.91329	73.8959	3.05146	5.00E-05	0.0249845
RNA-Seq	Trinity	XLOC_075182	c7813_g1_i1:0-428	cdRNA02-Dia-R1	cdRNA05-postDia	18.1996	200.865	3.46425	5.00E-05	0.0249845
RNA-Seq	Trinity	XLOC_075182	c7813_g1_i1:0-428	cdRNA04-Dia-R3	cdRNA05-postDia	18.1045	200.865	3.47181	5.00E-05	0.0249845
RNA-Seq	Trinity	XLOC_075182	c7813_g1_i1:0-428	cdRNA03-Dia-R2	cdRNA05-postDia	15.849	200.865	3.66376	5.00E-05	0.0249845
RNA-Seq	Trinity	XLOC_075182	c7813_g1_i1:0-428	cdRNA01-preDia	cdRNA05-postDia	15.1695	200.865	3.72698	5.00E-05	0.0249845
RNA-Seq	Trinity	XLOC_075256	c78209_g1_i1:0-1200	cdRNA03-Dia-R2	cdRNA05-postDia	2.29723	23.9291	3.3808	5.00E-05	0.0249845
RNA-Seq	Trinity	XLOC_075256	c78209_g1_i1:0-1200	cdRNA04-Dia-R3	cdRNA05-postDia	2.28333	23.9291	3.38955	5.00E-05	0.0249845
RNA-Seq	Trinity	XLOC_075256	c78209_g1_i1:0-1200	cdRNA02-Dia-R1	cdRNA05-postDia	2.00185	23.9291	3.57936	5.00E-05	0.0249845
RNA-Seq	Trinity	XLOC_075519	c78458_g1_i1:1-628	cdRNA02-Dia-R1	cdRNA05-postDia	22.9343	409.38	4.15786	5.00E-05	0.0249845
RNA-Seq	Trinity	XLOC_075519	c78458_g1_i1:1-628	cdRNA01-preDia	cdRNA05-postDia	8.96752	409.38	5.51259	5.00E-05	0.0249845
RNA-Seq	Trinity	XLOC_075519	c78458_g1_i1:1-628	cdRNA03-Dia-R2	cdRNA05-postDia	6.15504	409.38	6.05553	5.00E-05	0.0249845
RNA-Seq	Trinity	XLOC_075519	c78458_g1_i1:1-628	cdRNA04-Dia-R3	cdRNA05-postDia	5.82429	409.38	6.13522	5.00E-05	0.0249845
RNA-Seq	Trinity	XLOC_075624	c78556_g1_i1:0-855	cdRNA03-Dia-R2	cdRNA05-postDia	6.66567	35.3858	2.40835	5.00E-05	0.0249845
RNA-Seq	Trinity	XLOC_075624	c78556_g1_i1:0-855	cdRNA04-Dia-R3	cdRNA05-postDia	5.70584	35.3858	2.63266	5.00E-05	0.0249845
RNA-Seq	Trinity	XLOC_075624	c78556_g1_i1:0-855	cdRNA01-preDia	cdRNA05-postDia	5.28061	35.3858	2.74439	5.00E-05	0.0249845
RNA-Seq	Trinity	XLOC_075668	c78597_g1_i1:0-761	cdRNA01-preDia	cdRNA03-Dia-R2	14.8264	2.47881	-2.58045	0.0001	0.0432976
RNA-Seq	Trinity	XLOC_075668	c78597_g1_i1:0-761	cdRNA04-Dia-R3	cdRNA05-postDia	3.26632	32.3418	3.30766	5.00E-05	0.0249845
RNA-Seq	Trinity	XLOC_075668	c78597_g1_i1:0-761	cdRNA03-Dia-R2	cdRNA05-postDia	2.47881	32.3418	3.70568	5.00E-05	0.0249845
RNA-Seq	Trinity	XLOC_075716	c78642_g1_i1:0-1684	cdRNA01-preDia	cdRNA04-Dia-R3	21.8128	2.27933	-3.25849	5.00E-05	0.0249845
RNA-Seq	Trinity	XLOC_075716	c78642_g1_i1:0-1684	cdRNA01-preDia	cdRNA03-Dia-R2	21.8128	2.4789	-3.1374	5.00E-05	0.0249845
RNA-Seq	Trinity	XLOC_075945	c7885_g2_i1:0-1819	cdRNA03-Dia-R2	cdRNA05-postDia	1.90528	10.1181	2.40886	5.00E-05	0.0249845
RNA-Seq	Trinity	XLOC_075997	c78906_g1_i1:17-728	cdRNA01-preDia	cdRNA05-postDia	35.6945	1.10052	-5.01944	5.00E-05	0.0249845
RNA-Seq	Trinity	XLOC_075997	c78906_g1_i1:17-728	cdRNA01-preDia	cdRNA03-Dia-R2	35.6945	1.16711	-4.93468	5.00E-05	0.0249845
RNA-Seq	Trinity	XLOC_075997	c78906_g1_i1:17-728	cdRNA01-preDia	cdRNA04-Dia-R3	35.6945	1.49518	-4.57731	5.00E-05	0.0249845
RNA-Seq	Trinity	XLOC_076095	c7899_g1_i2:3-838	cdRNA01-preDia	cdRNA05-postDia	14.5279	120.828	3.05605	5.00E-05	0.0249845
RNA-Seq	Trinity	XLOC_076117	c79019_g1_i1:13-573	cdRNA04-Dia-R3	cdRNA05-postDia	1.70348	21.2706	3.6423	0.0001	0.0432976
RNA-Seq	Trinity	XLOC_076142	c79039_g1_i1:0-762	cdRNA01-preDia	cdRNA05-postDia	59.4825	1.99988	-4.89448	5.00E-05	0.0249845
RNA-Seq	Trinity	XLOC_076142	c79039_g1_i1:0-762	cdRNA01-preDia	cdRNA02-Dia-R1	59.4825	3.30651	-4.16909	5.00E-05	0.0249845
RNA-Seq	Trinity	XLOC_076142	c79039_g1_i1:0-762	cdRNA01-preDia	cdRNA04-Dia-R3	59.4825	5.50204	-3.43443	5.00E-05	0.0249845
RNA-Seq	Trinity	XLOC_076142	c79039_g1_i1:0-762	cdRNA01-preDia	cdRNA03-Dia-R2	59.4825	5.6557	-3.39469	5.00E-05	0.0249845
RNA-Seq	Trinity	XLOC_076250	c79145_g1_i1:2-229	cdRNA02-Dia-R1	cdRNA05-postDia	20.6906	205.98	3.31546	5.00E-05	0.0249845
RNA-Seq	Trinity	XLOC_076325	c79215_g1_i1:0-588	cdRNA04-Dia-R3	cdRNA05-postDia	171.549	1405.91	3.03481	5.00E-05	0.0249845
RNA-Seq	Trinity	XLOC_076325	c79215_g1_i1:0-588	cdRNA03-Dia-R2	cdRNA05-postDia	156.971	1405.91	3.16293	5.00E-05	0.0249845
RNA-Seq	Trinity	XLOC_076411	c79297_g1_i1:4-256	cdRNA02-Dia-R1	cdRNA05-postDia	115.537	1411.14	3.61043	5.00E-05	0.0249845
RNA-Seq	Trinity	XLOC_076411	c79297_g1_i1:4-256	cdRNA01-preDia	cdRNA05-postDia	60.3151	1411.14	4.54819	5.00E-05	0.0249845
RNA-Seq	Trinity	XLOC_076411	c79297_g1_i1:4-256	cdRNA03-Dia-R2	cdRNA05-postDia	49.0892	1411.14	4.84531	5.00E-05	0.0249845
RNA-Seq	Trinity	XLOC_076411	c79297_g1_i1:4-256	cdRNA04-Dia-R3	cdRNA05-postDia	42.7637	1411.14	5.04433	5.00E-05	0.0249845
RNA-Seq	Trinity	XLOC_076459	c7933_g1_i2:0-1875	cdRNA01-preDia	cdRNA04-Dia-R3	32.4672	5.62542	-2.52895	5.00E-05	0.0249845
RNA-Seq	Trinity	XLOC_076459	c7933_g1_i2:0-1875	cdRNA01-preDia	cdRNA03-Dia-R2	32.4672	5.90202	-2.4597	5.00E-05	0.0249845
RNA-Seq	Trinity	XLOC_076666	c79536_g1_i1:0-1585	cdRNA04-Dia-R3	cdRNA05-postDia	12.1862	0.865794	-3.81508	5.00E-05	0.0249845
RNA-Seq	Trinity	XLOC_076666	c79536_g1_i1:0-1585	cdRNA03-Dia-R2	cdRNA05-postDia	10.7407	0.865794	-3.63292	5.00E-05	0.0249845
RNA-Seq	Trinity	XLOC_076666	c79536_g1_i1:0-1585	cdRNA02-Dia-R1	cdRNA03-Dia-R2	0.732662	10.7407	3.87379	5.00E-05	0.0249845
RNA-Seq	Trinity	XLOC_076666	c79536_g1_i1:0-1585	cdRNA02-Dia-R1	cdRNA04-Dia-R3	0.732662	12.1862	4.05596	5.00E-05	0.0249845

RNA-Seq	Trinity	XLOC_076834	c79699_g1_i1:0-617	cdRNA04-Dia-R3	cdRNA05-postDia	5.04757	29.915	2.56721	5.00E-05	0.0249845
RNA-Seq	Trinity	XLOC_076930	c7978_g1_i1:0-1210	cdRNA04-Dia-R3	cdRNA05-postDia	4.1445	26.306	2.66612	0.0001	0.0432976
RNA-Seq	Trinity	XLOC_076950	c79807_g1_i1:0-348	cdRNA01-preDia	cdRNA05-postDia	138.569	978.638	2.82017	5.00E-05	0.0249845
RNA-Seq	Trinity	XLOC_077115	c79970_g1_i1:0-928	cdRNA04-Dia-R3	cdRNA05-postDia	3.66467	18.9366	2.36943	5.00E-05	0.0249845
RNA-Seq	Trinity	XLOC_077159	c8000_g1_i1:0-660	cdRNA02-Dia-R1	cdRNA05-postDia	11.0289	60.2189	2.44893	5.00E-05	0.0249845
RNA-Seq	Trinity	XLOC_077159	c8000_g1_i1:0-660	cdRNA03-Dia-R2	cdRNA05-postDia	10.7272	60.2189	2.48895	5.00E-05	0.0249845
RNA-Seq	Trinity	XLOC_077223	c8006_g1_i2:40-2498	cdRNA03-Dia-R2	cdRNA05-postDia	19.1295	1.69748	-3.49433	5.00E-05	0.0249845
RNA-Seq	Trinity	XLOC_077223	c8006_g1_i2:40-2498	cdRNA04-Dia-R3	cdRNA05-postDia	18.6827	1.69748	-3.46024	5.00E-05	0.0249845
RNA-Seq	Trinity	XLOC_077223	c8006_g1_i2:40-2498	cdRNA02-Dia-R1	cdRNA04-Dia-R3	3.27429	18.6827	2.51245	5.00E-05	0.0249845
RNA-Seq	Trinity	XLOC_077223	c8006_g1_i2:40-2498	cdRNA02-Dia-R1	cdRNA03-Dia-R2	3.27429	19.1295	2.54655	5.00E-05	0.0249845
RNA-Seq	Trinity	XLOC_077223	c8006_g1_i2:40-2498	cdRNA01-preDia	cdRNA04-Dia-R3	0.315552	18.6827	5.88769	5.00E-05	0.0249845
RNA-Seq	Trinity	XLOC_077223	c8006_g1_i2:40-2498	cdRNA01-preDia	cdRNA03-Dia-R2	0.315552	19.1295	5.92178	5.00E-05	0.0249845
RNA-Seq	Trinity	XLOC_077523	c8035_g1_i1:0-2055	cdRNA03-Dia-R2	cdRNA05-postDia	33.6735	3.89727	-3.11108	5.00E-05	0.0249845
RNA-Seq	Trinity	XLOC_077523	c8035_g1_i1:0-2055	cdRNA04-Dia-R3	cdRNA05-postDia	32.9685	3.89727	-3.08056	5.00E-05	0.0249845
RNA-Seq	Trinity	XLOC_077523	c8035_g1_i1:0-2055	cdRNA01-preDia	cdRNA04-Dia-R3	4.5043	32.9685	2.87171	5.00E-05	0.0249845
RNA-Seq	Trinity	XLOC_077523	c8035_g1_i1:0-2055	cdRNA01-preDia	cdRNA03-Dia-R2	4.5043	33.6735	2.90224	5.00E-05	0.0249845
RNA-Seq	Trinity	XLOC_077687	c80508_g1_i1:12-785	cdRNA03-Dia-R2	cdRNA05-postDia	2.42654	19.8924	3.03525	5.00E-05	0.0249845
RNA-Seq	Trinity	XLOC_077846	c80663_g1_i1:0-536	cdRNA03-Dia-R2	cdRNA05-postDia	4.63394	32.5316	2.81153	0.0001	0.0432976
RNA-Seq	Trinity	XLOC_077909	c80722_g1_i1:0-331	cdRNA02-Dia-R1	cdRNA05-postDia	15.5122	97.3637	2.64998	5.00E-05	0.0249845
RNA-Seq	Trinity	XLOC_077909	c80722_g1_i1:0-331	cdRNA01-preDia	cdRNA05-postDia	13.5265	97.3637	2.8476	0.0001	0.0432976
RNA-Seq	Trinity	XLOC_078030	c8083_g1_i1:0-1386	cdRNA03-Dia-R2	cdRNA05-postDia	12.7492	62.8851	2.30231	0.0001	0.0432976
RNA-Seq	Trinity	XLOC_078030	c8083_g1_i1:0-1386	cdRNA04-Dia-R3	cdRNA05-postDia	12.4421	62.8851	2.33749	0.0001	0.0432976
RNA-Seq	Trinity	XLOC_078030	c8083_g1_i1:0-1386	cdRNA01-preDia	cdRNA05-postDia	11.7672	62.8851	2.41794	5.00E-05	0.0249845
RNA-Seq	Trinity	XLOC_078223	c81033_g1_i1:0-369	cdRNA03-Dia-R2	cdRNA05-postDia	75.3764	10.1216	-2.89668	5.00E-05	0.0249845
RNA-Seq	Trinity	XLOC_078223	c81033_g1_i1:0-369	cdRNA04-Dia-R3	cdRNA05-postDia	67.8395	10.1216	-2.74469	5.00E-05	0.0249845
RNA-Seq	Trinity	XLOC_078365	c81174_g1_i1:10-809	cdRNA03-Dia-R2	cdRNA05-postDia	15.8457	1.94251	-3.0281	5.00E-05	0.0249845
RNA-Seq	Trinity	XLOC_078365	c81174_g1_i1:10-809	cdRNA04-Dia-R3	cdRNA05-postDia	15.3523	1.94251	-2.98246	5.00E-05	0.0249845
RNA-Seq	Trinity	XLOC_078450	c81257_g1_i1:0-302	cdRNA03-Dia-R2	cdRNA05-postDia	26.7785	149.779	2.48369	0.0001	0.0432976
RNA-Seq	Trinity	XLOC_078450	c81257_g1_i1:0-302	cdRNA01-preDia	cdRNA05-postDia	16.7155	149.779	3.16358	5.00E-05	0.0249845
RNA-Seq	Trinity	XLOC_078484	c8128_g1_i1:0-630	cdRNA02-Dia-R1	cdRNA05-postDia	6.28698	40.603	2.69115	5.00E-05	0.0249845
RNA-Seq	Trinity	XLOC_078484	c8128_g1_i1:0-630	cdRNA04-Dia-R3	cdRNA05-postDia	5.07149	40.603	3.00111	5.00E-05	0.0249845
RNA-Seq	Trinity	XLOC_078484	c8128_g1_i1:0-630	cdRNA03-Dia-R2	cdRNA05-postDia	4.90788	40.603	3.04841	5.00E-05	0.0249845
RNA-Seq	Trinity	XLOC_078484	c8128_g1_i1:0-630	cdRNA01-preDia	cdRNA05-postDia	2.13334	40.603	4.2504	5.00E-05	0.0249845
RNA-Seq	Trinity	XLOC_078597	c81398_g1_i1:0-1066	cdRNA01-preDia	cdRNA05-postDia	3.85053	26.3081	2.77238	5.00E-05	0.0249845
RNA-Seq	Trinity	XLOC_078652	c8144_g1_i1:0-651	cdRNA03-Dia-R2	cdRNA05-postDia	9.44288	52.1587	2.46561	5.00E-05	0.0249845
RNA-Seq	Trinity	XLOC_078652	c8144_g1_i1:0-651	cdRNA04-Dia-R3	cdRNA05-postDia	9.41209	52.1587	2.47032	5.00E-05	0.0249845
RNA-Seq	Trinity	XLOC_078652	c8144_g1_i1:0-651	cdRNA01-preDia	cdRNA05-postDia	9.10483	52.1587	2.5182	5.00E-05	0.0249845
RNA-Seq	Trinity	XLOC_078652	c8144_g1_i1:0-651	cdRNA02-Dia-R1	cdRNA05-postDia	7.65597	52.1587	2.76825	5.00E-05	0.0249845
RNA-Seq	Trinity	XLOC_079060	c81840_g1_i1:0-877	cdRNA01-preDia	cdRNA05-postDia	2.87692	22.8589	2.99016	5.00E-05	0.0249845
RNA-Seq	Trinity	XLOC_079230	c82005_g1_i1:0-1072	cdRNA01-preDia	cdRNA05-postDia	1.54777	14.3717	3.21497	5.00E-05	0.0249845
RNA-Seq	Trinity	XLOC_079337	c82111_g1_i1:0-245	cdRNA01-preDia	cdRNA02-Dia-R1	17.1812	127.24	2.88864	5.00E-05	0.0249845
RNA-Seq	Trinity	XLOC_079515	c82286_g1_i1:0-621	cdRNA04-Dia-R3	cdRNA05-postDia	19.0877	3.24953	-2.55434	5.00E-05	0.0249845
RNA-Seq	Trinity	XLOC_079939	c82687_g1_i1:4-290	cdRNA03-Dia-R2	cdRNA05-postDia	18.9203	96.9414	2.35718	0.0001	0.0432976
RNA-Seq	Trinity	XLOC_080352	c83081_g1_i1:0-702	cdRNA01-preDia	cdRNA04-Dia-R3	3.72917	20.6258	2.46753	0.0001	0.0432976
RNA-Seq	Trinity	XLOC_080417	c83142_g1_i1:0-1225	cdRNA01-preDia	cdRNA05-postDia	26.0071	1.83467	-3.82532	5.00E-05	0.0249845
RNA-Seq	Trinity	XLOC_080417	c83142_g1_i1:0-1225	cdRNA02-Dia-R1	cdRNA05-postDia	22.4722	1.83467	-3.61455	5.00E-05	0.0249845
RNA-Seq	Trinity	XLOC_080417	c83142_g1_i1:0-1225	cdRNA03-Dia-R2	cdRNA05-postDia	18.4301	1.83467	-3.32847	5.00E-05	0.0249845

RNA-Seq	Trinity	XLOC_080417	c83142_g1_i1:0-1225	cdRNA04-Dia-R3	cdRNA05-postDia	16.7428	1.83467	-3.18995	5.00E-05	0.0249845
RNA-Seq	Trinity	XLOC_080628	c83345_g1_i1:6-660	cdRNA01-preDia	cdRNA05-postDia	5.88307	39.0575	2.73096	5.00E-05	0.0249845
RNA-Seq	Trinity	XLOC_080642	c8335_g1_i1:0-2442	cdRNA03-Dia-R2	cdRNA05-postDia	7.93215	38.4664	2.27782	0.0001	0.0432976
RNA-Seq	Trinity	XLOC_080684	c83401_g1_i1:0-890	cdRNA03-Dia-R2	cdRNA05-postDia	1.55265	17.3712	3.4839	5.00E-05	0.0249845
RNA-Seq	Trinity	XLOC_081065	c83767_g1_i1:4-580	cdRNA04-Dia-R3	cdRNA05-postDia	4.58498	28.1766	2.61951	0.0001	0.0432976
RNA-Seq	Trinity	XLOC_081090	c8378_g1_i1:0-856	cdRNA01-preDia	cdRNA05-postDia	3.3332	75.92	4.5095	5.00E-05	0.0249845
RNA-Seq	Trinity	XLOC_081187	c8387_g1_i1:0-469	cdRNA02-Dia-R1	cdRNA05-postDia	3.70569	40.6058	3.45387	5.00E-05	0.0249845
RNA-Seq	Trinity	XLOC_081441	c8412_g1_i1:6-641	cdRNA01-preDia	cdRNA03-Dia-R2	32.729	1.18985	-4.78171	0.0001	0.0432976
RNA-Seq	Trinity	XLOC_081441	c8412_g1_i1:6-641	cdRNA01-preDia	cdRNA04-Dia-R3	32.729	1.14324	-4.83937	5.00E-05	0.0249845
RNA-Seq	Trinity	XLOC_081441	c8412_g1_i1:6-641	cdRNA01-preDia	cdRNA05-postDia	32.729	2.95904	-3.46737	5.00E-05	0.0249845
RNA-Seq	Trinity	XLOC_081537	c84221_g1_i1:0-690	cdRNA02-Dia-R1	cdRNA05-postDia	2.38803	16.6471	2.80138	5.00E-05	0.0249845
RNA-Seq	Trinity	XLOC_081557	c84240_g1_i1:0-476	cdRNA03-Dia-R2	cdRNA05-postDia	117.763	23.1504	-2.34677	5.00E-05	0.0249845
RNA-Seq	Trinity	XLOC_081588	c8426_g2_i1:1-241	cdRNA02-Dia-R1	cdRNA05-postDia	24.7508	151.094	2.60989	0.0001	0.0432976
RNA-Seq	Trinity	XLOC_081661	c8433_g1_i2:0-389	cdRNA03-Dia-R2	cdRNA05-postDia	6.39185	120.113	4.23201	5.00E-05	0.0249845
RNA-Seq	Trinity	XLOC_081661	c8433_g1_i2:0-389	cdRNA04-Dia-R3	cdRNA05-postDia	3.88957	120.113	4.94864	5.00E-05	0.0249845
RNA-Seq	Trinity	XLOC_081770	c84445_g1_i1:0-297	cdRNA01-preDia	cdRNA03-Dia-R2	722.132	107.168	-2.75239	5.00E-05	0.0249845
RNA-Seq	Trinity	XLOC_081770	c84445_g1_i1:0-297	cdRNA01-preDia	cdRNA04-Dia-R3	722.132	109.21	-2.72516	5.00E-05	0.0249845
RNA-Seq	Trinity	XLOC_082042	c84721_g1_i1:0-260	cdRNA04-Dia-R3	cdRNA05-postDia	38.2928	555.893	3.85966	5.00E-05	0.0249845
RNA-Seq	Trinity	XLOC_082042	c84721_g1_i1:0-260	cdRNA03-Dia-R2	cdRNA05-postDia	37.4749	555.893	3.89081	5.00E-05	0.0249845
RNA-Seq	Trinity	XLOC_082042	c84721_g1_i1:0-260	cdRNA01-preDia	cdRNA05-postDia	29.1951	555.893	4.25101	5.00E-05	0.0249845
RNA-Seq	Trinity	XLOC_082042	c84721_g1_i1:0-260	cdRNA02-Dia-R1	cdRNA05-postDia	26.9236	555.893	4.36787	5.00E-05	0.0249845
RNA-Seq	Trinity	XLOC_082117	c84793_g1_i1:0-527	cdRNA04-Dia-R3	cdRNA05-postDia	27.1066	4.56534	-2.56985	0.0001	0.0432976
RNA-Seq	Trinity	XLOC_084629	c87281_g1_i1:0-366	cdRNA02-Dia-R1	cdRNA05-postDia	11.1757	65.022	2.54056	5.00E-05	0.0249845
RNA-Seq	Trinity	XLOC_084629	c87281_g1_i1:0-366	cdRNA03-Dia-R2	cdRNA05-postDia	10.9289	65.022	2.57278	5.00E-05	0.0249845
RNA-Seq	Trinity	XLOC_084629	c87281_g1_i1:0-366	cdRNA04-Dia-R3	cdRNA05-postDia	9.56732	65.022	2.76474	5.00E-05	0.0249845
RNA-Seq	Trinity	XLOC_084752	c87405_g1_i1:0-237	cdRNA01-preDia	cdRNA04-Dia-R3	463.911	89.306	-2.37702	5.00E-05	0.0249845
RNA-Seq	Trinity	XLOC_084982	c87644_g1_i1:0-610	cdRNA03-Dia-R2	cdRNA05-postDia	2.81773	22.9703	3.02717	5.00E-05	0.0249845
RNA-Seq	Trinity	XLOC_085030	c8768_g1_i1:0-1819	cdRNA01-preDia	cdRNA03-Dia-R2	38.2417	5.81355	-2.71766	5.00E-05	0.0249845
RNA-Seq	Trinity	XLOC_085030	c8768_g1_i1:0-1819	cdRNA01-preDia	cdRNA04-Dia-R3	38.2417	6.43069	-2.5721	5.00E-05	0.0249845
RNA-Seq	Trinity	XLOC_085148	c87812_g1_i1:23-238	cdRNA02-Dia-R1	cdRNA05-postDia	44.6672	366.683	3.03725	5.00E-05	0.0249845
RNA-Seq	Trinity	XLOC_085148	c87812_g1_i1:23-238	cdRNA01-preDia	cdRNA05-postDia	16.4747	366.683	4.47621	5.00E-05	0.0249845
RNA-Seq	Trinity	XLOC_085148	c87812_g1_i1:23-238	cdRNA03-Dia-R2	cdRNA05-postDia	14.2547	366.683	4.68503	0.0001	0.0432976
RNA-Seq	Trinity	XLOC_086304	c89041_g1_i1:0-734	cdRNA01-preDia	cdRNA05-postDia	2.60937	32.1846	3.6246	5.00E-05	0.0249845
RNA-Seq	Trinity	XLOC_086771	c89551_g1_i1:0-6398	cdRNA01-preDia	cdRNA05-postDia	3.15782	17.8044	2.49523	5.00E-05	0.0249845
RNA-Seq	Trinity	XLOC_086853	c89626_g1_i1:23-3789	cdRNA01-preDia	cdRNA04-Dia-R3	8.60837	48.3426	2.48948	0.0001	0.0432976
RNA-Seq	Trinity	XLOC_086853	c89626_g1_i1:23-3789	cdRNA01-preDia	cdRNA03-Dia-R2	8.60837	49.1289	2.51276	5.00E-05	0.0249845
RNA-Seq	Trinity	XLOC_087048	c89808_g1_i1:0-2041	cdRNA04-Dia-R3	cdRNA05-postDia	3.34261	18.5283	2.47069	0.0001	0.0432976
RNA-Seq	Trinity	XLOC_087048	c89808_g1_i1:0-2041	cdRNA03-Dia-R2	cdRNA05-postDia	3.32859	18.5283	2.47675	0.0001	0.0432976
RNA-Seq	Trinity	XLOC_087090	c89843_g1_i1:0-602	cdRNA01-preDia	cdRNA05-postDia	3.87188	66.0903	4.09333	5.00E-05	0.0249845
RNA-Seq	Trinity	XLOC_087090	c89843_g1_i1:0-602	cdRNA04-Dia-R3	cdRNA05-postDia	3.71407	66.0903	4.15336	5.00E-05	0.0249845
RNA-Seq	Trinity	XLOC_087090	c89843_g1_i1:0-602	cdRNA02-Dia-R1	cdRNA05-postDia	3.36463	66.0903	4.29592	5.00E-05	0.0249845
RNA-Seq	Trinity	XLOC_087090	c89843_g1_i1:0-602	cdRNA03-Dia-R2	cdRNA05-postDia	2.87436	66.0903	4.52312	5.00E-05	0.0249845
RNA-Seq	Trinity	XLOC_087342	c90072_g1_i1:0-1115	cdRNA01-preDia	cdRNA05-postDia	24.5063	161.958	2.72439	5.00E-05	0.0249845
RNA-Seq	Trinity	XLOC_087365	c90095_g1_i1:0-1014	cdRNA02-Dia-R1	cdRNA05-postDia	10.6176	1.42691	-2.8955	5.00E-05	0.0249845
RNA-Seq	Trinity	XLOC_087375	c90104_g1_i1:0-678	cdRNA04-Dia-R3	cdRNA05-postDia	42.9405	5.4653	-2.97397	5.00E-05	0.0249845
RNA-Seq	Trinity	XLOC_087375	c90104_g1_i1:0-678	cdRNA03-Dia-R2	cdRNA05-postDia	39.8645	5.4653	-2.86673	5.00E-05	0.0249845
RNA-Seq	Trinity	XLOC_088079	c90757_g1_i1:0-764	cdRNA01-preDia	cdRNA03-Dia-R2	2.11679	16.2725	2.94249	5.00E-05	0.0249845

RNA-Seq	Trinity	XLOC_088079	c90757_g1_i1:0-764	cdRNA01-preDia	cdRNA04-Dia-R3	2.11679	16.3795	2.95194	5.00E-05	0.0249845
RNA-Seq	Trinity	XLOC_088359	c9100_g1_i1:0-1252	cdRNA01-preDia	cdRNA05-postDia	7.38999	70.165	3.24711	5.00E-05	0.0249845
RNA-Seq	Trinity	XLOC_088359	c9100_g1_i1:0-1252	cdRNA01-preDia	cdRNA04-Dia-R3	7.38999	71.322	3.2707	5.00E-05	0.0249845
RNA-Seq	Trinity	XLOC_088359	c9100_g1_i1:0-1252	cdRNA01-preDia	cdRNA03-Dia-R2	7.38999	74.9831	3.34292	5.00E-05	0.0249845
RNA-Seq	Trinity	XLOC_088467	c9110_g1_i1:4-681	cdRNA02-Dia-R1	cdRNA03-Dia-R2	33.03	5.83802	-2.50023	5.00E-05	0.0249845
RNA-Seq	Trinity	XLOC_088467	c9110_g1_i1:4-681	cdRNA04-Dia-R3	cdRNA05-postDia	6.97155	77.35	3.47185	5.00E-05	0.0249845
RNA-Seq	Trinity	XLOC_088467	c9110_g1_i1:4-681	cdRNA03-Dia-R2	cdRNA05-postDia	5.83802	77.35	3.72785	5.00E-05	0.0249845
RNA-Seq	Trinity	XLOC_088467	c9110_g1_i1:4-681	cdRNA01-preDia	cdRNA05-postDia	5.17933	77.35	3.90057	5.00E-05	0.0249845
RNA-Seq	Trinity	XLOC_088470	c91112_g1_i1:0-737	cdRNA03-Dia-R2	cdRNA05-postDia	3.6266	24.526	2.75763	0.0001	0.0432976
RNA-Seq	Trinity	XLOC_088556	c91190_g1_i1:1-1997	cdRNA04-Dia-R3	cdRNA05-postDia	4.46336	34.83	2.96413	5.00E-05	0.0249845
RNA-Seq	Trinity	XLOC_088556	c91190_g1_i1:1-1997	cdRNA03-Dia-R2	cdRNA05-postDia	4.1266	34.83	3.0773	5.00E-05	0.0249845
RNA-Seq	Trinity	XLOC_088589	c91221_g1_i1:27-512	cdRNA02-Dia-R1	cdRNA05-postDia	11.1195	59.2845	2.41456	0.0001	0.0432976
RNA-Seq	Trinity	XLOC_088996	c91601_g1_i1:0-636	cdRNA03-Dia-R2	cdRNA05-postDia	10.3186	54.6155	2.40406	0.0001	0.0432976
RNA-Seq	Trinity	XLOC_088998	c91603_g1_i1:0-266	cdRNA02-Dia-R1	cdRNA05-postDia	40.228	236.831	2.55758	5.00E-05	0.0249845
RNA-Seq	Trinity	XLOC_088998	c91603_g1_i1:0-266	cdRNA01-preDia	cdRNA05-postDia	22.2561	236.831	3.41158	5.00E-05	0.0249845
RNA-Seq	Trinity	XLOC_089447	c92009_g1_i1:19-1227	cdRNA03-Dia-R2	cdRNA05-postDia	19.1351	2.2627	-3.08011	5.00E-05	0.0249845
RNA-Seq	Trinity	XLOC_089447	c92009_g1_i1:19-1227	cdRNA04-Dia-R3	cdRNA05-postDia	18.7629	2.2627	-3.05177	5.00E-05	0.0249845
RNA-Seq	Trinity	XLOC_089455	c92017_g1_i1:0-1168	cdRNA03-Dia-R2	cdRNA05-postDia	107.309	13.6902	-2.97055	5.00E-05	0.0249845
RNA-Seq	Trinity	XLOC_089455	c92017_g1_i1:0-1168	cdRNA04-Dia-R3	cdRNA05-postDia	104.48	13.6902	-2.93201	5.00E-05	0.0249845
RNA-Seq	Trinity	XLOC_089755	c9229_g1_i1:0-2555	cdRNA01-preDia	cdRNA02-Dia-R1	4.90775	29.5819	2.59158	5.00E-05	0.0249845
RNA-Seq	Trinity	XLOC_089755	c9229_g1_i1:0-2555	cdRNA01-preDia	cdRNA05-postDia	4.90775	36.0581	2.87719	5.00E-05	0.0249845
RNA-Seq	Trinity	XLOC_090028	c92547_g1_i1:0-964	cdRNA04-Dia-R3	cdRNA05-postDia	5.38624	29.1047	2.4339	5.00E-05	0.0249845
RNA-Seq	Trinity	XLOC_090028	c92547_g1_i1:0-964	cdRNA03-Dia-R2	cdRNA05-postDia	4.82728	29.1047	2.59197	5.00E-05	0.0249845
RNA-Seq	Trinity	XLOC_090196	c926_g1_i1:0-355	cdRNA02-Dia-R1	cdRNA05-postDia	11.7729	89.0843	2.9197	5.00E-05	0.0249845
RNA-Seq	Trinity	XLOC_090196	c926_g1_i1:0-355	cdRNA04-Dia-R3	cdRNA05-postDia	9.4968	89.0843	3.22966	5.00E-05	0.0249845
RNA-Seq	Trinity	XLOC_090196	c926_g1_i1:0-355	cdRNA03-Dia-R2	cdRNA05-postDia	8.32341	89.0843	3.41992	5.00E-05	0.0249845
RNA-Seq	Trinity	XLOC_090196	c926_g1_i1:0-355	cdRNA01-preDia	cdRNA05-postDia	6.77389	89.0843	3.71711	5.00E-05	0.0249845
RNA-Seq	Trinity	XLOC_090323	c92812_g1_i1:16-1438	cdRNA02-Dia-R1	cdRNA05-postDia	5.08199	31.0531	2.61127	5.00E-05	0.0249845
RNA-Seq	Trinity	XLOC_090499	c9297_g1_i1:0-464	cdRNA02-Dia-R1	cdRNA05-postDia	14.5355	74.1893	2.35163	0.0001	0.0432976
RNA-Seq	Trinity	XLOC_090499	c9297_g1_i1:0-464	cdRNA01-preDia	cdRNA05-postDia	3.64729	74.1893	4.34631	5.00E-05	0.0249845
RNA-Seq	Trinity	XLOC_090521	c9299_g1_i1:5-434	cdRNA02-Dia-R1	cdRNA05-postDia	4.89339	47.4691	3.27808	5.00E-05	0.0249845
RNA-Seq	Trinity	XLOC_090521	c9299_g1_i1:5-434	cdRNA04-Dia-R3	cdRNA05-postDia	3.87433	47.4691	3.61497	0.0001	0.0432976
RNA-Seq	Trinity	XLOC_090521	c9299_g1_i1:5-434	cdRNA03-Dia-R2	cdRNA05-postDia	2.62977	47.4691	4.17398	5.00E-05	0.0249845
RNA-Seq	Trinity	XLOC_090521	c9299_g1_i1:5-434	cdRNA01-preDia	cdRNA05-postDia	2.45849	47.4691	4.27115	0.0001	0.0432976
RNA-Seq	Trinity	XLOC_090522	c9299_g2_i1:0-323	cdRNA02-Dia-R1	cdRNA05-postDia	12.7915	111.024	3.11761	5.00E-05	0.0249845
RNA-Seq	Trinity	XLOC_090522	c9299_g2_i1:0-323	cdRNA04-Dia-R3	cdRNA05-postDia	7.51954	111.024	3.88409	5.00E-05	0.0249845
RNA-Seq	Trinity	XLOC_090522	c9299_g2_i1:0-323	cdRNA03-Dia-R2	cdRNA05-postDia	5.21744	111.024	4.41139	5.00E-05	0.0249845
RNA-Seq	Trinity	XLOC_090579	c93051_g1_i1:0-876	cdRNA01-preDia	cdRNA05-postDia	9.11399	70.1021	2.9433	5.00E-05	0.0249845
RNA-Seq	Trinity	XLOC_090587	c93059_g1_i1:13-1214	cdRNA02-Dia-R1	cdRNA05-postDia	1.55212	9.83389	2.66352	5.00E-05	0.0249845
RNA-Seq	Trinity	XLOC_090587	c93059_g1_i1:13-1214	cdRNA04-Dia-R3	cdRNA05-postDia	1.36863	9.83389	2.84503	5.00E-05	0.0249845
RNA-Seq	Trinity	XLOC_090628	c9309_g1_i1:0-453	cdRNA02-Dia-R1	cdRNA05-postDia	15.2971	129.965	3.08678	5.00E-05	0.0249845
RNA-Seq	Trinity	XLOC_090628	c9309_g1_i1:0-453	cdRNA03-Dia-R2	cdRNA05-postDia	9.18855	129.965	3.82214	5.00E-05	0.0249845
RNA-Seq	Trinity	XLOC_090628	c9309_g1_i1:0-453	cdRNA04-Dia-R3	cdRNA05-postDia	8.3719	129.965	3.95642	5.00E-05	0.0249845
RNA-Seq	Trinity	XLOC_090628	c9309_g1_i1:0-453	cdRNA01-preDia	cdRNA05-postDia	5.55393	129.965	4.54846	5.00E-05	0.0249845
RNA-Seq	Trinity	XLOC_090936	c93386_g1_i1:0-555	cdRNA01-preDia	cdRNA02-Dia-R1	3.2646	43.8318	3.747	5.00E-05	0.0249845
RNA-Seq	Trinity	XLOC_090982	c93429_g1_i1:0-224	cdRNA02-Dia-R1	cdRNA05-postDia	36.0374	217.187	2.59137	5.00E-05	0.0249845
RNA-Seq	Trinity	XLOC_091158	c93592_g1_i1:0-756	cdRNA04-Dia-R3	cdRNA05-postDia	14.0763	67.6521	2.26487	5.00E-05	0.0249845

RNA-Seq	Trinity	XLOC_091732	c9412_g1_i2:0-1624	cdRNA01-preDia	cdRNA05-postDia	20.0352	139.073	2.79523	5.00E-05	0.0249845
RNA-Seq	Trinity	XLOC_091733	c9412_g1_i3:0-1941	cdRNA01-preDia	cdRNA05-postDia	12.2842	66.9764	2.44685	5.00E-05	0.0249845
RNA-Seq	Trinity	XLOC_091766	c94160_g1_i1:1-796	cdRNA01-preDia	cdRNA05-postDia	5.34869	30.2115	2.49784	5.00E-05	0.0249845
RNA-Seq	Trinity	XLOC_091927	c94316_g1_i1:0-752	cdRNA01-preDia	cdRNA05-postDia	18.4588	87.2733	2.24123	5.00E-05	0.0249845
RNA-Seq	Trinity	XLOC_091927	c94316_g1_i1:0-752	cdRNA02-Dia-R1	cdRNA05-postDia	10.4807	87.2733	3.05781	5.00E-05	0.0249845
RNA-Seq	Trinity	XLOC_091927	c94316_g1_i1:0-752	cdRNA04-Dia-R3	cdRNA05-postDia	10.4441	87.2733	3.06286	5.00E-05	0.0249845
RNA-Seq	Trinity	XLOC_091927	c94316_g1_i1:0-752	cdRNA03-Dia-R2	cdRNA05-postDia	9.86213	87.2733	3.14557	5.00E-05	0.0249845
RNA-Seq	Trinity	XLOC_092068	c94449_g1_i1:42-292	cdRNA01-preDia	cdRNA05-postDia	18.8161	145.051	2.94652	5.00E-05	0.0249845
RNA-Seq	Trinity	XLOC_092104	c94481_g1_i1:0-265	cdRNA02-Dia-R1	cdRNA05-postDia	100.433	688.429	2.77707	5.00E-05	0.0249845
RNA-Seq	Trinity	XLOC_092104	c94481_g1_i1:0-265	cdRNA04-Dia-R3	cdRNA05-postDia	99.3135	688.429	2.79325	5.00E-05	0.0249845
RNA-Seq	Trinity	XLOC_092104	c94481_g1_i1:0-265	cdRNA03-Dia-R2	cdRNA05-postDia	80.3312	688.429	3.09928	5.00E-05	0.0249845
RNA-Seq	Trinity	XLOC_092104	c94481_g1_i1:0-265	cdRNA01-preDia	cdRNA05-postDia	61.3322	688.429	3.48859	5.00E-05	0.0249845
RNA-Seq	Trinity	XLOC_092110	c94487_g1_i1:0-1423	cdRNA04-Dia-R3	cdRNA05-postDia	15.7024	2.58291	-2.60392	5.00E-05	0.0249845
RNA-Seq	Trinity	XLOC_092110	c94487_g1_i1:0-1423	cdRNA03-Dia-R2	cdRNA05-postDia	15.566	2.58291	-2.59133	5.00E-05	0.0249845
RNA-Seq	Trinity	XLOC_092253	c9461_g1_i1:2-1684	cdRNA04-Dia-R3	cdRNA05-postDia	12.0019	1.86064	-2.6894	0.0001	0.0432976
RNA-Seq	Trinity	XLOC_092253	c9461_g1_i1:2-1684	cdRNA03-Dia-R2	cdRNA05-postDia	11.9041	1.86064	-2.67759	0.0001	0.0432976
RNA-Seq	Trinity	XLOC_092391	c9474_g1_i1:3-899	cdRNA01-preDia	cdRNA02-Dia-R1	22.155	1.03209	-4.42399	5.00E-05	0.0249845
RNA-Seq	Trinity	XLOC_092391	c9474_g1_i1:3-899	cdRNA01-preDia	cdRNA05-postDia	22.155	2.24616	-3.3021	5.00E-05	0.0249845
RNA-Seq	Trinity	XLOC_092429	c94787_g1_i1:0-448	cdRNA04-Dia-R3	cdRNA05-postDia	8.23277	49.327	2.58293	5.00E-05	0.0249845
RNA-Seq	Trinity	XLOC_092429	c94787_g1_i1:0-448	cdRNA01-preDia	cdRNA05-postDia	3.07677	49.327	4.00289	5.00E-05	0.0249845
RNA-Seq	Trinity	XLOC_092463	c94819_g1_i1:0-561	cdRNA03-Dia-R2	cdRNA05-postDia	30.6081	4.80038	-2.67269	5.00E-05	0.0249845
RNA-Seq	Trinity	XLOC_092463	c94819_g1_i1:0-561	cdRNA04-Dia-R3	cdRNA05-postDia	28.5596	4.80038	-2.57275	5.00E-05	0.0249845
RNA-Seq	Trinity	XLOC_092621	c94969_g1_i1:0-529	cdRNA01-preDia	cdRNA05-postDia	8.51044	46.3534	2.44537	5.00E-05	0.0249845
RNA-Seq	Trinity	XLOC_092621	c94969_g1_i1:0-529	cdRNA03-Dia-R2	cdRNA05-postDia	7.03993	46.3534	2.71904	5.00E-05	0.0249845
RNA-Seq	Trinity	XLOC_092621	c94969_g1_i1:0-529	cdRNA02-Dia-R1	cdRNA05-postDia	5.67693	46.3534	3.02949	5.00E-05	0.0249845
RNA-Seq	Trinity	XLOC_093138	c95457_g1_i1:0-847	cdRNA03-Dia-R2	cdRNA05-postDia	55.0318	7.68587	-2.83998	5.00E-05	0.0249845
RNA-Seq	Trinity	XLOC_093138	c95457_g1_i1:0-847	cdRNA01-preDia	cdRNA05-postDia	48.1168	7.68587	-2.64626	5.00E-05	0.0249845
RNA-Seq	Trinity	XLOC_093138	c95457_g1_i1:0-847	cdRNA04-Dia-R3	cdRNA05-postDia	46.2146	7.68587	-2.58807	5.00E-05	0.0249845
RNA-Seq	Trinity	XLOC_093138	c95457_g1_i1:0-847	cdRNA02-Dia-R1	cdRNA05-postDia	36.238	7.68587	-2.23722	5.00E-05	0.0249845
RNA-Seq	Trinity	XLOC_093174	c9548_g1_i1:0-1100	cdRNA01-preDia	cdRNA04-Dia-R3	3.17521	30.8387	3.27982	5.00E-05	0.0249845
RNA-Seq	Trinity	XLOC_093174	c9548_g1_i1:0-1100	cdRNA01-preDia	cdRNA03-Dia-R2	3.17521	34.0335	3.42203	5.00E-05	0.0249845
RNA-Seq	Trinity	XLOC_093236	c95550_g1_i1:0-570	cdRNA01-preDia	cdRNA05-postDia	5.5059	49.5457	3.16971	5.00E-05	0.0249845
RNA-Seq	Trinity	XLOC_093308	c95612_g1_i1:0-1029	cdRNA01-preDia	cdRNA05-postDia	1.34107	9.4203	2.81239	5.00E-05	0.0249845
RNA-Seq	Trinity	XLOC_093308	c95612_g1_i1:0-1029	cdRNA01-preDia	cdRNA03-Dia-R2	1.34107	11.7629	3.13279	5.00E-05	0.0249845
RNA-Seq	Trinity	XLOC_093308	c95612_g1_i1:0-1029	cdRNA01-preDia	cdRNA04-Dia-R3	1.34107	11.8074	3.13824	5.00E-05	0.0249845
RNA-Seq	Trinity	XLOC_093564	c95853_g1_i1:0-318	cdRNA01-preDia	cdRNA05-postDia	8.83364	76.4201	3.11287	0.0001	0.0432976
RNA-Seq	Trinity	XLOC_093635	c9591_g1_i1:0-1170	cdRNA01-preDia	cdRNA05-postDia	8.67176	68.9421	2.99099	5.00E-05	0.0249845
RNA-Seq	Trinity	XLOC_093635	c9591_g1_i1:0-1170	cdRNA03-Dia-R2	cdRNA05-postDia	8.04496	68.9421	3.09923	5.00E-05	0.0249845
RNA-Seq	Trinity	XLOC_093635	c9591_g1_i1:0-1170	cdRNA04-Dia-R3	cdRNA05-postDia	7.72977	68.9421	3.15689	5.00E-05	0.0249845
RNA-Seq	Trinity	XLOC_093635	c9591_g1_i1:0-1170	cdRNA02-Dia-R1	cdRNA05-postDia	6.16126	68.9421	3.48409	5.00E-05	0.0249845
RNA-Seq	Trinity	XLOC_093645	c95930_g1_i1:0-579	cdRNA01-preDia	cdRNA02-Dia-R1	34.1392	1.74582	-4.28945	5.00E-05	0.0249845
RNA-Seq	Trinity	XLOC_093833	c96102_g1_i1:0-345	cdRNA04-Dia-R3	cdRNA05-postDia	12.0153	74.6814	2.63587	0.0001	0.0432976
RNA-Seq	Trinity	XLOC_093833	c96102_g1_i1:0-345	cdRNA03-Dia-R2	cdRNA05-postDia	10.2821	74.6814	2.86061	0.0001	0.0432976
RNA-Seq	Trinity	XLOC_093833	c96102_g1_i1:0-345	cdRNA01-preDia	cdRNA05-postDia	10.0207	74.6814	2.89777	5.00E-05	0.0249845
RNA-Seq	Trinity	XLOC_093931	c96192_g1_i1:0-279	cdRNA03-Dia-R2	cdRNA05-postDia	19.3304	120.594	2.64122	0.0001	0.0432976
RNA-Seq	Trinity	XLOC_093931	c96192_g1_i1:0-279	cdRNA04-Dia-R3	cdRNA05-postDia	19.0374	120.594	2.66326	5.00E-05	0.0249845
RNA-Seq	Trinity	XLOC_093931	c96192_g1_i1:0-279	cdRNA02-Dia-R1	cdRNA05-postDia	18.2277	120.594	2.72596	5.00E-05	0.0249845

RNA-Seq	Trinity	XLOC_094082	c96331_g1_i1:0-456	cdRNA03-Dia-R2	cdRNA05-postDia	5.79188	52.822	3.18903	5.00E-05	0.0249845
RNA-Seq	Trinity	XLOC_094082	c96331_g1_i1:0-456	cdRNA04-Dia-R3	cdRNA05-postDia	5.56496	52.822	3.24669	5.00E-05	0.0249845
RNA-Seq	Trinity	XLOC_094109	c96358_g1_i1:0-785	cdRNA02-Dia-R1	cdRNA05-postDia	8.55368	43.1475	2.33466	0.0001	0.0432976
RNA-Seq	Trinity	XLOC_094252	c96493_g1_i1:0-1058	cdRNA04-Dia-R3	cdRNA05-postDia	38.2139	4.99356	-2.93596	5.00E-05	0.0249845
RNA-Seq	Trinity	XLOC_094252	c96493_g1_i1:0-1058	cdRNA03-Dia-R2	cdRNA05-postDia	36.5387	4.99356	-2.87128	5.00E-05	0.0249845
RNA-Seq	Trinity	XLOC_094524	c96749_g1_i1:0-229	cdRNA01-preDia	cdRNA03-Dia-R2	23.1324	190.084	3.03866	0.0001	0.0432976
RNA-Seq	Trinity	XLOC_094524	c96749_g1_i1:0-229	cdRNA01-preDia	cdRNA04-Dia-R3	23.1324	200.559	3.11605	0.0001	0.0432976
RNA-Seq	Trinity	XLOC_094785	c9699_g1_i1:0-298	cdRNA01-preDia	cdRNA05-postDia	12.0876	102.413	3.0828	5.00E-05	0.0249845
RNA-Seq	Trinity	XLOC_094850	c97058_g1_i1:0-1735	cdRNA03-Dia-R2	cdRNA05-postDia	6.59632	44.5933	2.75709	5.00E-05	0.0249845
RNA-Seq	Trinity	XLOC_094850	c97058_g1_i1:0-1735	cdRNA04-Dia-R3	cdRNA05-postDia	6.04079	44.5933	2.88402	5.00E-05	0.0249845
RNA-Seq	Trinity	XLOC_094850	c97058_g1_i1:0-1735	cdRNA01-preDia	cdRNA05-postDia	5.21347	44.5933	3.09651	5.00E-05	0.0249845
RNA-Seq	Trinity	XLOC_094850	c97058_g1_i1:0-1735	cdRNA02-Dia-R1	cdRNA05-postDia	4.06247	44.5933	3.4564	5.00E-05	0.0249845
RNA-Seq	Trinity	XLOC_095094	c9729_g1_i1:0-222	cdRNA04-Dia-R3	cdRNA05-postDia	126.743	795.638	2.65021	5.00E-05	0.0249845
RNA-Seq	Trinity	XLOC_095094	c9729_g1_i1:0-222	cdRNA03-Dia-R2	cdRNA05-postDia	122.067	795.638	2.70444	5.00E-05	0.0249845
RNA-Seq	Trinity	XLOC_095094	c9729_g1_i1:0-222	cdRNA01-preDia	cdRNA05-postDia	118.323	795.638	2.74938	5.00E-05	0.0249845
RNA-Seq	Trinity	XLOC_095539	c97717_g1_i1:0-692	cdRNA01-preDia	cdRNA05-postDia	13.1981	68.1123	2.36759	0.0001	0.0432976
RNA-Seq	Trinity	XLOC_095927	c98083_g1_i1:0-545	cdRNA02-Dia-R1	cdRNA05-postDia	11.5193	60.541	2.39386	0.0001	0.0432976
RNA-Seq	Trinity	XLOC_096375	c9849_g1_i2:0-825	cdRNA02-Dia-R1	cdRNA05-postDia	13.1302	77.5277	2.56182	5.00E-05	0.0249845
RNA-Seq	Trinity	XLOC_096375	c9849_g1_i2:0-825	cdRNA01-preDia	cdRNA05-postDia	4.6449	77.5277	4.06099	5.00E-05	0.0249845
RNA-Seq	Trinity	XLOC_096397	c98519_g1_i1:7-473	cdRNA04-Dia-R3	cdRNA05-postDia	76.8166	12.017	-2.67634	5.00E-05	0.0249845
RNA-Seq	Trinity	XLOC_096397	c98519_g1_i1:7-473	cdRNA03-Dia-R2	cdRNA05-postDia	70.7646	12.017	-2.55795	5.00E-05	0.0249845
RNA-Seq	Trinity	XLOC_096420	c98538_g1_i1:0-1099	cdRNA01-preDia	cdRNA03-Dia-R2	11.214	1.45454	-2.94666	5.00E-05	0.0249845
RNA-Seq	Trinity	XLOC_096420	c98538_g1_i1:0-1099	cdRNA01-preDia	cdRNA04-Dia-R3	11.214	1.56695	-2.83926	0.0001	0.0432976
RNA-Seq	Trinity	XLOC_096420	c98538_g1_i1:0-1099	cdRNA04-Dia-R3	cdRNA05-postDia	1.56695	14.6061	3.22054	5.00E-05	0.0249845
RNA-Seq	Trinity	XLOC_096420	c98538_g1_i1:0-1099	cdRNA03-Dia-R2	cdRNA05-postDia	1.45454	14.6061	3.32794	5.00E-05	0.0249845
RNA-Seq	Trinity	XLOC_096557	c98670_g1_i1:0-349	cdRNA03-Dia-R2	cdRNA05-postDia	7.57441	62.3126	3.04032	5.00E-05	0.0249845
RNA-Seq	Trinity	XLOC_096557	c98670_g1_i1:0-349	cdRNA04-Dia-R3	cdRNA05-postDia	7.27765	62.3126	3.09798	5.00E-05	0.0249845
RNA-Seq	Trinity	XLOC_096558	c98671_g1_i1:0-536	cdRNA01-preDia	cdRNA03-Dia-R2	2.97536	45.9829	3.94996	5.00E-05	0.0249845
RNA-Seq	Trinity	XLOC_096558	c98671_g1_i1:0-536	cdRNA01-preDia	cdRNA04-Dia-R3	2.97536	51.1454	4.10347	5.00E-05	0.0249845
RNA-Seq	Trinity	XLOC_096631	c9873_g1_i1:0-499	cdRNA04-Dia-R3	cdRNA05-postDia	3.08357	29.1812	3.24237	5.00E-05	0.0249845
RNA-Seq	Trinity	XLOC_097026	c99115_g1_i1:1-1066	cdRNA03-Dia-R2	cdRNA05-postDia	9.23365	99.0054	3.42253	5.00E-05	0.0249845
RNA-Seq	Trinity	XLOC_097026	c99115_g1_i1:1-1066	cdRNA04-Dia-R3	cdRNA05-postDia	9.54284	99.0054	3.37502	5.00E-05	0.0249845
RNA-Seq	Trinity	XLOC_097289	c99359_g1_i1:0-394	cdRNA01-preDia	cdRNA04-Dia-R3	22.2387	110.649	2.31485	0.0001	0.0432976
RNA-Seq	Trinity	XLOC_097469	c99527_g1_i1:0-863	cdRNA01-preDia	cdRNA05-postDia	14.2704	66.7072	2.22481	0.0001	0.0432976
RNA-Seq	Trinity	XLOC_097471	c9952_g1_i1:0-1093	cdRNA03-Dia-R2	cdRNA05-postDia	10.4724	1.25528	-3.06051	5.00E-05	0.0249845
RNA-Seq	Trinity	XLOC_097471	c9952_g1_i1:0-1093	cdRNA04-Dia-R3	cdRNA05-postDia	8.99617	1.25528	-2.8413	0.0001	0.0432976
RNA-Seq	Trinity	XLOC_097824	c99863_g1_i1:0-948	cdRNA01-preDia	cdRNA05-postDia	3.08036	18.749	2.60564	5.00E-05	0.0249845

Supplementary Table S2. Differentially expressed genes in diapause versus pre- or post-diapause.

NGS	assembler	gene_id	transcript_length	homolog_gene_id	homolog_transcript_length	identities	e_value	product_name	gene_aliases	gene_ontology_go	go_id	go_name	gene_cluster	rpkm_RNA01-preDia	rpkm_RNA02-Dia-R1	rpkm_RNA03-Dia-R2	rpkm_RNA04-Dia-R3	rpkm_RNA05-postDia	Log2(Dia-R1/preDia)	Log2(Dia-R2/preDia)	Log2(Dia-R3/preDia)	Log2(Dia-R1/postDia)	Log2(Dia-R2/postDia)	Log2(Dia-R3/postDia)
WGS	ABySS	XLOC_026778	2196	XM_008279397.1	2213	737/953	0	UDP-GlcNAc:betaGal beta-1,3-N-acetylglucosaminyltransferase 3	b3gnt3, tmem3	molecular_function	GO:0003978	transferase activity	Carbohydrate Modification related	0.780905	1.04137	1.13019	1.43872	11.5748	0.415263796	0.533346374	0.881566891	-3.474432609	-3.356350031	-3.008129515
WGS	ABySS	XLOC_037689	1753	XM_010749444.1	1754	1089/1349	0	acidic mammalian chitinase-like	chia, amcase	molecular_function	GO:0004568	chitinase activity	Carbohydrate Modification related	10.7517	127.069	67.5815	70.2286	361.499	3.562975417	2.652063583	2.707493887	-1.508379546	-2.419291379	-2.363861076
WGS	ABySS	XLOC_039760	1804	XM_008414270.1	1890	1072/1427	0	acidic mammalian chitinase-like	chia, amcase	molecular_function	GO:0004568	chitinase activity	Carbohydrate Modification related	22.8397	69.8326	59.5896	57.7154	609.283	1.612356987	1.383516862	1.337412619	-3.125139894	-3.353980019	-3.400084263
RNA-Seq	Trinity	XLOC_055003	1550	XM_010749444.1	1754	1089/1349	0	acidic mammalian chitinase-like	chia, amcase	molecular_function	GO:0004568	chitinase activity	Carbohydrate Modification related	13.416	165.731	80.5687	86.2262	518.208	3.626816985	2.586264883	2.684171708	-1.644687804	-2.685239905	-2.587333081
RNA-Seq	Trinity	XLOC_022639	680	XM_007574441.1	718	99/133	2.00E-13	galactose-specific lectin nattectin-like	ctl	molecular_function	GO:0005534	galactose binding	Carbohydrate Modification related	72.6346	1.37539	4.97248	4.77766	0.418641	-5.722744283	-3.868619476	-3.926280856	1.716055246	3.570180053	3.512518673
WGS	ABySS	XLOC_058665	566	XM_005477871.1	644	268/406	3.00E-11	galactose-specific lectin nattectin-like	ctl	molecular_function	GO:0005534	galactose binding	Carbohydrate Modification related	87.8898	3.86292	11.5147	11.7137	0.690517	-4.507932039	-2.93221892	-2.907498893	2.483942962	4.059656081	4.084376108
RNA-Seq	Trinity	XLOC_074600	761	XM_005477871.1	644	348/532	4.00E-17	galactose-specific lectin nattectin-like	ctl	molecular_function	GO:0005534	galactose binding	Carbohydrate Modification related	107.963	4.5243	12.5863	14.1222	1.0241	-4.576698565	-3.10061083	-2.934500212	2.143337999	3.619425734	3.785536352
RNA-Seq	Trans-ABySS	XLOC_109339	662	XM_005477871.1	644	348/532	4.00E-17	galactose-specific lectin nattectin-like	ctl	molecular_function	GO:0005534	galactose binding	Carbohydrate Modification related	126.869	5.20719	14.5119	16.2184	1.16172	-4.606690736	-3.12803127	-2.96763619	2.164242659	3.642902125	3.803297205
RNA-Seq	Trans-ABySS	XLOC_151142	667	XM_007574441.1	718	99/133	2.00E-13	galactose-specific lectin nattectin-like	ctl	molecular_function	GO:0005534	galactose binding	Carbohydrate Modification related	86.1945	1.59607	5.78062	5.43995	0.478824	-5.754999984	-3.898299673	-3.985930519	1.736956555	3.593656866	3.50602602
RNA-Seq	Trans-ABySS	XLOC_019418	524	XM_007542092.1	2010	335/487	2.00E-33	flocculation protein FLO11-like	flo11	biological_process	GO:0000501	flocculation via cell wall protein-carbohydrate interaction	Carbohydrate Modification related	13.6826	6.61296	7.38796	7.20381	48.911	-1.04897432	-0.88909444	-0.925510365	-2.886790881	-2.726911001	-2.763326925
RNA-Seq	Trinity	XLOC_008085	1226	XM_008284196.1	1554	923/1068	0	UDP-galactose-4-epimerase	gale	molecular_function	GO:0004842	UDP-glucose 4-epimerase activity	Carbohydrate Modification related	8.85388	3.26125	4.36177	4.45049	27.8051	-1.440884781	-1.021396123	-0.992345634	-3.091852582	-2.672363923	-2.643313435
WGS	ABySS	XLOC_019303	799	XM_008284197.1	1551	231/267	3.00E-83	UDP-galactose-4-epimerase	gale	molecular_function	GO:0003978	UDP-glucose 4-epimerase activity	Carbohydrate Modification related	12.3591	6.04432	7.58597	6.71653	65.0461	-1.031921742	-0.704168117	-0.879785706	-3.427810612	-3.100056988	-3.275674577
RNA-Seq	Trinity	XLOC_071640	1355	XM_007570971.1	1784	976/1121	0	GDP-mannose 4,6-dehydratase	gmds	molecular_function	GO:0008446	GDP-mannose 4,6-dehydratase activity	Carbohydrate Modification related	5.08085	3.7231	3.66728	3.16136	24.3425	-0.448565506	-0.470359451	-0.684524541	-2.70890107	-2.730695016	-2.944860105
RNA-Seq	Trans-ABySS	XLOC_009204	934	AB665331.1	995	492/671	3.00E-108	pentraxin-2	nptx2, narp, np2	molecular_function	GO:0004867	carbohydrate binding	Carbohydrate Modification related	488.399	101.323	94.7147	90.5266	4.79839	-2.269098548	-2.366399988	-2.431646571	4.400267468	4.302966028	4.237719445
RNA-Seq	Trinity	XLOC_069671	921	AB665331.1	995	492/671	3.00E-108	pentraxin-2	nptx2, narp, np2	molecular_function	GO:0030246	carbohydrate binding	Carbohydrate Modification related	430.02	91.559	82.3086	77.5508	5.33624	-2.231630149	-2.385288677	-2.471190191	4.100806247	3.947147719	3.861246205
RNA-Seq	Trinity	XLOC_004218	1675	XM_005813857.1	1587	464/555	1.00E-164	UDP-glucuronosyltransferase 2B31-like	ugt2b17	molecular_function	GO:0016740	glucuronosyltransferase activity	Carbohydrate Modification related	14.1029	16.629	14.8211	15.0619	1.21703	0.237709557	0.07166067	0.094911915	3.772264777	3.60621589	3.629467136
RNA-Seq	Trans-ABySS	XLOC_014429	1679	XM_005813857.1	1587	1237/1563	0	UDP-glucuronosyltransferase 2B31-like	ugt2b17	molecular_function	GO:0015020	glucuronosyltransferase activity	Carbohydrate Modification related	20.0375	23.2971	21.7747	21.8807	1.50305	0.21744786	0.119950322	0.126956373	3.954185473	3.856687935	3.863693986
RNA-Seq	Trans-ABySS	XLOC_103444	452	XM_008404090.1	2141	337/458	3.00E-73	UDP-glucuronosyltransferase-like	ugt2b17	molecular_function	GO:0015020	glucuronosyltransferase activity	Carbohydrate Modification related	22.6202	23.2958	26.6197	23.7542	112.275	0.04245819	0.234882627	0.070570935	-2.268894942	-2.076470505	-2.240782197
WGS	ABySS	XLOC_050849	2595	XM_008301011.1	3273	1598/2194	0	BCL2-associated athanogene 3	bag3	biological_process	GO:0060047	heart contraction	Cardiac and Skeletal Muscle related	11.3618	14.7037	57.8181	55.0261	9.15053	0.371987824	2.347329787	2.275924668	0.684252025	2.659593988	2.588188869
WGS	ABySS	XLOC_001268	67	XM_011493673.1	2643	63/65	6.00E-21	myosin heavy chain, fast skeletal muscle-like	myh2, myhsa2, mhc IIa, myh3	biological_process	GO:0060048	cardiac muscle contraction	Cardiac and Skeletal Muscle related	118140	112133	100335	88927.3	16581.8	-0.075286601	-0.235672566	-0.409799229	2.757538387	2.597152422	2.42302576
RNA-Seq	Trinity	XLOC_029329	713	XM_007548725.1	4057	449/640	8.00E-73	myosin light chain kinase 2, skeletal/cardiac muscle-like	mylk2, mlck	biological_process	GO:0060048	cardiac muscle contraction	Cardiac and Skeletal Muscle related	2.87228	3.44942	1.16253	2.45736	19.575	0.264157406	-1.304928448	-0.225087169	-2.504586601	-4.073672456	-2.993831176
WGS	ABySS	XLOC_022756	2532	XM_008280772.1	5551	2142/2540	0	myosin XVIIIB	myo18b	biological_process	GO:0048739	cardiac muscle fiber development	Cardiac and Skeletal Muscle related	10.0026	25.3167	69.9431	67.7842	4.60838	1.339714312	2.805806688	2.760573979	2.457757774	3.923850151	3.878617442
WGS	ABySS	XLOC_022757	2139	XM_008280772.1	5551	1852/2120	0	myosin XVIIIB	myo18b	biological_process	GO:0048739	cardiac muscle fiber development	Cardiac and Skeletal Muscle related	13.5261	34.7455	113.321	115.719	13.0337	1.361080217	3.066597408	3.096807931	1.414579448	3.120096639	3.150307163
WGS	ABySS	XLOC_060894	570	XM_008280772.1	5551	456/629	1.00E-91	myosin XVIIIB	myo18b	biological_process	GO:0048739	cardiac muscle fiber development	Cardiac and Skeletal Muscle related	18.6569	42.902	113.626	117.879	10.1103	1.201335614	2.606511796	2.659525531	2.085219098	3.49039528	3.543409015
WGS	ABySS	XLOC_003476	1382	XM_008280771.1	6805	915/1122	0	myosin-XVIIIb	myo18b	biological_process	GO:0038195	cardiac muscle fiber development	Cardiac and Skeletal Muscle related	14.0068	32.3879	102.72	92.5226	10.4072	1.209327534	2.874517808	2.723678413	1.637872957	3.303063231	3.152223836
WGS	ABySS	XLOC_016872	1107	XM_005795793.1	2103	917/1105	0	cirhin-like	cirh1a, te292	molecular_function	GO:0005515	protein binding	Cell Signaling related	11.4217	19.2047	63.1924	56.5183	11.9492	0.749682031	2.467973663	2.306940675	0.684545395	2.402837026	2.241804039
WGS	ABySS	XLOC_019695	2540	XM_008276979.1	2446	1094/1261	0	F-box protein 32	fbxo32, mafbx, atrogin1	molecular_function	GO:0005515	protein binding	Cell Signaling related	7.17055	29.3549	75.7021	78.3115	15.2043	2.033445655	3.400177635	3.449068495	0.949121945	2.315853925	2.364744785
WGS	ABySS	XLOC_056679	2259	XM_007552552.1	2770	1545/1890	0	hedgehog acyltransferase-like	hhat, mart2, ski1	molecular_function	GO:0005515	protein binding	Cell Signaling related	51.2101	21.3271	8.99019	8.63788	48.4374	-1.263740571	-2.510004865	-2.567679197	-1.183433621	-2.429697915	-2.487372247
RNA-Seq	Trans-ABySS	XLOC_009126	137	XM_007551843.1	3173	113/135	9.00E-31	putative GPI-anchored glycoprotein PJ4664.02-like	n/a	biological_process	GO:0018149	cytoplasmic membrane-bounded vesicle	Cell Signaling related	361.281	734.168	857.184	807.815	5398.38	1.022988849	1.246483537	1.160903551	-2.878344395	-2.654849707	-2.740429694
RNA-Seq	Trans-ABySS	XLOC_010058	153	XM_005458737.1	2888	39/48	0.64	putative GPI-anchored protein PB15E9.01c-like	n/a	cellular_component	GO:0016023	cytoplasmic membrane-bounded vesicle	Cell Signaling related	130.616	169.945	200.135	187.458	1439.09	0.379736284	0.615641858	0.521235763	-3.082016999	-2.846111425	-2.94051752
RNA-Seq	Trinity	XLOC_043815	614	XM_008413658.1	2921	176/252	8.00E-18	putative GPI-anchored protein PB15E9.01c	n/a	cellular_component	GO:0016023	cytoplasmic membrane-bounded vesicle	Cell Signaling related	36.14	40.0649	41.4687	42.34	230.67	0.14874237	0.198426315	0.228424763	-2.525419604	-2.475735659	-2.445737211
WGS	ABySS	XLOC_028859	1101	XM_008413658.1	2921	176/252	1.00E-17	putative GPI-anchored protein PB15E9.01c	n/a	cellular_component	GO:0016023	cytoplasmic membrane-bounded vesicle	Cell Signaling related	20.1371	25.52	24.7868	28.1521	173.39	0.341772397	0.299716098	0.483386612	-2.764320462	-2.806376761	-2.622706247
RNA-Seq	Trinity	XLOC_031868	464	XM_008413658.1	2921	84/120	0.05	putative GPI-anchored protein PB15E9.01c	n/a	cellular_component	GO:0016023	cytoplasmic membrane-bounded vesicle	Cell Signaling related	18.9963	22.8906	23.8202	24.3447	139.948	0.269036833	0.326467081	0.357889277	-2.612063686	-2.554633438	-2.523211242
WGS	ABySS	XLOC_028937	897	XM_006794753.1	1499	261/386	2.00E-16	urokinase plasminogen activator surface receptor-like	plaur, upar, cd87	biological_process	GO:0038195	urokinase plasminogen activator signaling pathway	Cell Signaling related	4.4422	13.8204	12.0923	11.1071	70.3443	1.637453118	1.444742423	1.322135933	-2.347634155	-2.54034485	-2.66295134
RNA-Seq	Trinity	XLOC_055599	571	XM_005797393.1	2132	140/172	4.00E-37	14-3-3 protein beta/alpha-like	ywhab	molecular_function	GO:0005515	protein binding	Cell Signaling related	180.446	308.691	626.224	615.528	53.1029	0.774596258	1.795111638	1.770257323	2.539300866	3.559816245	3.53496193
RNA-Seq	Trinity	XLOC_081441	635	AF508263.1	1186	590/635	0	actin	acta1, asma	cellular_component	GO:0005856	cytoskeleton	Cytoskeleton related	32.729	0.69045	1.18985	1.14324	2.95904	-5.56688877	-4.781717908	-4.839369318	-2.09952035	-1.314349489	-1.372000899
RNA-Seq	Trinity	XLOC_060688	299	XM_008294975.1	1246	280/299	3.00E-123	actin, alpha cardiac	actc1	cellular_component	GO:0005856	cytoskeleton	Cytoskeleton related	130.568	51.4226	37.8704	47.1112	402.339	-1.344326898	-1.785658795	-1.470659375	-2.967937128	-3.409269025	-3.094269604
WGS	ABySS	XLOC_059905	2035	XM_008289217.1	1952	1000/1300	0	cytoskeleton-associated protein 4	ckap4, ergic-63, p63, climp-63	cellular_component	GO:0005856	cytoskeleton	Cytoskeleton related	3.88502	1.29666	1.89556	1.51729	15.5372	-1.583121789	-1.035297903	-1.356425171	-3.582854393	-3.035030507	-3.356157776
RNA-Seq	Trinity	XLOC_075256	1200	XM_008289217.1	1952	686/918	2.00E-169	cytoskeleton-associated protein 4	ckap4, ergic-63, p63, climp-63	cellular_component	GO:0005856	cytoskeleton	Cytoskeleton related	6.42694	2.00185	2.29723	2.28333	23.9291	-1.682798128	-1.484236696	-1.492992623	-3.579360355	-3.380798924	-3.38955485
RNA-Seq	Trans-ABySS	XLOC_144332	708	XM_008289217.1	1952	541/695	1.00E-155	cytoskeleton-associated protein 4	ckap4, ergic-63, p63, climp-63	cellular_component	GO:0005856	cytoskeleton	Cytoskeleton related	6.51562	2.66588	2.56669	3.13398	24.7209	-1.289290625	-1.343993406	-1.055906493	-3.213047521	-3.267750302	-2.979663389
WGS	ABySS	XLOC_021426	3735	XM_008294644.1	4144	2698/3354	0	filamin A interacting protein 1	filip1, filip1l	cellular_component	GO:0005856	cytoskeleton	Cytoskeleton related	5.97128	9.21074	26.0478	26.3035	5.53075	0.625276849	2.125049402	2.139142655	0.735841937	2.235614491	2.249707744
RNA-Seq	Trinity	XLOC_078652	651	XM_007552802.1	3079	203/258	3.00E-49	actin cytoskeleton-regulatory complex protein PAN1-like	nlrp2, pan1, nap2, pypaf2	cellular_component	GO:0005856	cytoskeleton	Cytoskeleton related	9.10483	7.65597	9.44288	9.41209	52.1587	-0.250046904	0.052594856	0.047883036	-2.768250831	-2.465609071	-2.470320891
WGS	ABySS	XLOC_053262	930	XM_008399234.1	3493	714/872	0	plastin-1-like	pls1, fimbrin	molecular_function	GO:0008134	actin filament binding	Cytoskeleton related	4.97253	8.6477	6.54897	6.21294	44.2075	0.7983364	0.397287947	0.321296047	-2.35390277	-2.754951223	-2.830943123
RNA-Seq	Trinity	XLOC_075624	855	XM_008399233.1	3478	674/812	0	plastin-1-like	pls1, fimbrin	molecular_function	GO:0051015	actin filament binding	Cytoskeleton related	5.28061	8.86818	6.66567	5.70584	35.3858	0.747933458	0.3360453	0.111734697	-1.996460578	-2.408348736	-2.632659339
RNA-Seq	Trans-ABySS	XLOC_000264	539	XM_008431347.1	1612	499/539	0	tubulin, beta class I	tubb	molecular_function	GO:0004252	structural constitutent of cytoskeleton	Cytoskeleton related	149.488	57.9548	26.0982	33.0046	169.281	-1.367029618	-2.518007466	-2.17929066	-1.546419995	-2.697397843	-2.358681037
RNA-Seq	Trinity	XLOC_033784	221	XM_008431347.1	1612	204/220	5.00E-85	tubulin, beta class I	tubb	molecular_function	GO:0005200	structural constitutent of cytoskeleton	Cytoskeleton related	537.548	236.723	96.9395	128.67	708.788	-1.1831937	-2.471237039	-2.062717863	-1.582154294	-2.870197632	-2.461678457
WGS	ABySS	XLOC_022322	1721	XM_008294471.1	1837	346/521	2.00E-29	ADM-like	adm	biological_process	GO:0048589	developmental growth	Developmental related	1.10745	1.09534	1.37638	1.10149	12.0753	-0.015862804	0.313637269	-0.007785167	-3.462608366	-3.133108293	-3.454530729
WGS	ABySS	XLOC_032093	971	NM_001279646.1	2657	218/305	6.00E-32	seminal plasma glycoprotein 120	bsph1, spp120	molecular_function	GO:0003824	zygote formation	Developmental related	4.81928	11.8163	3.29896	2.85743	67.9495	1.293888831	-0.546806339	-0.754099468	-2.523684575	-4.364379745	-4.571672874
RNA-Seq	Trinity	XLOC_044094	843	NM_001279646.1	2657	218/305	8.00E-33	seminal plasma glycoprotein 120	bsph1, spp120	biological_process	GO:0007338	zygote formation	Developmental related	6.12181	16.0669	4.56839	3.67761	119.003	1.392061423	-0.422272452	-0.735189775	-2.888834441	-4.703168316	-5.016085639
RNA-Seq	Trans-ABySS	XLOC_139318	222	NM_001279646.1	2657	136/190	1.00E-17	seminal plasma glycoprotein 120	bsph1, spp120	biological_process	GO:0007338	zygote formation	Developmental related	39.8352	80.469	20.0366	25.5671	603.018	1.014389286	-0.991406099	-0.639755289	-2.905696058	-4.911491443	-4.559840633
WGS	ABySS	XLOC_007573	973	XM_004068819.2	1032	729/887	0	elastase-1-like	cela1, ela1	biological_process	GO:0051302	regulation of cell division	Developmental related	24.1615	100.534	170.345	163.616	184.901	2.056901567	2.817677673	2.759531908	-0.879069532	-0.118293426	-0.176439191
RNA-Seq	Trans-ABySS	XLOC_009586	966	XM_004068819.2	1032	709/860	0	elastase-1-like	cela1, ela1	biological_process	GO:0051302	regulation of cell division	Developmental related	20.3508	83.932	136.574	135.41	178.406	2.04413545	2.746525446	2.734176872	-1.087871272	-0.385481276	-0.39782985
RNA-Seq	Trinity	XLOC_023195	279	XM_004068819.2	1032	234/278	2.00E-78	elastase-1-like	cela1, ela1	biological_process	GO:0051302	regulation of cell division	Developmental related	108.428	473.191	745.186	782.853	1227.92	2.125685274	2.78086321	2.852004071	-1.375722032	-0.720544096	-0.649403235
RNA-Seq	Trinity	XLOC_046401	1059	XM_011489102.1	2682	647/868	3.00E-157	trithorax group protein osa-like	eed, wait1	cellular_component	GO:0005887	photoreceptor cell differentiation	Developmental related	26.5273	9.22349	5.25984	6.11772	41.7197	-1.524093194	-2.334387023	-2.116411859	-2.177344135	-2.987637964	-2.7696628
RNA-Seq	Trans-ABySS	XLOC_046550	774	XM_011489102.1	2682	600/797	2.00E-147	trithorax group protein osa-like	eed, wait1	biological_process	GO:0046530	photoreceptor cell differentiation	Developmental related	25.9254	9.66456	4.38248	5.84179	52.3515	-1.423590291	-2.564546836	-2.149883846	-2.437454917	-3.578411462	-3.163748472
RNA-Seq	Trinity	XLOC_020363	1754	XM_008276284.1	3513	192/253	1.00E-35	APC membrane recruitment protein 2	fam123a, amer2	biological_process	GO:0090090	negative regulation of canonical Wnt signaling pathway	Developmental related	0.892251	1.19036	2.21424	2.1764	11.4938	0.415876435	1.311290085	1.286422215	-3.271385992	-2.375972342	-2.400840212
RNA-Seq	Trans-ABySS	XLOC_037631	1222	XM_007568789.1	1496	815/1000	0	formin-like protein	fmnl1, c17orf1	biological_process	GO:0008360	regulation of cell shape	Developmental related	38.7102	8.67812	3.94371	4.40323	42.5104	-2.157259321	-3.295088389	-3.136079652	-2.292361393	-3.430190462	-3.271181724
WGS	ABySS	XLOC_039111	1422	XM_007568789.1	1496	809/993	0	formin-like protein	fmnl1, c17orf1	biological_process	GO:0008360	regulation of cell shape	Developmental related	23.2205	5.21564	2.73717	2.77063	22.8726	-2.154482839	-3.084642092	-3.067113072	-2.132704172	-3.062863425	-3.045334405
RNA-Seq	Trinity	XLOC_047644	864	XM_007568789.1	1496	713/870	0	formin-like protein	fmnl1, c17orf1	biological_process	GO:0008360	regulation of cell shape	Developmental related	36.4004	7.66512	3.64601	4.47946	47.524	-2.247574021	-3.319563881	-3.022557573	-2.632275987	-3.704265847	-3.407259539
WGS	ABySS	XLOC_030839	862	XM_005450877.1	1656	382/477	7.00E-117	insulin-like growth factor-binding protein 4-like	igfbp4, ibp4	biological_process	GO:0040008	regulation of growth	Developmental related	6.06953	17.8764	28.2273	27.8673	2.65298	1.558399522	2.217434429	2.19891652	2.752370527	3.411405434	3.392887525
RNA-Seq	Trinity	XLOC_060376	842	XM_005450877.1	1656	400/500	4.00E-123	insulin-like growth factor-binding protein 4-like	igfbp4, ibp4	biological_process	GO:0040008	regulation of growth	Developmental related	7.86554	24.8611	29.5556	37.309	4.06046	1.66027241	1.909813788	2.245905971	2.61417505	2.863716427	3.199808611
RNA-Seq	Trans-ABySS	XLOC_015919	1127	XM_005805981.1	1106	694/871	0	interleukin-12 subunit beta-like	il12b, clmf2, nksf2	biological_process	GO:0032946	positive regulation of mononuclear cell proliferation	Developmental related	32.9597	27.8769	22.763	20.767	2.20978	-0.241632973	-0.534012404	-0.666410292	3.657095487	3.364716056	3.232318169
WGS	ABySS	XLOC_036716	1342	XM_005805981.1	1106	694/871	0	interleukin-12 subunit beta-like	il12b, clmf2, nksf2	biological_process	GO:0032946	positive regulation of mononuclear cell proliferation	Developmental related	21.6184	18.5189	15.9902	15.0004	1.39918	-0.223261345	-0.435071768	-0.52725878	3.726344929	3.514534506	3.422347494
RNA-Seq	Trinity	XLOC_080417	1225	XM_005805981.1	1106	694/871	0	interleukin-12 subunit beta-like	il12b, clmf2, nksf2	biological_process	GO:0032946	positive regulation of mononuclear cell proliferation	Developmental related	26.0071	22.4722	18.4301	16.7428	1.83467	-0.210764167	-0.496841637	-0.635364717	3.614548874	3.328471403	3.189948323
WGS	ABySS	XLOC_029459	1573	XM_008296262.1	574	177/244	2.00E-28	cell death-inducing p53-target protein 1 homolog	litaf	biological_process	GO:0006915	apoptotic process	Developmental related	3.39533	2.56521	2.13207	2.48551	23.9007	-0.404474864	-0.671296995	-0.450009892	-3.219904031	-3.486726162	-3.265439059
RNA-Seq	Trans-ABySS	XLOC_015998	1059	XM_008421839.1	2153	776/951	0	matrilin 1, cartilage matrix protein	matn1, crtm, cmp	biological_process	GO:0030500	regulation of bone mineralization	Developmental related	10.6156	6.52166	8.34764	8.18855	89.924	-0.702874781	-0.346745626	-0.374506003	-3.785395076	-3.429265922	-3.457026299
RNA-Seq	Trinity	XLOC_064147	433	XM_008421839.1	2153	58/67	4.00E-11	matrilin 1, cartilage matrix protein	matn1, crtm, cmp	biological_process	GO:0030500	regulation of bone mineralization	Developmental related	20.3598	12.2148	16.5367	16.2198	212.841	-0.737093148	-0.300052025	-0.327967359	-4.12307394	-3.686032817	-3.713948151
RNA-Seq	Trinity	XLOC_071977	681	XM_008421839.1	2153	565/680	0	matrilin 1, cartilage matrix protein	matn1, crtm, cmp	biological_process	GO:0030500	regulation of bone mineralization	Developmental related	9.52626	4.80378	8.4355	7.23093	68.179	-0.987739844	-0.17543634	-0.397728715	-3.827085453	-3.01478195	-3.237074324
RNA-Seq	Trinity	XLOC_093635	1170	XM_008290482.1	1473	808/951	0	matrilin 1, cartilage matrix protein	matn1, crtm, cmp	biological_process	GO:0030500	regulation of bone mineralization	Developmental related	8.67176	6.16126	8.04496	7.72977	68.9421	-0.493099412	-0.108239581	-0.165899342	-3.484087922	-3.099228091	-3.156887852
RNA-Seq	Trans-ABySS	XLOC_108519	384	XM_008421839.1	2153	52/58	5.00E-10	matrilin 1, cartilage matrix protein	matn1, crtm, cmp	biological_process	GO:0030500	regulation of bone mineralization	Developmental related	27.5894	16.5907	22.2797	22.5066	291.833	-0.733739324	-0.308384275	-0.293765952	-4.136696367	-3.711341318	-3.696722995
RNA-Seq	Trans-ABySS	XLOC_177540	647	XM_008421839.1	2153	532/643	0	matrilin 1, cartilage matrix protein	matn1, crtm, cmp	biological_process	GO:0030500	regulation of bone mineralization	Developmental related	11.8399	5.83845	10.17	8.76922	81.3717	-1.01999958	-0.219337217	-0.433136467	-3.800869815	-3.000207452	-3.214006702
RNA-Seq	Trinity	XLOC_022347	1567	XM_008289800.1	6252	1066/1570	5.00E-151	marker of proliferation Ki-67	mki67, kia	biological_process	GO:0008283	cell proliferation	Developmental related	8.7841	3.53702	1.24454	1.50168	13.4801	-1.312360102	-2.819281879	-2.548317063	-1.930224917	-3.437146694	-3.166181879
RNA-Seq	Trans-ABySS	XLOC_009923	658	XM_008426224.1	570	455/551	9.00E-150	retinol binding protein 2, cellular	rbp2, rbpc2, crbp2	molecular_function	GO:0005524	erythrocyte development	Developmental related	67.7106	116.529	61.6049	66.9495	371.022	0.783235428	-0.136336597	-0.016308422	-1.670815699	-2.590387724	-2.470359549
WGS	ABySS	XLOC_028363	763	XM_008426224.1	570	409/493	2.00E-135	retinol binding protein 2, cellular	rbp2, rbpc2, crbp2	biological_process	GO:0048821	erythrocyte development	Developmental related	39.9726	70.8825	38.8083	35.2847	210.415	0.826418073	-0.042646178	-0.179968671	-1.569736161	-2.438800412	-2.576122906
RNA-Seq	Trinity	XLOC_058663	662	XM_008426224.1	570	455/551	9.00E-150	retinol binding protein 2, cellular	rbp2, rbpc2, crbp2	biological_process	GO:0048821	erythrocyte development	Developmental related	58.1483	102.335	54.0046	58.9231	330.671	0.815490732	-0.106654716	0.01909632	-1.692096875	-2.614242323	-2.488491287
RNA-Seq	Trinity	XLOC_022951	480	XM_007575874.1	756	307/411	2.00E-69	sperm acrosome membrane-associated protein 4-like	spaca4, samp14	molecular_function	GO:0005337	spermatid development	Developmental related	5.58328	9.27238	7.43215	7.00364	46.3518	0.731826783	0.412666711	0.32699202	-2.321613768	-2.64077384	-2.726448531
WGS	ABySS	XLOC_057752	795	XM_011480728.1	575	297/392	7.00E-69	sperm acrosome membrane-associated protein 4-like	spaca4, samp14	biological_process	GO:0007286	spermatid development	Developmental related	3.88696	11.6457	6.69312	7.39651	117.366	1.583083193	0.784036619	0.928202443	-3.333145267	-4.132191841	-3.988026017
RNA-Seq	Trinity	XLOC_060521	795	XM_011480728.1	575	297/392	3.00E-69	sperm acrosome membrane-associated protein 4-like	spaca4, samp14	biological_process	GO:0007286	spermatid development	Developmental related	3.94466	11.7826	5.94065	6.92644	109.651	1.578685064	0.590719837	0.812213081	-3.218189138	-4.206154366	-3.984661122
RNA-Seq	Trans-ABySS	XLOC_065495	462	XM_011480728.1	575	279/369	2.00E-63	sperm acrosome membrane-associated protein 4-like	spaca4, samp14	biological_process	GO:0007286	spermatid development	Developmental related	6.01039	19.5287	10.3735	9.92763	169.447	1.700065402	0.787372227	0.723990741	-3.117166275	-4.029859451	-4.093240937
RNA-Seq	Trans-ABySS	XLOC_144435	297	XM_008291544.1	1323	206/286	5.00E-34	sperm acrosome membrane-associated protein 4-like	spaca4, samp14	biological_process	GO:0007286	spermatid development	Developmental related	13.6108	23.1589	16.9839	14.936	114.979	0.766814864	0.319415915	0.134041966	-2.311731753	-2.759130701	-2.944504651
RNA-Seq	Trans-ABySS	XLOC_008985	1541	XM_007542812.1	1721	1044/1310	0	sequestosome 1	sqstm1, p62	biological_process	GO:0007338	regulation of autophagy	Developmental related	20.707	42.0323	128.486	123.729	20.6278	1.02137985	2.633420712	2.578993225	1.02690844	2.638949302	2.584521815
RNA-Seq	Trinity	XLOC_022085	410	XM_007542812.1	1721	190/217	2.00E-68	sequestosome 1	sqstm1, p62	biological_process	GO:0010506	regulation of autophagy	Developmental related	67.6954	146.017	431.352	419.786	37.3317	1.109006635	2.671735936	2.632524344	1.967663232	3.530392533	3.491180941
WGS	ABySS	XLOC_048892	1901	XM_005812279.1	1366	1064/1324	0	sequestosome-1	sqstm1, p62	biological_process	GO:0010506	regulation of autophagy	Developmental related	30.5027	64.3124	213.436	199.269	23.9877	1.076159978	2.806794678	2.707708433	1.422802094	3.153436794	3.054350549
RNA-Seq	Trinity	XLOC_075668	761	XM_008426983.1	2771	490/653	6.00E-122	CD248 molecule, endosialin	tem1, cd248, cd164l1	biological_process	GO:0008284	positive regulation of cell proliferation	Developmental related	14.8264	7.15916	2.47881	3.26632	32.3418	-1.050306111	-2.580448741	-2.182430296	-2.175537747	-3.705680376	-3.307661932
RNA-Seq	Trinity	XLOC_002472	985	XM_005802652.1	630	432/579	3.00E-100	cartilage intermediate layer protein 2-like	cilp2	cellular_component	GO:0070062	extracellular exosome	ECM - extra cellular matrices related	125.826	94.0393	136.53	148.652	18.9324	-0.420094359	0.117787928	0.24050881	2.312406492	2.850288779	2.97300966
RNA-Seq	Trinity	XLOC_002473	956	XM_005802652.1	630	413/565	1.00E-88	cartilage intermediate layer protein 2-like	cilp2	cellular_component	GO:0070062	extracellular exosome	ECM - extra cellular matrices related	7.46135	15.473	7.08189	7.35886	193.975	1.052244353	-0.075302249	-0.019954396	-3.648045879	-4.775592481	-4.720244628
RNA-Seq	Trans-ABySS	XLOC_007440	214	XM_005802652.1	630	125/173	4.00E-17	cartilage intermediate layer protein 2-like	cilp2	cellular_component	GO:0070062	extracellular exosome	ECM - extra cellular matrices related	63.1119	159.361	59.2058	60.2776	1170.74	1.336314644	-0.092173543	-0.066290081	-2.877050204	-4.305538391	-4.279654929
RNA-Seq	Trans-ABySS	XLOC_009185	972	XM_005802652.1	630	432/579	3.00E-100	cartilage intermediate layer protein 2-like	cilp2	cellular_component	GO:0070062	extracellular exosome	ECM - extra cellular matrices related	67.0487	46.6242	68.4797	70.5389	8.42914	-0.524130389	0.030467021	0.073209718	2.467621621	3.022219032	3.064961728
WGS	ABySS	XLOC_015705	1002	XM_005802652.1	630	406/569	1.00E-73	cartilage intermediate layer protein 2-like	cilp2	cellular_component	GO:0070062	extracellular exosome	ECM - extra cellular matrices related	59.6553	44.7914	40.3762	43.0597	436.198	-0.413428558	-0.56314518	-0.47031205	-3.28368949	-3.433406112	-3.340572982
WGS	ABySS	XLOC_052116	1549	XM_005802652.1	630	275/378	2.00E-51	cartilage intermediate layer protein 2-like	cilp2	cellular_component	GO:0070062	extracellular exosome	ECM - extra cellular matrices related	1.98636	8.80506	13.1571	13.4077	101.891	2.14820572	2.727642515	2.754862754	-3.532549979	-2.953113184	-2.925892945
RNA-Seq	Trinity	XLOC_061590	416	XM_010773817.1	742	59/79	0.002	cartilage intermediate layer protein 2-like	cilp2	cellular_component	GO:0070062	extracellular exosome	ECM - extra cellular matrices related	2.98174	10.5264	16.0005	16.8037	127.631	1.819785754	2.42389062	2.494552565	-3.599894756	-2.995789891	-2.925127945
RNA-Seq	Trans-ABySS	XLOC_194255	282	XM_010773817.1	742	108/151	4.00E-09	cartilage intermediate layer protein 2-like	cilp2	cellular_component	GO:0070062	extracellular exosome	ECM - extra cellular matrices related	8.1293	29.1369	39.7534	32.9721	278.71	1.841644356	2.289875221	2.020042741	-3.257845471	-2.809614606	-3.079447086
WGS	ABySS	XLOC_051397	2610	XM_008298221.1	2648	2148/2590	0	collagen, type X, alpha 1	col10a1	cellular_component	GO:0005578	proteinaceous extracellular matrix	ECM - extra cellular matrices related	14.4364	6.07333	2.69089	2.14073	12.3414	-1.249151356	-2.423555701	-2.753536271	-1.022946396	-2.19735074	-2.52733131
RNA-Seq	Trinity	XLOC_022246	1239	XM_011481317.1	8605	439/566	4.00E-112	collagen alpha-1(XI) chain-like	col11a1	molecular_function	GO:0005201	ECM structural constituent	ECM - extra cellular matrices related	23.7847	9.28708	7.31806	6.4454	57.8767	-1.356736861	-1.700500679	-1.883692029	-2.639685697	-2.983449516	-3.166640865
RNA-Seq	Trinity	XLOC_027303	302	XM_008277160.1	7289	188/272	6.00E-15	collagen alpha-1(XI) chain-like	col11a1	molecular_function	GO:0005201	ECM structural constituent	ECM - extra cellular matrices related	23.7127	8.49019	10.8667	14.9156	159.974	-1.481791197	-1.125746053	-0.668837929	-4.235896798	-3.879851654	-3.42294353
RNA-Seq	Trans-ABySS	XLOC_099528	161	XM_008277160.1	7289	74/97	7.00E-08	collagen alpha-1(XI) chain-like	col11a1	molecular_function	GO:0005201	ECM structural constituent	ECM - extra cellular matrices related	147.077	60.7639	80.6935	94.0131	1244.98	-1.27528528	-0.866047283	-0.64563795	-4.356764287	-3.947526289	-3.727116957
RNA-Seq	Trans-ABySS	XLOC_206598	570	XM_011481317.1	8605	295/374	1.00E-80	collagen alpha-1(XI) chain-like	col11a1	molecular_function	GO:0005201	ECM structural constituent	ECM - extra cellular matrices related	29.7829	12.1144	8.04385	8.6312	66.9927	-1.297761285	-1.888526154	-1.786851181	-2.467280945	-3.058045814	-2.956370841
RNA-Seq	Trans-ABySS	XLOC_205428	1138	XM_008422826.1	4174	700/865	0	collagen alpha-2(XI) chain-like	col11a1	molecular_function	GO:0005201	ECM structural constituent	ECM - extra cellular matrices related	29.9249	13.7905	5.42247	5.99003	61.9178	-1.117671661	-2.464324355	-2.320711292	-2.166679447	-3.513332141	-3.369719078
RNA-Seq	Trans-ABySS	XLOC_067114	637	XM_008408500.1	5410	461/612	9.00E-107	collagen alpha-1(II) chain	col2a1	molecular_function	GO:0005201	ECM structural constituent	ECM - extra cellular matrices related	52.0344	25.2119	18.2343	15.9717	152.473	-1.04536086	-1.512810889	-1.703947827	-2.596377041	-3.06382707	-3.254964008
RNA-Seq	Trinity	XLOC_055254	643	XM_008408500.1	5410	461/612	2.00E-106	collagen alpha-1(II) chain	col2a1	molecular_function	GO:0005201	ECM structural constituent	ECM - extra cellular matrices related	44.5536	22.0985	15.9051	13.9869	135.179	-1.011593563	-1.486052566	-1.671465764	-2.612850696	-3.087309699	-3.272722897
RNA-Seq	Trinity	XLOC_041860	3713	XM_008414192.1	6119	2752/3286	0	collagen alpha-1(II) chain-like	col2a1	molecular_function	GO:0005201	ECM structural constituent	ECM - extra cellular matrices related	19.9654	7.56488	4.19338	3.79145	26.7029	-1.400112874	-2.251316499	-2.396680374	-1.819607328	-2.670810954	-2.816174828
WGS	ABySS	XLOC_012829	3191	XM_008303482.1	4376	1347/1537	0	collagen type IV alpha-3-binding protein-like	col4a3bp, gpbp	molecular_function	GO:0005201	ECM structural constituent	ECM - extra cellular matrices related	7.73843	9.60001	33.0256	32.3343	6.97602	0.310995012	2.09347197	2.062952576	0.460631732	2.243108691	2.212589297
RNA-Seq	Trinity	XLOC_007658	1618	XM_005450741.1	3504	1324/1617	0	collagen alpha-1(IX) chain-like	col9a1	cellular_component	GO:0005578	proteinaceous extracellular matrix	ECM - extra cellular matrices related	16.8787	4.98789	2.95729	3.24351	14.5441	-1.758702237	-2.512856162	-2.379576	-1.54393247	-2.298086394	-2.164806232
WGS	ABySS	XLOC_026964	1558	XM_008284241.1	2645	883/1086	0	collagen alpha-1(IX) chain-like	col9a1	cellular_component	GO:0005578	proteinaceous extracellular matrix	ECM - extra cellular matrices related	8.61675	2.66671	2.03189	2.00676	15.8762	-1.692082884	-2.084321526	-2.10227574	-2.573732794	-2.965971435	-2.98392565
RNA-Seq	Trinity	XLOC_040030	2163	XM_008284241.1	2654	1764/2147	0	collagen alpha-1(IX) chain-like	col9a1	cellular_component	GO:0005578	proteinaceous extracellular matrix	ECM - extra cellular matrices related	12.0015	3.53949	2.27553	2.24442	19.7345	-1.761601327	-2.39894022	-2.418800153	-2.479106563	-3.116445455	-3.136305388
RNA-Seq	Trans-ABySS	XLOC_178249	1361	XM_005450741.1	3504	1104/1362	0	collagen alpha-1(IX) chain-like	col9a1	cellular_component	GO:0005578	proteinaceous extracellular matrix	ECM - extra cellular matrices related	21.3661	6.24199	3.54704	3.87424	17.9735	-1.775245643	-2.590636089	-2.46333836	-1.525793423	-2.341183869	-2.21388614
RNA-Seq	Trans-ABySS	XLOC_195749	1161	XM_008284241.1	2654	992/1162	0	collagen alpha-1(IX) chain-like	col9a1	cellular_component	GO:0005578	proteinaceous extracellular matrix	ECM - extra cellular matrices related	18.2058	4.87888	3.30946	3.47947	35.3977	-1.899776233	-2.459730398	-2.387458663	-2.859033718	-3.418987884	-3.346716149
WGS	ABySS	XLOC_060086	2426	XM_005932963.1	2898	1500/1831	0	cartilage matrix protein-like	comp, thbs5	cellular_component	GO:0070062	extracellular exosome	ECM - extra cellular matrices related	6.41046	3.70109	5.63468	5.09234	52.6995	-0.792477667	-0.186094205	-0.332099138	-3.831767151	-3.225383688	-3.371388622
WGS	ABySS	XLOC_034675	1849	XM_008399968.1	1277	684/811	0	cathepsin L1-like	ctsl, mep, catl	biological_process	GO:0030198	ECM organization	ECM - extra cellular matrices related	22.5418	109.617	138.918	147.474	153.376	2.28179693	2.623558919	2.709786	-0.484601194	-0.142839205	-0.056612124
RNA-Seq	Trans-ABySS	XLOC_012812	1157	XM_003447865.2	1861	746/1030	2.00E-155	dentin sialophosphoprotein-like	dspp	cellular_component	GO:0005578	proteinaceous extracellular matrix	ECM - extra cellular matrices related	74.9476	27.6338	10.8126	10.975	70.1154	-1.439448316	-2.793168805	-2.771661341	-1.343297385	-2.697017874	-2.67551041
RNA-Seq	Trinity	XLOC_042279	709	XM_003447865.2	1861	448/621	1.00E-82	dentin sialophosphoprotein-like	dspp	cellular_component	GO:0005578	proteinaceous extracellular matrix	ECM - extra cellular matrices related	77.6965	29.4639	12.5766	11.4084	78.9992	-1.398901205	-2.627107659	-2.76775314	-1.422889638	-2.651096092	-2.791741572
WGS	ABySS	XLOC_046240	1252	XM_003447865.2	1861	886/1261	7.00E-164	dentin sialophosphoprotein-like	dspp	cellular_component	GO:0005578	proteinaceous extracellular matrix	ECM - extra cellular matrices related	68.3814	24.5231	9.71677	9.96742	67.2436	-1.479462596	-2.815055235	-2.778311934	-1.4552556	-2.790848239	-2.754104938
RNA-Seq	Trinity	XLOC_068520	458	XM_003447865.2	1861	306/417	6.00E-65	dentin sialophosphoprotein-like	dspp	cellular_component	GO:0005578	proteinaceous extracellular matrix	ECM - extra cellular matrices related	78.4024	27.6489	9.93951	11.6392	72.3329	-1.503675733	-2.979651181	-2.751905917	-1.387429909	-2.863405358	-2.635660093
RNA-Seq	Trinity	XLOC_003211	635	XM_007541517.1	1941	185/257	3.00E-29	mucin, integumentary mucin C.1-like	muc1, pum, pem, ema	molecular_function	GO:0005201	ECM structural constituent	ECM - extra cellular matrices related	3.48376	24.58	13.0884	10.7288	98.7579	2.818767772	1.909571601	1.622771578	-2.006411243	-2.915607414	-3.202407437
RNA-Seq	Trinity	XLOC_003213	636	XM_007541517.1	1941	183/257	4.00E-26	mucin, integumentary mucin C.1-like	muc1, pum, pem, ema	molecular_function	GO:0005201	ECM structural constituent	ECM - extra cellular matrices related	3.01836	11.0216	6.93997	7.28223	78.7865	1.868496883	1.20116454	1.270615421	-2.837614772	-3.504947115	-3.435496234
WGS	ABySS	XLOC_045735	597	XM_007541516.1	1152	260/368	6.00E-38	mucin, integumentary mucin C.1-like	muc1, pum, pem, ema	molecular_function	GO:0005201	ECM structural constituent	ECM - extra cellular matrices related	2.94045	14.0216	5.91731	8.23063	98.2263	2.25354212	1.008904519	1.484965905	-2.808458372	-4.053095973	-3.577034588
WGS	ABySS	XLOC_019137	1720	XM_007544312.1	2768	1154/1397	0	mucin, intestinal mucin-like protein	muc2	molecular_function	GO:0005201	ECM structural constituent	ECM - extra cellular matrices related	1.75894	6.44014	5.89185	6.11093	32.4941	1.87238578	1.74401443	1.796685684	-2.335013834	-2.463385183	-2.410713929
RNA-Seq	Trans-ABySS	XLOC_005954	89	XM_007578609.1	1729	65/81	1.00E-09	mucin-2-like	muc2	molecular_function	GO:0030197	ECM constituent, lubricant activity	ECM - extra cellular matrices related	1122.25	4895.47	4348.14	3838.04	36842.1	2.125053279	1.954004296	1.773975651	-2.911836017	-3.082885	-3.262913645
RNA-Seq	Trans-ABySS	XLOC_008841	81	XM_008412471.1	3433	51/64	0.001	mucin-2-like	muc2	molecular_function	GO:0030197	ECM constituent, lubricant activity	ECM - extra cellular matrices related	1813.46	8270.27	5866.59	5875.56	64144.7	2.189189506	1.693777246	1.695981438	-2.955323732	-3.450735992	-3.4485318
WGS	ABySS	XLOC_011116	905	XM_007578609.1	1729	436/622	2.00E-70	mucin-2-like	muc2	molecular_function	GO:0030197	ECM constituent, lubricant activity	ECM - extra cellular matrices related	1.59211	6.66359	5.55992	5.7461	53.0336	2.065359621	1.804124108	1.851643084	-2.992535142	-3.253770654	-3.206251679
RNA-Seq	Trans-ABySS	XLOC_015205	329	XM_008412471.1	3433	257/351	4.00E-48	mucin-2-like	muc2	molecular_function	GO:0030197	ECM constituent, lubricant activity	ECM - extra cellular matrices related	5.04232	15.6499	11.9766	14.8329	92.7861	1.633993855	1.24805882	1.556641105	-2.567755257	-2.953690292	-2.645108007
RNA-Seq	Trinity	XLOC_024162	223	XM_008422657.1	714	181/237	1.00E-40	mucin-2-like	muc2	molecular_function	GO:0030197	ECM constituent, lubricant activity	ECM - extra cellular matrices related	28.1694	75.3503	44.6092	49.3838	247.877	1.419484421	0.663212441	0.809909019	-1.717939252	-2.474211232	-2.327514654
RNA-Seq	Trinity	XLOC_035227	425	XM_005809921.1	2527	351/427	6.00E-115	mucin-2-like	muc2	molecular_function	GO:0030197	ECM constituent, lubricant activity	ECM - extra cellular matrices related	2.68172	10.637	8.74592	7.71728	58.0651	1.987860801	1.70545154	1.524933838	-2.448579974	-2.730989236	-2.911506937
RNA-Seq	Trinity	XLOC_093833	345	XM_005465643.1	3152	156/211	1.00E-21	mucin-2-like	muc2	molecular_function	GO:0030197	ECM constituent, lubricant activity	ECM - extra cellular matrices related	10.0207	14.6744	10.2821	12.0153	74.6814	0.550318224	0.037151656	0.261889378	-2.347447457	-2.860614025	-2.635876303
RNA-Seq	Trans-ABySS	XLOC_128190	341	XM_005465643.1	3152	147/201	3.00E-17	mucin-2-like	muc2	molecular_function	GO:0030197	ECM constituent, lubricant activity	ECM - extra cellular matrices related	12.5502	17.5129	12.5975	14.2199	89.2581	0.480707647	0.005427101	0.180200964	-2.349565094	-2.824845641	-2.650071777
RNA-Seq	Trans-ABySS	XLOC_197203	365	XM_005809921.1	2527	298/368	5.00E-91	mucin-2-like	muc2	molecular_function	GO:0030197	ECM constituent, lubricant activity	ECM - extra cellular matrices related	3.95503	14.3559	11.3281	10.5768	82.6652	1.859883236	1.518145363	1.419142664	-2.52563634	-2.867374212	-2.966376911
RNA-Seq	Trans-ABySS	XLOC_205146	733	XM_005809921.1	2527	594/731	0	mucin-2-like	muc2	molecular_function	GO:0030197	ECM constituent, lubricant activity	ECM - extra cellular matrices related	3.19007	9.39763	8.60102	9.11901	50.5417	1.558708886	1.430919678	1.515289126	-2.427105318	-2.554894526	-2.470525078
RNA-Seq	Trans-ABySS	XLOC_010889	114	XM_007578609.1	1729	81/104	7.00E-13	mucin-2-like	muc2	molecular_function	GO:0030197	ECM constituent, lubricant activity	ECM - extra cellular matrices related	127.35	594.002	429.011	503.28	4690.76	2.221668829	1.75221568	1.982562308	-2.981281994	-3.450735144	-3.220388516
WGS	ABySS	XLOC_026525	1111	XM_008422657.1	714	132/176	1.00E-19	mucin-2-like	muc2	molecular_function	GO:0030197	ECM constituent, lubricant activity	ECM - extra cellular matrices related	1.10322	4.59171	4.38225	4.767	26.3049	2.057311012	1.989951274	2.111361109	-2.518228132	-2.58558787	-2.464178035
RNA-Seq	Trinity	XLOC_095094	222	XM_008422657.1	714	145/209	7.00E-13	mucin-2-like	muc2	molecular_function	GO:0030197	ECM constituent, lubricant activity	ECM - extra cellular matrices related	118.323	163.373	122.067	126.743	795.638	0.465439038	0.044942693	0.099175533	-2.283942607	-2.704438951	-2.650206112
WGS	ABySS	XLOC_065291	363	XM_008426306.1	2922	181/235	1.00E-41	periostin-like	postn, pn, osf2	biological_process	GO:0009648	extracellular matrix	ECM - extra cellular matrices related	323.462	194.722	162.881	158.66	1199.23	-0.732180345	-0.989777912	-1.027657782	-2.622620583	-2.88021815	-2.91809802
RNA-Seq	Trans-ABySS	XLOC_002689	289	XM_005459145.1	2633	208/295	5.00E-29	alpha-tectorin-like	tecta	biological_process	GO:0007160	cell-matrix adhesion	ECM - extra cellular matrices related	16.6942	31.296	13.3588	14.2051	382.126	0.906631315	-0.321556541	-0.232937972	-3.609998242	-4.838186099	-4.74956753
RNA-Seq	Trinity	XLOC_005473	1991	XM_005459133.1	3736	781/1116	1.00E-124	alpha-tectorin-like	tecta	biological_process	GO:0007160	cell-matrix adhesion	ECM - extra cellular matrices related	35.2464	58.3024	13.8193	13.7658	330.887	0.726079362	-1.350791371	-1.356387456	-2.504711434	-4.581582167	-4.587178252
RNA-Seq	Trinity	XLOC_005475	2532	XM_005809408.1	1935	1227/1772	0	alpha-tectorin-like	tecta	biological_process	GO:0007160	cell-matrix adhesion	ECM - extra cellular matrices related	13.2902	22.2426	6.15123	5.87767	150.468	0.742962623	-1.11141599	-1.177046549	-2.758059358	-4.612437971	-4.67806853
RNA-Seq	Trinity	XLOC_005501	1944	XM_005466079.1	2027	938/1314	0	alpha-tectorin-like	tecta	biological_process	GO:0007160	cell-matrix adhesion	ECM - extra cellular matrices related	3.47451	8.12388	3.37812	2.94079	68.6476	1.225359396	-0.040588956	-0.240605767	-3.078968448	-4.3449168	-4.544933612
RNA-Seq	Trans-ABySS	XLOC_008454	109	XM_004575237.1	1295	100/115	4.00E-30	alpha-tectorin-like	tecta	biological_process	GO:0007160	cell-matrix adhesion	ECM - extra cellular matrices related	667.983	618.095	301.526	432.848	6560.41	-0.111982792	-1.147528979	-0.625950893	-3.407885481	-4.443431667	-3.921853582
RNA-Seq	Trinity	XLOC_008774	345	XM_005459145.1	2633	252/345	4.00E-42	alpha-tectorin-like	tecta	biological_process	GO:0007160	cell-matrix adhesion	ECM - extra cellular matrices related	10.8557	39.4112	11.3937	7.20919	957.662	1.86015292	0.069783568	-0.59054368	-4.602838974	-6.393208327	-7.053535574
RNA-Seq	Trans-ABySS	XLOC_008999	836	XM_005466079.1	2027	458/645	2.00E-83	alpha-tectorin-like	tecta	biological_process	GO:0007160	cell-matrix adhesion	ECM - extra cellular matrices related	3.31968	9.99206	3.31733	3.64757	116.04	1.58973796	-0.001021645	0.135891485	-3.53769625	-5.128455855	-4.991542725
WGS	ABySS	XLOC_009138	1209	XM_005459144.1	3287	102/122	9.00E-25	alpha-tectorin-like	tecta	biological_process	GO:0007160	cell-matrix adhesion	ECM - extra cellular matrices related	3.24649	10.7307	3.91584	3.08602	70.976	1.72479152	0.270441053	-0.073133354	-2.72558708	-4.179937547	-4.523511954
RNA-Seq	Trans-ABySS	XLOC_009766	3122	XM_005466079.1	2027	1230/1731	0	alpha-tectorin-like	tecta	biological_process	GO:0007160	cell-matrix adhesion	ECM - extra cellular matrices related	38.0138	62.3814	15.5624	15.3178	358.672	0.714592681	-1.288458683	-1.311314143	-2.523477289	-4.526528653	-4.549384113
WGS	ABySS	XLOC_010254	1802	XM_005466079.1	2027	918/1297	8.00E-159	alpha-tectorin-like	tecta	biological_process	GO:0007160	cell-matrix adhesion	ECM - extra cellular matrices related	12.8282	26.5844	9.33328	10.7162	549.656	1.051261154	-0.458862669	-0.25952534	-4.369877185	-5.880001009	-5.680663679
RNA-Seq	Trans-ABySS	XLOC_010642	1718	XM_005466079.1	2027	829/1091	0	alpha-tectorin-like	tecta	biological_process	GO:0007160	cell-matrix adhesion	ECM - extra cellular matrices related	8.43516	18.5703	7.39538	7.50046	195.84	1.138509783	-0.189791154	-0.169436356	-3.398606436	-4.726907373	-4.706552575
RNA-Seq	Trans-ABySS	XLOC_010835	1090	XM_005466079.1	2027	798/1117	2.00E-147	alpha-tectorin-like	tecta	biological_process	GO:0007160	cell-matrix adhesion	ECM - extra cellular matrices related	10.0898	24.0745	10.5729	9.82937	195.325	1.254608258	0.067473565	-0.037726721	-3.020298874	-4.207433567	-4.312633852
RNA-Seq	Trans-ABySS	XLOC_011030	220	XM_005459145.1	2633	59/72	5.00E-09	alpha-tectorin-like	tecta	biological_process	GO:0007160	cell-matrix adhesion	ECM - extra cellular matrices related	34.0212	81.2154	29.8316	23.0591	534.884	1.255319287	-0.189592671	-0.561097823	-2.719400828	-4.164312786	-4.535817938
RNA-Seq	Trans-ABySS	XLOC_011362	654	XM_005817073.1	1808	84/111	4.00E-11	alpha-tectorin-like	tecta	biological_process	GO:0007160	cell-matrix adhesion	ECM - extra cellular matrices related	6.48664	18.064	5.5511	3.51004	131.638	1.477574113	-0.224697691	-0.885983902	-2.865386716	-4.56765852	-5.228944731
WGS	ABySS	XLOC_036116	451	XM_005459133.1	3736	290/389	2.00E-57	alpha-tectorin-like	tecta	biological_process	GO:0007160	cell-matrix adhesion	ECM - extra cellular matrices related	10.0387	26.7922	9.00253	6.75451	227.925	1.416240597	-0.157170047	-0.571649435	-3.08867422	-4.662084863	-5.076564251
WGS	ABySS	XLOC_036930	1400	XM_005459144.1	3287	713/1006	2.00E-126	alpha-tectorin-like	tecta	biological_process	GO:0007160	cell-matrix adhesion	ECM - extra cellular matrices related	7.89778	6.05842	0.952582	1.12584	142.299	-0.382505584	-3.051531988	-2.810445369	-4.553840117	-7.22286652	-6.981779901
WGS	ABySS	XLOC_044492	2437	XM_005809408.1	1935	1210/1706	0	alpha-tectorin-like	tecta	biological_process	GO:0007160	cell-matrix adhesion	ECM - extra cellular matrices related	5.47929	15.1416	3.76551	3.92389	338.561	1.466456794	-0.541143686	-0.481704364	-4.482826227	-6.490426707	-6.430987385
WGS	ABySS	XLOC_050608	3533	XM_005466079.1	2027	1230/1731	0	alpha-tectorin-like	tecta	biological_process	GO:0007160	cell-matrix adhesion	ECM - extra cellular matrices related	35.4606	59.3161	15.4405	15.7422	409.949	0.742206797	-1.199497476	-1.171579773	-2.788948792	-4.730653065	-4.702735362
RNA-Seq	Trinity	XLOC_060883	261	XM_008435904.1	3732	193/263	1.00E-31	alpha-tectorin-like	tecta	biological_process	GO:0007160	cell-matrix adhesion	ECM - extra cellular matrices related	12.9569	43.9117	9.40769	15.8184	385.53	1.7608848	-0.461808161	0.287883094	-3.134165835	-5.356858796	-4.607167541
WGS	ABySS	XLOC_061864	1687	XM_005466079.1	2027	830/1190	2.00E-138	alpha-tectorin-like	tecta	biological_process	GO:0007160	cell-matrix adhesion	ECM - extra cellular matrices related	1.11906	5.51927	2.06067	1.86299	49.2933	2.302190073	0.880826097	0.73533254	-3.158842199	-4.580206175	-4.725699732
RNA-Seq	Trinity	XLOC_005474	2259	XM_005809408.1	1935	1187/1699	0	alpha-tectorin-like	tecta	biological_process	GO:0007160	cell-matrix adhesion	ECM - extra cellular matrices related	5.24931	10.8175	3.70496	3.44913	178.739	1.043167417	-0.502669832	-0.605895293	-4.046415432	-5.592252681	-5.695478142
RNA-Seq	Trans-ABySS	XLOC_008935	2132	XM_005809408.1	1935	1173/1697	4.00E-180	alpha-tectorin-like	tecta	biological_process	GO:0007160	cell-matrix adhesion	ECM - extra cellular matrices related	18.0974	37.145	11.2446	11.4477	609.952	1.037385582	-0.686550103	-0.660724674	-4.037455783	-5.761391468	-5.735566039
RNA-Seq	Trans-ABySS	XLOC_009137	236	XM_008435904.1	3732	178/230	3.00E-42	alpha-tectorin-like	tecta	biological_process	GO:0007160	cell-matrix adhesion	ECM - extra cellular matrices related	62.3833	129.603	40.9787	42.7032	2342.57	1.054867337	-0.606285654	-0.546815688	-4.17592114	-5.837074131	-5.777604165
RNA-Seq	Trans-ABySS	XLOC_009244	321	XM_004574879.1	1425	67/86	2.00E-07	alpha-tectorin-like	tecta	biological_process	GO:0007160	cell-matrix adhesion	ECM - extra cellular matrices related	3.06362	15.5119	4.49446	5.73503	403.56	2.340066141	0.552910428	0.904563671	-4.701335872	-6.488491585	-6.136838341
WGS	ABySS	XLOC_047761	2204	XM_005459144.1	3287	381/529	9.00E-68	alpha-tectorin-like	tecta	biological_process	GO:0007160	cell-matrix adhesion	ECM - extra cellular matrices related	10.623	22.7888	10.0277	10.9837	210.518	1.101133708	-0.083200509	0.048172877	-3.207546731	-4.391880948	-4.260507562
RNA-Seq	Trinity	XLOC_085148	215	XM_005809408.1	1935	160/210	2.00E-31	alpha-tectorin-like	tecta	biological_process	GO:0007160	cell-matrix adhesion	ECM - extra cellular matrices related	16.4747	44.6672	14.2547	6.32131	366.683	1.438963627	-0.208814517	-1.381956722	-3.037245656	-4.6850238	-5.858166005
RNA-Seq	Trans-ABySS	XLOC_040483	920	XM_005812637.1	2499	217/272	4.00E-60	tenascin-like	tnc, hxb	molecular_function	GO:0050294	ECM organization	ECM - extra cellular matrices related	7.07077	5.67659	8.03684	7.69139	58.0873	-0.316842787	0.184761029	0.121377016	-3.355126323	-2.853522507	-2.916906519
RNA-Seq	Trinity	XLOC_044397	1143	XM_005812637.1	2499	738/969	0	tenascin-like	tnc, hxb	biological_process	GO:0030198	ECM organization	ECM - extra cellular matrices related	6.18586	4.31012	7.56209	6.5796	35.6532	-0.521246146	0.289810837	0.089025697	-3.048231628	-2.237174645	-2.437959785
RNA-Seq	Trinity	XLOC_061826	1112	XM_005812637.1	2499	777/928	0	tenascin-like	tnc, hxb	biological_process	GO:0030198	ECM organization	ECM - extra cellular matrices related	6.74519	4.35895	8.5154	7.01294	39.4162	-0.629878427	0.336215218	0.056160303	-3.176736137	-2.210642492	-2.490697407
RNA-Seq	Trinity	XLOC_094850	1735	XM_005812637.1	2499	217/272	9.00E-60	tenascin-like	tnc, hxb	biological_process	GO:0030198	ECM organization	ECM - extra cellular matrices related	5.21347	4.06247	6.59632	6.04079	44.5933	-0.359886767	0.339417462	0.212493308	-3.456397902	-2.757093673	-2.884017827
RNA-Seq	Trans-ABySS	XLOC_097674	509	XM_008432454.1	4455	279/382	6.00E-52	tenascin-like	tnc, hxb	biological_process	GO:0030198	ECM organization	ECM - extra cellular matrices related	7.92542	7.04831	13.6013	12.7368	79.3731	-0.169210013	0.779185253	0.684443563	-3.493300869	-2.544905603	-2.639647293
RNA-Seq	Trans-ABySS	XLOC_141001	962	XM_005812637.1	2499	721/862	0	tenascin-like	tnc, hxb	biological_process	GO:0030198	ECM organization	ECM - extra cellular matrices related	8.78499	5.40638	10.8871	9.06745	49.6042	-0.700377725	0.309507165	0.045656241	-3.197727455	-2.187842565	-2.451693489
WGS	ABySS	XLOC_058898	1917	XM_010753555.1	1242	920/1088	0	family with sequence similarity 134, member B	fam134b, jk1	biological_process	GO:0019233	sensory perception of pain	Environmental Response related	3.13859	7.68135	31.7437	32.7145	2.72395	1.291243308	3.338281812	3.381741737	1.495659665	3.542698169	3.586158094
RNA-Seq	Trinity	XLOC_077523	2055	XM_010753555.1	1242	920/1085	0	family with sequence similarity 134, member B	fam134b, jk1	biological_process	GO:0019233	sensory perception of pain	Environmental Response related	4.5043	8.45665	33.6735	32.9685	3.89727	0.908783352	2.902238858	2.871713425	1.117622386	3.111077893	3.08055246
RNA-Seq	Trinity	XLOC_004853	296	HQ213893.1	2010	295/296	2.00E-146	heat shock protein 70	hspa1a, hsp70, hsp72	biological_process	GO:0071470	cellular response to osmotic stress	Environmental Response related	134.184	5.56281	7.78384	8.26612	4.96674	-4.592255015	-4.10758679	-4.020858538	0.163514604	0.648182829	0.734911081
RNA-Seq	Trinity	XLOC_009359	968	HQ213893.1	2010	731/738	0	heat shock protein 70	hspa1a, hsp70, hsp72	biological_process	GO:0071470	cellular response to osmotic stress	Environmental Response related	59.5808	3.15505	6.35104	6.10222	4.74713	-4.239112721	-3.229782733	-3.287441396	-0.58939269	0.419937298	0.362278635
RNA-Seq	Trans-ABySS	XLOC_014287	872	HQ213893.1	2010	733/740	0	heat shock protein 70	hspa1a, hsp70, hsp72	biological_process	GO:0071470	cellular response to osmotic stress	Environmental Response related	77.7685	3.97251	8.14688	7.86061	6.04026	-4.291063156	-3.254866349	-3.306472734	-0.6045598	0.431637007	0.380030622
RNA-Seq	Trans-ABySS	XLOC_037596	1158	HQ213893.1	2010	1150/1158	0	heat shock protein 70	hspa1a, hsp70, hsp72	biological_process	GO:0071470	cellular response to osmotic stress	Environmental Response related	62.6299	3.30976	5.37725	5.05662	2.0915	-4.242053063	-3.541911123	-3.630606305	0.662188609	1.362330549	1.273635367
RNA-Seq	Trinity	XLOC_076142	762	HQ213893.1	2010	754/760	0	heat shock protein 70	hspa1a, hsp70, hsp72	biological_process	GO:0071470	cellular response to osmotic stress	Environmental Response related	59.4825	3.30651	5.6557	5.50204	1.99988	-4.169084114	-3.394687783	-3.434426751	0.725395829	1.49979216	1.460053192
RNA-Seq	Trinity	XLOC_018510	587	XM_008423864.1	1088	513/586	0	heat shock protein 30-like	hspb1, hsp27	biological_process	GO:0034605	cellular response to heat	Environmental Response related	80.7798	2.87482	5.35588	5.04708	2.60146	-4.812451041	-3.914799032	-4.000473714	0.144150103	1.041802112	0.95612743
RNA-Seq	Trans-ABySS	XLOC_076714	598	XM_008423864.1	797	515/588	0	heat shock protein 30-like	hspb1, hsp27	biological_process	GO:0034605	cellular response to heat	Environmental Response related	91.681	3.15438	5.8872	5.63415	2.81338	-4.861194407	-3.960969235	-4.024352901	0.165052039	1.065277211	1.001893545
RNA-Seq	Trinity	XLOC_012471	984	XM_008436414.1	2024	702/846	0	protein MSS51 homolog, mitochondrial	mss51, zmynd17	molecular_function	GO:0003756	social behavior	Environmental Response related	10.1317	27.3933	56.3915	53.6962	9.04404	1.43494681	2.476601454	2.40594373	1.598783796	2.640438441	2.569780716
RNA-Seq	Trinity	XLOC_044838	372	XM_007571459.1	1490	316/371	1.00E-113	protein MSS51 homolog, mitochondrial	mss51, zmynd17	biological_process	GO:0035176	social behavior	Environmental Response related	12.6755	28.8156	97.7225	93.2183	17.5202	1.184807403	2.946648115	2.878570546	0.717830816	2.479671528	2.411593959
RNA-Seq	Trinity	XLOC_005395	942	XM_007544523.1	3606	724/942	0	otoancorin-like	otoa	molecular_function	GO:0004586	sensory perception of sound	Environmental Response related	9.04851	15.3229	9.6751	8.52565	45.6875	0.759937215	0.096596327	-0.085870416	-1.576110135	-2.239451023	-2.421917766
RNA-Seq	Trans-ABySS	XLOC_013678	544	XM_007544523.1	3606	411/546	5.00E-96	otoancorin-like	otoa	biological_process	GO:0007605	sensory perception of sound	Environmental Response related	13.5485	23.7104	14.3004	14.5646	78.8967	0.807386867	0.077922367	0.104332945	-1.734444956	-2.463909457	-2.437498878
RNA-Seq	Trinity	XLOC_078030	1386	XM_007544523.1	3606	688/867	0	otoancorin-like	otoa	biological_process	GO:0007605	sensory perception of sound	Environmental Response related	11.7672	20.4435	12.7492	12.4421	62.8851	0.796871139	0.11563565	0.080458934	-1.621076013	-2.302311503	-2.337488219
RNA-Seq	Trans-ABySS	XLOC_010975	539	KJ577417.1	24550	153/186	5.00E-42	period 2	per2	molecular_function	GO:0030246	photoperiodism	Environmental Response related	3.81207	5.9158	38.3484	34.1514	7.94125	0.633998668	3.330519869	3.163298205	-0.424792835	2.271728367	2.104506702
WGS	ABySS	XLOC_042841	995	XM_010742313.1	739	188/230	1.00E-54	proline-rich transmembrane protein 1-like	prrt1, ng5, dspd1, c6orf31, ifitmd7	molecular_function	GO:0051015	response to biotic stimulus	Environmental Response related	2.20379	5.54254	5.5018	6.6116	52.3593	1.330560521	1.319916941	1.585012689	-3.239826628	-3.250470209	-2.98537446

RNA-Seq	Trans-ABySS	XLOC_206524	684	XM_005799009.1	763	207/264	6.00E-52	proline-rich transmembrane protein 1-like	prrt1, ng5, dspd1, c6orf31, ifitmd7	biological_process	GO:0009607	response to biotic stimulus	Environmental Response related	2.9456	8.04763	6.87694	7.9154	51.2671	1.450002445	1.223205227	1.426100726	-2.67139741	-2.898194628	-2.695299129
RNA-Seq	Trinity	XLOC_026320	1174	XM_007549327.1	3731	970/1178	0	ectonucleoside triphosphate diphosphohydrolase 5	entpd5	molecular_function	GO:0016787	hydrolase activity	Hormone related	6.19185	5.30751	2.39947	3.00882	31.0213	-0.222335338	-1.367654747	-1.041172721	-2.547152057	-3.692471466	-3.36598944
RNA-Seq	Trans-ABySS	XLOC_107555	1083	XM_007549327.1	3731	852/989	0	ectonucleoside triphosphate diphosphohydrolase 5	entpd5	molecular_function	GO:0016787	hydrolase activity	Hormone related	7.21248	6.15253	2.68966	3.49682	34.2006	-0.229315626	-1.4230716	-1.044451878	-2.474769943	-3.668525917	-3.289906195
RNA-Seq	Trans-ABySS	XLOC_016550	608	HQ602761.1	3540	109/143	5.00E-19	follicle stimulating hormone receptor	fshr	biological_process	GO:0042699	follicle-stimulating hormone signaling pathway	Hormone related	2.82234	5.15489	2.76033	4.12102	45.8754	0.869049846	-0.032051043	0.546109669	-3.15370719	-4.054808079	-3.476647367
RNA-Seq	Trinity	XLOC_090521	429	HQ602761.1	3540	74/89	1.00E-15	follicle stimulating hormone receptor	fshr	biological_process	GO:0042699	follicle-stimulating hormone signaling pathway	Hormone related	2.45849	4.89339	2.62977	3.87433	47.4691	0.993061782	0.09716414	0.656174355	-3.278082523	-4.173980165	-3.61496995
RNA-Seq	Trinity	XLOC_055364	640	XM_005743200.1	1332	126/149	9.00E-37	sulfotransferase family cytosolic 2B member 1-like	sult2b1	cellular_component	GO:0005575	steroid sulfotransferase activity	Hormone related	1.26877	4.29995	4.61436	6.17223	28.8854	1.76088932	1.8627	2.282361259	-2.747948684	-2.646138004	-2.226476745
RNA-Seq	Trinity	XLOC_072399	1513	XM_008426380.1	643	452/552	7.00E-148	thyroid hormone responsive	thrsp, s14	molecular_function	GO:0004497	regulation of triglyceride biosynthetic process	Hormone related	15.546	81.8883	89.1747	90.3429	121.758	2.397113917	2.520091036	2.538867804	-0.572287323	-0.449310204	-0.430533436
RNA-Seq	Trans-ABySS	XLOC_010631	596	XM_005816386.1	1668	114/141	2.00E-27	CUB and zona pellucida-like domain-containing protein 1-like	zpld1	biological_process	GO:0009755	hormone-mediated signaling pathway	Hormone related	3.83953	20.9937	4.43793	3.37596	137.88	2.450954829	0.208957192	-0.18563191	-2.715384844	-4.957382482	-5.351971583
WGS	ABySS	XLOC_013393	1485	XM_005468143.1	2448	583/862	3.00E-69	CUB and zona pellucida-like domain-containing protein 1-like	zpld1	biological_process	GO:0009755	hormone-mediated signaling pathway	Hormone related	2.11777	11.3189	2.53326	1.4325	58.7158	2.418115941	0.258449241	-0.564010775	-2.375015014	-4.534681714	-5.35714173
WGS	ABySS	XLOC_028837	1562	XM_005816386.1	1668	631/771	0	CUB and zona pellucida-like domain-containing protein 1-like	zpld1	biological_process	GO:0009755	hormone-mediated signaling pathway	Hormone related	1.97166	7.78911	1.2544	1.35946	56.7629	1.982047703	-0.652413325	-0.536377087	-2.865417901	-5.499878928	-5.38384269
RNA-Seq	Trinity	XLOC_045837	1846	XM_005816386.1	1668	871/1066	0	CUB and zona pellucida-like domain-containing protein 1-like	zpld1	biological_process	GO:0009755	hormone-mediated signaling pathway	Hormone related	3.00807	14.4989	2.78691	2.49305	91.9685	2.269033405	-0.110171727	-0.270926323	-2.66519636	-5.044401492	-5.205156088
RNA-Seq	Trans-ABySS	XLOC_110541	1044	XM_005816386.1	1668	824/1009	0	CUB and zona pellucida-like domain-containing protein 1-like	zpld1	biological_process	GO:0009755	hormone-mediated signaling pathway	Hormone related	4.1266	17.1152	3.02695	3.25259	112.116	2.052252644	-0.447088757	-0.343364624	-2.711642123	-5.210983524	-5.107259391
RNA-Seq	Trans-ABySS	XLOC_162166	608	XM_005816386.1	1668	62/81	8.00E-06	CUB and zona pellucida-like domain-containing protein 1-like	zpld1	biological_process	GO:0009755	hormone-mediated signaling pathway	Hormone related	4.51575	19.7881	4.41653	2.32468	74.7396	2.131595572	-0.032052307	-0.95793348	-1.917239747	-4.080887626	-5.006768799
WGS	ABySS	XLOC_029800	457	XM_008425137.1	5274	122/155	5.00E-24	alpha-2-macroglobulin-like protein 1	a2m	cellular_component	GO:0005615	extracellular space	Immune System related	48.0669	106.549	163.045	157.598	20.454	1.148401386	1.762154532	1.713133559	2.381062143	2.994815289	2.945794316
RNA-Seq	Trinity	XLOC_073711	1428	XM_008296517.1	5520	1063/1433	0	complement C4-like	c4a	cellular_component	GO:0005615	extracellular space	Immune System related	0.7425	8.21775	11.1837	10.0642	2.44212	3.468280511	3.91286273	3.760697661	1.750609349	2.195191569	2.0430265
WGS	ABySS	XLOC_014993	1950	AB162342.1	103035	350/467	3.00E-76	MHC class I a region	hla-a	cellular_component	GO:0042612	MHC class I protein complex	Immune System related	0.529012	1.01536	0.795661	1.07943	12.5751	0.940618978	0.588853437	1.028897335	-3.630506636	-3.982272177	-3.542228279
RNA-Seq	Trinity	XLOC_068610	449	XM_003968367.1	328	153/205	2.00E-27	interferon-induced transmembrane protein 1-like	ifitm1, if17, leu13, cd225	biological_process	GO:0002376	immune system process	Immune System related	0.645098	2.18628	4.83029	3.71283	64.0912	1.760887932	2.904519559	2.524929011	-4.873576196	-3.729944569	-4.109535117
RNA-Seq	Trans-ABySS	XLOC_020213	836	XM_008286999.1	1787	616/748	0	myeloid leukemia factor 1	mlf1	biological_process	GO:0002318	myeloid progenitor cell differentiation	Immune System related	15.9489	6.9094	5.92884	6.55212	35.0787	-1.206824584	-1.427635156	-1.28342324	-2.343962943	-2.564773514	-2.420561598
RNA-Seq	Trinity	XLOC_008895	2145	DQ423779.1	2021	1968/1971	0	Rhesus glycoprotein	rhcg, rhgk	biological_process	GO:0048821	plasma membrane	Immune System related	1.18074	8.07987	18.0682	18.6296	24.9482	2.774640764	3.935689566	3.979833476	-1.626531743	-0.465482941	-0.421339031
RNA-Seq	Trans-ABySS	XLOC_013361	812	DQ423779.1	2021	654/654	0	Rhesus glycoprotein	rhcg, rhgk	cellular_component	GO:0005886	plasma membrane	Immune System related	0.975204	10.9942	26.1188	25.4174	34.4214	3.494894776	4.743240762	4.703968608	-1.646563147	-0.398217161	-0.437489315
RNA-Seq	Trans-ABySS	XLOC_018320	1324	DQ423779.1	2021	1305/1308	0	Rhesus glycoprotein	rhcg, rhgk	cellular_component	GO:0005886	plasma membrane	Immune System related	1.79182	9.50734	20.7506	21.8026	27.554	2.407616038	3.533655431	3.605002568	-1.535148109	-0.409108717	-0.33776158
WGS	ABySS	XLOC_048181	2765	DQ423779.1	2021	1968/1971	0	Rhesus glycoprotein	rhcg, rhgk	cellular_component	GO:0005886	plasma membrane	Immune System related	1.00289	7.02212	15.9987	16.7534	22.4791	2.807743275	3.995719401	4.062218631	-1.67860572	-0.490629594	-0.424130364
WGS	ABySS	XLOC_041292	2449	XM_007552670.1	2057	1413/1739	0	calreticulin	calr, ro, cc1qr	molecular_function	GO:0005509	calcium ion binding	Ion Binding related	20.3181	13.0457	12.9078	13.2268	65.929	-0.63919114	-0.654522369	-0.61930143	-2.337338841	-2.35267007	-2.317449131
RNA-Seq	Trans-ABySS	XLOC_163337	329	XM_007552670.1	2057	98/119	4.00E-22	calreticulin	calr, ro, cc1qr	molecular_function	GO:0005509	calcium ion binding	Ion Binding related	80.6772	49.0719	47.2018	45.1729	249.647	-0.717263883	-0.773319138	-0.83670348	-2.346920533	-2.402975788	-2.46636013
WGS	ABySS	XLOC_034062	1392	XM_008429102.1	1549	967/1184	0	hemopexin-like	hpx	molecular_function	GO:0046872	metal ion binding	Ion Binding related	8.65433	13.5274	13.1781	13.2348	70.9792	0.644390538	0.606648341	0.612842355	-2.391511738	-2.429253934	-2.423059921
RNA-Seq	Trinity	XLOC_070223	1423	XM_008429102.1	1549	967/1184	0	hemopexin-like	hpx	molecular_function	GO:0046872	metal ion binding	Ion Binding related	9.04379	14.7696	13.9479	13.5258	75.2314	0.707631357	0.625048528	0.580714529	-2.348704183	-2.431287012	-2.475621012
RNA-Seq	Trans-ABySS	XLOC_007479	312	XM_004574851.1	924	236/317	6.00E-46	uromodulin-like	umod, thp, thgp, umodl1	molecular_function	GO:0005509	calcium ion binding	Ion Binding related	45.302	78.5566	27.7924	28.9106	1887.73	0.794157746	-0.704884321	-0.647976194	-4.586776132	-6.085818199	-6.028910072
RNA-Seq	Trans-ABySS	XLOC_009484	596	XM_007568140.1	2441	429/600	5.00E-73	uromodulin-like	umod, thp, thgp, umodl1	molecular_function	GO:0005509	calcium ion binding	Ion Binding related	7.21367	25.9636	6.6	5.33619	499.152	1.847685104	-0.128267402	-0.43492339	-4.264916867	-6.240869372	-6.547525361
RNA-Seq	Trans-ABySS	XLOC_010924	551	XM_007568140.1	2441	323/445	2.00E-57	uromodulin-like	umod, thp, thgp, umodl1	molecular_function	GO:0005509	calcium ion binding	Ion Binding related	4.206	13.7457	2.82809	3.19863	195.627	1.708459623	-0.572620817	-0.394994731	-3.831053218	-6.112133659	-5.934507572
WGS	ABySS	XLOC_050994	453	XM_007568140.1	2441	204/295	1.00E-21	uromodulin-like	umod, thp, thgp, umodl1	molecular_function	GO:0005509	calcium ion binding	Ion Binding related	19.7597	31.6194	21.7395	17.8466	3281.8	0.678248948	0.137757716	-0.146911794	-6.697533519	-7.238024751	-7.522694261
RNA-Seq	Trans-ABySS	XLOC_113053	222	XM_007568140.1	2441	72/88	8.00E-13	uromodulin-like	umod, thp, thgp, umodl1	molecular_function	GO:0005509	calcium ion binding	Ion Binding related	26.1774	55.3224	14.4709	13.8488	1000.23	1.079541932	-0.855167161	-0.918560841	-4.176324227	-6.11103332	-6.174426999
WGS	ABySS	XLOC_063107	837	XM_003454657.2	2465	233/332	3.00E-29	uromodulin-like	umod, thp, thgp, umodl1	molecular_function	GO:0005509	calcium ion binding	Ion Binding related	9.63683	20.7765	7.90236	7.38108	162.041	1.108322079	-0.286275084	-0.384726728	-2.963334349	-4.357931512	-4.456383156
RNA-Seq	Trans-ABySS	XLOC_001380	431	n/a	n/a	n/a	n/a	n/a	n/a	n/a	n/a	n/a	n/a	3.61645	15.9806	11.397	8.27434	99.4173	2.143675465	1.656008007	1.194070035	-2.637175347	-3.124842805	-3.586780777
RNA-Seq	Trans-ABySS	XLOC_003936	74	n/a	n/a	n/a	n/a	n/a	n/a	n/a	n/a	n/a	n/a	3713.34	12306.5	17137.3	14337	71860.2	1.728631192	2.206350507	1.948953851	-2.545772437	-2.068053121	-2.325449777
WGS	ABySS	XLOC_004111	259	n/a	n/a	n/a	n/a	n/a	n/a	n/a	n/a	n/a	n/a	34.4377	178.262	92.0356	89.6358	763.441	2.3719385	1.418203221	1.380086259	-2.09851747	-3.052252749	-3.090369711
RNA-Seq	Trinity	XLOC_004264	1664	n/a	n/a	n/a	n/a	n/a	n/a	n/a	n/a	n/a	n/a	1.65093	5.25878	4.37723	4.024	45.0966	1.671449192	1.406739241	1.285351354	-3.100218619	-3.36492857	-3.486316457
RNA-Seq	Trinity	XLOC_005196	462	n/a	n/a	n/a	n/a	n/a	n/a	n/a	n/a	n/a	n/a	4.13501	13.0334	7.03327	5.43555	41.0895	1.656250763	0.766304784	0.394535204	-1.656556289	-2.546502268	-2.918271847
RNA-Seq	Trans-ABySS	XLOC_005645	371	n/a	n/a	n/a	n/a	n/a	n/a	n/a	n/a	n/a	n/a	14.7172	30.3087	7.46348	7.90796	623.166	1.042228754	-0.979582841	-0.896125741	-4.361812643	-6.383624238	-6.300167138
RNA-Seq	Trans-ABySS	XLOC_005648	95	n/a	n/a	n/a	n/a	n/a	n/a	n/a	n/a	n/a	n/a	4618.86	4100.65	3836.67	4115.92	25096.1	-0.171684206	-0.267682137	-0.166321877	-2.613538666	-2.709536597	-2.608176337
RNA-Seq	Trans-ABySS	XLOC_006350	146	n/a	n/a	n/a	n/a	n/a	n/a	n/a	n/a	n/a	n/a	192.954	368.708	91.8072	117.148	2666.69	0.934221768	-1.071577745	-0.719924628	-2.854499498	-4.860299011	-4.508645894
RNA-Seq	Trans-ABySS	XLOC_006511	66	n/a	n/a	n/a	n/a	n/a	n/a	n/a	n/a	n/a	n/a	65445.5	35152.6	18528.5	18192.7	211439	-0.896662597	-1.820547906	-1.846934326	-2.588538202	-3.512423511	-3.538809931
RNA-Seq	Trans-ABySS	XLOC_007726	92	n/a	n/a	n/a	n/a	n/a	n/a	n/a	n/a	n/a	n/a	7394.77	7234.33	6800.52	7128.04	43276.1	-0.031645867	-0.12086021	-0.052999843	-2.580639176	-2.66985352	-2.601993153
RNA-Seq	Trans-ABySS	XLOC_008111	82	n/a	n/a	n/a	n/a	n/a	n/a	n/a	n/a	n/a	n/a	2091.41	1877.21	1598.02	917.597	19505.7	-0.155885865	-0.388190451	-1.188853336	-3.377233817	-3.609538404	-4.409891288
RNA-Seq	Trinity	XLOC_008571	435	n/a	n/a	n/a	n/a	n/a	n/a	n/a	n/a	n/a	n/a	69.6164	33.751	4.95224	8.03974	18.4602	-1.044496963	-3.813274073	-3.11420646	0.870512065	-1.898265045	-1.199197432
RNA-Seq	Trans-ABySS	XLOC_008788	413	n/a	n/a	n/a	n/a	n/a	n/a	n/a	n/a	n/a	n/a	243.936	430.17	868.946	847.73	66.6036	0.818404229	1.832763838	1.797102157	2.691234851	3.705594461	3.669932779
RNA-Seq	Trans-ABySS	XLOC_008800	119	n/a	n/a	n/a	n/a	n/a	n/a	n/a	n/a	n/a	n/a	1596.49	1402.22	855.887	608.789	11772.5	-0.187190799	-0.899411276	-1.39088932	-3.0696361	-3.781856578	-4.273334621
RNA-Seq	Trans-ABySS	XLOC_009051	311	n/a	n/a	n/a	n/a	n/a	n/a	n/a	n/a	n/a	n/a	185.216	91.142	51.176	67.6339	611.229	-1.023020798	-1.855669439	-1.45339028	-2.74552506	-3.578173701	-3.175894542
RNA-Seq	Trans-ABySS	XLOC_009055	144	n/a	n/a	n/a	n/a	n/a	n/a	n/a	n/a	n/a	n/a	605.245	855.026	597.328	623.153	3690.81	0.498449035	-0.018995906	0.042067169	-2.109897275	-2.627342215	-2.56627914
RNA-Seq	Trans-ABySS	XLOC_009070	128	n/a	n/a	n/a	n/a	n/a	n/a	n/a	n/a	n/a	n/a	149.205	626.995	669.546	460.038	5426.57	2.071158056	2.165887294	1.624457153	-3.113514751	-3.018785512	-3.560215654
RNA-Seq	Trans-ABySS	XLOC_009200	99	n/a	n/a	n/a	n/a	n/a	n/a	n/a	n/a	n/a	n/a	1178.16	1484.71	1331.52	1200.77	7915.65	0.333645687	0.176538621	0.027424361	-2.414526658	-2.571633725	-2.720747985
RNA-Seq	Trans-ABySS	XLOC_009321	332	n/a	n/a	n/a	n/a	n/a	n/a	n/a	n/a	n/a	n/a	21.8389	14.5273	17.9141	20.441	144.164	-0.588133598	-0.285804626	-0.095434415	-3.310872448	-3.008543476	-2.818173264
RNA-Seq	Trans-ABySS	XLOC_009501	67	n/a	n/a	n/a	n/a	n/a	n/a	n/a	n/a	n/a	n/a	9716.81	8782.59	7775.44	5374.22	70631.9	-0.145836303	-0.321558442	-0.854427378	-3.007601545	-3.183323683	-3.716192619
RNA-Seq	Trans-ABySS	XLOC_009509	63	n/a	n/a	n/a	n/a	n/a	n/a	n/a	n/a	n/a	n/a	3520.49	11408.1	7919.23	5601.75	90695.1	1.696210383	1.169583917	0.670101354	-2.990966075	-3.517592542	-4.017075104
RNA-Seq	Trans-ABySS	XLOC_009542	142	n/a	n/a	n/a	n/a	n/a	n/a	n/a	n/a	n/a	n/a	92.9748	299.572	312.579	533.019	2101.47	1.687991142	1.749309212	2.519275316	-2.81042417	-2.749106099	-1.979139996
RNA-Seq	Trans-ABySS	XLOC_009546	119	n/a	n/a	n/a	n/a	n/a	n/a	n/a	n/a	n/a	n/a	591.293	705.466	358.547	442.756	3621.41	0.254703356	-0.721710951	-0.41736134	-2.359903063	-3.33631737	-3.031967759
RNA-Seq	Trinity	XLOC_009726	585	n/a	n/a	n/a	n/a	n/a	n/a	n/a	n/a	n/a	n/a	1.97046	2.57749	30.8544	25.6663	4.18419	0.387434367	3.968871872	3.703270967	-0.698981534	2.88245597	2.616855065
RNA-Seq	Trans-ABySS	XLOC_009750	1008	n/a	n/a	n/a	n/a	n/a	n/a	n/a	n/a	n/a	n/a	24.7904	11.6982	3.107	4.14159	251.258	-1.08349499	-2.996187403	-2.581524905	-4.424811067	-6.33750348	-5.922840982
RNA-Seq	Trinity	XLOC_010144	301	n/a	n/a	n/a	n/a	n/a	n/a	n/a	n/a	n/a	n/a	17.649	11.2243	16.4453	14.6724	122.632	-0.652960968	-0.101911116	-0.266481567	-3.44963811	-2.898588258	-3.063158709
RNA-Seq	Trans-ABySS	XLOC_011060	425	n/a	n/a	n/a	n/a	n/a	n/a	n/a	n/a	n/a	n/a	11.7627	23.2532	146.326	134.628	13.2407	0.983210014	3.63689498	3.516687335	0.812449871	3.466134838	3.345927193
RNA-Seq	Trans-ABySS	XLOC_011063	103	n/a	n/a	n/a	n/a	n/a	n/a	n/a	n/a	n/a	n/a	1995.34	920.47	557.572	513.083	6288.29	-1.116191991	-1.839404579	-1.959370468	-2.772225145	-3.495437733	-3.615403622
RNA-Seq	Trans-ABySS	XLOC_011183	360	n/a	n/a	n/a	n/a	n/a	n/a	n/a	n/a	n/a	n/a	36.4183	22.1007	29.3494	29.4513	152.946	-0.720571514	-0.311332569	-0.306332262	-2.790858406	-2.381619461	-2.376619154
RNA-Seq	Trans-ABySS	XLOC_011827	408	n/a	n/a	n/a	n/a	n/a	n/a	n/a	n/a	n/a	n/a	26.3754	70.2916	132.477	128.038	21.9469	1.414159321	2.328477029	2.279307168	1.679335121	2.593652829	2.544482968
RNA-Seq	Trans-ABySS	XLOC_012461	314	n/a	n/a	n/a	n/a	n/a	n/a	n/a	n/a	n/a	n/a	21.875	18.1988	17.8286	20.0956	122.475	-0.265439692	-0.295089598	-0.122403364	-2.750572062	-2.780221967	-2.607535734
RNA-Seq	Trinity	XLOC_012791	357	n/a	n/a	n/a	n/a	n/a	n/a	n/a	n/a	n/a	n/a	29.3236	22.8593	25.937	38.4916	149.442	-0.359281005	-0.177050611	0.39248141	-2.708732537	-2.526502143	-1.956970121
RNA-Seq	Trinity	XLOC_013781	824	n/a	n/a	n/a	n/a	n/a	n/a	n/a	n/a	n/a	n/a	0.382389	2.92787	15.2068	16.0782	1.86418	2.93673857	5.313531763	5.393921069	0.651310329	3.028103521	3.108492827
RNA-Seq	Trans-ABySS	XLOC_015198	259	n/a	n/a	n/a	n/a	n/a	n/a	n/a	n/a	n/a	n/a	180.912	88.1415	25.86	26.7023	22.9989	-1.037394752	-2.806493926	-2.760252187	1.938256588	0.169157414	0.215399153
RNA-Seq	Trans-ABySS	XLOC_015217	62	n/a	n/a	n/a	n/a	n/a	n/a	n/a	n/a	n/a	n/a	11261.2	10959.4	8260.32	4517.3	109476	-0.039191754	-0.447090993	-1.317827937	-3.320373906	-3.728273146	-4.59901009
RNA-Seq	Trinity	XLOC_016567	470	n/a	n/a	n/a	n/a	n/a	n/a	n/a	n/a	n/a	n/a	11.4378	10.4041	10.5292	10.6867	87.9481	-0.136657413	-0.119413758	-0.097993159	-3.079500239	-3.062256585	-3.040835986
RNA-Seq	Trinity	XLOC_018656	700	n/a	n/a	n/a	n/a	n/a	n/a	n/a	n/a	n/a	n/a	2.31025	4.07979	19.8037	21.0911	5.62466	0.820445915	3.099649116	3.190513456	-0.463270997	1.815932204	1.906796545
WGS	ABySS	XLOC_021138	570	n/a	n/a	n/a	n/a	n/a	n/a	n/a	n/a	n/a	n/a	19.3474	7.76518	5.37709	6.05797	63.4185	-1.31704843	-1.847242179	-1.675233364	-3.029812482	-3.56000623	-3.387997416
RNA-Seq	Trinity	XLOC_022799	364	n/a	n/a	n/a	n/a	n/a	n/a	n/a	n/a	n/a	n/a	33.9812	21.3268	29.0087	31.179	154.485	-0.672069289	-0.228251156	-0.124162143	-2.856727347	-2.412909215	-2.308820201
WGS	ABySS	XLOC_025317	2351	n/a	n/a	n/a	n/a	n/a	n/a	n/a	n/a	n/a	n/a	0.806639	0.865039	1.0245	0.929462	10.8999	0.100842018	0.344924919	0.204472723	-3.655405911	-3.41132301	-3.551775206
RNA-Seq	Trinity	XLOC_028773	756	n/a	n/a	n/a	n/a	n/a	n/a	n/a	n/a	n/a	n/a	2.4338	6.63099	20.0816	16.8915	16.8228	1.446013661	3.044591697	2.795014925	-1.343121664	0.255456372	0.005879599
RNA-Seq	Trinity	XLOC_029041	700	n/a	n/a	n/a	n/a	n/a	n/a	n/a	n/a	n/a	n/a	10.9936	7.19964	9.3849	8.71154	44.9169	-0.610667218	-0.228250616	-0.335664211	-2.641261687	-2.258845084	-2.36625868
RNA-Seq	Trinity	XLOC_030100	472	n/a	n/a	n/a	n/a	n/a	n/a	n/a	n/a	n/a	n/a	17.3973	47.6347	89.4929	86.5523	15.1682	1.453149481	2.362909807	2.314708735	1.650963011	2.560723337	2.512522265
RNA-Seq	Trinity	XLOC_031531	934	n/a	n/a	n/a	n/a	n/a	n/a	n/a	n/a	n/a	n/a	41.9494	21.9726	3.62064	3.27109	9.66348	-0.932944583	-3.534333534	-3.680806817	1.185090864	-1.416298087	-1.56277137
RNA-Seq	Trinity	XLOC_035568	265	n/a	n/a	n/a	n/a	n/a	n/a	n/a	n/a	n/a	n/a	6.18949	12.2893	8.98811	18.3514	136.21	0.989510297	0.538197241	1.567997684	-3.470357977	-3.921671033	-2.891870591
RNA-Seq	Trinity	XLOC_037379	524	n/a	n/a	n/a	n/a	n/a	n/a	n/a	n/a	n/a	n/a	6.5443	4.46371	4.68443	5.09312	35.6196	-0.551995584	-0.482365373	-0.361689176	-2.99635611	-2.926725898	-2.806049701
RNA-Seq	Trinity	XLOC_038496	357	n/a	n/a	n/a	n/a	n/a	n/a	n/a	n/a	n/a	n/a	13.3757	27.8961	169.233	156.927	16.6046	1.060449046	3.661324618	3.552407296	0.748480472	3.349356043	3.240438722
RNA-Seq	Trinity	XLOC_040887	655	n/a	n/a	n/a	n/a	n/a	n/a	n/a	n/a	n/a	n/a	12.2657	14.9782	7.7847	8.4882	68.4863	0.288234688	-0.655916222	-0.531099016	-2.192951161	-3.137102072	-3.012284865
WGS	ABySS	XLOC_041178	4826	n/a	n/a	n/a	n/a	n/a	n/a	n/a	n/a	n/a	n/a	1.99574	4.69083	10.6934	11.3065	1.1387	1.232919435	2.421724948	2.502156716	2.04245551	3.231261023	3.311692791
WGS	ABySS	XLOC_041182	4378	n/a	n/a	n/a	n/a	n/a	n/a	n/a	n/a	n/a	n/a	0.700747	3.20233	12.8509	13.0413	2.00563	2.192156417	4.196831928	4.218050216	0.675066504	2.679742014	2.700960303
RNA-Seq	Trinity	XLOC_042215	330	n/a	n/a	n/a	n/a	n/a	n/a	n/a	n/a	n/a	n/a	15.7951	7.23072	9.89437	8.91255	103.711	-1.127265855	-0.674797316	-0.825566901	-3.842285799	-3.38981726	-3.540586845
RNA-Seq	Trans-ABySS	XLOC_042646	528	n/a	n/a	n/a	n/a	n/a	n/a	n/a	n/a	n/a	n/a	9.71296	6.22031	7.43443	8.30069	51.0441	-0.642924539	-0.385688886	-0.226679754	-3.036685828	-2.779450175	-2.620441043
RNA-Seq	Trinity	XLOC_044040	703	n/a	n/a	n/a	n/a	n/a	n/a	n/a	n/a	n/a	n/a	1.82125	7.15635	20.2381	22.1037	3.3545	1.974294976	3.474072975	3.601287009	1.093126204	2.592904204	2.720118238
RNA-Seq	Trans-ABySS	XLOC_044707	675	n/a	n/a	n/a	n/a	n/a	n/a	n/a	n/a	n/a	n/a	26.4359	18.0431	17.7103	19.7587	130.099	-0.551051213	-0.577909793	-0.420010414	-2.850090737	-2.876949317	-2.719049938
RNA-Seq	Trans-ABySS	XLOC_053034	335	n/a	n/a	n/a	n/a	n/a	n/a	n/a	n/a	n/a	n/a	5.51404	17.0052	12.471	9.35446	185.607	1.624794336	1.177395516	0.762544641	-3.448203242	-3.895602063	-4.310452937
RNA-Seq	Trinity	XLOC_054356	265	n/a	n/a	n/a	n/a	n/a	n/a	n/a	n/a	n/a	n/a	37.1369	17.3746	19.6615	22.1296	292.284	-1.095873627	-0.917480001	-0.746876017	-4.072319187	-3.89392556	-3.723321577
RNA-Seq	Trinity	XLOC_055916	642	n/a	n/a	n/a	n/a	n/a	n/a	n/a	n/a	n/a	n/a	6.31489	19.4313	44.4018	34.6144	3.36671	1.621552915	2.813788654	2.454542832	2.528971062	3.721206802	3.361960979
WGS	ABySS	XLOC_057659	599	n/a	n/a	n/a	n/a	n/a	n/a	n/a	n/a	n/a	n/a	4.12707	4.91915	4.43002	4.14282	81.2863	0.25329114	0.102195304	0.00549523	-4.046531268	-4.197627104	-4.294327177
WGS	ABySS	XLOC_058902	2882	n/a	n/a	n/a	n/a	n/a	n/a	n/a	n/a	n/a	n/a	1.85387	3.6147	13.8047	14.6964	2.60029	0.963335836	2.89654755	2.986850813	0.475203386	2.408415101	2.498718363
RNA-Seq	Trinity	XLOC_060301	302	n/a	n/a	n/a	n/a	n/a	n/a	n/a	n/a	n/a	n/a	305.156	253.535	352.39	391.907	54.5008	-0.267362035	0.207626027	0.360964385	2.21783561	2.692823673	2.84616203
WGS	ABySS	XLOC_062169	434	n/a	n/a	n/a	n/a	n/a	n/a	n/a	n/a	n/a	n/a	147.472	242.611	162.732	150.52	1024.45	0.718203903	0.142056913	0.029514134	-2.078132704	-2.654279694	-2.766822473
RNA-Seq	Trinity	XLOC_062679	227	n/a	n/a	n/a	n/a	n/a	n/a	n/a	n/a	n/a	n/a	26.5572	90.349	60.3413	51.8281	374.09	1.766405586	1.184042731	0.964631459	-2.049804863	-2.632167717	-2.85157899
RNA-Seq	Trinity	XLOC_063472	521	n/a	n/a	n/a	n/a	n/a	n/a	n/a	n/a	n/a	n/a	1.6204	8.35479	46.2921	49.6197	9.30348	2.366253576	4.836344115	4.936491117	-0.155166896	2.314923643	2.415070645
WGS	ABySS	XLOC_063550	2874	n/a	n/a	n/a	n/a	n/a	n/a	n/a	n/a	n/a	n/a	3.07021	1.85492	2.28358	2.57438	14.1663	-0.726980371	-0.427040006	-0.254112315	-2.933034128	-2.633093762	-2.460166071
RNA-Seq	Trinity	XLOC_064113	602	n/a	n/a	n/a	n/a	n/a	n/a	n/a	n/a	n/a	n/a	11.4171	4.03756	5.05491	4.28547	61.9847	-1.499640641	-1.175438934	-1.413670903	-3.940356547	-3.616154841	-3.854386809
RNA-Seq	Trinity	XLOC_067820	214	n/a	n/a	n/a	n/a	n/a	n/a	n/a	n/a	n/a	n/a	79.2251	50.4224	28.9644	35.3221	337.646	-0.651892787	-1.451676796	-1.165386458	-2.743374772	-3.543158782	-3.256868443
RNA-Seq	Trinity	XLOC_068132	236	n/a	n/a	n/a	n/a	n/a	n/a	n/a	n/a	n/a	n/a	36.2991	34.0204	42.6821	34.8197	224.558	-0.093533675	0.233697381	-0.060030005	-2.722616111	-2.395385055	-2.689112442
RNA-Seq	Trans-ABySS	XLOC_070666	194	n/a	n/a	n/a	n/a	n/a	n/a	n/a	n/a	n/a	n/a	54.346	52.0319	53.152	59.3452	378.634	-0.062777463	-0.032049875	0.126957492	-2.863335671	-2.832608082	-2.673600715
RNA-Seq	Trinity	XLOC_071923	1037	n/a	n/a	n/a	n/a	n/a	n/a	n/a	n/a	n/a	n/a	3.22561	10.7889	28.7934	30.5774	5.88497	1.741903858	3.158094234	3.244821818	0.874440814	2.29063119	2.377358773
RNA-Seq	Trans-ABySS	XLOC_072495	168	n/a	n/a	n/a	n/a	n/a	n/a	n/a	n/a	n/a	n/a	223.251	130.634	80.7579	71.5617	1100.03	-0.773136203	-1.466991337	-1.641407072	-3.07394053	-3.767795663	-3.942211399
RNA-Seq	Trans-ABySS	XLOC_074874	175	n/a	n/a	n/a	n/a	n/a	n/a	n/a	n/a	n/a	n/a	49.9571	58.0929	84.8612	59.0644	843.514	0.21767212	0.764415348	0.241599103	-3.859978257	-3.313235029	-3.836051274
RNA-Seq	Trinity	XLOC_075182	428	n/a	n/a	n/a	n/a	n/a	n/a	n/a	n/a	n/a	n/a	15.1695	18.1996	15.849	18.1045	200.865	0.262733209	0.063218282	0.2551748	-3.464247554	-3.663762481	-3.471805963
RNA-Seq	Trinity	XLOC_075519	627	n/a	n/a	n/a	n/a	n/a	n/a	n/a	n/a	n/a	n/a	8.96752	22.9343	6.15504	5.82429	409.38	1.354725911	-0.542940825	-0.622626867	-4.157861843	-6.055528579	-6.135214621
RNA-Seq	Trinity	XLOC_075997	711	n/a	n/a	n/a	n/a	n/a	n/a	n/a	n/a	n/a	n/a	35.6945	12.2086	1.16711	1.49518	1.10052	-1.547804022	-4.934689347	-4.577310712	3.471640502	0.084755177	0.442133812
RNA-Seq	Trinity	XLOC_076411	252	n/a	n/a	n/a	n/a	n/a	n/a	n/a	n/a	n/a	n/a	60.3151	115.537	49.0892	42.7637	1411.14	0.937763806	-0.297113573	-0.496132546	-3.610434281	-4.84531166	-5.044330632
RNA-Seq	Trinity	XLOC_082042	260	n/a	n/a	n/a	n/a	n/a	n/a	n/a	n/a	n/a	n/a	29.1951	26.9236	37.4749	38.2928	555.893	-0.116854925	0.360198376	0.391346902	-4.367863982	-3.890810682	-3.859662155
RNA-Seq	Trans-ABySS	XLOC_082397	54	n/a	n/a	n/a	n/a	n/a	n/a	n/a	n/a	n/a	n/a	1732530	316623	93437.9	82543	332212	-2.45204237	-4.212728673	-4.39159065	-0.069338128	-1.830024431	-2.008886408
RNA-Seq	Trans-ABySS	XLOC_083167	340	n/a	n/a	n/a	n/a	n/a	n/a	n/a	n/a	n/a	n/a	20.9529	19.3503	18.5409	19.6117	141.939	-0.114794001	-0.176438659	-0.095435337	-2.874843209	-2.936487866	-2.855484544
RNA-Seq	Trinity	XLOC_084629	366	n/a	n/a	n/a	n/a	n/a	n/a	n/a	n/a	n/a	n/a	18.2448	11.1757	10.9289	9.56732	65.022	-0.707120137	-0.739337136	-0.931298578	-2.540562734	-2.572779732	-2.764741174
RNA-Seq	Trinity	XLOC_090522	323	n/a	n/a	n/a	n/a	n/a	n/a	n/a	n/a	n/a	n/a	2.28639	12.7915	5.21744	7.51954	111.024	2.484042036	1.190270593	1.717572898	-3.117614219	-4.411385662	-3.884083357
RNA-Seq	Trans-ABySS	XLOC_090548	369	n/a	n/a	n/a	n/a	n/a	n/a	n/a	n/a	n/a	n/a	23.166	13.4052	13.2166	12.9066	83.9172	-0.789216216	-0.809657872	-0.84389996	-2.646173798	-2.666615453	-2.700857541
RNA-Seq	Trinity	XLOC_092104	265	n/a	n/a	n/a	n/a	n/a	n/a	n/a	n/a	n/a	n/a	61.3322	100.433	80.3312	99.3135	688.429	0.711516777	0.389315726	0.69534514	-2.777074488	-3.099275539	-2.793246126
RNA-Seq	Trinity	XLOC_092621	529	n/a	n/a	n/a	n/a	n/a	n/a	n/a	n/a	n/a	n/a	8.51044	5.67693	7.03993	7.93033	46.3534	-0.58412277	-0.273672639	-0.101852822	-3.029492306	-2.719042175	-2.547222357
RNA-Seq	Trinity	XLOC_093138	847	n/a	n/a	n/a	n/a	n/a	n/a	n/a	n/a	n/a	n/a	48.1168	36.238	55.0318	46.2146	7.68587	-0.409037365	0.193724818	-0.058192003	2.237222855	2.839985038	2.588068216
RNA-Seq	Trans-ABySS	XLOC_095359	517	n/a	n/a	n/a	n/a	n/a	n/a	n/a	n/a	n/a	n/a	1.89429	9.55104	53.0148	56.4643	10.4826	2.334000623	4.806666052	4.897609883	-0.134266853	2.338398576	2.429342407
RNA-Seq	Trans-ABySS	XLOC_097520	208	n/a	n/a	n/a	n/a	n/a	n/a	n/a	n/a	n/a	n/a	70.985	34.504	38.8782	38.5359	335.38	-1.040750577	-0.868552772	-0.881311112	-3.280961132	-3.108763327	-3.121521668
RNA-Seq	Trans-ABySS	XLOC_098886	660	n/a	n/a	n/a	n/a	n/a	n/a	n/a	n/a	n/a	n/a	14.4029	17.6053	9.0975	9.82987	83.7192	0.289650486	-0.662817274	-0.551115083	-2.249548714	-3.202016474	-3.090314283
RNA-Seq	Trans-ABySS	XLOC_107210	325	n/a	n/a	n/a	n/a	n/a	n/a	n/a	n/a	n/a	n/a	31.1846	36.0906	31.9518	35.4432	398.442	0.210789374	0.035063454	0.18467511	-3.464674698	-3.640400618	-3.490788962
RNA-Seq	Trans-ABySS	XLOC_107321	217	n/a	n/a	n/a	n/a	n/a	n/a	n/a	n/a	n/a	n/a	8.60046	21.7167	51.6706	60.9496	407.298	1.336319162	2.586857906	2.825131022	-4.229207933	-2.978669189	-2.740396072
RNA-Seq	Trans-ABySS	XLOC_107468	585	n/a	n/a	n/a	n/a	n/a	n/a	n/a	n/a	n/a	n/a	16.8789	12.9599	12.5274	12.9973	67.2647	-0.3811663	-0.430133865	-0.377008931	-2.375795002	-2.424762567	-2.371637633
RNA-Seq	Trans-ABySS	XLOC_110238	156	n/a	n/a	n/a	n/a	n/a	n/a	n/a	n/a	n/a	n/a	250.653	145.893	94.8952	98.3843	1151.55	-0.780780838	-1.401284482	-1.349191485	-2.980594485	-3.601098129	-3.549005133
RNA-Seq	Trans-ABySS	XLOC_110270	325	n/a	n/a	n/a	n/a	n/a	n/a	n/a	n/a	n/a	n/a	4.45495	12.3036	7.98796	11.8144	151.22	1.465599361	0.842417869	1.407065218	-3.619496549	-4.242678041	-3.678030691
RNA-Seq	Trans-ABySS	XLOC_110588	448	n/a	n/a	n/a	n/a	n/a	n/a	n/a	n/a	n/a	n/a	42.8043	22.9893	12.7968	9.7974	185.347	-0.896793195	-1.741972642	-2.127284885	-3.011194321	-3.856373768	-4.241686011
RNA-Seq	Trans-ABySS	XLOC_110768	804	n/a	n/a	n/a	n/a	n/a	n/a	n/a	n/a	n/a	n/a	12.5426	31.239	15.0922	15.0838	355.824	1.316511831	0.266966683	0.266163486	-3.509743646	-4.559288793	-4.560091991
RNA-Seq	Trans-ABySS	XLOC_111734	230	n/a	n/a	n/a	n/a	n/a	n/a	n/a	n/a	n/a	n/a	33.4092	104.385	30.6948	25.5851	1732.2	1.643597071	-0.122251167	-0.384941565	-4.052619195	-5.818467434	-6.081157831
RNA-Seq	Trans-ABySS	XLOC_112597	265	n/a	n/a	n/a	n/a	n/a	n/a	n/a	n/a	n/a	n/a	116.908	75.5766	45.1006	58.9678	357.374	-0.629362135	-1.374155125	-0.987374381	-2.241423156	-2.986216146	-2.599435402
RNA-Seq	Trans-ABySS	XLOC_113704	542	n/a	n/a	n/a	n/a	n/a	n/a	n/a	n/a	n/a	n/a	8.63598	23.5526	62.6869	45.5942	4.32892	1.447454522	2.85973218	2.400418505	2.443807285	3.856084943	3.396771268
RNA-Seq	Trans-ABySS	XLOC_114018	247	n/a	n/a	n/a	n/a	n/a	n/a	n/a	n/a	n/a	n/a	546.114	459.741	631.011	707.929	104.145	-0.248380809	0.208463013	0.374402534	2.142227755	2.599071578	2.765011099
RNA-Seq	Trans-ABySS	XLOC_114314	372	n/a	n/a	n/a	n/a	n/a	n/a	n/a	n/a	n/a	n/a	20.05	37.714	14.3096	14.2017	694.851	0.911497936	-0.486618892	-0.4975386	-4.203531569	-5.601648398	-5.612568106
RNA-Seq	Trans-ABySS	XLOC_121783	330	n/a	n/a	n/a	n/a	n/a	n/a	n/a	n/a	n/a	n/a	290.996	51.3399	20.2788	22.7532	119.899	-2.502846931	-3.842955134	-3.676857957	-1.223667235	-2.563775438	-2.397678262
RNA-Seq	Trans-ABySS	XLOC_122764	717	n/a	n/a	n/a	n/a	n/a	n/a	n/a	n/a	n/a	n/a	18.7594	7.59543	3.5651	3.16221	22.7314	-1.304410138	-2.395599236	-2.568608601	-1.581482993	-2.672672091	-2.845681456
RNA-Seq	Trans-ABySS	XLOC_132147	253	n/a	n/a	n/a	n/a	n/a	n/a	n/a	n/a	n/a	n/a	50.0853	25.3251	24.858	23.7895	286.33	-0.98381927	-1.010677009	-1.074062286	-3.499038965	-3.525896704	-3.58928198
RNA-Seq	Trans-ABySS	XLOC_141663	705	n/a	n/a	n/a	n/a	n/a	n/a	n/a	n/a	n/a	n/a	51.0787	26.0801	3.8986	3.47072	11.8959	-0.969772406	-3.711693762	-3.879414922	1.132484976	-1.609436379	-1.777157539
RNA-Seq	Trans-ABySS	XLOC_168869	322	n/a	n/a	n/a	n/a	n/a	n/a	n/a	n/a	n/a	n/a	19.3782	8.67487	11.8917	10.6693	120.05	-1.159520525	-0.704479595	-0.860969042	-3.790649455	-3.335608525	-3.492097973
RNA-Seq	Trans-ABySS	XLOC_169115	231	n/a	n/a	n/a	n/a	n/a	n/a	n/a	n/a	n/a	n/a	64.8787	38.2252	52.715	39.2382	353.203	-0.763220862	-0.299531374	-0.725486051	-3.207901642	-2.744212154	-3.17016683
RNA-Seq	Trans-ABySS	XLOC_179823	265	n/a	n/a	n/a	n/a	n/a	n/a	n/a	n/a	n/a	n/a	42.8663	19.6117	22.2327	24.9245	325.172	-1.128129298	-0.947160733	-0.782279333	-4.051416532	-3.870447966	-3.705566566
RNA-Seq	Trans-ABySS	XLOC_199252	640	n/a	n/a	n/a	n/a	n/a	n/a	n/a	n/a	n/a	n/a	40.0649	14.6378	12.6864	9.98704	96.5086	-1.452640137	-1.65905614	-2.004209821	-2.72095877	-2.927374773	-3.272528454
RNA-Seq	Trans-ABySS	XLOC_203358	236	n/a	n/a	n/a	n/a	n/a	n/a	n/a	n/a	n/a	n/a	35.3816	65.8299	30.051	28.7593	397.368	0.895743716	-0.23558629	-0.298970729	-2.593660786	-3.724990792	-3.788375231
RNA-Seq	Trans-ABySS	XLOC_204233	227	n/a	n/a	n/a	n/a	n/a	n/a	n/a	n/a	n/a	n/a	30.6544	101.982	68.2323	58.3738	416.183	1.734148464	1.154360681	0.92922682	-2.028903499	-2.608691283	-2.833825143
RNA-Seq	Trans-ABySS	XLOC_204980	1360	n/a	n/a	n/a	0	n/a	n/a	n/a	n/a	n/a	n/a	14.0854	9.06598	9.83997	9.2693	53.6221	-0.635665651	-0.517474712	-0.603668236	-2.564292837	-2.446101898	-2.532295422
RNA-Seq	Trans-ABySS	XLOC_013081	369	XR_177503.2	454	166/213	0	uncharacterized LOC101175589	n/a	n/a	n/a	n/a	n/a	6.34308	70.8853	75.2537	70.2122	65.686	3.482231033	3.568507074	3.468466291	0.109900562	0.196176602	0.096135819
RNA-Seq	Trinity	XLOC_058898	466	XR_177503.2	454	192/245	7.00E-49	uncharacterized LOC101175589	n/a	n/a	n/a	n/a	n/a	3.46864	39.98	43.2116	39.9273	37.7242	3.526836452	3.63897663	3.524933496	0.083788166	0.195928344	0.08188521
RNA-Seq	Trans-ABySS	XLOC_009763	303	XM_005810207.1	1153	165/227	3.00E-23	uncharacterized LOC102233786	n/a	n/a	n/a	n/a	n/a	12.8982	94.9921	20.0097	18.7333	182.032	2.880637792	0.633529792	0.538435317	-0.938312647	-3.185420646	-3.280515121
RNA-Seq	Trinity	XLOC_078484	630	XM_007567606.1	3448	379/506	4.00E-89	uncharacterized LOC103148713	n/a	n/a	n/a	n/a	n/a	2.13334	6.28698	4.90788	5.07149	40.603	1.559253261	1.201986062	1.249295759	-2.691147248	-3.048414447	-3.001104749
RNA-Seq	Trans-ABySS	XLOC_091466	621	XM_007567606.1	3448	379/506	2.00E-89	uncharacterized LOC103148713	n/a	n/a	n/a	n/a	n/a	2.51561	7.16908	5.66947	5.83527	46.1465	1.510879716	1.172305598	1.213891136	-2.686361334	-3.024935451	-2.983349914
RNA-Seq	Trinity	XLOC_049693	497	XR_520011.1	767	173/226	5.00E-37	uncharacterized LOC103353523	n/a	n/a	n/a	n/a	n/a	5.1245	9.85161	35.0048	36.0911	3.94459	0.942948269	2.772069612	2.816159963	1.320484162	3.149605504	3.193695855
RNA-Seq	Trans-ABySS	XLOC_155556	440	XR_520011.1	767	151/195	3.00E-33	uncharacterized LOC103353523	n/a	n/a	n/a	n/a	n/a	7.15041	13.6634	48.3861	50.2857	5.4483	0.934218656	2.758494788	2.814050319	1.326438479	3.15071461	3.206270142
RNA-Seq	Trinity	XLOC_003990	2087	XM_008282441.1	3604	1610/2084	0	uncharacterized LOC103357750	n/a	n/a	n/a	n/a	n/a	1.76271	4.31451	4.39953	3.90509	36.3843	1.291401579	1.319554267	1.147560655	-3.076047428	-3.04789474	-3.219888351
RNA-Seq	Trans-ABySS	XLOC_057545	701	XM_008282441.1	3604	545/699	8.00E-158	uncharacterized LOC103357750	n/a	n/a	n/a	n/a	n/a	2.66131	8.11029	7.98801	5.75497	60.6653	1.607616931	1.585699568	1.112671837	-2.903046137	-2.9249635	-3.397991231
RNA-Seq	Trans-ABySS	XLOC_073725	535	XM_008282441.1	3604	413/531	4.00E-111	uncharacterized LOC103357750	n/a	n/a	n/a	n/a	n/a	2.48023	6.08877	6.06434	5.80368	41.3916	1.295676904	1.289876728	1.226494062	-2.765115295	-2.770915471	-2.834298137
RNA-Seq	Trans-ABySS	XLOC_075417	572	XM_008282441.1	3604	443/582	3.00E-113	uncharacterized LOC103357750	n/a	n/a	n/a	n/a	n/a	3.09843	4.29	6.54556	5.45218	36.9891	0.469440273	1.078979251	0.815295817	-3.108050645	-2.498511667	-2.7621951
RNA-Seq	Trans-ABySS	XLOC_020003	848	XM_008305493.1	2994	646/846	8.00E-171	uncharacterized LOC103375268	n/a	n/a	n/a	n/a	n/a	2.33714	6.78066	6.02616	6.0323	57.1456	1.536681548	1.366494821	1.367964021	-3.07514481	-3.245331537	-3.243862337
RNA-Seq	Trans-ABySS	XLOC_102617	849	XM_008434469.1	9246	675/848	0	uncharacterized LOC103479810	n/a	n/a	n/a	n/a	n/a	1.97995	2.86429	2.76635	3.11075	24.4039	0.53271357	0.4825197	0.651798457	-3.090862251	-3.14105612	-2.971777364
RNA-Seq	Trinity	XLOC_041841	201	XM_010738958.1	1741	132/179	8.00E-16	uncharacterized LOC104925355	n/a	n/a	n/a	n/a	n/a	41.5553	266.019	48.4016	51.8201	609.156	2.678424895	0.220022248	0.318479308	-1.195282446	-3.653685094	-3.555228033
RNA-Seq	Trinity	XLOC_088467	677	XM_010738958.1	1741	403/542	2.00E-88	uncharacterized LOC104925355	n/a	n/a	n/a	n/a	n/a	5.17933	33.03	5.83802	6.97155	77.35	2.672939582	0.172713671	0.428713967	-1.227624322	-3.727850233	-3.471849937
WGS	ABySS	XLOC_014264	853	XM_007230335.1	3010	214/313	2.00E-13	uncharacterized protein LOC103027396	n/a	n/a	n/a	n/a	n/a	1.55963	7.34191	5.37773	4.59921	62.8035	2.234951619	1.785793513	1.560182262	-3.096617627	-3.545775733	-3.771386984
RNA-Seq	Trinity	XLOC_000251	604	XM_008435664.1	1957	219/333	3.00E-10	uncharacterized protein YBL113C-like	n/a	n/a	n/a	n/a	n/a	8.59409	10.8618	11.7358	10.6127	76.122	0.337846413	0.449499399	0.304374951	-2.809050263	-2.697397277	-2.842521725
RNA-Seq	Trinity	XLOC_005464	1065	NR_121949.1	2914	949/1057	0	*Vibrio campbellii* strain ATCC BAA-1116 23S ribosomal RNA gene	n/a	biological_process	GO:0006364	rRNA processing	n/a	11.3355	13.1685	1.87852	1.58481	77.5347	0.216242992	-2.593179648	-2.838466235	-2.557751101	-5.36717374	-5.612460327
RNA-Seq	Trans-ABySS	XLOC_009352	378	NR_121949.1	2914	365/378	5.00E-174	*Vibrio campbellii* strain ATCC BAA-1116 23S ribosomal RNA gene	n/a	biological_process	GO:0006364	rRNA processing	n/a	3.66433	22.5533	3.32783	1.95987	296.942	2.621717204	-0.138967698	-0.902791472	-3.718770716	-6.479455619	-7.243279393
RNA-Seq	Trans-ABySS	XLOC_009651	590	NR_121949.1	2914	542/592	0	*Vibrio campbellii* strain ATCC BAA-1116 23S ribosomal RNA gene	n/a	biological_process	GO:0006364	rRNA processing	n/a	17.8419	16.882	2.77348	3.42843	96.8333	-0.079783428	-2.68550003	-2.379649286	-2.520017432	-5.125734034	-4.81988329
RNA-Seq	Trans-ABySS	XLOC_003074	167	KP120945.1	1429	167/167	4.00E-78	*Vibrio parahaemolyticus* strain S11 16S ribosomal RNA gene	n/a	biological_process	GO:0006364	rRNA processing	n/a	106.32	147.392	58.1088	40.9766	2079.35	0.471245212	-0.871584443	-1.375540822	-3.818402489	-5.161232143	-5.665188523
RNA-Seq	Trans-ABySS	XLOC_011323	563	KP183027.1	1519	540/558	0	Uncultured *Vibrio sp*. clone 12S_133 16S ribosomal RNA	n/a	biological_process	GO:0006364	rRNA processing	n/a	16.7698	35.9289	5.71563	2.9728	33.2232	1.099279283	-1.552881051	-2.495971171	0.112953729	-2.539206606	-3.482296726
WGS	ABySS	XLOC_008959	1544	XM_005800760.1	1580	259/305	2.00E-90	barrier-to-autointegration factor-like	banf1, baf	molecular_function	GO:0003677	DNA binding	Nucleic Acid Modification related	23.7183	12.3221	10.0378	11.6191	61.3109	-0.94475246	-1.240557503	-1.029502285	-2.314895434	-2.610700476	-2.399645259
RNA-Seq	Trinity	XLOC_025325	1158	XM_005800760.1	1580	50/64	0.004	barrier-to-autointegration factor-like	banf1, baf	molecular_function	GO:0003677	DNA binding	Nucleic Acid Modification related	25.0171	13.5693	12.2399	13.9853	89.6951	-0.882568263	-1.03132279	-0.839003359	-2.724682875	-2.873437402	-2.681117972
RNA-Seq	Trinity	XLOC_039853	1071	XM_008433318.1	1243	365/489	8.00E-92	cmpk1 cytidine monophosphate (UMP-CMP) kinase 1, cytosolic	cmpk1, umpk, umk, cmk	molecular_function	GO:0004127	cytidylate kinase activity	Nucleic Acid Modification related	19.6429	12.7342	13.5136	14.2083	100.442	-0.625299611	-0.539595882	-0.46727399	-2.979582428	-2.893878698	-2.821556807
RNA-Seq	Trans-ABySS	XLOC_179703	179	XM_008433318.1	1243	130/164	3.00E-30	cmpk1 cytidine monophosphate (UMP-CMP) kinase 1, cytosolic	cmpk1, umpk, umk, cmk	molecular_function	GO:0004127	cytidylate kinase activity	Nucleic Acid Modification related	205.729	98.0385	61.5461	131.394	795.293	-1.069324857	-1.741005828	-0.646845776	-3.020066157	-3.691747127	-2.597587075
WGS	ABySS	XLOC_029694	2527	XM_003437795.2	1263	756/991	0	cmpk1 UMP-CMP kinase-like	cmpk1, umpk, umk, cmk	biological_process	GO:0006165	nucleoside diphosphate phosphorylation	Nucleic Acid Modification related	12.6981	7.29836	6.76987	8.23867	54.8776	-0.798968424	-0.907412609	-0.624129283	-2.910573168	-3.019017353	-2.735734027
RNA-Seq	Trans-ABySS	XLOC_002834	82	AF467776.1	1234	76/80	3.00E-25	EF1alpha	eef1a1, ccs3	molecular_function	GO:0003746	translation elongation factor activity	Nucleic Acid Modification related	35031.2	39902.7	62258.7	49550.3	3558.05	0.18784596	0.829635044	0.500253383	3.48732768	4.129116763	3.799735103
RNA-Seq	Trans-ABySS	XLOC_002879	87	AF467776.1	1234	83/87	4.00E-29	EF1alpha	eef1a1, ccs3	molecular_function	GO:0003746	translation elongation factor activity	Nucleic Acid Modification related	11056.3	12942.9	17754.6	17353	2346.8	0.227292238	0.68332419	0.650316431	2.463394103	2.919426055	2.886418296
RNA-Seq	Trans-ABySS	XLOC_006546	117	XM_008292885.1	1491	100/107	2.00E-35	EF1A elongation factor 1-alpha	eef1a1, ccs3	molecular_function	GO:0003746	translation elongation factor activity	Nucleic Acid Modification related	7199.21	8144.37	13029.5	10784.9	1064.24	0.177964503	0.855871215	0.583102292	2.935979571	3.613886283	3.34111736
RNA-Seq	Trinity	XLOC_072219	751	XM_008291180.1	4099	700/751	0	transcription factor MafB-like	mafb, krml	biological_process	GO:0010866	transcription factor binding	Nucleic Acid Modification related	3.20248	15.5473	3.2956	2.12674	88.9037	2.279402593	0.041351587	-0.59054589	-2.515579404	-4.75363041	-5.385527887
RNA-Seq	Trans-ABySS	XLOC_009611	422	XM_007543902.1	1332	298/388	3.00E-73	GATA zinc finger domain-containing protein 14-like	muc5ac, muc2	molecular_function	GO:0043565	sequence-specific DNA binding	Nucleic Acid Modification related	16.9452	40.8287	29.8721	32.1126	162.699	1.268706968	0.817921998	0.922262813	-1.994549846	-2.445334816	-2.340994001
RNA-Seq	Trans-ABySS	XLOC_011402	500	XM_005466488.1	1353	360/494	6.00E-70	GATA zinc finger domain-containing protein 14-like	muc5ac, muc2	molecular_function	GO:0043565	sequence-specific DNA binding	Nucleic Acid Modification related	2.31124	8.17043	7.68556	10.2396	48.982	1.82174493	1.733483307	2.147420376	-2.583767769	-2.672029393	-2.258092323
RNA-Seq	Trans-ABySS	XLOC_012533	686	XM_005466488.1	1353	115/157	2.00E-14	GATA zinc finger domain-containing protein 14-like	muc5ac, muc2	molecular_function	GO:0043565	sequence-specific DNA binding	Nucleic Acid Modification related	9.27326	11.6381	12.2161	11.5138	82.239	0.327707037	0.397635267	0.312215547	-2.820967174	-2.751038945	-2.836458665
WGS	ABySS	XLOC_021386	647	XM_005466488.1	1353	497/651	9.00E-128	GATA zinc finger domain-containing protein 14-like	muc5ac, muc2	molecular_function	GO:0043565	sequence-specific DNA binding	Nucleic Acid Modification related	6.4286	9.01983	10.2787	8.6754	81.1705	0.488595657	0.67708132	0.432425692	-3.169783353	-2.981297689	-3.225953317
WGS	ABySS	XLOC_048860	972	XM_005466488.1	1353	655/927	1.00E-123	GATA zinc finger domain-containing protein 14-like	muc5ac, muc2	molecular_function	GO:0043565	sequence-specific DNA binding	Nucleic Acid Modification related	3.90866	6.30211	6.43154	6.49588	34.9304	0.689160841	0.71849013	0.732850886	-2.470576322	-2.441247034	-2.426886277
RNA-Seq	Trinity	XLOC_001258	118	XM_007543902.1	1332	92/116	8.00E-16	GATA zinc finger domain-containing protein 14-like	muc5ac, muc2	molecular_function	GO:0043565	sequence-specific DNA binding	Nucleic Acid Modification related	351.759	971.644	630.566	741.492	6180.04	1.465840486	0.842060046	1.075843791	-2.669116448	-3.292896889	-3.059113143
RNA-Seq	Trans-ABySS	XLOC_009438	125	XM_008436116.1	2319	102/131	9.00E-18	GATA zinc finger domain-containing protein 14-like	muc5ac, muc2	molecular_function	GO:0043565	sequence-specific DNA binding	Nucleic Acid Modification related	483.131	683.164	576.47	506.471	4086.19	0.499817527	0.254831105	0.068065238	-2.58045243	-2.825438852	-3.012204719
RNA-Seq	Trans-ABySS	XLOC_009524	103	XM_008436116.1	2319	80/97	4.00E-16	GATA zinc finger domain-containing protein 14-like	muc5ac, muc2	molecular_function	GO:0043565	sequence-specific DNA binding	Nucleic Acid Modification related	613.952	629.795	879.249	1169.83	6586.72	0.036756437	0.518145922	0.93010112	-3.386606014	-2.905216529	-2.493261331
WGS	ABySS	XLOC_024149	1937	XM_010749954.1	1833	904/1188	0	transcriptional regulator Myc-B-like	myc	molecular_function	GO:0008134	perinuclear region of cytoplasm	Nucleic Acid Modification related	3.83541	5.77666	20.3099	20.6623	2.23631	0.590854776	2.404730423	2.429548142	1.369115394	3.182991041	3.20780876

RNA-Seq	Trinity	XLOC_047528	2955	XM_008292023.1	4511	1178/1687	0	nuclear factor erythroid 2-related factor 1-like	nfe2l1, nrf1, tcf11	molecular_function	GO:0050262	sequence-specific DNA binding transcription factor activity	Nucleic Acid Modification related	2.34126	6.72853	17.0646	16.7404	2.63675	1.523006193	2.865649536	2.837976939	1.351530559	2.694173902	2.666501305
WGS	ABySS	XLOC_057800	5762	XM_008292023.1	4511	1178/1687	0	nuclear factor erythroid 2-related factor 1-like	nfe2l1, nrf1, tcf11	molecular_function	GO:0003700	sequence-specific DNA binding transcription factor activity	Nucleic Acid Modification related	2.03854	6.66462	16.1938	17.4237	2.87951	1.708986353	2.989833394	3.095442849	1.210699287	2.491546328	2.597155782
WGS	ABySS	XLOC_025598	3002	XM_008279547.1	2748	1599/1951	0	bifunctional 3'-phosphoadenosine 5'-phosphosulfate synthase 2-like	papss2, atpsk2	molecular_function	GO:0004020	adenylylsulfate kinase activity	Nucleic Acid Modification related	7.2166	6.73459	6.56785	6.41013	29.6843	-0.099729174	-0.135898113	-0.170965676	-2.14003807	-2.176207009	-2.211274572
RNA-Seq	Trinity	XLOC_007218	716	XM_007572624.1	4249	458/632	7.00E-93	peroxisome proliferator-activated receptor gamma, coactivator-related 1	pprc1, prc	cellular_component	GO:0031012	transcription factor binding	Nucleic Acid Modification related	1.15762	1.51117	2.38848	2.44296	20.083	0.384504214	1.044931044	1.077468489	-3.732236924	-3.071810094	-3.039272648
WGS	ABySS	XLOC_026735	2505	XM_008282397.1	4389	1420/2099	0	peroxisome proliferator-activated receptor gamma, coactivator-related 1	pprc1, prc	molecular_function	GO:0008134	transcription factor binding	Nucleic Acid Modification related	0.980819	1.09741	1.2493	1.48125	8.14104	0.162043796	0.349061128	0.594756322	-2.89111048	-2.704093148	-2.458397953
WGS	ABySS	XLOC_053648	1280	XM_008423981.1	1237	793/949	0	RNA terminal phosphate cyclase-like 1	rcl1, rnac, rpcl1	cellular_component	GO:0005886	RNA-3'-phosphate cyclase activity	Nucleic Acid Modification related	33.2183	68.2428	186.658	178.838	13.4036	1.038698599	2.490347195	2.428603169	2.348056303	3.799704899	3.737960874
WGS	ABySS	XLOC_032776	341	AJ890104.1	319	288/316	6.00E-121	7SK small nuclear RNA	rn7sk	cellular_component	GO:0005575	cellular_component	Nucleic Acid Modification related	494.935	134.893	74.0496	74.9263	103.328	-1.875423583	-2.74067522	-2.723694953	0.384584233	-0.480667404	-0.463687136
RNA-Seq	Trans-ABySS	XLOC_009541	500	AB120309.1	1800	473/504	0	18S Ribosomal RNA	rps4y1, rps4x, ccg2	molecular_function	GO:0003735	structural constitutent of ribosome	Nucleic Acid Modification related	79.0445	9.53217	7.38417	7.35521	15.5784	-3.051788495	-3.420157409	-3.425826644	-0.708670481	-1.077039395	-1.082708629
RNA-Seq	Trinity	XLOC_031627	501	XM_003452437.2	2395	117/122	3.00E-46	histone-lysine N-methyltransferase SETDB1-B-like	setdb1, eset	cellular_component	GO:0043231	intracellular membrane-bounded organelle	Nucleic Acid Modification related	25.2774	36.972	27.8923	26.6719	298.033	0.548585011	0.142018827	0.077472523	-3.01096709	-3.417533274	-3.482079578
RNA-Seq	Trans-ABySS	XLOC_008787	509	XM_003452437.2	2395	117/122	2.00E-46	histone-lysine N-methyltransferase SETDB1-B-like	setdb1, eset	cellular_component	GO:0043231	intracellular membrane-bounded organelle	Nucleic Acid Modification related	28.1128	39.7568	30.4202	28.2728	317.347	0.499974488	0.113802487	0.008187616	-2.996787659	-3.382959661	-3.488574532
RNA-Seq	Trans-ABySS	XLOC_019399	1761	XM_008294582.1	1974			solute carrier family 28 member 3	slc28a3, cnt3	molecular_function	GO:0005215	nucleoside diphosphate phosphorylation	Nucleic Acid Modification related	8.17291	13.0954	16.1299	14.477	1.70867	0.680138375	0.980815742	0.824840918	2.93811443	3.238791797	3.082816973
WGS	ABySS	XLOC_008910	5972	XM_005448177.1	6058	2476/3452	0	serine/arginine repetitive matrix protein 2-like	srrm2, srm300	biological_process	GO:0010506	poly (A) RNA binding	Nucleic Acid Modification related	5.25019	2.15993	0.870346	0.957299	8.73614	-1.281385076	-2.59270868	-2.455328126	-2.016011419	-3.327335023	-3.189954469
RNA-Seq	Trinity	XLOC_055066	2317	XM_008293211.1	9087	1839/2334	0	serine/arginine repetitive matrix protein 2-like	srrm2, srm300	molecular_function	GO:0044822	poly (A) RNA binding	Nucleic Acid Modification related	4.41331	1.69006	0.989497	1.20187	8.77085	-1.384786623	-2.15709385	-1.876580233	-2.375642198	-3.147949425	-2.867435808
RNA-Seq	Trans-ABySS	XLOC_014724	726	XM_003459808.2	778	413/503	2.00E-139	anterior gradient protein 2 homolog	agr2	biological_process	GO:0070254	mucus secretion	Other Cellular or Enzymatic Processes related	22.3678	12.4194	11.7073	12.349	101.759	-0.84882789	-0.934014974	-0.857029146	-3.034489017	-3.119676101	-3.042690273
RNA-Seq	Trinity	XLOC_091927	752	XM_003459808.2	778	413/503	5.00E-139	anterior gradient protein 2 homolog	agr2	biological_process	GO:0070254	mucus secretion	Other Cellular or Enzymatic Processes related	18.4588	10.4807	9.86213	10.4441	87.2733	-0.81657369	-0.904337592	-0.821620591	-3.057805273	-3.145569174	-3.062852173
WGS	ABySS	XLOC_031051	1527	XM_008303846.1	1852	160/191	2.00E-47	ethanolamine-phosphate phospho-lyase	agxt2l1, etnppl	molecular_function	GO:0030170	pyridoxal phosphate binding	Other Cellular or Enzymatic Processes related	1.84624	2.85562	3.87786	5.18844	21.2456	0.629213905	1.070670613	1.490710724	-2.895288171	-2.453831463	-2.033791352
RNA-Seq	Trans-ABySS	XLOC_011164	1534	XM_007557123.1	1328	837/1023	0	acetylserotonin O-methyltransferase	asmt, hiomt, hiomty, asmty, asmtl, asmtlx	molecular_function	GO:0008171	O-methyltransferase activity	Other Cellular Processes or Enzymatic related	36.1451	28.8678	22.0692	20.7675	182.96	-0.324338917	-0.711765748	-0.799472527	-2.663995201	-3.051422032	-3.139128811
WGS	ABySS	XLOC_052146	2385	XM_007557123.1	1328	837/1023	0	acetylserotonin O-methyltransferase	asmt, hiomt, hiomty, asmty, asmtl, asmtlx	molecular_function	GO:0008171	O-methyltransferase activity	Other Cellular Processes or Enzymatic related	28.3317	25.3384	18.9875	19.5184	167.143	-0.161091743	-0.577367206	-0.537582377	-2.721685603	-3.137961066	-3.098176236
RNA-Seq	Trinity	XLOC_038360	522	XM_008429426.1	2904	254/317	1.00E-70	chromosome LG15 open reading frame	c100r11	biological_process	GO:0030318	melanocyte differentiation	Other Cellular Processes or Enzymatic related	4.8459	9.35788	33.9896	31.8236	33.0864	0.949417095	2.810256845	2.715260509	-1.821984693	0.038855058	-0.056141279
RNA-Seq	Trinity	XLOC_007097	1141	XM_008294176.1	2449	740/950	0	calumenin	calu	cellular_component	GO:0042470	melanosome	Other Cellular Processes or Enzymatic related	9.8679	4.00173	3.57861	3.03388	22.2883	-1.302119265	-1.463343769	-1.701579072	-2.477590846	-2.638815351	-2.877050653
RNA-Seq	Trinity	XLOC_056868	885	XM_008285987.1	2842	555/749	1.00E-47	protein-cysteine N-palmitoyltransferase HHAT-like protein	hhat, mart2, ski1	biological_process	GO:0035176	transferase activity	Other Cellular Processes or Enzymatic related	55.4118	23.2476	10.8325	9.6845	64.3766	-1.253111447	-2.354826996	-2.516443761	-1.469454599	-2.571170147	-2.732786913
RNA-Seq	Trans-ABySS	XLOC_010607	1394	XM_008414801.1	1258	919/1101	0	sas N-acetylneuraminic acid synthase	nans, sas	molecular_function	GO:0003963	N-acylneuraminate-9-phosphate synthase activity	Other Cellular Processes or Enzymatic related	32.3869	25.4329	28.2267	29.7398	156.978	-0.348714408	-0.198349911	-0.123015438	-2.625794503	-2.475430006	-2.400095533
WGS	ABySS	XLOC_031745	1987	XM_008414801.1	1258	908/1089	0	sas N-acetylneuraminic acid synthase	nans, sas	molecular_function	GO:0047444	N-acylneuraminate-9-phosphate synthase activity	Other Cellular Processes or Enzymatic related	20.4821	14.807	17.7984	18.5812	134.202	-0.46808427	-0.202616085	-0.140519964	-3.180054897	-2.914586712	-2.852490591
RNA-Seq	Trans-ABySS	XLOC_013832	243	XM_003449951.2	2065	125/137	2.00E-45	sas sialic acid synthase-like	nans, sas	molecular_function	GO:0047444	catalytic activity	Other Cellular Processes or Enzymatic related	53.4248	18.1117	12.4407	16.6683	495.463	-1.560587635	-2.102441938	-1.680402631	-4.773783452	-5.315637756	-4.893598448
RNA-Seq	Trinity	XLOC_005653	1256	XM_005947638.1	2030	872/1152	0	natterin-3-like	natterin-3	biological_process	GO:0042554	pathogenesis	Other Cellular Processes or Enzymatic related	4.31948	9.92906	1.42496	1.76546	60.5785	1.200799498	-1.599936222	-1.29081351	-2.609076809	-5.409812529	-5.100689816
WGS	ABySS	XLOC_009089	1302	XM_005947638.1	2030	971/1152	0	natterin-3-like	natterin-3	biological_process	GO:0009405	pathogenesis	Other Cellular Processes or Enzymatic related	3.02276	10.4256	2.07583	1.57443	72.9595	1.786192072	-0.542178138	-0.941036821	-2.806965428	-5.135335638	-5.534194322
WGS	ABySS	XLOC_018062	1293	XM_005730593.1	1357	988/1188	0	natterin-3-like	natterin-3	biological_process	GO:0009405	pathogenesis	Other Cellular Processes or Enzymatic related	2.52254	5.57746	0.733832	1.06844	41.7589	1.144731116	-1.781355424	-1.239371253	-2.904403542	-5.830490082	-5.288505911
RNA-Seq	Trans-ABySS	XLOC_043063	462	XM_005947638.1	2030	268/313	3.00E-96	natterin-3-like	natterin-3	biological_process	GO:0009405	pathogenesis	Other Cellular Processes or Enzymatic related	5.30328	21.8721	4.49519	3.47467	143.326	2.044134918	-0.238502825	-0.610008954	-2.712136696	-4.994774439	-5.366280567
RNA-Seq	Trinity	XLOC_047108	721	XM_005947638.1	2030	488/568	0	natterin-3-like	natterin-3	biological_process	GO:0009405	pathogenesis	Other Cellular Processes or Enzymatic related	4.3564	16.8651	3.12841	2.05277	116.866	1.952832541	-0.477706827	-1.085564435	-2.792742488	-5.223281855	-5.831139464
RNA-Seq	Trans-ABySS	XLOC_056750	886	XM_005947638.1	2030	742/885	0	natterin-3-like	natterin-3	biological_process	GO:0009405	pathogenesis	Other Cellular Processes or Enzymatic related	5.3501	16.4997	2.61628	2.12825	102.072	1.624802031	-1.032048908	-1.329898227	-2.629075467	-5.285926406	-5.583775725
RNA-Seq	Trinity	XLOC_058031	592	XM_005730593.1	1357	505/612	2.00E-171	natterin-3-like	natterin-3	biological_process	GO:0009405	pathogenesis	Other Cellular Processes or Enzymatic related	3.15697	10.4307	1.22004	1.75837	100.047	1.724223524	-1.371612103	-0.844301874	-3.261770022	-6.357605649	-5.83029542
RNA-Seq	Trans-ABySS	XLOC_174284	1006	XM_005730593.1	1357	813/941	0	natterin-3-like	natterin-3	biological_process	GO:0009405	pathogenesis	Other Cellular Processes or Enzymatic related	4.89082	10.052	1.27927	1.9695	70.7112	1.039334302	-1.934755581	-1.312246952	-2.814456166	-5.788546049	-5.166037421
RNA-Seq	Trinity	XLOC_024997	717	XM_008306940.1	2924	136/182	4.00E-21	ornithine decarboxylase 1	odc1	molecular_function	GO:0003700	ornithine decarboxylase activity	Other Cellular Processes or Enzymatic related	11.3225	6.26495	5.84421	5.3197	32.0081	-0.853817639	-0.954112615	-1.089775746	-2.35306214	-2.453357117	-2.589020248
RNA-Seq	Trinity	XLOC_073804	609	XM_008422382.1	1007	369/481	2.00E-96	zymogen granule membrane protein 16-like	zg16, zg16a, jcln, jcln1	cellular_component	GO:0042589	zymogen granule membrane	Other Cellular Processes or Enzymatic related	9.95144	20.1328	7.69483	6.08314	49.9903	1.016570624	-0.371015849	-0.710089095	-1.312100354	-2.699686826	-3.038760072
WGS	ABySS	XLOC_006967	1567	XM_005810399.1	1740	286/351	6.00E-90	calcium/calmodulin-dependent protein kinase II inhibitor 1-like	camk2n1	molecular_function	GO:0004860	protein kinase inhibitor activity	Protein Modification related	28.7752	40.0558	85.1695	79.0432	9.6015	0.477185201	1.565510925	1.457815399	2.060679443	3.149005166	3.04130964
RNA-Seq	Trinity	XLOC_096420	1099	XM_008295185.1	1137	883/1063	0	cyclin-dependent kinase 1	cdk1, cdc2	biological_process	GO:0006468	protein phosphorylation	Protein Modification related	11.214	3.35826	1.45454	1.56695	14.6061	-1.73951514	-2.946666099	-2.839269924	-2.120785178	-3.327936137	-3.220539963
WGS	ABySS	XLOC_014000	2161	XM_007577695.1	1198	829/1017	0	carboxypeptidase O	cpo	biological_process	GO:0006508	proteolysis	Protein Modification related	10.0063	4.64632	8.63514	7.85598	0.749936	-1.106748188	-0.212617138	-0.34904545	2.631249133	3.525380183	3.388951871
RNA-Seq	Trans-ABySS	XLOC_011422	1422	XM_008302146.1	4032	911/1160	0	dual specificity phosphatase 27	dusp27	biological_process	GO:0006470	protein dephosphorylation	Protein Modification related	10.2229	20.7391	129.246	124.812	14.962	1.020548774	3.660243213	3.60988023	0.471050252	3.110744691	3.060381707
RNA-Seq	Trinity	XLOC_023173	1352	XM_008302146.1	4032	551/722	1.00E-145	dual specificity phosphatase 27	dusp27	biological_process	GO:0006470	protein dephosphorylation	Protein Modification related	9.08681	15.8386	83.8806	81.0895	15.6574	0.801599001	3.206491363	3.157669299	0.016600154	2.421492516	2.372670452
WGS	ABySS	XLOC_054021	5406	XM_008302146.1	4032	2830/3484	0	dual specificity phosphatase 27	dusp27	biological_process	GO:0006470	protein dephosphorylation	Protein Modification related	4.96999	11.0229	71.5262	71.4763	8.96252	1.149188976	3.847156943	3.846150101	0.298527492	2.996495459	2.995488616
RNA-Seq	Trinity	XLOC_058506	2889	XM_008302146.1	4032	2364/2882	0	dual specificity phosphatase 27	dusp27	biological_process	GO:0006470	protein dephosphorylation	Protein Modification related	7.03455	15.8715	90.5055	90.6582	10.3758	1.17390844	3.685475425	3.687907475	0.613215906	3.124782891	3.127214941
RNA-Seq	Trinity	XLOC_025725	910	XM_008290725.1	2805	733/851	0	fkbp9 FK506 binding protein 9, 63 kDa	fkbp9, fkbp60, fkbp63	biological_process	GO:0061077	chaperone-mediated protein folding	Protein Modification related	10.8578	4.63663	4.35836	3.16754	43.2421	-1.227583305	-1.316874542	-1.777297072	-3.221288077	-3.310579315	-3.771001844
RNA-Seq	Trans-ABySS	XLOC_090466	1206	XM_008290725.1	2805	1051/1215	0	fkbp9 FK506 binding protein 9, 63 kDa	fkbp9, fkbp60, fkbp63	biological_process	GO:0061077	chaperone-mediated protein folding	Protein Modification related	11.9715	4.5592	4.31832	3.62144	41.5962	-1.392751327	-1.471061869	-1.724968552	-3.189599135	-3.267909677	-3.52181636
RNA-Seq	Trans-ABySS	XLOC_184619	524	XM_008290725.1	2805	451/524	3.00E-172	fkbp9 FK506 binding protein 9, 63 kDa	fkbp9, fkbp60, fkbp63	biological_process	GO:0061077	chaperone-mediated protein folding	Protein Modification related	10.8321	4.40864	3.34549	4.13552	39.2119	-1.296907383	-1.695023526	-1.389172312	-3.152885969	-3.551002112	-3.245150899
WGS	ABySS	XLOC_009953	2058	XM_008290725.1	2805	974/1125	0	fkbp9 peptidyl-prolyl cis-trans isomerase FKBP9	fkbp9, fkbp60, fkbp63	biological_process	GO:0000413	protein peptidyl-prolyl isomerization	Protein Modification related	6.99873	2.6799	2.79588	2.40989	27.6377	-1.384913984	-1.323790711	-1.538125856	-3.366386487	-3.305263214	-3.519598359
RNA-Seq	Trinity	XLOC_093308	1029	XM_008283673.1	3957	823/998	0	glutaminase 2 (liver, mitochondrial)	gls2, ga	molecular_function	GO:0004359	glutaminase activity	Protein Modification related	1.34107	6.88961	11.7629	11.8074	9.4203	2.361037775	3.132787334	3.138234868	-0.451350686	0.320398873	0.325846407
RNA-Seq	Trans-ABySS	XLOC_009181	1007	XM_008275810.1	1074			nicotinamide riboside kinase 2	itgb1bp3, nmrk2, mibp	molecular_function	GO:0030550	ribosylnicotinamide kinase activity	Protein Modification related	53.3913	32.6702	25.1647	25.1463	203.642	-0.70862939	-1.085203278	-1.086258538	-2.639987948	-3.016561836	-3.017617096
WGS	ABySS	XLOC_018768	1090	XM_008275810.1	1074	882/1034	0	nicotinamide riboside kinase 2	itgb1bp3, nmrk2, mibp	molecular_function	GO:0050262	ribosylnicotinamide kinase activity	Protein Modification related	86.2251	50.4086	46.0453	47.0226	285.75	-0.77443801	-0.905053994	-0.874753585	-2.503011706	-2.63362769	-2.603327282
RNA-Seq	Trans-ABySS	XLOC_013161	2205	XM_008277553.1	2335	1726/2066	0	kelch-like family member 30	klhl30	biological_process	GO:0016567	protein ubiquitination	Protein Modification related	10.6447	20.3962	65.6749	64.4947	7.60052	0.938165099	2.625206808	2.599045318	1.424130359	3.111172068	3.085010578
WGS	ABySS	XLOC_058827	3777	XM_008277553.1	2335	1726/2066	0	kelch-like family member 30	klhl30	biological_process	GO:0016567	protein ubiquitination	Protein Modification related	7.10613	13.6752	38.683	40.9406	6.15685	0.944425946	2.444563698	2.526396259	1.151297607	2.65143536	2.73326792
RNA-Seq	Trans-ABySS	XLOC_014433	1922	XM_008276980.1	2937	1544/1861	0	kelch-like family member 38	klhl38, c8orfk36	biological_process	GO:0016567	protein ubiquitination	Protein Modification related	0.610613	6.60866	38.714	36.9257	4.07784	3.436027564	5.986453262	5.918223155	0.696552604	3.246978302	3.178748195
WGS	ABySS	XLOC_019696	3010	XM_008276980.1	2937	1592/1923	0	kelch-like family member 38	klhl38, c8orfk36	biological_process	GO:0016567	protein ubiquitination	Protein Modification related	1.01853	6.91955	46.6824	47.1531	5.02631	2.764189744	5.518318354	5.532792242	0.461178565	3.215307176	3.229781064
RNA-Seq	Trinity	XLOC_077223	2458	XM_008276980.1	2937	1592/1923	0	kelch-like family member 38	klhl38, c8orfk36	biological_process	GO:0016567	protein ubiquitination	Protein Modification related	0.315552	3.27429	19.1295	18.6827	1.69748	3.375232433	5.921777588	5.887681389	0.947787529	3.494332684	3.460236485
RNA-Seq	Trans-ABySS	XLOC_171194	413	XM_008276980.1	2937	130/166	4.00E-29	kelch-like family member 38	klhl38, c8orfk36	biological_process	GO:0016567	protein ubiquitination	Protein Modification related	1.52733	8.51663	61.6713	60.2458	5.93917	2.479270865	5.335515544	5.301776954	0.520021346	3.376266025	3.342527435
WGS	ABySS	XLOC_005989	771	XM_008290401.1	3958	545/626	0	E3 ubiquitin-protein ligase makorin-1	mkrn1, rnf61	biological_process	GO:0000209	protein polyubiquitination	Protein Modification related	43.6234	101.384	259.018	264.872	23.4769	1.216655867	2.569878236	2.60212122	2.110516164	3.463738533	3.495981516
WGS	ABySS	XLOC_032284	2714	XM_011486942.1	3189	803/950	0	myosin light chain kinase family member 4-like	mylk4	biological_process	GO:0006468	protein phosphorylation	Protein Modification related	2.48326	2.37424	1.6635	1.20363	16.6589	-0.064769544	-0.578013455	-1.04484335	-2.810755459	-3.32399937	-3.790829266
RNA-Seq	Trinity	XLOC_005833	1195	XM_005810668.1	3997	431/523	2.00E-145	papilin-like	papln	biological_process	GO:0007605	serine-type endopeptidase activity	Protein Modification related	9.44982	6.57638	6.0905	5.85188	36.7183	-0.522993185	-0.633726178	-0.691386664	-2.481133697	-2.59186669	-2.649527175
RNA-Seq	Trans-ABySS	XLOC_014979	1144	XM_007547866.1	2394	884/1041	0	protein disulfide isomerase family A, member 4	pdia4, erp70, erp72, erp-72	biological_process	GO:0009607	protein disulfide isomerase activity	Protein Modification related	19.1659	10.6132	13.9992	14.76	86.9342	-0.852682036	-0.453197361	-0.376849025	-3.034064136	-2.634579461	-2.558231125
RNA-Seq	Trinity	XLOC_074663	1239	XM_007547866.1	2394	886/1044	0	protein disulfide isomerase family A, member 4	pdia4, erp70, erp72, erp-72	molecular_function	GO:0003756	protein disulfide isomerase activity	Protein Modification related	15.5765	8.91329	11.7394	12.4513	73.8959	-0.805341149	-0.408012426	-0.323074723	-3.05146437	-2.654135647	-2.569197944
WGS	ABySS	XLOC_011174	1058	XM_008303114.1	2586	926/1058	0	serine/threonine-protein phosphatase 5	ppp5c	molecular_function	GO:0044822	protein dephosphorylation	Protein Modification related	5.89527	5.29236	5.19804	4.58051	29.8522	-0.155646689	-0.181590154	-0.364049651	-2.495854151	-2.521797616	-2.704257113
RNA-Seq	Trinity	XLOC_093931	379	XM_006805212.1	2549	78/90	2.00E-19	serine/threonine-protein phosphatase 5	ppp5c	biological_process	GO:0006470	protein dephosphorylation	Protein Modification related	19.8462	18.2277	19.3304	19.0374	120.594	-0.122730266	-0.037991306	-0.060026339	-2.725953693	-2.641214733	-2.663249766
RNA-Seq	Trans-ABySS	XLOC_150350	827	XM_008303934.1	989	691/840	0	trypsin-2-like	prss2, try2	biological_process	GO:0046530	serine-type endopeptidase activity	Protein Modification related	2.63554	43.8345	24.9888	25.8333	17.7215	4.055896299	3.245111129	3.293061455	1.306566069	0.4957809	0.543731225
WGS	ABySS	XLOC_045404	1749	XM_005748280.1	1630	297/343	1.00E-110	tyrosine aminotransferase-like	tat	molecular_function	GO:0005200	L-tyrosine:2-oxoglutarate aminotransferase activity	Protein Modification related	5.57477	30.5133	45.9012	43.7258	18.5961	2.452454026	3.041547681	2.97150059	0.714438126	1.303531782	1.233484691
RNA-Seq	Trinity	XLOC_055125	2052	XM_005748280.1	1630	1242/1468	0	tyrosine aminotransferase-like	tat	molecular_function	GO:0004838	L-tyrosine:2-oxoglutarate aminotransferase activity	Protein Modification related	6.30771	33.7849	47.0273	46.8257	20.2081	2.421190347	2.898310266	2.892112323	0.741444901	1.218564821	1.212366878
RNA-Seq	Trinity	XLOC_008548	1060	XM_008301506.1	2453	725/999	3.00E-144	protein-glutamine gamma-glutamyltransferase 2-like	tgm2, tgc, gnah	molecular_function	GO:0016740	peptide cross-linking	Protein Modification related	17.0334	8.16977	1.52081	1.37266	11.8727	-1.059999069	-3.485454608	-3.633320209	-0.539280691	-2.964736231	-3.112601832
WGS	ABySS	XLOC_060866	2768	XM_008301506.1	2453	1546/2087	0	protein-glutamine gamma-glutamyltransferase 2-like	tgm2, tgc, gnah	biological_process	GO:0018149	peptide cross-linking	Protein Modification related	9.76245	4.99075	0.882727	0.730608	6.90682	-0.967986617	-3.467204023	-3.740073798	-0.468764989	-2.967982395	-3.24085217
WGS	ABySS	XLOC_040168	3257	XM_007559692.1	2581	419/545	3.00E-106	protein-glutamine gamma-glutamyltransferase E-like	tgm3, tge	biological_process	GO:0018149	peptide cross-linking	Protein Modification related	30.5112	13.8392	11.5818	11.4946	82.1932	-1.140578374	-1.397479433	-1.408382659	-2.570258494	-2.827159553	-2.838062779
RNA-Seq	Trans-ABySS	XLOC_003313	116	XM_007545808.1	1087	107/115	6.00E-39	ubiquitin B	ubb	molecular_function	GO:0004838	ubiquitin homeostasis	Protein Modification related	5905.65	5279.29	6251.85	5466.07	971.782	-0.16175194	0.082187307	-0.111591922	2.441639303	2.685578549	2.49179932
RNA-Seq	Trinity	XLOC_070839	958	XM_008278434.1	4720	199/278	5.00E-27	ubiquitin-like modifier activating enzyme 6	ube1l2, uba6	biological_process	GO:0006464	ubiquitin-dependent protein catabolic process	Protein Modification related	3.9304	3.11796	4.13323	3.66967	20.139	-0.334073724	0.072593503	-0.099025812	-2.691317723	-2.284650496	-2.456269811
RNA-Seq	Trinity	XLOC_087090	602	XM_008404684.1	818	103/122	6.00E-28	ubiquitin-like 5	ubl5, hub1	biological_process	GO:0010992	cellular protein modification process	Protein Modification related	3.87188	3.36463	2.87436	3.71407	66.0903	-0.202586376	-0.429793476	-0.060033234	-4.295918775	-4.523125876	-4.153365633
RNA-Seq	Trans-ABySS	XLOC_115118	512	XM_008404684.1	818	103/122	6.00E-28	ubiquitin-like 5	ubl5, hub1	biological_process	GO:0006464	cellular protein modification process	Protein Modification related	4.88663	3.70794	2.89653	4.71244	67.2275	-0.398221973	-0.754514265	-0.052365623	-4.180361697	-4.536653988	-3.834505346
WGS	ABySS	XLOC_044125	1984	XM_008279403.1	4000	906/1064	0	ubiquitin-like with PHD and ring finger domains 1	uhrf1, icbp90, np95	biological_process	GO:0006511	ubiquitin-protein transferase activity	Protein Modification related	6.68226	3.27888	1.90376	2.28727	17.7879	-1.027133014	-1.811484503	-1.546709439	-2.439621191	-3.223972679	-2.959197616
RNA-Seq	Trinity	XLOC_005593	1680	XM_005811264.1	2001	1046/1240	0	arginase-2, mitochondrial-like	arg2	cellular_component	GO:0005739	mitochondrion	Respiration related	7.60296	32.7631	65.7218	65.5607	40.3684	2.107438763	3.111738888	3.108198152	-0.301154538	0.703145587	0.699604852
RNA-Seq	Trans-ABySS	XLOC_010554	1661	XM_005811264.1	2001	1046/1240	0	arginase-2, mitochondrial-like	arg2	cellular_component	GO:0005739	mitochondrion	Respiration related	8.3895	36.6339	73.8465	73.075	43.64	2.126522561	3.137872809	3.122721188	-0.252471805	0.758878444	0.743726822
WGS	ABySS	XLOC_017417	3044	XM_005811264.1	2001	1046/1240	0	arginase-2, mitochondrial-like	arg2	cellular_component	GO:0005739	mitochondrion	Respiration related	6.86922	30.0114	65.1922	63.8586	48.5947	2.127292425	3.246481166	3.216662729	-0.695288354	0.423900387	0.39408195
RNA-Seq	Trinity	XLOC_088359	1252	XM_003445474.2	1974	78/99	5.00E-12	arginase-2, mitochondrial-like	arg2	cellular_component	GO:0005739	mitochondrion	Respiration related	7.38999	32.7973	74.9831	71.322	70.165	2.149932734	3.342921154	3.270702842	-1.097174507	0.095813913	0.023595601
RNA-Seq	Trans-ABySS	XLOC_106102	1150	XM_003445474.2	1974	78/99	5.00E-12	arginase-2, mitochondrial-like	arg2	cellular_component	GO:0005739	mitochondrion	Respiration related	8.87127	39.6269	90.8659	87.0504	84.0861	2.159267551	3.356526425	3.294638368	-1.085387223	0.111871651	0.049983594
RNA-Seq	Trans-ABySS	XLOC_159568	194	XM_005796607.1	3228	188/194	2.00E-84	sodium/potassium-transporting ATPase subunit alpha-2-like	atp1a2	biological_process	GO:0006470	sodium:potassium-exchanging ATPase activity	Respiration related	199.269	96.0588	47.8368	66.1275	514.237	-1.052727598	-2.058524499	-1.591395024	-2.420443726	-3.426240628	-2.959111152
RNA-Seq	Trinity	XLOC_008599	884	XM_008398669.1	2499	312/379	8.00E-102	egl-9 family hypoxia-inducible factor 3	egln3, phd3, hifp4h3	molecular_function	GO:0016491	oxidoreductase activity	Respiration related	45.673	20.3497	5.4532	5.90843	4.80604	-1.166334029	-3.066166582	-2.950494825	2.082086965	0.182254412	0.29792617
RNA-Seq	Trans-ABySS	XLOC_009969	1424	XM_008398669.1	2499	628/757	0	egl-9 family hypoxia-inducible factor 3	egln3, phd3, hifp4h3	molecular_function	GO:0016491	oxidoreductase activity	Respiration related	50.4327	21.2007	6.58149	6.15863	4.83377	-1.250247564	-2.937873323	-3.033678103	2.132891166	0.445265408	0.349460628
WGS	ABySS	XLOC_052912	716	XM_008398669.1	2499	304/364	5.00E-103	egl-9 family hypoxia-inducible factor 3	egln3, phd3, hifp4h3	molecular_function	GO:0016491	oxidoreductase activity	Respiration related	36.9064	14.8392	4.14529	4.25837	2.08094	-1.314457702	-3.154326078	-3.115497805	2.834106043	0.994237666	1.033065939
RNA-Seq	Trinity	XLOC_072793	476	XM_008398669.1	2499	323/386	4.00E-111	egl-9 family hypoxia-inducible factor 3	egln3, phd3, hifp4h3	molecular_function	GO:0016491	oxidoreductase activity	Respiration related	73.2142	29.2108	8.41161	7.80336	3.95608	-1.325621618	-3.121669622	-3.229956125	2.884358364	1.088310361	0.980023858
RNA-Seq	Trans-ABySS	XLOC_013457	305	XM_007575907.1	990	246/298	3.00E-78	GTPase IMAP family member 4-like	gimap4, imap4, ian1	molecular_function	GO:0003924	GTPase activity	Respiration related	17.0454	24.7852	8.12172	8.18171	120.244	0.540096445	-1.06952526	-1.058908148	-2.278414102	-3.888035807	-3.877418695
WGS	ABySS	XLOC_035576	1329	XM_005465863.1	2446	395/545	8.00E-75	GTPase IMAP family member 4-like	gimap4, imap4, ian1	molecular_function	GO:0003924	GTPase activity	Respiration related	2.99411	4.28757	2.37358	2.66868	18.2355	0.518033	-0.33506255	-0.166000902	-2.088517627	-2.941613177	-2.772551529
RNA-Seq	Trans-ABySS	XLOC_009115	554	XM_011477879.1	519	378/438	1.00E-143	hemoglobin embryonic subunit alpha-like	hba1	biological_process	GO:0015671	oxygen transport	Respiration related	129.008	16.8909	10.8331	9.87954	144.798	-2.933142426	-3.573942484	-3.706872852	-3.099723569	-3.740523627	-3.873453995
RNA-Seq	Trinity	XLOC_022258	604	XM_011477879.1	519	378/438	1.00E-143	hemoglobin embryonic subunit alpha-like	hba1	biological_process	GO:0015671	oxygen transport	Respiration related	116.267	16.3671	10.8482	9.57041	128.232	-2.82857105	-3.421914089	-3.602717134	-2.969885702	-3.563228741	-3.744031786
RNA-Seq	Trans-ABySS	XLOC_007003	357	XM_008436239.1	576	141/174	5.00E-35	hemoglobin subunit alpha-1-like	hba1	biological_process	GO:0015671	oxygen transport	Respiration related	642.213	106.053	40.6539	51.69	153.268	-2.598266437	-3.981586205	-3.635094762	-0.531271084	-1.914590852	-1.568099409
RNA-Seq	Trinity	XLOC_055436	779	XM_008436239.1	576	399/481	3.00E-132	hemoglobin subunit alpha-1-like	hba1	biological_process	GO:0015671	oxygen transport	Respiration related	3307.79	622.032	208.769	229.198	333.672	-2.41080694	-3.985888238	-3.851201286	0.898558172	-0.676523125	-0.541836173
RNA-Seq	Trinity	XLOC_041494	602	XM_005809039.1	552	383/459	2.00E-133	hemoglobin subunit beta-like	hbb	biological_process	GO:0015671	oxygen transport	Respiration related	215.138	26.0946	15.7594	14.6658	29.5404	-3.043439179	-3.770977858	-3.874734696	-0.178938074	-0.906476753	-1.010233591
RNA-Seq	Trans-ABySS	XLOC_109244	545	XM_005809039.1	552	383/459	2.00E-133	hemoglobin subunit beta-like	hbb	biological_process	GO:0015671	oxygen transport	Respiration related	289.435	34.3778	20.6682	19.2791	38.3575	-3.073690263	-3.807754736	-3.908129781	-0.158031471	-0.892095944	-0.99247099
WGS	ABySS	XLOC_020585	702	XM_005809039.1	552	383/459	3.00E-133	hemoglobin subunit beta-like	hbb	biological_process	GO:0015671	oxygen transport	Respiration related	178.09	20.8314	13.807	12.5101	26.866	-3.095774804	-3.689134721	-3.831441282	-0.367021738	-0.960381655	-1.102688217
RNA-Seq	Trans-ABySS	XLOC_012373	452	XM_008302151.1	1414	357/450	4.00E-103	interferon-inducible GTPase 5-like	irgm, irgm1, lrg47, ifi1	molecular_function	GO:0005525	GTP binding	Respiration related	6.43663	17.2009	10.4321	9.46726	145.171	1.418106608	0.696652159	0.556641403	-3.077197325	-3.798651774	-3.938662529
RNA-Seq	Trinity	XLOC_090628	453	XM_008302151.1	1414	357/450	4.00E-103	interferon-inducible GTPase 5-like	irgm, irgm1, lrg47, ifi1	molecular_function	GO:0005525	GTP binding	Respiration related	5.55393	15.2971	9.18855	8.3719	129.965	1.461677276	0.726328221	0.592046085	-3.086793073	-3.822142128	-3.956424264
RNA-Seq	Trans-ABySS	XLOC_015244	293	XM_006800610.1	1204	170/240	1.00E-22	interferon-inducible GTPase 5-like	irgm, irgm1, lrg47, ifi1	molecular_function	GO:0005525	GTP binding	Respiration related	12.1758	21.5212	21.9113	14.1316	178.025	0.821741957	0.847658516	0.214908253	-3.048249424	-3.022332865	-3.655083129
RNA-Seq	Trans-ABySS	XLOC_016925	347	XM_006800610.1	1204	164/199	2.00E-45	interferon-inducible GTPase 5-like	irgm, irgm1, lrg47, ifi1	molecular_function	GO:0005525	GTP binding	Respiration related	8.24137	14.0067	9.61035	11.2741	101.997	0.765161006	0.221704791	0.452056181	-2.86433772	-3.407793936	-3.177442545
RNA-Seq	Trinity	XLOC_050107	592	XM_006800610.1	1204	298/427	7.00E-42	interferon-inducible GTPase 5-like	irgm, irgm1, lrg47, ifi1	molecular_function	GO:0005525	GTP binding	Respiration related	5.09188	9.74047	7.72695	5.95891	70.9778	0.935792968	0.601700643	0.226850038	-2.865304565	-3.19939689	-3.574247495
WGS	ABySS	XLOC_058124	1625	XM_005755462.1	1950	291/403	1.00E-52	interferon-inducible GTPase 5-like	irgm, irgm1, lrg47, ifi1	molecular_function	GO:0005525	GTP binding	Respiration related	0.352084	1.52482	0.869011	1.01424	13.7105	2.114647375	1.303454772	1.526407506	-3.168570332	-3.979762936	-3.756810202
RNA-Seq	Trinity	XLOC_090196	355	XM_006800610.1	1204	164/199	4.00E-45	interferon-inducible GTPase 5-like	irgm, irgm1, lrg47, ifi1	molecular_function	GO:0005525	GTP binding	Respiration related	6.77389	11.7729	8.32341	9.4968	89.0843	0.797413276	0.297190145	0.487456912	-2.919701456	-3.419924588	-3.229657821
RNA-Seq	Trinity	XLOC_005264	2648	XM_005795089.1	2691	1700/2171	0	myeloperoxidase-like	mpo	molecular_function	GO:0004601	peroxidase activity	Respiration related	0.194014	1.14458	0.38739	0.418739	72.1444	2.560585637	0.997625952	1.109890532	-5.977997206	-7.540956891	-7.428692312
WGS	ABySS	XLOC_001085	92	AF283503.1	17329	92/92	2.00E-38	ATPase 8	mt-atp8, atp8	biological_process	GO:0022904	respiratory electron transport chain	Respiration related	11889.5	13505.4	23781	26648.6	116635	0.183848325	1.000121337	1.164371697	-3.110392505	-2.294119492	-2.129869132
WGS	ABySS	XLOC_065131	106	GQ389376.1	827	106/106	6.00E-45	cytochrome c oxidase subunit III	mtco3	molecular_function	GO:0004129	cytochrome-c oxidase activity	Respiration related	8790.63	9492.26	9795.58	10414.2	58535.5	0.110785055	0.156164355	0.24451355	-2.624488318	-2.579109018	-2.490759822
WGS	ABySS	XLOC_006227	2050	XM_008289840.1	1668	1300/1599	0	bifunctional methylenetetrahydrofolate dehydrogenase/cyclohydrolase	mthfd2	molecular_function	GO:0004488	methylenetetrahydrofolate dehydrogenase (NADP+) activity	Respiration related	14.7721	8.212	7.45265	7.84564	49.2746	-0.847069401	-0.987049521	-0.912911891	-2.585038627	-2.725018747	-2.650881116
RNA-Seq	Trans-ABySS	XLOC_108561	175	XM_008289840.1	1668	104/136	2.00E-13	bifunctional methylenetetrahydrofolate dehydrogenase/cyclohydrolase	mthfd2	molecular_function	GO:0004488	methylenetetrahydrofolate dehydrogenase (NADP+) activity	Respiration related	194.57	110.377	74.575	123.051	935.533	-0.817849704	-1.383525306	-0.661032901	-3.083348965	-3.649024566	-2.926532162
RNA-Seq	Trinity	XLOC_061719	1479	XM_008418588.1	1606	1115/1368	0	methenyltetrahydrofolate cyclohydrolase	mthfd2	molecular_function	GO:0004488	methylenetetrahydrofolate dehydrogenase (NADP+) activity	Respiration related	16.2704	8.9627	7.5804	8.14553	52.9216	-0.860244407	-1.101903836	-0.998169242	-2.561851367	-2.803510797	-2.699776202
RNA-Seq	Trans-ABySS	XLOC_074735	540	XM_007572116.1	1708	437/491	1.00E-180	methenyltetrahydrofolate cyclohydrolase	mthfd2	molecular_function	GO:0004488	methylenetetrahydrofolate dehydrogenase (NADP+) activity	Respiration related	29.5914	16.5949	13.9396	13.3404	106.492	-0.834438018	-1.08598879	-1.149376028	-2.681933214	-2.933483986	-2.996871225
WGS	ABySS	XLOC_002335	114	AY946275.1	1624	114/114	3.00E-50	NADH dehydrogenase subunit 2	mt-nd2	molecular_function	GO:0003954	NADH dehydrogenase activity	Respiration related	4931.08	7228.48	3807.07	4505.63	26502.6	0.551788651	-0.373222562	-0.130174814	-1.874369685	-2.799380898	-2.55633315
WGS	ABySS	XLOC_003881	83	AY946275.1	1624	83/83	2.00E-33	NADH dehydrogenase subunit 2	mt-nd2	molecular_function	GO:0003954	NADH dehydrogenase activity	Respiration related	11593.7	15289.1	14998.4	16152.1	95492.2	0.399162424	0.371467545	0.478380688	-2.642879411	-2.670574291	-2.563661148
WGS	ABySS	XLOC_004963	119	AF283503.1	17329	117/119	3.00E-50	NADH dehydrogenase subunit 4	mt-nd2	molecular_function	GO:0003954	NADH dehydrogenase activity	Respiration related	3821.8	5216.76	5002.78	5729.83	39395.8	0.448901781	0.388477728	0.584240054	-2.916815864	-2.977239916	-2.781477591
RNA-Seq	Trans-ABySS	XLOC_021855	672	XM_008420668.1	2323	570/693	0	NADPH oxidase 1	nox1, noh1, mox1	molecular_function	GO:0003954	superoxide anion generation	Respiration related	5.94479	7.17737	6.10012	7.11496	39.0336	0.271829447	0.037211776	0.259229798	-2.443189326	-2.677806998	-2.455788975
RNA-Seq	Trinity	XLOC_045985	1200	XM_007569664.1	2330	1000/1184	0	NADPH oxidase 1	nox1, noh1, mox1	biological_process	GO:0042554	superoxide anion generation	Respiration related	4.24496	4.54152	4.23799	4.87111	23.2489	0.097424274	-0.002370776	0.198499603	-2.355915319	-2.455710369	-2.25483999
RNA-Seq	Trinity	XLOC_036519	720	XM_008438131.1	780	446/546	2.00E-146	putative protein PTGES3L	ptges3, p23, cpges	cellular_component	GO:0016023	ATP binding	Respiration related	11.1036	5.53647	6.42184	5.94987	39.0824	-1.003989176	-0.789968876	-0.90009745	-2.819480738	-2.605460438	-2.715589012
WGS	ABySS	XLOC_057279	2585	XM_008291990.1	2431	1340/1683	0	thromboxane A synthase 1 (platelet)	tbxas1, cyp5	biological_process	GO:0030198	monooxygenase activity	Respiration related	62.0056	42.2585	59.7818	61.3553	10.9096	-0.553156959	-0.052692181	-0.015210545	1.953643352	2.45410813	2.491589766
RNA-Seq	Trinity	XLOC_005595	1937	XM_008291610.1	1546	819/928	0	mitochondrial uncoupling protein 2-like	ucp2	biological_process	GO:0001666	response to hypoxia	Respiration related	12.6773	404.984	539.54	539.005	70.4887	4.997545492	5.411410502	5.409979237	2.522401008	2.936266017	2.934834752
WGS	ABySS	XLOC_056583	3868	XM_008291610.1	1546	839/957	0	mitochondrial uncoupling protein 2-like	ucp2	biological_process	GO:0001666	response to hypoxia	Respiration related	4.77073	185.395	242.557	253.376	30.8912	5.280248486	5.667969966	5.730926028	2.585334516	2.973055995	3.036012057
RNA-Seq	Trans-ABySS	XLOC_130700	184	XM_007575878.1	793	52/67	0.002	neurotoxin 77-like	lntx-77	cellular_component	GO:0005575	acetylcholine receptor inhibitor activity	Toxin-Like related	39.4155	80.6347	49.2576	79.9336	1100.57	1.03263774	0.321583261	1.02003899	-3.770706283	-4.481760762	-3.783305032
RNA-Seq	Trinity	XLOC_058640	442	XM_011492215.1	677	88/114	5.00E-14	long neurotoxin homolog Pa ID-like	lntx-id	molecular_function	GO:0030550	acetylcholine receptor inhibitor activity	Toxin-Like related	136.466	229.059	141.215	132.815	813.482	0.747177695	0.049351788	-0.039123461	-1.828391174	-2.526217082	-2.614692331
WGS	ABySS	XLOC_012336	2078	XM_004565298.1	2091	1477/2061	0	neoverrucotoxin subunit alpha-like	neovtx	biological_process	GO:0009405	cellular_component	Toxin-Like related	6.48248	3.36102	1.99358	1.66667	55.3423	-0.947646722	-1.70118435	-1.95957737	-4.04141157	-4.794949198	-5.053342218
RNA-Seq	Trinity	XLOC_069661	2317	XM_004565298.1	2091	1378/1934	0	neoverrucotoxin subunit alpha-like	neovtx	cellular_component	GO:0005575	cellular_component	Toxin-Like related	10.0608	5.14059	2.87514	2.45755	68.0731	-0.968739172	-1.807040915	-2.033452354	-3.727078954	-4.565380698	-4.791792136
RNA-Seq	Trans-ABySS	XLOC_077346	655	XM_004565298.1	2091	419/522	4.00E-130	neoverrucotoxin subunit alpha-like	neovtx	cellular_component	GO:0005575	cellular_component	Toxin-Like related	13.0456	5.58593	3.95628	2.93433	92.2174	-1.223693899	-1.721346859	-2.152460266	-4.045169591	-4.542822551	-4.973935957
RNA-Seq	Trans-ABySS	XLOC_078235	802	XM_004565298.1	2091	477/700	2.00E-57	neoverrucotoxin subunit alpha-like	neovtx	cellular_component	GO:0005575	cellular_component	Toxin-Like related	14.7964	7.41458	3.58053	2.71274	89.1236	-0.996809329	-2.047001147	-2.447423522	-3.587370631	-4.637562449	-5.037984824
RNA-Seq	Trans-ABySS	XLOC_010843	755	XM_008416470.1	1963	319/449	4.00E-52	cytolysin RTX-S-2-like	rtx-s II	biological_process	GO:0019835	cytolysis	Toxin-Like related	58.4397	24.0801	28.0111	28.2792	326.228	-1.279107389	-1.060950133	-1.047207464	-3.759967325	-3.541810069	-3.5280674
WGS	ABySS	XLOC_025316	1743	XM_008416470.1	1963	319/449	9.00E-52	cytolysin RTX-S-2-like	rtx-s II	biological_process	GO:0019835	cytolysis	Toxin-Like related	25.4459	11.373	12.4702	13.7836	175.068	-1.161820357	-1.028948615	-0.884480479	-3.944230636	-3.811358894	-3.666890758
RNA-Seq	Trinity	XLOC_033129	819	XM_008416470.1	1963	319/449	4.00E-52	cytolysin RTX-S-2-like	rtx-s II	biological_process	GO:0019835	cytolysis	Toxin-Like related	46.7737	19.5488	22.8994	23.3581	268.162	-1.258617506	-1.030387759	-1.00177463	-3.777952858	-3.549723111	-3.521109982
WGS	ABySS	XLOC_025786	1741	XM_008292372.1	3344	382/553	2.00E-48	stonustoxin subunit beta-like	sntx	biological_process	GO:0007286	cellular_component	Toxin-Like related	3.23008	1.44636	1.00428	1.00982	20.8952	-1.159143212	-1.685408338	-1.677471741	-3.852672978	-4.378938104	-4.371001506
RNA-Seq	Trans-ABySS	XLOC_162104	847	XM_008292372.1	3344	314/458	1.00E-36	stonustoxin subunit beta-like	sntx	cellular_component	GO:0005575	cellular_component	Toxin-Like related	14.4702	7.5748	4.16244	4.31548	92.0578	-0.93380516	-1.797583481	-1.745491921	-3.603260263	-4.467038584	-4.414947025
WGS	ABySS	XLOC_048034	587	XM_008298326.1	607	421/521	4.00E-129	gastrotropin-like	fabp6, ileal, ilbp, ibabp	molecular_function	GO:0005215	transporter activity	Transporter related	17.8246	7.13111	5.98029	6.23037	113.295	-1.321671138	-1.57558235	-1.516479954	-3.989813724	-4.243724936	-4.18462254
RNA-Seq	Trinity	XLOC_023851	1761	XM_008415460.1	1857	1442/1721	0	importin subunit alpha-1-like	kpna2, qip2, srp1-a, rch1	molecular_function	GO:0008565	protein transporter activity	Transporter related	10.6589	3.61237	1.84957	1.63104	13.3619	-1.561040985	-2.526796752	-2.708194491	-1.887107592	-2.852863359	-3.034261098
WGS	ABySS	XLOC_009251	2686	XM_007565384.1	2873	1661/2049	0	solute carrier family 15 member 1-like	slc15a1	molecular_function	GO:0005391	transporter activity	Transporter related	19.1086	105.262	101.91	100.339	26.6663	2.461690724	2.415001636	2.392588477	1.980895145	1.934206057	1.911792898
RNA-Seq	Trinity	XLOC_076666	1585	XM_005470012.1	2005	925/1354	2.00E-135	excitatory amino acid transporter 3-like	slc1a1, dcbxa, eaac1, eaat3, sczd18	molecular_function	GO:0005313	L-glutamate transmembrane transporter activity	Transporter related	0.314738	0.732662	10.7407	12.1862	0.865794	1.218996418	5.092792837	5.27495314	-0.240876011	3.632920408	3.81508071
RNA-Seq	Trans-ABySS	XLOC_013132	659	XM_007572872.1	2928	122/143	5.00E-36	antigen peptide transporter 2-like	tap2, mhc2, abcb3, ring11, psf2, apt2	biological_process	GO:0055085	transmembrane transport	Transporter related	3.70888	10.0401	26.078	24.6756	2.53366	1.436718143	2.813777734	2.734029669	1.986478796	3.363538387	3.283790322
RNA-Seq	Trans-ABySS	XLOC_011912	901	XM_008287783.1	621	486/599	4.00E-155	transmembrane 4 L6 family member 5-like	tm4sf1, taal6, m3s1	cellular_component	GO:0048471	integral component of plasma membrane	Transporter related	4.50355	14.7566	12.5416	13.9235	116.309	1.712225771	1.477586828	1.628387328	-2.978530477	-3.21316942	-3.06236892
RNA-Seq	Trinity	XLOC_022249	992	XM_008287783.1	621	486/599	9.00E-155	transmembrane 4 L6 family member 5-like	tm4sf1, taal6, m3s1	cellular_component	GO:0005887	integral component of plasma membrane	Transporter related	3.84243	12.7224	11.5641	12.5039	108.673	1.727279969	1.589562107	1.702287263	-3.094550788	-3.23226865	-3.119543494
WGS	ABySS	XLOC_044345	1544	XM_008287783.1	621	486/599	1.00E-154	transmembrane 4 L6 family member 5-like	tm4sf1, taal6, m3s1	cellular_component	GO:0005887	integral component of plasma membrane	Transporter related	2.8856	8.61923	8.27042	9.71075	70.2346	1.578687663	1.519089268	1.750711397	-3.02655103	-3.086149426	-2.854527297

Supplementary Table S3. Total number of Kmar embryos used for RNA extraction

RNA Sample	Embryonic Stage	Diapause?	Total Embryos
RNA01-preDia	30-31	No	96
RNA02-Dia-R1	32	Yes	85
RNA03-Dia-R2	32	Yes	91
RNA04-Dia-R3	32	Yes	92
RNA05-postDia	Larvae	No	20

Supplementary Table S4. Numbers of filtered RNA-Seq reads for downstream analyses.

RNA Sample	Total Reads	Adapters (%)	N[1] (%)	Low Quality[2] (%)	Total Filtered Reads
RNA01-preDia	32,669,979	0.09%	0.01%	0.29%	32,539,312
RNA02-Dia-R1	40,359,918	0.05%	0.01%	0.23%	40,231,019
RNA03-Dia-R2	32,492,341	0.06%	0.01%	0.25%	32,380,877
RNA04-Dia-R3	33,842,811	0.10%	0.01%	0.25%	33,714,161
RNA05-postDia	32,041,323	0.09%	0.01%	0.23%	31,906,229

[1]>5% of unknown bases;

[2]>50% of bases with Phred score ≤10

Supplementary Table S5. NGS data used in this study and deposited at the NCBI SRA database.

NGS	Sample	No of Reads	NCBI SRA ID[2]	Total Length (nt)	Genome Coverage[3]
RNA-Seq	01. Pre-Diapause, Pooled RNA, 96 Embryos	32,539,312	SRR1999414	1,626,965,600	2.53
RNA-Seq	02. Diapause, Replicate #1, Pooled RNA, 85 Embryos	40,231,019	SRR2001218	2,011,550,950	3.13
RNA-Seq	03. Diapause, Replicate #2, Pooled RNA, 91 Embryos	32,380,877	SRR2001221	1,619,043,850	2.52
RNA-Seq	04. Diapause, Replicate #3, Pooled RNA, 92 Embryos	33,714,161	SRR2001227	1,685,708,050	2.62
RNA-Seq	05. Post-Diapause, Pooled RNA, 20 Larvae	31,906,229	SRR2001231	1,595,311,450	2.48
WGS	Kmar FDS08 (Km04), gDNA, sense strand	219,138,546	SRR2079677	21,913,854,600	34.12
WGS	Kmar FDS08 (Km04), gDNA, anti-sense strand	219,138,546	SRR2080613	21,913,854,600	34.12
RADseq[1]	Km01 FDS04 DNA	970,981	SRR1585247	145,647,150	0.23
RADseq	Km02 FDS05 gDNA	885,028	SRR1585250	132,754,200	0.21
RADseq	Km03 ERIN14 gDNA	559,997	SRR1585251	83,999,550	0.13
RADseq	Km04 FDS08 gDNA	656,403	SRR1585252	98,460,450	0.15
RADseq	Km05 EPP02 gDNA	983,923	SRR1585253	147,588,450	0.23
RADseq	Km07 ERIN15 gDNA	728,613	SRR1585254	109,291,950	0.17
RADseq	Km08 ERIN18 gDNA	932,971	SRR1585255	139,945,650	0.22
RADseq	Km09 FDS07 gDNA	1,079,868	SRR1585256	161,980,200	0.25
RADseq	Km10 PC13 gDNA	1,017,990	SRR1585257	152,698,500	0.24
RADseq	Km11 EPP01 gDNA	1,162,674	SRR1585258	174,401,100	0.27
RADseq	Km12 EPP07 gDNA	1,330,378	SRR1585259	199,556,700	0.31
RADseq	Km13 FDS11 gDNA	1,059,856	SRR1585260	158,978,400	0.25
RADseq	Km14 ERIN12 gDNA	867,305	SRR1585261	130,095,750	0.20
RADseq	Km15 FDS09 gDNA	1,587,698	SRR1585262	238,154,700	0.37
RADseq	Km16 PC12 gDNA	1,062,268	SRR1585263	159,340,200	0.25
RADseq	Km17 FDS02 gDNA	1,352,690	SRR1585264	202,903,500	0.32
RADseq	Km18 EPP04 gDNA	1,223,542	SRR1585266	183,531,300	0.29
RADseq	Km19 FDS10 gDNA	1,167,249	SRR1585268	175,087,350	0.27

[1]Reference: Mesak F *et al* . 2014;

[2]http://www.ncbi.nlm.nih.gov/sra/;

[3]Kmar genome size: 642,279,823 bp.
