# Supplementary material for: Transcriptomics of diapause in an isogenic self-fertilizing vertebrate
Source: BMC Genomics. 2015 Nov 23;16:989. doi: 10.1186/s12864-015-2210-0 (PMC4657215; doi:10.1186/s12864-015-2210-0)

**Supplementary Fig. S1.**

**A.**

| RNA Sample | NanoDrop 8000 Total Mass (µg) | $OD_{260/280}$ | BGI - Agilent2100 Total Mass (µg) | RIN | 28S/18S |
|---|---|---|---|---|---|
| 01 | 213.8 | 2.13 | 163.9 | 9.4 | 1.3 |
| 02 | 238.1 | 2.08 | 232.4 | 9.0 | 1.1 |
| 03 | 144.6 | 2.00 | 125.2 | 9.0 | 0.8 |
| 04 | 164.3 | 2.01 | 125.1 | 8.0 | 0.9 |
| 05 | 24.6 | 1.95 | 31.3 | 8.8 | 1.1 |

**B.**

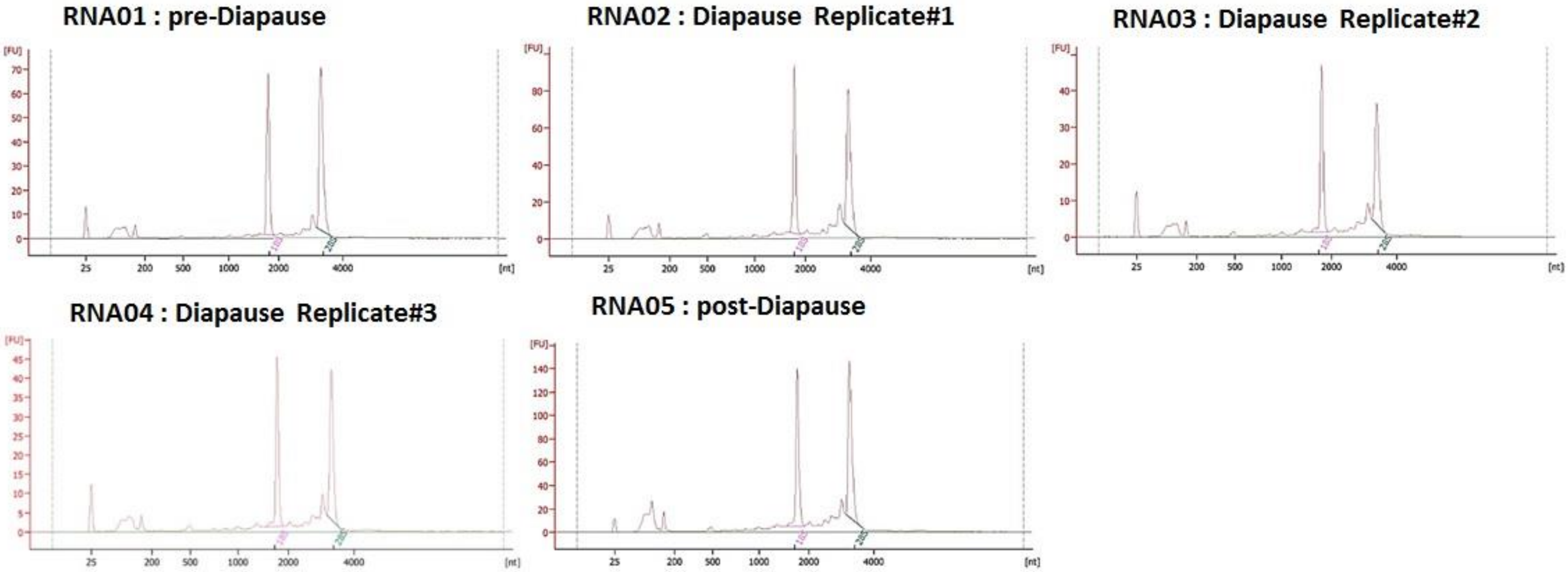

**Supplementary Fig. S2.**

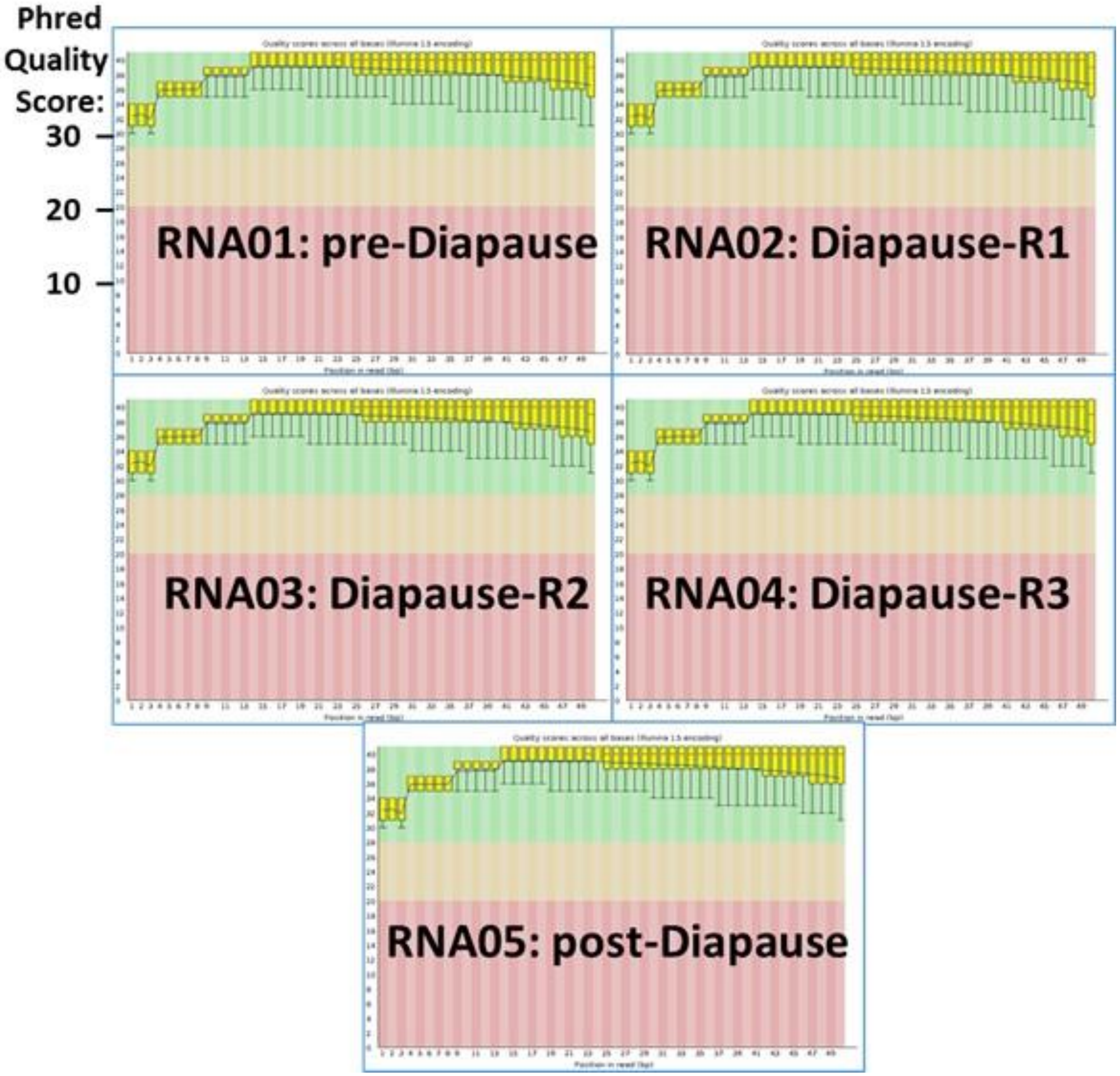

**Supplementary Fig. S3.**

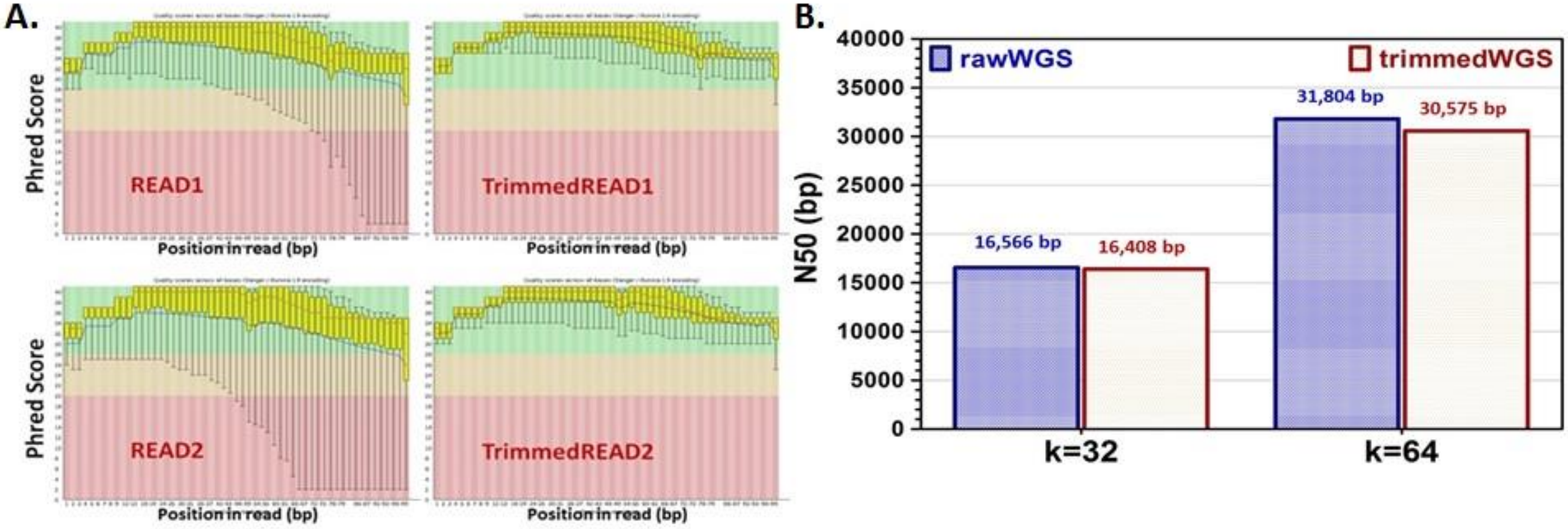

**Supplementary Fig. S4.**

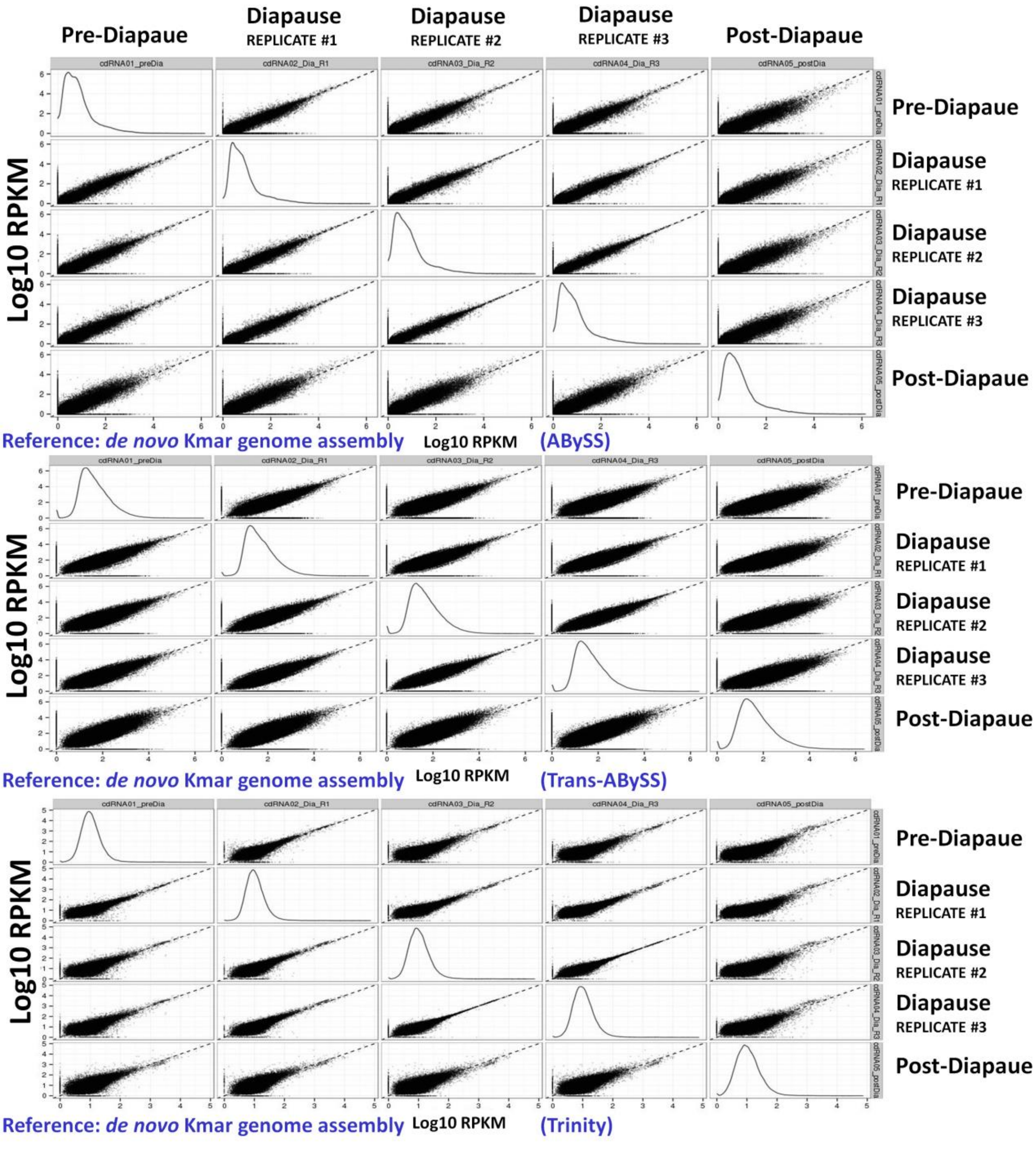

**Supplementary Fig. S5.**

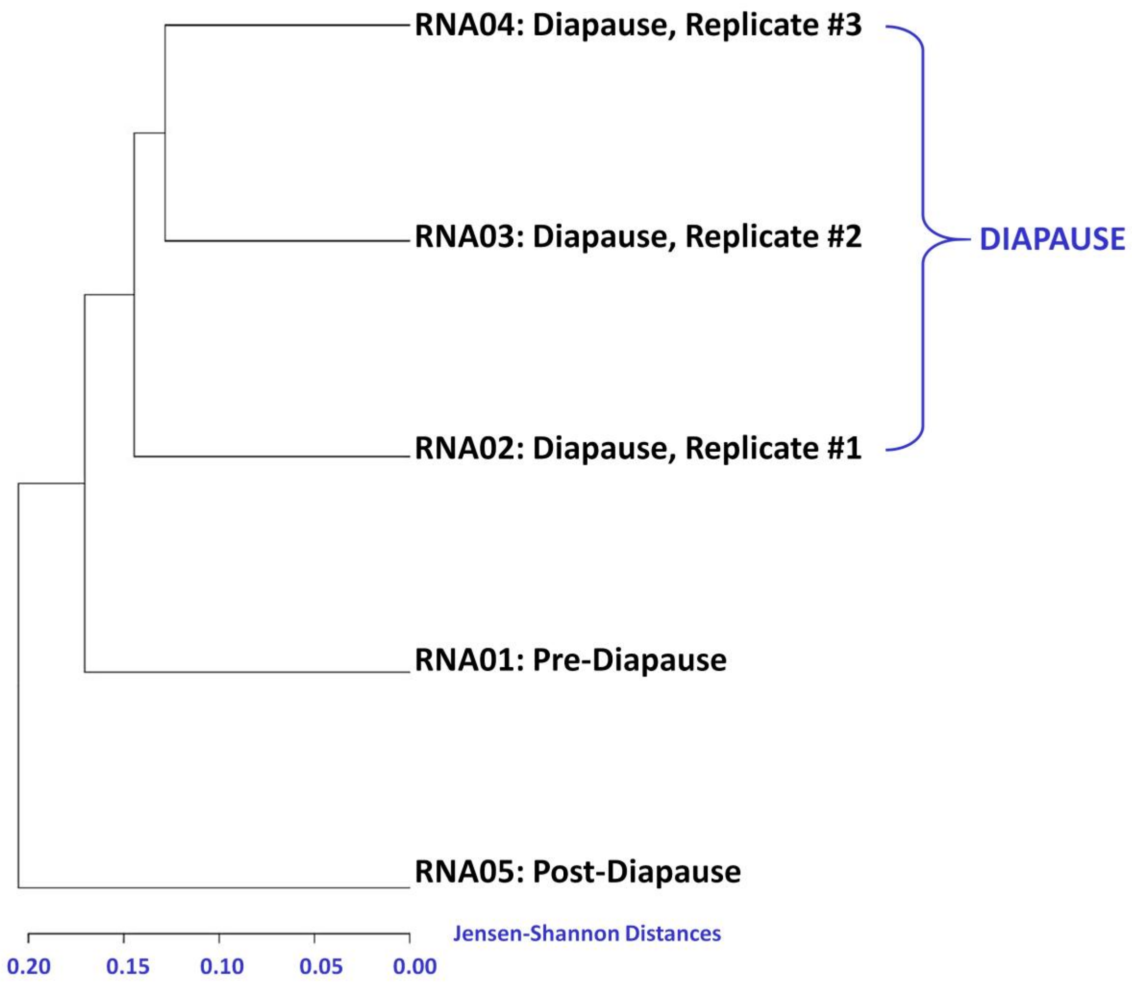

Supplement: Additional file 2: — Figure S1. Quantity and quality of Kmar pooled RNA samples. A high quality of the samples was obtained prior to RNA-Seq. (A) table showing RNA quantity and quality as measured by NanoDrop and Agilent bioanalyzer. [Note: RIN is an RNA integrity number from Agilent2100’s measurement.] (B) Chromatogram for each pooled RNA sample that shows high integrity for both 28S and 18S rRNA. A 500 ng subset of pooled RNA was used for RNA-Seq processing. Figure S2. Quality of RNA-Seq data. Graphs indicate the high quality of RNA-Seq data from each sample. Figure S3. Quality of Kmar pair-ended (read1 and read2) WGS data. (A) Graphs indicate quality of WGS reads before and after the trimming. (B) Both raw and trimmed WGS reads were assembled to build a de novo genome at k = 32 and k = 64. The graph shows a relatively similar N50 value achieved by either raw or trimmed WGS reads. Figure S4. Pairwise comparison between samples for all genome/transcriptome references. The scatter diagram shows a negative binomial data distribution for transcript abundance in all samples. The number of transcripts for ABySS is 67,374; Trans-ABySS is 206,747; and Trinity is 97,979. These scatter diagram indicate that most of the transcripts were present in all developmental stages of Kmar. Figure S5. Phylogenetics of the RNA-Seq data. The tree shows Jensen-Shannon distance analyses for the five RNA-Seq samples. Transcriptomes from all three diapause replicates clustered together and displayed a slightly closer distance to pre- than post-diapause. (PDF 729 kb) [file 12864_2015_2210_MOESM2_ESM.pdf]
